# Heterocyclic Cathinones as Inhibitors of Kynurenine Aminotransferase II—Design, Synthesis, and Evaluation

**DOI:** 10.3390/ph14121291

**Published:** 2021-12-10

**Authors:** Michal Maryška, Lucie Svobodová, Wim Dehaen, Martina Hrabinová, Michaela Rumlová, Ondřej Soukup, Martin Kuchař

**Affiliations:** 1 Forensic Laboratory of Biologically Active Substances, Department of Chemistry of Natural Compounds, Faculty of Food and Biochemical Technology, University of Chemistry and Technology Prague, Technická 5, 16628 Prague, Czech Republic; maryskam@vscht.cz (M.M.); svobodbl@vscht.cz (L.S.); 2 National Institute of Mental Health, Topolová 748, 250 67 Klecany, Czech Republic; 3 CZ-OPENSCREEN: National Infrastructure for Chemical Biology, Department of Informatics and Chemistry, Faculty of Chemical Technology, University of Chemistry and Technology Prague, Technická 5, 16628 Prague, Czech Republic; dehaeni@vscht.cz; 4 Biomedical Research Center, University Hospital Hradec Králové, Sokolská 581, 50005 Hradec Kralové, Czech Republic; martina.hrabinova@unob.cz (M.H.); ondrej.soukup@fnhk.cz (O.S.); 5 Department of Toxicology and Military Pharmacy, University of Defense, Třebešská 1575, 50005 Hradec Králové, Czech Republic; 6 Department of Biotechnology, Faculty of Food and Biochemical Technology, University of Chemistry and Technology Prague, Technická 5, 16628 Prague, Czech Republic; rumlovai@vscht.cz

**Keywords:** KAT-II, inhibitor, drug design, heterocyclic cathinones, enzyme assay

## Abstract

Kynurenic acid is a neuroprotective metabolite of tryptophan formed by kynurenine aminotransferase (KAT) catalyzed transformation of kynurenine. However, its high brain levels are associated with cognitive deficit and with the pathophysiology of schizophrenia. Although several classes of KAT inhibitors have been published, the search for new inhibitor chemotypes is crucial for the process of finding suitable clinical candidates. Therefore, we used pharmacophore modeling and molecular docking, which predicted derivatives of heterocyclic amino ketones as new potential irreversible inhibitors of kynurenine aminotransferase II. Thiazole and triazole-based amino ketones were synthesized within a SAR study and their inhibitory activities were evaluated in vitro. The observed activities confirmed our computational model and, moreover, the best compounds showed sub-micromolar inhibitory activity with 2-alaninoyl-5-(4-fluorophenyl)thiazole having IC_50_ = 0.097 µM.

## 1. Introduction

**Figure 1:**
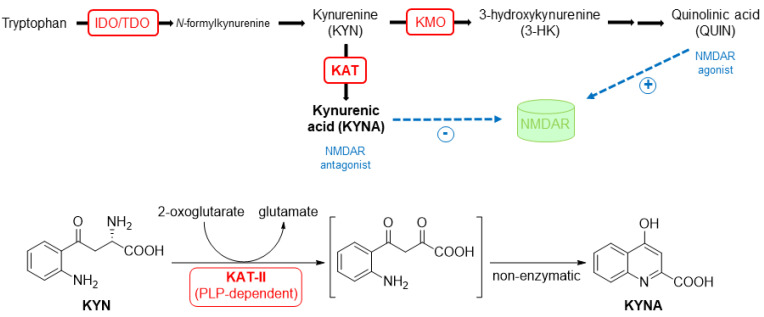
Schematic representation of the kynurenine pathway and the detailed description of KAT-II catalyzed transformation of kynurenine to kynurenic acid. IDO = indolamine-2,3-dioxygenase, TDO = tryptophan-2,3-dioxygenase, KMO = kynurenine-3-monooxygenase, KAT = kynurenine aminotransferase, NMDAR = *N*-methyl-d-aspartate receptor, KYN = kynurenine, KYNA = kynurenic acid, 3-HK = 3-hydroxykynurenine, QUIN = quinolinic acid, PLP = pyridoxal-5′-phosphate.

Kynurenic acid (KYNA) is a neuroactive metabolite produced in l-tryptophan catabolism, specifically in the kynurenine pathway (Figure 1). KYNA is known especially as an endogenous antagonist of *N*-methyl-d-aspartate receptor (NMDAR) and α7 nicotinic acetylcholine receptor (α7nAChR), and is considered to have a neuroprotective effect [1,2]. However, its high brain tissue levels are associated with imbalances in the glutamate, dopamine, and acetylcholine system [3]. Therefore, it can be related to the neurological disease schizophrenia, for which glutamatergic and dopaminergic hypoactivity is characteristic [4,5,6]. Concurrently, in the brain of patients with schizophrenia, higher levels of KYNA, compared to healthy individuals, have been demonstrated [7,8,9,10,11]. KYNA is formed via irreversible transamination from l-kynurenine and the reaction is catalyzed by a class of enzymes called kynurenine aminotransferases (KATs) [12]. From four known isoforms named KAT-I–IV, KAT-II is predominant in brain tissue and plays the primary role of KYNA biosynthesis in neurons [13,14]. Therefore, KAT-II is a good target for lowering brain levels of KYNA. Studies on mice have proven that the inhibition of KAT-II leads to improvement in cognitive functions, highlighting the potential use of KAT-II inhibitors in schizophrenia treatment [4,11,15].

**Figure 2:**
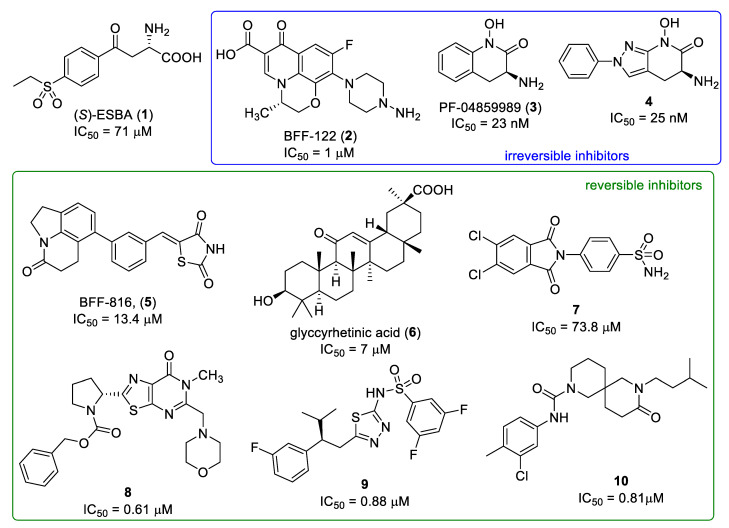
Examples of published KAT-II inhibitors.

Several KAT-II inhibitors have been published and some of them used as tool compounds for preclinical exploration. Early published inhibitors include (*S*)-ESBA (**1**) and BFF-122 (**2**) (Figure 2), both have been used for in vivo experiments on rodents [16,17,18] While **1** is a substrate-like competitive inhibitor, **2** acts as an irreversible inhibitor as it forms a covalent bond with the pyridoxal-5′-phosphate (PLP) cofactor. The most examined group of KAT-II inhibitors are hydroxamate derivatives from Pfizer with their lead compound PF-04859989 (**3**) (Figure 2) [19]. Compound **3** is a brain-penetrable, nanomolar irreversible inhibitor and its effects have been explored in numerous in vivo experiments [20,21,22,23,24,25,26,27]. However, this inhibitor suffers from poor pharmacokinetic properties due to its fast metabolism [28]. This issue was addressed in further research of hydroxamate derivatives [28,29,30,31,32,33], resulting in the discovery of compound **4** (Figure 2), the most effective irreversible KAT-II inhibitor known to date [32]. Some reversible KAT-II inhibitors have also been described including BFF-816 (**5**) [34], glycyrrhetinic acid (**6**) and its derivatives [35], compound **7** [36], and compound **8** (Figure 2) [37]. However, they often lack activity or the ability to penetrate the blood–brain barrier effectively, or there is limited information available (compound **8**). Recently, compound **9** and a class of 9-oxodiazaspiro[5,5]undecan-2-carboxamide derivatives (e.g., compound **10**) (Figure 2) were published as sub-micromolar reversible inhibitors of KAT-II [38], but, so far, there is no evidence of further examination in vivo.

Although there has been ongoing research of KAT-II inhibitors over the last few decades, none of the inhibitors advanced into clinical trials and the number of chemotypes remains rather low. Therefore, our objective was to discover a new class of KAT-II inhibitors using a structure-based drug discovery approach utilizing pharmacophore modelling and molecular docking. We designed and modeled thiazole- or triazole-based heterocyclic amino ketones as irreversible inhibitors of KAT-II and synthesized them to evaluate their inhibitory activity in vitro.

## 2. Results and Discussion

### 2.1. Pharmacophore Modeling and Design

**Figure 3:**
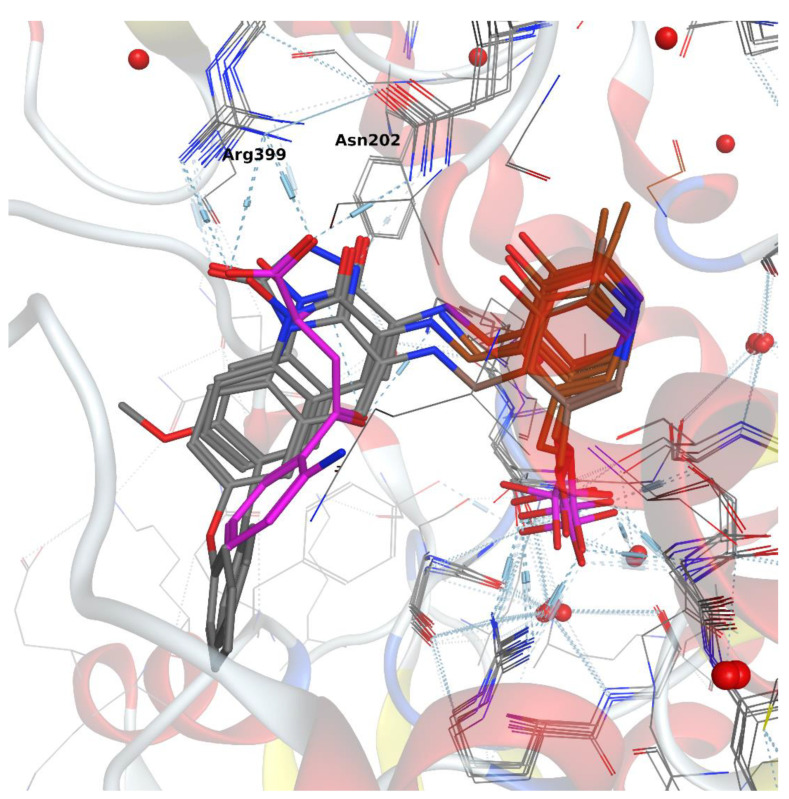
Overlay of inhibitors from pdb:4GDY, pdb:4UE8, pdb:4GE9, and pdb:2R2N inside of the KAT-II pocket. Kynurenine is shown in purple, and the PLP cofactor is shown in brown. Other inhibitors are shown in grey. Hydrogen bonding networks are shown as blue dotted lines.

**Figure 4:**
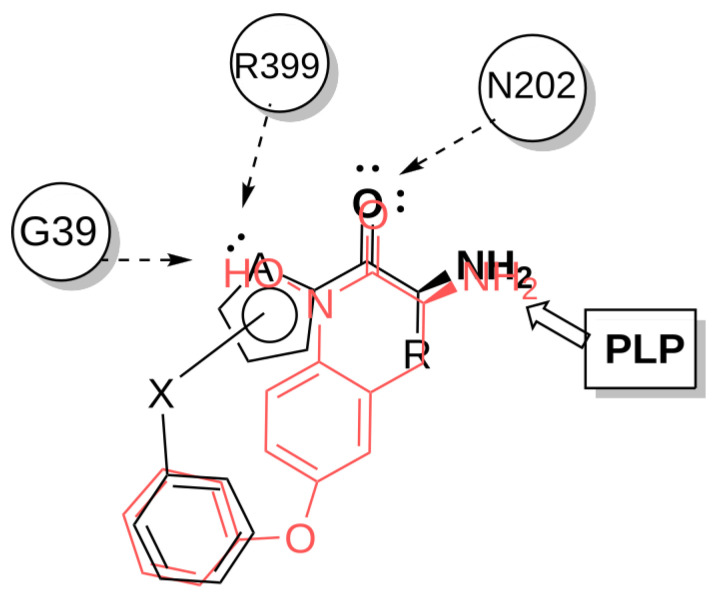
Heterocyclic α-amino ketones as a promising inhibitor class, showing the main interactions, from left to right: aromatic heteroatom accepting a H-bond from G39 or R399; ketone accepting a H-bond from N202; α-amino group in the vicinity of the PLP cofactor.

Using an overlay of crystallized KAT-II inhibitor–enzyme complexes, common features were selected, and a simple 3D pharmacophore was constructed (Figure 3). Important features were (1) an amino group that will form an imine with the PLP cofactor; (2) a hydrogen bond acceptor that will accept a hydrogen bond from Asn202; and (3) a hydrogen bond acceptor that will accept a hydrogen bond from Arg399 or a backbone H bond from the Gly-39 amine. Furthermore, a pocket around the inter-subunit interface of the KAT-II homodimer was found to typically accommodate an aromatic ring. With this knowledge in mind, a common substructure consisting of a heterocyclic α-amino ketone was designed to target these interactions (Figure 4).

**Figure 5:**
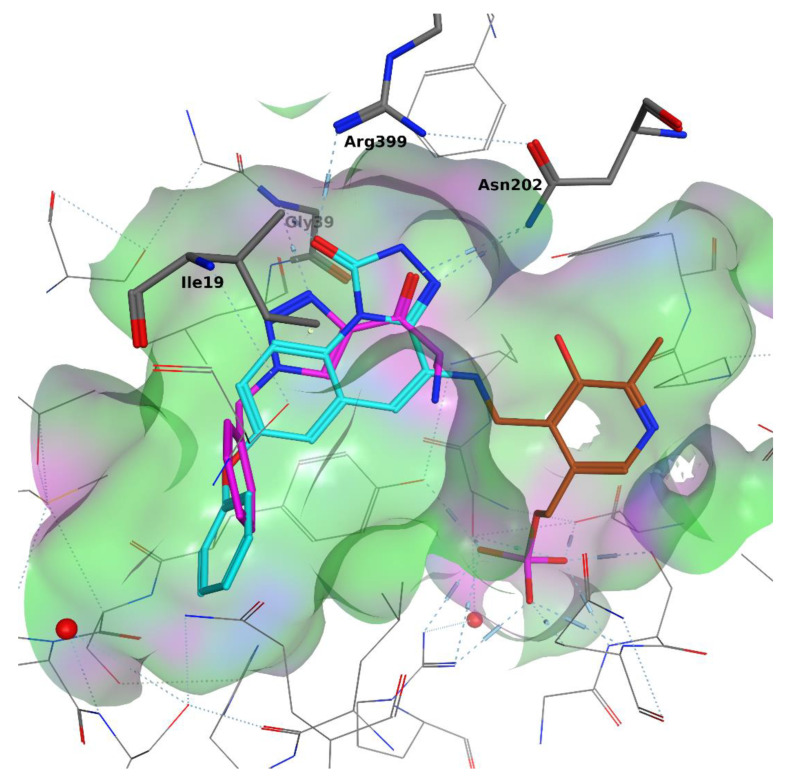
Structure of the representative triazole-based amino ketone (purple) in an overlay with the 4GDY ligand (cyan), which is covalently bound to the cofactor (brown) inside the KAT-II binding pocket. The triazole nitrogen binds to G39, but not to R399. The ketone oxygen binds to N202. An H–π interaction between the triazole π system and an Ile19 hydrogen is also visible. Coloring of the surface is purple: H-bonding, green: hydrophobic, and blue: mild polar. PF-04859989 (**3**) is shown in red to show the overlay of the equivalent functional groups.

A docking study was undertaken to validate the rationale behind the generated compounds in silico. The structure of KAT-II covalently bound to Pfizer’s tricyclic compound was employed because the hydrogen bonding patterns are most prominently visible (Figure 5). The docking results show that for heterocyclic α-amino ketones (also called heterocyclic cathinones), the ketone group accepts a hydrogen bond from Asn202 (overlaying with the carbonyl oxygen of PF-04859989 (**3**)) and the heterocycle nitrogen’s lone pair accepts a backbone hydrogen bond from Gly-39 (overlaying with the hydroxamate oxygen of **3**). Interestingly, an H–π interaction between the five-membered heterocycle and Ile-19 was detected in some docking poses. Finally, the α-amino functionality was found to be quite close to the nucleophilic amine site (at a distance of about 1 Å, depending on the compound).

### 2.2. Synthesis of In Silico Designed Amino Ketones

**Figure 6:**
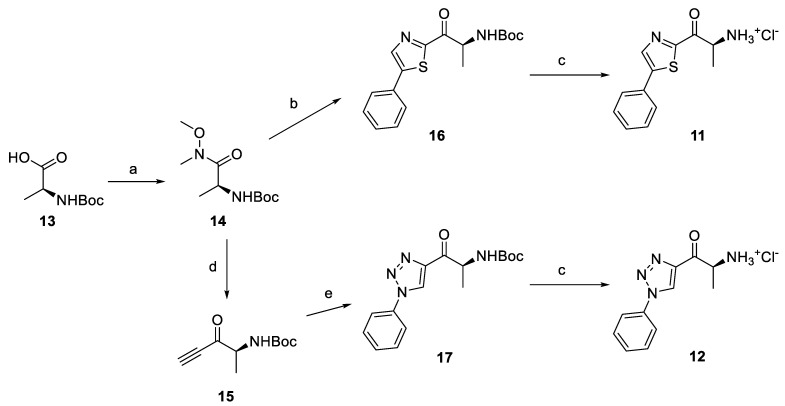
Scheme of the synthesis of alaninoyl triazole and thiazole derivatives. Reagents and conditions: (a) (i) carbonyldiimidazole (CDI), DCM, r.t., (ii) *N*,*O*-dimethylhydroxylamine hydrochloride, DCM, r.t.; (b) (i) 2-bromo-5-phenylthiazole, *i*-PrMgCl.LiCl, THF, −10 °C, (ii) **14**, *i*-PrMgCl.LiCl, −10 °C–r.t.; (c) AcCl, MeOH, r.t.; (d) (i) ethynylmagnesium bromide, THF, −78 °C, (ii) NaHSO_4_, H_2_O, 0 °C; and (e) phenyl azide, CuSO_4_.5H_2_O, sodium ascorbate, *t-*BuOH/H_2_O 1:1, r.t.

To confirm the activity of the designed chemotype, we first prepared 2-alaninoyl-5-phenylthiazole (**11**) and 4-alaninoyl-1-phenyl-1*H*-1,2,3-triazole (**12**). Thiazole and triazole fragments were chosen because of their good performance in docking studies and their chemical properties, as they commonly occur as fragments in medicinal chemistry. Both syntheses of **11** and **12** started from Boc-l-alanine (**13**), which was converted to the corresponding Weinreb amide **14** (Figure 6) [39]. The reaction of the prepared amide **14** with ethynylmagnesium bromide [40] or magnesiated 2-bromo-5-phenylthiazole [41] gave alkynone **15** and 2-acylated 5-phenylthiazole **16**, respectively. Thiazole **11** was obtained by deprotection in methanolic HCl, generated by the addition of acetyl chloride into a methanolic solution of **16**. Triazole intermediate **17** was prepared by the copper(I)-catalyzed alkyne-azide cycloaddition (CuAAC) reaction [42] with phenyl azide followed by deprotection to afford amino ketone **12**.

### 2.3. Inhibitory Activity of the Designed Lead Compounds

The inhibitory activity of amino ketones **11** and **12** was evaluated using a fluorimetric in vitro assay. The activity was determined as the percentage of inhibition (%*I*) at a concentration of 1 µmol/L and also as IC_50_ values (Table 1). The observed inhibitory activity confirmed the validity of the computational model and was suitable to conduct a synthesis-directed SAR study around the predicted heterocyclic α-amino ketone structure.

### 2.4. Synthesis of Derivatives for SAR Study

#### 2.4.1. Amino Ketone Derivatives with Variation of the Alkyl Chain

**Figure 7:**
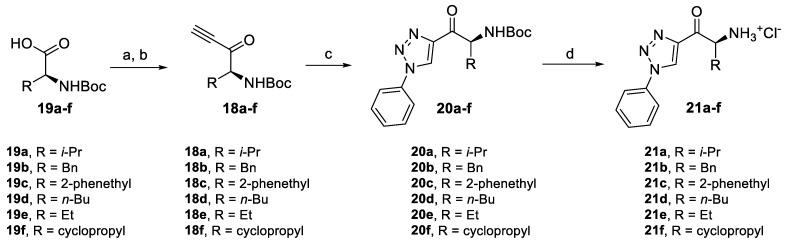
Synthesis of triazole derivatives with variation of the alkyl chain. Reagents and conditions: (a) (i) CDI, DCM, r.t., (ii) *N*,*O*-dimethylhydroxylamine hydrochloride, DCM, r.t., 91–100%; (b) (i) ethynylmagnesium bromide, THF, −78 °C, (ii) NaHSO_4_, H_2_O, 0 °C, 86–95%; (c) phenyl azide, CuSO_4_.5H_2_O, sodium ascorbate, *t-*BuOH/H_2_O 1:1, r.t., 83–94%; and (d) AcCl, MeOH, r.t., 94–97%.

First, we prepared a series of derivatives based on thiazole and triazole with variation of the amino ketone sidechain. For triazole derivatives, the same synthetic approach was used as for the preparation of **12**, altering only the starting Boc-amino acids (Figure 7). Alkynones **18a–f** derived from amino acids **19a–f** were prepared and converted via CuAAC reaction to corresponding triazoles **20a–f**. After subsequent deprotection, amino ketones **21a–f** were obtained in very high yields.

**Figure 8:**
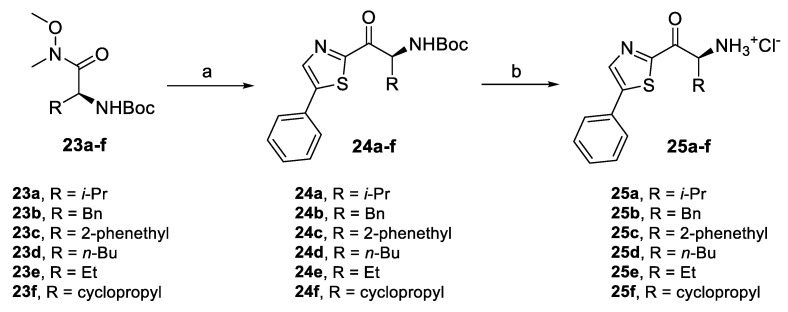
Synthesis of thiazole derivatives with variation of the alkyl chain. Reagents and conditions: (a) (i) 5-phenylthiazole (**22**), *n*-BuLi, THF, −78 °C, (ii) **23a–f**, *n*-BuLi, −78 °C, 67–78%, and (b) AcCl, MeOH, r.t., 69–93%.

In the same way, a series of thiazole derivatives with a side chain variation was synthesized. A similar synthetic approach as for the preparation of **11** was used, but with different reaction conditions used for the acylation step (Figure 8). Lithiation of 5-phenylthiazole (**22**) at −78 °C and subsequent acylation with Weinreb amides **23a–f** at the same temperature allowed for the preparation of acylated thiazoles **24a–f** in higher yields than in the synthesis of **11** and without the formation of intensively colored by-products, leading to easier purification. As in the case of the previous Boc-amino ketones, acidic deprotection of **24a–f** gave amino ketones **25a–f** in great yields.

#### 2.4.2. Triazole Derivatives with Variation of Aryl Substituent

Furthermore, we explored the effect of aryl substitution on the inhibitory activity within the series of triazole-based amino ketones **26a–v**. We used the same synthetic approach as for previous triazole derivatives using CuAAC of respective aryl azides with alkynon **15** (Table 2) to prepare Boc-protected triazole derivatives **27a–v**. Based on the available precursors, two methods for the synthesis of aryl azides were employed–Cu catalyzed azidation of haloarenes (Method A) [43] or diazotation of anilines followed by substitution by azide (Method B) [44]. In both cases, crude azides were used for CuAAC without any purification. As we discovered, azido pyridines were shown not to be compatible with the aqueous conditions used for the CuAAC used in the previous preparations of triazole derivatives. Therefore, we used a different CuAAC method, employing copper(I) thiophene-2-carboxylate (CuTC) as a catalyst in toluene [45]. Moreover, this method had another advantage in the handling with aryl azides in the previous step–if the azides were extracted with toluene, then these crude extracts could be used directly in the CuAAC reaction.

#### 2.4.3. Triazole Derivatives with Alkyl Linker

**Figure 9:**
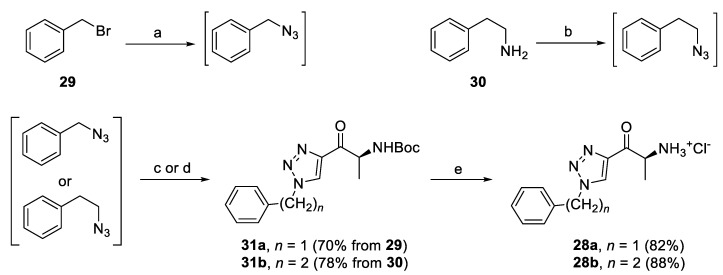
Synthesis of triazole derivatives with variation of the alkyl linker. Reagents and conditions: (a) NaN_3_, acetone/H_2_O, r.t.; (b) ADMP, 4-(dimethylamino)pyridine (DMAP), DCM, 30 °C; (c) **15**, CuSO_4_.5H_2_O, sodium ascorbate, *t-*BuOH/H_2_O 1:1, r.t.; (d) **15**, CuTC, toluene, r.t.; and (e) AcCl, MeOH, r.t.

The effect of a short hydrocarbon linker between the phenyl ring and the heterocycle was also examined. Triazole derivatives **28a,b** with benzyl and 2-phenethyl substituent were prepared using the same reaction sequence as above-mentioned (Figure 9). The corresponding azides were prepared by nucleophilic substitution of benzyl bromide (**29**) and by diazotransfer to phenethylamine (**30**) from ADMP (2-azido-1,3-dimethylimidazolinium hexafluorophosphate), respectively (Figure 9) [46].

### 2.5. Evaluation of Inhibitory Activity of SAR Derivatives

The obtained inhibition data for the series of triazole derivatives with alkyl chain variation (**21a–f)**, in comparison with the lead compound **12**, suggested that any variation in the alkyl chain led to a decrease in activity (Table 3, entry 1–6). Within the examined substituents, the ethyl-substituted derivative **21e** showed reasonable activity (Table 3, entry 5), but larger groups had a greater influence on the decrease in the activity, and cyclopropyl was the least suitable (Table 3, entry 6). A similar trend was also observed in the series of thiazole derivatives **25a–f** (Table 3, entry 7–12), but in this case, the ethyl-derivative **25e** showed even slightly higher inhibitory activity (Table 3, entry 11) than the methyl-derivative **11**.

It was also found that the short alkyl linker had no positive effect on inhibitory activity (Table 3, entry 13–14). The values of %*I* were lower for both derivative **28a** and **28b**, in comparison with **12**.

From the inhibition data obtained for the series of triazole derivatives with aryl modification **26a–v** (Table 3, entry 15–36), it was found that *para*-substitution with electron withdrawing groups led to the best results. Derivatives with 4-fluoro (**26n**) and 4-nitro (**26q**) groups had the highest inhibitory activity (Table 3, entries 28 and 31), followed by the 3-nitro derivative **26p** (Table 3, entry 30), which was the most active among the other *meta*-substituted derivatives (Table 3, entries 16, 26, 30, and 33). In contrast to these results, the use of electron donating or sterically demanding groups led to a decrease in inhibitory activity (Table 3, entries 18, 19, 23, 24, 29, and 32). The only and significant exception was the use of the 4-methoxy group with derivative **26a** belonging among the most active compounds. The explanation for this compound’s activity is not straightforward, as this goes against the general trend. It is not related to ring electronics generally, as the ortho and meta isomer of **26a** showed reduced activity (Table 3, entries 16 and 17). One explanation we considered is hydrogen bonding, but inspection of the docking pose of derivative **26a** did not show any hydrogen bonding. Therefore, we speculate a sterically favorable orientation may be a better explanation. However, only follow-up measurements can confirm this hypothesis. Interesting values of %*I* were also measured for 2,4-dimethyl and 2-chloro derivatives **26g** and **26t** (Table 3, entries 21 and 34). However, use of 2,6-dimethyl and 3,4-dimethyl groups led to a greater decrease in activity, as in the case of other 3,4-disubstituted derivatives (Table 3, entries 20, 22, 35, and 36).

Although some structure–activity trends between substitution patterns on synthesized derivatives and experimentally measured activity are clearly present within the examined group of derivatives, none of them showed increased inhibitory activity in comparison to compound **12**. Only derivatives **26a, 26g, 26n, 26p–q** retained comparable potency. Therefore, the best performing aryl substituent (4-fluorophenyl) was further examined with thiazole-based derivatives.

### 2.6. Synthesis of Optimized Inhibitors Based on SAR Evaluation

**Figure 10:**
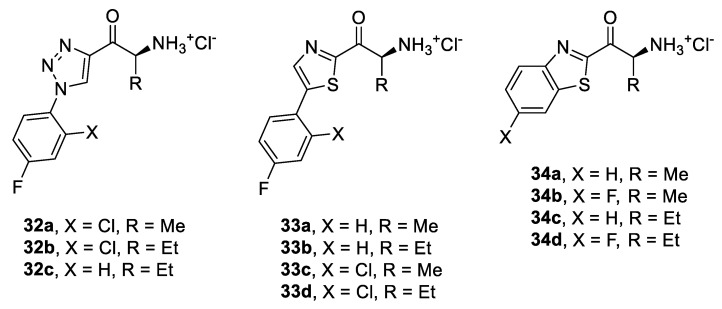
Structures of inhibitors prepared based on SAR optimization.

Based on the obtained inhibition data from SAR, we chose the best fragments and combined them to design a series of optimized amino ketones **32a–c** and **33a–d** (Figure 10). Methyl and ethyl were chosen as the most suitable alkyl side chains together with 4-fluorophenyl as the best aryl substituent. The combination of 4-fluoro and 2-chloro substitution was also examined, assuming there may be an additive positive contribution by both groups. In addition, benzo[*d*]thiazole derivatives **34a–d** were prepared since this motif could not be explored in the triazole-based series because the aromatic nitrogen atom cannot accommodate a merged ring.

**Figure 11:**
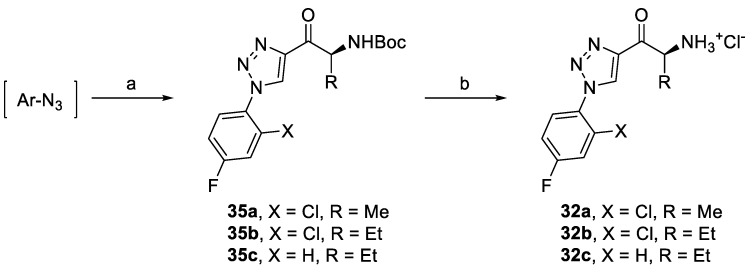
Synthesis of triazole derivatives based on SAR. Reagents and conditions: (a) **15** or **18e**, CuTC, toluene, r.t., 79–83%, and (b) AcCl, MeOH, 0 °C–r.t., 65–90%.

**Figure 12:**
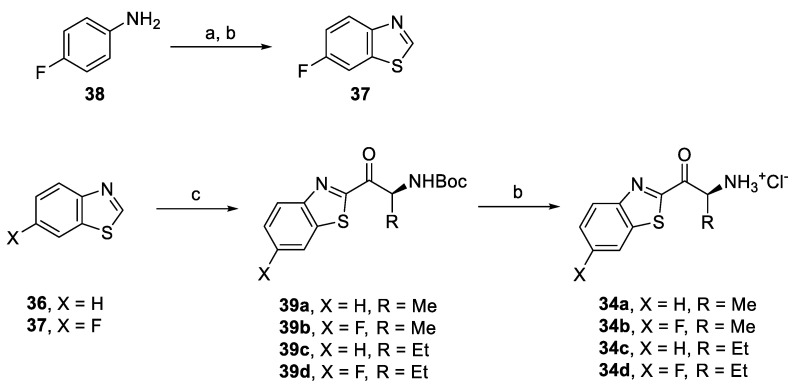
Synthesis of triazole derivatives based on SAR. Reagents and conditions: (a) KSCN, Br_2_, AcOH, r.t., 79%; (b) *i-*AmONO, THF, reflux; 72%, (c) **14** or **23e**, *i-*PrMgCl.LiCl, −10 °C to r.t., 79–83%; and (d) AcCl, MeOH, 0 °C–r.t., 80–91%.

Triazole derivatives **32a–c** were prepared from corresponding aryl azides and alkynones **15** or **18e** (Figure 11) and by subsequent deprotection of Boc-intermediates **35a–c**. Benzothiazole derivatives **34a–d** were synthesized by acylation of either commercially available benzo[*d*]thiazole (**36**) or 6-fluorobenzo[*d*]thiazole (**37**) obtained in two steps from 4-fluoroaniline (**38**) (Figure 12) [47,48]. Deprotection of Boc-intermediates **39a–d** gave amino ketones **34a–d**.

**Figure 13:**
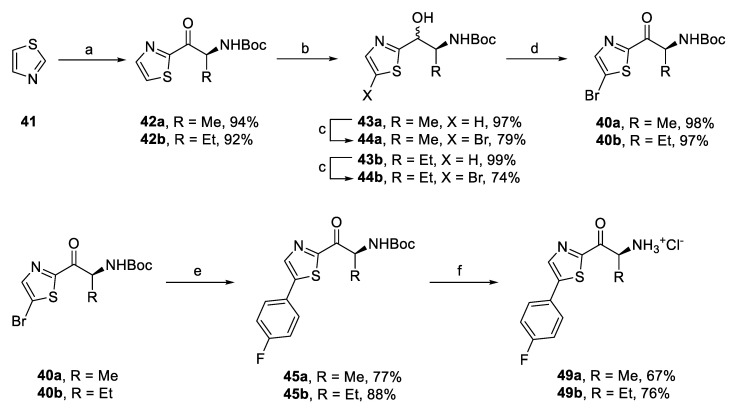
Synthesis of aryl-thiazole derivatives based on SAR. Reagents and conditions: (a) **14** or **23e**, *i-*PrMgCl.LiCl, −10 °C–r.t.; (b) NaBH_4_, MeOH/THF, 0 °C–r.t.; (c) NBS, DMF, 0–50 °C; (d) DMP, DCM, r.t.; (e) (4-fluorophenyl)boronic acid, XPhos Pd G2, K_3_PO_4_, THF/H_2_O, 40 °C; and (f) AcCl, MeOH, 0 °C–r.t.

**Figure 14:**
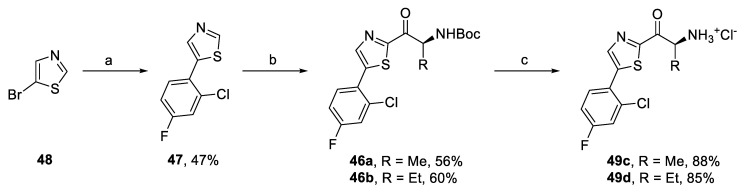
Synthesis of 2-chloro-4-fluorophenyl derivatives **49c,d**. Reagents and conditions: (a) 2-chloro-4-fluorophenyl)boronic acid, Pd(PPh_3_)_4_, K_2_CO_3_, DME/H_2_O, reflux; (b) (i) *n*-BuLi, THF, -78 C, (ii) **14** or **23e**, *i-*PrMgCl.LiCl, −78 °C; and (c) AcCl, MeOH, 0 °C–r.t.

For the series of thiazole-based derivatives, two synthetic approaches were employed. First, bromo derivatives **40a,b** were prepared in high overall yields of 71% and 65% from thiazole (**41**) by a sequence of four steps: acylation, reduction of the carbonyl group, bromination, and oxidation of the alcohol group (Figure 13). Intermediates **40a,b** were then reacted in Suzuki coupling with (4-fluorophenyl)boronic acid [49], giving aryl derivatives **45a,b** in good yields (Figure 13). Contrary to these results, the use of (2-chloro-4-fluorophenyl)boronic acid did not lead to the expected coupling products **46a,b**. Therefore, we prepared corresponding aryl thiazole **47** by the Suzuki coupling of 5-bromothiazole (**48**) and 2-chloro-4-fluorophenyl)boronic acid (Figure 14). Compound **47** was then converted to thiazole derivatives **46a,b** by acylation using the conditions described earlier. Subsequent Boc deprotection gave amino ketones **49a–d** (Figure 13 and Figure 14).

### 2.7. Inhibitory Activity of Structurally Optimized Derivatives

Within the structurally optimized compounds, the best inhibitory activity was observed in the case of fluoro-derivative **49a** (Table 4, entry 8). The benzo[*d*]thiazole motif proved to be active, with derivatives **34a** and **34c** having similar or higher activity as compounds **11** and **25e**, and fluorinated analogue **34b** being even more potent (Table 4, entries 4, 6 and 5). Although we could not explain the substantially lower inhibitory activity for derivative **34d** (Table 4, entry 7), similar derivatives showed the observed activity in accordance with the estimated SAR findings. Unexpectedly, combination of 4-fluoro and 2-chloro substituents had a different effect for triazole and thiazole derivatives. Compounds **32a,b** performed worse than the derivatives **26n** and **26t** (Table 3, entry 1–2). On the other hand, inhibitory activity of thiazole derivatives **49c,d** was higher in comparison to compound **11** (Table 4, entries 10–11). Moreover, derivative **49d** showed comparable inhibitory activity as derivative **49a**, making the two of them the best inhibitors from our study with sub-micromolar IC_50_ values.

## 3. Materials and Methods

### 3.1. Computational Methods

#### 3.1.1. Pharmacophore Modeling

An overlay of inhibitors was generated based on the selection of the available inhibitor-enzyme structures. The used structures were pdb:4GDY, pdb:4UE8, pdb:4GE9, and pdb:2R2N. The Pharmacophore Builder tool of the Molecular Operating Environment [50] software package was subsequently used to hand-select common features apparent in the overlay. Prospective compounds were screened by performing a pharmacophore search. Conformations for screened compounds were prepared using the Conformational Search tool. A stochastic search with a maximal conformation limit of 1000 and a high energy window (50 kcal/mol) and otherwise standard settings was used so a large amount of conformations would be sampled for the pharmacophore search.

#### 3.1.2. Docking

All ligands were docked to pdb:4GDY, a structure of a cofactor-bound tricyclic KAT inhibitor bound to KAT, using the docking algorithm of the Molecular Operating Environment [50] software package. The docking was performed under standard settings: the used forcefield was MMFF94x, placement was made by the Triangle Matcher and scored using the London dG scoring function, retaining 100 poses. Refinement was made under induced fit settings, using the GBVI/WSA dG scoring function, retaining 20 poses. Compounds were pre-prepared for docking by protonation at physiological pH and energy minimization with an MMFF94x forcefield.

### 3.2. Enzyme Production and Purification

The nucleotide sequence encoding *h*KAT-II gene was constructed by Invitrogen, Thermo Fisher Scientific. The *h*KAT-II gene, optimized for heterologous expression in *E. coli,* was cloned into a pET15b vector using *Nde*I-*Xho*I. The *h*KAT-II protein, carrying an *N*-terminal hexahistidine tag, was produced in *E.coli* BL21(DE3)-RIL cells for 4 h at 37 °C. The bacterial cells were then harvested and disintegrated using One-Shot. Low-speed (25.000× *g*, 30 min) centrifugation cleared cell lysate containing *h*KAT-II was loaded on the top of a HisTrap Fast Flow column (Cytiva) equilibrated in loading buffer (40 mM Tris, pH 8; 1000 mM NaCl, 40 µM PLP, 5 mM MgCl_2_, 20 mM imidazole). Following elution of the bound proteins by the loading buffer containing 600 mM imidazole, purified *h*KAT-II was dialyzed into a storage buffer (20 mM Hepes pH 7.5; 50 mM NaCl, 70 µM PLP, 10% glycerol), concentrated to 1.0–1.5 mg/mL, aliquoted, and stored at −80 °C.

### 3.3. Activity Assay of hKAT-II

*h*KAT-II activity was measured using a fluorescence assay utilizing l*-*α-aminoadipic acid (AAD) and α-ketoglutarate as the substrates as previously described [51]. The assay medium (100 µL) consisted of 0.75 µM of *h*KAT-II, 0.3 mM of AAD, 50 µM α-ketoglutarate, 3 mM NAD^+^, 88 µg/mL of glutamic dehydrogenase, and 5 µM pyridoxal phosphate (PLP) in a 0.1 M phosphate buffer pH 7.5. The activity of the enzyme was assessed on a Spark multiple mode reader (Tecan Group Ltd., Männedorf, Switzerland) with an excitation wavelength of 340 nm and emission wavelength of 390 nm at 37 °C. The plate was shaken for 20 s before reading. The kinetic interval was set at 30 s between each reading, and data were collected for 30 min. The activity of the enzyme was assayed in pentaplicate in each measurement (*n* = 3). Kinetic data were then collected over a period of 30 min, and the linear part of the initial velocity was used to calculate the enzyme activity expressed as the slope of the linearized curve via the linear regression function (Excel, Microsoft, USA).

### 3.4. Coupled Fluorescence In Vitro Inhibition Assay

The inhibitory activity of the tested compounds was determined via 30 min pre-incubation with the enzyme at various concentrations of the inhibitor at room temperature in a 0.1 M phosphate buffer pH 7.5 with 5 µM PLP. The enzyme reaction was initiated by the addition of a substrate and cofactors. The final mixture contained 20 nM of *h*KAT-II, 0.3 mM of AAD, 50 µM α-ketoglutarate, 3 mM NAD^+^, and 88 µg/mL of glutamic dehydrogenase. Kinetic data were collected over a period of 30 min, and the slope of the linearized curve was calculated as above. The inhibitory activity in %*I* at a given concentration was calculated for novel compounds using the following equation:

(1) $$\mathrm{Inhibition}\;\left[\%I\right]=\left(\frac{V_{0-}\;V_{i}}{V_{0}}\right)\times 100,$$

where *V*_0_ and *V*_i_ are the activity of the enzyme in the absence and presence of the inhibitor, respectively. All the data were obtained in pentaplicate for each measurement (*n* = 3). Alternatively, the IC_50_ values for selected novel compounds were assessed. GraphPad Prism 8 software (San Diego, USA) was used for the IC_50_ value calculation using non-linear regression (four parameters). PF-04859989 (**3**) was used as the reference inhibitor to validate the assay. The obtained IC_50_ value for the reference inhibitor **3** (IC_50_ = 28 ± 5 nM) was in agreement with the published data [19,51].

### 3.5. Chemistry

All commercially available chemicals and reagents were purchased from either Sigma-Aldrich (Merck) (Darmstadt, Germany), Fluorochem (Hadfield, United Kingdom), TCI (Zwijndrecht, Belgium), or ABCR (Karlsruhe, Germany), and were used without any further purification. The reference inhibitor PF-04859989 (**3**) was synthesized according to the published procedure [51]. Anhydrous solvents were prepared by drying with molecular sieves. All reactions were carried out under an argon atmosphere. Thin layer chromatography (TLC) was performed on aluminum backed sheets coated with 60F 254 silica gel from Merck (Darmstadt, Germany). Flash chromatography was performed on a CombiFlash Rf 200 apparatus (Teledyne ISCO, Lincoln, NE, USA) using either silica gel (45–200 μm) from Merck, or prepacked Redisep Rf Gold columns (packed with silica gel). NMR spectra were recorded on Agilent 400-MR DD2 (400 MHz for ^1^H; 101 MHz for ^13^C) or Varian Gemini 300 (300 MHz for ^1^H; 282 MHz for ^19^F) spectrometers. Chemical shifts *δ* were reported in ppm and referenced to residual peaks of NMR solvents (^1^H, ^13^C): CDCl_3_–7.26 (^1^H), 77.0 (^13^C); DMSO-d6–2.50 (^1^H), 39.5 (^13^C); CD_3_OD–3.31 (^1^H), 49.0 (^13^C). Chemical shifts in ^19^F spectra were referenced to the peak of CFCl_3_ (0.0 ppm). High resolution mass spectra were measured on an Agilent 6550 iFunnel Q-TOF (Agilent, Santa Clara, CA, USA) or LTQ Orbitrap Velos (Thermo Fischer Scientific, Waltham, MA, USA) spectrometer using ESI ionization. Melting points were measured using a PHMK 78/1742 VEB Analytik Dresden apparatus (Kofler type) and were uncorrected. The purity of all compounds used for biochemical testing was 95% or higher, as determined by HPLC/UV on Agilent 1290 Infinity LC (Agilent, Santa Clara, CA, USA ).

Detailed procedures and characterization of the synthesized compounds together with copies of the NMR spectra are available in the Supplementary Materials related to this manuscript. Analytical data of compounds **14** [52], **23a** [53], **23b** [52], **23c** [54], **23d** [54], **23e** [54], **23f** [55], **15** [56], **18a** [57], **18b** [40], **22** [58], **24b** [59] and **42a** [60] were in agreement with those previously reported.

#### 3.5.1. General Procedure 1 (GP1): Synthesis of Weinreb Amides from Boc-l-Amino Acids

To the solution of Boc-l-amino acid (1.0 eq.) in dry DCM (2.5 mL/mmol) was added CDI (1.1 eq.) portionwise and the mixture was stirred at r.t. for 1.25 h. Solid *N*,*O*-dimethylhydroxylamine hydrochloride (1.11 eq.) was then added and the suspension was stirred overnight. DCM was evaporated and the residue was partitioned between EtOAc and 1 M HCl. The organic phase was washed once more with 1 M HCl, followed by saturated NaHCO_3_ (2×), and brine, and was dried by MgSO_4_. Evaporation of the solvent gave pure Weinreb amides.

#### 3.5.2. General Procedure 2 (GP2): Synthesis of Alkynones from Weinreb Amides

The solution of Weinreb amide (1.0 eq.) in dry THF (4 mL/mmol) was cooled to −78 °C, followed by dropwise addition of ethynylmagnesium bromide (4.0 eq., 0.5M solution in THF). The resulting solution was stirred at −78 °C for 1 h and then at r.t. overnight. The mixture was then poured into an ice-cold 1 M aqueous NaHSO_4_ (16 mL/mmol) and the biphasic mixture was stirred for 1 h at 0 °C. Most of the THF was evaporated and the aqueous layer was extracted by Et_2_O (3 × 15 mL/mmol). The combined organic phases were washed with 1 M NaHSO_4_, saturated NaHCO_3_, and brine, and dried by MgSO_4_. The crude product was purified by flash chromatography (SiO_2_, 15–35% EtOAc/hexane), yielding the corresponding alkynones.

#### 3.5.3. General Procedure 3 (GP3): Acylation of Thiazoles and Benzo[*d*]thiazoles with Weinreb Amides

Step 1: The thiazole derivative (1.5 eq.) was dissolved in dry THF (2 mL/mmol) and the solution was cooled either to −10 °C or −78 °C. Then, a solution of *i-*PrMgCl.LiCl (1.5 eq. 1.3 M solution in THF) or *n*-BuLi (1.5 eq, 2.5 M solution in hexane) was added dropwise and the resulting mixture was stirred at −10 °C (if *i-*PrMgCl.LiCl was used) or at −78 °C (if *n*-BuLi was used) for 1–2 h.

Step 2: The suspension/solution of Weinreb amide (1.0 eq.) in dry THF (2 mL/mmol) was cooled to −10 °C or −78 °C, and *i-*PrMgCl.LiCl (1.0 eq. 1.3 M solution in THF) or *n*-BuLi (1.0 eq, 2.5 M solution in hexane) was added dropwise. The resulting mixture was stirred for 10 min and then transferred into the solution of metallated thiazole from step 1. The reaction mixture was stirred for 15 min at −10 °C and then at r.t. overnight (if *i-*PrMgCl.LiCl was used) or was stirred 3–6 h at −78 °C (if *n*-BuLi was used). The mixture was quenched with saturated NH_4_Cl, diluted with water, and extracted with EtOAc (2 × 10 mL/mmol). The combined organic phases were washed with brine and dried by MgSO_4_. Purification of crude products by flash chromatography (SiO_2_, EtOAc/hexane, or Et_2_O/hexane) gave acylated thiazole or benzo[*d*]thiazole derivatives.

#### 3.5.4. General Procedure 4 (GP4): Synthesis of Aryl Azides

*Method A* (GP4A): Concentrated HCl (0.2 mL/mmol) was added to the mixture of the aniline derivative (1.0 eq.) and water (2.2 mL/mmol), and the resulting solution was cooled with an ice bath, followed by dropwise addition of NaNO_2_ (1.0 eq., 5 M aqueous solution). After 30 min, the mixture was neutralized with a cold saturated NaHCO_3_ solution and then, still with cooling, NaN_3_ (1.0 eq., 4M aqueous solution) was slowly added. The cooling bath was removed, and the mixture was stirred vigorously for 1 h at r.t. The mixture was extracted with toluene (6 mL/mmol), and the organic phase was washed with saturated NaHCO_3_, and brine, and dried by MgSO_4_. The obtained toluene solutions of crude azides were used directly in the next step.

*Method B* (GP4B): Degassed EtOH/H_2_O (7:3, 4 mL/mmol), followed by DMEDA (0.15 eq.) was added to the mixture of aryl bromide or aryl iodide (1.0 eq.), NaN_3_ (2.0 eq.), CuI (0.1 eq.), and sodium ascorbate (0.05 eq.), and the resulting mixture was refluxed under argon for 1.5 h (aryl bromide) or 40 min (aryl iodide). After cooling, the mixture was diluted with water and extracted with hexane (3 × 10 mL/mmol). The combined organic phases were washed with brine and dried by MgSO_4_. Careful removal of hexane on a rotavap (30 °C water bath) gave crude azides, which were used directly in the next step. Alternatively, the crude mixture was extracted by toluene and used directly in the next step.

#### 3.5.5. General Procedure 5 (GP5): Synthesis of Triazole Derivatives by CuAAC

*Method A* (GP5A): *t-*BuOH/H_2_O (1:1, 5 mL/mmol) was added to the mixture of crude azide (1.0 eq.) and alkynone (1.0 eq.), followed by sodium ascorbate (0.1 eq., 1 M aqueous solution), and CuSO_4_.5H_2_O (0.01 eq., 50 mg/mL aqueous solution). The mixture was stirred under argon at r.t. overnight. The suspension was diluted with ice-cold water and filtered. Solids were washed several times with water and vacuum dried. Trituration of the crude product with hexane gave pure triazoles. If no precipitation occurred after dilution with water, the mixture was extracted with EtOAc (3 × 15 mL/mmol) and the combined organic phases were washed with brine (2×) and dried by MgSO_4_. The crude product was purified by flash chromatography (SiO_2_, EtOAc/hexane).

*Method B* (GP5B): Alkynone (1.0 eq.) and crude azide (1.0 eq.) were dissolved in toluene (10 mL/mmol) (or the solution of crude azide in toluene from previous step was used), followed by the addition of CuTC (0.1 eq.) and the resulting mixture was stirred under argon overnight. The toluene was evaporated, and the residue was partitioned between DCM (20 mL/mmol) and saturated aqueous NH_4_Cl. The aqueous phase was further extracted with DCM (2 × 20 mL/mmol) and the combined organic phases were washed with brine and dried by MgSO_4_. The crude product was purified by flash chromatography (SiO_2_, EtOAc/hexane), followed by precipitation from the hexane (if needed).

#### 3.5.6. General Procedure 6 (GP6): Suzuki Coupling of 2-acyl-5-bromothiazoles

Degassed THF/H_2_O (4:1, 10 mL/mmol) was added to the mixture of the bromothiazole derivative (1.0 eq), (4-fluorophenyl)boronic acid (1.1 eq), XPhos Pd G2 (0.03 eq.), and K_3_PO_4_ (2.0 eq.), and the resulting mixture was stirred under an argon atmosphere at 40 °C. To achieve full conversion of the starting bromothiazole, additional (4-fluorophenyl)boronic (0.5 eq.) was added a few times in 2–3 h intervals (progress was monitored by TLC). Then, the reaction mixture was diluted with saturated aqueous NH_4_Cl and extracted with EtOAc (3 × 20 mL/mmol). The combined organic phases were washed with brine and dried by MgSO_4_. The crude products were purified by flash chromatography (SiO_2_, 10–25% EtOAc/hexane).

#### 3.5.7. General Procedure 7 (GP7): Synthesis of Heterocyclic Cathinones by Deprotection of the Boc Protecting Group

Acetyl chloride (8.0 eq.) was added dropwise to a solution/suspension of Boc-derivative (1.0 eq.) in dry MeOH (4 mL/mmol), cooled with an ice bath. The resulting mixture was then stirred at r.t. until full consumption of the starting material (5–18 h, checked by TLC). Volatiles were evaporated on a rotavap and the products were precipitated with acetonitrile or *i-*PrOH. In some cases, the crude products were purified by recrystallization from EtOH/ether, acetonitrile, or *i*-PrOH.

## 4. Conclusions

We successfully designed irreversible α-amino ketone inhibitors of KAT-II using pharmacophore modeling and molecular docking. A series of thiazole- and triazole-based amino ketones was synthesized within the SAR study. Their inhibitory activity was determined in vitro, ultimately leading to sub-micromolar inhibitors of KAT-II **34a,b**, **49a,b**, and **49d**. The most potent inhibitor **49a** showed considerable activity with IC_50_ = 0.097 µM, which is comparable to the most active KAT-II inhibitors that have been published so far. Currently, we are focusing on the in vivo evaluation of the prepared inhibitors together with pharmacokinetic experiments and possible further structure optimization. In addition, our inhibitors have great potential for use in behavioral studies on rodents.

## Figures and Tables

**Table 1:** Inhibitory activities of compounds **11** and **12**.

Entry	Compound	Inhibitory Activity [%*I* ± SEM] *c* = 1 µM	IC_50_ ± SEM [µM]
1	**11**	41.41 ± 4.15	1.62 ± 1.43
2	**12**	43.19 ± 2.30	3.21 ± 0.26

**Table 2:** Synthesis of triazole derivatives with variation in the aryl moiety.

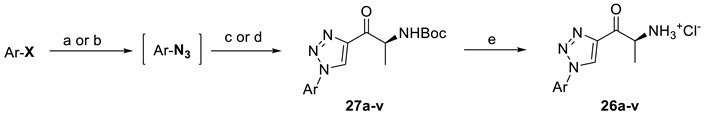 Reagents and conditions: (a) CuI, *N*,*N*’-dimethylethylenediamine (DMEDA), NaN_3_, sodium ascorbate, EtOH/H_2_O, reflux (X = Br, I); (b) (i) NaNO_2_, HCl, 0 °C, (ii) NaHCO_3_, NaN_3_, 0 °C–r.t. (X = NH_2_); (c) **15**, CuSO_4_.5H_2_O, sodium ascorbate, *t*-BuOH/H_2_O 1:1, r.t.; (d) **15**, CuTC, toluene, r.t.; and (e) AcCl, MeOH, r.t.									
**Entry**	**Ar**	**X**	**Boc** **Derivative**	**Amino** **Ketone**	**Entry**	**Ar**	**X**	**Boc** **Derivative**	**Amino** **Ketone**
1	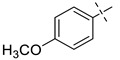	NH_2_	**27a**, 65%	**26a**, 82%	12	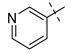	Br	**27l**, 67%	**26l**, 88% ^a^
2	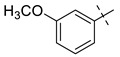	Br	**27b**, 67%	**26b**, 65%	13	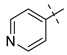	Br	**27m**, 66%	**26m**, 93% ^a^
3	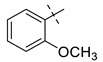	NH_2_	**27c**, 88%	**26c**, 79%	14	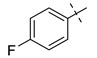	I	**27n**, 72%	**26n**, 95%
4	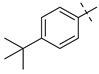	I	**27d**, 83%	**26d**, 89%	15	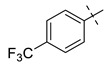	NH_2_	**27o**, 66%	**26o**, 71%
5	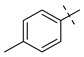	Br	**27e**, 76%	**26e**, 85%	16	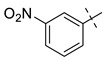	NH_2_	**27p**, 69%	**26p**, 70%
6	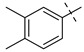	NH_2_	**27f**, 67%	**26f**, 72%	17	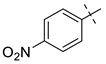	NH_2_	**27q**, 72%	**26q**, 75%
7	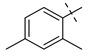	NH_2_	**27g**, 73%	**26g**, 71%	18	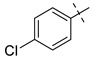	Br	**27r**, 44%	**26r**, 79%
8	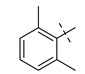	NH_2_	**27h**, 72%	**26h**, 70%	19	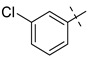	NH_2_	**27s**, 78%	**26s**, 67%
9	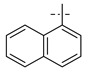	Br	**27i**, 58%	**26i**, 90%	20	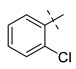	NH_2_	**27t**, 69%	**26t**, 72%
10	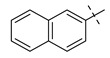	Br	**27j**, 86%	**26j**, 91%	21	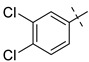	I	**27u**, 82%	**26u**, 76%
11	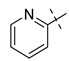	Br	**27k**, 47%	**26k**, 79%	22	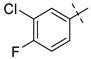	NH_2_	**27v**, 77%	**26v**, 79%

^a^ Product isolated as a dihydrochloride salt.

**Table 3:** Inhibitory activities of the prepared triazole and thiazole-based amino ketones in the SAR study.

Entry	Compound	Inhibitory Activity [%*I* ± SEM] *c* = 1 µM
	Triazole derivatives with the alkyl chain variation	
1	**21a**	10.32 ± 4.81
2	**21b**	14.52 ± 3.71
3	**21c**	16.50 ± 3.71
4	**21d**	13.46 ± 5.46
5	**21e**	21.82 ± 4.80
6	**21f**	7.44 ± 3.75
	Thiazole derivatives with the alkyl chain variation	
7	**25a**	31.24 ± 3.64
8	**25b**	7.22 ± 2.70
9	**25c**	31.32 ± 0.88
10	**25d**	37.22 ± 2.53
11	**25e**	44.18 ± 3.42
12	**25f**	6.12 ± 2.66
	Triazole derivatives with alkyl linker	
13	**28a**	14.54 ± 3.19
14	**28b**	11.84 ± 5.76
	Triazole derivatives with the aryl variation	
15	**26a**	38.50 ± 2.21
16	**26b**	18.20 ± 2.27
17	**26c**	23.41 ± 3.97
18	**26d**	0.98 ± 1.98
19	**26e**	18.56 ± 2.33
20	**26f**	9.10 ± 2.07
21	**26g**	41.60 ± 6.39
22	**26h**	25.38 ± 2.22
23	**26i**	4.32 ± 1.32
24	**26j**	27.81 ± 3.33
25	**26k**	20.73 ± 1.83
26	**26l**	23.63 ± 2.30
27	**26m**	33.48 ± 4.88
28	**26n**	41.77 ± 3.01
29	**26o**	9.70 ± 1.67
30	**26p**	41.43 ± 2.67
31	**26q**	41.73 ± 2.66
32	**26r**	23.86 ± 2.95
33	**26s**	20.63 ± 3.78
34	**26t**	35.52 ± 3.63
35	**26u**	19.33 ± 1.06
36	**26v**	23.62 ± 4.30

**Table 4:** Inhibitory activities of the optimized derivatives based on SAR evaluation.

Entry	Compound	Inhibitory Activity [%*I* ± SEM] *c* = 1 µM	IC_50_ ± SEM [µM]
1	**32a**	25.30 ± 1.10	N.D.
2	**32b**	21.25 ± 0.67	N.D.
3	**32c**	35.94 ± 2.87	N.D.
4	**34a**	52.49 ± 1.31	N.D.
5	**34b**	56.60 ± 3.68	0.987 ± 0.088
6	**34c**	36.16 ± 3.81	N.D.
7	**34d**	9.73 ± 1.41	N.D.
8	**49a**	61.54 ± 7.16	0.097 ± 0.014
9	**49b**	57.07 ± 0.31	N.D.
10	**49c**	47.65 ± 1.71	N.D.
11	**49d**	59.71 ± 0.97	0.304 ± 0.039

## Data Availability

Data is contained within the article or supplementary material.

## Supplementary Materials

The following are available online at https://www.mdpi.com/article/10.3390/ph14121291/s1.
