# Supplementary material for: Heterocyclic Cathinones as Inhibitors of Kynurenine Aminotransferase II—Design, Synthesis, and Evaluation
Source: Pharmaceuticals (Basel). 2021 Dec 10;14(12):1291. doi: 10.3390/ph14121291 (PMC8708382; doi:10.3390/ph14121291)
Supplement: Supplementary file 1 [file pharmaceuticals-14-01291-s001.zip › pharmaceuticals-1447267-supplementary/pharmaceuticals-1447267 SUPPL.pdf]

Supplementary material

for

Heterocyclic cathinones as inhibitors of kynurenine aminotransferase II – design, synthesis, and evaluation

Michal Maryška [1,2], Lucie Svobodová [1], Wim Dehaen [3], Martina Hrabinová [4,5], Michaela Rumlová [6], Ondřej Soukup [4,5] and Martin Kuchař [1,2,*]

1 Forensic Laboratory of Biologically Active Substances, Department of Chemistry of Natural Compounds, Faculty of Food and Biochemical Technology, University of Chemistry and Technology Prague, Technická 5, 16628 Prague, Czech Republic; maryskam@vscht.cz (M.M.); svobodbl@vscht.cz (L.S.)
2 National Institute of Mental Health, Topolová 748, 250 67 Klecany, Czech Republic
3 CZ-OPENSCREEN: National Infrastructure for Chemical Biology, Department of Informatics and
Chemistry, Faculty of Chemical Technology, University of Chemistry and Technology Prague, Technická 5, 16628 Prague, Czech Republic; dehaeni@vscht.cz
4 Biomedical Research Center, University Hospital Hradec Králové, Sokolská 581, 50005 Hradec Kralové, Czech Republic; martina.hrabinova@unob.cz (M.H.); ondrej.soukup@fnhk.cz (O.S.)
5 Department of Toxicology and Military Pharmacy, University of Defense, Třebešská 1575, 50005 Hradec Králové, Czech Republic
6 Department of Biotechnology, Faculty of Food and Biochemical Technology, University of Chemistry and Technology Prague, Technická 5, 16628 Prague, Czech Republic; rumlovai@vscht.cz
* Correspondence: kuchara@vscht.cz; Tel.: +420-220-444-431

Table of contents:

1. Synthesis and Characterization of the Prepared Compounds 2
1.1. General Synthetic Procedures 2
1.2. Weinreb amides 5
1.3. Alkynones 7
1.4. Thiazole-based Boc-amino ketones 8
1.5. Triazole-based Boc-amino ketones 16
1.6. Heterocyclic cathinones 30
2. References 44

1. Synthesis and Characterization of the Prepared Compounds

1.1. General Synthetic Procedures

General procedure 1 (GP1): Synthesis of Weinreb Amides from Boc-L-Amino Acids

To the solution of Boc-L-amino acid (1.0 eq.) in dry DCM (2.5 ml/mmol) was added CDI (1.1 eq.) portionwise and the mixture was stirred at r.t. for 1.25 hours. Solid *N*,*O*-dimethylhydroxylamine hydrochloride (1.11 eq.) was then added and the suspension was stirred overnight. DCM was evaporated and the residue was partitioned between EtOAc and 1M HCl. The organic phase was washed once more with 1M HCl, followed by saturated $NaHCO_3$ (2x), and brine, and was dried by $MgSO_4$. Evaporation of the solvent gave pure Weinreb amides.

General Procedure 2 (GP2): Synthesis of Alkynones from Weinreb Amides

The solution of Weinreb amide (1.0 eq.) in dry THF (4 ml/mmol) was cooled to -78 °C, followed by dropwise addition of ethynylmagnesium bromide (4.0 eq., 0.5M solution in THF). The resulting solution was stirred at -78 °C for 1 hour and then at r.t. overnight. The mixture was then poured into an ice-cold 1M aqueous $NaHSO_4$ (16 mL/mmol) and the biphasic mixture was stirred for 1 hour at 0 °C. Most of the THF was evaporated and the aqueous layer was extracted by Et_2O (3 × 15 mL/mmol). The combined organic phases were washed with 1M $NaHSO_4$, saturated $NaHCO_3$, and brine, and dried by $MgSO_4$. The crude product was purified by flash chromatography (SiO_2, 15–35 % EtOAc/hexane), yielding the corresponding alkynones.

General Procedure 3 (GP3): Acylation of Thiazoles and Benzo[*d*]thiazoles with Weinreb Amides

Step 1: The thiazole derivative (1.5 eq.) was dissolved in dry THF (2 mL/mmol) and the solution was cooled either to -10 °C or -78 °C. Then, a solution of *i*-PrMgCl.LiCl (1.5 eq. 1.3M solution in THF) or *n*-BuLi (1.5 eq, 2.5M solution in hexane) was added dropwise and the resulting mixture was stirred at -10 °C (if *i*-PrMgCl.LiCl was used) or at -78 °C (if *n*-BuLi was used) for 1–2 hours.

Step 2: The suspension/solution of Weinreb amide (1.0 eq.) in dry THF (2 mL/mmol) was cooled to -10 °C or -78 °C, and *i*-PrMgCl.LiCl (1.0 eq. 1.3M solution in THF) or *n*-BuLi (1.0 eq, 2.5M solution in hexane) was added dropwise. The resulting mixture was stirred for 10 minutes and then it was transferred into the solution of metallated thiazole from step 1. The reaction mixture was stirred for 15 minutes at -10 °C and then at r.t. overnight (if *i*-PrMgCl.LiCl was used) or was stirred 3–6h at -78 °C (if *n*-BuLi was used). The mixture was quenched with saturated NH_4Cl, diluted with water, and extracted with EtOAc (2 × 10 ml/mmol). The combined organic phases were washed with brine and dried by $MgSO_4$. Purification of crude products by flash chromatography (SiO_2, EtOAc/hexane or Et_2O/hexane) gave acylated thiazole or benzo[*d*]thiazole derivatives.

General Procedure 4 (GP4): Synthesis of Aryl Azides

Method A (**GP4A**): Concentrated HCl (0.2 mL/mmol) was added to the mixture of the aniline derivative (1.0 eq.) and water (2.2 mL/mmol), and the resulting solution was cooled with an ice

bath, followed by dropwise addition of $NaNO_2$ (1.0 eq., 5M aqueous solution). After 30 minutes, the mixture was neutralized with a cold saturated $NaHCO_3$ solution and then, still with cooling, NaN_3 (1.0 eq., 4M aqueous solution) was slowly added. The cooling bath was removed, and the mixture was stirred vigorously for 1 hour at r.t. The mixture was extracted with toluene (6 mL/mmol), and the organic phase was washed with saturated $NaHCO_3$, and brine, and dried by $MgSO_4$. The obtained toluene solutions of crude azides were used directly in the next step.

Method B (**GP4B**): Degassed EtOH/H_2O (7:3, 4 mL/mmol), followed by DMEDA (0.15 eq.) was added to the mixture of aryl bromide or aryl iodide (1.0 eq.), NaN_3 (2.0 eq.), CuI (0.1 eq.), and sodium ascorbate (0.05 eq.), and the resulting mixture was refluxed under argon for 1.5 h (aryl bromide) or 40 min (aryl iodide). After cooling, the mixture was diluted with water and extracted with hexane (3 × 10 mL/mmol). The combined organic phases were washed with brine and dried by $MgSO_4$. Careful removal of hexane on a rotavap (30 °C water bath) gave crude azides, which were used directly in the next step. Alternatively, the crude mixture was extracted by toluene and used directly in the next step.

General Procedure 5 (GP5): Synthesis of Triazole Derivatives by CuAAC

Method A (**GP5A**): *t*-BuOH/H_2O (1:1, 5 mL/mmol) was added to the mixture of crude azide (1.0 eq.) and alkynone (1.0 eq.), followed by sodium ascorbate (0.1 eq., 1M aqueous solution), and $CuSO_4.5H_2O$ (0.01 eq., 50 mg/ml aqueous solution). The mixture was stirred under argon at r.t. overnight. The suspension was diluted with ice-cold water and filtered. Solids were washed several times with water and vacuum dried. Trituration of the crude product with hexane gave pure triazoles. If no precipitation occurred after dilution with water, the mixture was extracted with EtOAc (3 × 15 mL/mmol) and the combined organic phases were washed with brine (2x) and dried by $MgSO_4$. The crude product was purified by flash chromatography (SiO_2, EtOAc/hexane).

Method B (**GP5B**): Alkynone (1.0 eq.) and crude azide (1.0 eq.) were dissolved in toluene (10 mL/mmol) (or the solution of crude azide in toluene from previous step was used), followed by the addition of CuTC (0.1 eq.) and the resulting mixture was stirred under argon overnight. The toluene was evaporated, and the residue was partitioned between DCM (20 mL/mmol) and saturated aqueous NH_4Cl. The aqueous phase was further extracted with DCM (2 × 20 mL/mmol) and the combined organic phases were washed with brine and dried by $MgSO_4$. The crude product was purified by flash chromatography (SiO_2, EtOAc/hexane), followed by precipitation from the hexane (if needed).

General Procedure 6 (GP6): Suzuki Coupling of 2-acyl-5-bromothiazoles

Degassed THF/H_2O (4:1, 10 mL/mmol) was added to the mixture of the bromothiazole derivative (1.0 eq), (4-fluorophenyl)boronic acid (1.1 eq), XPhos Pd G2 (0.03 eq.) and K_3PO_4 (2.0 eq.),and the resulting mixture was stirred under an argon atmosphere at 40 °C. To achieve full conversion of the starting bromothiazole, additional (4-fluorophenyl)boronic (0.5 eq.) was added a few times in 2–3 hour intervals (progress was monitored by TLC). Then, the reaction mixture was diluted with saturated aqueous NH_4Cl and extracted with EtOAc (3 × 20 mL/mmol). The combined organic phases were washed with brine and dried by $MgSO_4$. The crude products were purified by flash chromatography (SiO_2, 10–25 % EtOAc/hexane).

General procedure 7 (GP7): Synthesis of Heterocyclic Cathinones by Deprotection of Boc Protecting Group

Acetyl chloride (8.0 eq.) was added dropwise to a solution/suspension of Boc-derivative (1.0 eq.) in dry MeOH (4 mL/mmol), cooled with an ice bath. The resulting mixture was then stirred at r.t. until full consumption of the starting material (5–18 h, checked by TLC). Volatiles were evaporated on a rotavap and the products were precipitated with acetonitrile or *i*-PrOH. In some cases, the crude products were purified by recrystallization from EtOH/ether, acetonitrile, or *i*-PrOH.

1.2. Weinreb amides

tert-butyl (*S*)-{1-[methoxy(methyl)amino]-1-oxopropan-2-yl}carbamate (14)

Prepared according to **GP1** from Boc-L-alanine (**13)** (9.46 g, 50.0 mmol), CDI (8.92 g, 55.0 mmol) and *N*,*O*-dimethyl-hydroxylamine hydrochloride (5.42 g, 55.5 mmol) as a white solid (10.52 g, 91 %). Analytical data were in agreement with those previously reported.[1]

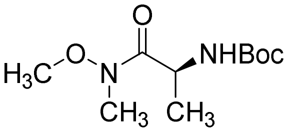

^{1}H NMR (400 MHz, $CDCl_3$) δ: 1.30 (d, 3H, J = 6.9 Hz), 1.43 (s, 9H), 3.20 (s, 3H), 3.76 (s, 3H), 4.60 – 4.74 (m br, 1H), 5.25 (d br, 1H, J = 6.8 Hz).

tert-butyl (*S*)-{1-[methoxy(methyl)amino]-3-methyl-1-oxobutan-2-yl}carbamate (23a)

Prepared according to **GP1** from Boc-L-valine (**19a**) (544 mg, 2.5 mmol), CDI (446 mg, 2.75 mmol) and *N*,*O*-dimethylhydroxylamine hydrochloride (271 mg, 2.78 mmol) as a colourless liquid (635 mg, 98 %). Analytical data were in agreement with those previously reported.[2]

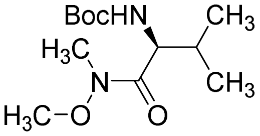

^{1}H NMR (400 MHz, $CDCl_3$) δ: 0.90 (d, 3H, J = 6.8 Hz), 0.95 (d, 3H, J = 6.9 Hz), 1.42 (s, 9H), 1.91–2.03 (m, 1H), 3.20 (s, 3H), 3.76 (s, 3H), 4.49 – 4.63 (m br, 1H), 5.13 (d br, 1H, J = 9.8 Hz).

tert-butyl (*S*)-{1-[methoxy(methyl)amino]-1-oxo-3-phenylpropan-2-yl}carbamate (23b)

Prepared according to **GP1** from Boc-L-phenylalanine (**19b**) (1.33 g, 5.0 mmol), CDI (892 mg, 5.5 mmol) and *N*,*O*-dimethylhydroxylamine hydrochloride (542 mg, 5.55 mmol) as a colourless syrup (1.45 g, 94 %). Analytical data were in agreement with those previously reported.[1]

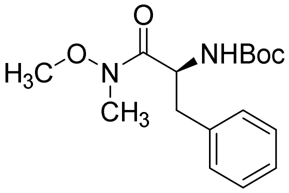

^{1}H NMR (400 MHz, $CDCl_3$) δ: 1.38 (s, 9H), 2.87 (dd br, 1H, J_1 = 13.4 Hz, J_2 = 7.6 Hz), 3.05 (dd br, 1H, J_1 = 13.8 Hz, J_2 = 6.3 Hz), 3.16 (s br, 3H), 3.65 (s, 3H), 4.88 – 4.99 (m br, 1H), 5.17 (d br, 1H, J = 8.0 Hz), 7.12 – 7.32 (m, 5H).

tert-butyl (_S_)-{1-[methoxy(methyl)amino]-1-oxo-4-phenylbutan-2-yl} carbamate (23c)

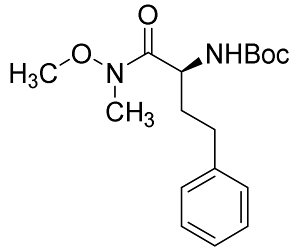

Prepared according to **GP1** from Boc-L-homophenylalanine (**19c**) (699 mg, 2.5 mmol), CDI (446 mg, 2.75 mmol) and *N*,*O*-dimethylhydroxylamine hydrochloride as a yellowish oil (805 mg, quant.). Analytical data were in agreement with those previously reported.[3]

^{1}H NMR (400 MHz, $CDCl_3$) δ: 1.45 (s, 9H), 1.75–1.89 (m, 1H), 1.96–2.07 (m, 1H), 2.59–2.80 (m, 2H), 3.15 (s, 3H), 3.61 (s, 3H), 4.59–4.76 (m br, 1H), 5.26 (d br, 1H, J = 9.4 Hz), 7.14–7.22 (m, 3H), 7.23–7.30 (m, 2H). ^{13}C NMR (101 MHz, $CDCl_3$) δ: 28.3, 31.6, 32.0, 34.5, 50.0, 61.4, 79.5, 125.9, 128.3, 128.5, 141.1, 155.5, 173.0.

tert-butyl (_S_)-{1-[methoxy(methyl)amino]-1-oxohexan-2-yl}carbamate (23d)

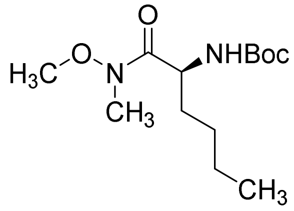

Prepared according to **GP1** from Boc-L-norleucine (**19d**) (579 mg, 2.5 mmol), CDI (446 mg, 2.75 mmol) and *N*,*O*-dimethyl-hydroxylamine hydrochloride as a colourless oil (675 mg, 99 %). Analytical data were in agreement with those previously reported.[3]

^{1}H NMR (400 MHz, $CDCl_3$) δ: 0.79–0.93 (m, 3H), 1.22–1.38 (m, 4H), 1.38–1.57 (m, 10 H), 1.61–1.74 (m, 1H), 3.18 (s, 3H), 3.75 (s, 3H), 4.57–4.71 (m br, 1H), 5.13 (d br, 1H, J = 9.3 Hz). ^{13}C NMR (101 MHz, $CDCl_3$) δ: 13.8, 22.3, 27.4, 28.3, 32.0, 32.6, 50.2, 61.5, 79.4, 155.5, 173.4.

tert-butyl (_S_)-{1-[methoxy(methyl)amino]-1-oxobutan-2-yl}carbamate (23e)

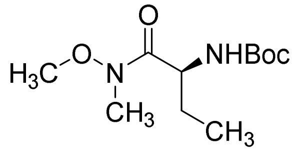

Prepared according to **GP1** from (*S*)-2-(*tert*-butoxycarbonyl-amino)butanoic acid (**19e**) (2.03 g, 10.0 mmol), CDI (1.79 g, 11.0 mmol) and *N*,*O*-dimethylhydroxylamine hydrochloride (1.08 g, 11.1 mmol) as a white solid (2.24 g, 91 %). Analytical data were in agreement with those previously reported.[3]

^{1}H NMR (400 MHz, $CDCl_3$) δ: 0.94 (t, 3H, J = 7.4 Hz), 1.43 (s, 9H), 1.51 – 1.63 (m, 1H), 1.75 (dtd, 1H, J_1 = 15.0 Hz, J_2 = 7.5 Hz, J_2 = 5.2 Hz), 3.20 (s, 3H), 3.77 (s, 3H), 4.53 – 4.72 (m br, 1H), 5.17 (d br, 1H, J = 8.6 Hz).

tert-butyl (_S_)-{1-cyclopropyl-2-[methoxy(methyl)amino]-2-oxoethyl}carbamate (23f)

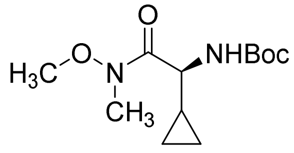

Prepared according to **GP1** from Boc-L-cyclopropylglycine (**19f**) (539 mg, 2.5 mmol), CDI (446 mg, 2.75 mmol) and *N*,*O*-dimethyl-hydroxylamine hydrochloride (271 mg, 2.78 mmol) as a white solid (645 mg, quant.). Analytical data were in agreement with those previously reported.[4]

^{1}H NMR (400 MHz, $CDCl_3$) δ: 0.31–0.38 (m, 1H), 0.39–0.52 (m, 3H), 1.03–1.16 (m, 1H), 1.41 (s, 9H), 3.21 (s, 3H), 3.75 (s, 3H), 4.31–4.50 (m br, 1H), 5.21 (d br, 1H, J = 8.5 Hz). ^{13}C NMR (101 MHz, $CDCl_3$) δ: 2.0, 2.1, 13.5, 28.3, 31.9, 52.0, 61.5, 79.5, 155.4, 172.3.

1.3. Alkynones

tert-butyl (*S*)-(3-oxopent-4-yn-2-yl)carbamate (15)

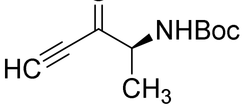

Prepared according to **GP2** from Weinreb amide **14** (3.48 g, 15.0 mmol) and ethynylmagnesium bromide (60.0 mmol, 120 mL, 0.5M solution in THF) as a pale yellow solid (2.78 g, 94 %). Analytical data were in agreement with those previously reported.[5]

^{1}H NMR (400 MHz, $CDCl_3$) δ: 1.40–1.45 (m, 12H), 3.36 (s, 1H), 4.34–4.47 (m br, 1H), 5.12 (s br, 1H). ^{13}C NMR (101 MHz, $CDCl_3$) δ: 17.2, 28.2, 56.9, 79.4, 80.1, 81.8, 155.0, 186.6.

tert-butyl (*S*)-(2-methyl-4-oxohex-5-yn-3-yl)carbamate (18a)

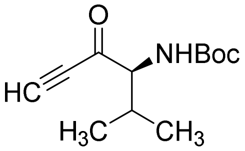

Prepared according to **GP2** from Weinreb amide **23a** (261 mg, 1.0 mmol), ethynylmagnesium bromide (4.0 mmol, 8 mL, 0.5M solution in THF) as a pale yellow syrup (194 mg, 86%). Analytical data were in agreement with those previously reported.[6]

^{1}H NMR (400 MHz, $CDCl_3$) δ: 0.83 (d, 3H, J = 6.9 Hz), 1.04 (d, 3H, J = 6.9 Hz), 1.44 (s, 9H), 2.28 (s br, minor rotamer) and 2.38–2.51 (m, major rotamer) (in total 1H), 3.37 (s, 1H), 4.04 (s br, minor rotamer) and 4.39 (dd, 1H, J_1 = 8.9 Hz, J_2 = 4.0 Hz, major rotamer) (in total 1H), 4.76 (s br, minor rotamer) and 5.04 (d br, J = 8.9 Hz, major rotamer) (in total 1H).

tert-butyl (*S*)-(3-oxo-1-phenylpent-4-yn-2-yl)carbamate (18b)

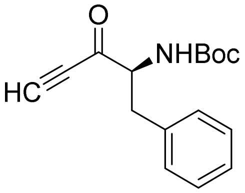

Prepared according to **GP2** from Weinreb amide **23b** (617 mg, 2.0 mmol) and ethynylmagnesium bromide (8.0 mmol, 16 mL, 0.5M solution in THF) as a pale yellow syrup (500 mg, 92%). Analytical data were in agreement with those previously reported.[7]

^{1}H NMR (400 MHz, $CDCl_3$) δ: 1.41 (s, 9H), 3.17 (dd, 1H, J_1 = 14.2 Hz, J_2 = 6.3 Hz), 3.27 (dd, 1H, J_1 = 14.1 Hz, J_2 = 5.7 Hz), 3.41 (s, 1H), 4.63–4.73 (m br, 1H), 4.99 (d, 1H, J = 7.9 Hz), 7.12–7.19 (m, 2H), 7.20–7.34 (m, 3H).

tert-butyl (*S*)-(4-oxo-1-phenylhex-5-yn-3-yl)carbamate (18c)

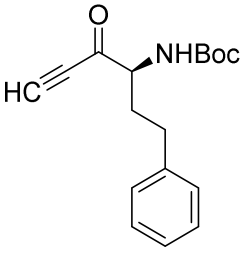

Prepared according to **GP2** from Weinreb amide **23c** (323 mg, 1.0 mmol) and ethynylmagnesium bromide (4.0 mmol, 8 mL, 0.5M solution in THF) as a pale yellow syrup (265 mg, 93%).

^{1}H NMR (400 MHz, $CDCl_3$) δ: 1.46 (s, 9H), 1.88–2.02 (m, 1H), 2.15–2.41 (m, 1H, major a minor rotamer), 2.69 (t, 2H, J = 8.0 Hz), 3.36 (s, 1H), 4.09 (s br, minor rotamer) and 4.41–4.51 (m br, major rotamer) (in total 1H), 4.90 (s br, minor rotamer) and 5.13 (d br, J = 8.1 Hz, major rotamer) (in total 1H), 7.15–7.24 (m, 3H), 7.26–7.34 (m, 2H). ^{13}C NMR (101 MHz, $CDCl_3$) δ: 28.3, 31.4, 32.9, 61.0, 79.6, 80.2, 81.9, 126.3, 128.4, 128.5, 140.5, 155.3, 186.2. HRMS (ESI): *m/z* $[M+H]^+$ calculated for $C_{17}H_{21}NO_3$: 288.15942, found: 288.15953.

***tert*-butyl (*S*)-(3-oxooct-1-yn-4-yl)carbamate (18d)**

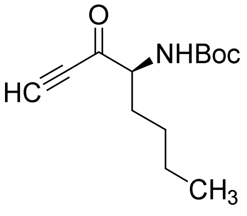

Prepared according to **GP2** from Weinreb amide **23d** (275 mg, 1.0 mmol) and ethynylmagnesium bromide (4.0 mmol, 8 mL, 0.5M solution in THF) as a pale yellow syrup (208 mg, 87%).

^{1}H NMR (400 MHz, $CDCl_3$) δ: 0.83–0.96 (m, 3H), 1.20–1.39 (m, 4H), 1.44 (s, 9H), 1.56–1.68 (m, 1H), 1.84 (s br, minor rotamer) and 1.87–2.02 (m, major rotamer) (in total 1H), 3.35 (s, 1H), 4.08 (s br, minor rotamer) and 4.32–4.45 (m br, major rotamer) (in total 1H), 4.77 (s br, minor rotamer) and 5.05 (d br, *J* = 8.0 Hz, major rotamer) (in total 1H). ^{13}C NMR (101 MHz, $CDCl_3$) δ: 13.8, 22.3, 27.1, 28.3, 30.9, 61.2, 79.7, 80.1, 81.6, 155.3, 186.7. HRMS (ESI): *m/z* $[M+H]^+$ calculated for $C_{13}H_{21}NO_3$: 240.15942, found: 240.15949.

***tert*-butyl (*S*)-(4-oxohex-5-yn-3-yl)carbamate (18e)**

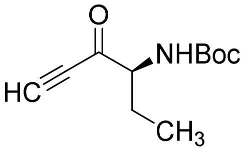

Prepared according to **GP2** from Weinreb amide **23e** (1.23 g, 5.0 mmol) and ethynylmagnesium bromide (20.0 mmol, 40 mL, 0.5M solution in THF) as a pale yellow syrup (1.00 g, 95%).

^{1}H NMR (400 MHz, $CDCl_3$) δ: 0.94 (t, 3H, *J* = 7.4 Hz), 1.44 (s, 9H), 1.68–1.82 (m, 1H), 1.58–2.11 (m, 1H, major a minor rotamer), 3.35 (s, 1H), 4.06 (s br, minor rotamer) and 4.33–4.43 (m br, major rotamer) (in total 1H), 4.79 (s br, minor rotamer) and 5.10 (d br, *J* = 7.5 Hz, major rotamer) (in total 1H). ^{13}C NMR (101 MHz, $CDCl_3$) δ: 9.3, 24.4, 28.3, 62.2, 79.7, 80.1, 81.6, 155.3, 186.5. HRMS (ESI): *m/z* $[M+H]^+$ calculated for $C_{11}H_{17}NO_3$: 212.12812, found: 212.12813.

***tert*-butyl (*S*)-(1-cyclopropyl-2-oxobut-3-yn-1-yl)carbamate (18f)**

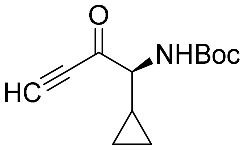

Prepared according to **GP2** from Weinreb amide **23f** (259 mg, 1.0 mmol) and ethynylmagnesium bromide (4.0 mmol, 8 mL, 0.5M solution in THF) as a pale yellow syrup (207 mg, 93%).

^{1}H NMR (400 MHz, $CDCl_3$) δ: 0.39–0.49 (m, 1H), 0.51–0.76 (m, 3H), 0.94–1.07 (m, 1H), 1.43 (s, 9H), 3.37 (s, 1H), 3.84 (t, 1H, *J* = 8.0 Hz), 4.87 (s br, minor rotamer) and 5.14 (s br, major rotamer) (in total 1H). ^{13}C NMR (101 MHz, $CDCl_3$) δ: 2.8, 3.9, 12.7, 28.2, 64.2, 80.2, 80.3, 81.7, 155.2, 185.5. HRMS (ESI): *m/z* $[M+H]^+$ calculated for $C_{12}H_{17}NO_3$: 224.12812, found: 224.12815.

1.4. Thiazole-based Boc-amino ketones

5-phenylthiazole (22)

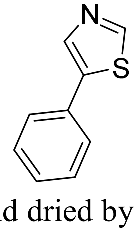

To the mixture of 5-bromothiazole (**48**) (2.46 g, 15.0 mmol), phenylboronic acid (2.75 g, 22.5 mmol) and K_2CO_3 (3.11 g, 22.5 mmol) were added degassed DME/H_2O (1:1, 60 mL) and $Pd(PPh_3)_4$ (867 mg, 0.75 mmol) and the resulting mixture was heated to reflux overnight. Then it was diluted with water and extracted with EtOAc (3 × 100 ml). The combined organic phases were washed with brine and dried by $MgSO_4$. Purification of the crude product by flash chromatography (SiO_2, 15–20 %

EtOAc/hexane) gave the product **22** (2.21 g, 91 %) as a white solid. Analytical data were inagreement with those previously reported.[8]

^{1}H NMR (400 MHz, $CDCl_3$) δ: 7.32–7.37 (m, 1H), 7.40–7.46 (m, 2H), 7.54–7.62 (m, 2H), 8.08 (s, 1H), 8.76 (s, 1H). ^{13}C NMR (101 MHz, $CDCl_3$) δ: 127.1, 128.6, 129.2, 131.2, 139.1, 139.5, 152.2.

5-(2-chloro-4-fluorophenyl)thiazole (47)

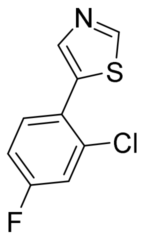

Prepared from 5-bromothiazole (**48**) (492 mg, 3.0 mmol) and (2-chloro-4-fluorophenyl)boronic acid (785 mg, 4.5 mmol) using the same procedure as for the preparation of compound **22**. Purification of the crude product by flash chromatography (SiO_2, 15–30% EtOAc/hexane) gave the product **47** (301 mg, 47%) as a white solid.

^{1}H NMR (400 MHz, $CDCl_3$) δ: 7.06 (ddd, 1H, J_1 = 8.7 Hz, J_2 = 7.7 Hz, J_3 = 2.6 Hz), 7.26 (dd, 1H, J_1 = 8.4 Hz, J_2 = 2.6 Hz), 7.48 (dd, 1H, J_1 = 8.7 Hz, J_2 = 5.9 Hz), 8.03 (s, 1H), 8.87 (s, 1H). ^{13}C NMR (101 MHz, $CDCl_3$) δ: 114.6 (d, J = 21.4 Hz), 117.8 (d, J = 24.9 Hz), 126.2 (d, J = 3.9 Hz), 132.7 (d, J = 8.8 Hz), 133.9 (d, J = 10.3 Hz), 134.0, 142.8, 153.6, 162.3 (d, J = 252.6 Hz). ^{19}F NMR (282 MHz, $CDCl_3$) δ: -111.2 (m, 1F).

***tert*-butyl (*S*)-[1-oxo-1-(5-phenylthiazol-2-yl)propan-2-yl]carbamate (16)**

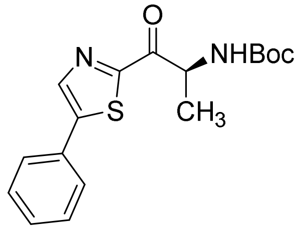

Prepared according to **GP3** from Weinreb amide **14** (233 mg, 1.0 mmol) and 2-bromo-5-phenylthiazole (361 mg, 1.5 mmol) using *i*-PrMgCl.LiCl. Purification by flash chromatography (SiO_2, 15–40% EtOAc/hexane) followed by the second flash chromatography (SiO_2, 50% Et_2O/hexane) gave compound **16** (195 mg, 59%) as a pale orange solid.

m.p.: 114–116 °C. ^{1}H NMR (400 MHz, $CDCl_3$) δ: 1.45 (s, 9H), 1.54 (d, 3H, J = 7.1 Hz), 5.38 (d br, 1H, J = 7.1 Hz), 5.41–5.52 (m br, 1H), 7.40–7.49 (m, 3H), 7.60 – 7.66 (m, 2H), 8.17 (s, 1H). ^{13}C NMR (101 MHz, $CDCl_3$) δ: 19.2, 28.3, 52.0, 79.8, 127.3, 129.4, 129.7, 130.3, 140.6, 147.4, 155.0, 162.5, 192.8. HRMS (ESI): *m/z* $[M+H]^+$ calculated for $C_{17}H_{20}N_2O_3S$: 333.12674, found: 333.12678.

***tert*-butyl (*S*)-[3-methyl-1-oxo-1-(5-phenylthiazol-2-yl)butan-2-yl]carbamate (24a)**

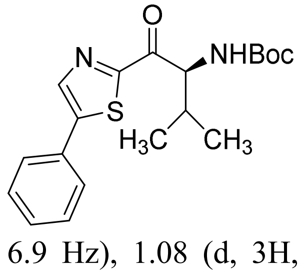

Prepared according to **GP3** from Weinreb amide **23a** (521 mg, 2.0 mmol) and 5-phenylthiazole (484 mg, 3.0 mmol) using *n*-BuLi. Purification by flash chromatography (SiO_2, 50% Et_2O/hexane) followed by precipitation from hexane gave compound **24a** (491 mg, 68 %) as a pale yellow solid.

m.p.: 85–88 °C. ^{1}H NMR (400 MHz, $CDCl_3$) δ: 0.86 (d, 3H, J = 6.9 Hz), 1.08 (d, 3H, J = 6.8 Hz), 1.45 (s, 9H), 2.34–2.53 (m, 1H), 5.35–5.40 (m br, 2H), 7.37–7.49 (m, 3H), 7.59–7.66 (m, 2H), 8.17 (s, 1H). ^{13}C NMR (101 MHz, $CDCl_3$) δ: 17.1, 20.1, 28.5, 31.6, 60.9, 79.8, 127.4, 129.5, 129.8, 130.5, 140.7, 147.5, 155.9, 163.6, 192.8. HRMS (ESI): *m/z* $[M+H]^+$ calculated for $C_{19}H_{24}N_2O_3S$: 361.15804, found: 361.15876.

***tert*-butyl (*S*)-[1-oxo-3-phenyl-1-(5-phenylthiazol-2-yl)propan-2-yl]carbamate (24b)**

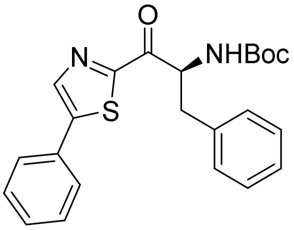

Prepared according to **GP3** from Weinreb amide **23b** (700 mg, 2.3 mmol) and 5-phenylthiazole (556 mg, 3.45 mmol) using *n*-BuLi. Purification by flash chromatography (SiO_2, 50% Et_2O/hexane) followed by precipitation from hexane gave compound **24b** (654 mg, 71 %) as a white solid. Analytical data were in agreement with those previously reported.[9]

m.p.: 109–112 °C. ^{1}H NMR (400 MHz, $CDCl_3$) δ: 1.38 (s, 9H), 2.87–3.22 (m, 1H), 3.24–3.47 (m, 1H), 5.00–5.33 (m, 1H), 5.42–5.82 (m br, 1H), 7.10 (d, 2H, *J* = 7.3 Hz), 7.15–7.28 (m, 3H), 7.38–7.48 (m, 3H), 7.62 (d, 2H, *J* = 7.1 Hz), 8.18 (s, 1H). ^{13}C NMR (101 MHz, $CDCl_3$) δ: 28.4, 39.0, 57.3, 79.9, 127.0, 127.4, 128.6, 129.5, 129.6, 129.9, 130.5, 136.2, 140.8, 147.7, 155.2, 163.0, 191.6. HRMS (ESI): *m/z* $[M+H]^+$ calculated for $C_{23}H_{24}N_2O_3S$: 409.15804, found: 409.15898.

***tert*-butyl (*S*)-[1-oxo-4-phenyl-1-(5-phenylthiazol-2-yl)butan-2-yl]carbamate (24c)**

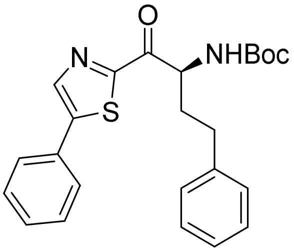

Prepared according to **GP3** from Weinreb amide **23c** (400 mg, 1.2 mmol) and 5-phenylthiazole (291 mg, 1.8 mmol) using *n*-BuLi. Purification by flash chromatography (SiO_2, 50% Et_2O/hexane) followed by precipitation from hexane gave compound **24c** (366 mg, 70%) as a white solid.

m.p.: 44–47 °C. ^{1}H NMR (400 MHz, $CDCl_3$) δ: 1.47 (s, 9H), 1.98–2.12 (m, 1H), 2.33–2.44 (m, 1H), 2.70–2.86 (m, 2H), 5.41–5.60 (m br, 2H), 7.13–7.20 (m, 3H), 7.21–7.32 (m, 2H), 7.41–7.49 (m, 3H), 7.60–7.65 (m, 2H), 8.17 (s, 1H). ^{13}C NMR (101 MHz, $CDCl_3$) δ: 28.5, 32.0, 35.1, 56.5, 80.0, 126.2, 127.4, 128.5, 128.5, 129.5, 129.9, 130.5, 140.8, 141.1, 147.7, 155.4, 162.9, 192.4. HRMS (ESI): *m/z* $[M+H]^+$ calculated for $C_{24}H_{26}N_2O_3S$: 423.17369, found: 423.17446.

***tert*-butyl (*S*)-[1-oxo-1-(5-phenylthiazol-2-yl)hexan-2-yl]carbamate (24d)**

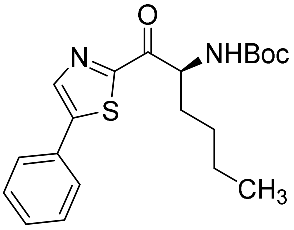

Prepared according to **GP3** from Weinreb amide **23d** (549 mg, 2.0 mmol) and 5-phenylthiazole (484 mg, 3.0 mmol) using *n*-BuLi. Purification by flash chromatography (SiO_2, 50% Et_2O/hexane) followed by precipitation from hexane gave compound **24d** (585 mg, 78%) as a white solid.

m.p.: 55–58 °C. ^{1}H NMR (400 MHz, $CDCl_3$) δ: 0.89 (t, 3H, *J* = 7.1 Hz), 1.26–1.49 (m, 13H), 1.63–1.77 (m, 1H), 1.94–2.08 (m, 1H), 5.28–5.50 (m br, 2H), 7.37–7.50 (m, 3H), 7.58–7.68 (m, 2H), 8.17 (s, 1H). ^{13}C NMR (101 MHz, $CDCl_3$) δ: 14.0, 22.6, 27.7, 28.5, 33.1, 56.3, 79.9, 127.4, 129.5, 129.8, 130.5, 140.8, 147.5, 155.6, 163.6, 192.9. HRMS (ESI): *m/z* $[M+H]^+$ calculated for $C_{20}H_{26}N_2O_3S$: 375.17369, found: 375.17435.

tert-butyl (S)-[1-oxo-1-(5-phenylthiazol-2-yl)butan-2-yl]carbamate (24e)

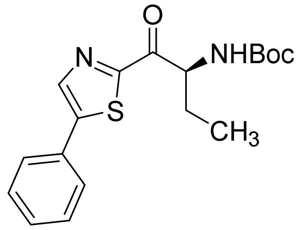

Prepared according to **GP3** from Weinreb amide **23e** (493 mg, 2.0 mmol) and 5-phenylthiazole (484 mg, 3.0 mmol) using *n*-BuLi. Purification by flash chromatography (SiO_2, 50% Et_2O/hexane) followed by precipitation from hexane gave compound **24e** (465 mg, 67 %) as a white.

m.p.: 87–89 °C. ^{1}H NMR (400 MHz, $CDCl_3$) δ: 0.99 (t, 3H, J = 7.4 Hz), 1.45 (s, 9H), 1.79 (dp, 1H, J_1 = 14.7 Hz, J_2 = 7.4 Hz), 2.02–2.15 (m, 1H), 5.34–5.44 (m br, 2H), 7.37–7.49 (m, 3H), 7.58–7.66 (m, 2H), 8.16 (s, 1H). ^{13}C NMR (101 MHz, $CDCl_3$) δ: 9.9, 26.6, 28.5, 57.3, 79.9, 127.4, 129.5, 129.9, 130.5, 140.7, 147.5, 155.6, 163.1, 192.7. HRMS (ESI): *m/z* $[M+H]^+$ calculated for $C_{18}H_{22}N_2O_3S$: 347.14239, found: 347.14247.

tert-butyl (S)-[2-cyclopropyl-1-oxo-1-(5-phenylthiazol-2-yl)ethan-2-yl]carbamate (24f)

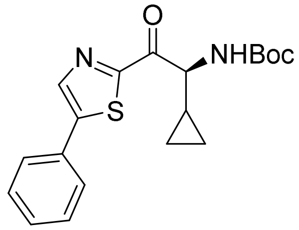

Prepared according to **GP3** from Weinreb amide **23f** (387 mg, 1.5 mmol) and 5-phenylthiazole (363 mg, 2.25 mmol) using *n*-BuLi. Purification by flash chromatography (SiO_2, 50% Et_2O/hexane) followed by precipitation from hexane gave compound **24f** (416 mg, 77%) as a white solid.

m.p.: 139–142 °C. ^{1}H NMR (400 MHz, $CDCl_3$) δ: 0.45–0.60 (m, 3H), 0.64–0.73 (m, 1H), 1.09–1.23 (m, 1H), 1.43 (s, 9H), 4.99 (t, 1H, J = 8.0 Hz), 5.42 (d, 1H, J = 7.8 Hz), 7.37–7.50 (m, 3H), 7.59–7.67 (m, 2H), 8.17 (s, 1H). ^{13}C NMR (101 MHz, $CDCl_3$) δ: 3.4, 3.6, 14.1, 28.4, 58.3, 80.0, 127.4, 129.5, 129.8, 130.6, 140.8, 147.5, 155.4, 163.8, 191.8. HRMS (ESI): *m/z* $[M+H]^+$ calculated for $C_{19}H_{22}N_2O_3S$: 359.14239, found: 359.14325.

tert-butyl (S)-[1-(benzo[d]thiazol-2-yl)-1-oxopropan-2-yl]carbamate (39a)

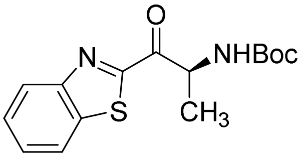

Prepared according to **GP3** from Weinreb amide **14** (140 mg, 0.6 mmol) and benzo[*d*]thiazole (**36**) (122 mg, 0.9 mmol) using *i*-PrMgCl.LiCl. Purification by flash chromatography (SiO_2, 10–30% EtOAc/hexane) followed by precipitation from hexane gave compound **39a** (147 mg, 80%) as a pale yellow solid.

m.p.: 99–100 °C. ^{1}H NMR (400 MHz, $CDCl_3$) δ: 1.45 (s, 9H), 1.58 (d, 3H, J = 7.3 Hz), 5.38 (s br, 1H), 5.52–5.68 (m br, 1H), 7.50–7.63 (m, 2H), 7.96–8.02 (m, 1H), 8.17–8.23 (m, 1H). ^{13}C NMR (101 MHz, $CDCl_3$) δ: 19.1, 28.3, 52.4, 79.9, 122.3, 125.7, 127.1, 127.9, 137.2, 153.5, 155.0, 163.6, 194.4. HRMS (ESI): *m/z* $[M+H]^+$ calculated for $C_{15}H_{18}N_2O_3S$: 307.11109, found: 307.11129.

tert-butyl (S)-[1-(6-fluorobenzo[d]thiazol-2-yl)-1-oxopropan-2-yl]carbamate (39b)

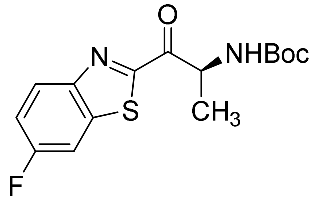

Prepared according to **GP3** from Weinreb amide **14** (140 mg, 0.6 mmol) and 6-fluorobenzo[*d*]thiazole (**37**) (138 mg, 0.9 mmol) using *i*-PrMgCl.LiCl. Purification by flash chromatography (SiO_2, 10–30% EtOAc/hexane) followed by precipitation from hexane gave compound **39b** (157 mg, 81%) as a pale yellow solid.

m.p.: 105–107 °C. ^{1}H NMR (400 MHz, $CDCl_3$) δ: 1.44 (s, 9H), 1.56 (d, 3H, J = 7.2 Hz), 5.35 (s br, 1H), 5.49–5.66 (m br, 1H) 7.33 (td, 1H, J_1 = 8.9 Hz, J_2 = 2.6 Hz), 7.65 (dd, 1H,

J_1 = 8.0 Hz, J_2 = 2.6 Hz), 8.15 (dd, 1H, J_1 = 9.1 Hz, J_2 = 4.8 Hz). ^{13}C NMR (101 MHz, $CDCl_3$) δ: 19.0, 28.3, 52.3, 80.0, 108.3 (d, J = 26.6 Hz), 116.5 (d, J = 25.3 Hz), 127.1 (d, J = 9.8 Hz), 138.5 (d, J = 11.6 Hz), 150.2 (d, J = 1.5 Hz), 155.0, 162.1 (d, J = 250.7 Hz), 163.5, 194.1. ^{19}F NMR (282 MHz, $CDCl_3$) δ: -111.2 (m, 1F). HRMS (ESI): m/z $[M+H]^+$ calculated for $C_{15}H_{17}FN_2O_3S$: 325.10167, found: 325.10185.

***tert*-butyl (*S*)-[1-(benzo[*d*]thiazol-2-yl)-1-oxobutan-2-yl]carbamate (39c)**

Prepared according to **GP3** from Weinreb amide **23e** (148 mg, 0.6 mmol) and benzo[*d*]thiazole (**36**) (122 mg, 0.9 mmol) using *i*-PrMgCl.LiCl. Purification by flash chromatography (SiO_2, 10–30 % EtOAc/hexane) followed by precipitation from hexane gave compound **39c** (160 mg, 83 %) as a pale yellow solid.

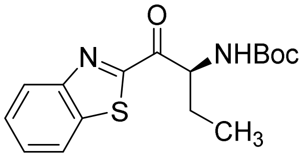

m.p.: 100 – 101 °C. ^{1}H NMR (400 MHz, $CDCl_3$) δ: 1.00 (t, 3H, J = 7.5 Hz), 1.45 (s, 9H), 1.85 (dt, 1H, J_1 = 14.0 Hz, J_2 = 7.4 Hz), 2.07–2.22 (m, 1H), 5.38 (d br, 1H, J = 8.5 Hz), 5.48–5.64 (m br, 1H), 7.50–7.63 (m, 2H), 7.95–8.02 (m, 1H), 8.17–8.24 (m, 1H). ^{13}C NMR (101 MHz, $CDCl_3$) δ: 9.7, 26.3, 28.3, 57.5, 79.8, 122.3, 125.8, 127.0, 127.9, 137.2, 153.6, 155.4, 163.9, 194.2. HRMS (ESI): m/z $[M+H]^+$ calculated for $C_{16}H_{20}N_2O_3S$: 321.12674, found: 321.12691.

***tert*-butyl (*S*)-[1-(6-fluorobenzo[*d*]thiazol-2-yl)-1-oxobutan-2-yl]carbamate (39d)**

Prepared according to **GP3** from Weinreb amide **23e** (148 mg, 0.6 mmol) and 6-fluorobenzo[*d*]thiazole (**37**) (138 mg, 0.9 mmol) using *n*-BuLi. Purification by flash chromatography (SiO_2, 10–30% EtOAc/hexane) followed by recrystallization from cyclohexane gave compound **39d** (100 mg, 49%) as a white solid.

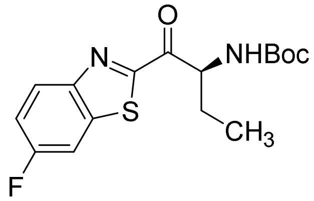

m.p.: 110–113 °C. ^{1}H NMR (400 MHz, $CDCl_3$) δ: 1.00 (t, 3H, J = 7.5 Hz), 1.44 (s, 9H), 1.82 (dp, 1H, J_1 = 14.0 Hz, J_2 = 7.4 Hz), 2.13 (dp, 1H, J_1 = 13.8 Hz, J_2 = 7.1 Hz), 5.31–5.38 (m, 1H), 5.48–5.58 (m br, 1H), 7.32 (dt, 1H, J_1 = 9.1 Hz, J_2 = 5.5 Hz), 7.61–7.68 (m, 1H), 8.16 (dd, 1H, J_1 = 9.0 Hz, J_2 = 4.8 Hz). ^{13}C NMR (101 MHz, $CDCl_3$) δ: 9.9, 26.3, 28.4, 57.6, 80.0, 108.4 (d, J = 26.7 Hz), 116.6 (d, J = 25.4 Hz), 127.3 (d, J = 9.9 Hz), 138.6 (d, J = 11.6 Hz), 150.4, 155.6, 162.2 (d, J = 250.9 Hz), 164.0, 194.0. ^{19}F NMR (282 MHz, $CDCl_3$) δ: -111.2 (m, 1F). HRMS (ESI): m/z $[M+Na]^+$ calculated for $C_{16}H_{19}FN_2O_3S$: 361.09926, found: 361.09943.

***tert*-butyl (*S*)-[1-oxo-1-(thiazol-2-yl)propan-2-yl]carbamate (42a)**

Prepared according to **GP3** from Weinreb amide **14** (1.16 g, 5.0 mmol) and thiazole (645 mg, 7.5 mmol) using *i*-PrMgCl.LiCl. Purification by flash chromatography (SiO_2, 20–30 % EtOAc/hexane) gave compound **42a** (1.20 g, 94%) as a white solid. Analytical data were in agreement with those previously reported.[10]

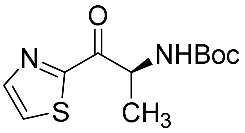

^{1}H NMR (400 MHz, $CDCl_3$) δ: 1.43 (s, 9H), 1.51 (d, 3H, J = 7.0 Hz), 5.29–5.40 (m, 1H), 5.40–5.54 (m br, 1H), 7.71 (d, 1H, J = 3.0 Hz), 8.04 (d, 1H, J = 3.0 Hz).

***tert*-butyl (*S*)-[1-oxo-1-(thiazol-2-yl)butan-2-yl]carbamate (42b)**

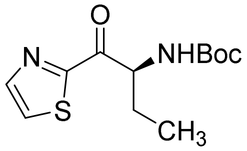

Prepared according to **GP3** from Weinreb amide **23e** (739 mg, 3.0 mmol) and thiazole (387 mg, 4.5 mmol) using *i*-PrMgCl.LiCl. Purification by flash chromatography (SiO_2, 20–30% EtOAc/hexane) gave compound **42b** (748 mg, 92%) as a pale yellow solid.

m.p.: 58–61 °C. ^{1}H NMR (400 MHz, $CDCl_3$) δ: 0.97 (t, 3H, J = 7.5 Hz), 1.44 (s, 9H), 1.69–1.84 (m, 1H), 2.01–2.16 (m, 1H) 5.34–5.43 (m br, 1H), 7.70 (d, 1H, J = 3.0 Hz), 8.04 (d, 1H, J = 3.0 Hz). ^{13}C NMR (101 MHz, $CDCl_3$) δ: 9.9, 26.4, 28.4, 57.7, 79.9, 126.7, 145.2, 155.5, 164.9, 192.7. HRMS (ESI): *m/z* $[M+Na]^+$ calculated for $C_{12}H_{18}N_2O_3S$: 293.09303, found: 293.09336.

***tert*-butyl (*S*)-[1-hydroxy-1-(thiazol-2-yl)propan-2-yl]carbamate (43a)**

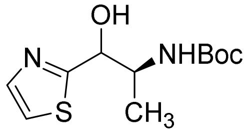

Thiazole derivative **42a** (1.03 g, 4.0 mmol) was dissolved in THF/MeOH (1:1, 5 mL) and the solution was cooled to 0 °C. $NaBH_4$ (230 mg, 6.0 mmol) was then added portionwise and the resulting solution was stirred at r.t. for 1 hour. The mixture was diluted with 5% aqueous NH_4Cl and extracted with EtOAc (3 × 50 ml). The combined organic phases were washed with brine and dried by $MgSO_4$. Evaporation of solvents gave the alcohol **43a** (1.00 g, 97 %, mixture of diastereomers) as pale yellow waxy solid, which was used without any purification directly in the next step.

Major diastereomer: ^{1}H NMR (400 MHz, $CDCl_3$) δ: 1.17 (d, 3H, J = 7.0 Hz), 1.43 (s, 9H), 4.12–4.30 (m, 1H), 4.77–4.88 (m, 1H), 5.00–5.05 (s br, 1H), 5.05–5.09 (m, 1H), 7.31 (d, 1H, J = 3.3 Hz), 7.73 (d, 1H, J = 3.3 Hz). ^{13}C NMR (101 MHz, $CDCl_3$) δ: 15.7, 28.4, 52.3, 75.6, 80.4, 119.4, 142.4, 157.1, 172.6. Minor diastereomer: ^{1}H NMR (400 MHz, $CDCl_3$) δ: 1.22 (d, 3H, J = 6.8 Hz), 1.38 (s, 9H), 3.94–4.07 (m, 1H), 4.66–4.77 (m, 1H), 4.98 (t, 1H, J = 4.6 Hz), 5.10–5.20 (m, 1H), 7.30 (d, 1H, J = 3.3 Hz), 7.72 (d, 1H, J = 3.3 Hz). ^{13}C NMR (101 MHz, $CDCl_3$) δ: 16.9, 28.4, 51.9, 75.0, 80.0, 119.3, 142.3, 173.4. HRMS (ESI): *m/z* $[M+Na]^+$ calculated for $C_{11}H_{18}N_2O_3S$: 281.09303, found: 281.09334.

***tert*-butyl (*S*)-[1-hydroxy-1-(thiazol-2-yl)butan-2-yl]carbamate (43b)**

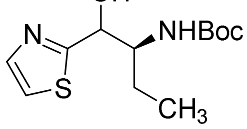

Prepared from the thiazole derivate **42b** (705 mg, 2.6 mmol) using the same procedure as for the preparation of compound **43a**, yielding the product **43b** (708 mg, quant., mixture of diastereomers) as pale yellow waxy solid, which was used without any purification directly in the next step.

Major diastereomer: ^{1}H NMR (400 MHz, $CDCl_3$) δ: 0.92–1.01 (m, 3H), 1.43 (s, 9H), 1.49–1.66 (m, 2H), 3.88–3.99 (m, 1H), 4.76 (d, 1H, J = 8.1 Hz), 5.07–5.11 (m, 1H), 5.11–5.20 (m, 1H), 7.30 (d, 1H, J = 3.2 Hz), 7.73 (d, 1H, J = 3.2 Hz). ^{13}C NMR (101 MHz, $CDCl_3$) δ: 11.0, 23.3, 28.4, 58.5, 75.2, 80.4, 119.3, 142.5, 157.8, 172.9. Minor diastereomer: ^{1}H NMR (400 MHz, $CDCl_3$) δ: 0.91–1.02 (m, 3H), 1.36 (s, 9H), 1.49–1.69 (m, 1H), 1.69–1.82 (m, 1H), 3.73–3.83 (m, 1H), 4.95 (d, 1H, J = 5.6 Hz), 5.00–5.06 (m, 1H), 5.06–5.11 (m, 1H), 7.29 (d, 1H, J = 3.2 Hz), 7.71 (d, 1H, J = 3.2 Hz). ^{13}C NMR (101 MHz, $CDCl_3$) δ: 10.9, 24.1, 28.4, 57.9, 73.9, 79.9, 119.3, 142.2, 156.9, 173.9. HRMS (ESI): *m/z* $[M+Na]^+$ calculated for $C_{12}H_{20}N_2O_3S$: 295.10868, found: 295.10907.

***tert*-butyl (*S*)-[1-(5-bromothiazol-2-yl)-1-hydroxypropan-2-yl]carbamate (44a)**

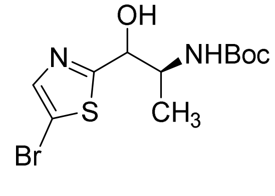

The solution of alcohol **43a** (1.00 g, 3.88 mmol) in DMF /20 ml) was cooled to 0 °C, followed by the addition of NBS (1.03 g, 5.8 mmol). The resulting solution was stirred at 50 °C overnight. The mixture was diluted with EtOAc (120 mL), washed with 10% aqueous $Na_2S_2O_3$ and brine (4x) and the organic phase was dried by $MgSO_4$. Purification of the crude product by flash chromatography (SiO_2, 20–35 % EtOAc/hexane) gave the two diastereomers of product **44a** (736 mg (pale yellow solid) + 398 mg (pale yellow waxy solid), 79% combined yield).

Major diastereomer: m.p.: 106–109 °C. ^1H NMR (400 MHz, $CDCl_3$) δ: 1.22 (d, 3H, J = 7.1 Hz), 1.44 (s, 9H), 4.06 – 4.22 (m, 1H), 4.72 (d, 1H, J = 6.9 Hz), 4.96 (dd, 1H, J_1 = 5.5 Hz, J_2 = 2.6 Hz), 5.29 (d, 1H, J = 5.5 Hz), 7.61 (s, 1H). ^{13}C NMR (101 MHz, $CDCl_3$) δ: 15.9, 28.4, 52.2, 76.3, 80.9, 109.3, 143.8, 157.5, 174.6. Minor diastereomer: ^1H NMR (400 MHz, $CDCl_3$) δ: 1.24 (d, 3H, J = 6.9 Hz), 1.40 (s, 9H), 3.92–4.05 (m, 1H), 4.88 (dd, 1H, J_1 = 5.3 Hz, J_2 = 4.1 Hz), 4.92 – 5.00 (m, 1H), 5.07–5.18 (m, 1H), 7.60 (s, 1H). ^{13}C NMR (101 MHz, $CDCl_3$) δ: 16.6, 28.4, 51.8, 75.4, 80.4, 109.3, 143.6, 156.7, 175.3. HRMS (ESI): *m/z* $[M+Na]^+$ calculated for $C_{11}H_{17}BrN_2O_3S$: 361.00150, found: 361.00155.

***tert*-butyl (*S*)-[1-(5-bromothiazol-2-yl)-1-hydroxybutan-2-yl]carbamate (44b)**

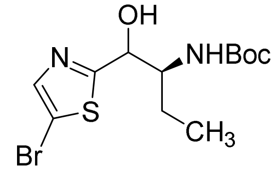

Prepared from the alcohol **43b** (690 mg, 2.5 mmol) using the same procedure as for the preparation of compound **44a**. Purification of the crude product by flash chromatography (SiO_2, 20–35 % EtOAc/hexane) gave the two diastereomers of product **44b** (357 mg (pale yellow solid) + 293 mg (pale yellow waxy solid), 74% combined yield).

Major diastereomer: m.p.: 104–108 °C. ^1H NMR (400 MHz, $CDCl_3$) δ: 0.99 (t, 3H, J = 7.4 Hz), 1.44 (s, 9H), 1.50–1.62 (m, 1H), 1.62–1.74 (m, 1H), 3.85–3.96 (m, 1H), 4.62 (d, 1H, J = 7.5 Hz), 4.99 (dd, 1H, J_1 = 5.6 Hz, J_2 = 2.5 Hz), 5.33 (d, 1H, J = 5.5 Hz), 7.60 (s, 1H). ^{13}C NMR (101 MHz, $CDCl_3$) δ: 11.1, 23.3, 28.4, 58.4, 75.7, 80.9, 109.3, 143.8, 158.1, 174.9. Minor diastereomer: ^1H NMR (400 MHz, $CDCl_3$) δ: 0.98 (t, 3H, J = 7.4 Hz), 1.39 (s, 9H), 1.53–1.69 (m, 1H), 1.70–1.83 (m, 1H), 3.67–3.78 (m, 1H), 4.95 (dd, 1H, J_1 = 5.8 Hz, J_2 = 3.5 Hz), 4.99–5.07 (m, 2H), 7.59 (s, 1H). ^{13}C NMR (101 MHz, $CDCl_3$) δ: 11.0, 23.8, 28.4, 57.9, 74.4, 80.4, 109.2, 143.6, 157.1, 175.8. HRMS (ESI): *m/z* $[M+Na]^+$ calculated for $C_{12}H_{19}BrN_2O_3S$: 375.01715, found: 375.01753.

***tert*-butyl (*S*)-[1-(5-bromothiazol-2-yl)-1-oxopropan-2-yl]carbamate (40a)**

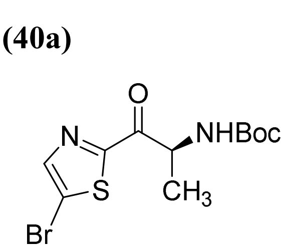

The solution of bromothiazole derivative **44a** 514 mg, 1.5 mmol) in DCM (7 mL) was cooled to 0 °C, followed by the addition of Dess-Martin periodinane (808 mg, 1.9 mmol). The resulting mixture was stirred at r.t. overnight and then the reaction was quenched by the addition of $Na_2S_2O_3$/$NaHCO_3$ solution. The biphasic mixture was stirred vigorously until it became clear and the aqueous layer was extracted with DCM (3 × 10 mL). The combined organic phases were washed with brine and dried by MgSO4. Evaporation of solvents gave the pure product **40a** (493 mg, 98%) as a white solid.

m.p.: 135–140 °C. ^{1}H NMR (400 MHz, $CDCl_3$) δ: 1.43 (s, 9H), 1.49 (d, 3H, J = 7.1 Hz), 5.18–5.32 (m br, 1H), 5.32–5.48 (m br, 1H), 7.91 (s, 1H). ^{13}C NMR (101 MHz, $CDCl_3$) δ: 19.1, 28.5, 52.0, 80.1, 118.9, 146.6, 155.1, 165.6, 192.3. HRMS (ESI): *m/z* $[M+Na]^+$ calculated for $C_{11}H_{15}BrN_2O_3S$: 358.98585, found: 358.98600.

***tert*-butyl (*S*)-[1-(5-bromothiazol-2-yl)-1-oxobutan-2-yl]carbamate (40b)**

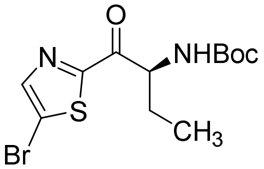

Prepared from the bromothiazole derivate **44b** (490 mg, 1.4 mmol) using the same procedure as for the preparation of compound **40a**, yielding the product **40b** (471 mg, 97 %) as a pale yellow solid.

m.p.: 81–84 °C. ^{1}H NMR (400 MHz, $CDCl_3$) δ: 0.96 (t, 3H, J = 7.4 Hz), 1.43 (s, 9H), 1.73 (dd, 1H, J_1 = 14.7 Hz, J_2 = 7.4 Hz), 1.98–2.11 (m, 1H), 5.02–5.43 (m br, 2H), 7.91 (s, 1H). ^{13}C NMR (101 MHz, $CDCl_3$) δ: 9.9, 26.3, 28.4, 57.1, 80.0, 118.8, 146.6, 155.5, 166.0, 192.0. HRMS (ESI): *m/z* $[M+Na]^+$ calculated for $C_{12}H_{17}BrN_2O_3S$: 373.00150, found: 373.00174.

***tert*-butyl (*S*)-[1-(5-(4-fluorophenyl)thiazol-2-yl)-1-oxopropan-2-yl]carbamate (45a)**

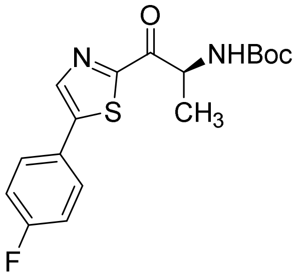

Prepared according to **GP6** from bromothiazole **40a** (235 mg, 0.7 mmol) and (4-fluorophenyl)boronic acid (588 mg, 4.2 mmol in total) as a white solid (189 mg, 77%).

m.p.: 176–180 °C. ^{1}H NMR (400 MHz, $CDCl_3$) δ: 1.45 (s, 9H), 1.53 (t, 3H, J = 7.1 Hz), 5.31–5.40 (m, 1H), 5.40–5.52 (m, 1H), 7.09–7.20 (m, 2H), 7.56–7.66 (m, 2H), 8.11 (s, 1H). ^{13}C NMR (101 MHz, $CDCl_3$) δ: 19.4, 28.5, 52.2, 80.0, 116.7 (d, J = 22.1 Hz), 126.7 (d, J = 3.4 Hz), 129.3 (d, J = 8.4 Hz), 140.7, 146.4, 155.2, 162.6, 163.5 (d, J = 251.5 Hz), 193.0. ^{19}F NMR (282 MHz, $CDCl_3$) δ: -111.0 (m, 1F). HRMS (ESI): *m/z* $[M+Na]^+$ calculated for $C_{17}H_{19}FN_2O_3S$: 373.09926, found: 373.09976.

***tert*-butyl (*S*)-[1-(5-(4-fluorophenyl)thiazol-2-yl)-1-oxobutan-2-yl]carbamate (45b)**

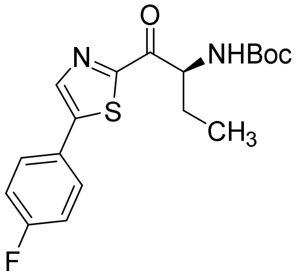

Prepared according to **GP6** from bromothiazole **40b** (210 mg, 0.6 mmol) and (4-fluorophenyl)boronic acid (504 mg, 3.6 mmol in total) as a pale yellow solid (186 mg, 88 %).

m.p.: 129–132 °C. ^{1}H NMR (400 MHz, $CDCl_3$) δ: 0.99 (t, 3H, J = 7.4 Hz), 1.45 (s, 9H), 1.71–1.87 (m, 1H), 2.02–2.15 (m, 1H), 5.32–5.46 (m, 2H), 7.10–7.21 (m, 2H), 7.56–7.66 (m, 2H), 8.10 (s, 1H). ^{13}C NMR (101 MHz, $CDCl_3$) δ: 9.9, 26.5, 28.5, 57.3, 79.9, 116.7 (d, J = 22.1 Hz), 126.7 (d, J = 3.3 Hz), 129.3 (d, J = 8.4 Hz), 140.7, 146.3, 155.6, 163.2, 163.7 (d, J = 250.7 Hz), 192.7. ^{19}F NMR (282 MHz, $CDCl_3$) δ: -111.0 (m, 1F). HRMS (ESI): *m/z* $[M+Na]^+$ calculated for $C_{18}H_{21}FN_2O_3S$: 387.11491, found: 387.11562.

tert-butyl (_S_)-[1-(5-(2-chloro-4-fluorophenyl)thiazol-2-yl)-1-oxopropan-2-yl]carbamate (46a)

Prepared according to **GP3** from Weinreb amide **14** (117 mg, 0.5 mmol) and 5-(2-chloro-4-fluorophenyl)thiazole (**47**) (161 mg, 0.75 mmol) using *n*-Buli for metalation and *i*-PrMgCl.LiCl for pre-deprotonation of Weinreb amide. Purification by flash chromatography (SiO_2, 20–35% EtOAc/hexane) followed by recrystallization from hexane/Et_2O gave compound **46a** (107 mg, 56%) as a white solid.

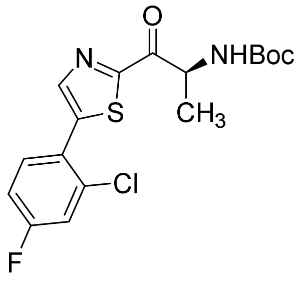

m.p.: 163–170 °C (decomp.). ^{1}H NMR (400 MHz, $CDCl_3$) δ: 1.45 (s, 9H), 1.55 (t, 3H, J = 7.1 Hz), 5.34 (d br, 1H, J = 7.0 Hz), 5.40–5.55 (m br, 1H), 7.10 (ddd, 1H, J_1 = 8.7 Hz, J_2 = 7.6 Hz, J_3 = 2.6 Hz), 7.29 (dd, 1H, J_1 = 8.3 Hz, J_2 = 2.6 Hz), 7.52 (dd, 1H, J_1 = 8.7 Hz, J_2 = 5.8 Hz), 8.13 (s, 1H). ^{13}C NMR (101 MHz, $CDCl_3$) δ: 19.1, 28.3, 52.1, 79.8, 114.9 (d, J = 21.4 Hz), 118.2 (d, J = 25.1 Hz), 125.5 (d, J = 3.8 Hz), 132.7 (d, J = 9.1 Hz), 134.0 (d, J = 10.5 Hz), 141.8, 144.3, 155.1, 162.8 (d, J = 254.2 Hz), 164.1, 193.1. ^{19}F NMR (282 MHz, $CDCl_3$) δ: -109.5 (m, 1F). HRMS (ESI): *m/z* $[M+H]^+$ calculated for $C_{17}H_{18}ClFN_2O_3S$: 385.07835, found: 385.07875.

tert-butyl (_S_)-[1-(5-(2-chloro-4-fluorophenyl)thiazol-2-yl)-1-oxobutan-2-yl]carbamate (46b)

Prepared according to **GP3** from Weinreb amide **23e** (124 mg, 0.5 mmol) and 5-(2-chloro-4-fluorophenyl)thiazole (**47**) (161 mg, 0.75 mmol) using *n*-Buli for metalation and *i*-PrMgCl.LiCl for pre-deprotonation of Weinreb amide. Purification by flash chromatography (SiO_2, 20–35% EtOAc/hexane) followed by recrystallization from hexane/Et_2O gave compound **46b** (120 mg, 60%) as a white solid.

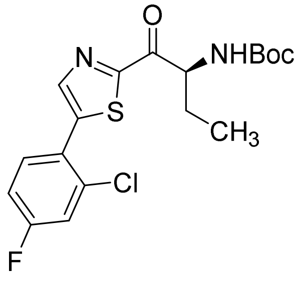

m.p.: 145 – 148 °C. ^{1}H NMR (400 MHz, $CDCl_3$) δ: 1.01 (t, 3H, J = 7.5 Hz), 1.45 (s, 9H), 1.80 (dp, J_1 = 14.8 Hz, J_2 = 7.4 Hz), 2.05 – 2.20 (m, 1H), 5.34 (d br, 1H, J = 8.7 Hz), 5.38 – 5.50 (m br, 1H), 7.10 (ddd, 1H, J_1 = 8.7 Hz, J_2 = 7.6 Hz, J_3 = 2.6 Hz), 7.29 (dd, 1H, J_1 = 8.3 Hz, J_2 = 2.6 Hz), 7.52 (dd, 1H, J_1 = 8.7 Hz, J_2 = 5.9 Hz), 8.13 (s, 1H). ^{13}C NMR (101 MHz, $CDCl_3$) δ: 9.8, 26.3, 28.3, 57.3, 79.8, 114.9 (d, J = 21.6 Hz), 118.2 (d, J = 24.9 Hz), 125.5 (d, J = 3.6 Hz), 132.7 (d, J = 9.0 Hz), 134.0 (d, J = 10.5 Hz), 141.7, 144.3, 155.4, 162.8 (d, J = 254.1 Hz), 164.4, 192.7. ^{19}F NMR (282 MHz, $CDCl_3$) δ: -109.5 (m, 1F). HRMS (ESI): *m/z* $[M+H]^+$ calculated for $C_{18}H_{20}ClFN_2O_3S$: 399.09400, found: 399.09446.

1.5. Triazole-based Boc-amino ketones

tert-butyl (_S_)-[1-oxo-1-(1-phenyl-1_H_-1,2,3-triazol-4-yl)propan-2-yl]carbamate (17)

Step 1: Azidobenzene was prepared according to **GP4A** from aniline (466 mg, 5.0 mmol), $NaNO_2$ (345 mg, 5.0 mmol) and NaN_3 (326 mg, 5.0 mmol) using different workup. Et_2O was used for extraction and organic layer was then carefully evaported. Crude azidobenzene (ca. 89 wt.% purity according to NMR) was used in the next step.

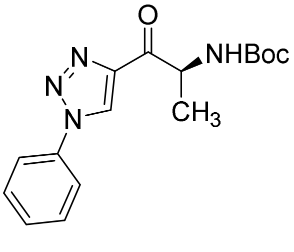

Step 2: Crude azidobenzene (239 mg, 2.0 mmol) and alkynone **15** (395 mg, 2.0 mmol) were reacted according to **GP5A**. Triazole **17** (530 mg, 84%) was obtained as pale yellow solid after filtration and precipitation from hexane.

m.p.: 101–103 °C. ^{1}H NMR (400 MHz, $CDCl_3$) δ: 1.45 (s, 9H), 1.57 (d, 3H, J = 6.5 Hz), 5.26–5.50 (m br, 2H), 7.48–7.54 (m, 1H), 7.54–7.61 (m, 2H), 7.71–7.79 (m, 2H), 8.56 (s, 1H). ^{13}C NMR (101 MHz, $CDCl_3$) δ: 18.9, 28.3, 53.1, 79.7, 120.8, 124.7, 129.7, 130.0, 136.2, 145.9, 155.1, 193.7. HRMS (ESI): *m/z* $[M+H]^+$ calculated for $C_{16}H_{20}N_4O_3$: 317.16082, found: 317.16070.

***tert*-butyl (*S*)-[3-methyl-1-oxo-1-(1-phenyl-1*H*-1,2,3-triazol-4-yl)butan-2-yl]carbamate (20a)**

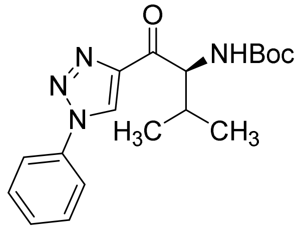

Step 1: Azidobenzene was prepared in the same way as for **17**.

Step 2: Crude azidobenzene (81 mg, 0.68 mmol) and alkynone **18a** (153 mg, 0.68 mmol) were reacted according to **GP5A**. After extraction with EtOAc and purification by flash chromatography (SiO_2, 10–25 % EtOAc/hexane), triazole **20a** (220 mg, 94%) was obtained as a colourless oil, which crystalized upon standing in a fridge.

m.p.: 100–101 °C. ^{1}H NMR (400 MHz, $CDCl_3$) δ: 0.85 (d, 3H, J = 6.9 Hz), 1.11 (d, 3H, J = 6.8 Hz), 1.45 (s, 9H), 2.43–2.62 (m, 1H), 5.30 (dd br, 1H, J_1 = 9.3 Hz, J_2 = 4.4 Hz), 5.40 (d br, 1H, J = 9.2 Hz), 7.47–7.54 (m, 1H), 7.54–7.61 (m, 2H), 7.72–7.79 (m, 2H), 8.54 (s, 1H). ^{13}C NMR (101 MHz, $CDCl_3$) δ: 16.7, 20.0, 28.3, 31.0, 62.0, 79.6, 120.7, 124.5, 129.6, 130.0, 136.2, 146.6, 155.8, 193.5. HRMS (ESI): *m/z* $[M+H]^+$ calculated for $C_{18}H_{24}N_4O_3$: 345.19212, found: 345.19193.

***tert*-butyl (*S*)-[1-oxo-3-phenyl-1-(1-phenyl-1*H*-1,2,3-triazol-4-yl)propan-2-yl]carbamate (20b)**

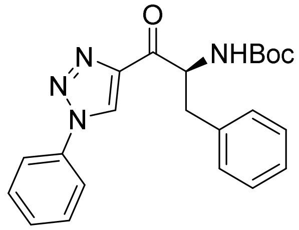

Step 1: Azidobenzene was prepared in the same way as for **17**.

Step 2: Crude azidobenzene (120 mg, 1.0 mmol) and alkynone **18b** (274 mg, 1.0 mmol) were reacted according to **GP5A**. Triazole **20b** (277 mg, 71%) was obtained as a white solid after filtration and precipitation from hexane.

m.p.: 131–133 °C. ^{1}H NMR (400 MHz, $CDCl_3$) δ: 1.31 (s, minor rotamer) and 1.41 (s, major rotamer) (in total 9H), 3.01 (s br, minor rotamer) and 3.20 (dd, J_1 = 14.1 Hz, J_2 = 7.0 Hz, major rotamer) (in total 1H), 3.39 (s br, minor rotamer) and 3.47 (dd, J_1 = 14.1 Hz, J_2 = 5.3 Hz, major rotamer) (in total 1H), 5.16 (s br, minor rotamer) and 5.33 (d br, J = 8.1 Hz, major rotamer) (in total 1H), 5.53–5.75 (m br, 1H), 7.07–7.34 (m, 5H), 7.44–7.65 (m, 3H), 7.77 (d, J = 7.7 Hz, 2H), 8.54 (s, 1H). ^{13}C NMR (101 MHz, $CDCl_3$) δ: 28.3, 38.3, 58.2, 79.8, 120.8, 124.6, 126.9, 128.4, 129.5, 129.7, 130.0, 136.2, 136.2, 146.2, 155.1, 192.3. HRMS (ESI): *m/z* $[M+H]^+$ calculated for $C_{22}H_{24}N_4O_3$: 393.19212, found: 393.19232.

tert-butyl (S)-[1-oxo-4-phenyl-1-(1-phenyl-1H-1,2,3-triazol-4-yl)butan-2-yl]carbamate (20c)

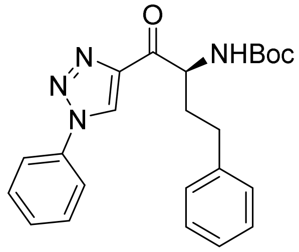

Step 1: Azidobenzene was prepared in the same way as for **17**.

Step 2: Crude azidobenzene (102 mg, 0.85 mmol) and alkynone **18c** (245 mg, 0.85 mmol) were reacted according to **GP5A**. Triazole **20c** (307 mg, 89%) was obtained as a white solid after filtration and precipitation from hexane.

m.p.: 98–100 °C. ^{1}H NMR (400 MHz, $CDCl_3$) δ: 1.46 (s, 9H), 2.02–2.16 (m, 1H), 2.36–2.51 (m br, 1H), 2.69–2.88 (m, 2H), 5.37–5.46 (m br, 1H), 5.50 (d br, 1H, J = 8.3 Hz), 7.12–7.20 (m, 3H), 7.21–7.26 (m, 2H), 7.48–7.54 (m, 1H), 7.57 (t, 2H, J = 7.7 Hz), 7.73 (d, 2H, J = 7.7 Hz), 8.44 (s, 1H). ^{13}C NMR (101 MHz, $CDCl_3$) δ: 28.3, 31.7, 34.6, 57.3, 79.8, 120.8, 124.5, 126.0, 128.4, 128.5, 129.7, 130.0, 136.2, 141.0, 146.0, 155.5, 193.0. HRMS (ESI): *m/z* $[M+H]^+$ calculated for $C_{23}H_{26}N_4O_3$: 407.20777, found: 407.20813.

tert-butyl (S)-[1-oxo-1-(1-phenyl-1H-1,2,3-triazol-4-yl)hexan-2-yl]carbamate (20d)

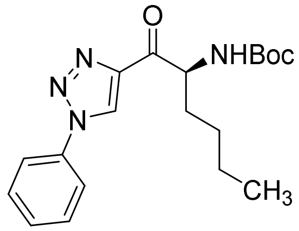

Step 1: Azidobenzene was prepared in the same way as for **17**.

Step 2: Crude azidobenzene (96 mg, 0.8 mmol) and alkynone **18d** (190 mg, 0.8 mmol) were reacted according to **GP5A**. Triazole **20d** (237 mg, 83%) was obtained as a white solid after filtration and precipitation from hexane.

m.p.: 95 – 97 °C. ^{1}H NMR (400 MHz, $CDCl_3$) δ: 0.89 (t, 3H, J = 7.1 Hz), 1.27–1.51 (m, 13H), 1.67–1.81 (m, 1H), 1.99–2.17 (m, 1H), 5.37 (s br, 2H), 7.48–7.54 (m, 1H), 7.54–7.61 (m, 2H), 7.72–7.79 (m, 2H), 8.55 (s, 1H). ^{13}C NMR (101 MHz, $CDCl_3$) δ: 13.9, 22.4, 27.5, 28.3, 32.5, 57.3, 79.7, 120.8, 124.5, 129.6, 130.0, 136.3, 146.2, 155.5, 193.7. HRMS (ESI): *m/z* $[M+H]^+$ calculated for $C_{19}H_{26}N_4O_3$: 359.20777, found: 359.20788.

tert-butyl (S)-[1-oxo-1-(1-phenyl-1H-1,2,3-triazol-4-yl)butan-2-yl]carbamate (20e)

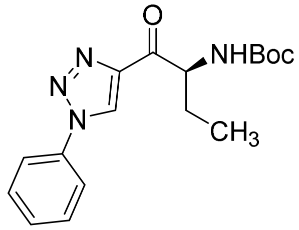

Step 1: Azidobenzene was prepared in the same way as for **17**.

Crude azidobenzene (102 mg, 0.85 mmol) and alkynone **18e** (180 mg, 0.85 mmol) were reacted according to **GP5A**. Triazole **20e** (232 mg, 83%) was obtained as a white solid after filtration and precipitation from hexane.

m.p.: 114–115 °C. ^{1}H NMR (400 MHz, $CDCl_3$) δ: 0.99 (t, 3H, J = 7.4 Hz), 1.45 (s, 9H), 1.84 (dp, 1H, J_1 = 14.6 Hz, J_2 = 7.3 Hz), 2.11–2.22 (m, 1H), 5.29–5.39 (m br, 1H), 5.42 (d br, 1H, J = 7.4 Hz), 7.47–7.54 (m, 1H), 7.54–7.62 (m, 2H), 7.72–7.79 (m, 2H), 8.55 (s, 1H). ^{13}C NMR (101 MHz, $CDCl_3$) δ: 9.6, 25.9, 28.3, 58.3, 79.7, 120.8, 124.5, 129.6, 130.0, 136.2, 146.2, 155.5, 193.4. HRMS (ESI): *m/z* $[M+H]^+$ calculated for $C_{17}H_{22}N_4O_3$: 331.17647, found: 331.17646.

tert-butyl (S)-[1-cyclopropyl-2-oxo-2-(1-phenyl-1H-1,2,3-triazol-4-yl)ethyl]carbamate (20f)

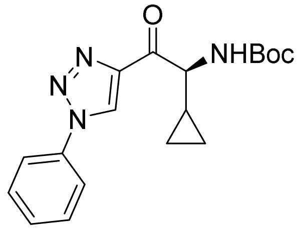

Step 1: Azidobenzene was prepared in the same way as for **17**.

Step 2: Crude azidobenzene (102 mg, 0.85 mmol) and alkynone **18f** (190 mg, 0.85 mmol) were reacted according to **GP5A**. Triazole **20f** (240 mg, 83%) was obtained as a white solid after filtration and precipitation from hexane.

m.p.: 102–105 °C. ^{1}H NMR (400 MHz, $CDCl_3$) δ: 0.46–0.63 (m, 3H), 0.69–0.81 (m, 1H), 1.13–1.24 (m, 1H), 1.43 (s, 9H), 4.82 (s br, minor rotamer) and 4.90 (t br, J = 7.8 Hz, major rotamer) (in total 1H), 5.16 (s br, minor rotamer) and 5.45 (d br, J = 7.3 Hz, major rotamer) (in total 1H), 7.47–7.54 (m, 1H), 7.57 (t, 2H, J = 7.7 Hz), 7.76 (d, 2H, J = 7.7 Hz), 8.58 (s, 1H). ^{13}C NMR (101 MHz, $CDCl_3$) δ: 3.1, 3.5, 13.6, 28.3, 59.4, 79.7, 120.8, 124.8, 129.6, 130.0, 136.3, 146.6, 155.3, 192.7. HRMS (ESI): *m/z* $[M+H]^+$ calculated for $C_{18}H_{22}N_4O_3$: 343.17647, found: 343.17646.

tert-butyl (S)-[1-(1-benzyl-1H-1,2,3-triazol-4-yl)-1-oxopropan-2-yl]carbamate (31a)

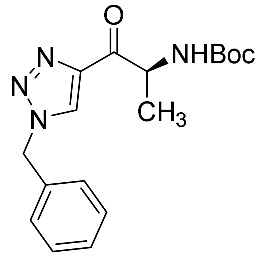

Step 1: To benzyl bromide (**29**) (86 mg, 0.5 mmol) and NaN_3 (49 mg, 0.75 mmol) was added acetone/water (4:1, 5 mL) and the mixture was stirred at r.t. for one day. Then more water was added and the mixture was extracted with DCM (3 × 3ml), combined organic phases were dried by $MgSO_4$ and evaporated at 30 °C. The obtained crude benzyl azide was used directly into next step.

Step 2: Crude benzyl azide and alkynone **15** (99 mg, 0.5 mmol) were reacted according to **GP5A**. Triazole **31a** (115 mg, 70%) was obtained as a white solid after filtration and precipitation from hexane.

m.p.: 86–91 °C (decomp.). ^{1}H NMR (400 MHz, $CDCl_3$) δ: 1.42 (s, 9H), 1.50 (d, 3H, J = 6.9 Hz), 5.22–5.42 (m br, 2H), 5.56 (s, 2H), 7.27–7.32 (m, 2H), 7.37–7.43 (m, 3H), 8.01 (s, 1H). ^{13}C NMR (101 MHz, $CDCl_3$) δ: 19.0, 28.3, 52.9, 54.5, 79.6, 126.5, 128.4, 129.2, 129.3, 133.4, 145.6, 155.1, 193.7. HRMS (ESI): *m/z* $[M+H]^+$ calculated for $C_{17}H_{22}N_4O_3$: 331.17647, found: 331.17648.

tert-butyl (S)-[1-oxo-1-(1-phenethyl-1H-1,2,3-triazol-4-yl)propan-2-yl]carbamate (31b)

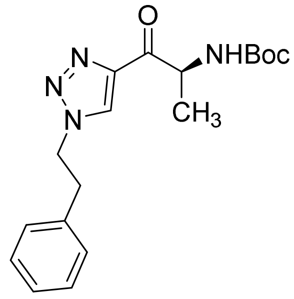

Step 1: To the solution of phenethyl amine (**30**) (61 mg, 0.5 mmol) and diethyl amine (183 mg, 258 μL, 2.5 mmol) in acetonitrile (1 mL) was added the solution of ADMP (172 mg, 0.6 mmol) in acetonitrile (1 mL) and the resulting solution was stirred at 30 °C for 1.25 h. Then it was diluted with DCM a washed with saturated aqueous $NaHCO_3$. Aqueous phase was further extracted with DCM, the combined organic phases were washed with brine and dried by MgSO4. Careful evaporation offered the crude phenethyl azide, which was used directly into next step.

Step 2: Crude phenethyl azide and alkynone **15** (89 mg, 0.45 mmol) were reacted according to **GP5B**. Purification by flash chromatography (SiO_2, 15–30 % EtOAc/hexane) gave triazole **31b** (120 mg, 78%) as an off-white solid.

m.p.: 99–101 °C. ^{1}H NMR (400 MHz, $CDCl_3$) δ: 1.43 (s, 9H), 1.48 (d, 3H, J = 7.0 Hz), 3.23 (t, 2H, J = 7.2 Hz), 4.58–4.72 (m, 2H), 5.22–5.34 (m br, 1H), 5.40 (d br, 1H, J = 7.6 Hz), 7.04–7.13 (m, 2H), 7.22–7.34 (m, 3H), 7.83 (s, 1H). ^{13}C NMR (101 MHz, $CDCl_3$) δ: 19.1, 28.3, 36.4, 51.9, 52.9, 79.6, 126.8, 127.4, 128.5, 129.0, 136.3, 145.0, 155.0, 193.5. HRMS (ESI): *m/z* $[M+H]^+$ calculated for $C_{18}H_{24}N_4O_3$: 345.19212, found: 345.19200.

***tert*-butyl (*S*)-[1-(1-(4-methoxyphenyl)-1*H*-1,2,3-triazol-4-yl)-1-oxopropan-2-yl]carbamate (27a)**

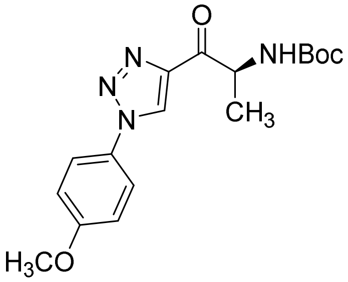

Step 1: The corresponding azide was prepared according to **GP4A** from *p*-anisidine (62 mg, 0.5 mmol), $NaNO_2$ (35 mg, 0.5 mmol) and NaN_3 (33 mg, 0.5 mmol). The obtained solution of crude azide in toluene was used directly into next step.

Step 2: Crude azide and alkynone **15** (99 mg, 0.5 mmol) were reacted according to **GP5B**. Purification by flash chromatography (SiO_2, 20–40 % EtOAc/hexane) and subsequent precipitation from hexane gave triazole **27a** (112 mg, 65%) as a white solid.

m.p.: 151–157 °C (decomp.). ^{1}H NMR (400 MHz, $CDCl_3$) δ: 1.44 (s, 9H), 1.55 (d, 3H, J = 6.6 Hz), 3.87 (s, 3H), 5.27–5.50 (m br, 2H), 7.04 (d, 2H, J = 9.1 Hz), 7.64 (d, 2H, J = 8.8 Hz), 8.47 (s, 1H). ^{13}C NMR (101 MHz, $CDCl_3$) δ: 19.0, 28.3, 53.0, 55.7, 79.7, 115.0, 122.4, 124.6, 129.6, 145.7, 155.1, 160.4, 193.8. HRMS (ESI): *m/z* $[M+H]^+$ calculated for $C_{17}H_{22}N_4O_4$: 347.17138, found: 347.17145.

***tert*-butyl (*S*)-[1-(1-(3-methoxyphenyl)-1*H*-1,2,3-triazol-4-yl)-1-oxopropan-2-yl]carbamate (27b)**

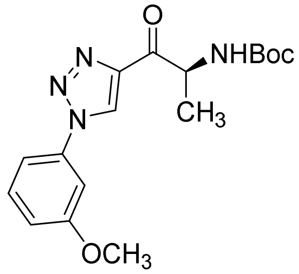

Step 1: 3-Methoxyphenyl azide was prepared according to **GP4B** from 3-bromoanisole (104 mg, 0.5 mmol), NaN_3 (65 mg, 1.0 mmol), CuI (10 mg, 0.05 mmol), sodium ascorbate (5 mg, 0.025 mmol) and DMEDA (7 mg, 9 µL, 0.075 mmol). Evaporation of solvents offered crude azide, which was used directly into next step.

Step 2: Crude azide and alkynone **15** (99 mg, 0.5 mmol) were reacted according to **GP5B**. Purification by flash chromatography (SiO_2, 20–40 % EtOAc/hexane) gave triazole **27b** (116 mg, 67%) as a yellowish syrup.

^{1}H NMR (400 MHz, $CDCl_3$) δ: 1.44 (s, 9H), 1.55 (d, 3H, J = 6.8 Hz), 3.88 (s, 3H), 5.22–5.53 (m br, 2H), 7.02 (ddd, 1H, J_1 = 8.4 Hz, J_2 = 2.5 Hz, J_3 = 0.8 Hz), 7.24–7.30 (m, 1H), 7.34 (t, 1H, J = 2.2 Hz), 7.44 (t, 1H, J = 8.2 Hz), 8.56 (s, 1H). ^{13}C NMR (101 MHz, $CDCl_3$) δ: 18.9, 28.3, 53.0, 55.7, 79.7, 106.6, 112.6, 115.4, 124.7, 130.8, 137.2, 145.7, 155.1, 160.7, 193.7. HRMS (ESI): *m/z* $[M+H]^+$ calculated for $C_{17}H_{22}N_4O_4$: 347.17139, found: 347.17139.

tert-butyl (_S_)-[1-(1-(2-methoxyphenyl)-1_H_-1,2,3-triazol-4-yl)-1-oxopropan-2-yl]carbamate (27c)

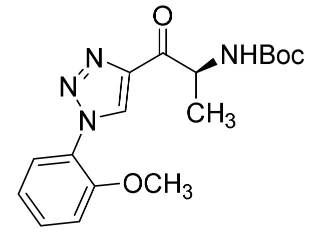

Step 1: The corresponding azide was prepared according to **GP4A** from *o*-anisidine (62 mg, 0.5 mmol), $NaNO_2$ (35 mg, 0.5 mmol) and NaN_3 (33 mg, 0.5 mmol). The obtained solution of crude azide in toluene was used directly into next step.

Step 2: Crude azide and alkynone **15** (99 mg, 0.5 mmol) were reacted according to **GP5B**. Purification by flash chromatography (SiO_2, 20–40 % EtOAc/hexane) gave triazole **27c** (152 mg, 88%) as a pale yellow thick oil, which solidified upon standing in a fridge.

m.p.: 118–120 °C. ^{1}H NMR (400 MHz, $CDCl_3$) δ: 1.45 (s, 9H), 1.57 (d, 3H, J = 6.8 Hz), 3.91 (s, 3H), 5.31–5.53 (m br, 2H), 7.08–7.17 (m, 2H), 7.43–7.50 (m, 1H), 7.83 (dd, 1H, J_1 = 7.9 Hz, J_2 = 1.7 Hz), 8.71 (s, 1H). ^{13}C NMR (101 MHz, $CDCl_3$) δ: 19.1, 28.3, 53.1, 56.0, 79.6, 112.3, 121.3, 125.2, 125.3, 128.7, 130.8, 144.9, 150.9, 155.1, 193.8. HRMS (ESI): *m/z* $[M+H]^+$ calculated for $C_{17}H_{22}N_4O_4$: 347.17138, found: 347.17142.

tert-butyl (_S_)-[1-(1-(4-_tert_-butylphenyl)-1_H_-1,2,3-triazol-4-yl)-1-oxopropan-2-yl]carbamate (27d)

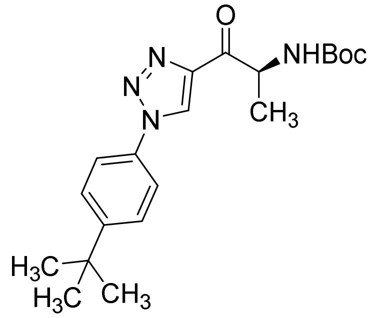

Step 1: The corresponding azide was prepared according to **GP4B** from 1-*tert*-butyl-4-iodobenzene (131 mg, 0.5 mmol), NaN_3 (65 mg, 1.0 mmol), CuI (10 mg, 0.05 mmol), sodium ascorbate (5 mg, 0.025 mmol) and DMEDA (7 mg, 9 µL, 0.075 mmol). Evaporation of solvents offered crude 1-azido-*tert*-butylbenzene (85 mg), which was used directly into next step.

Step 2: Crude azide and alkynone **15** (99 mg, 0.5 mmol) were reacted according to **GP5B**. Purification by flash chromatography (SiO_2, 10–25 % EtOAc/hexane) gave triazole **27d** (154 mg, 83%) as a white solid.

m.p.: >144 °C decomp. ^{1}H NMR (400 MHz, $CDCl_3$) δ: 1.37 (s, 9H), 1.45 (s, 9H), 1.56 (d, 3H, J = 6.6 Hz), 5.28–5.50 (m br, 2H), 7.53–7.59 (m, 2H), 7.63–7.69 (m, 2H), 8.52 (s, 1H). ^{13}C NMR (101 MHz, $CDCl_3$) δ: 19.0, 28.3, 31.2, 34.9, 53.0, 79.7, 120.5, 124.6, 126.9, 133.8, 145.7, 153.1, 155.1, 193.8. HRMS (ESI): *m/z* $[M+H]^+$ calculated for $C_{20}H_{28}N_4O_3$: 373.22342, found: 373.22356.

tert-butyl (_S_)-[1-(1-(4-methylphenyl)-1_H_-1,2,3-triazol-4-yl)-1-oxopropan-2-yl]carbamate (27e)

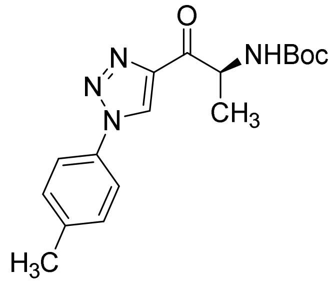

Step 1: The corresponding azide was prepared according to **GP4B** from 4-bromotoluene (86 mg, 0.5 mmol), NaN_3 (65 mg, 1.0 mmol), CuI (10 mg, 0.05 mmol), sodium ascorbate (5 mg, 0.025 mmol) and DMEDA (7 mg, 9 µL, 0.075 mmol). Evaporation of solvents offered crude 4-azidotoluene (60 mg), which was used directly into next step.

Step 2: Crude azide and alkynone **15** (89 mg, 0.45 mmol) were reacted according to **GP5B**. Purification by flash chromatography (SiO_2, 15–25 % EtOAc/hexane) gave triazole **27e** (125 mg, 76%) as a white solid.

m.p.: 147–148 °C. ^{1}H NMR (400 MHz, $CDCl_3$) δ: 1.44 (s, 9H), 1.56 (d, 3H, J = 6.6 Hz), 2.44 (s, 3H), 5.27–5.48 (m br, 2H), 7.35 (d, 2H, J = 8.1 Hz), 7.62 (d, 2H, J = 8.4 Hz), 8.52 (s, 1H). ^{13}C NMR (101 MHz, $CDCl_3$) δ: 19.0, 21.2, 28.3, 53.0, 79.7, 120.7, 124.6, 130.5, 133.9, 139.9, 145.7, 155.1, 193.8. HRMS (ESI): *m/z* $[M+H]^+$ calculated for $C_{17}H_{22}N_4O_3$: 331.17647, found: 331.17643.

***tert*-butyl (*S*)-[1-(1-(3,4-dimethylphenyl)-1*H*-1,2,3-triazol-4-yl)-1-oxopropan-2-yl]carbamate (27f)**

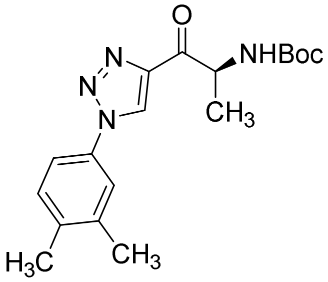

Step 1: The corresponding azide was prepared according to **GP4A** from 3,4-dimethylaniline (61 mg, 0.5 mmol), $NaNO_2$ (35 mg, 0.5 mmol) and NaN_3 (33 mg, 0.5 mmol). The obtained solution of crude azide in toluene was used directly into next step.

Step 2: Crude azide and alkynone **15** (99 mg, 0.5 mmol) were reacted according to **GP5B**. Purification by flash chromatography (SiO_2, 15–30 % EtOAc/hexane) and subsequent precipitation from hexane gave triazole **27f** (115 mg, 67%) as a white solid.

m.p.: 114–115 °C. ^{1}H NMR (400 MHz, $CDCl_3$) δ: 1.44 (s, 9H), 1.56 (d, 3H, J = 6.7 Hz), 2.33 (s, 3H), 2.35 (s, 3H), 5.29–5.51 (m br, 2H), 7.28 (d, 1H, J = 8.2 Hz), 7.43 (dd, 1H, J_1 = 8.1 Hz, J_2 = 2.4 Hz), 7.52 (d, 1H, J = 2.4 Hz), 8.50 (s, 1H). ^{13}C NMR (101 MHz, $CDCl_3$) δ: 19.0, 19.5, 19.9, 28.3, 53.0, 79.7, 118.1, 121.9, 124.6, 130.8, 134.1, 138.6, 138.7, 145.6, 155.1, 193.7. HRMS (ESI): *m/z* $[M+H]^+$ calculated for $C_{18}H_{24}N_4O_3$: 345.19212, found: 345.19205.

***tert*-butyl (*S*)-[1-(1-(2,4-dimethylphenyl)-1*H*-1,2,3-triazol-4-yl)-1-oxopropan-2-yl]carbamate (27g)**

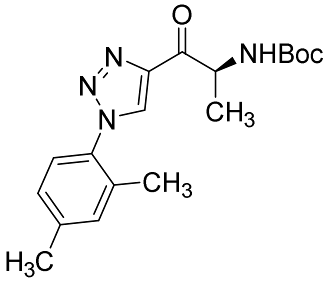

Step 1: The corresponding azide was prepared according to **GP4A** from 2,4-dimethylaniline (61 mg, 0.5 mmol), $NaNO_2$ (35 mg, 0.5 mmol) and NaN_3 (33 mg, 0.5 mmol). The obtained solution of crude azide in toluene was used directly into next step.

Step 2: Crude azide and alkynone **15** (99 mg, 0.5 mmol) were reacted according to **GP5B**. Purification by flash chromatography (SiO_2, 15–30 % EtOAc/hexane) and subsequent precipitation from hexane gave triazole **27g** (125 mg, 73 %) as a white solid.

m.p.: 104 – 105 °C. ^{1}H NMR (400 MHz, $CDCl_3$) δ: 1.45 (s, 9H), 1.58 (d, 3H, J = 6.6 Hz), 2.18 (s, 3H), 2.41 (s, 3H), 5.30–5.50 (m br, 2H), 7.12–7.18 (m, 1H), 7.18–7.23 (m, 2H), 8.28 (s, 1H). ^{13}C NMR (101 MHz, $CDCl_3$) δ: 17.8, 19.0, 21.2, 28.3, 53.1, 79.7, 125.6, 127.6, 128.1, 132.2, 133.1, 133.2, 140.7, 145.1, 155.1, 193.8. HRMS (ESI): *m/z* $[M+H]^+$ calculated for $C_{18}H_{24}N_4O_3$: 345.19212, found: 345.19197.

tert-butyl (S)-[1-(1-(2,6-dimethylphenyl)-1H-1,2,3-triazol-4-yl)-1-oxo-propan-2-yl]carbamate (27h)

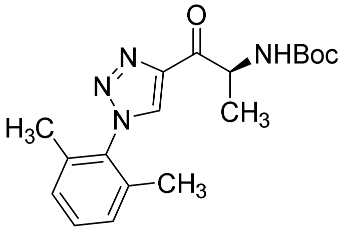

Step 1: The corresponding azide was prepared according to **GP4A** from 2,6-dimethylaniline (61 mg, 0.5 mmol), $NaNO_2$ (35 mg, 0.5 mmol) and NaN_3 (33 mg, 0.5 mmol). The obtained solution of crude azide in toluene was used directly into next step.

Step 2: Crude azide and alkynone **15** (99 mg, 0.5 mmol) were reacted according to **GP5B**. Purification by flash chromatography (SiO_2, 15–30 % EtOAc/hexane) and subsequent precipitation from hexane gave triazole **27h** (122 mg, 72 %) as a white solid.

m.p.: 105–112 °C. ^{1}H NMR (400 MHz, $CDCl_3$) δ: 1.45 (s, 9H), 1.60 (d, 3H, J = 6.2 Hz), 1.99 (s, 6 H), 5.30–5.51 (m br, 2H), 7.20 (d, 2H, J = 7.6 Hz), 7.35 (t, 1H, J = 7.6 Hz), 8.21 (s, 1H). ^{13}C NMR (101 MHz, $CDCl_3$) δ: 17.4, 19.1, 28.3, 53.2, 79.7, 128.5, 128.7, 130.5, 135.1, 145.2, 155.1, 193.7. HRMS (ESI): *m/z* $[M+H]^+$ calculated for $C_{18}H_{24}N_4O_3$: 345.19212, found: 345.19207.

tert-butyl (S)-[1-(1-(naphthalen-1-yl)-1H-1,2,3-triazol-4-yl)-1-oxopropan-2-yl]carbamate (27i)

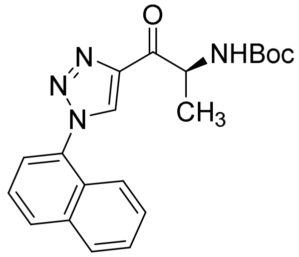

Step 1: Naphthyl azide was prepared according to **GP4B** from 1-bromonaphthalene (208 mg, 1.0 mmol), NaN_3 (130 mg, 2.0 mmol), CuI (20 mg, 0.1 mmol), sodium ascorbate (10 mg, 0.05 mmol) and DMEDA (14 mg, 17 µL, 0.15 mmol). The reaction mixture was refluxed for 4 h. Evaporation of solvents offered crude azide (ca 1:1 mixture with 1-bromonaphthalene according to NMR), which was used directly into next step.

Step 2: Crude azide (72 mg, 0.42 mmol) and alkynone **15** (83 mg, 0.42 mmol) were reacted according to **GP5B**. Purification by flash chromatography (SiO_2, 20–40 % EtOAc/hexane) gave triazole **27i** (106 mg, 58%) as a white solid.

m.p.: 98–111 °C. ^{1}H NMR (400 MHz, $CDCl_3$) δ: 1.47 (s, 9H), 1.64 (d, 3H, J = 6.9 Hz), 5.33–5.53 (m br, 2H), 7.54–7.65 (m, 5H), 7.96 – 8.01 (m, 1H), 8.07 (dd, 1H, J_1 = 7.0 Hz, J_2 = 2.6 Hz), 8.51 (s, 1H). ^{13}C NMR (101 MHz, $CDCl_3$) δ: 18.9, 28.3, 53.1, 79.7, 121.8, 123.6, 124.9, 127.3, 128.0, 128.2, 128.4, 129.1, 131.1, 132.7, 134.1, 145.3, 155.2, 193.8. HRMS (ESI): *m/z* $[M+H]^+$ calculated for $C_{20}H_{22}N_4O_3$: 367.17647, found: 367.17647.

tert-butyl (S)-[1-(1-(naphthalen-2-yl)-1H-1,2,3-triazol-4-yl)-1-oxopropan-2-yl]carbamate (27j)

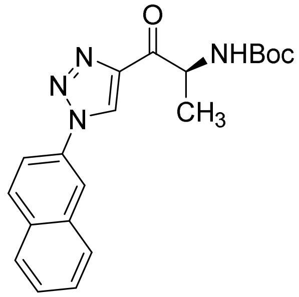

Step 1: The corresponding azide was prepared according to **GP4B** from 2-bromonaphthalene (208 mg, 1.0 mmol), NaN_3 (130 mg, 2.0 mmol), CuI (20 mg, 0.1 mmol), sodium ascorbate (10 mg, 0.05 mmol) and DMEDA (14 mg, 17 µL, 0.15 mmol), the mixture was refluxed for 4 h. Evaporation of solvents offered crude 2-azidonaphthalene (190 mg), which was used directly into next step.

Step 2: Crude azide and alkynone **15** (198 mg, 1.0 mmol) were reacted according to **GP5B**. Purification by flash chromatography (SiO_2, 10–35 % EtOAc/hexane) and subsequent precipitation from hexane gave triazole **27j** (315 mg, 86 %) as a white solid.

m.p.: 150–155 °C. ^{1}H NMR (400 MHz, $CDCl_3$) δ: 1.46 (s, 9H), 1.59 (d, 3H, J = 6.4 Hz), 5.32–5.53 (m br, 2H), 7.57–7.64 (m, 2H), 7.87 (dd, 1H, J_1 = 8.8 Hz, J_2 = 2.2 Hz), 7.90–7.97 (m, 2H), 8.03 (d, 1H, J = 8.9 Hz), 8.21 (d, 1H, J = 2.2 Hz), 8.69 (s, 1H). ^{13}C NMR (101 MHz, $CDCl_3$) δ: 19.0, 28.3, 53.1, 79.7, 118.7, 119.2, 124.8, 127.5, 127.7, 128.0, 128.4, 130.3, 133.1, 133.2, 133.5, 145.9, 155.1, 193.7. HRMS (ESI): *m/z* [M+H]$^+$ calculated for $C_{20}H_{22}N_4O_3$: 367.17647, found: 367.17677.

***tert*-butyl (*S*)-[1-oxo-1-(1-(pyridin-2-yl)-1*H*-1,2,3-triazol-4-yl)propan-2-yl]carbamate (27k)**

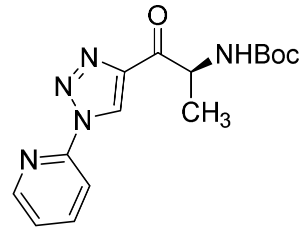

Step 1: The mixture of 2-bromopyridine (316 mg, 2.0 mmol), NaN_3 (260 mg, 4.0 mmol), CuI (40 mg, 0.2 mmol), sodium ascorbate (20 mg, 0.1 mmol), DMEDA (28 mg, 34 µl, 0.3 mmol) and EtOH/water (7:3, 5 mL) was refluxed for 1 h under argon atmosphere. Then, it was partitioned between EtOAc (30 mL) and water. Aqueous phase was further extracted with EtOAc (2 × 15 mL), the combined organic phases were washed with brine. Evaporation of solvents gave the tautomeric form of 2-azidopyridine, tetrazolo[1,5-*a*]pyridine (189 mg, 79%) as a white solid.

Step 2: CuTC (25 mg, 0.13 mmol) was added to the solution of tetrazolo[1,5-*a*]pyridine (160 mg, 1.33 mmol) and alkynone **15** (263 mg, 1.33 mmol) in toluene (8 mL). The resulting mixture was refluxed for 1 h under argon atmosphere followed by workup according to **GP5B**. Purification by flash chromatography (SiO_2, 20–40% EtOAc/hexane) gave triazole **27k** (198 mg, 47 %) as a white solid.

m.p.: 83–86 °C. ^{1}H NMR (400 MHz, $CDCl_3$) δ: 1.45 (s, 9H), 1.55 (d, 3H, J = 6.9 Hz), 5.27–5.55 (m br, 2H), 7.42 (ddd, 1H, J_1 = 7.5 Hz, J_2 = 4.8 Hz, J_3 = 1.0 Hz), 7.97 (td, 1H, J_1 = 7.8 Hz, J_2 = 1.8 Hz), 8.23 (d, 1H, J = 8.2 Hz), 8.51–8.56 (m, 1H), 9.14 (s, 1H). ^{13}C NMR (101 MHz, $CDCl_3$) δ: 19.1, 28.3, 53.1, 79.7, 114.1, 124.0, 124.4, 139.4, 145.3, 148.4, 149.0, 155.1, 193.5. HRMS (ESI): *m/z* [M+H]$^+$ calculated for $C_{15}H_{19}N_5O_3$: 318.15607, found: 318.15595.

***tert*-butyl (*S*)-[1-oxo-1-(1-(pyridin-3-yl)-1*H*-1,2,3-triazol-4-yl)propan-2-yl]carbamate (27l)**

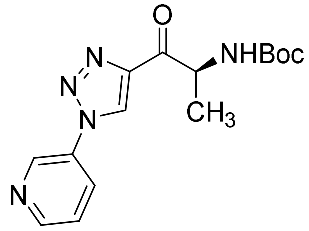

Step 1: The corresponding azide was prepared according to **GP4B** from 3-bromopyridine (80 mg, 0.5 mmol), NaN_3 (65 mg, 1.0 mmol), CuI (10 mg, 0.05 mmol), sodium ascorbate (5 mg, 0.025 mmol) and DMEDA (7 mg, 9 µL, 0.075 mmol). The mixture was extracted with toluene and crude azide was obtained as a solution in toluene and used directly into next step.

Step 2: Crude azide and alkynone **15** (99 mg, 0.5 mmol) were reacted according to **GP5B**. Purification by flash chromatography (SiO_2, 60–100 % EtOAc/hexane) gave triazole **27l** (106 mg, 67%) as a white solid.

m.p.: 138–144 °C (decomp.). ^{1}H NMR (400 MHz, $CDCl_3$) δ: 1.42 (s, 9H), 1.54 (d, 3H, J = 7.1 Hz), 5.24–5.41 (m br, 1H), 5.45 (d br, 1H, J = 7.8 Hz), 7.55 (dd, 1H, J_1 = 8.3 Hz, J_2 = 4.8 Hz), 8.16 (ddd, 1H, J_1 = 8.3 Hz, J_2 = 2.7 Hz, J_3 = 1.5 Hz), 8.69 (s, 1H), 8.76 (d, 1H, J = 4.8 Hz), 9.05 (s, 1H). ^{13}C NMR (101 MHz, $CDCl_3$) δ: 18.6, 28.3, 53.0, 79.8, 124.4, 124.8, 128.4, 133.0, 141.8, 146.2, 150.7, 155.1, 193.5. HRMS (ESI): *m/z* $[M+H]^+$ calculated for $C_{15}H_{19}N_5O_3$: 318.15607, found: 318.15591.

***tert*-butyl (*S*)-[1-oxo-1-(1-(pyridin-4-yl)-1*H*-1,2,3-triazol-4-yl)propan-2-yl]carbamate (27m)**

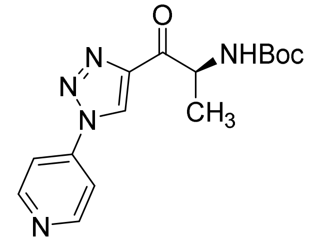

Step 1: The solution of 4-bromopyridine hydrochloride (98 mg, 0.5 mmol), NaN_3 (75 mg, 1.15 mmol) and NaOH (20 mg, 0.5 mmol) in EtOH/water (1:1, 2 mL) was refluxed for 3 hours. Then the mixture was diluted with saturated $NaHCO_3$ and extracted with DCM (3 × 5 mL), combined organic phases were washed with saturated $NaHCO_3$ and brine and dried by MgSO4. Evaporation of solvents offered crude 4-azidopyridine (50 mg), which was used directly into next step.

Step 2: Crude azide and alkynone **15** (83 mg, 0.42 mmol) were reacted according to **GP5B**. Purification by flash chromatography (SiO_2, EtOAc) gave triazole **27m** (105 mg, 66 %) as a pale yellow solid.

m.p.: >143 °C decomp. ^{1}H NMR (400 MHz, $CDCl_3$) δ: 1.44 (s, 9H), 1.55 (d, 3H, J = 7.0 Hz), 5.26–5.47 (m br, 2H), 7.73–7.79 (m, 2H), 8.71 (s, 1H), 8.81–8.88 (m, 2H). ^{13}C NMR (101 MHz, $CDCl_3$) δ: 18.6, 28.3, 53.1, 79.9, 114.0, 124.2, 142.4, 146.3, 152.0, 155.1, 193.5. HRMS (ESI): *m/z* $[M+H]^+$ calculated for $C_{15}H_{19}N_5O_3$: 318.15607, found: 318.15603.

***tert*-butyl (*S*)-[1-(1-(4-fluorophenyl)-1*H*-1,2,3-triazol-4-yl)-1-oxopropan-2-yl]carbamate (27n)**

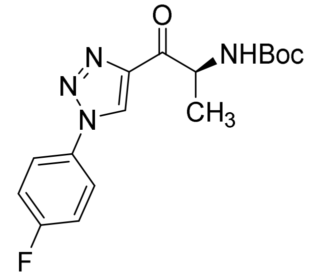

Step 1: The corresponding azide was prepared according to **GP4B** from 1-fluoro-4-iodobenzene (223 mg, 1.0 mmol), NaN_3 (130 mg, 2.0 mmol), CuI (20 mg, 0.1 mmol), sodium ascorbate (10 mg, 0.05 mmol) and DMEDA (14 mg, 17 µL, 0.15 mmol). The mixture was extracted with toluene and crude azide was obtained as a solution in toluene and used directly into next step.

Step 2: Crude azide and alkynone **15** (198 mg, 1.0 mmol) were reacted according to **GP5B**. Purification by flash chromatography (SiO_2, 15–35 % EtOAc/hexane) gave triazole **27n** (240 mg, 72%) as a white solid.

m.p.: 130–132 °C. ^{1}H NMR (400 MHz, $CDCl_3$) δ: 1.44 (s, 9H), 1.55 (d, 3H, J = 6.5 Hz), 5.27–5.51 (m br, 2H), 7.22–7.30 (m, 2H), 7.69–7.79 (m, 2H), 8.54 (s, 1H). ^{13}C NMR (101 MHz, $CDCl_3$) δ: 18.8, 28.3, 53.0, 79.8, 117.0 (d, J = 23.3 Hz), 122.9 (d, J = 8.8 Hz), 124.8, 132.5 (d, J = 3.3 Hz), 145.9, 155.1, 162.9 (d, J = 250.7 Hz), 193.7. ^{19}F NMR (282 MHz, $CDCl_3$) δ: -111.0 (m, 1F). HRMS (ESI): *m/z* $[M+H]^+$ calculated for $C_{16}H_{19}FN_4O_3$: 335.15140, found: 335.15144.

tert-butyl (_S_)-[1-oxo-1-(1-(4-(trifluoromethyl)phenyl)-1_H_-1,2,3-triazol-4-yl)propan-2-yl]carbamate (27o)

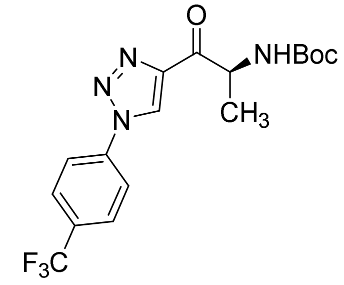

Step 1: The corresponding azide was prepared according to **GP4A** from 4-(trifluoromethyl)aniline (162 mg, 1.0 mmol), $NaNO_2$ (69 mg, 0.5 mmol) and NaN_3 (65 mg, 0.5 mmol). The obtained solution of crude azide in toluene was used directly into next step.

Step 2: Crude azide and alkynone **15** (198 mg, 1.0 mmol) were reacted according to **GP5B**. Purification by flash chromatography (SiO_2, 15–35% EtOAc/hexane) and subsequent precipitation from hexane gave triazole **27n** (252 mg, 66%) as an off-white solid.

m.p.: 145–146 °C. ^{1}H NMR (400 MHz, $CDCl_3$) δ: 1.44 (s, 9H), 1.56 (d, 3H, J = 6.9 Hz), 5.29–5.46 (m br, 2H), 7.85 (d, 2H, J = 8.5 Hz), 7.94 (d, 2H, J = 8.5 Hz), 8.65 (s, 1H). ^{13}C NMR (101 MHz, $CDCl_3$) δ: 18.7, 28.3, 53.1, 79.8, 120.9, 123.2 (q, J = 272.6 Hz), 124.6, 127.4 (q, J = 3.7 Hz), 131.7 (q, J = 33.3 Hz), 138.7, 146.2, 155.1, 193.6. ^{19}F NMR (282 MHz, $CDCl_3$) δ: -63.2 (s, 3F). HRMS (ESI): m/z $[M+H]^+$ calculated for $C_{17}H_{19}F_3N_4O_3$: 385.14820, found: 385.14821.

tert-butyl (_S_)-[1-(1-(3-nitrophenyl)-1_H_-1,2,3-triazol-4-yl)-1-oxopropan-2-yl]carbamate (27p)

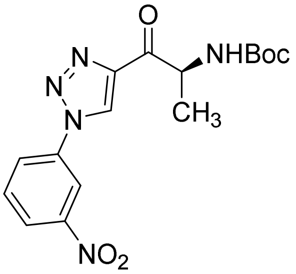

Step 1: The corresponding azide was prepared according to **GP4A** from 3-nitroaniline (70 mg, 0.5 mmol), $NaNO_2$ (35 mg, 0.5 mmol) and NaN_3 (33 mg, 0.5 mmol). The reaction mixture was extracted with EtOAc and organic phase was washed with saturated $NaHCO_3$ and brine and dried by $MgSO_4$. Evaporation of solvents offered the crude 3-nitroazidobenzene, which was used directly into next step.

Step 2: Crude azide and alkynone **15** (99 mg, 0.5 mmol) were reacted according to **GP5B**. Purification by flash chromatography (SiO_2, 20–40 % EtOAc/hexane) and subsequent precipitation from hexane gave triazole **27p** (125 mg, 69 %) as a pale yellow solid.

m.p.: 122–128 °C. ^{1}H NMR (400 MHz, $CDCl_3$) δ: 1.43 (s, 9H), 1.56 (d, 3H, J = 6.0 Hz), 5.19–5.52 (m br, 2H), 7.82 (t, 1H, J = 8.2 Hz), 8.20 (d, 1H, J = 8.3 Hz), 8.38 (d, 1H, J = 8.3 Hz), 8.68 (s, 1H), 8.76 (s, 1H). ^{13}C NMR (101 MHz, $CDCl_3$) δ: 18.6, 28.3, 53.1, 79.9, 115.8, 124.1, 124.7, 126.2, 131.3, 137.0, 146.4, 149.0, 155.2, 193.5. HRMS (ESI): m/z $[M+H]^+$ calculated for $C_{16}H_{19}N_5O_5$: 362.14590, found: 362.14611.

tert-butyl (_S_)-[1-(1-(4-nitrophenyl)-1_H_-1,2,3-triazol-4-yl)-1-oxopropan-2-yl]carbamate (27q)

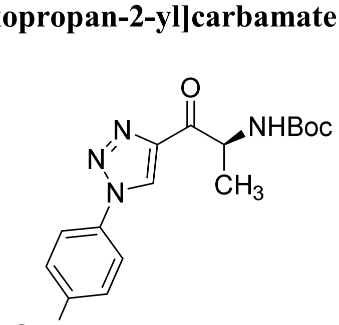

Step 1: The corresponding azide was prepared according to **GP4A** from 3-nitroaniline (70 mg, 0.5 mmol), $NaNO_2$ (35 mg, 0.5 mmol) and NaN_3 (33 mg, 0.5 mmol). The reaction mixture was extracted with EtOAc and organic phase was washed with saturated $NaHCO_3$ and brine and dried by $MgSO_4$. Evaporation of solvents offered the crude 4-nitroazidobenzene, which was used directly into next step.

Step 2: Crude azide and alkynone **15** (99 mg, 0.5 mmol) were reacted according to **GP5B**. Purification by flash chromatography (SiO_2, 20–40% EtOAc/hexane) and subsequent precipitation from hexane gave triazole **27q** (130 mg, 72%) as a pale yellow solid.

m.p.: 155–157 °C. ^{1}H NMR (400 MHz, $CDCl_3$) δ: 1.44 (s, 9H), 1.55 (d, 3H, J = 6.6 Hz), 5.25–5.46 (m br, 2H), 7.99–8.08 (m, 2H), 8.42–8.51 (m, 2H), 8.73 (s, 1H). ^{13}C NMR (101 MHz, $CDCl_3$) δ: 18.6, 28.3, 53.1, 79.9, 121.1, 124.7, 125.7, 140.4, 146.4, 147.8, 155.1, 193.5. HRMS (ESI): *m/z* $[M+H]^+$ calculated for $C_{16}H_{19}N_5O_5$: 362.14590, found: 362.14612.

***tert*-butyl (*S*)-[1-(1-(4-chlorophenyl)-1*H*-1,2,3-triazol-4-yl)-1-oxopropan-2-yl]carbamate (27r)**

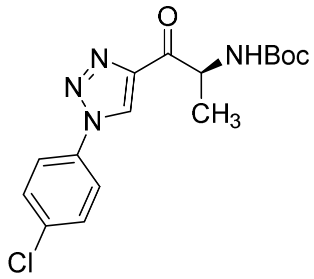

Step 1: The corresponding azide was prepared according to **GP4B** from 1-bromo-4-chlorobenzene (192 mg, 1.0 mmol), NaN_3 (130 mg, 2.0 mmol), CuI (20 mg, 0.1 mmol), sodium ascorbate (10 mg, 0.05 mmol) and DMEDA (14 mg, 17 µL, 0.15 mmol). The mixture was extracted with toluene and crude azide was obtained as a solution in toluene and used directly into next step.

Step 2: Crude azide and alkynone **15** (178 mg, 0.9 mmol) were reacted according to **GP5B**. Purification by flash chromatography (SiO_2, 20–35 % EtOAc/hexane) gave triazole **27r** (154 mg, 44%) as a white solid.

m.p.: 140–142 °C. ^{1}H NMR (400 MHz, $CDCl_3$) δ: 1.44 (s, 9H), 1.55 (d, 3H, J = 7.0 Hz), 5.28–5.45 (m br, 2H), 7.52–7.57 (m, 2H), 7.69–7.74 (m, 2H), 8.55 (s, 1H). ^{13}C NMR (101 MHz, $CDCl_3$) δ: 18.8, 28.3, 53.1, 79.8, 122.0, 124.5, 130.2, 134.7, 135.6, 146.0, 155.1, 193.6. HRMS (ESI): *m/z* $[M+H]^+$ calculated for $C_{16}H_{19}ClN_4O_3$: 351.12184, found: 351.12191.

***tert*-butyl (*S*)-[1-(1-(3-chlorophenyl)-1*H*-1,2,3-triazol-4-yl)-1-oxopropan-2-yl]carbamate (27s)**

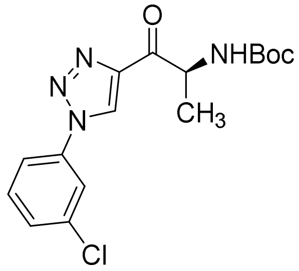

Step 1: The corresponding azide was prepared according to **GP4A** from 3-chloroaniline (64 mg, 0.5 mmol), $NaNO_2$ (35 mg, 0.5 mmol) and NaN_3 (33 mg, 0.5 mmol). The obtained solution of crude azide in toluene was used directly into next step.

Step 2: Crude azide and alkynone **15** (99 mg, 0.5 mmol) were reacted according to **GP5B**. Purification by flash chromatography (SiO_2, 20–40% EtOAc/hexane) and subsequent precipitation from hexane gave triazole **27s** (137 mg, 78%) as a pale yellow solid.

m.p.: 99–100 °C. ^{1}H NMR (400 MHz, $CDCl_3$) δ: 1.44 (s, 9H), 1.55 (d, 3H, J = 6.2 Hz), 5.30–5.45 (m br, 2H), 7.44–7.55 (m, 2H), 7.66 (dt, 1H, J_1 = 7.4 Hz, J_2 = 2.0 Hz), 7.82 (t, 1H, J = 2.0 Hz), 8.58 (s, 1H). ^{13}C NMR (101 MHz, $CDCl_3$) δ: 18.8, 28.3, 53.1, 79.8, 118.8, 121.2, 124.6, 129.8, 131.1, 135.9, 137.1, 146.0, 155.1, 193.6. HRMS (ESI): *m/z* $[M+H]^+$ calculated for $C_{16}H_{19}ClN_4O_3$: 351.12184, found: 351.12191.

tert-butyl (_S_)-[1-(1-(2-chlorophenyl)-1_H_-1,2,3-triazol-4-yl)-1-oxopropan-2-yl]carbamate (27t)

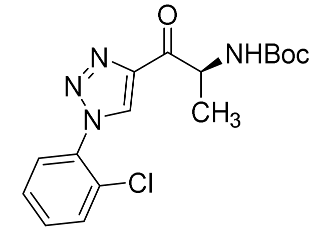

Step 1: The corresponding azide was prepared according to **GP4A** from 2-chloroaniline (64 mg, 0.5 mmol), $NaNO_2$ (35 mg, 0.5 mmol) and NaN_3 (33 mg, 0.5 mmol). The obtained solution of crude azide in toluene was used directly into next step.

Step 2: Crude azide and alkynone **15** (99 mg, 0.5 mmol) were reacted according to **GP5B**. Purification by flash chromatography (SiO_2, 20–40 % EtOAc/hexane) gave triazole **27t** (120 mg, 69%) as a pale yellow foam.

^{1}H NMR (400 MHz, $CDCl_3$) δ: 1.45 (s, 9H), 1.58 (d, 3H, J = 6.1 Hz), 5.26–5.52 (m br, 2H), 7.45–7.55 (m, 2H), 7.58–7.67 (m, 2H), 8.57 (s, 1H). ^{13}C NMR (101 MHz, $CDCl_3$) δ: 18.9, 28.3, 53.1, 79.7, 127.6, 128.1, 128.5, 128.7, 130.9, 131.4, 134.0, 145.1, 155.1, 193.6. HRMS (ESI): *m/z* $[M+H]^+$ calculated for $C_{16}H_{19}ClN_4O_3$: 351.12184, found: 351.12197.

tert-butyl (_S_)-[1-(1-(3,4-dichlorophenyl)-1_H_-1,2,3-triazol-4-yl)-1-oxopropan-2-yl]carbamate (27u)

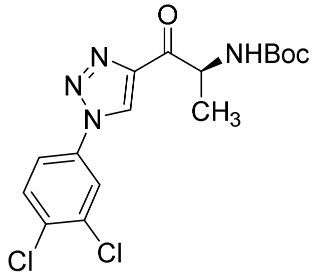

Step 1: 3,4-Dichlorophenyl azide was prepared according to **GP4B** from 3,4-dichloroiodobenzene (273 mg, 1.0 mmol), NaN_3 (130 mg, 2.0 mmol), CuI (20 mg, 0.1 mmol), sodium ascorbate (10 mg, 0.05 mmol) and DMEDA (14 mg, 17 μL, 0.15 mmol). Evaporation of solvents offered crude azide, which was used directly into next step.

Step 2: Crude azide and alkynone **15** (198 mg, 1.0 mmol) were reacted according to **GP5A**. Triazole **27u** (315 mg, 82%) was obtained as an off-white solid after filtration and precipitation from hexane.

m.p.: 130–134 °C (decomp.). ^{1}H NMR (400 MHz, $CDCl_3$) δ: 1.45 (s, 9H), 1.55 (d, 3H, J = 6.4 Hz), 5.37 (s br, 2H), 7.59–7.69 (m, 2H), 7.94 (d, 1H, J = 2.3 Hz), 8.55 (s, 1H). ^{13}C NMR (101 MHz, $CDCl_3$) δ: 18.7, 28.3, 53.0, 79.8, 119.7, 122.6, 124.5, 131.7, 134.0, 134.3, 135.2, 146.1, 155.1, 193.6. HRMS (ESI): *m/z* $[M+H]^+$ calculated for $C_{16}H_{18}Cl_2N_4O_3$: 385.08287, found: 385.08301.

tert-butyl (_S_)-[1-(1-(3-chloro-4-fluorophenyl)-1_H_-1,2,3-triazol-4-yl)-1-oxopropan-2-yl]carbamate (27v)

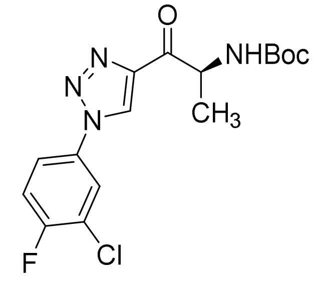

Step 1: The corresponding azide was prepared according to **GP4A** from 3-chloro-4-fluoroaniline (146 mg, 1.0 mmol), $NaNO_2$ (69 mg, 1.0 mmol) and NaN_3 (65 mg, 1.0 mmol). The obtained solution of crude azide in toluene was used directly into next step.

Step 2: Crude azide and alkynone **15** (198 mg, 1.0 mmol) were reacted according to **GP5B**. Purification by flash chromatography (SiO_2, 20–40% EtOAc/hexane) and subsequent precipitation from hexane gave triazole **27v** (283 mg, 77%) as a white solid.

m.p.: 124–125 °C. ^{1}H NMR (400 MHz, $CDCl_3$) δ: 1.44 (s, 9H), 1.55 (d, 3H, J = 7.0 Hz), 5.22–5.46 (m br, 2H), 7.35 (t, 1H, J = 8.5 Hz), 7.66 (ddd, 1H, J_1 = 9.0 Hz, J_2 = 3.9 Hz, J_3 = 2.7 Hz), 7.89 (dd, 1H, J_1 = 6.2 Hz, J_2 = 2.7 Hz), 8.56 (s, 1H). ^{13}C NMR (101 MHz, $CDCl_3$)

δ: 18.7, 28.3, 53.0, 79.8, 117.9 (d, J = 23.0 Hz), 120.6 (d, J = 7.9 Hz), 123.0 (d, J = 19.4 Hz), 123.5, 124.7, 132.8 (d, J = 3.9 Hz), 146.1, 155.1, 158.5 (d, J = 253.3 Hz), 193.6. ^{19}F NMR (282 MHz, $CDCl_3$) δ: -113.1 (m, 1F). HRMS (ESI): *m/z* $[M+H]^+$ calculated for $C_{16}H_{18}ClFN_4O_3$: 369.11242, found: 369.11258.

***tert*-butyl (*S*)-[1-(1-(2-chloro-4-fluorophenyl)-1*H*-1,2,3-triazol-4-yl)-1-oxopropan-2-yl]carbamate (35a)**

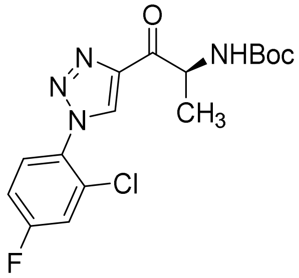

Step 1: The corresponding azide was prepared according to **GP4A** from 2-chloro-4-fluoroaniline (73 mg, 0.5 mmol), $NaNO_2$ (35 mg, 0.5 mmol) and NaN_3 (33 mg, 0.5 mmol). The obtained solution of crude azide in toluene was used directly into next step.

Step 2: Crude azide and alkynone **15** (99 mg, 0.5 mmol) were reacted according to **GP5B**. Purification by flash chromatography (SiO_2, 15–30 % EtOAc/hexane) gave triazole **35a** (150 mg, 81%) as a white solid.

m.p.: >140 °C decomp. ^{1}H NMR (400 MHz, $CDCl_3$) δ: 1.45 (s, 9H), 1.58 (d, 3H, J = 6.5 Hz), 5.31–5.46 (m br, 2H), 7.22 (ddd, 1H, J_1 = 8.8 Hz, J_2 = 7.4 Hz, J_3 = 2.7 Hz), 7.37 (dd, 1H, J_1 = 7.9 Hz, J_2 = 2.8 Hz), 7.63 (dd, 1H, J_1 = 8.9 Hz, J_2 = 5.3 Hz), 8.52 (s, 1H). ^{13}C NMR (101 MHz, $CDCl_3$) δ: 18.8, 28.3, 53.1, 79.7, 115.6 (d, J = 22.9 Hz), 118.3 (d, J = 26.1 Hz), 128.7, 129.1 (d, J = 9.7 Hz), 130.1 (d, J = 10.9 Hz), 130.5 (d, J = 3.8 Hz), 145.2, 155.1, 162.9 (d, J = 255.5 Hz), 193.6. ^{19}F NMR (282 MHz, $CDCl_3$) δ: -107.2 (m, 1F). HRMS (ESI): *m/z* $[M+H]^+$ calculated for $C_{16}H_{18}ClFN_4O_3$: 369.11242, found: 369.11247.

***tert*-butyl (*S*)-[1-(1-(2-chloro-4-fluorophenyl)-1*H*-1,2,3-triazol-4-yl)-1-oxobutan-2-yl]carbamate (35b)**

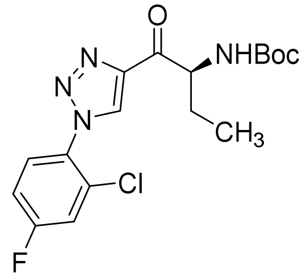

Step 1: The corresponding azide was prepared in the same way as for **35a**.

Step 2: Crude azide and alkynone **18e** (106 mg, 0.5 mmol) were reacted according to **GP5B**. Purification by flash chromatography (SiO_2, 15–30% EtOAc/hexane) gave triazole **35b** (158 mg, 83%) as a white solid.

m.p.: 91–111 °C (decomp.). ^{1}H NMR (400 MHz, $CDCl_3$) δ: 1.01 (t, 3H, J = 7.4 Hz), 1.45 (s, 9H), 1.83 (dp, 1H, J_1 = 14.5 Hz, J_2 = 7.3 Hz), 2.08–2.26 (m br, 1H), 5.25–5.44 (m br, 2H), 7.21 (ddd, 1H, J_1 = 8.9 Hz, J_2 = 7.4 Hz, J_3 = 2.7 Hz), 7.37 (dd, 1H, J_1 = 7.9 Hz, J_2 = 2.7 Hz), 7.63 (dd, 1H, J_1 = 8.9 Hz, J_2 = 5.3 Hz), 8.52 (s, 1H). ^{13}C NMR (101 MHz, $CDCl_3$) δ: 9.7, 25.8, 28.3, 58.3, 79.7, 115.5 (d, J = 22.8 Hz), 118.3 (d, J = 26.0 Hz), 128.6, 129.0 (d, J = 9.6 Hz), 130.1 (d, J = 10.8 Hz), 130.5 (d, J = 3.9 Hz), 145.6, 155.5, 162.9 (d, J = 255.6 Hz), 193.3. ^{19}F NMR (282 MHz, $CDCl_3$) δ: -107.2 (m, 1F). HRMS (ESI): *m/z* $[M+H]^+$ calculated for $C_{17}H_{20}ClFN_4O_3$: 383.12807, found: 383.12835.

tert-butyl (S)-[1-(1-(4-fluorophenyl)-1H-1,2,3-triazol-4-yl)-1-oxobutan-2-yl]carbamate (35c)

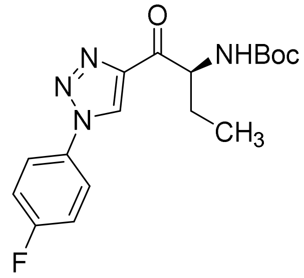

Step 1: The corresponding azide was prepared in the same way as for **27n**.

Step 2: Crude azide and alkynone **18e** (106 mg, 0.5 mmol) were reacted according to **GP5B**. Purification by flash chromatography (SiO_2, 15–30 % EtOAc/hexane) gave triazole **35c** (137 mg, 79%) as a white solid.

m.p.: 116–120 °C. ^{1}H NMR (400 MHz, $CDCl_3$) δ: 0.99 (t, 3H, J = 7.4 Hz), 1.45 (s, 9H), 1.83 (dp, 1H, J_1 = 14.5 Hz, J_2 = 7.3 Hz), 2.06 – 2.24 (m br, 1H), 5.22 – 5.46 (m br, 2H), 7.22–7.31 (m, 2H), 7.69–7.79 (m, 2H), 8.51 (s, 1H). ^{13}C NMR (101 MHz, $CDCl_3$) δ: 9.6, 25.8, 28.3, 58.2, 79.7, 117.0 (d, J = 23.5 Hz), 122.8 (d, J = 8.8 Hz), 124.7, 132.5 (d, J = 3.2 Hz), 146.3, 155.5, 162.9 (d, J = 250.7 Hz), 193.4. ^{19}F NMR (282 MHz, $CDCl_3$) δ: -111.0 (m, 1F). HRMS (ESI): *m/z* $[M+H]^+$ calculated for $C_{17}H_{21}FN_4O_3$: 349.16705, found: 349.16713.

1.6. Heterocyclic cathinones

(S)-2-amino-1-(1-phenyl-1H-1,2,3-triazol-4-yl)propan-1-on hydrochloride (12)

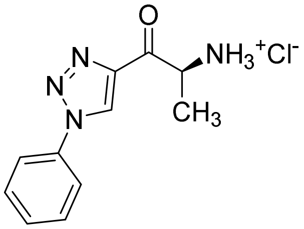

Prepared according to **GP7** from Boc-derivative **17** (159 mg, 0.5 mmol) and acetyl chloride (314 mg, 285 µL, 4.0 mmol) as an off-white solid (112 mg, 89 %) after recrystallization from EtOH/Et_2O.

m.p.: >178 °C decomp. ^{1}H NMR (400 MHz, CD_3OD) δ: 1.75 (d, 3H, J = 7.0 Hz), 5.08 (q, 1H, J = 7.4 Hz), 7.53 – 7.59 (m, 1H), 7.60–7.66 (m, 2H), 7.90–7.96 (m, 2H), 9.35 (s, 1H). ^{13}C NMR (101 MHz, CD_3OD) δ: 17.2, 54.0, 122.0, 127.7, 130.9, 131.1, 137.7, 145.7, 190.4. HRMS (ESI): *m/z* $[M+H]^+$ calculated for $C_{13}H_{14}NO_2$: 217.10839, found: 217.10829. Purity (HPLC/UV): 99.8 %.

(S)-2-amino-3-methyl-1-(1-phenyl-1H-1,2,3-triazol-4-yl)butan-1-on hydrochloride (21a)

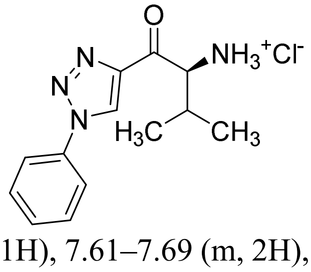

Prepared according to **GP7** from Boc-derivative **20a** (185 mg, 0.54 mmol) and acetyl chloride (339 mg, 308 µL, 4.32 mmol) as a white solid (142 mg, 94 %) after precipitation from acetonitrile.

m.p.: >182 °C decomp. ^{1}H NMR (400 MHz, DMSO-d_6) δ: 0.90 (d, 3H, J = 7.0 Hz), 1.08 (d, 3H, J = 7.0 Hz), 2.41–2.53 (m, 1H, interfering with solvent peak), 4.88 (d, 1H, J = 4.5 Hz), 7.54–7.60 (m, 1H), 7.61–7.69 (m, 2H), 7.97–8.05 (m, 2H), 8.67 (s br, 3H), 9.82 (s, 1H). ^{13}C NMR (101 MHz, DMSO-d_6) δ: 17.1, 19.1, 29.6, 60.1, 120.7, 127.3, 129.7, 130.0, 135.9, 145.3, 189.2. HRMS (ESI): *m/z* $[M+H]^+$ calculated for $C_{13}H_{16}N_4O$: 245.13969, found: 245.14084. Purity (HPLC/UV): 99.7 %.

(*S*)-2-amino-3-phenyl-1-(1-phenyl-1*H*-1,2,3-triazol-4-yl)propan-1-on hydrochloride (21b)

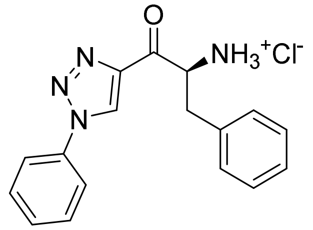

Prepared according to **GP7** from Boc-derivative **20b** (197 mg, 0.5 mmol) and acetyl chloride (314 mg, 285 µL, 4.0 mmol) as an off-white solid (155 mg, 95 %) after precipitation from acetonitrile.

m.p.: >174 °C decomp. ^{1}H NMR (400 MHz, DMSO-d_6) δ: 3.32–3.42 (m, 2H), 5.21 (t, 1H, J = 6.3 Hz), 7.17–7.32 (m, 5H), 7.53–7.60 (m, 1H), 7.61–7.69 (m, 2H), 7.94–8.01 (m, 2H), 8.77 (s br, 3H), 9.73 (s, 1H). ^{13}C NMR (101 MHz, DMSO-d_6) δ: 36.3, 56.6, 120.6, 127.2, 127.3, 128.5, 129.7, 129.7, 130.0, 134.4, 135.8, 144.8, 188.6. HRMS (ESI): *m/z* $[M+H]^+$ calculated for $C_{17}H_{16}N_4O$: 293.13969, found: 293.13974. Purity (HPLC/UV): 96.9 %.

(*S*)-2-amino-4-phenyl-1-(1-phenyl-1*H*-1,2,3-triazol-4-yl)butan-1-on hydrochloride (21c)

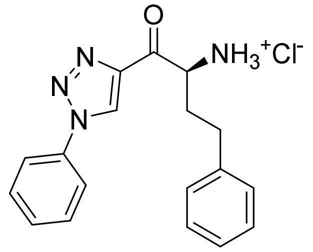

Prepared according to **GP7** from Boc-derivative **20c** (290 mg, 0.71 mmol) and acetyl chloride (446 mg, 405 µL, 5.68 mmol) as a white solid (232 mg, 95 %) after recrystallization from acetonitrile.

m.p.: >175 °C decomp. ^{1}H NMR (400 MHz, DMSO-d_6) δ: 2.20–2.42 (m, 2H), 2.70 (ddd, 1H, J_1 = 13.8 Hz, J_2 = 11.1 Hz, J_3 = 5.6 Hz), 2.81 (ddd, 1H, J_1 = 13.6 Hz, J_2 = 11.1 Hz, J_3 = 5.7 Hz), 5.02 (dd, 1H, J_1 = 7.2 Hz, J_2 = 4.4 Hz), 7.13 – 7.21 (m, 3H), 7.21 – 7.29 (m, 2H), 7.53 – 7.60 (m, 1H), 7.61 – 7.69 (m, 2H), 7.97 – 8.05 (m, 2H), 8.85 (s br, 3H), 9.74 (s, 1H). ^{13}C NMR (101 MHz, DMSO-d_6) δ: 30.3, 32.3, 55.6, 120.7, 126.2, 127.3, 128.3, 128.4, 129.7, 130.0, 135.9, 140.3, 144.6, 189.0. HRMS (ESI): *m/z* $[M+H]^+$ calculated for $C_{18}H_{18}N_4O$: 307.15534, found: 307.15497. Purity (HPLC/UV): 97.9 %.

(*S*)-2-amino-1-(1-phenyl-1*H*-1,2,3-triazol-4-yl)hexan-1-on hydrochloride (21d)

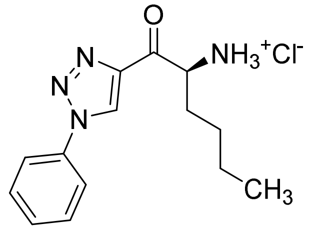

Prepared according to **GP7** from Boc-derivative **20d** (220 mg, 0.61 mmol) and acetyl chloride (383 mg, 348 µL, 4.88 mmol) as a white solid (176 mg, 97 %) after recrystallization from acetonitrile.

m.p.: >173 °C decomp. ^{1}H NMR (400 MHz, DMSO-d_6) δ: 0.83 (t, 3H, J = 7.1 Hz), 1.18 – 1.36 (m, 3H), 1.36 – 1.50 (m, 1H), 1.88–2.10 (m, 2H), 4.94 (dd, 1H, J_1 = 7.2 Hz, J_2 = 4.7 Hz), 7.53–7.60 (m, 1H), 7.61–7.69 (m, 2H), 7.97–8.05 (m, 2H), 8.69 (s br, 3H), 9.79 (s, 1H). ^{13}C NMR (101 MHz, DMSO-d_6) δ: 13.6, 21.8, 26.2, 30.2, 55.5, 120.7, 127.3, 129.7, 130.0, 135.9, 144.7, 189.4. HRMS (ESI): *m/z* $[M+H]^+$ calculated for $C_{14}H_{18}N_4O$: 259.15534, found: 259.15511. Purity (HPLC/UV): 99.9 %.

(*S*)-2-amino-1-(1-phenyl-1*H*-1,2,3-triazol-4-yl)butan-1-on hydrochloride (21e)

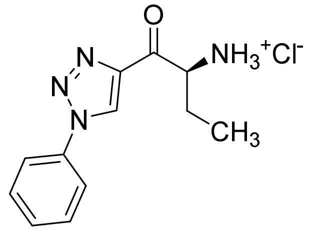

Prepared according to **GP7** from Boc-derivative **20e** (220 mg, 0.67 mmol) and acetyl chloride (421 mg, 382 µL, 5.36 mmol) as a white solid (173 mg, 97 %) after precipitation from acetonitrile.

m.p.: >179 °C decomp. ^{1}H NMR (400 MHz, DMSO-d_6) δ: 0.94 (t, 3H, J = 7.5 Hz), 1.95–2.18 (m, 2H), 4.92 (dd, 1H, J_1 = 6.5 Hz, J_2 = 4.8 Hz), 7.54–7.60 (m, 1H), 7.61 – 7.69 (m, 2H), 7.97–8.05 (m, 2H), 8.71 (s br, 3H), 9.81 (s, 1H). ^{13}C NMR (101 MHz, DMSO-d_6) δ: 9.0, 23.8, 56.6, 120.8, 127.3, 129.7, 130.0, 135.9,

144.6, 189.2. HRMS (ESI): *m/z* $[M+H]^+$ calculated for $C_{12}H_{14}N_4O$: 231.12404, found: 231.12390. Purity (HPLC/UV): 99.6 %.

(*S*)-2-amino-2-cyclopropyl-1-(1-phenyl-1*H*-1,2,3-triazol-4-yl)ethan-1-on hydrochloride (21f)

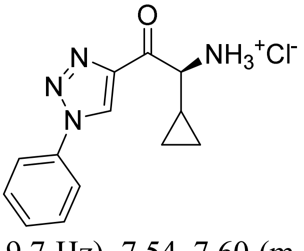

Prepared according to **GP7** from Boc-derivative **20f** (220 mg, 0.64 mmol) and acetyl chloride (402 mg, 365 µL, 5.12 mmol) as a white solid (170 mg, 95 %) after precipitation from acetonitrile.

m.p.: >183 °C decomp. ^{1}H NMR (400 MHz, DMSO-d_6) δ: 0.54 – 0.64 (m, 1H), 0.64–0.72 (m, 1H), 0.76–0.87 (m, 2H), 1.06 (dddd, 1H, J_1 = 12.8 Hz, J_2 = 9.7 Hz, J_3 = 8.1 Hz, J_4 = 4.7 Hz), 4.34 (d, 1H, J = 9.7 Hz), 7.54–7.60 (m, 1H), 7.61–7.69 (m, 2H), 7.98–8.06 (m, 2H), 8.79 (s br, 3H), 9.86 (s, 1H). ^{13}C NMR (101 MHz, DMSO-d_6) δ: 4.7, 4.9, 12.2, 58.8, 120.7, 127.6, 129.7, 130.1, 135.9, 145.2, 188.5. HRMS (ESI): *m/z* $[M+H]^+$ calculated for $C_{13}H_{14}N_4O$: 243.12404, found: 243.12380. Purity (HPLC/UV): 99.5 %.

(*S*)-2-amino-1-(1-benzyl-1*H*-1,2,3-triazol-4-yl)propan-1-on hydrochloride (28a)

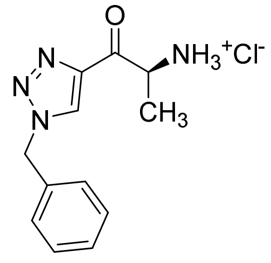

Prepared according to **GP7** from Boc-derivative **31a** (100 mg, 0.3 mmol) and acetyl chloride (189 mg, 172 µL, 2.4 mmol) as an off-white solid (65 mg, 82 %) after precipitation from acetonitrile.

m.p.: >182 °C decomp. ^{1}H NMR (400 MHz, DMSO-d_6) δ: 1.53 (d, 3H, J = 7.2 Hz), 4.73–4.94 (m br, 1H), 5.72 (s, 2H), 7.28 – 7.47 (m, 5H), 8.59 (s br, 3H), 9.19 (s, 1H). ^{13}C NMR (101 MHz, DMSO-d_6) δ: 16.6, 51.8, 53.3, 128.2, 128.5, 128.9, 129.2, 135.2, 143.6, 189.6. HRMS (ESI): *m/z* $[M+H]^+$ calculated for $C_{12}H_{14}N_4O$: 231.12404, found: 231.12383. Purity (HPLC/UV): 99.8 %.

(*S*)-2-amino-1-(1-phenethyl-1*H*-1,2,3-triazol-4-yl)propan-1-on hydrochloride (28b)

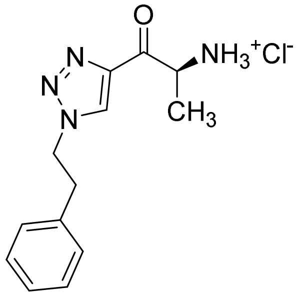

Prepared according to **GP7** from Boc-derivative **31b** (100 mg, 0.29 mmol) and acetyl chloride (182 mg, 165 µL, 2.32 mmol) as a white solid (72 mg, 88 %) after precipitation from acetonitrile.

m.p.: >172 °C decomp. ^{1}H NMR (400 MHz, DMSO-d_6) δ: 1.50 (d, 3H, J = 7.1 Hz), 3.22 (t, 2H, J = 7.2 Hz), 4.74 (t, 2H, J = 7.1 Hz), 4.81 (q, 1H, J = 7.2 Hz), 7.13–7.23 (m, 3H), 7.24–7.32 (m, 2H), 8.58 (s br, 3H), 8.98 (s, 1H). ^{13}C NMR (101 MHz, DMSO-d_6) δ: 16.5, 35.2, 50.9, 51.7, 126.7, 128.5, 128.7, 129.0, 137.2, 143.2, 189.6. HRMS (ESI): *m/z* $[M+H]^+$ calculated for $C_{13}H_{16}N_4O$: 245.13969, found: 245.13963. Purity (HPLC/UV): 99.9 %.

(*S*)-2-amino-1-[1-(4-methoxyphenyl)-1*H*-1,2,3-triazol-4-yl]propan-1-on hydrochloride (26a)

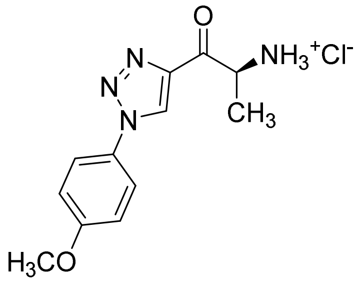

Prepared according to **GP7** from Boc-derivative **27a** (112 mg, 0.32 mmol) and acetyl chloride (201 mg, 180 µl, 2.56 mmol) as a white solid (74 mg, 82 %) after precipitation from *i*-PrOH.

m.p.: >180 °C decomp. ^{1}H NMR (400 MHz, DMSO-d_6) δ: 1.59 (d, 3H, J = 7.2 Hz), 3.84 (s, 3H), 4.91 (q, 1H, J = 7.2 Hz),

7.13–7.22 (m, 2H), 7.87–7.95 (m, 2H), 8.68 (s br, 3H), 9.67 (s, 1H). ^{13}C NMR (101 MHz, DMSO-d_6) δ: 16.6, 51.9, 55.7, 115.0, 122.4, 127.1, 129.2, 144.0, 160.0, 189.6. HRMS (ESI): *m/z* [M+H]$^+$ calculated for $C_{12}H_{14}N_4O_2$: 247.11895, found: 247.11880. Purity (HPLC/UV): 98.7 %.

(*S*)-2-amino-1-[1-(3-methoxyphenyl)-1*H*-1,2,3-triazol-4-yl]propan-1-on hydrochloride (26b)

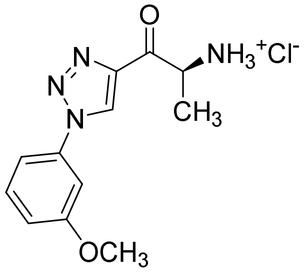

Prepared according to **GP7** from Boc-derivative **27b** (116 mg, 0.33 mmol) and acetyl chloride (208 mg, 190 µL, 2.64 mmol) as a white solid (60 mg, 65 %) after precipitation from acetonitrile.

m.p.: >171 °C decomp. ^{1}H NMR (400 MHz, DMSO-d_6) δ: 1.59 (d, 3H, J = 7.3 Hz), 3.87 (s, 3H), 4.92 (q, 1H, J = 7.2 Hz), 7.09–7.17 (m, 1H), 7.50–7.63 (m, 3H), 8.68 (s br, 3H), 9.82 (s, 1H). ^{13}C NMR (101 MHz, DMSO-d_6) δ: 16.6, 52.0, 55.8, 106.2, 112.6, 115.5, 127.5, 131.0, 136.9, 144.1, 160.2, 189.5. HRMS (ESI): *m/z* [M+H]$^+$ calculated for $C_{12}H_{14}N_4O_2$: 247.11895, found: 247.11881. Purity (HPLC/UV): 99.5 %.

(*S*)-2-amino-1-[1-(2-methoxyphenyl)-1*H*-1,2,3-triazol-4-yl]propan-1-on hydrochloride (26c)

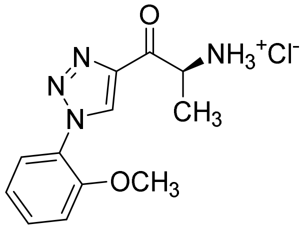

Prepared according to **GP7** from Boc-derivative **27c** (126 mg, 0.36 mmol) and acetyl chloride (226 mg, 205 µL, 2.88 mmol) as a white solid (80 mg, 79 %) after precipitation from acetonitrile.

m.p.: >168 °C decomp. ^{1}H NMR (400 MHz, DMSO-d_6) δ: 1.60 (d, 3H, J = 7.2 Hz), 3.86 (s, 3H), 4.92 (q br, 1H, J = 7.2 Hz), 7.18 (td, 1H, J_1 = 7.7 Hz, J_2 = 1.2 Hz), 7.37 (dd, 1H, J_1 = 8.5 Hz, J_2 = 1.2 Hz), 7.61 (ddd, 1H, J_1 = 8.6 Hz, J_2 = 7.5 Hz, J_3 = 1.7 Hz), 7.68 (dd, 1H, J_1 = 7.9 Hz, J_2 = 1.7 Hz), 8.65 (s br, 3H), 9.36 (s, 1H). ^{13}C NMR (101 MHz, DMSO-d_6) δ: 16.6, 51.9, 56.3, 113.1, 120.9, 124.7, 126.2, 130.9, 131.7, 143.3, 152.0, 189.6. HRMS (ESI): *m/z* [M+H]$^+$ calculated for $C_{12}H_{14}N_4O_2$: 247.11895, found: 247.11888. Purity (HPLC/UV): 99.0 %.

(*S*)-2-amino-1-[1-(4-*tert*-butylphenyl)-1*H*-1,2,3-triazol-4-yl]propan-1-on hydrochloride (26d)

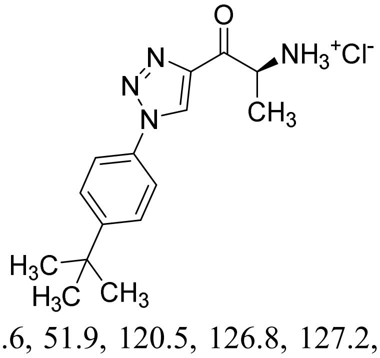

Prepared according to **GP7** from Boc-derivative **27d** (134 mg, 0.36 mmol) and acetyl chloride (226 mg, 206 µL, 2.88 mmol) as a white solid (99 mg, 89 %) after precipitation from acetonitrile.

m.p.: >181 °C decomp. ^{1}H NMR (400 MHz, DMSO-d_6) δ: 1.33 (s, 9H), 1.60 (d, 3H, J = 7.2 Hz), 4.92 (q, 1H, J = 7.2 Hz), 7.65 (d, 2H, J = 8.5 Hz), 7.91 (d, 2H, J = 8.5 Hz), 8.69 (s br, 3H), 9.74 (s, 1H). ^{13}C NMR (101 MHz, DMSO-d_6) δ: 16.6, 31.0, 34.6, 51.9, 120.5, 126.8, 127.2, 133.6, 144.1, 152.4, 189.5. HRMS (ESI): *m/z* [M+H]$^+$ calculated for $C_{15}H_{20}N_4O$: 273.17099, found: 273.17207. Purity (HPLC/UV): 98.9 %.

(*S*)-2-amino-1-[1-(4-methylphenyl)-1*H*-1,2,3-triazol-4-yl]propan-1-on hydrochloride (26e)

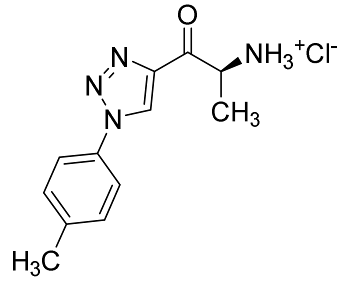

Prepared according to **GP7** from Boc-derivative **27e** (103 mg, 0.31 mmol) and acetyl chloride (194 mg, 177 µL, 2.48 mmol) as a white solid (70 mg, 85 %) after precipitation from acetonitrile.

m.p.: >184 °C decomp. ^{1}H NMR (400 MHz, DMSO-d_6) δ: 1.59 (d, 3H, J = 7.2 Hz), 2.39 (s, 3H), 4.91 (q, 1H, J = 7.2 Hz), 7.44 (d, 2H, J = 8.3 Hz), 7.88 (d, 2H, J = 8.3 Hz), 8.69 (s br, 3H), 9.74 (s, 1H). ^{13}C NMR (101 MHz, DMSO-d_6) δ: 16.6, 20.7, 51.9, 120.6, 127.2, 130.4, 133.6, 139.5, 144.1, 189.5. HRMS (ESI): *m/z* [M+H]$^+$ calculated for $C_{12}H_{14}N_4O$: 231.12404, found: 231.12399. Purity (HPLC/UV): 99.6 %.

(*S*)-2-amino-1-[1-(3,4-dimethylphenyl)-1*H*-1,2,3-triazol-4-yl]propan-1-on hydrochloride (26f)

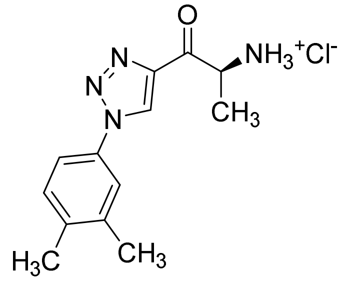

Prepared according to **GP7** from Boc-derivative **27f** (93 mg, 0.27 mmol) and acetyl chloride (170 mg, 154 µL, 2.16 mmol) as a white solid (55 mg, 72 %) after precipitation from acetonitrile.

m.p.: >170 °C decomp. ^{1}H NMR (400 MHz, DMSO-d_6) δ: 1.59 (d, 3H, J = 7.2 Hz), 2.30 (s, 3H), 2.33 (s, 3H), 4.91 (q, 1H, J = 7.2 Hz), 7.38 (d, 1H, J = 8.2 Hz), 7.70 (dd, 1H, J_1 = 8.2 Hz, J_2 = 2.4 Hz), 7.81 (d, 1H, J = 2.4 Hz), 8.68 (s br, 3H), 9.70 (s, 1H). ^{13}C NMR (101 MHz, DMSO-d_6) δ: 16.6, 19.0, 19.4, 51.9, 117.9, 121.4, 127.0, 130.6, 133.7, 138.2, 138.3, 144.0, 189.5. HRMS (ESI): *m/z* [M+H]$^+$ calculated for $C_{13}H_{16}N_4O$: 245.13969, found: 245.14067. Purity (HPLC/UV): 98.8 %.

(*S*)-2-amino-1-[1-(2,4-dimethylphenyl)-1*H*-1,2,3-triazol-4-yl]propan-1-on hydrochloride (26g)

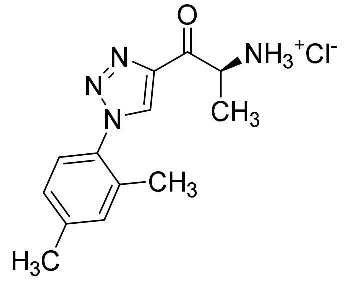

Prepared according to **GP7** from Boc-derivative **27g** (103 mg, 0.30 mmol) and acetyl chloride (189 mg, 171 µL, 2.40 mmol) as a white solid (60 mg, 71 %) after precipitation from acetonitrile.

m.p.: >175 °C decomp. ^{1}H NMR (400 MHz, DMSO-d_6) δ: 1.60 (d, 3H, J = 7.1 Hz), 2.13 (s, 3H), 2.38 (s, 3H), 4.92 (q, 1H, J = 7.1 Hz), 7.25 (d, 1H, J = 8.1 Hz), 7.33 (s, 1H), 7.40 (d, 1H, J = 8.0 Hz), 8.65 (s br, 3H), 9.42 (s, 1H). ^{13}C NMR (101 MHz, DMSO-d_6) δ: 16.6, 17.2, 20.7, 52.0, 126.0, 127.5, 130.6, 131.9, 132.8, 133.0, 140.3, 143.4, 189.6. HRMS (ESI): *m/z* [M+H]$^+$ calculated for $C_{13}H_{16}N_4O$: 245.13969, found: 245.14015. Purity (HPLC/UV): 99.8 %.

(*S*)-2-amino-1-[1-(2,6-dimethylphenyl)-1*H*-1,2,3-triazol-4-yl]propan-1-on hydrochloride (26h)

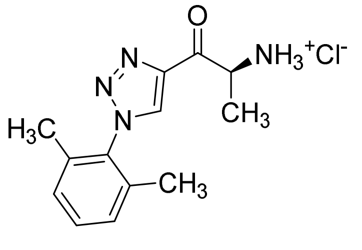

Prepared according to **GP7** from Boc-derivative **27h** (108 mg, 0.31 mmol) and acetyl chloride (194 mg, 177 µL, 2.48 mmol) as a white solid (61 mg, 70 %) after precipitation from acetonitrile.

m.p.: >176 °C decomp. ^{1}H NMR (400 MHz, DMSO-d_6) δ: 1.61 (d, 3H, J = 7.2 Hz), 1,96 (s, 6H), 2.38 (s, 3H), 4.95 (q, 1H,

J = 7.2 Hz), 7.34 (d, 2H, J = 7.6 Hz), 7.46 (dd, 1H, J_1 = 8.2 Hz, J_2 = 7.0 Hz), 8.62 (s br, 3H), 9.40 (s, 1H). ^{13}C NMR (101 MHz, DMSO-d_6) δ: 16.6, 16.9, 52.0, 128.6, 130.6, 131.1, 134.7, 134.9, 143.6, 189.6. HRMS (ESI): m/z $[M+H]^+$ calculated for $C_{13}H_{16}N_4O$: 245.13969, found: 245.13959. Purity (HPLC/UV): 99.3 %.

(*S*)-2-amino-1-[1-(naphthalen-1-yl)-1*H*-1,2,3-triazol-4-yl]propan-1-on hydrochloride (26i)

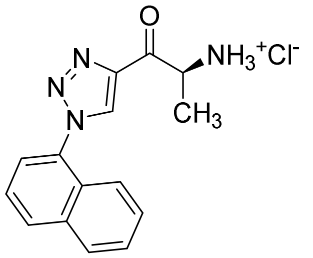

Prepared according to **GP7** from Boc-derivative **27i** (140 mg, 0.38 mmol) and acetyl chloride (237 mg, 218 µL, 3.04 mmol) as a white solid (104 mg, 90 %) after precipitation from acetonitrile.

m.p.: >172 °C decomp. ^{1}H NMR (400 MHz, DMSO-d_6) δ: 1.66 (d, 3H, J = 7.2 Hz), 4.99 (q, 1H, J = 7.2 Hz), 7.48 (d, 1H, J = 8.4 Hz), 7.62–7.79 (m, 3H), 7.85 (dd, 1H, J_1 = 7.3 Hz, J_2 = 1.2 Hz), 8.17 (dd, 1H, J_1 = 8.0 Hz, J_2 = 1.5 Hz), 8.27 (d, 1H, J = 8.2 Hz), 8.62 (s br, 3H), 9.63 (s, 1H). ^{13}C NMR (101 MHz, DMSO-d_6) δ: 16.7, 52.1, 121.8, 124.5, 125.5, 127.4, 127.7, 128.3, 128.5, 131.1, 131.8, 132.4, 133.6, 143.6, 189.6. HRMS (ESI): m/z $[M+H]^+$ calculated for $C_{15}H_{14}N_4O$: 267.12404, found: 267.12486. Purity (HPLC/UV): 99.7 %.

(*S*)-2-amino-1-[1-(naphthalen-2-yl)-1*H*-1,2,3-triazol-4-yl]propan-1-on hydrochloride (26j)

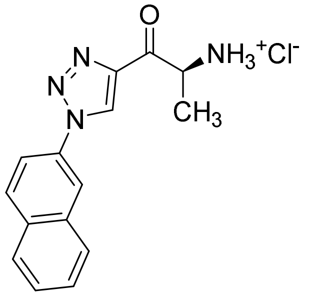

Prepared according to **GP7** from Boc-derivative **27j** (110 mg, 0.30 mmol) and acetyl chloride (189 mg, 171 µL, 2.40 mmol) as a white solid (83 mg, 91 %) after precipitation from acetonitrile.

m.p.: >182 °C decomp. ^{1}H NMR (400 MHz, DMSO-d_6) δ: 1.63 (d, 3H, J = 7.2 Hz), 4.95 (q, 1H, J = 7.2 Hz), 7.61–7.70 (m, 2H), 8.03–8.11 (m, 2H), 8.15 (dd, 1H, J_1 = 8.9 Hz, J_2 = 2.2 Hz), 8.21 (d, 1H, J_1 = 8.9 Hz), 8.64 (d, 1H, J = 2.2 Hz), 8.72 (s br, 3H), 9.92 (s, 1H). ^{13}C NMR (101 MHz, DMSO-d_6) δ: 16.6, 52.0, 118.8, 118.9, 127.5, 127.5, 127.7, 128.0, 128.4, 130.1, 132.7, 132.7, 133.3, 144.2, 189.5. HRMS (ESI): m/z $[M+H]^+$ calculated for $C_{15}H_{14}N_4O$: 267.12404, found: 267.12394. Purity (HPLC/UV): 96.9 %.

(*S*)-2-amino-1-[1-(pyridin-2-yl)-1*H*-1,2,3-triazol-4-yl]propan-1-on hydrochloride (26k)

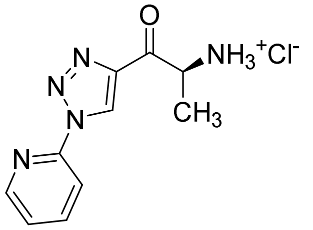

Prepared according to **GP7** from Boc-derivative **27k** (200 mg, 0.63 mmol) and acetyl chloride (396 mg, 360 µL, 5.04 mmol) as a white solid (126 mg, 79 %) after recrystallization from acetonitrile.

m.p.: >198 °C decomp. ^{1}H NMR (400 MHz, DMSO-d_6) δ: 1.57 (d, 3H, J = 7.2 Hz), 4.97 (q, 1H, J = 7.2 Hz), 7.65 (h, 1H, J = 4.6 Hz), 8.14–8.24 (m, 2H), 8.67 (dt, 1H, J_1 = 4.8 Hz, J_2 = 1.4 Hz), 8.72 (s br, 3H), 9.70 (s, 1H). ^{13}C NMR (101 MHz, DMSO-d_6) δ: 16.6, 52.0, 114.8, 125.4, 126.2, 140.5, 143.8, 147.8, 149.3, 189.4. HRMS (ESI): m/z $[M+H]^+$ calculated for $C_{10}H_{11}N_5O$: 218.10364, found: 218.10469. Purity (HPLC/UV): 99.2 %.

(*S*)-2-amino-1-[1-(pyridin-3-yl)-1*H*-1,2,3-triazol-4-yl]propan-1-on dihydrochloride (26l)

Prepared according to **GP7** from Boc-derivative **27l** (65 mg, 0.20 mmol) and acetyl chloride (126 mg, 115 µL, 1.60 mmol) as a white solid (51 mg, 88 %) after precipitation from acetonitrile.

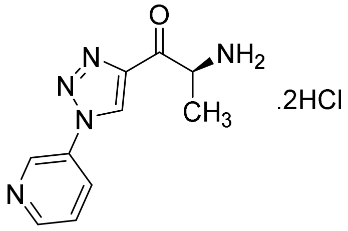

m.p.: 140–143 °C (decomp.). ^{1}H NMR (400 MHz, DMSO-d_6) δ: 1.60 (d, 3H, J = 7.2 Hz), 4.83–4.99 (m br, 1H), 7.45 (s br, 1H), 7.80 (dd, 1H, J_1 = 8.4 Hz, J_2 = 4.7 Hz), 8.54 (ddd, 1H, J_1 = 8.4 Hz, J_2 = 2.5 Hz, J_3 = 1.2 Hz), 8.72 (d br, 3H, J = 5.4 Hz), 8.82 (s br, 1H), 9.30 (s br, 1H), 9.90 (s, 1H). ^{13}C NMR (101 MHz, DMSO-d_6) δ: 16.5, 52.0, 125.2, 128.2, 130.0, 133.1, 140.9, 144.3, 149.3, 189.4. HRMS (ESI): *m/z* [M+H]$^+$ calculated for $C_{10}H_{11}N_5O$: 218.10364, found: 218.10384. Purity (HPLC/UV): 97.3 %.

(*S*)-2-amino-1-[1-(pyridin-4-yl)-1*H*-1,2,3-triazol-4-yl]propan-1-on dihydrochloride (26m)

Prepared according to **GP7** from Boc-derivative **27m** (90 mg, 0.28 mmol) and acetyl chloride (176 mg, 160 µL, 2.24 mmol) as a white solid (75 mg, 93 %) after precipitation from acetonitrile.

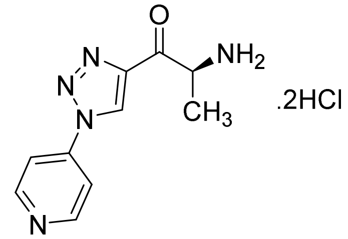

m.p.: >183 °C decomp. ^{1}H NMR (400 MHz, DMSO-d_6) δ: 1.60 (d, 3H, J = 7.2 Hz), 4.86–4.97 (m br, 1H), 8.47 (d, 2H, J = 6.8 Hz), 8.80 (d br, 3H, J = 5.4 Hz), 9.07 (d, 2H, J = 6.5 Hz), 10.23 (s, 1H), 11.65 (s br, 1H). ^{13}C NMR (101 MHz, DMSO-d_6) δ: 16.4, 52.1, 115.9, 128.6, 144.5, 145.2, 147.7, 189.3. HRMS (ESI): *m/z* [M+H]$^+$ calculated for $C_{10}H_{11}N_5O$: 218.10364, found: 218.10361. Purity (HPLC/UV): 98.6 %.

(*S*)-2-amino-1-[1-(4-fluorophenyl)-1*H*-1,2,3-triazol-4-yl]propan-1-on hydrochloride (26n)

Prepared according to **GP7** from Boc-derivative **27n** (220 mg, 0.66 mmol) and acetyl chloride (415 mg, 377 µL, 5.28 mmol) as a white solid (170 mg, 95 %) after precipitation from acetonitrile.

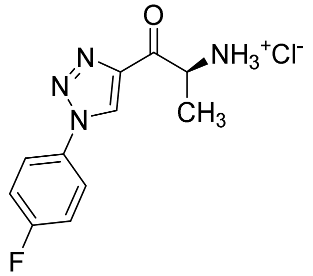

m.p.: >183 °C decomp. ^{1}H NMR (400 MHz, DMSO-d_6) δ: 1.59 (d, 3H, J = 7.2 Hz), 4.91 (q, 1H, J = 7.2 Hz), 7.46–7.57 (m, 2H), 8.01–8.11 (m, 2H), 8.70 (s br, 3H), 9.77 (s, 1H). ^{13}C NMR (101 MHz, DMSO-d_6) δ: 16.5, 52.0, 116.9 (d, J = 23.5 Hz), 123.3 (d, J = 8.9 Hz), 127.6, 132.4 (d, J = 2.8 Hz), 144.1, 162.2 (d, J = 247.0 Hz), 189.5. ^{19}F NMR (282 MHz, DMSO-d_6) δ: -111.2 (tt, 1F, J_1 = 8.9 Hz, J_2 = 4.8 Hz). HRMS (ESI): *m/z* [M+H]$^+$ calculated for $C_{11}H_{11}FN_4O$: 235.09897, found: 235.09888. Purity (HPLC/UV): 98.4 %.

(*S*)-2-amino-1-[1-(4-(trifluoromethyl)phenyl)-1*H*-1,2,3-triazol-4-yl]propan-1-on hydrochloride (26o)

Prepared according to **GP7** from Boc-derivative **27o** (173 mg, 0.45 mmol) and acetyl chloride (283 mg, 256 µL, 3.60 mmol) as a pale orange solid (102 mg, 71 %) after precipitation from acetonitrile.

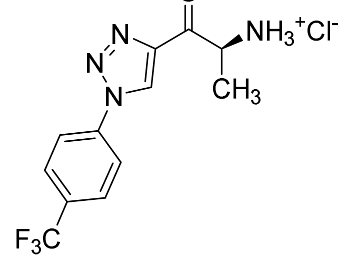

m.p.: >196 °C decomp. ^{1}H NMR (400 MHz, DMSO-d_6) δ: 1.59 (d, 3H, J = 7.2 Hz), 4.90–4.98 (m br, 1H), 8.06 (d, 2H, J = 8.5 Hz), 8.28 (d, 2H, J = 8.4 Hz), 8.59 (s br, 3H), 9.92 (s, 1H). ^{13}C NMR (101 MHz, DMSO-d_6) δ: 16.5,

52.0, 121.4, 123.7 (q, *J* = 272.3 Hz), 127.3 (q, *J* = 3.8 Hz), 127.9, 129.6 (q, *J* = 32.4 Hz), 138.7 (d, *J* = 1.7 Hz), 144.3, 189.4. ^{19}F NMR (282 MHz, DMSO-d_6) δ: -60.6 (s, 3F). HRMS (ESI): *m/z* $[M+H]^+$ calculated for $C_{12}H_{11}F_3N_4O$: 285.09577, found: 285.09567. Purity (HPLC/UV): 98.6 %.

(*S*)-2-amino-1-[1-(3-nitrophenyl)-1*H*-1,2,3-triazol-4-yl]propan-1-on hydrochloride (26p)

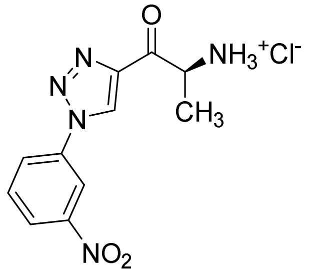

Prepared according to **GP7** from Boc-derivative **27p** (114 mg, 0.32 mmol) and acetyl chloride (201 mg, 180 µL, 2.56 mmol) as a white solid (65 mg, 70 %) after precipitation from *i*-PrOH.

m.p.: >183 °C decomp. ^{1}H NMR (400 MHz, DMSO-d_6) δ: 1.60 (d, 3H, *J* = 7.2 Hz), 4.84–4.99 (m br, 1H), 7.96 (t, 1H, *J* = 8.2 Hz), 8.41 (dd, 1H, J_1 = 8.2 Hz, J_2 = 2.2 Hz), 8.51 (dd, 1H, J_1 = 8.1 Hz, J_2 = 2.2 Hz), 8.70 (s br, 3H), 8.85 (t, 1H, *J* = 2.2 Hz), 10.02 (s, 1H). ^{13}C NMR (101 MHz, DMSO-d_6) δ: 16.5, 52.1, 115.7, 124.1, 126.9, 128.2, 131.7, 136.5, 144.2, 148.5, 189.6. HRMS (ESI): *m/z* $[M+H]^+$ calculated for $C_{11}H_{11}N_5O_3$: 262.09347, found: 262.09459. Purity (HPLC/UV): 99.7 %.

(*S*)-2-amino-1-[1-(4-nitrophenyl)-1*H*-1,2,3-triazol-4-yl]propan-1-on hydrochloride (26q)

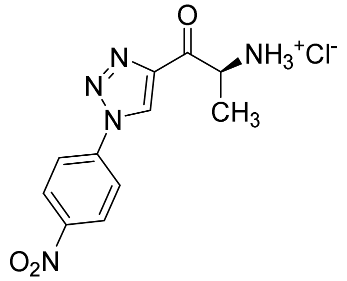

Prepared according to **GP7** from Boc-derivative **27q** (112 mg, 0.31 mmol) and acetyl chloride (195 mg, 177 µL, 2.48 mmol) as a white solid (69 mg, 75 %) after precipitation from *i*-PrOH.

m.p.: >191 °C decomp. ^{1}H NMR (400 MHz, DMSO-d_6) δ: 1.60 (d, 3H, *J* = 7.2 Hz), 4.93 (q, 1H, *J* = 7.2 Hz), 8.34 (d, 2H, *J* = 9.0 Hz), 8.50 (d, 2H, *J* = 9.0 Hz), 8.68 (s br, 3H), 10.01 (s, 1H). ^{13}C NMR (101 MHz, DMSO-d_6) δ: 16.5, 52,1, 121.6, 125.6, 128.2, 140.2, 144.3, 147.4, 189.4. HRMS (ESI): *m/z* $[M+H]^+$ calculated for $C_{11}H_{11}N_5O_3$: 262.09347, found: 262.09419. Purity (HPLC/UV): 98.8 %.

(*S*)-2-amino-1-[1-(4-chlorophenyl)-1*H*-1,2,3-triazol-4-yl]propan-1-on hydrochloride (26r)

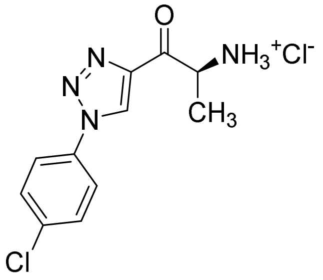

Prepared according to **GP7** from Boc-derivative **27r** (100 mg, 0.29 mmol) and acetyl chloride (182 mg, 165 µl, 2.32 mmol) as a pale pink solid (65 mg, 79 %) after precipitation from acetonitrile.

m.p.: >177 °C decomp. ^{1}H NMR (400 MHz, DMSO-d_6) δ: 1.59 (d, 3H, *J* = 7.2 Hz), 4.86 – 4.99 (m br, 1H), 7.71 – 7.77 (m, 2H), 8.02 – 8.08 (m, 2H), 8.60 (s br, 3H), 9.80 (s, 1H). ^{13}C NMR (101 MHz, DMSO-d_6) δ: 16.5, 52.0, 122.5, 127.6, 130.0, 134.0, 134.7, 144.2, 189.4. HRMS (ESI): *m/z* $[M+H]^+$ calculated for $C_{11}H_{11}ClN_4O$: 251.06942, found: 251.06943. Purity (HPLC/UV): 98.9 %.

(*S*)-2-amino-1-[1-(3-chlorophenyl)-1*H*-1,2,3-triazol-4-yl]propan-1-on hydrochloride (26s)

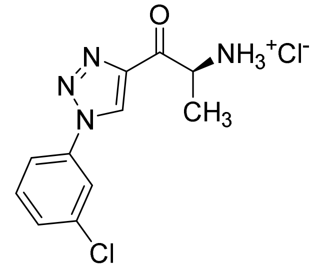

Prepared according to **GP7** from Boc-derivative **27s** (119 mg, 0.34 mmol) and acetyl chloride (214 mg, 194 µl, 2.72 mmol) as an off-white solid (66 mg, 67 %) after precipitation from *i*-PrOH.

m.p.: >171 °C decomp. ^{1}H NMR (400 MHz, DMSO-d_6) δ: 1.59 (d, 3H, J = 7.2 Hz), 4.91 (q, 1H, J = 7.2 Hz), 7.61 – 7.73 (m, 2H), 7.99 – 8.06 (m, 1H), 8.17 (t br, 1H, J = 2.0 Hz), 8.66 (s br, 3H), 9.84 (s, 1H). ^{13}C NMR (101 MHz, DMSO-d_6) δ: 16.5, 52.0, 119.4, 120.7, 127.8, 129.5, 131.7, 134.3, 136.9, 144.1, 189.4. HRMS (ESI): *m/z* [M+H]$^+$ calculated for $C_{11}H_{11}ClN_4O$: 251.06942, found: 251.07006. Purity (HPLC/UV): 99.1 %.

(*S*)-2-amino-1-[1-(2-chlorophenyl)-1*H*-1,2,3-triazol-4-yl]propan-1-on hydrochloride (26t)

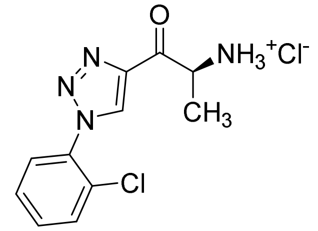

Prepared according to **GP7** from Boc-derivative **27t** (101 mg, 0.29 mmol) and acetyl chloride (182 mg, 165 µl, 2.32 mmol) as an off-white solid (60 mg, 72 %) after precipitation from acetonitrile.

m.p.: 138 – 169 °C (decomp.). ^{1}H NMR (400 MHz, DMSO-d_6) δ: 1.60 (d, 3H, J = 7.2 Hz), 4.95 (q, 1H, J = 7.2 Hz), 7.64 (td, 1H, J_1 = 7.6 Hz, J_2 = 2.2 Hz), 7.71 (td, 1H, J_1 = 7.8 Hz, J_2 = 1.8 Hz), 7.81 (dd, 1H, J_1 = 7.7 Hz, J_2 = 1.7 Hz), 7.84 (dd, 1H, J_1 = 8.0 Hz, J_2 = 1.4 Hz), 8.53 (s br, 3H), 9.55 (s, 1H). ^{13}C NMR (101 MHz, DMSO-d_6) δ: 16.5, 52.0, 128.6, 128.6, 128.8, 130.6, 131.4, 132.5, 133.6, 143.4, 189.4. HRMS (ESI): *m/z* [M+H]$^+$ calculated for $C_{11}H_{11}ClN_4O$: 251.06942, found: 251.07022. Purity (HPLC/UV): 99.4 %.

(*S*)-2-amino-1-[1-(3,4-dichlorophenyl)-1*H*-1,2,3-triazol-4-yl]propan-1-on hydrochloride (26u)

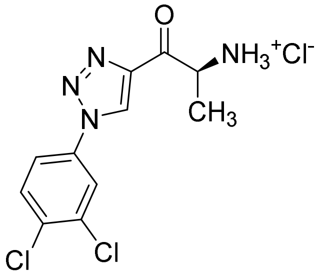

Prepared according to **GP7** from Boc-derivative **27u** (296 mg, 0.77 mmol) and acetyl chloride (484 mg, 440 µl, 6.16 mmol) as an off-white solid (188 mg, 76 %) after recrystallization from acetonitrile.

m.p.: >163 °C decomp. ^{1}H NMR (400 MHz, DMSO-d_6) δ: 1.58 (d, 3H, J = 7.2 Hz), 4.92 (q, 1H, J = 7.2 Hz), 7.95 (d, 1H, J = 8.8 Hz), 8.07 (dd, 1H, J_1 = 8.8 Hz, J_2 = 2.6 Hz), 8.39 (d, 1H, J = 2.5 Hz), 8.58 (s br, 3H), 9.85 (s, 1H). ^{13}C NMR (101 MHz, DMSO-d_6) δ: 16.5, 52.0, 120.9, 122.6, 128.0, 132.0, 132.1, 132.5, 135.4, 144.1, 189.4. HRMS (ESI): *m/z* [M+H]$^+$ calculated for $C_{11}H_{10}Cl_2N_4O$: 285.03044, found: 285.03009. Purity (HPLC/UV): 99.3 %.

(*S*)-2-amino-1-[1-(3-chloro-4-fluorophenyl)-1*H*-1,2,3-triazol-4-yl]propan-1-on hydrochloride (26v)

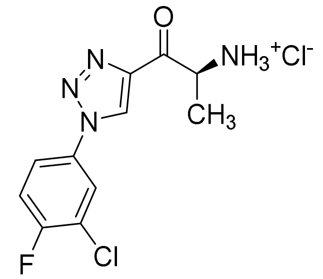

Prepared according to **GP7** from Boc-derivative **27v** (185 mg, 0.50 mmol) and acetyl chloride (314 mg, 285 µl, 4.00 mmol) as a white solid (120 mg, 79 %) after precipitation from *i*-PrOH.

m.p.: >185 °C decomp. ^{1}H NMR (400 MHz, DMSO-d_6) δ: 1.59 (d, 3H, J = 7.2 Hz), 4.90 (q, 1H, J = 7.2 Hz), 7.74 (t, 1H, J = 9.0 Hz), 8.07 (ddd, 1H, J_1 = 9.0 Hz, J_2 = 4.1 Hz, J_3 = 2.7 Hz), 8.34 (dd, 1H,

J_1 = 6.4 Hz, J_2 = 2.7 Hz), 8.70 (s br, 3H), 9.82 (s, 1H). ^{13}C NMR (101 MHz, DMSO-d_6) δ: 16.5, 52.0, 118.3 (d, J = 22.9 Hz), 120.9 (d, J = 19.3 Hz), 121.8 (d, J = 8.2 Hz), 123.3, 127.9, 132.9 (d, J = 3.5 Hz), 144.1, 157.4 (d, J = 249.5 Hz), 189.4. ^{19}F NMR (282 MHz, DMSO-d_6) δ: -114.3 (ddd, 1F, J_1 = 8.8 Hz, J_2 = 6.4 Hz, J_3 = 4.2 Hz). HRMS (ESI): *m/z* [M+H]$^+$ calculated for $C_{11}H_{10}ClFN_4O$: 269.05999, found: 269.06059. Purity (HPLC/MS): 99.1 %.

(*S*)-2-amino-1-[1-(2-chloro-4-fluorophenyl)-1*H*-1,2,3-triazol-4-yl]propan-1-on hydrochloride (32a)

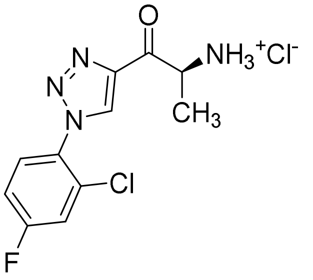

Prepared according to **GP7** from Boc-derivative **35a** (120 mg, 0.33 mmol) and acetyl chloride (204 mg, 185 µl, 2.64 mmol) as a white solid (65 mg, 66 %) after precipitation from acetonitrile.

m.p.: >158 °C decomp. ^{1}H NMR (400 MHz, DMSO-d_6) δ: 1.60 (d, 3H, J = 7.2 Hz), 4.93 (q, 1H, J = 7.2 Hz), 7.56 (td, 1H, J_1 = 8.4 Hz, J_2 = 2.8 Hz), 7.87 – 7.95 (m, 2H), 8.63 (s br, 3H), 9.54 (s, 1H). ^{13}C NMR (101 MHz, DMSO-d_6) δ: 16.5, 52.0, 115.9 (d, J = 22.8 Hz), 118.1 (d, J = 27.6 Hz), 130.1 – 130.8 (m, 3C), 131.6, 143.4, 162.7 (d, J = 252.3 Hz), 189.5. ^{19}F NMR (282 MHz, DMSO-d_6) δ: -106.5 (m, 1F). HRMS (ESI): *m/z* [M+H]$^+$ calculated for $C_{11}H_{10}ClFN_4O$: 269.05999, found: 269.06094. Purity (HPLC/UV): >99.9 %.

(*S*)-2-amino-1-[1-(2-chloro-4-fluorophenyl)-1*H*-1,2,3-triazol-4-yl]butan-1-on hydrochloride (32b)

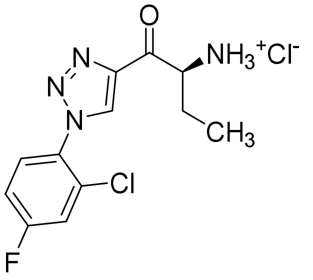

Prepared according to **GP7** from Boc-derivative **35b** (124 mg, 0.32 mmol) and acetyl chloride (203 mg, 184 µl, 2.56 mmol) as a white solid (67 mg, 65 %) after recrystallization from acetonitrile.

m.p.: >196 °C decomp. ^{1}H NMR (400 MHz, DMSO-d_6) δ: 0.95 (t, 3H, J = 7.5 Hz), 1.97 – 2.17 (m, 2H), 4.88 – 4.95 (m, 1H), 7.56 (td, 1H, J_1 = 8.4 Hz, J_2 = 2.8 Hz), 7.87 – 7.96 (m, 2H), 8.73 (s br, 3H), 9.55 (s, 1H). ^{13}C NMR (101 MHz, DMSO-d_6) δ: 9.1, 23.8, 56.6, 115.9 (d, J = 22.9 Hz), 118.1 (d, J = 26.9 Hz), 130.0 – 130.9 (m, 3C), 131.6, 143.9, 162.7 (d, J = 252.3 Hz), 189.2. ^{19}F NMR (282 MHz, DMSO-d_6) δ: -106.5 (m, 1F). HRMS (ESI): *m/z* [M+H]$^+$ calculated for $C_{12}H_{12}ClFN_4O$: 283.07564, found: 283.07694. Purity (HPLC/UV): 99.8 %.

(*S*)-2-amino-1-[1-(4-fluorophenyl)-1*H*-1,2,3-triazol-4-yl]butan-1-on hydrochloride (32c)

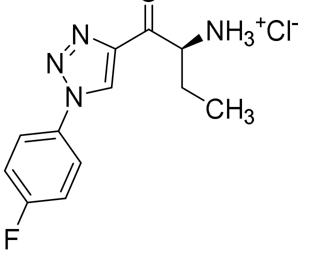

Prepared according to **GP7** from Boc-derivative **35c** (105 mg, 0.30 mmol) and acetyl chloride (188 mg, 171 µl, 2.40 mmol) as a white solid (77 mg, 90 %) after recrystallization from *i*-PrOH.

m.p.: >182 °C decomp. ^{1}H NMR (400 MHz, DMSO-d_6) δ: 0.94 (t, 3H, J = 7.5 Hz), 1.95 – 2.18 (m, 2H), 4.91 (dd, 1H, J_1 = 6.5 Hz, J_2 = 4.7 Hz), 7.51 (t, 2H, J = 8.7 Hz), 8.06 (dd, 2H, J_1 = 8.9 Hz, J_2 = 4.6 Hz), 8.72 (s br, 3H), 9.78 (s, 1H). ^{13}C NMR (101 MHz, DMSO-d_6) δ: 9.0, 23.8, 56.5, 116.9 (d, J = 23.3 Hz), 123.2 (d, J = 8.9 Hz), 127.6, 132.4 (d, J = 2.9 Hz), 144.6, 162.2 (d, J = 247.2 Hz), 189.2. ^{19}F NMR (282 MHz, DMSO-d_6) δ: -111.2 (m, 1F). HRMS (ESI): *m/z* [M+H]$^+$ calculated for $C_{12}H_{13}FN_4O$: 249.11462, found: 249.11581. Purity (HPLC/UV): 98.6 %.

(*S*)-2-amino-1-(5-phenylthiazol-2-yl)propan-1-on hydrochloride (11)

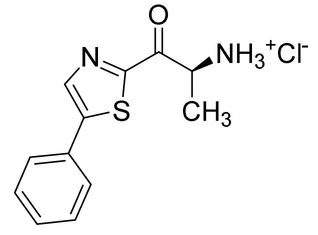

Prepared according to **GP7** from Boc-derivative **16** (150 mg, 0.45 mmol) and acetyl chloride (282 mg, 255 µl, 3.6 mmol) as a white solid (115 mg, 96 %) after recrystallization from EtOH/Et_2O.

m.p.: >168 °C decomp. ^{1}H NMR (400 MHz, DMSO-d_6) δ: 1.61 (d, 3H, J = 7.2 Hz), 4.97 (q, 1H, J = 7.2 Hz), 7.45 – 7.57 (m, 3H), 7.82 – 7.91 (m, 2H), 8.62 – 8.77 (m br, 4H). ^{13}C NMR (101 MHz, DMSO-d_6) δ: 16.6, 51.2, 127.4, 129.5, 129.6, 130.2, 142.0, 147.6, 160.7, 189.1. HRMS (ESI): *m/z* $[M+H]^+$ calculated for $C_{12}H_{12}N_2OS$: 233.07431, found: 233.07413. Purity (HPLC/UV): 99.4 %.

(*S*)-2-amino-3-methyl-1-(5-phenylthiazol-2-yl])butan-1-on hydrochloride (25a)

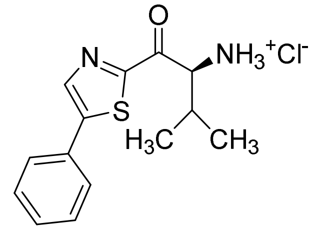

Prepared according to **GP7** from Boc-derivative **24a** (200 mg, 0.55 mmol) and acetyl chloride (345 mg, 314 µl, 4.40 mmol) as a white solid (131 mg, 80 %) after recrystallization from *i*-PrOH.

m.p.: >170 °C decomp. ^{1}H NMR (400 MHz, CD_3OD) δ: 1.00 (d, 3H, J = 7.0 Hz), 1.17 (d, 3H, J = 7.0 Hz), 2.67 (heptd, 1H, J_1 = 7.0 Hz, J_2 = 4.6 Hz), 5.07 (d, 1H, J = 4.7 Hz), 7.42 – 7.54 (m, 3H), 7.74 – 7.82 (m, 2H), 8.45 (s, 1H). ^{13}C NMR (101 MHz, CD_3OD) δ: 17.2, 19.5, 31.3, 61.6, 128.5, 130.7, 131.2, 131.4, 142.6, 150.4, 162.8, 189.6. HRMS (ESI): *m/z* $[M+H]^+$ calculated for $C_{14}H_{16}N_2OS$: 261.10561, found: 261.10582. Purity (HPLC/UV): 99.4 %.

(*S*)-2-amino-3-phenyl-1-(5-phenylthiazol-2-yl])propan-1-on hydrochloride (25b)

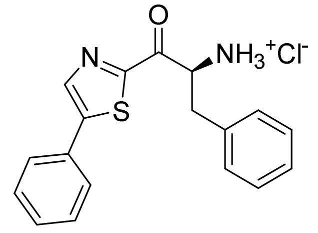

Prepared according to **GP7** from Boc-derivative **24b** (400 mg, 0.98 mmol) and acetyl chloride (615 mg, 559 µl, 7.84 mmol) as a white solid (315 mg, 93 %) after recrystallization from *i*-PrOH.

m.p.: >175 °C decomp. ^{1}H NMR (400 MHz, CD_3OD) δ: 3.26 (dd, 1H, J_1 = 14.4 Hz, J_2 = 8.5 Hz), 3.60 (dd, 1H, J_1 = 14.4 Hz, J_2 = 5.0 Hz), 5.40 (dd, 1H, J_1 = 8.5 Hz, J_2 = 5.0 Hz), 7.22 – 7.40 (m, 5H), 7.43 – 7.57 (m, 3H), 7.74 – 7.83 (m, 2H), 8.44 (s, 1H). ^{13}C NMR (101 MHz, CD_3OD) δ: 38.3, 58.3, 128.5, 129.0, 130.2, 130.5, 130.7, 131.2, 131.4, 135.4, 142.7, 150.4, 162.2, 189.0. HRMS (ESI): *m/z* $[M+H]^+$ calculated for $C_{18}H_{16}N_2OS$: 309.10561, found: 309.10604. Purity (HPLC/UV): 99.2 %.

(*S*)-2-amino-4-phenyl-1-(5-phenylthiazol-2-yl])butan-1-on hydrochloride (25c)

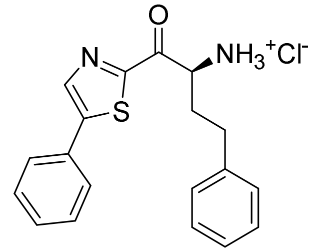

Prepared according to **GP7** from Boc-derivative **24c** (90 mg, 0.21 mmol) and acetyl chloride (132 mg, 120 µl, 1.68 mmol) as a white solid (52 mg, 69 %) after recrystallization from *i*-PrOH.

m.p.: >135 °C decomp. ^{1}H NMR (400 MHz, CD_3OD) δ: 2.32 – 2.45 (m, 1H), 2.46 – 2.57 (m, 1H), 2.69 – 2.90 (m, 2H), 5.17 (dd, 1H, J_1 = 7.3 Hz, J_2 = 4.7 Hz), 7.09 – 7.19 (m, 3H), 7.20 – 7.27 (m, 2H), 7.39 – 7.54 (m, 3H), 7.72 – 7.82 (m, 2H), 8.43 (s, 1H). ^{13}C NMR (101 MHz, CD_3OD) δ: 32.0, 34.0, 56.9, 127.6, 128.5, 129.5, 129.6, 130.7, 131.2, 131.3, 140.9, 142.5, 150.4, 162.3, 189.3. HRMS (ESI): *m/z* $[M+H]^+$ calculated for $C_{19}H_{18}N_2OS$: 323.12126, found: 323.12187. Purity (HPLC/UV): 98.7 %.

(*S*)-2-amino-1-(5-phenylthiazol-2-yl])hexan-1-on hydrochloride (25d)

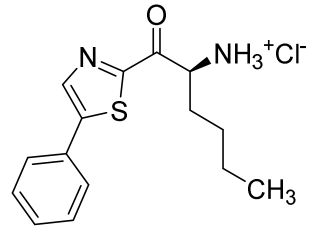

Prepared according to **GP7** from Boc-derivative **24d** (340 mg, 0.91 mmol) and acetyl chloride (571 mg, 519 µl, 7.28 mmol) as a pale yellow solid (202 mg, 71 %) after recrystallization from *i*-PrOH.

m.p.: 124 – 127 °C. ^{1}H NMR (400 MHz, CD_3OD) δ: 0.94 (t, 3H, J = 7.0 Hz), 1.33 – 1.58 (m, 4H), 1.97 – 2.11 (m, 1H), 2.15 – 2.28 (m, 1H), 5.11 (dd, 1H, J_1 = 7.8 Hz, J_2 = 4.6 Hz), 7.41 – 7.56 (m, 3H), 7.73 – 7.83 (m, 2H), 8.45 (s, 1H). ^{13}C NMR (101 MHz, CD_3OD) δ: 14.0, 23.3, 28.1, 32.0, 57.0, 128.5, 130.7, 131.2, 131.4, 142.6, 150.3, 162.3, 189.6. HRMS (ESI): *m/z* $[M+H]^+$ calculated for $C_{15}H_{18}N_2OS$: 275.12126, found: 275.12261. Purity (HPLC/UV): 99.1 %.

(*S*)-2-amino-1-(5-phenylthiazol-2-yl])butan-1-on hydrochloride (25e)

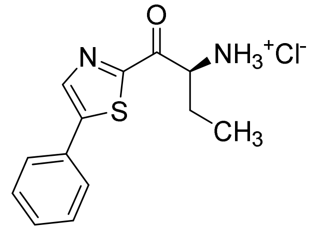

Prepared according to **GP7** from Boc-derivative **24e** (140 mg, 0.40 mmol) and acetyl chloride (251 mg, 228 µl, 3.20 mmol) as a pale yellow solid (101 mg, 89 %) after recrystallization from *i*-PrOH.

m.p.: >180 °C decomp. ^{1}H NMR (400 MHz, CD_3OD) δ: 1.07 (t, 3H, J = 7.5 Hz), 2.05 – 2.20 (m, 1H), 2.20 – 2.34 (m, 1H), 5.09 (dd, 1H, J_1 = 7.2 Hz, J_2 = 4.6 Hz), 7.42 – 7.54 (m, 3H), 7.75 – 7.82 (m, 2H), 8.45 (s, 1H). ^{13}C NMR (101 MHz, CD_3OD) δ: 9.6, 25.6, 58.1, 128.5, 130.7, 131.2, 131.4, 142.6, 150.3, 162.3, 189.5. HRMS (ESI): *m/z* $[M+H]^+$ calculated for $C_{13}H_{14}N_2OS$: 247.08996, found: 247.09110. Purity (HPLC/UV): 99.3 %.

(*S*)-2-amino-2-cyclopropyl-1-(5-phenylthiazol-2-yl])ethan-1-on hydrochloride (25f)

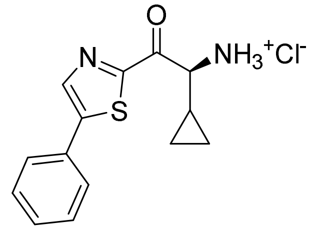

Prepared according to **GP7** from Boc-derivative **24f** (258 mg, 0.70 mmol) and acetyl chloride (440 mg, 440 µl, 5.60 mmol) as a pale yellow solid (152 mg, 72 %) after recrystallization from *i*-PrOH.

m.p.: >165 °C decomp. ^{1}H NMR (400 MHz, CD_3OD) δ: 0.67 – 0.80 (m, 2H), 0.80 – 0.92 (m, 1H), 0.92 – 1.04 (m, 1H), 1.08 – 1.22 (m, 1H), 4.49 (d, 1H, J = 10.1 Hz), 7.40 – 7.56 (m, 3H), 7.72 – 7.83 (m, 2H), 8.44 (s, 1H). ^{13}C NMR (101 MHz, CD_3OD) δ: 5.8, 5.9, 13.5, 60.5, 128.5, 130.7, 131.2, 131.3, 142.6, 150.3, 163.0, 188.8. HRMS (ESI): *m/z* $[M+H]^+$ calculated for $C_{14}H_{14}N_2OS$: 259.08996, found: 259.09079. Purity (HPLC/UV): 99.3 %.

(*S*)-2-amino-1-(benzo[*d*]thiazol-2-yl)propan-1-on hydrochloride (34a)

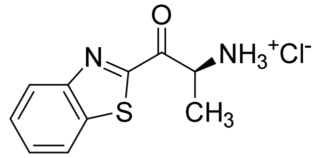

Prepared according to **GP7** from Boc-derivative **39a** (100 mg, 0.33 mmol) and acetyl chloride (204 mg, 185 µl, 2.64 mmol) as a white solid (72 mg, 91 %) after precipitation from *i*-PrOH.

m.p.: >156 °C decomp. ^{1}H NMR (400 MHz, DMSO-d_6) δ: 1.66 (d, 3H, J = 7.3 Hz), 5.11 (q, 1H, J = 7.3 Hz), 7.65 – 7.77 (m, 2H), 8.25 – 8.37 (m, 2H), 8.82 (s br, 3H). ^{13}C NMR (101 MHz, DMSO-d_6) δ: 16.4, 51.5, 123.4, 125.4, 127.9, 128.7, 136.5, 152.8, 162.8, 190.9. HRMS (ESI): *m/z* $[M+H]^+$ calculated for $C_{10}H_{10}N_2OS$: 207.05866, found: 207.05851. Purity (HPLC/UV): 99.1 %.

(*S*)-2-amino-1-(6-fluorobenzo[*d*]thiazol-2-yl)propan-1-on hydrochloride (34b)

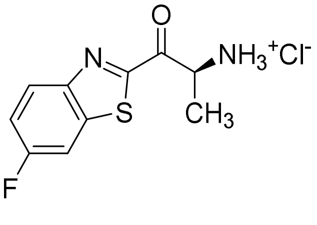

Prepared according to **GP7** from Boc-derivative **39b** (110 mg, 0.34 mmol) and acetyl chloride (214 mg, 195 µl, 2.72 mmol) as a pale yellow solid (76 mg, 86 %) after precipitation from *i*-PrOH.

m.p.: >152 °C decomp. ^{1}H NMR (400 MHz, DMSO-d_6) δ: 1.65 (d, 3H, J = 7.2 Hz), 5.08 (q, 1H, J = 7.2 Hz), 7.61 (td, 1H, J_1 = 9.1 Hz, J_2 = 2.7 Hz), 8.24 (dd, 1H, J_1 = 8.7 Hz, J_2 = 2.7 Hz), 8.36 (dd, 1H, J_1 = 9.1 Hz, J_2 = 4.9 Hz), 8.80 (s br, 3H). ^{13}C NMR (101 MHz, DMSO-d_6) δ: 16.4, 51.5, 109.6 (d, J = 27.7 Hz), 117.2 (d, J = 25.5 Hz), 127.3 (d, J = 10.1 Hz), 138.0 (d, J = 12.6 Hz), 149.7 (d, J = 1.3 Hz), 161.6 (d, J = 248.2 Hz), 163.0 (d, J = 3.6 Hz), 190.6. ^{19}F NMR (282 MHz, DMSO-d_6) δ: -109.9 (td, 1F, J_1 = 8.9 Hz, J_2 = 5.0 Hz). HRMS (ESI): *m/z* [M+H]$^+$ calculated for $C_{10}H_9FN_2OS$: 225.04924, found: 225.04908. Purity (HPLC/UV): 97.8 %.

(*S*)-2-amino-1-(benzo[*d*]thiazol-2-yl)butan-1-on hydrochloride (34c)

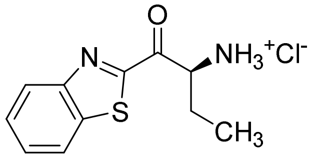

Prepared according to **GP7** from Boc-derivative **39c** (100 mg, 0.31 mmol) and acetyl chloride (196 mg, 178 µl, 2.48 mmol) as a white solid (70 mg, 87 %) after precipitation from *i*-PrOH.

m.p.: >140 °C decomp. ^{1}H NMR (400 MHz, DMSO-d_6) δ: 0.96 (t, 3H, J = 7.5 Hz), 2.01 – 2.25 (m, 2H), 5.07 – 5.15 (m, 1H), 7.66 – 7.76 (m, 2H), 8.26 – 8.38 (m, 2H), 8.83 (s br, 3H). ^{13}C NMR (101 MHz, DMSO-d_6) δ: 9.3, 23.8, 56.0, 123.5, 125.5, 128.0, 128.8, 136.6, 152.8, 163.1, 190.7. HRMS (ESI): *m/z* [M+H]$^+$ calculated for $C_{11}H_{12}N_2OS$: 221.07431, found: 221.07428. Purity (HPLC/UV): 96.6 %.

(*S*)-2-amino-1-(6-fluorobenzo[*d*]thiazol-2-yl)butan-1-on hydrochloride (34d)

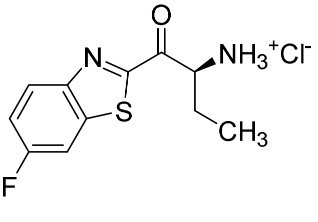

Prepared according to **GP7** from Boc-derivative **39d** (90 mg, 0.27 mmol) and acetyl chloride (170 mg, 154 µL, 2.16 mmol) as a pale yellow solid (58 mg, 80 %) after recrystallization from *i*-PrOH.

m.p.: >145 °C decomp. ^{1}H NMR (400 MHz, DMSO-d_6) δ: 0.96 (d, 3H, J = 7.5 Hz), 2.01–2.22 (m, 2H), 5.04–5.11 (s br, 1H), 7.61 (td, 1H, J_1 = 9.1 Hz, J_2 = 2.7 Hz), 8.25 (dd, 1H, J_1 = 8.8 Hz, J_2 = 2.7 Hz), 8.36 (dd, 1H, J_1 = 9.1 Hz, J_2 = 4.9 Hz), 8.82 (s br, 3H). ^{13}C NMR (101 MHz, DMSO-d_6) δ: 9.3, 23.8, 56.0, 109.6 (d, J = 27.7 Hz), 117.2 (d, J = 25.5 Hz), 127.3 (d, J = 10.1 Hz), 138.1 (d, J = 12.3 Hz), 149.8, 161.6 (d, J = 248.0 Hz), 163.3 (d, J = 3.6 Hz), 190.4. ^{19}F NMR (282 MHz, DMSO-d_6) δ: -109.8 (m, 1F). HRMS (ESI): *m/z* [M+H]$^+$ calculated for $C_{11}H_{11}FN_2OS$: 239.06489, found: 239.06494. Purity (HPLC/UV): 97.6 %.

(*S*)-2-amino-1-[5-(4-fluorophenyl)thiazol-2-yl)]propan-1-on (49a)

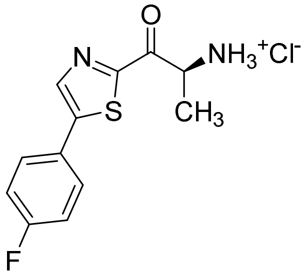

Prepared according to **GP7** from Boc-derivative **45a** (152 mg, 0.43 mmol) and acetyl chloride (270 mg, 245 µL, 3.44 mmol) as a pale yellow solid (82 mg, 67 %) after recrystallization from *i*-PrOH.

m.p.: >165 °C decomp. ^{1}H NMR (400 MHz, DMSO-d_6) δ: 1.60 (d, 3H, J = 7.2 Hz), 4.98 (q, 1H, J = 7.2 Hz), 7.34–7.44 (m, 2H), 7.90–7.99 (m, 2H), 8.60 (s br, 3H), 8.67 (s, 1H). ^{13}C NMR (101 MHz, DMSO-d_6) δ: 16.6, 51.2, 116.7 (d, J = 22.1 Hz), 126.2 (d, J = 3.2 Hz), 129.8 (d,

J = 8.6 Hz), 142.1, 146.5, 160.8 (d, J = 0.8 Hz), 163.1 (d, J = 248.8 Hz), 189.1. ^{19}F NMR (282 MHz, DMSO-d_6) δ: -110.6 (m, 1F). HRMS (ESI): *m/z* $[M+H]^+$ calculated for $C_{12}H_{11}FN_2OS$: 251.06489, found: 251.06504. Purity (HPLC/UV): 98.4 %.

(*S*)-2-amino-1-[5-(4-fluorophenyl)thiazol-2-yl)]butan-1-on (49b)

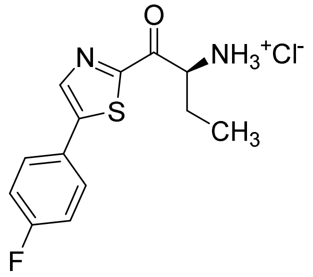

Prepared according to **GP7** from Boc-derivative **45b** (100 mg, 0.30 mmol) and acetyl chloride (188 mg, 171 µL, 2.40 mmol) as a pale yellow solid (62 mg, 76 %) after recrystallization from *i*-PrOH.

m.p.: >150 °C decomp. ^{1}H NMR (400 MHz, CD_3OD) δ: 1.08 (t, 3H, J = 7.5 Hz), 2.13 (dp, 1H, J_1 = 14.9 Hz, J_2 = 7.4 Hz), 2.20–2.35 (m, 1H), 5.09 (dd, 1H, J_1 = 7.2 Hz, J_2 = 4.7 Hz), 7.16–7.31 (m, 2H), 7.79–7.90 (m, 2H), 8.42 (s, 1H). ^{13}C NMR (101 MHz, DMSO-d_6) δ: 18.7, 33.4, 65.2, 126.1 (d, J = 22.1 Hz), 135.6 (d, J = 3.4 Hz), 139.3 (d, J = 8.6 Hz), 151.6, 156.1, 161.2 (d, J = 0.7 Hz), 163.1 (d, J = 248.6 Hz), 198.3. ^{19}F NMR (282 MHz, DMSO-d_6) δ: -109.9 (m, 1F). HRMS (ESI): *m/z* $[M+H]^+$ calculated for $C_{13}H_{13}FN_2OS$: 265.08054, found: 265.08071. Purity (HPLC/UV): 99.0 %.

(*S*)-2-amino-1-[5-(2-chloro-4-fluorophenyl)thiazol-2-yl]propan-1-on hydrochloride (49c)

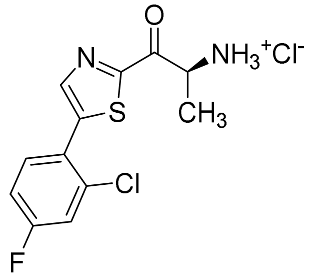

Prepared according to **GP7** from Boc-derivative **46a** (88 mg, 0.23 mmol) and acetyl chloride (144 mg, 130 µL, 1.84 mmol) as a white solid (65 mg, 88 %) after recrystallization from acetonitrile.

m.p.: 127 - 134 °C decomp. ^{1}H NMR (400 MHz, DMSO-d_6) δ: 1.62 (d, 3H, J = 7.2 Hz), 5.00 (q, 1H, J = 7.2 Hz), 7.45 (td, 1H, J_1 = 8.4 Hz, J_2 = 2.7 Hz), 7.75 (dd, 1H, J_1 = 8.8 Hz, J_2 = 2.6 Hz), 7.94 (dd, 1H, J_1 = 8.8 Hz, J_2 = 6.0 Hz), 8.56 (s, 1H), 8.65 (s br, 3H). ^{13}C NMR (101 MHz, DMSO-d_6) δ: 16.5, 51.3, 115.6 (d, J = 21.5 Hz), 118.0 (d, J = 25.6 Hz), 124.9 (d, J = 3.6 Hz), 132.8 (d, J = 11.0 Hz), 133.6 (d, J = 9.4 Hz), 141.6, 145.6, 162.4 (d, J = 252.0 Hz), 162.5, 189.3. ^{19}F NMR (282 MHz, DMSO-d_6) δ: -108.2 (m, 1F). HRMS (ESI): *m/z* $[M+H]^+$ calculated for $C_{12}H_{10}ClFN_2OS$: 285.02592, found: 285.02580. Purity (HPLC/UV): 98.8 %.

(*S*)-2-amino-1-[5-(2-chloro-4-fluorophenyl)thiazol-2-yl]butan-1-on hydrochloride (49d)

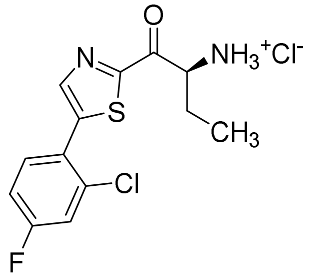

Prepared according to **GP7** from Boc-derivative **46b** (100 mg, 0.25 mmol) and acetyl chloride (157 mg, 143 µL, 2.00 mmol) as a pale orange solid (71 mg, 85 %) after recrystallization from acetonitrile.

m.p.: >134 °C decomp. ^{1}H NMR (400 MHz, DMSO-d_6) δ: 0.96 (t, 3H, J = 7.5 Hz), 1.96–2.21 (m, 2H), 4.98 (dd, 1H, J_1 = 6.7 Hz, J_2 = 4.9 Hz), 7.44 (td, 1H, J_1 = 8.4 Hz, J_2 = 2.7 Hz), 7.74 (dd, 1H, J_1 = 8.8 Hz, J_2 = 2.6 Hz), 7.94 (dd, 1H, J_1 = 8.8 Hz, J_2 = 6.0 Hz), 8.56 (s, 1H), 8.78 (s br, 3H). ^{13}C NMR (101 MHz, DMSO-d_6) δ: 9.3, 23.9, 55.8, 115.6 (d, J = 21.6 Hz), 118.1 (d, J = 25.7 Hz), 124.9 (d, J = 3.7 Hz), 132.8 (d, J = 10.9 Hz), 133.6 (d, J = 9.4 Hz), 141.7, 145.6, 162.5 (d, J = 251.8 Hz), 162.8, 189.1. ^{19}F NMR (282 MHz, DMSO-d_6) δ: -108.2 (td, 1F, J_1 = 8.4 Hz, J_2 = 5.9 Hz). HRMS (ESI): *m/z* $[M+H]^+$ calculated for $C_{13}H_{12}ClFN_2OS$: 299.04157, found: 299.04155. Purity (HPLC/UV): 97.9 %.

2. References

1. García-Urricelqui, A.; Cózar, A. de; Mielgo, A.; Palomo, C. Probing α-Amino Aldehydes as Weakly Acidic Pronucleophiles: Direct Access to Quaternary α-Amino Aldehydes by an Enantioselective Michael Addition Catalyzed by Brønsted Bases. *Chem. – A Eur. J.* **2021**, *27* (7), 2483–2492, doi:10.1002/CHEM.202004468.

2. Dai, C.; Stephenson, C. R. J. Total Synthesis of Syringolin A. *Org. Lett.* **2010**, *12* (15), 3453–3455, doi:10.1021/OL101252Y.

3. Du, X.; Zhang, H. Y.; Lei, M.; Li, Z. Y.; Zhu, Y. Q. An Efficient Preparation of Novel Epoxyketone Intermediates for the Synthesis of Carfilzomib and Its Derivatives. *J. Chem. Res.* **2016**, *40* (2), 82–86, doi:10.3184/174751916X14539789662063.

4. Ripa, L.; Edman, K.; Dearman, M.; Edenro, G.; Hendrickx, R.; Ullah, V.; Chang, H.-F.; Lepistö, M.; Chapman, D.; Geschwindner, S.; Wissler, L.; Svanberg, P.; Lawitz, K.; Malmberg, J.; Nikitidis, A.; Olsson, R. I.; Bird, J.; Llinas, A.; Hegelund-Myrbäck, T.; Berger, M.; Thorne, P.; Harrison, R.; Köhler, C.; Drmota, T. Discovery of a Novel Oral Glucocorticoid Receptor Modulator (AZD9567) with Improved Side Effect Profile. *J. Med. Chem.* **2018**, *61* (5), 1785–1799, doi:10.1021/acs.jmedchem.7B01690.

5. Audin, P.; Pothion, C.; Fehrentz, J.-A.; Loffet, A.; Martinez, J.; Paris, J. Diastereoselective Synthesis of N-Protected β-Amino-α-Hydroxyacids (Norstatines) from Urethane N-Carboxyanhydrides (UNCAs). *J. Chem. Res. Synopses* **1999**, No. 4, 282–283, doi:10.1039/a809421g.

6. Gouault, N.; Roch, M. Le; Cornée, C.; David, M.; Uriac, P. Synthesis of Substituted Pyrrolin-4-Ones from Amino Acids in Mild Conditions via a Gold-Catalyzed Approach. *J. Org. Chem.* **2009**, *74* (15), 5614–5617, doi:10.1021/jo900693a.

7. Pirc, S.; Bevk, D.; Golobič, A.; Stanovnik, B.; Svete, J. Transformation of Amino Acids into Nonracemic 1-(Heteroaryl)Ethanamines by the Enamino Ketone Methodology. *Helv. Chim. Acta* **2006**, *89* (1), 30–44, doi:10.1002/hlca.200690010.

8. Jeong, S.; Kim, E.; Kim, M.; Hwang, Y. J.; Padhi, B.; Choi, J.; Lee, Y.; Joo, J. M. Divergent Strategies for the π-Extension of Heteroaryl Halides Using Norbornadiene as an Acetylene Synthon. *Org. Lett.* **2020**, *22* (24), 9670–9676, doi:10.1021/acs.orglett.0c03732.

9. Steert, K.; Berg, M.; Mottram, J. C.; Westrop, G. D.; Coombs, G. H.; Cos, P.; Maes, L.; Joossens, J.; Van der Veken, P.; Haemers, A.; Augustyns, K. α-Ketoheterocycles as Inhibitors of Leishmania Mexicana Cysteine Protease CPB. *ChemMedChem* **2010**, *5* (10), 1734–1748, doi:10.1002/cmdc.201000265.

10. Huang, T.; Wu, X.; Yu, Y.; An, L.; Yin, X. A Convenient Synthesis of 2-Acyl Benzothiazoles/Thiazoles from Benzothiazole/Thiazole and *N,N'*-Carbonyldiimidazole Activated Carboxylic Acids. *Tetrahedron Lett.* **2019**, *60* (25), 1667–1670, doi: 10.1016/j.tetlet.2019.05.043.
