# Supplementary material for: Heterocyclic Cathinones as Inhibitors of Kynurenine Aminotransferase II—Design, Synthesis, and Evaluation
Source: Pharmaceuticals (Basel). 2021 Dec 10;14(12):1291. doi: 10.3390/ph14121291 (PMC8708382; doi:10.3390/ph14121291)

Supplementary material 2

for

Heterocyclic cathinones as inhibitors of kynurenine aminotransferase II – design, synthesis, and evaluation

Michal Maryška [1,2], Lucie Svobodová [1], Wim Dehaen [3], Martina Hrabinová [4,5], Michaela Rumlová [6], Ondřej Soukup [4,5] and Martin Kuchař [1,2]*

[1] Forensic Laboratory of Biologically Active Substances, Department of Chemistry of Natural Compounds, Faculty of Food and Biochemical Technology, UCT Prague, Technická 5, 16628 Prague, Czech Republic; maryskam@vscht.cz

[2] National Institute of Mental Health, Topolová 748, 250 67 Klecany, Czech Republic

[3] CZ-OPENSCREEN: National Infrastructure for Chemical Biology, Department of Informatics and Chemistry, Faculty of Chemical Technology, UCT Prague, Technická 5, 16628 Prague, Czech Republic

[4] Biomedical Research Centre, University Hospital Hradec Králové, Sokolská 581, 50005 Hradec Králové, Czech Republic

[5] Department of Toxicology and Military Pharmacy, University of Defence, Třebešská 1575, 50005 Hradec Králové, Czech Republic

[6] Department of Biotechnology, Faculty of Food and Biochemical Technology, UCT Prague, Technická 5, 16628 Prague, Czech Republic

* Correspondence: martin.kuchar@vscht.cz; Tel.: +420 220 444 431

Table of contents:

1. NMR spectra of new compounds 2

1. NMR spectra of new compounds

Compound **18c** (^{1}H NMR, 400 MHz):

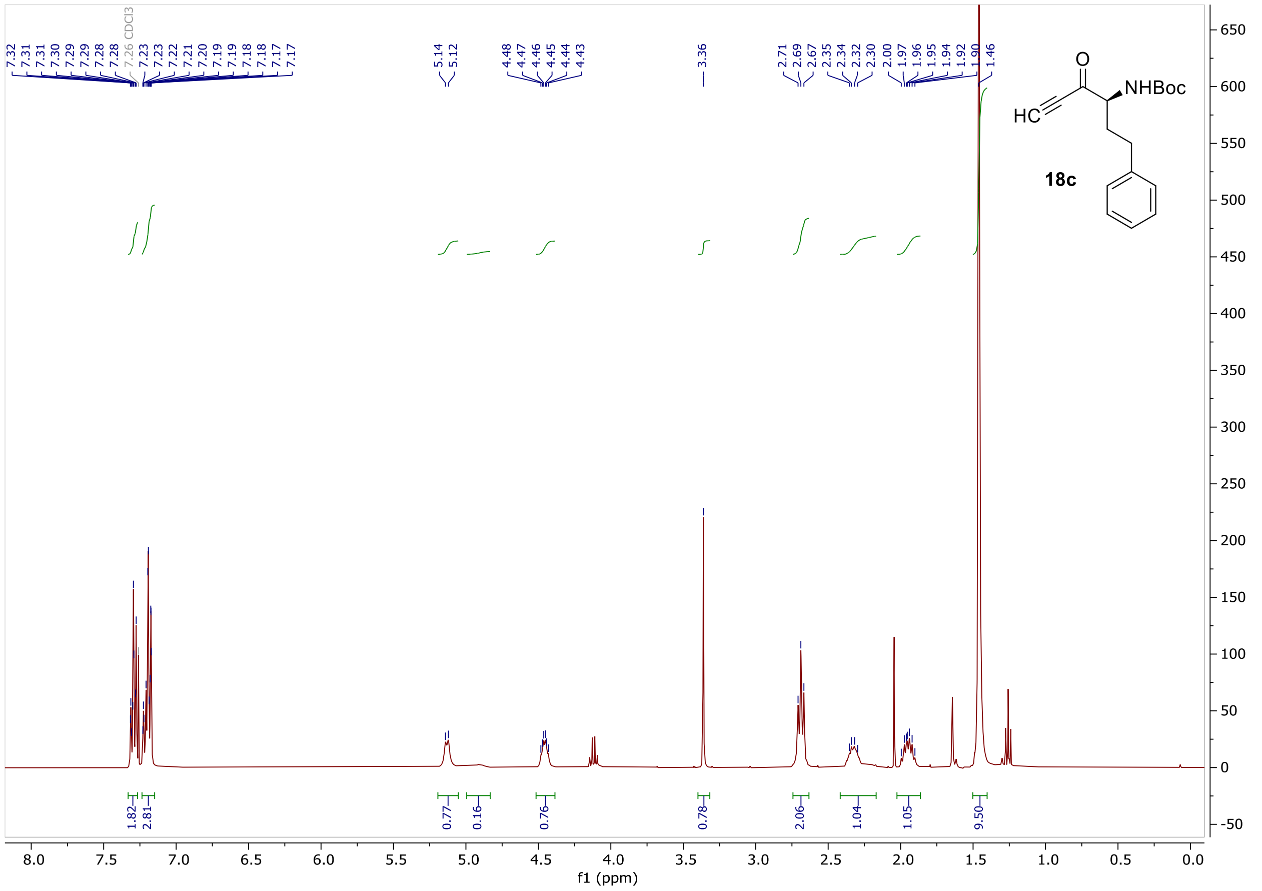

Compound **18c** (^{13}C NMR, 101 MHz):

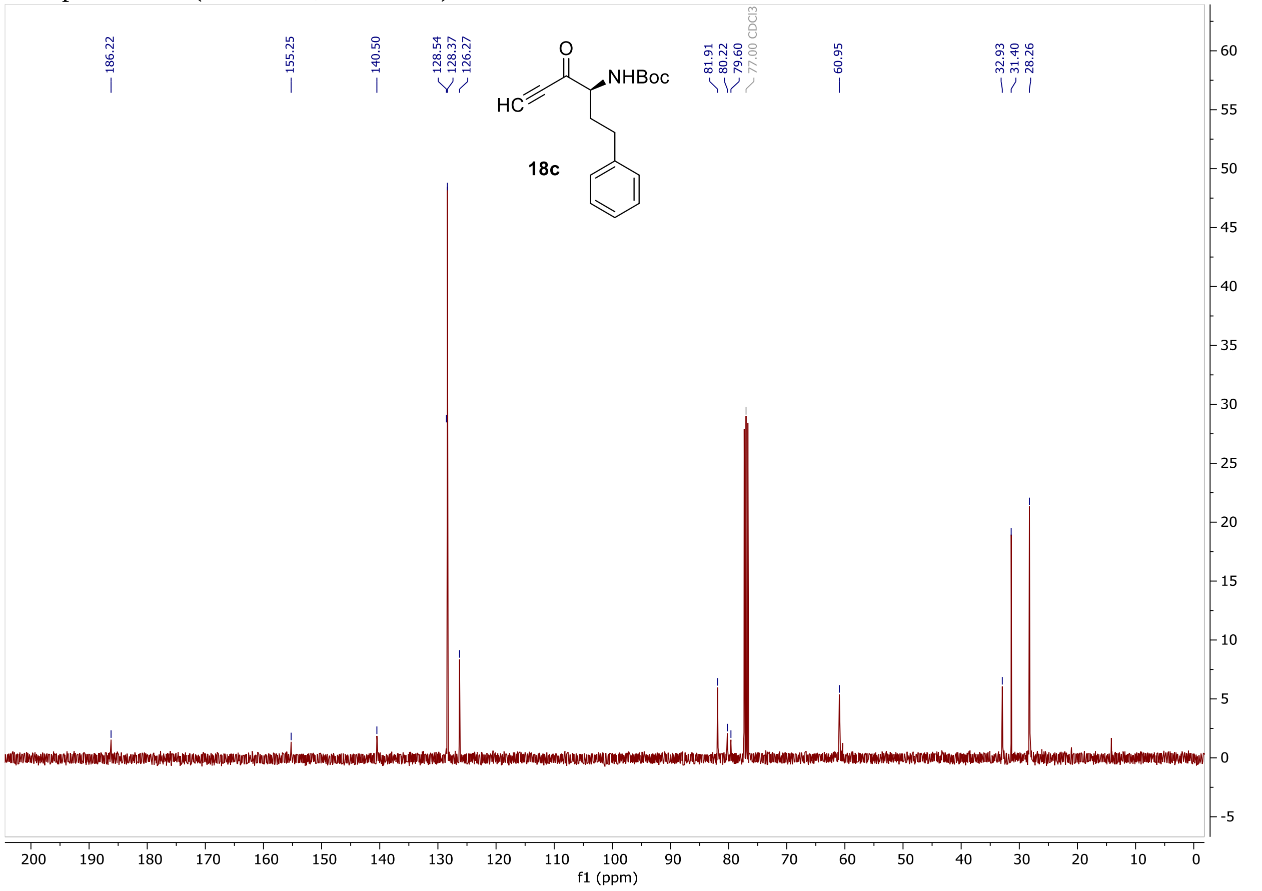

Compound **18d** (^{1}H NMR, 400 MHz):

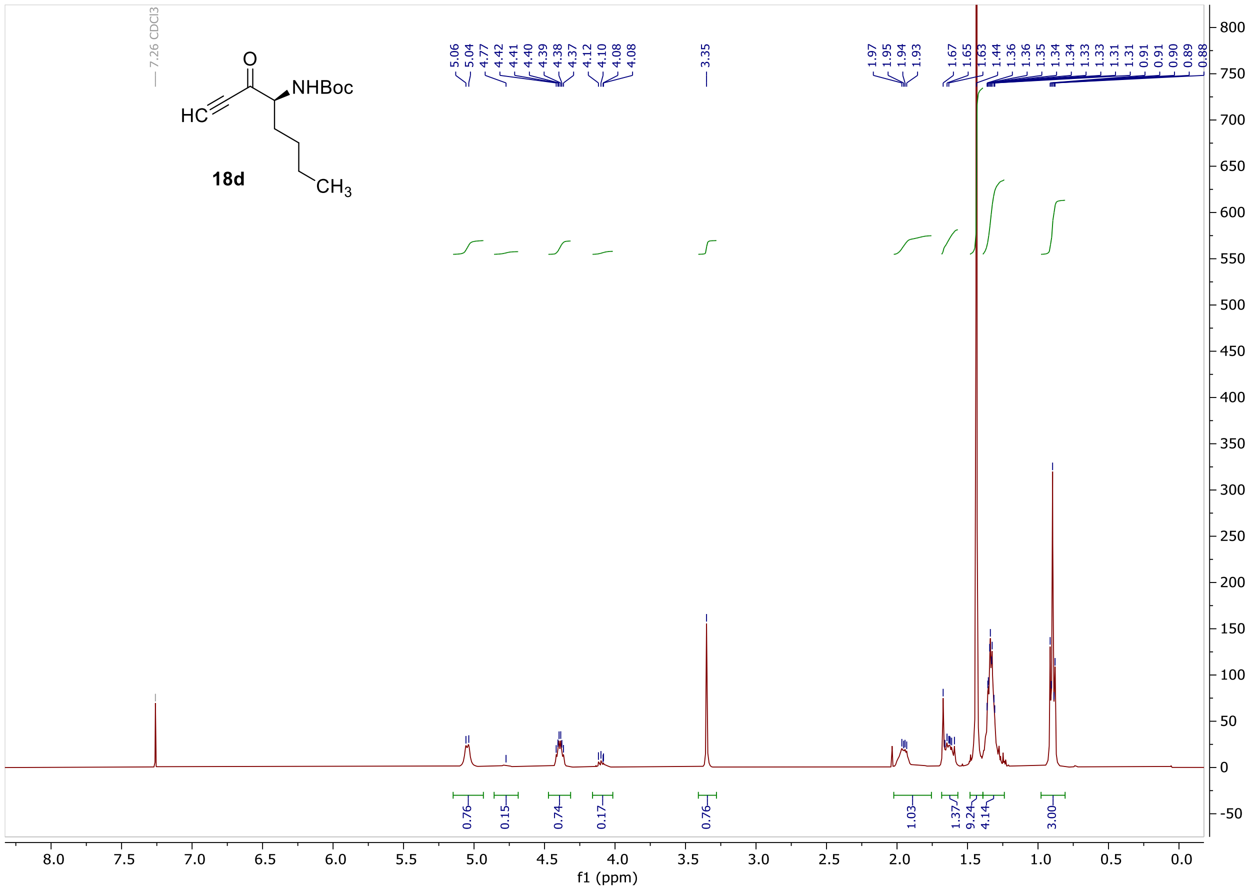

Compound **18d** (^{13}C NMR, 101 MHz):

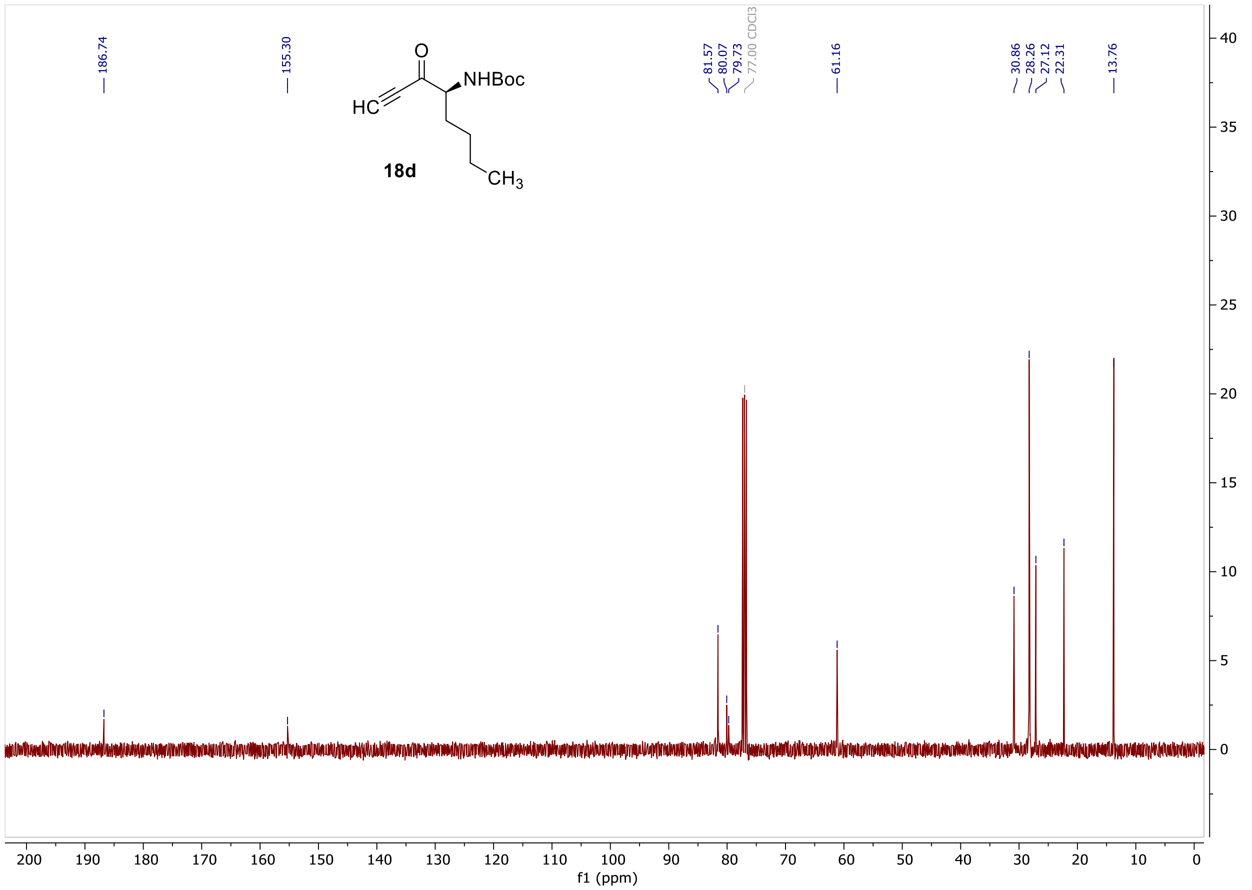

Compound **18e** (^{1}H NMR, 400 MHz):

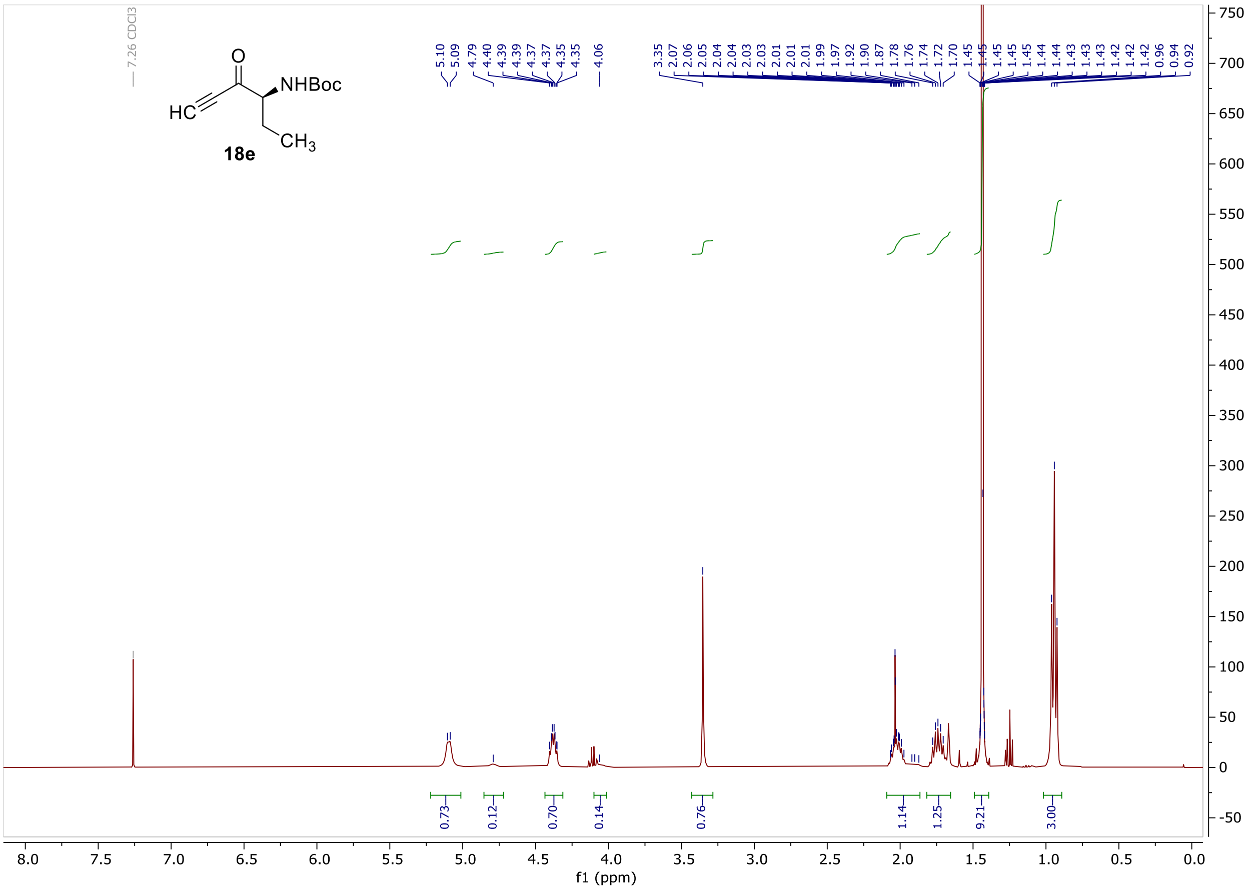

Compound **18e** (^{13}C NMR, 101 MHz):

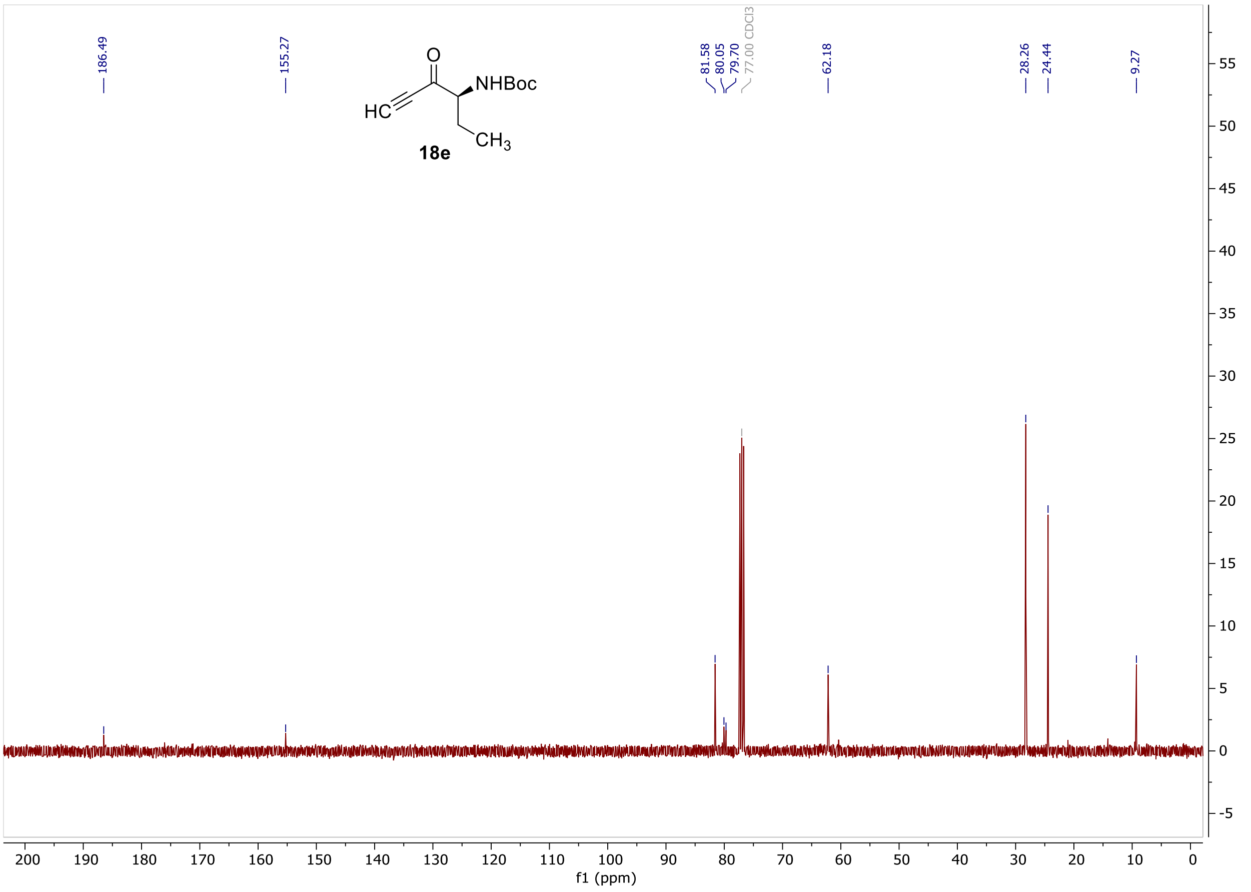

Compound **18f** (^{1}H NMR, 400 MHz):

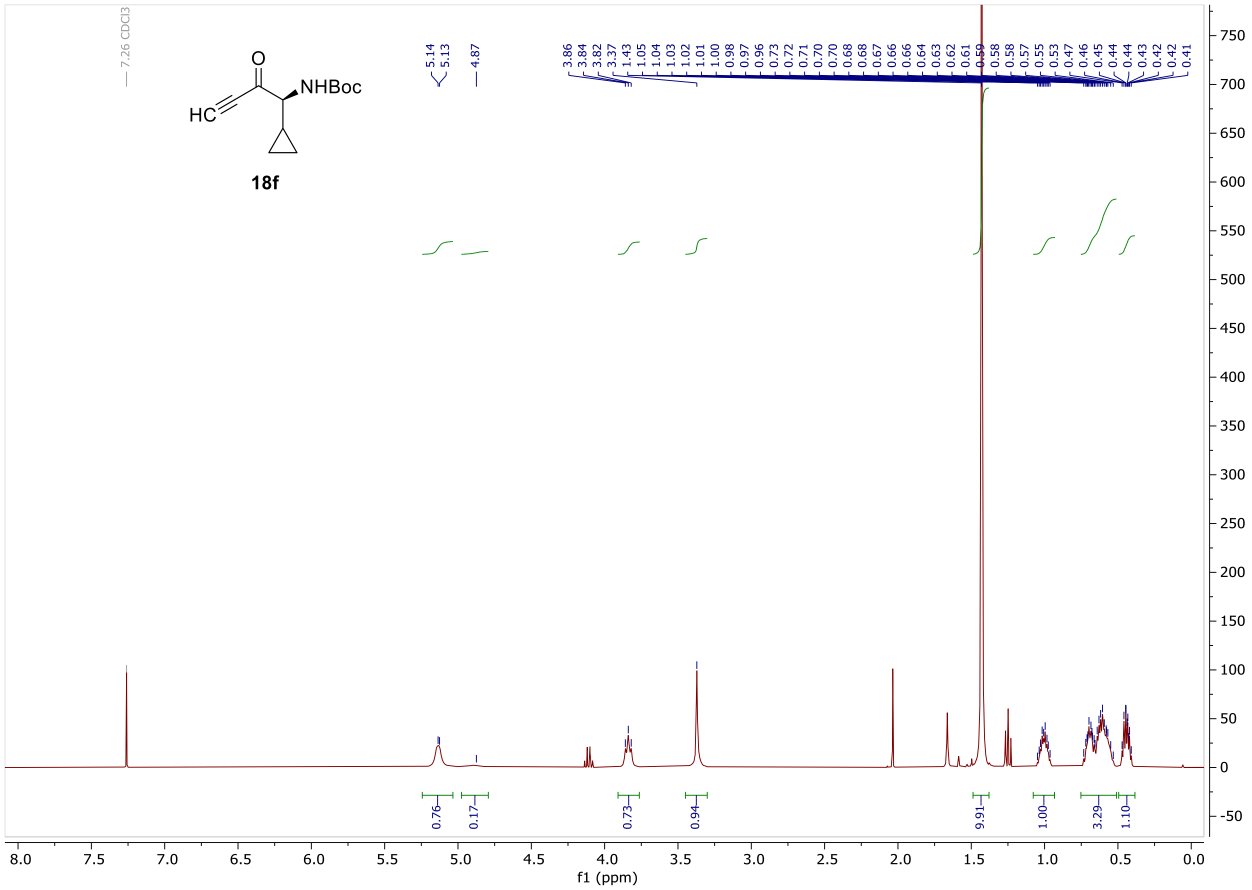

Compound **18f** (^{13}C NMR, 101 MHz):

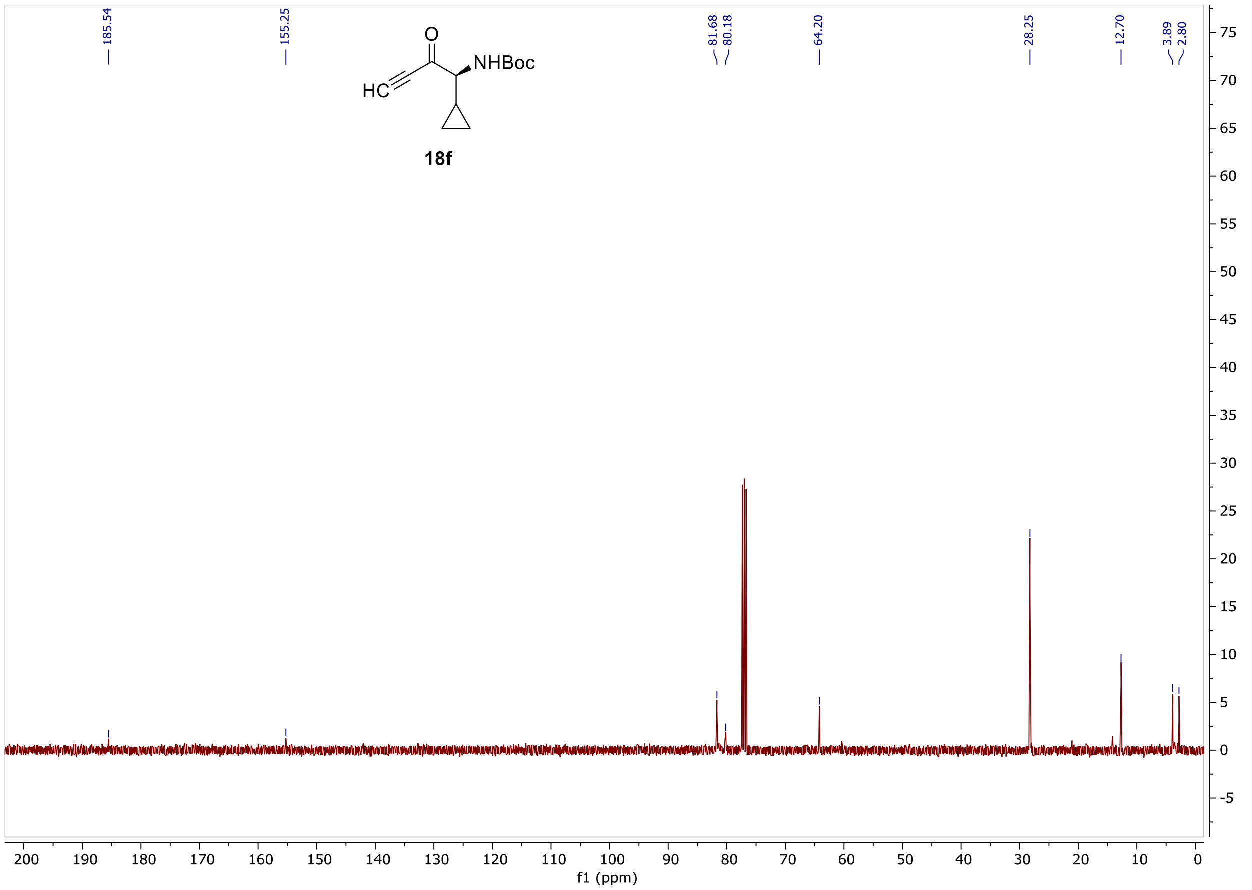

Compound **16** (^{1}H NMR, 400 MHz):

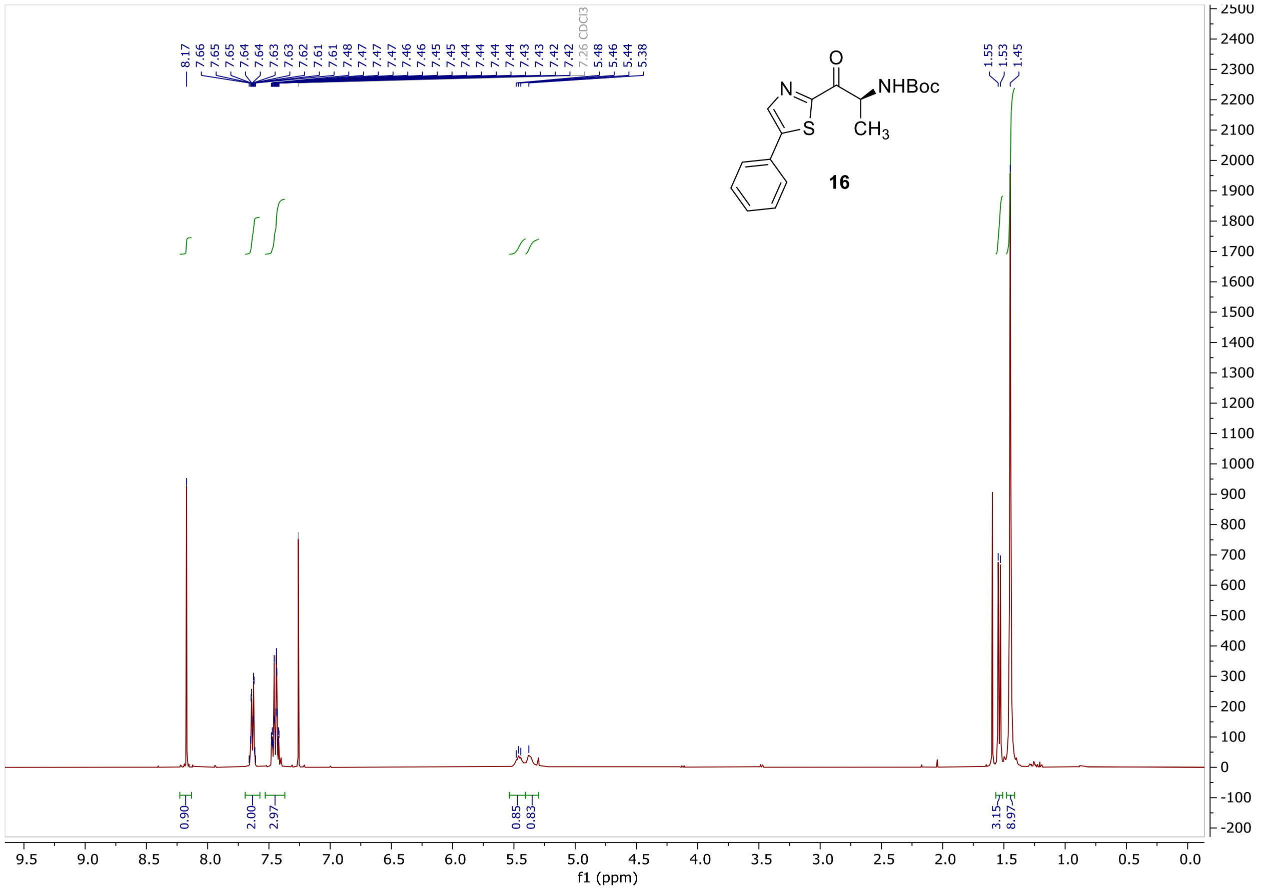

Compound **16** (^{13}C NMR, 101 MHz):

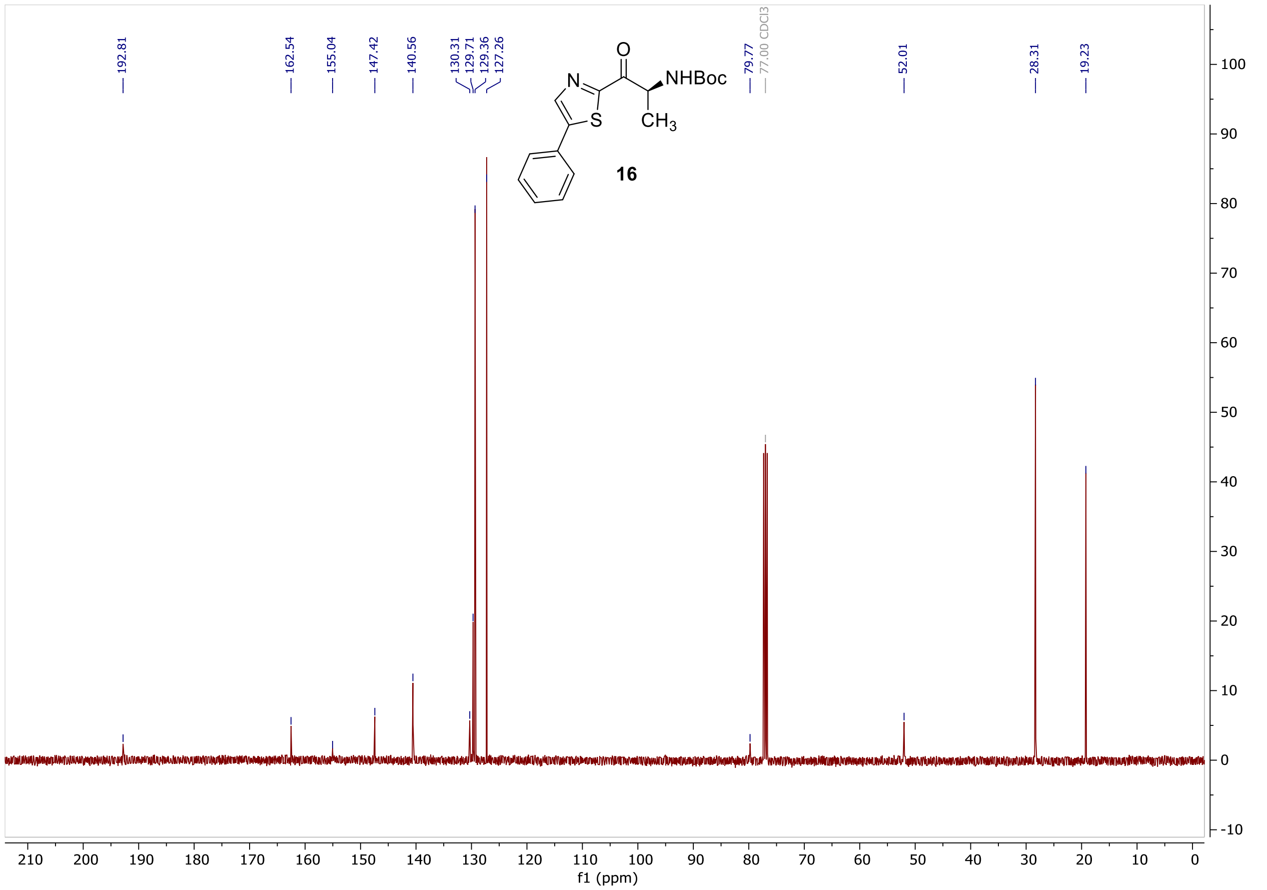

Compound **24a** (^{1}H NMR, 400 MHz):

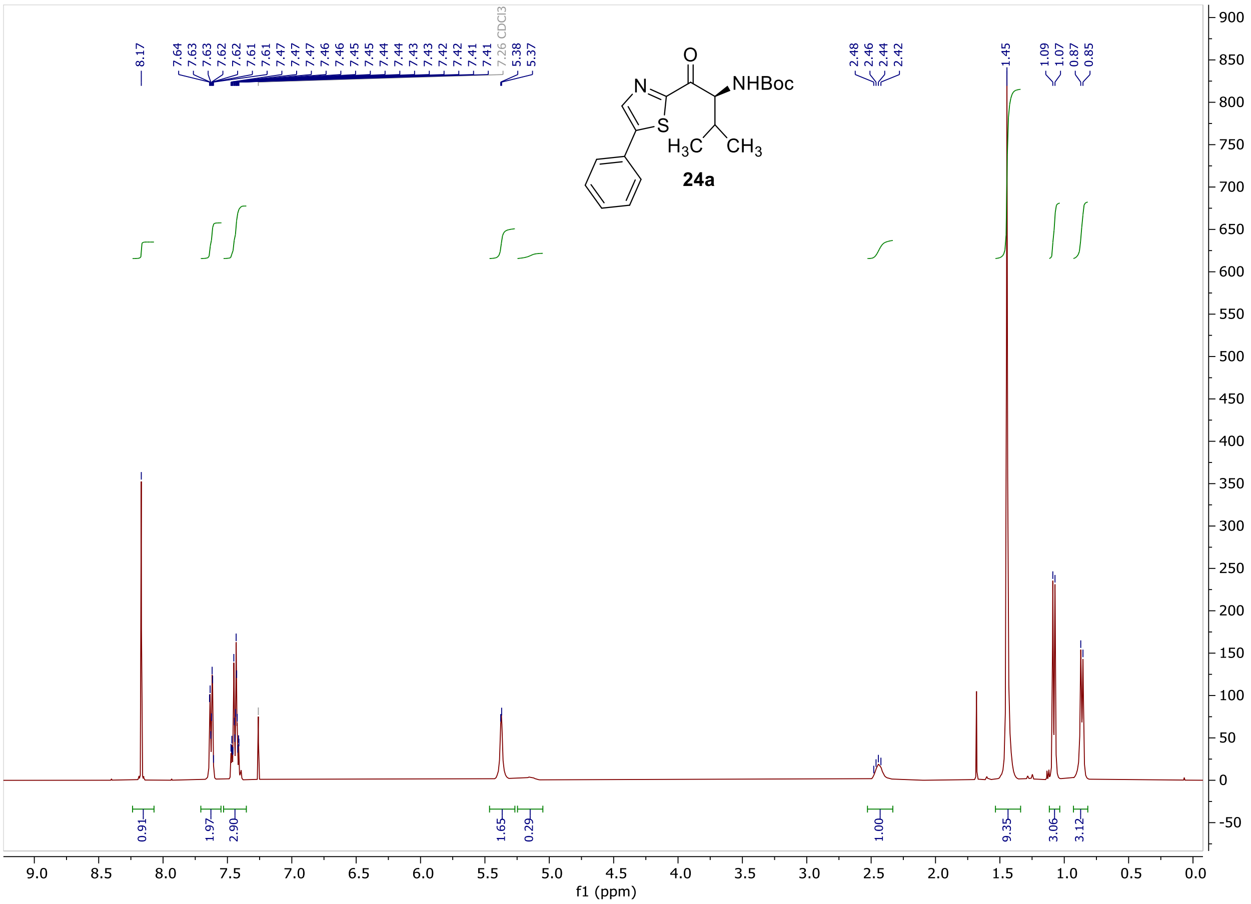

Compound **24a** (^{13}C NMR, 101 MHz):

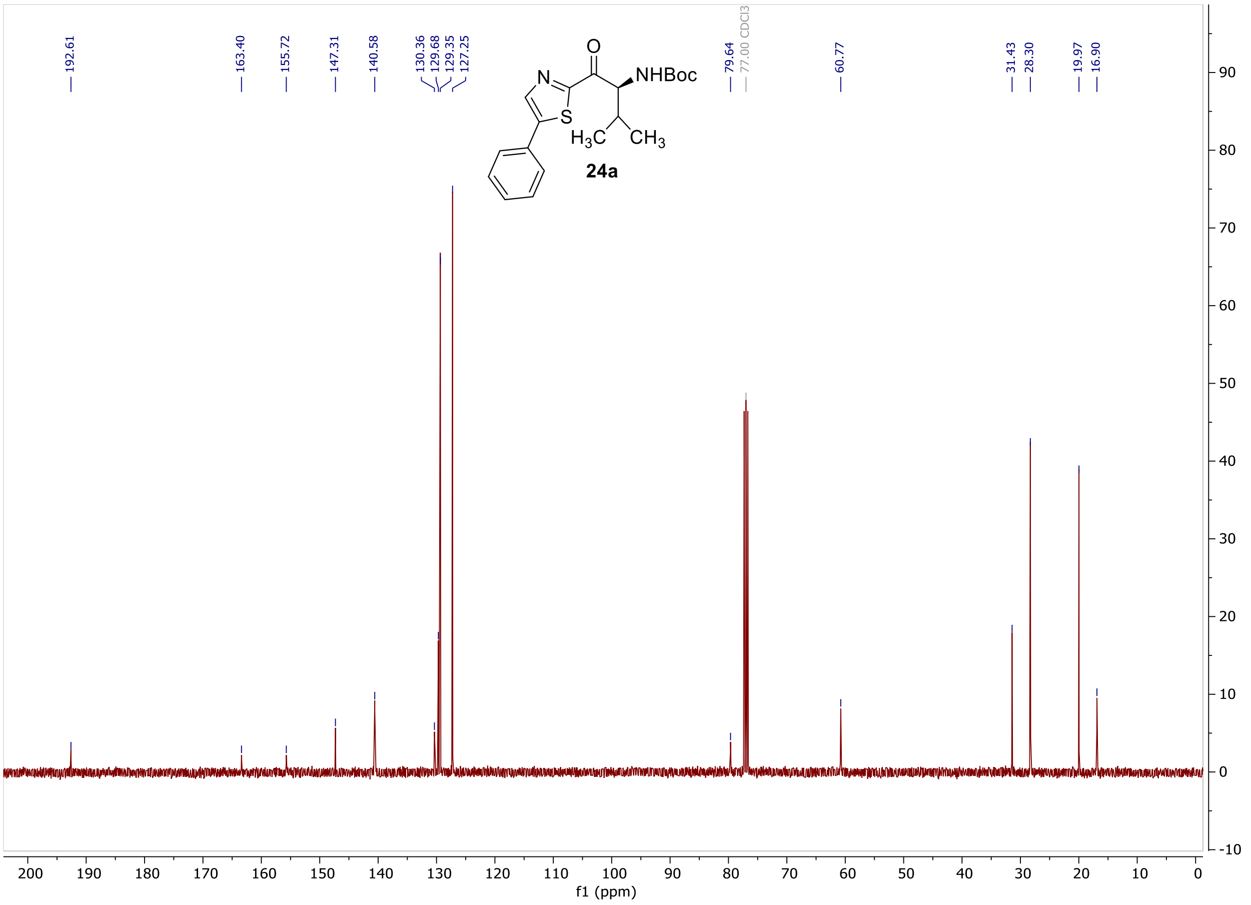

Compound **24b** (^{1}H NMR, 400 MHz):

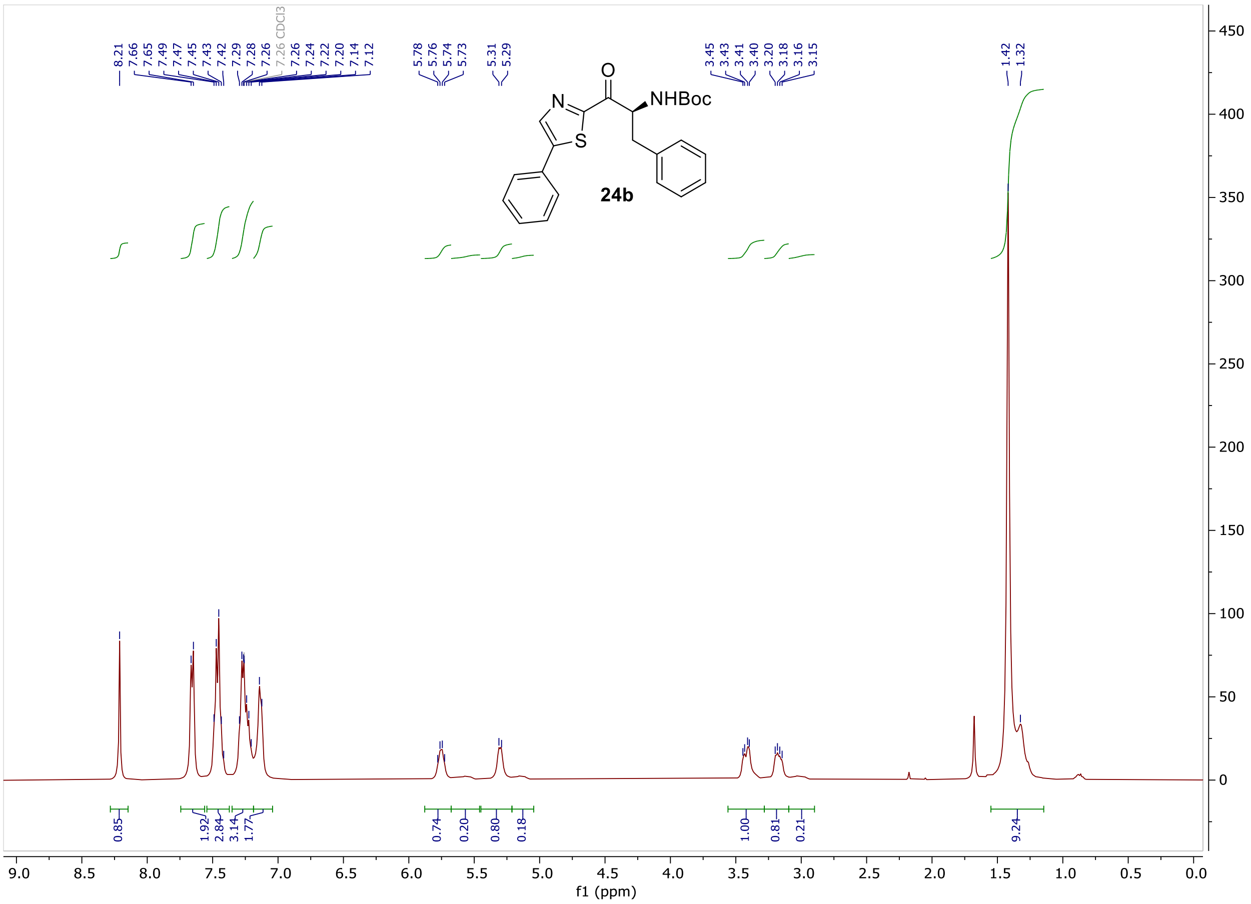

Compound **24b** (^{13}C NMR, 101 MHz):

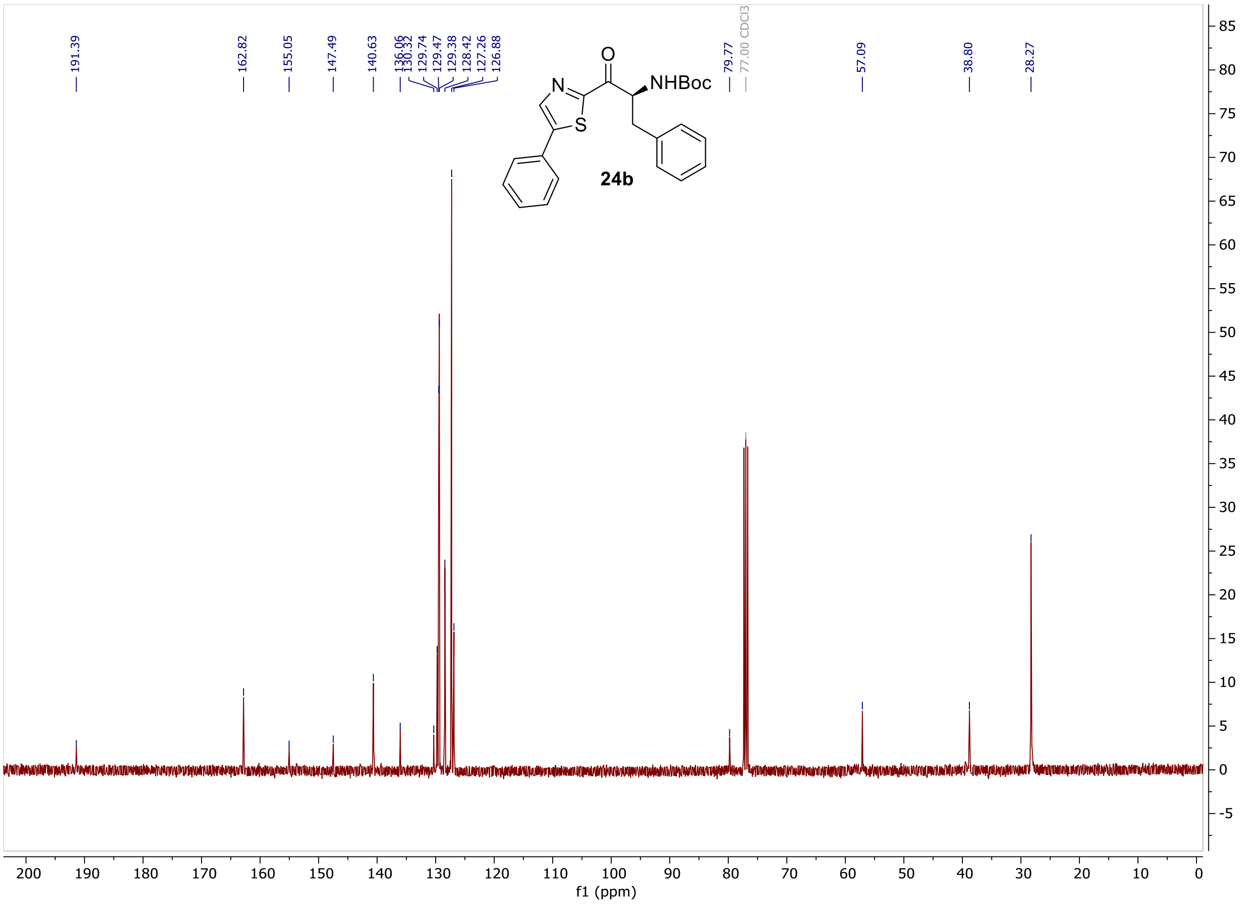

Compound **24c** (^{1}H NMR, 400 MHz):

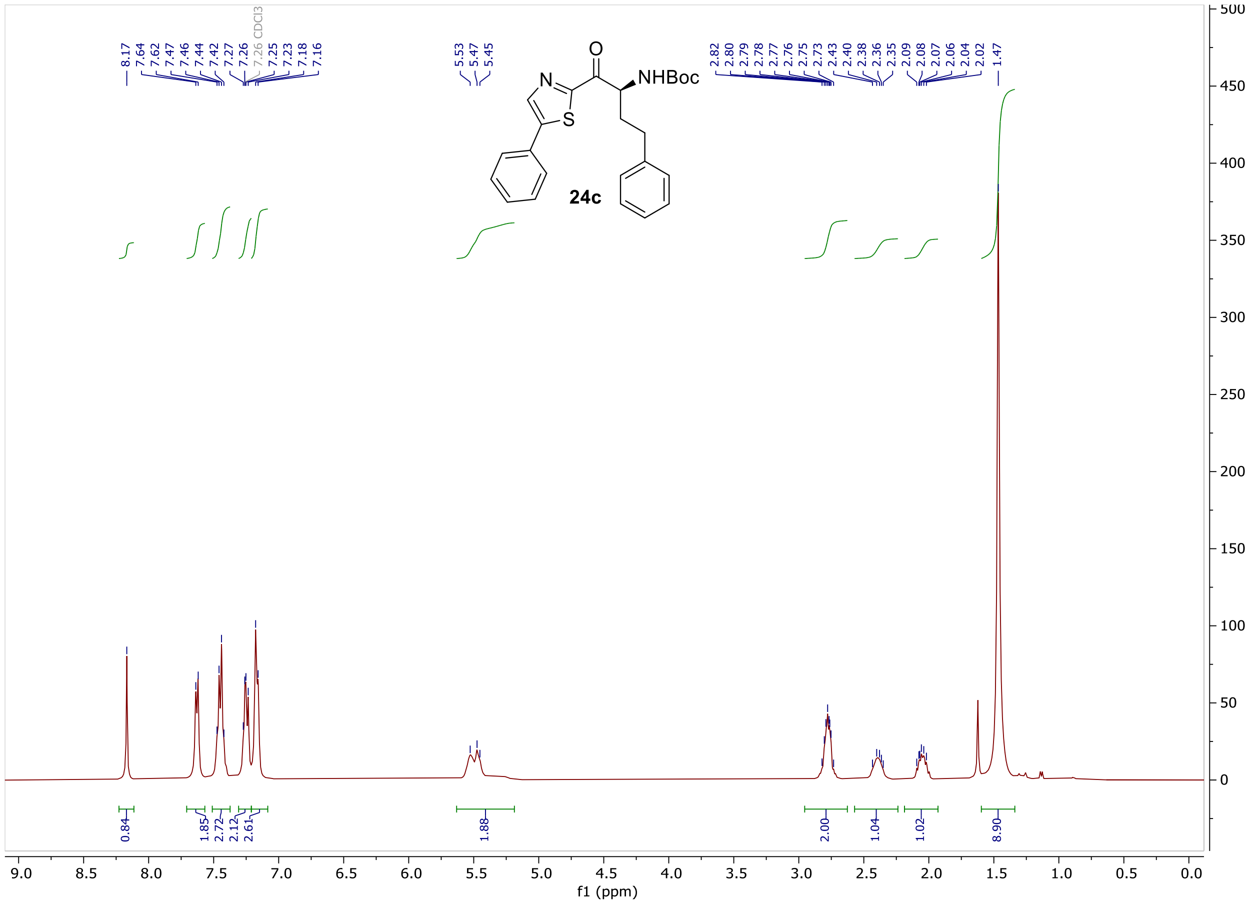

Compound **24c** (^{13}C NMR, 101 MHz):

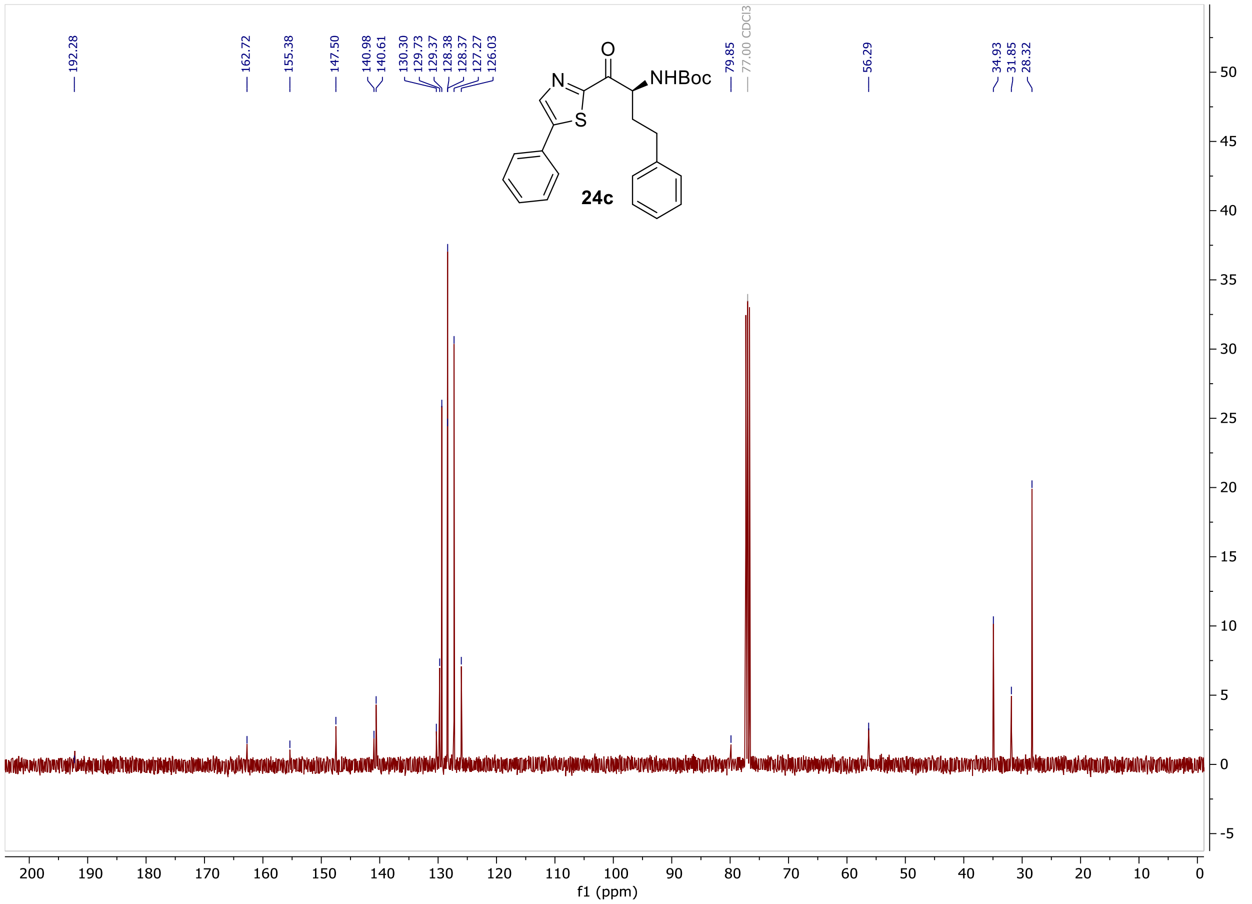

Compound **24d** (^{1}H NMR, 400 MHz):

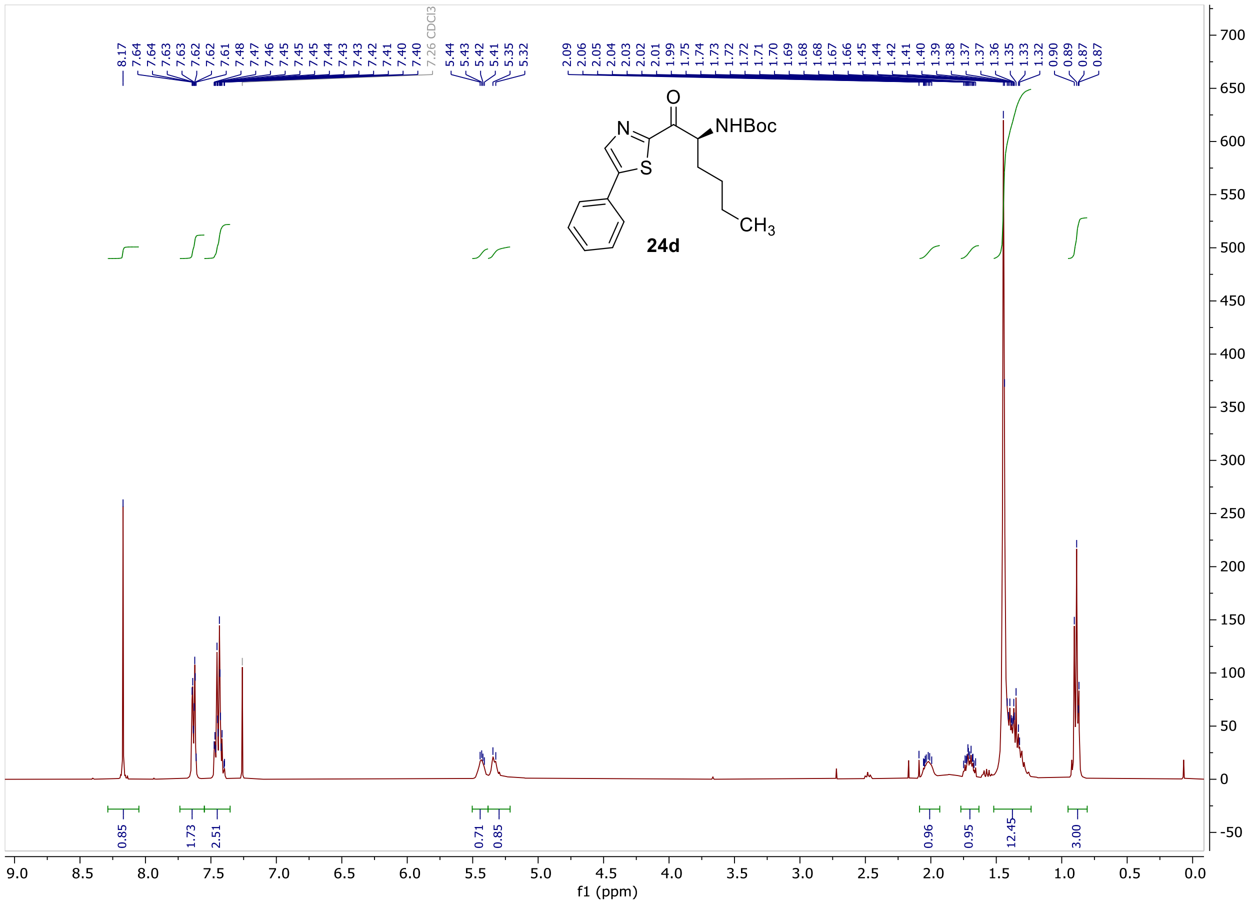

Compound **24d** (^{13}C NMR, 101 MHz):

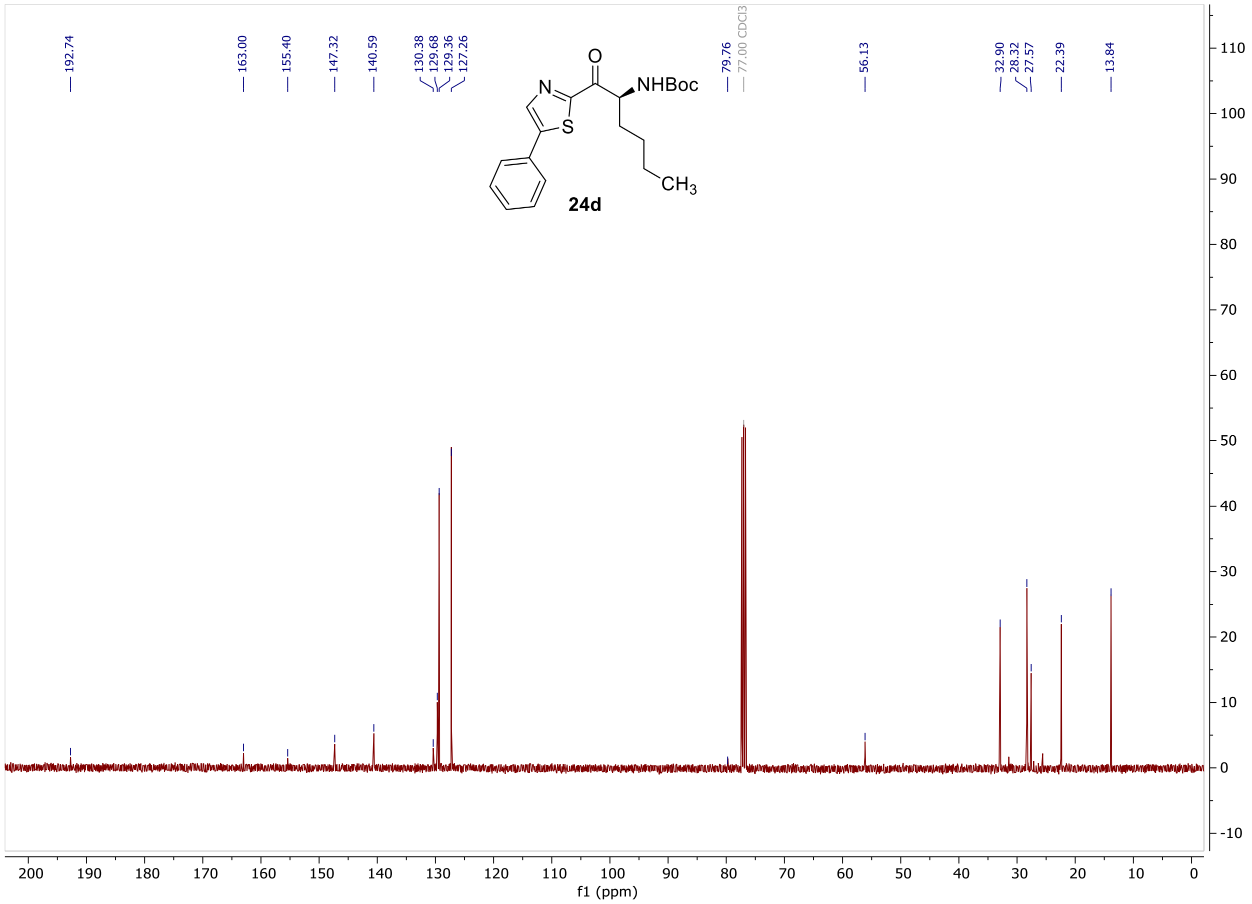

Compound **24e** (^{1}H NMR, 400 MHz):

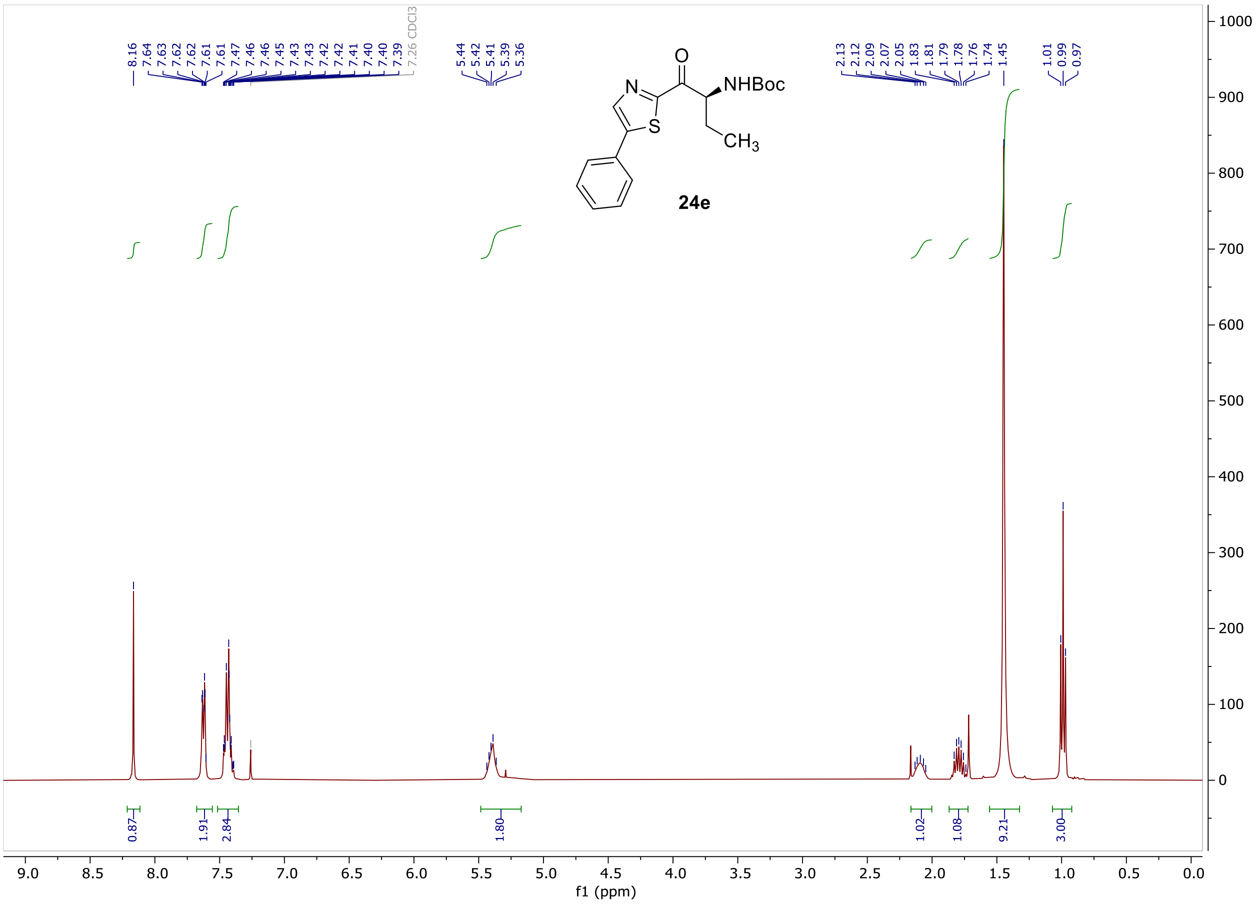

Compound **24e** (^{13}C NMR, 101 MHz):

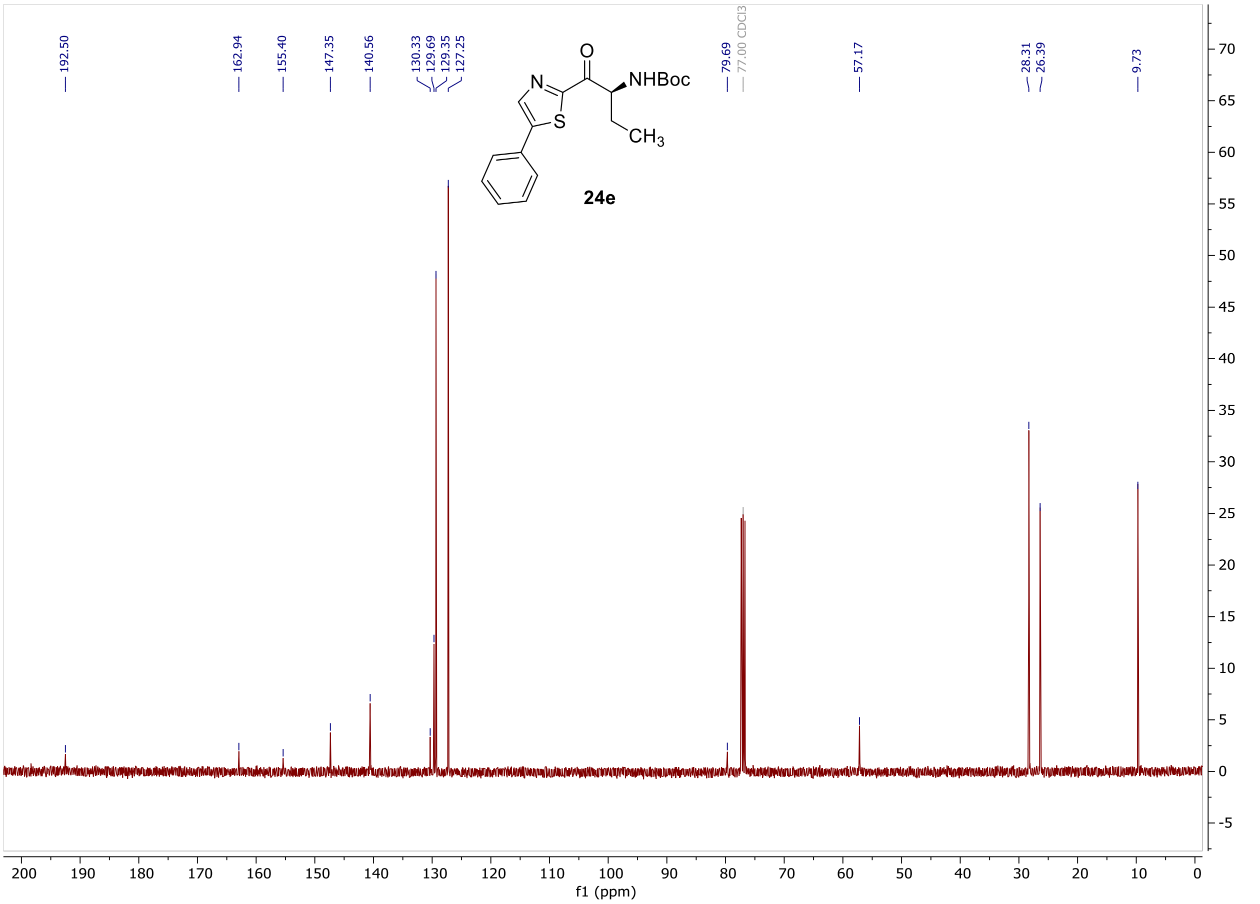

Compound **24f** (^{1}H NMR, 400 MHz):

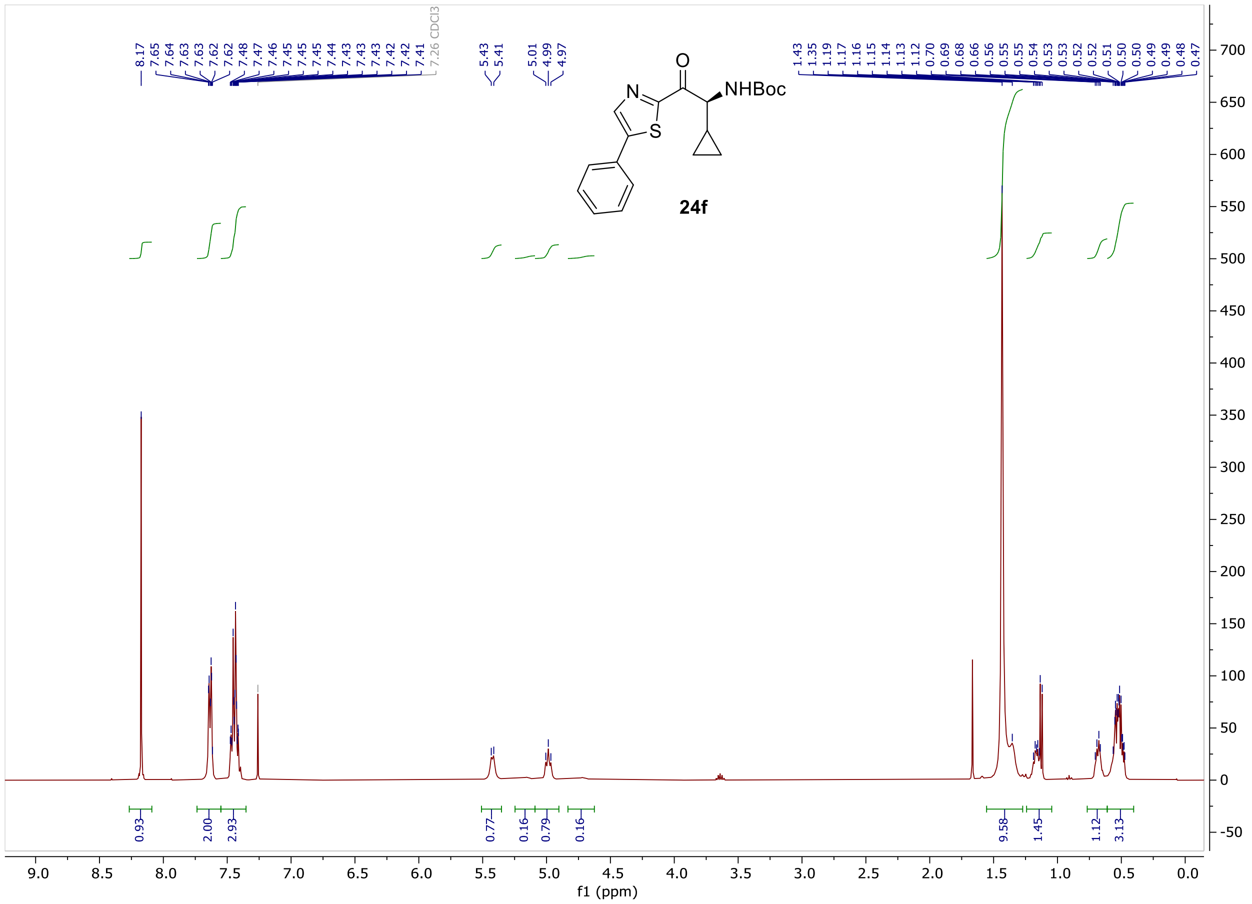

Compound **24f** (^{13}C NMR, 101 MHz):

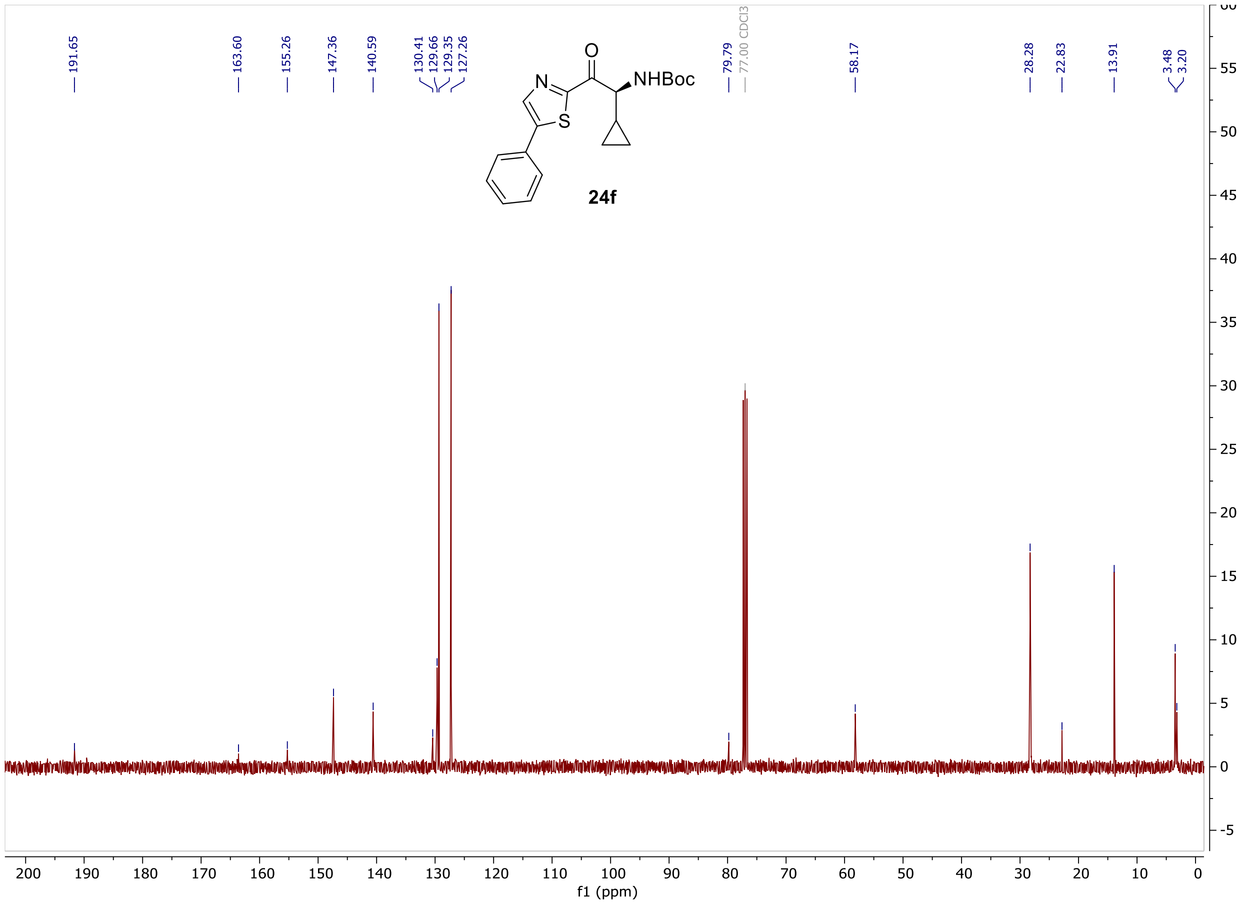

Compound **39a** (^{1}H NMR, 400 MHz):

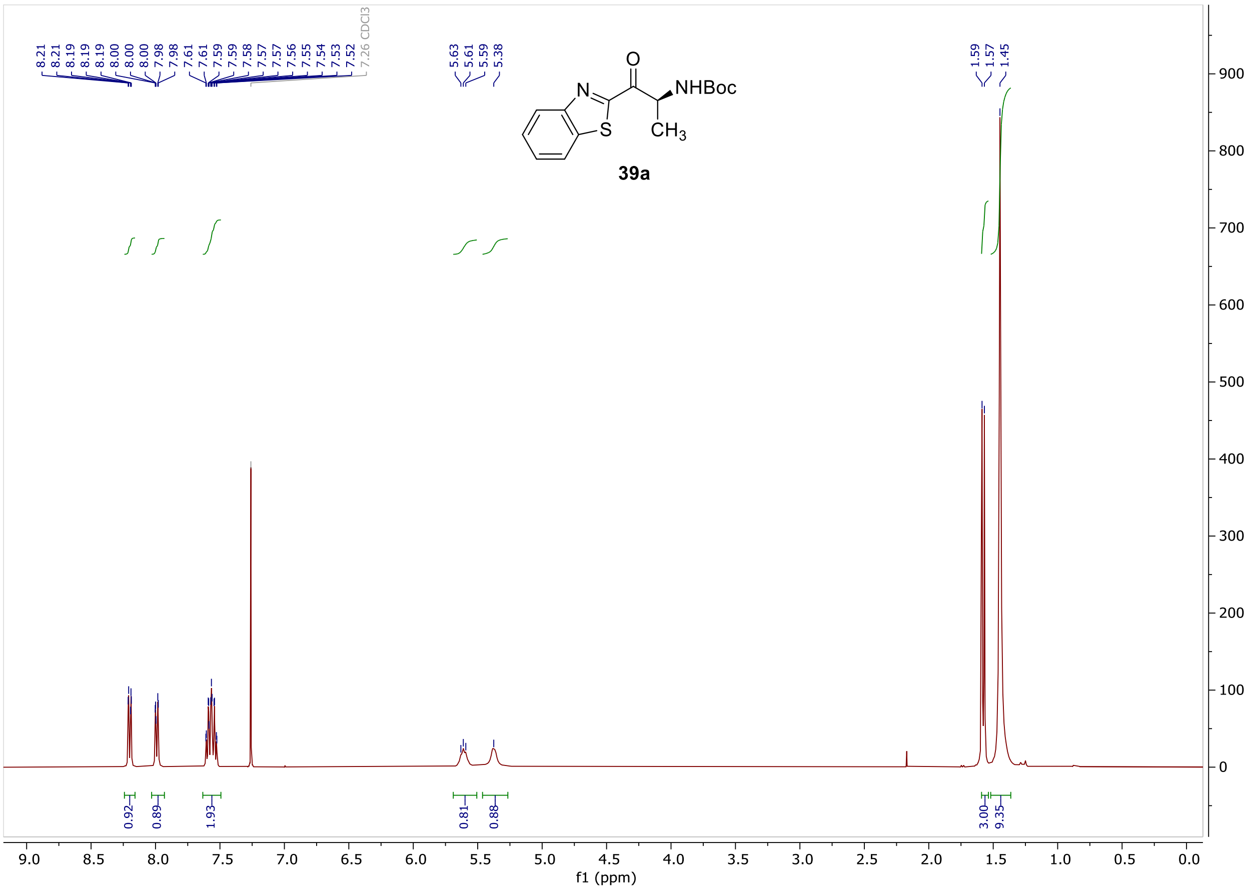

Compound **39a** (^{13}C NMR, 101 MHz):

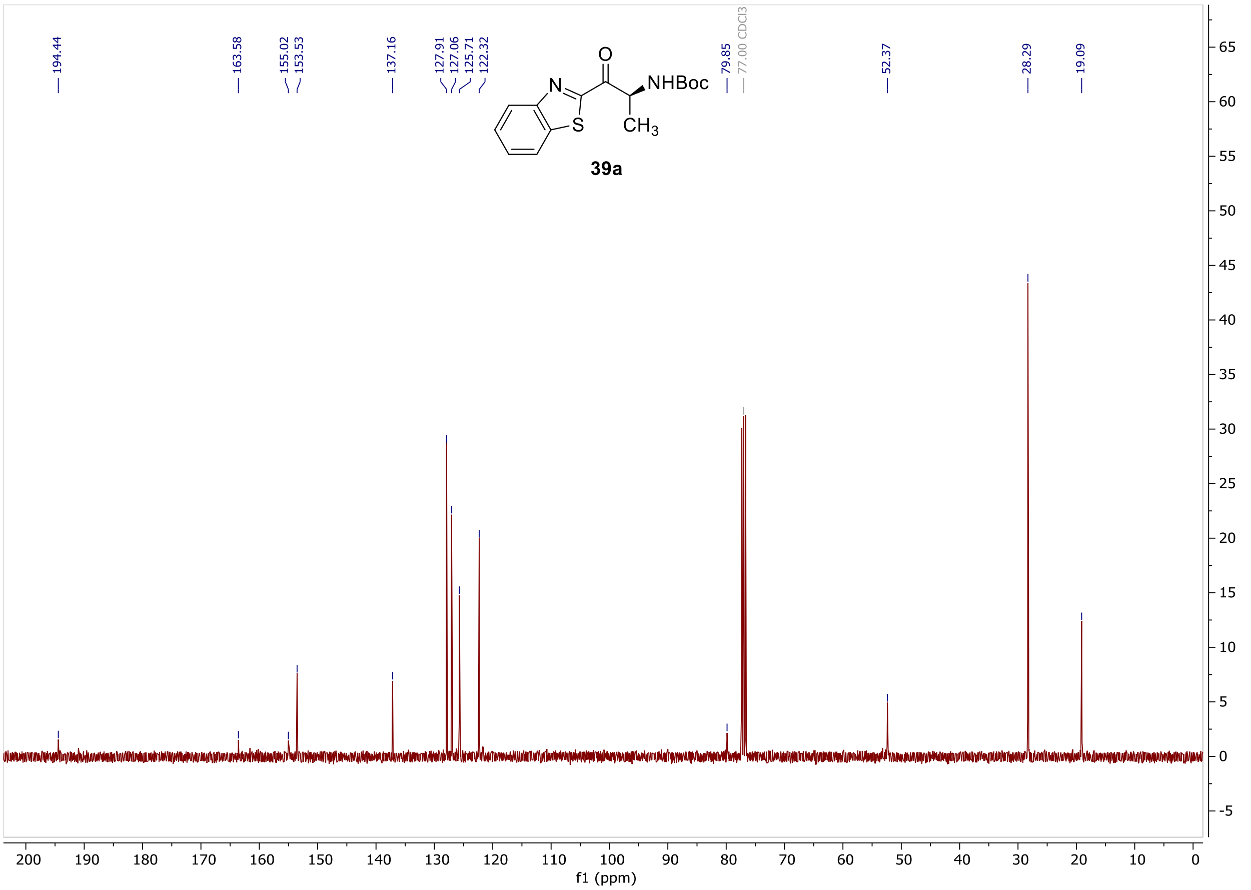

Compound **39b** (^{1}H NMR, 400 MHz):

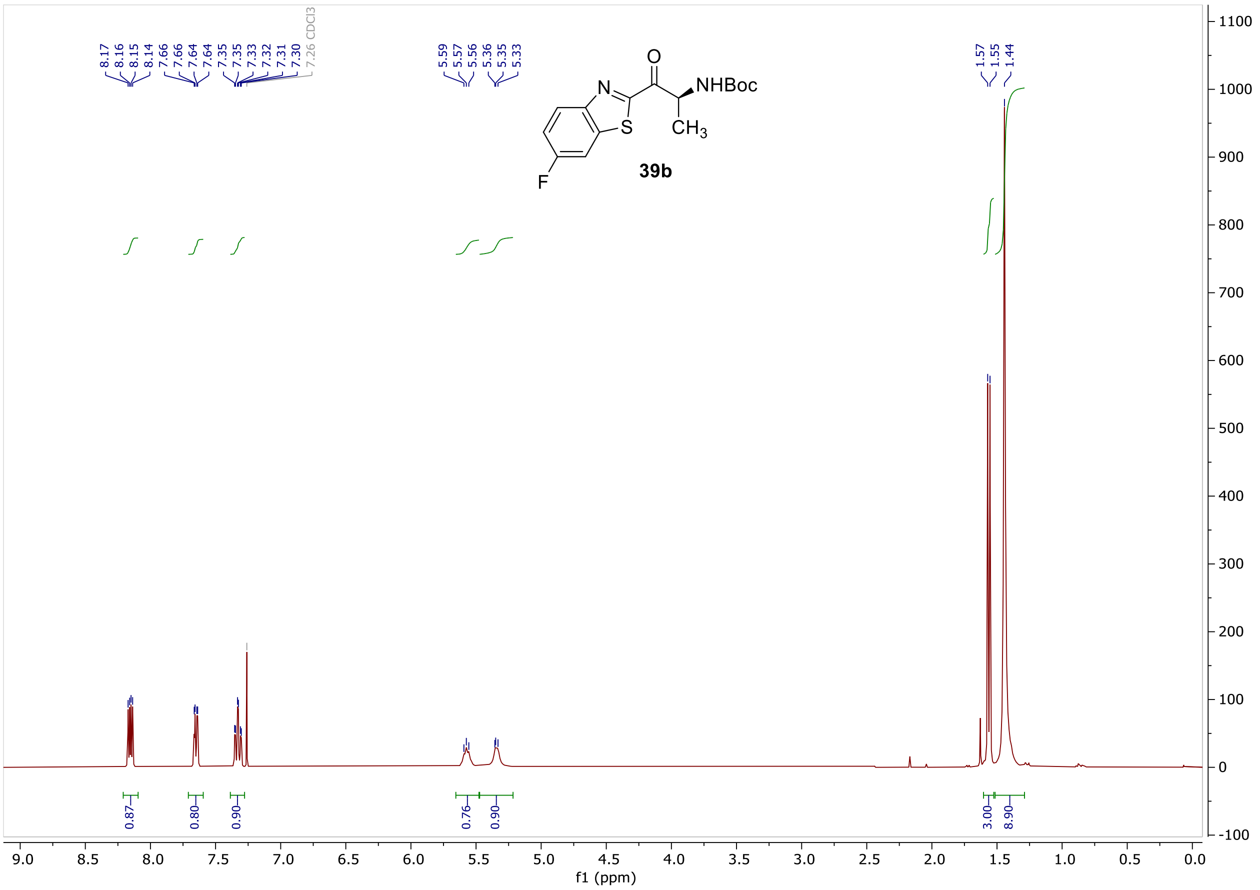

Compound **39b** (^{13}C NMR, 101 MHz):

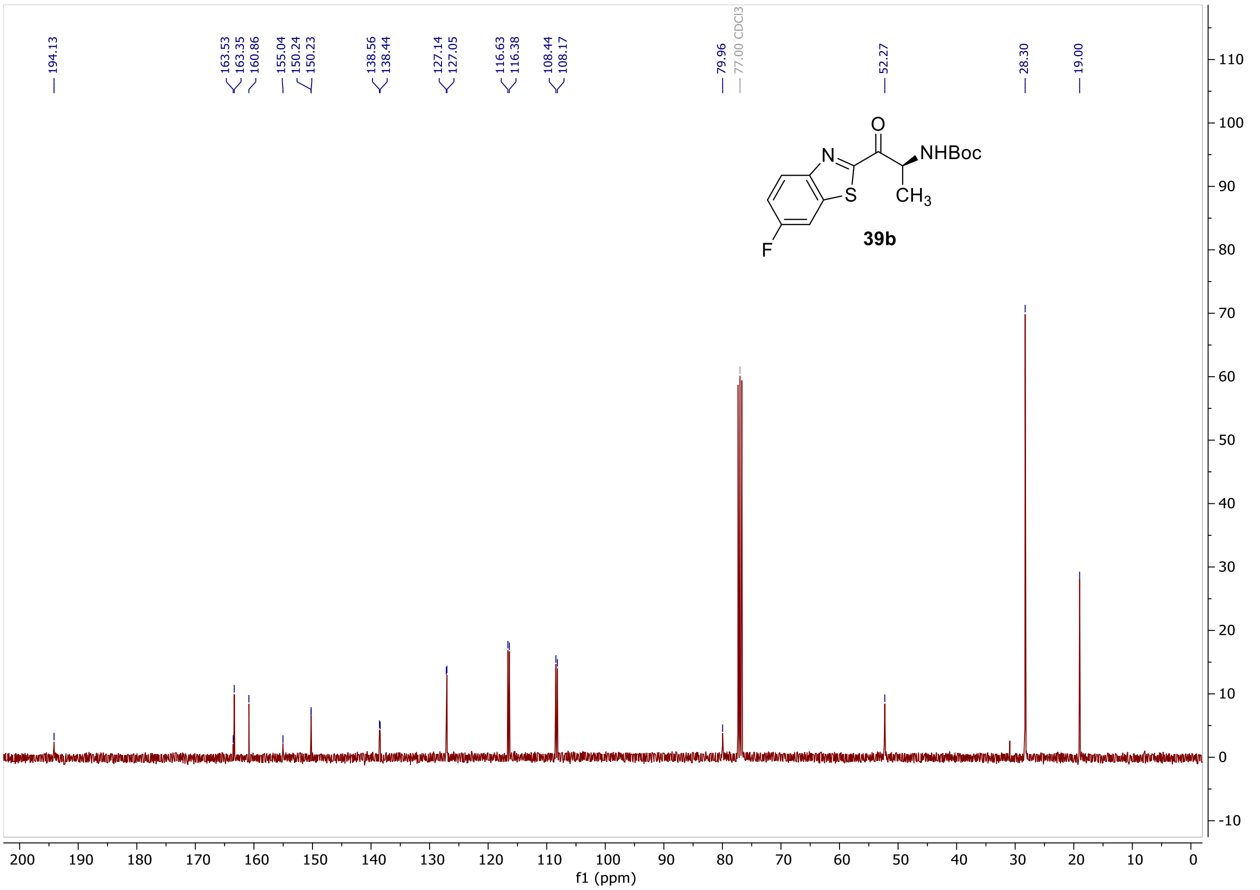

Compound **39b** (^{19}F NMR, 282 MHz):

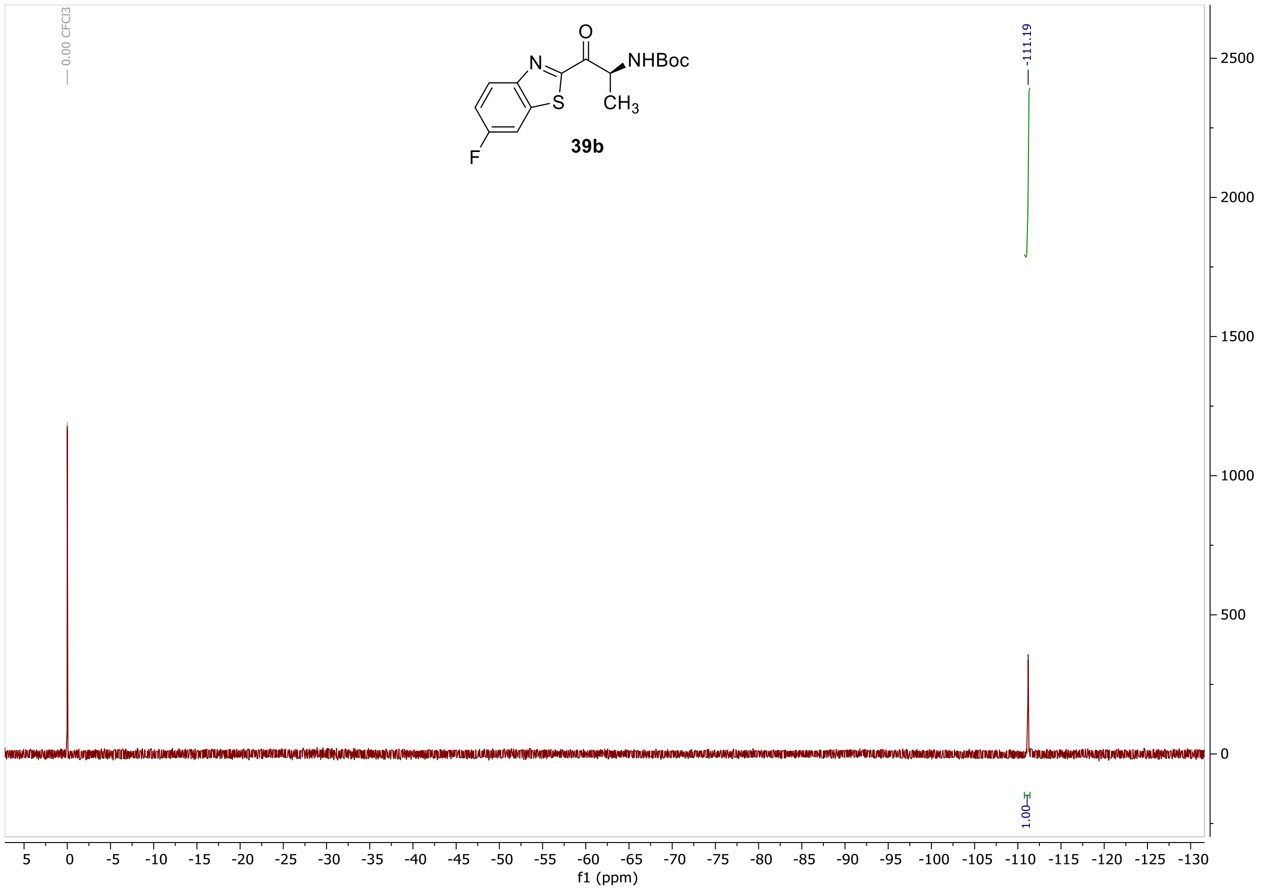

Compound **39c** (^{1}H NMR, 400 MHz):

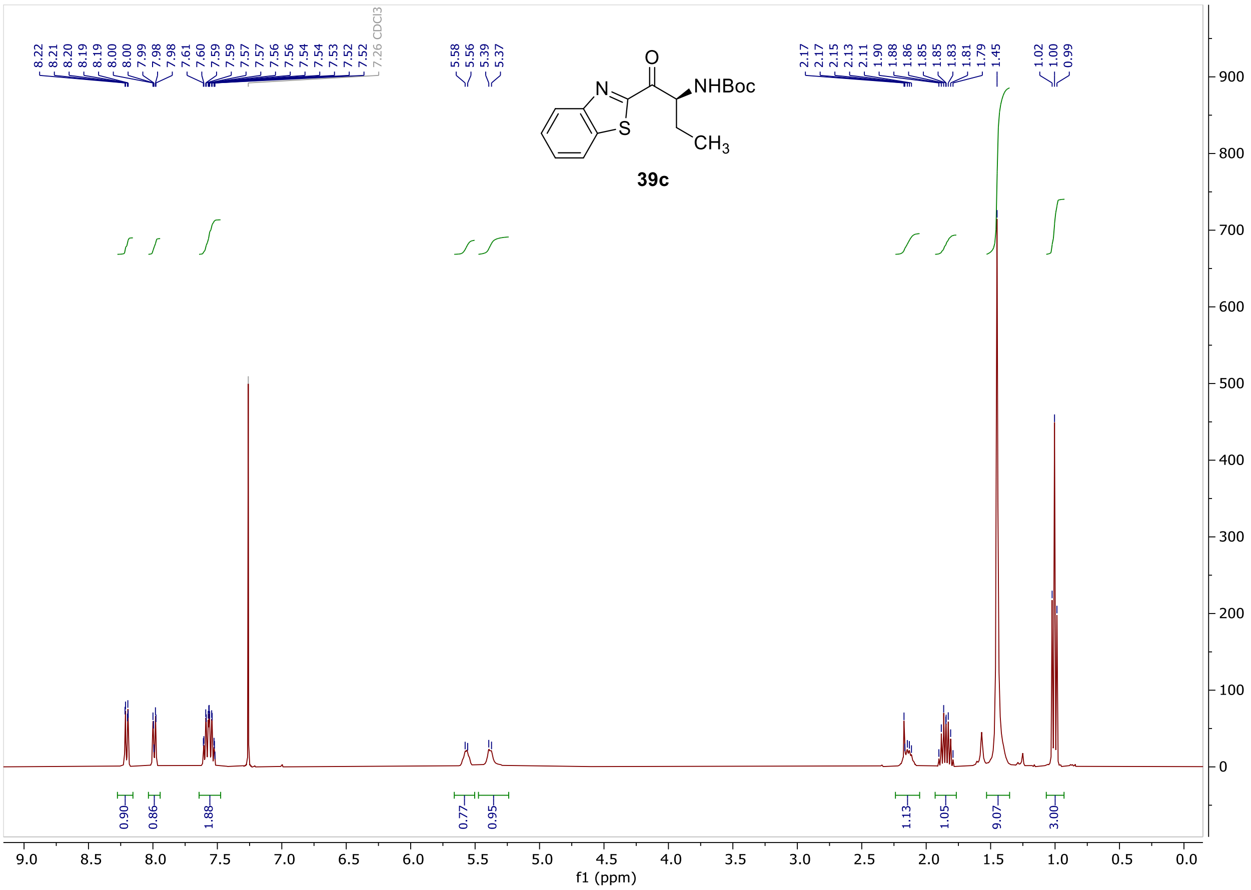

Compound **39c** (^{13}C NMR, 101 MHz):

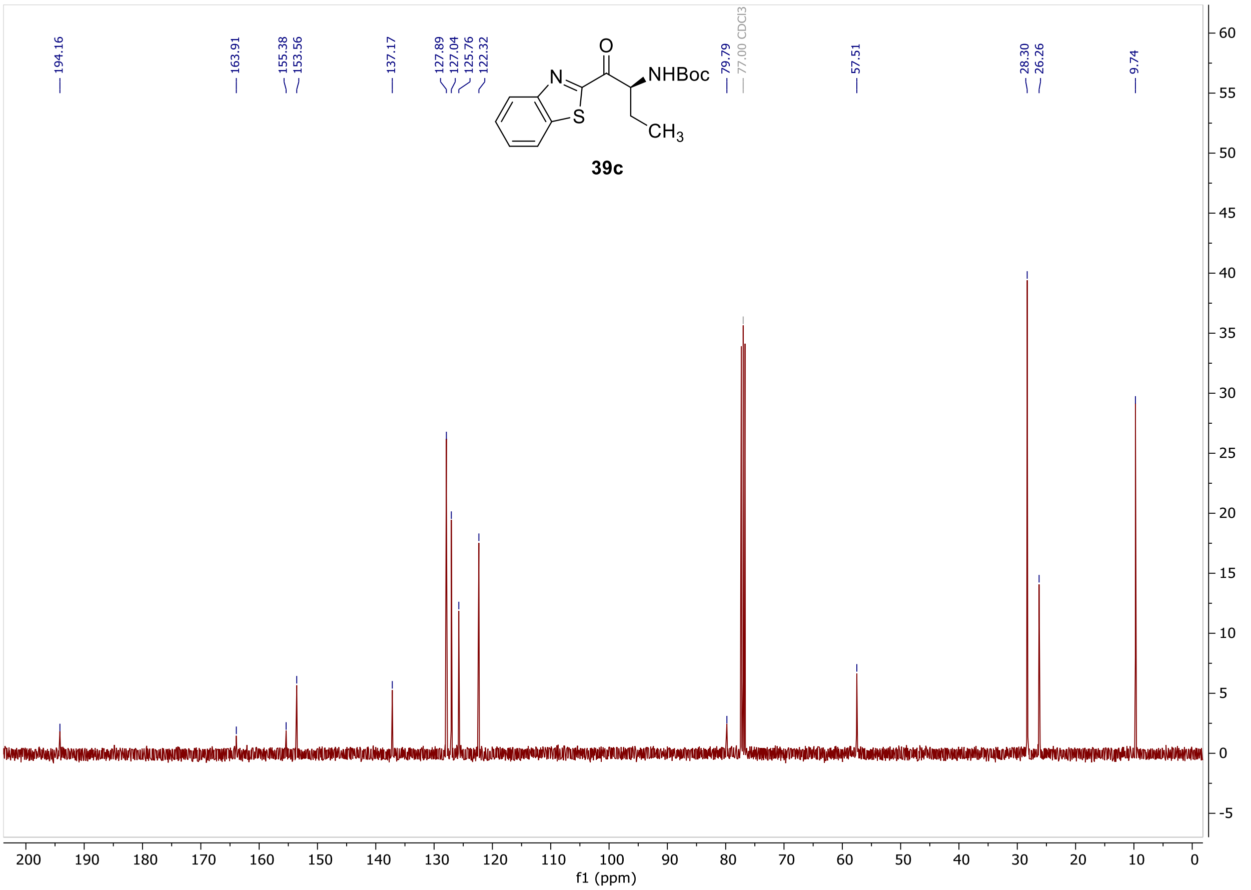

Compound **39d** (^{1}H NMR, 400 MHz):

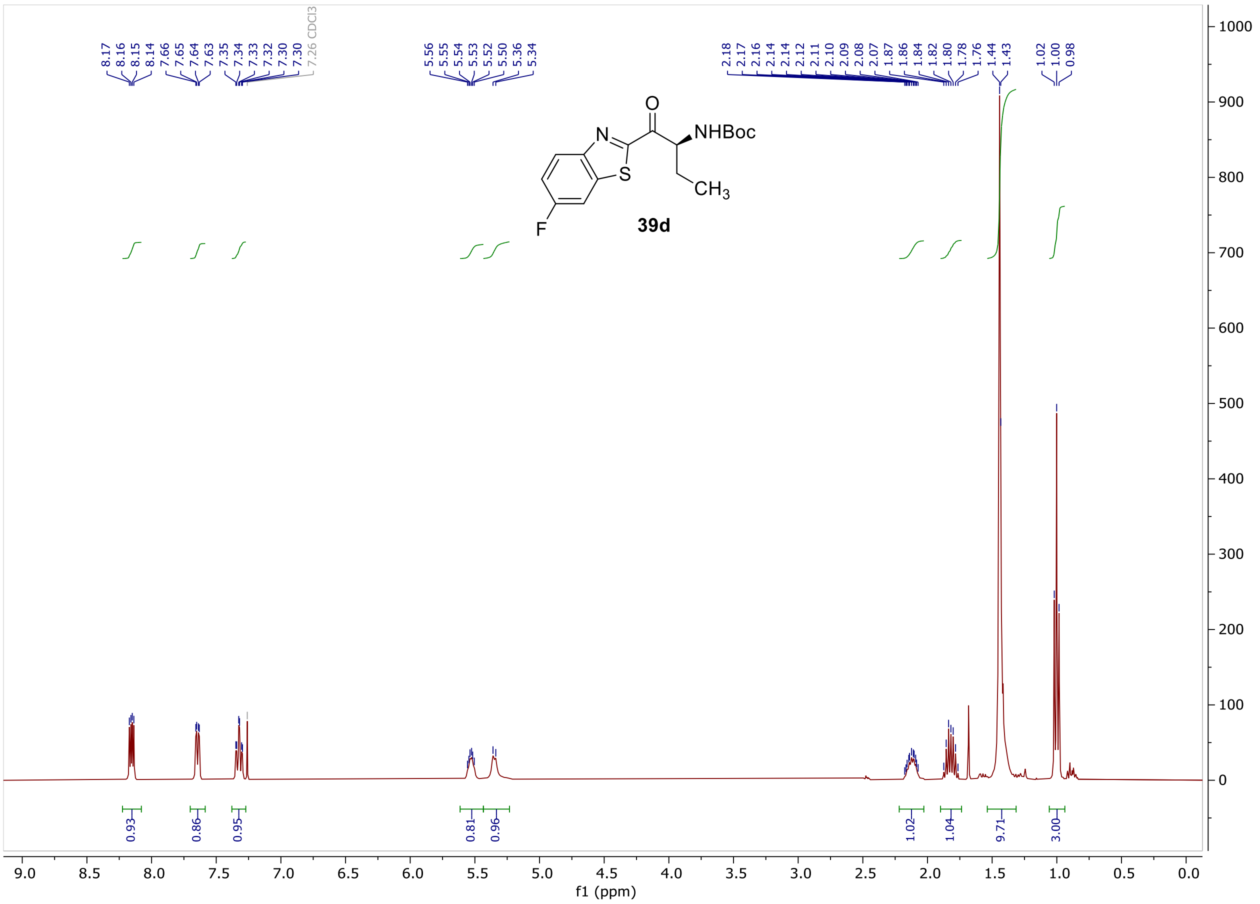

Compound **39d** (^{13}C NMR, 101 MHz):

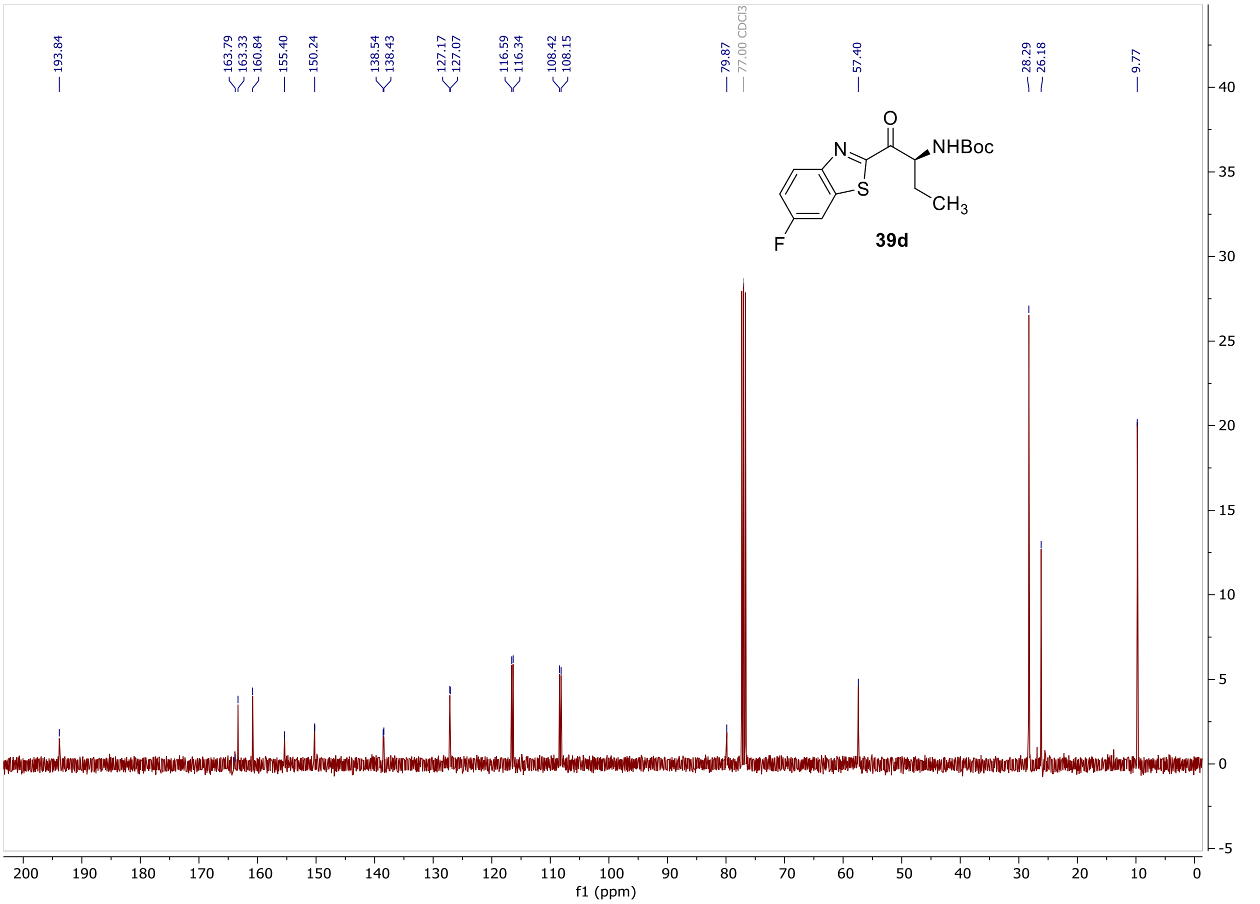

Compound **39d** (^{19}F NMR, 282 MHz):

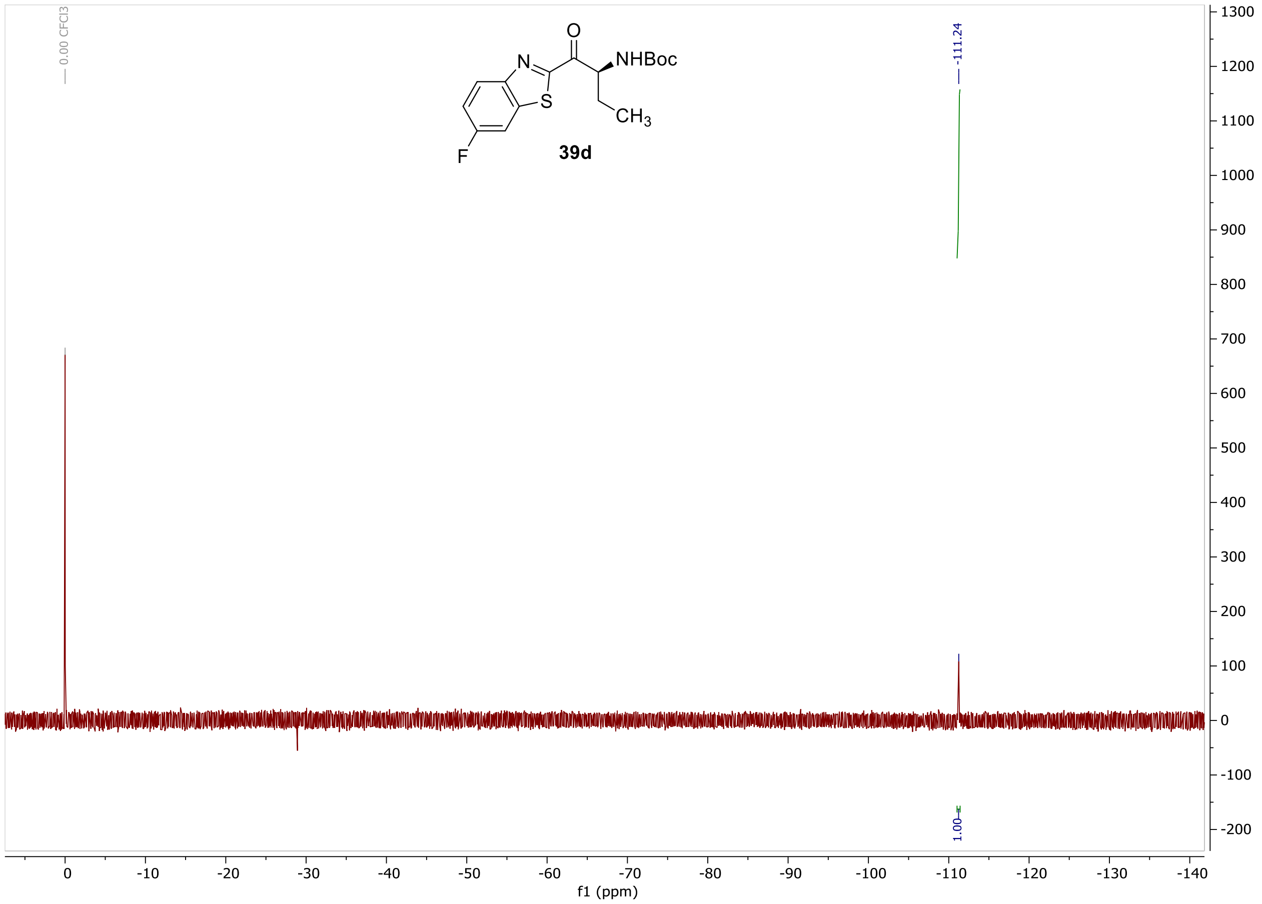

Compound **42b** (^{1}H NMR, 400 MHz):

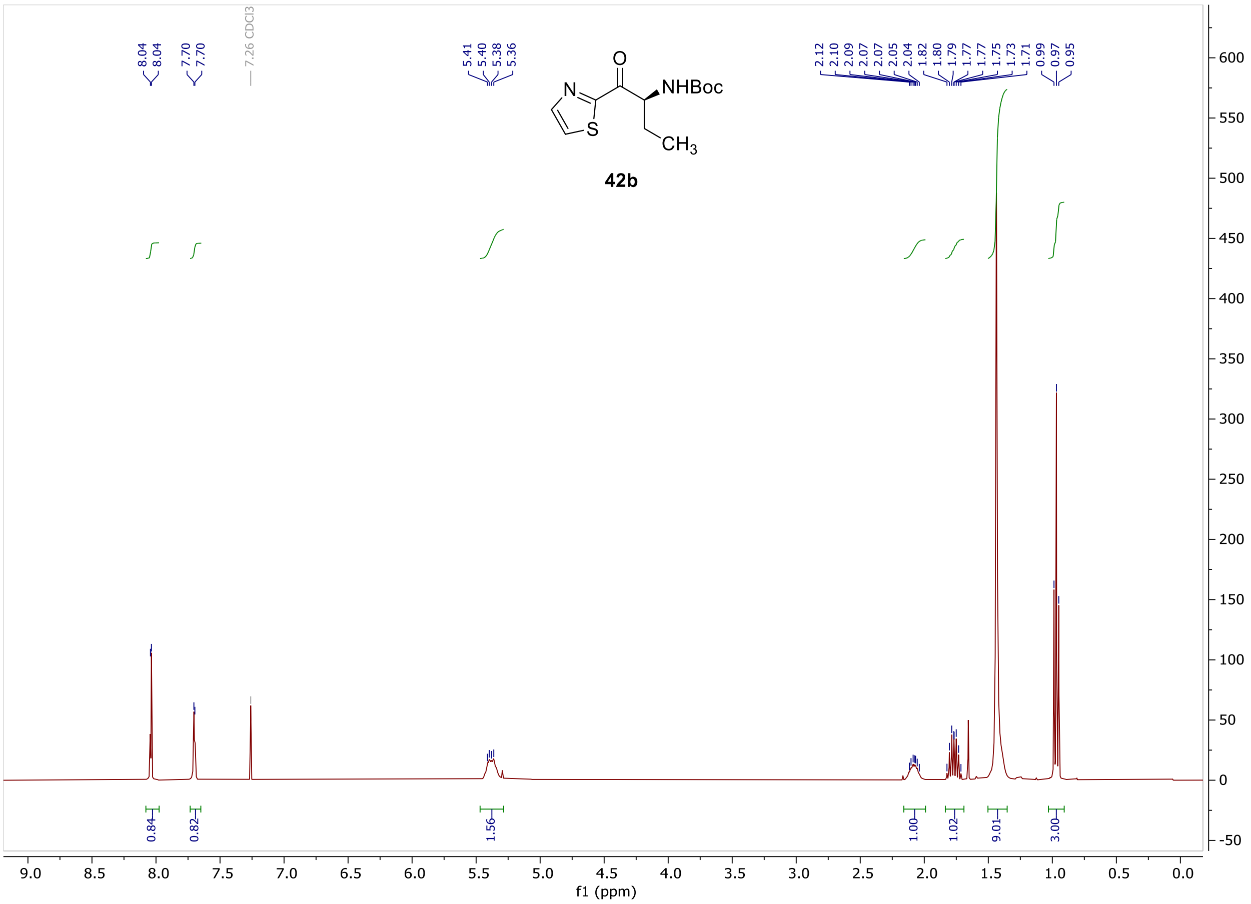

Compound **42b** (^{13}C NMR, 101 MHz):

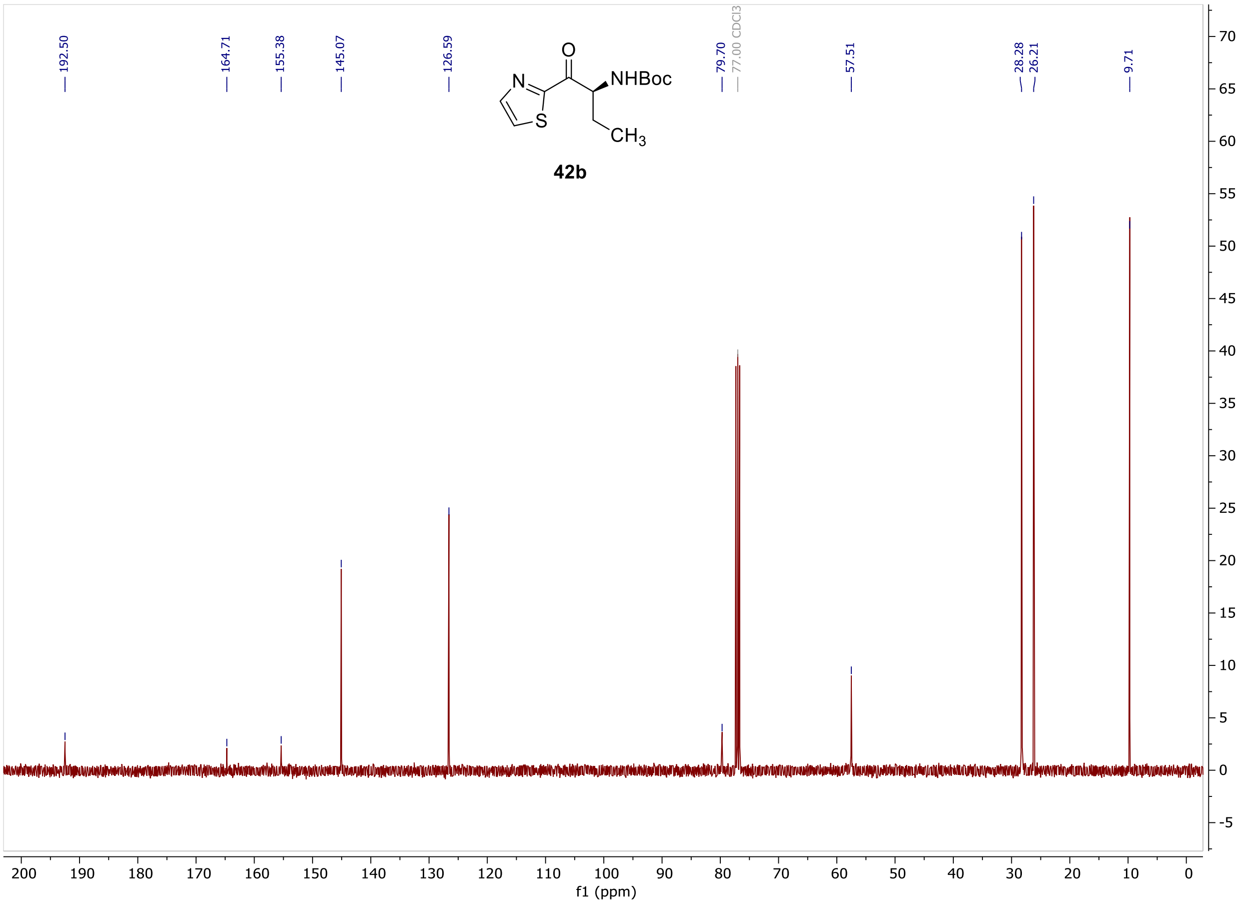

Compound **43a** (^{1}H NMR, 400 MHz):

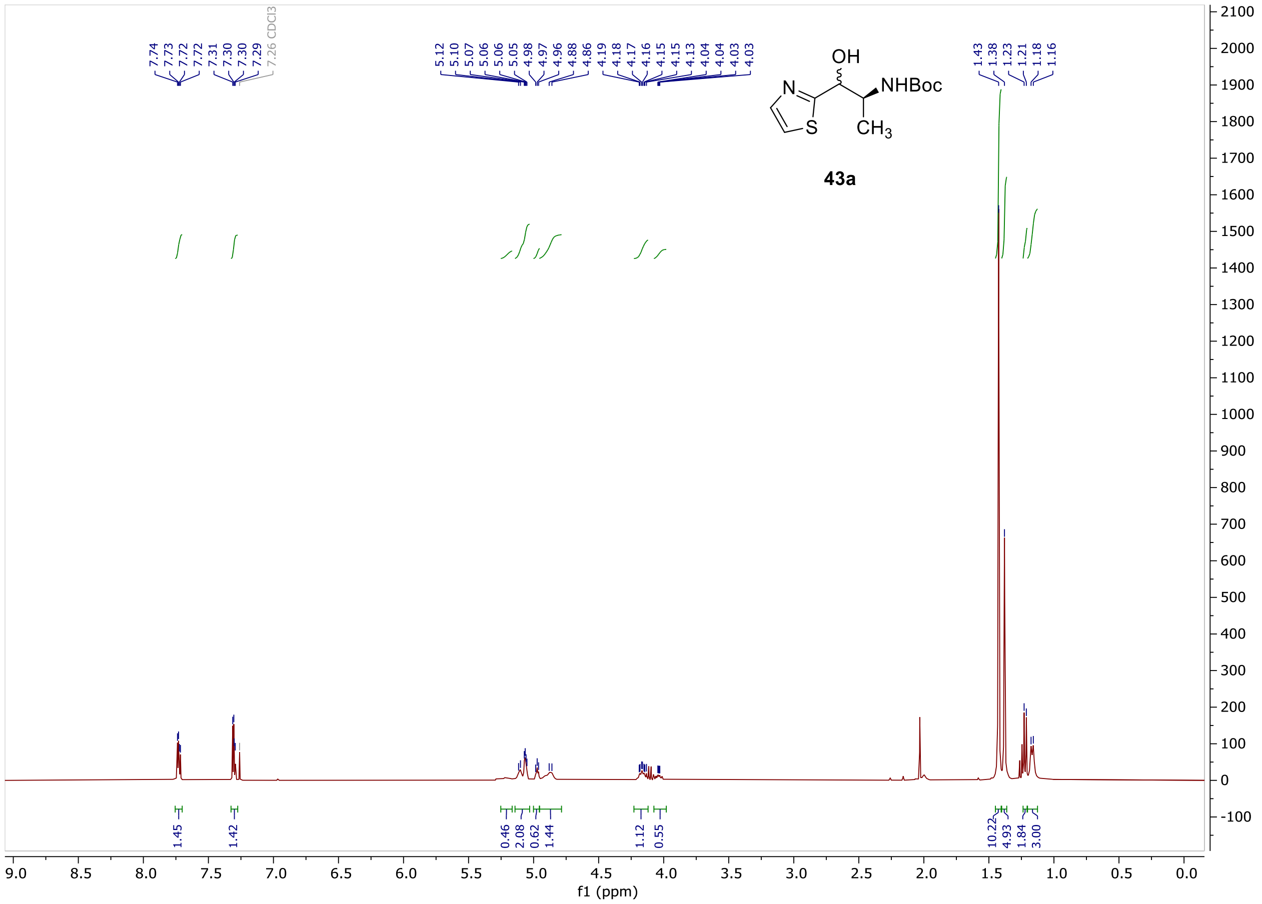

Compound **43a** (^{13}C NMR, 101 MHz):

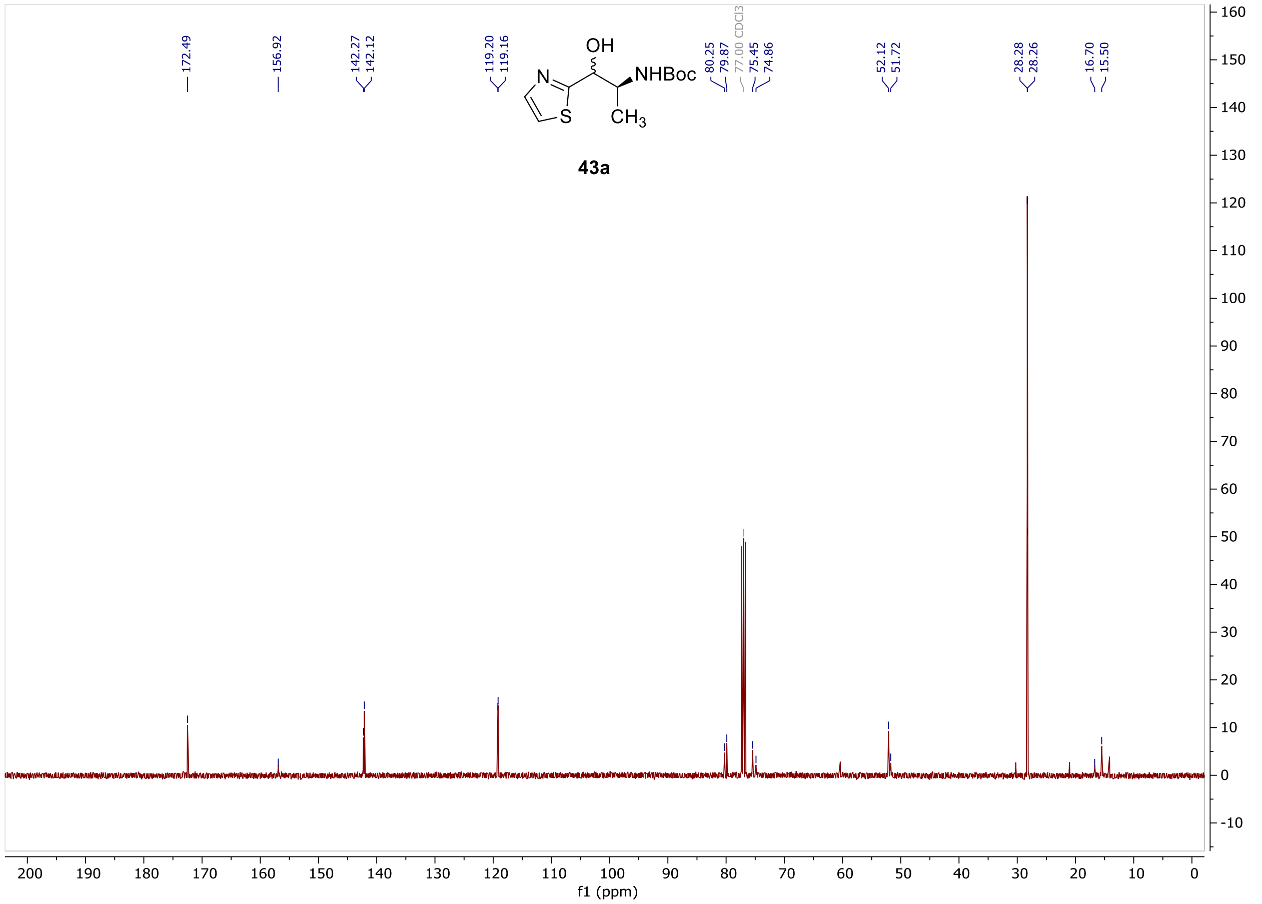

Compound **43b** (^{1}H NMR, 400 MHz):

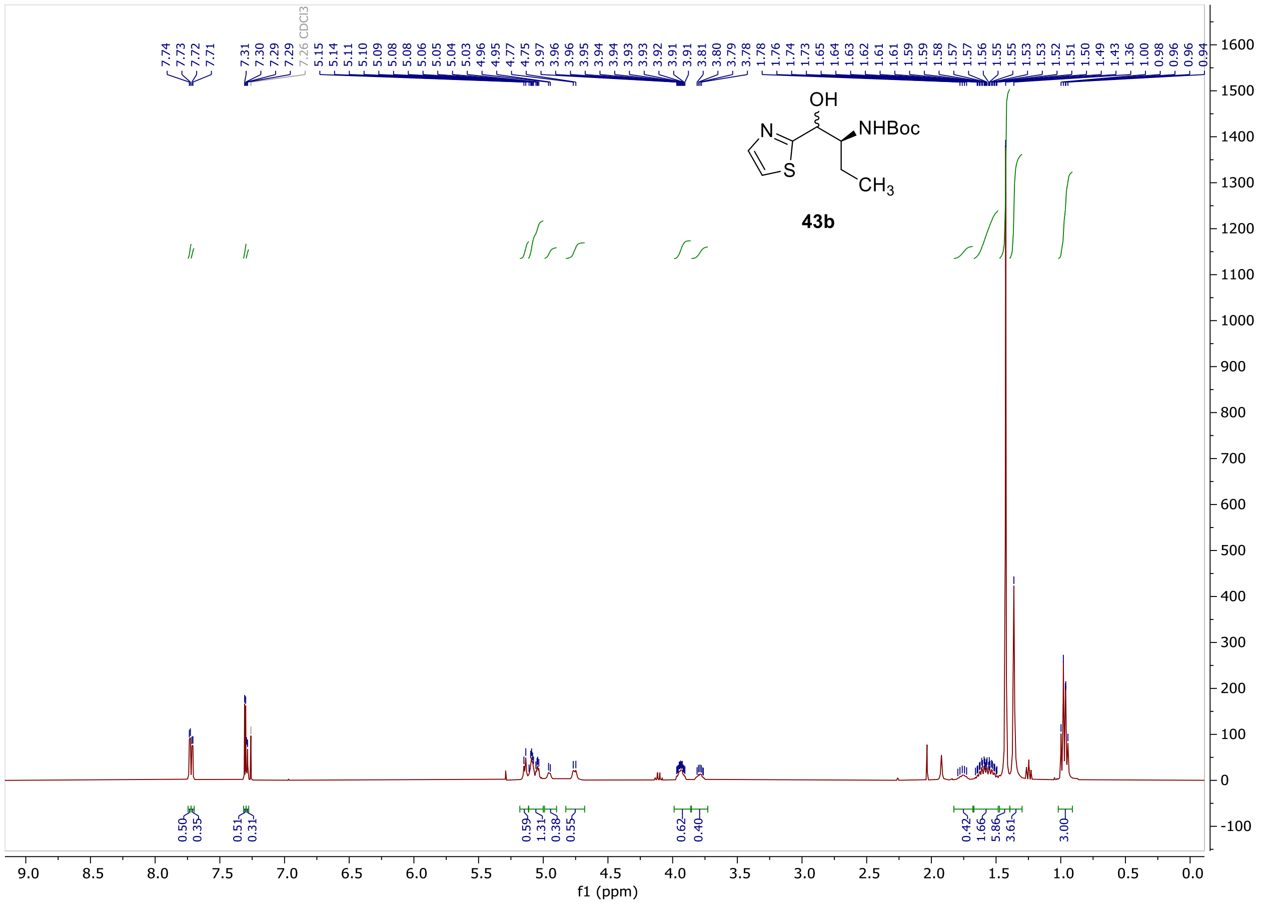

Compound **43b** (^{13}C NMR, 101 MHz):

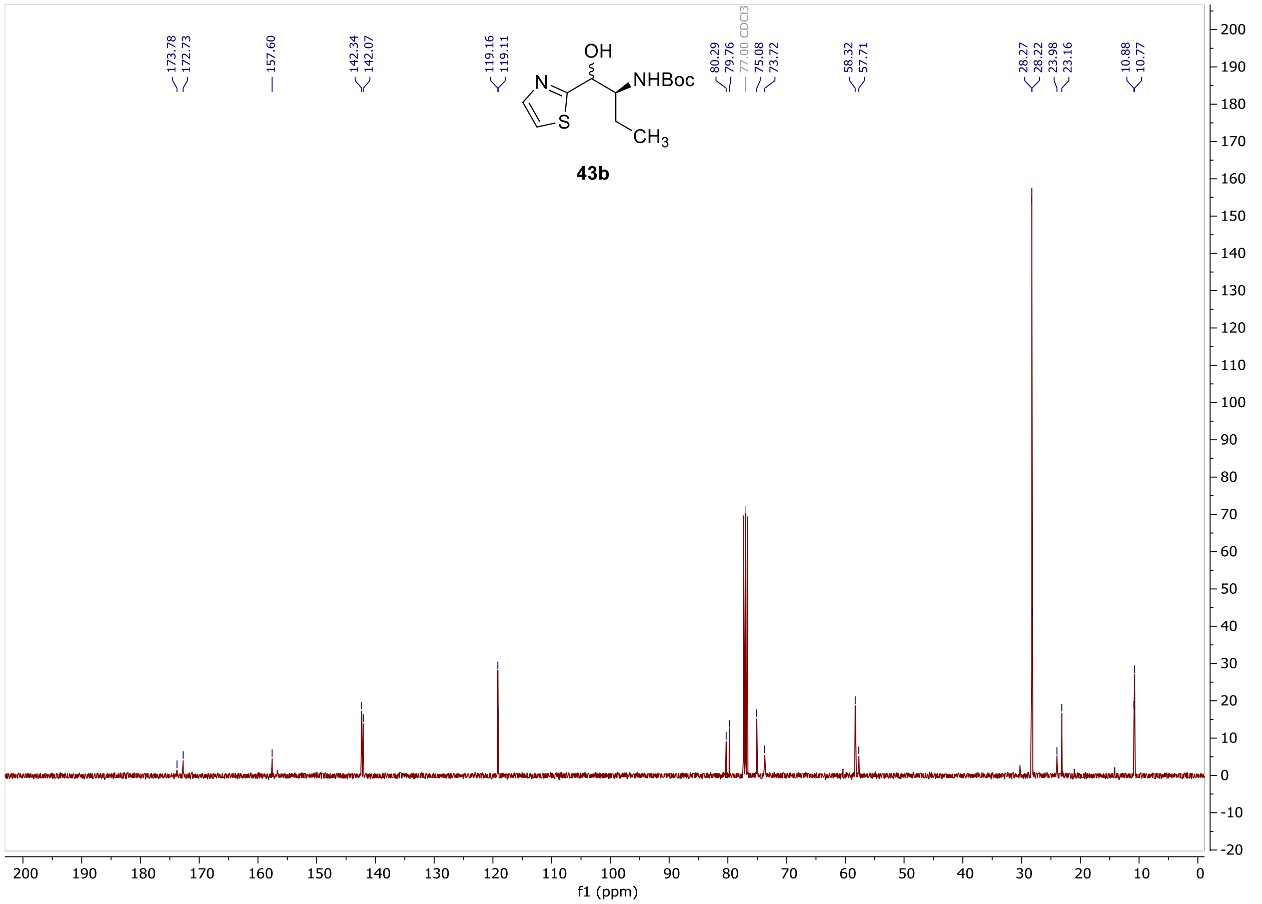

Compound **44a** – major diastereomer (^{1}H NMR, 400 MHz):

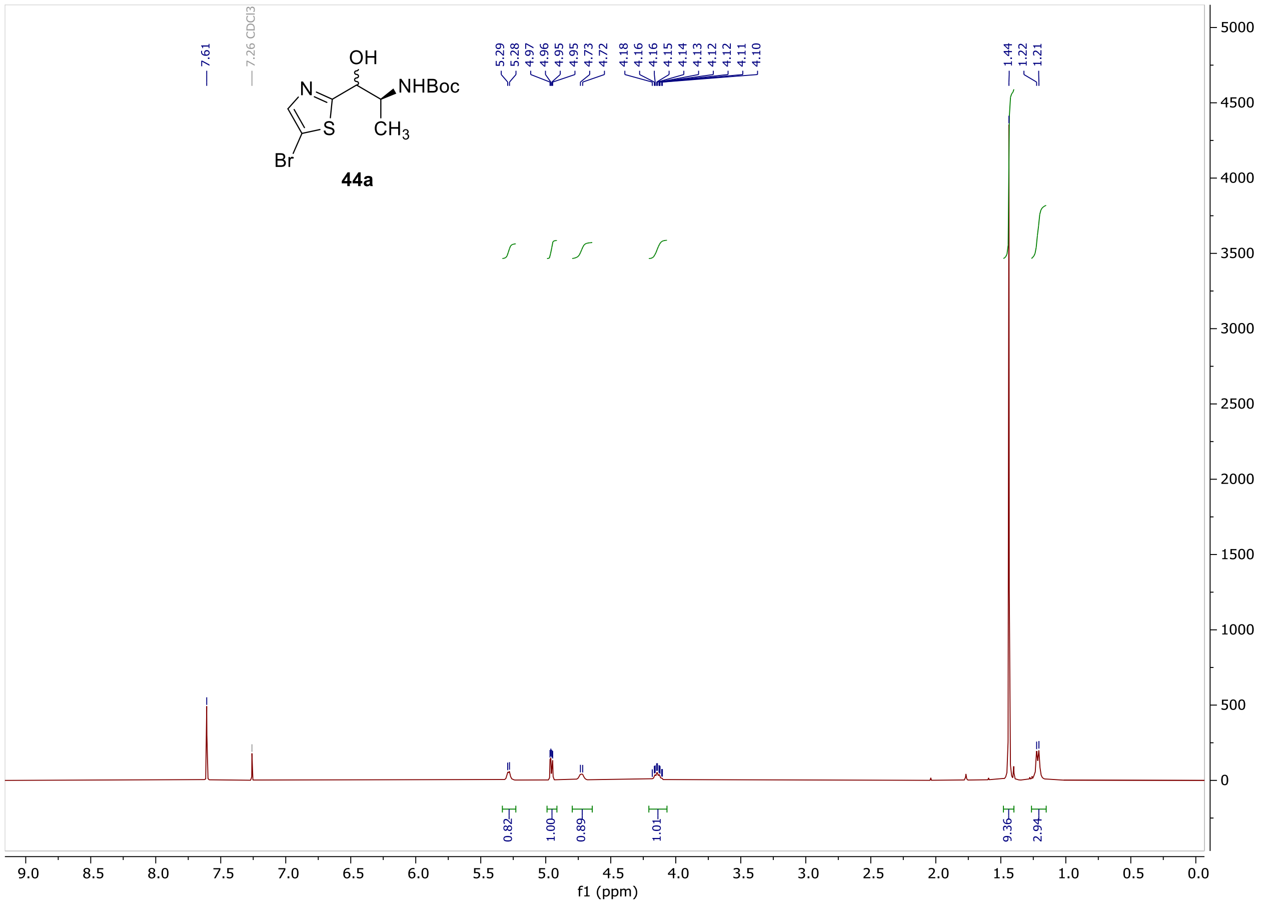

Compound **44a** – major diastereomer (^{13}C NMR, 101 MHz):

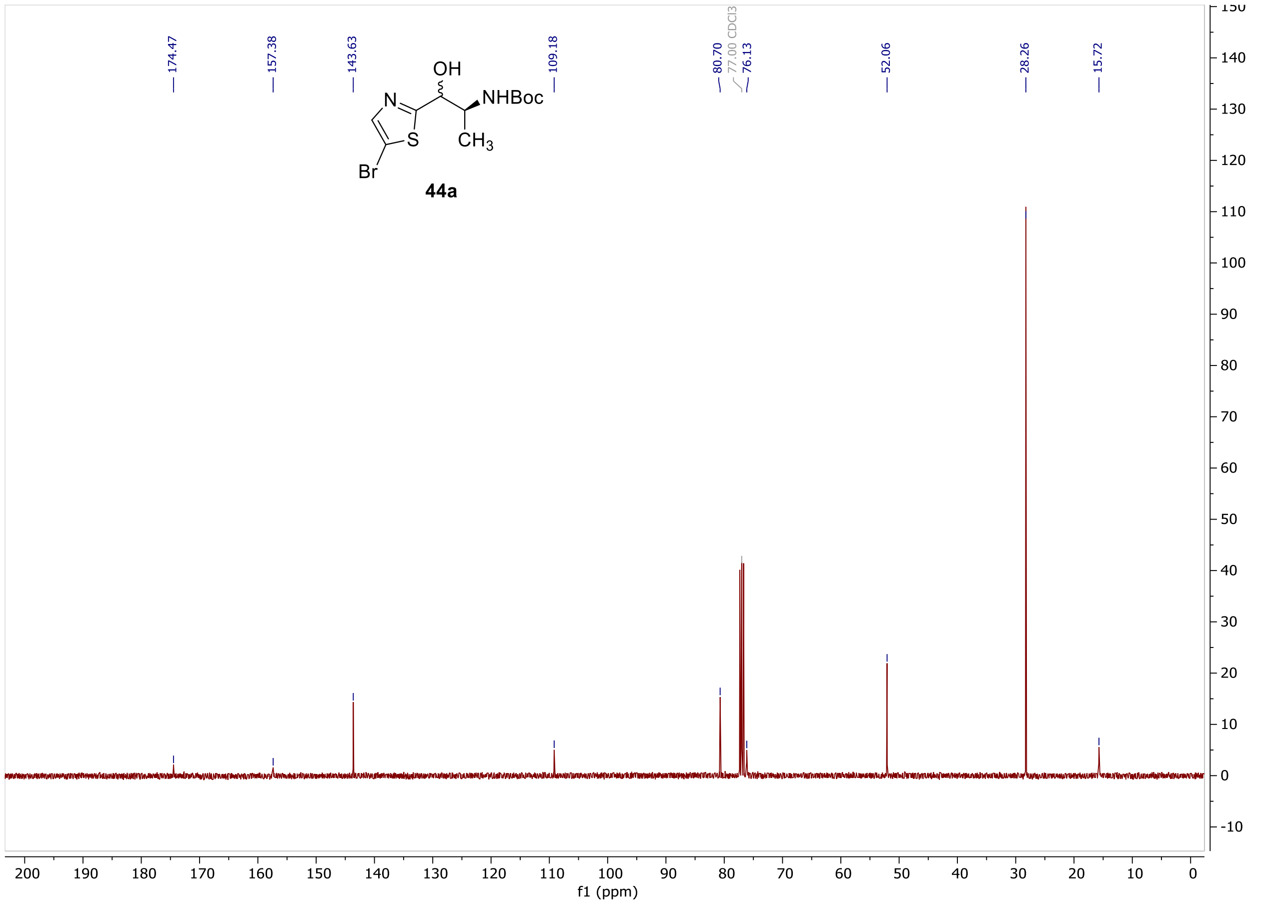

Compound **44a** – minor diastereomer (^{1}H NMR, 400 MHz):

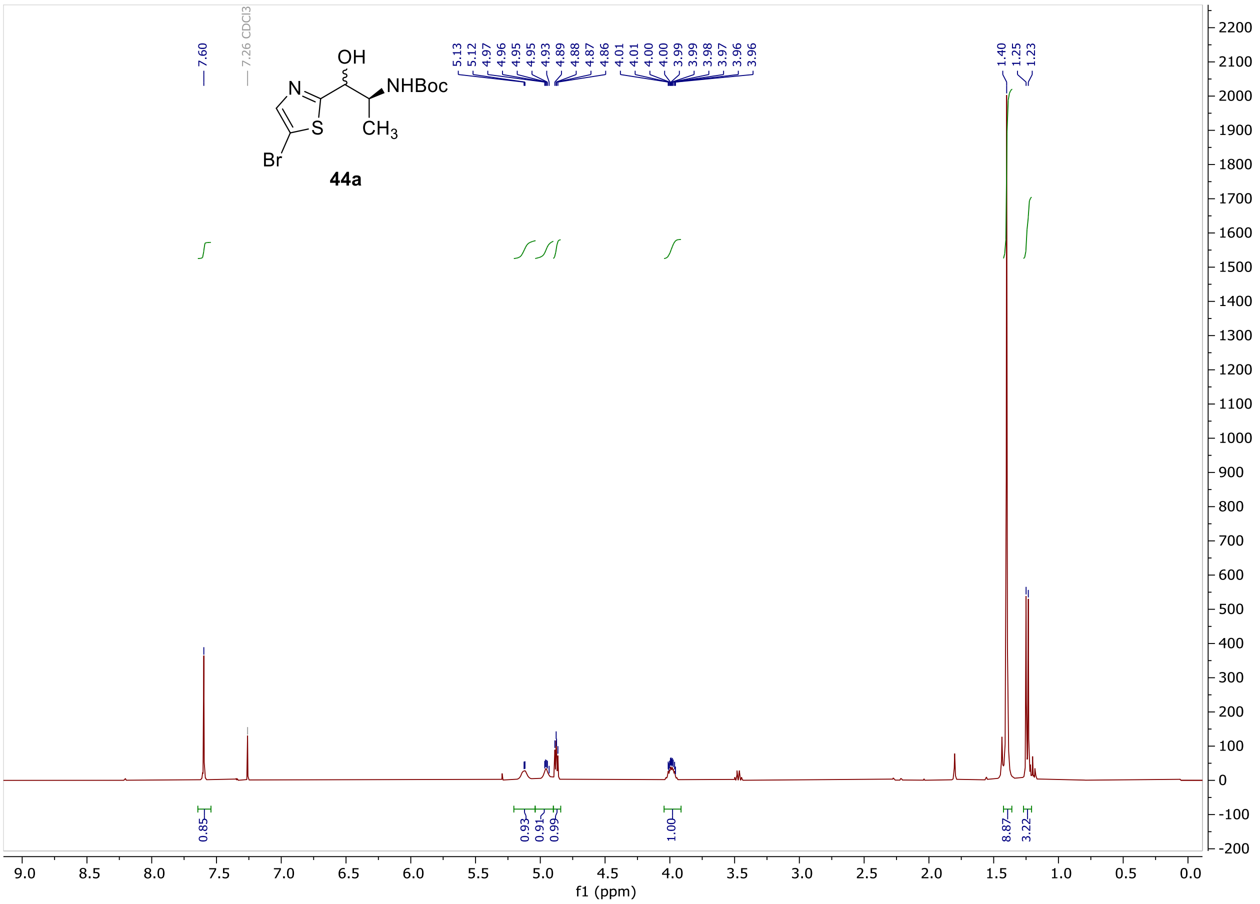

Compound **44a** – minor diastereomer (^{13}C NMR, 101 MHz):

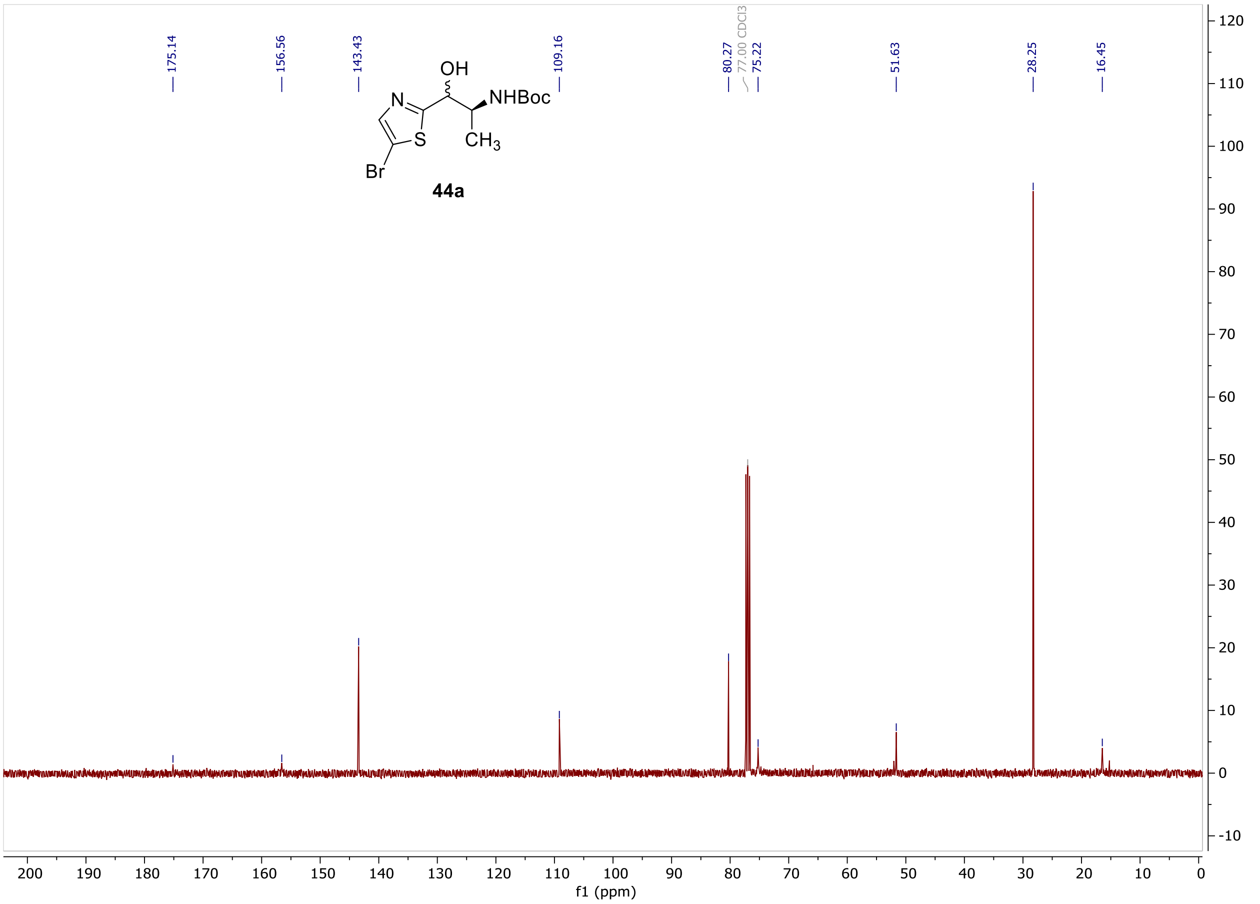

Compound **44b** – major diastereomer (^{1}H NMR, 400 MHz):

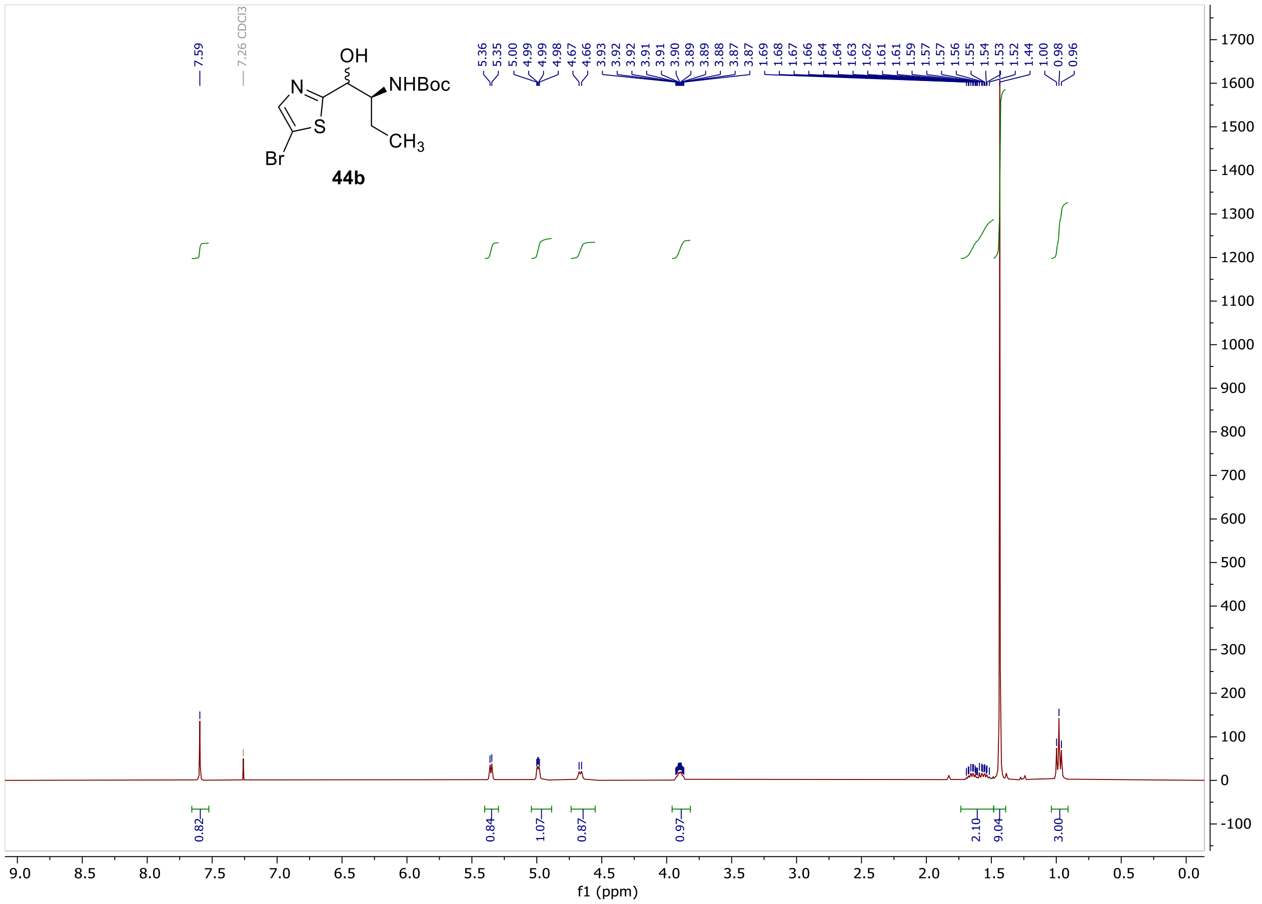

Compound **44b** – major diastereomer (^{13}C NMR, 101 MHz):

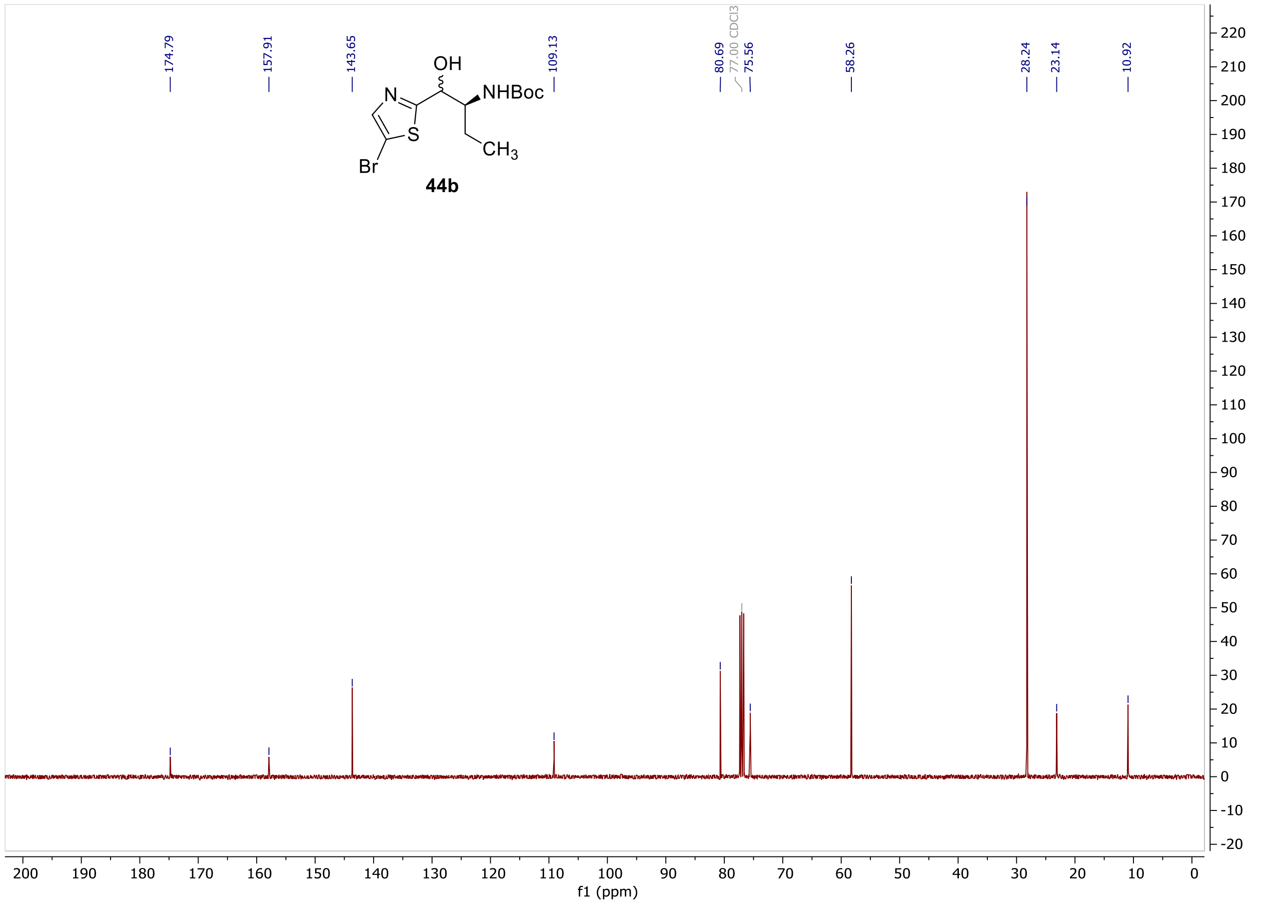

Compound **44b** – minor diastereomer (^{1}H NMR, 400 MHz):

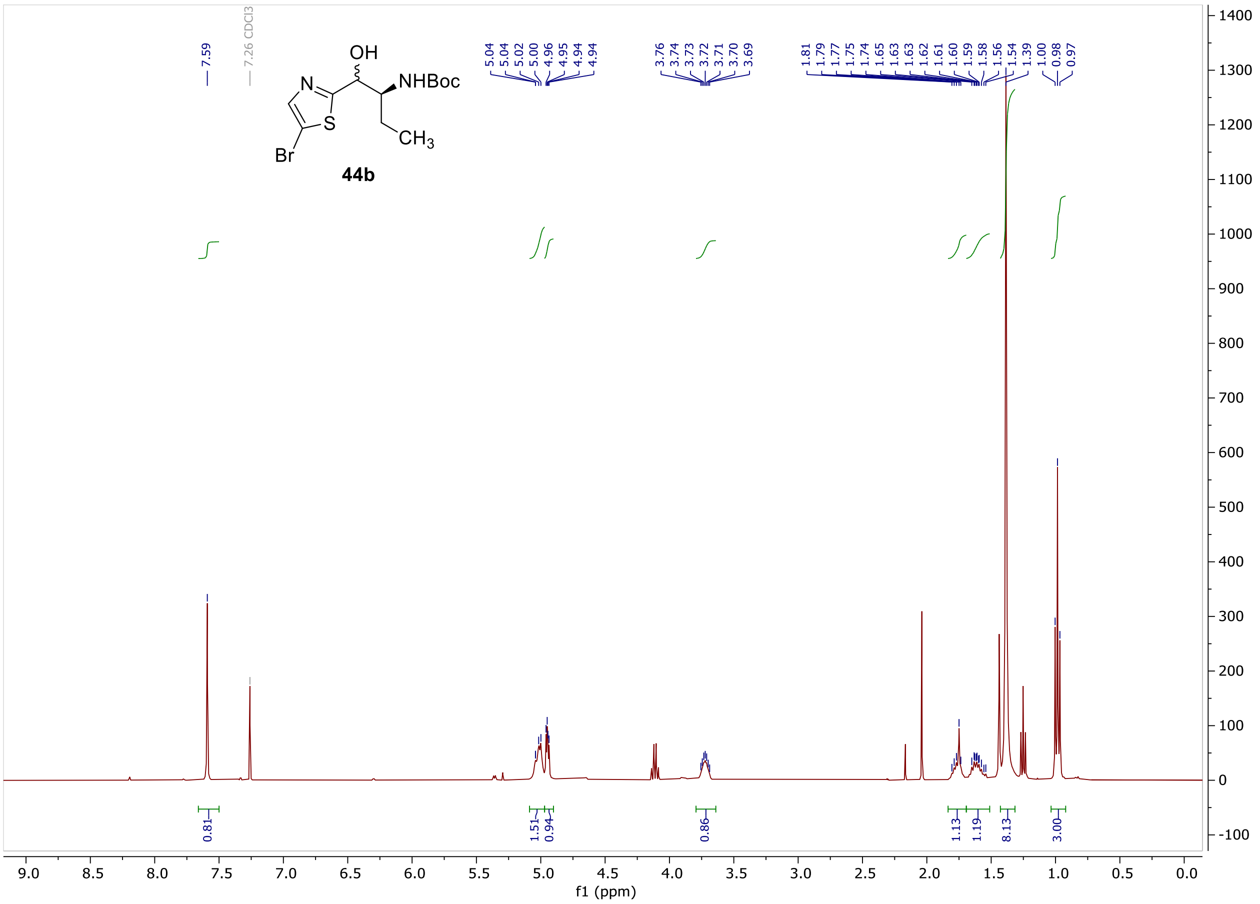

Compound **44b** – minor diastereomer (^{13}C NMR, 101 MHz):

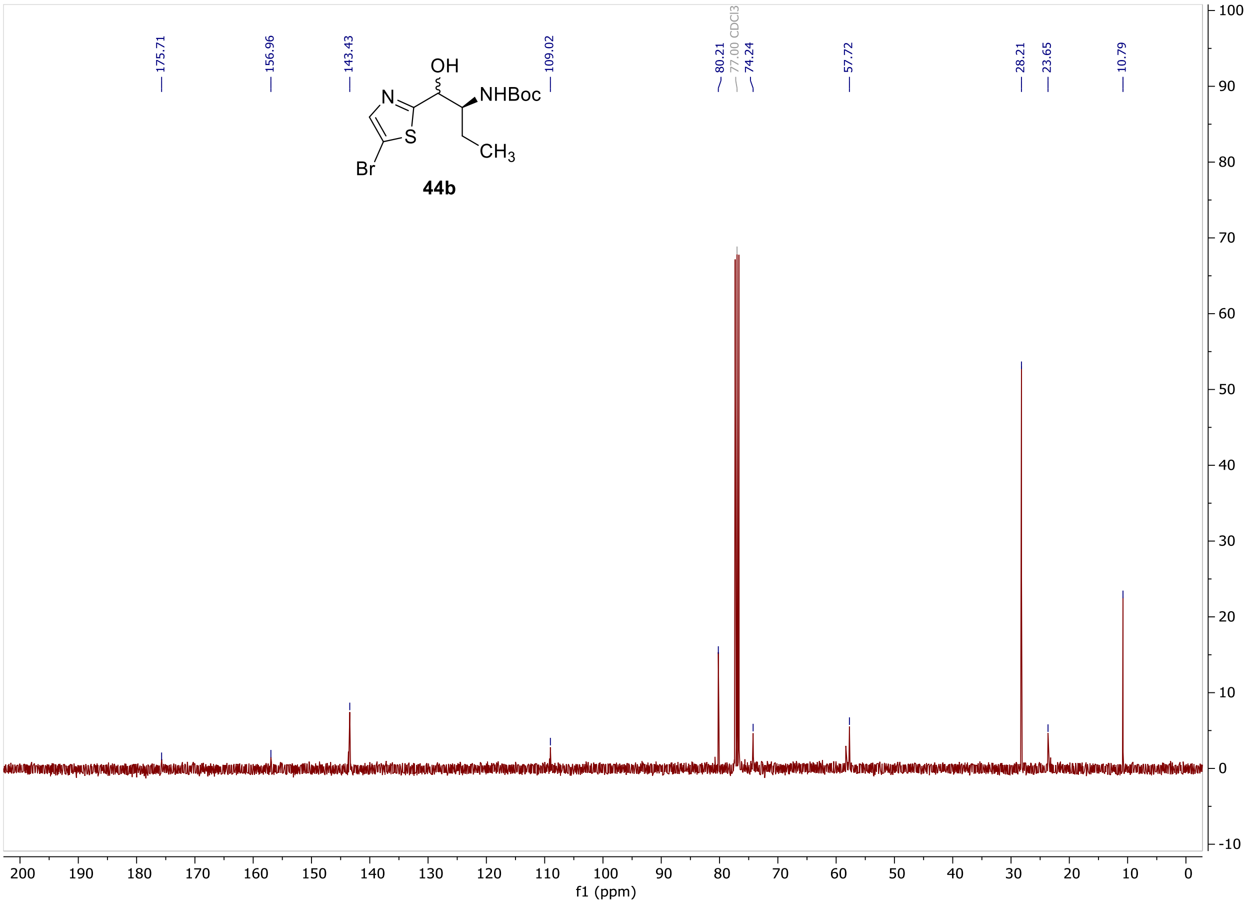

Compound **40a** (^{1}H NMR, 400 MHz):

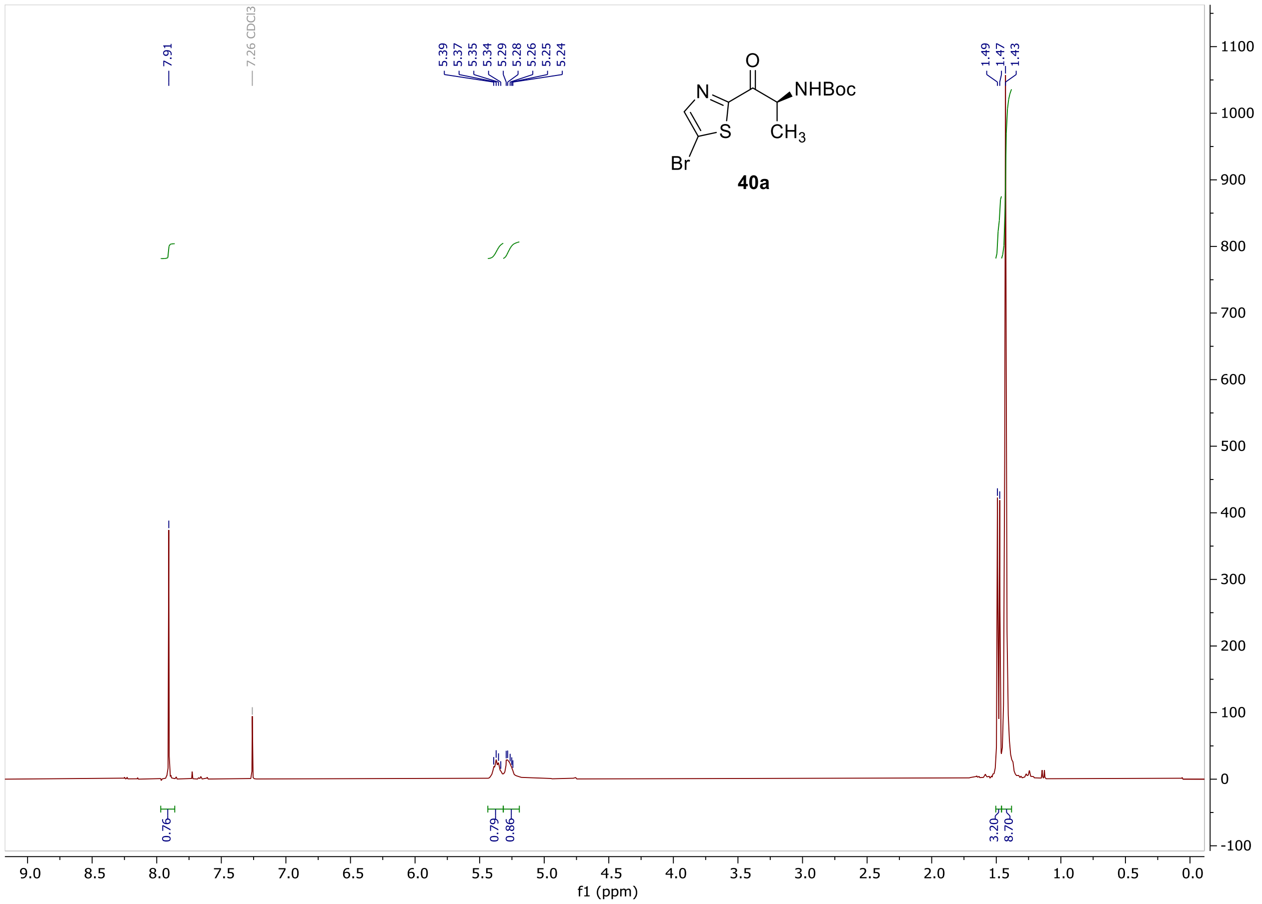

Compound **40a** (^{13}C NMR, 101 MHz):

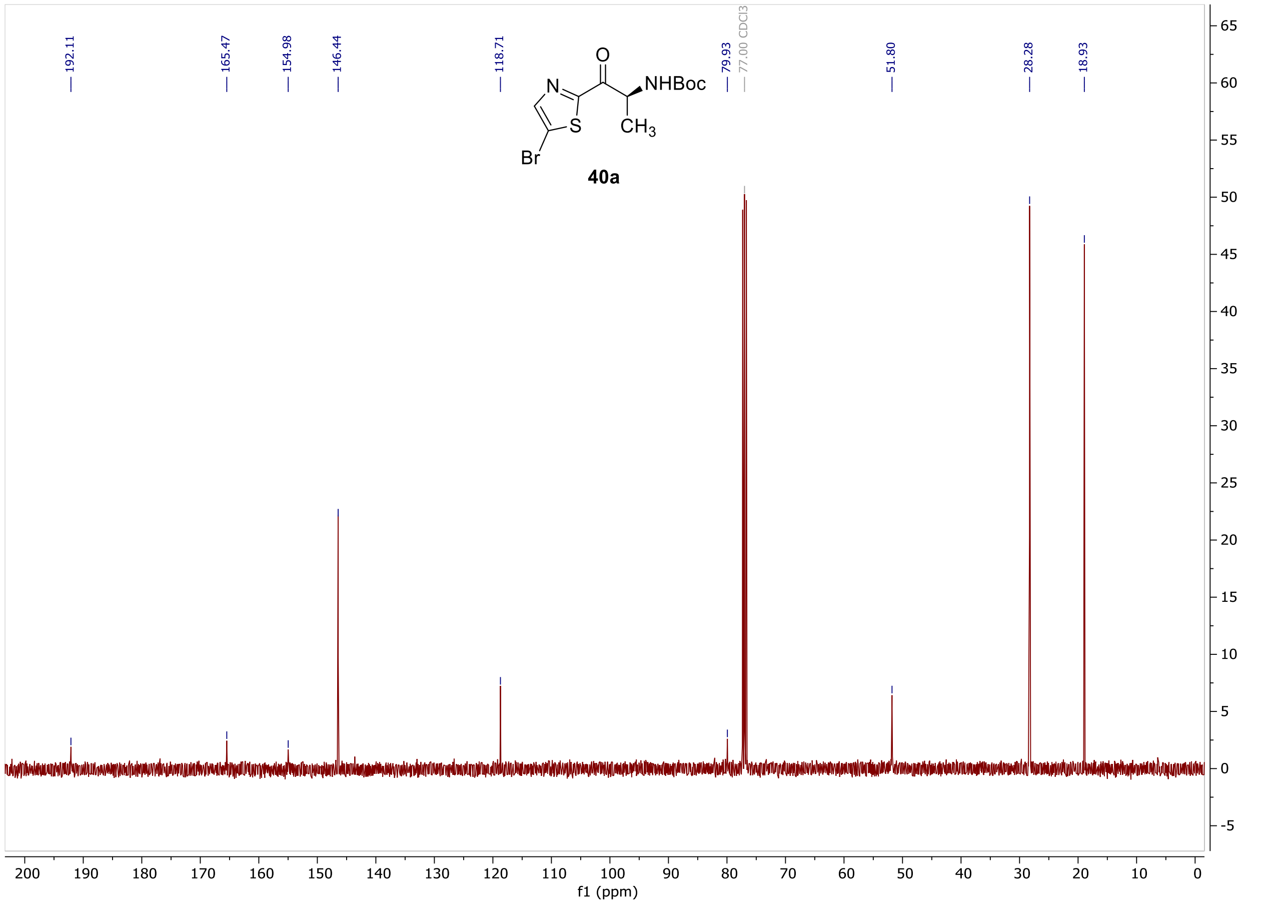

Compound **40b** (^{1}H NMR, 400 MHz):

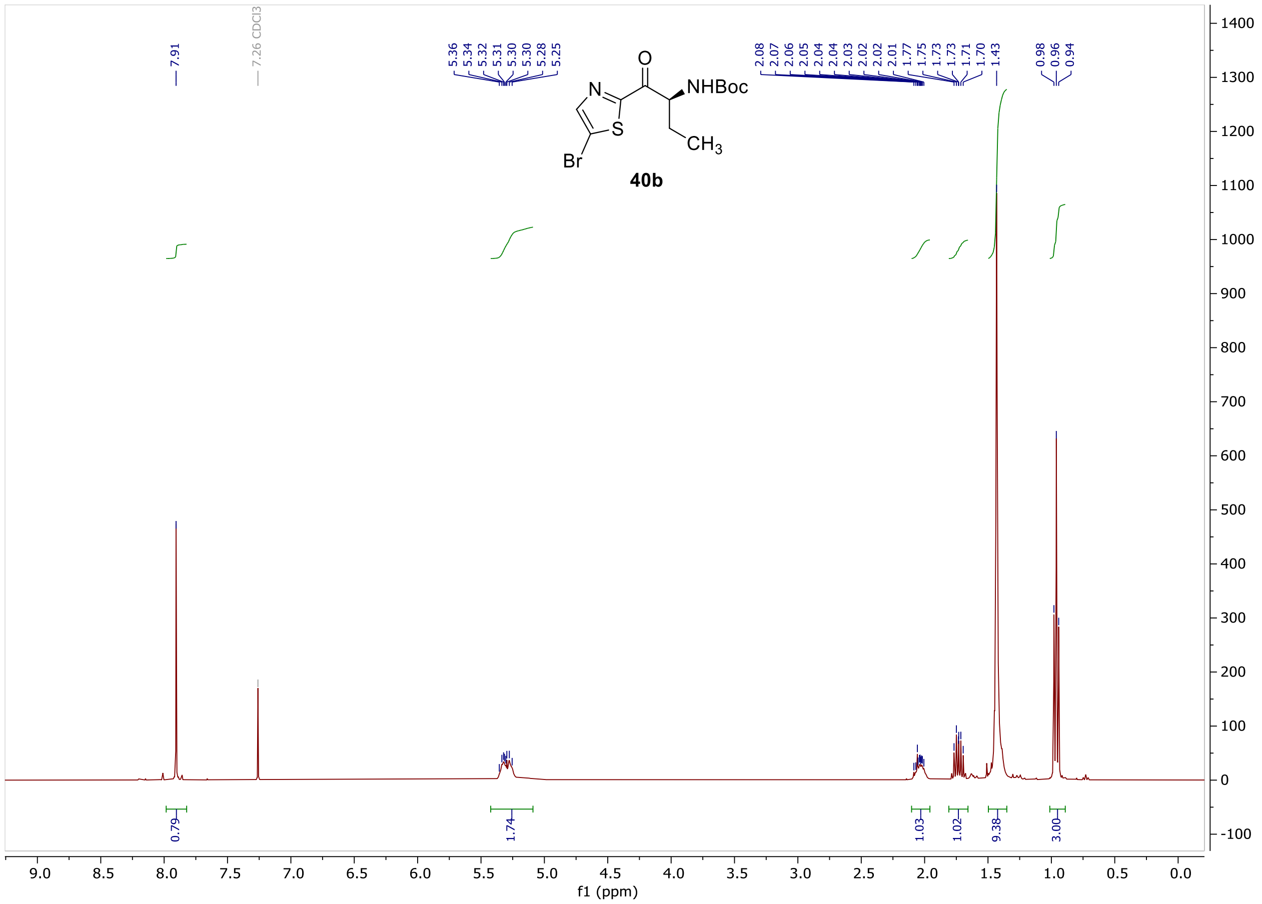

Compound **40b** (^{13}C NMR, 101 MHz):

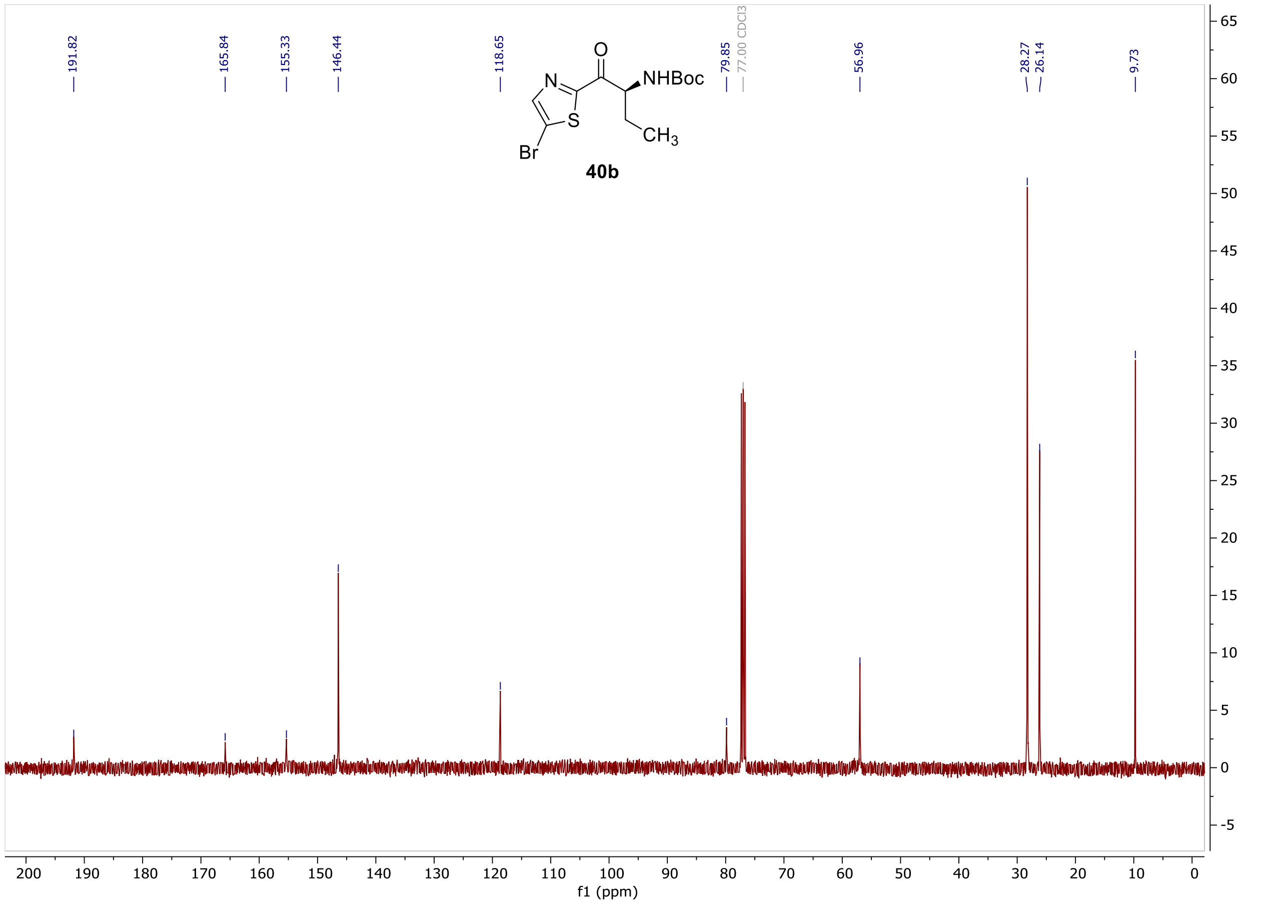

Compound **45a** (^{1}H NMR, 400 MHz):

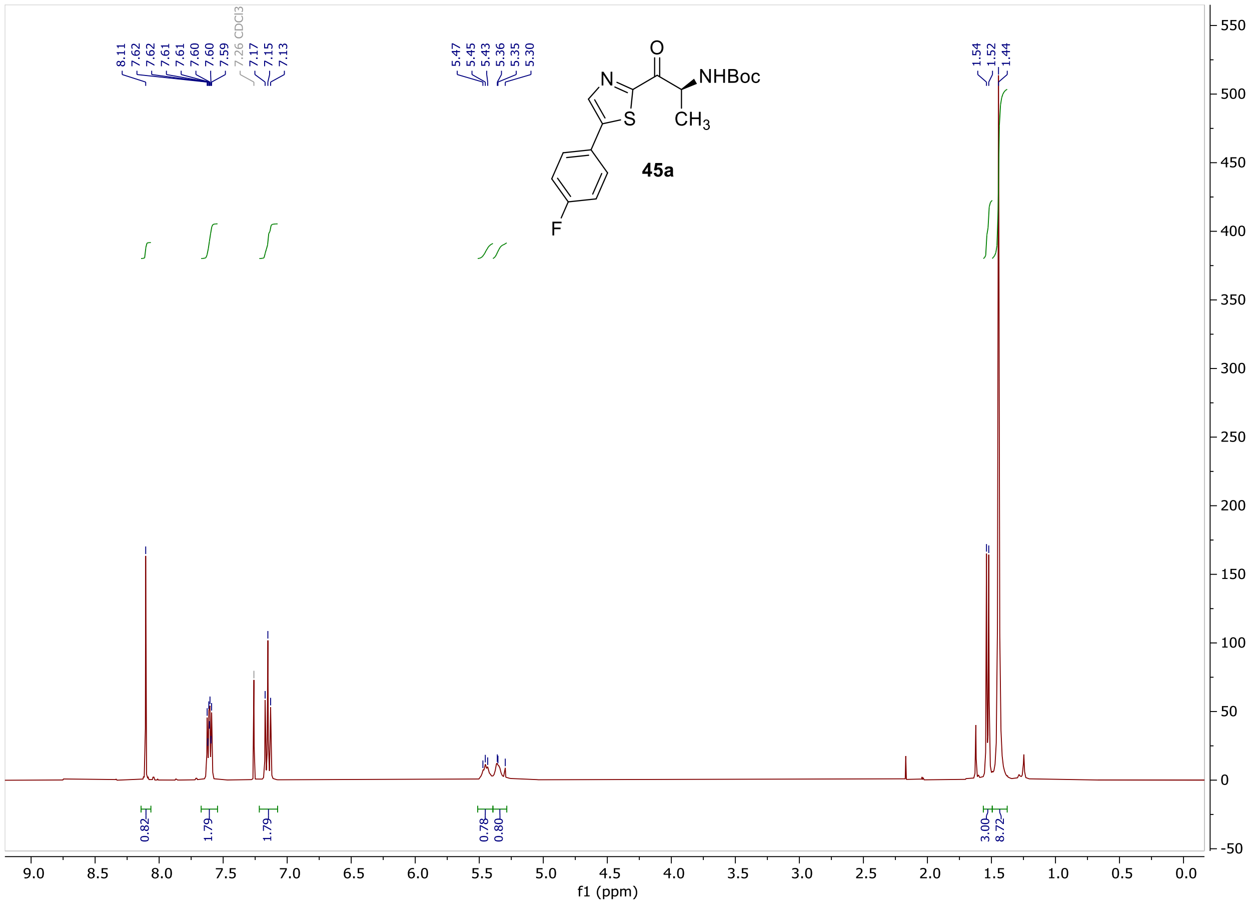

Compound **45a** (^{13}C NMR, 101 MHz):

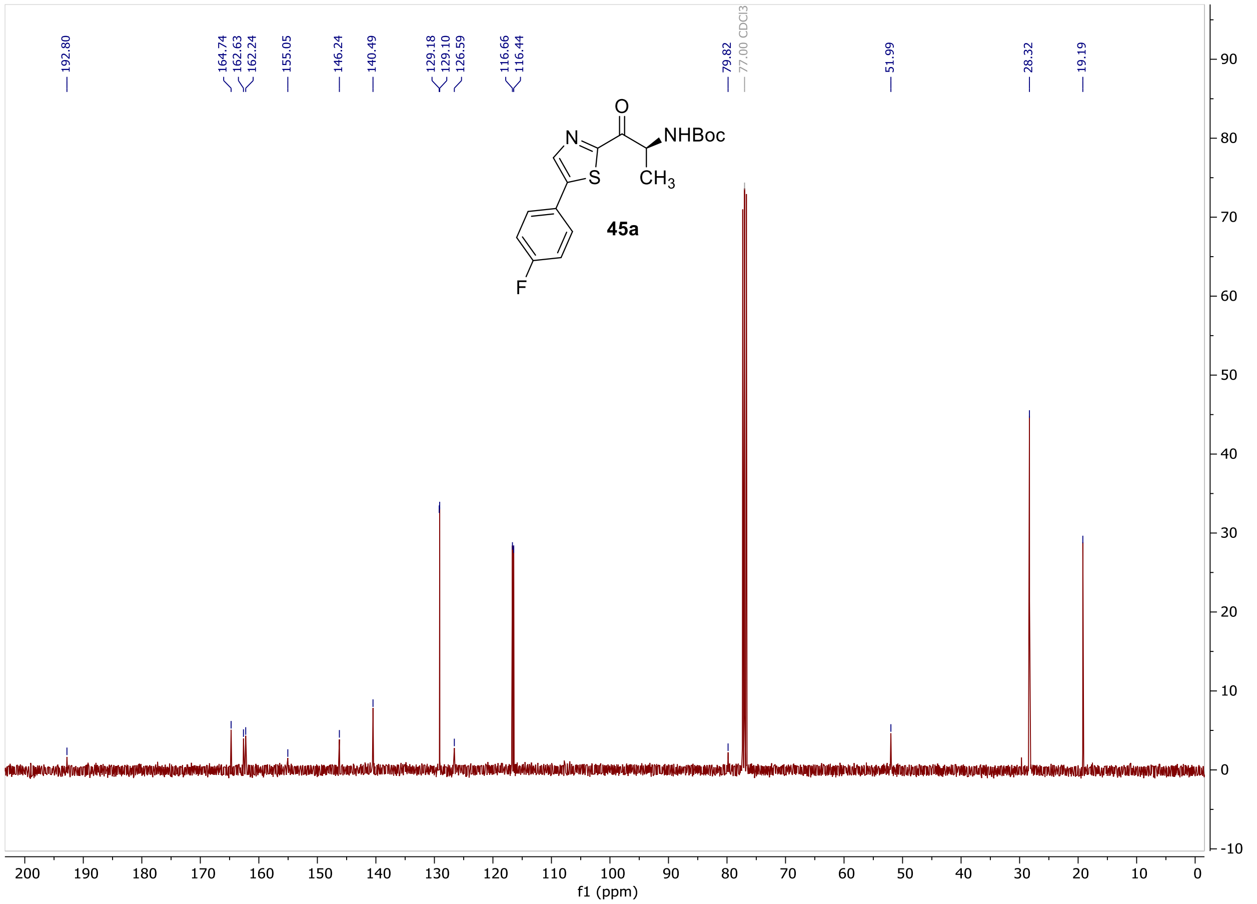

Compound **45a** (^{19}F NMR, 282 MHz):

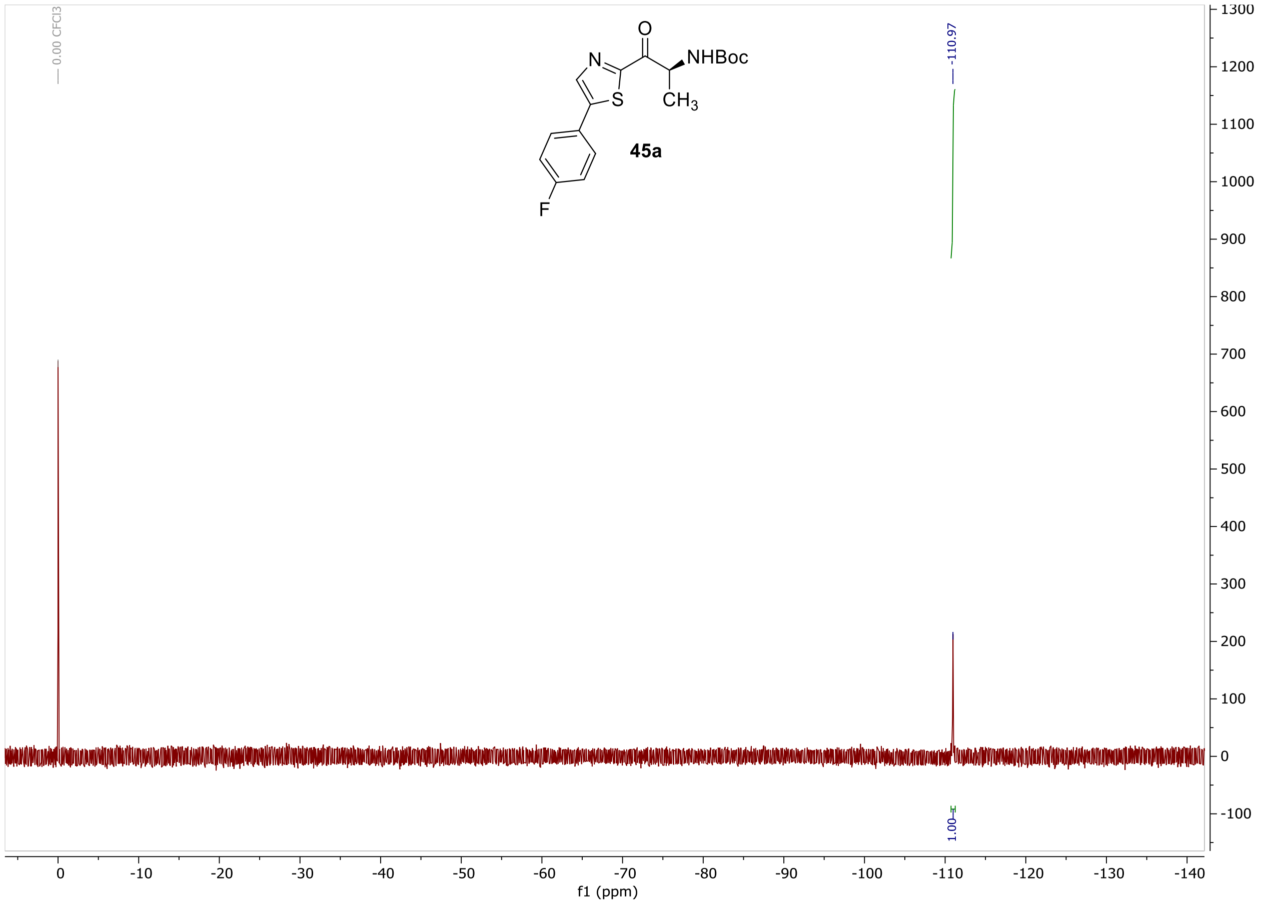

Compound **45b** (^{1}H NMR, 400 MHz):

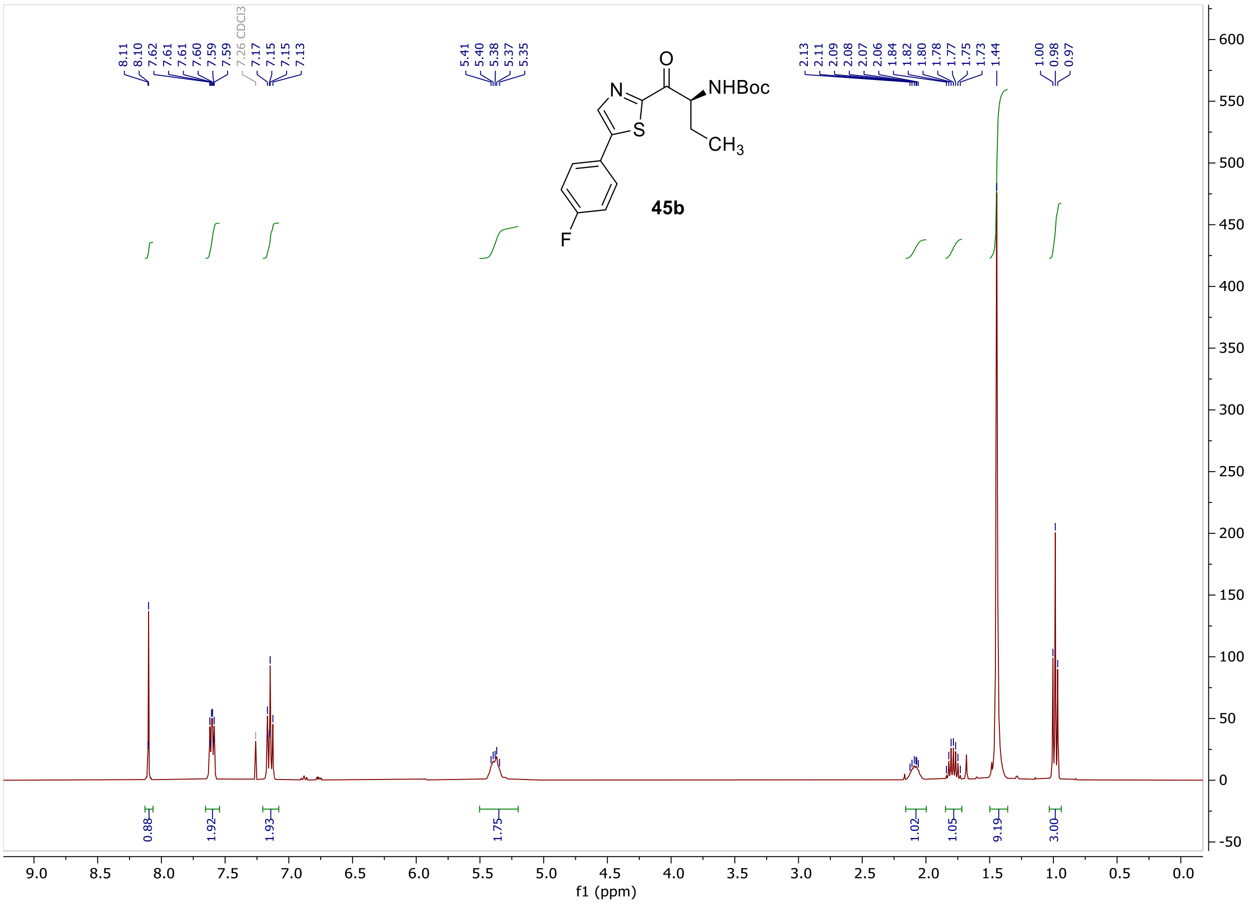

Compound **45b** (^{13}C NMR, 101 MHz):

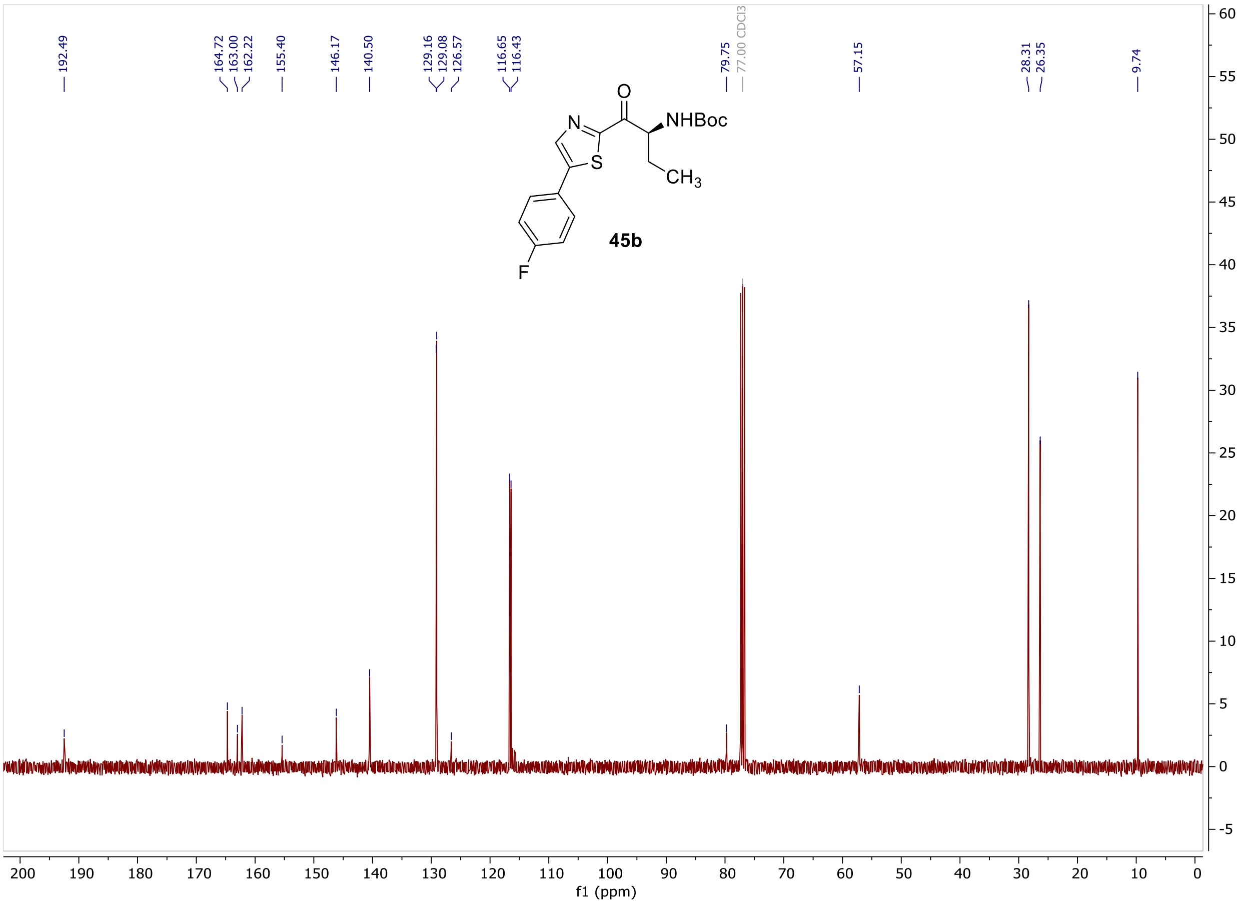

Compound **45b** (^{19}F NMR, 282 MHz):

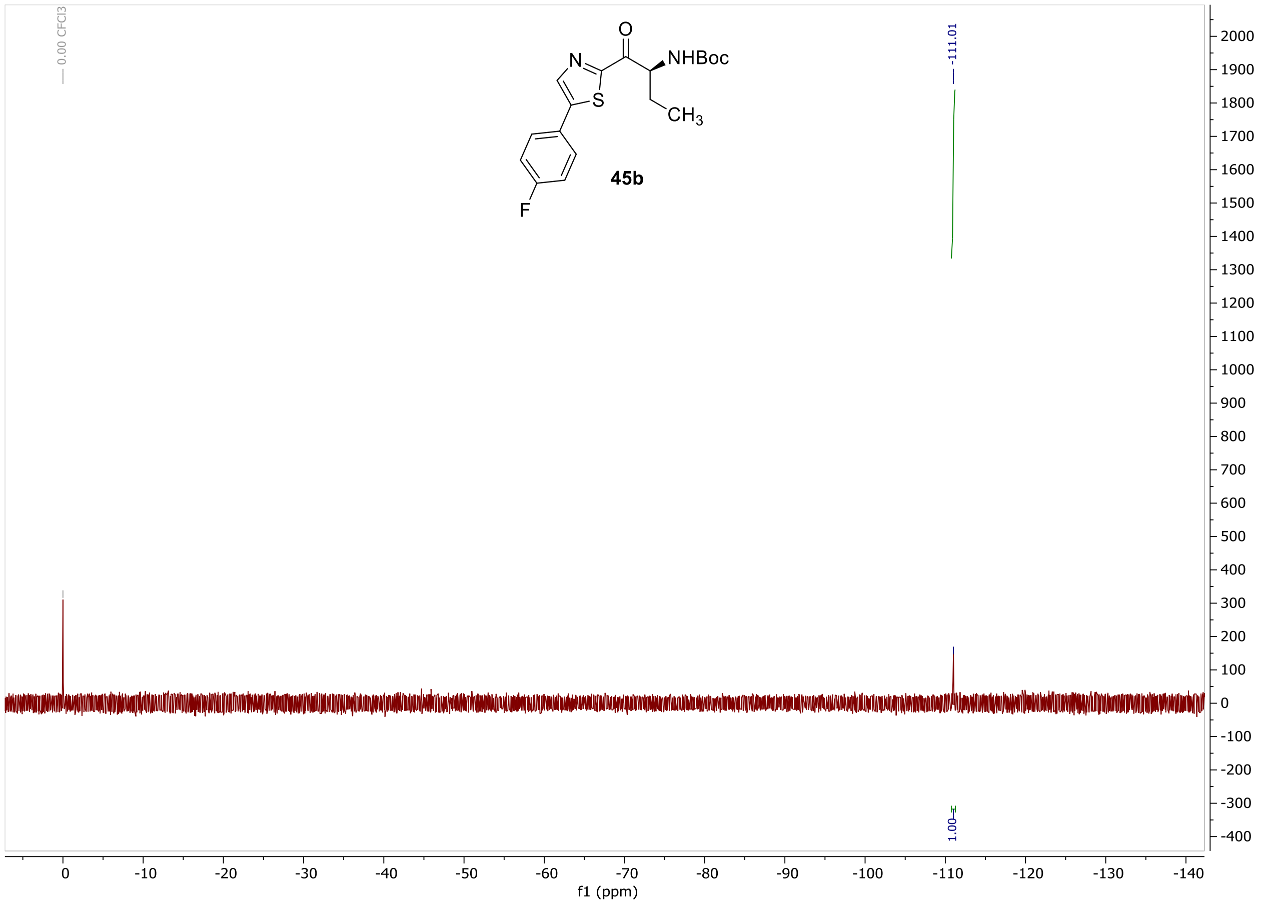

Compound **46a** (^{1}H NMR, 400 MHz):

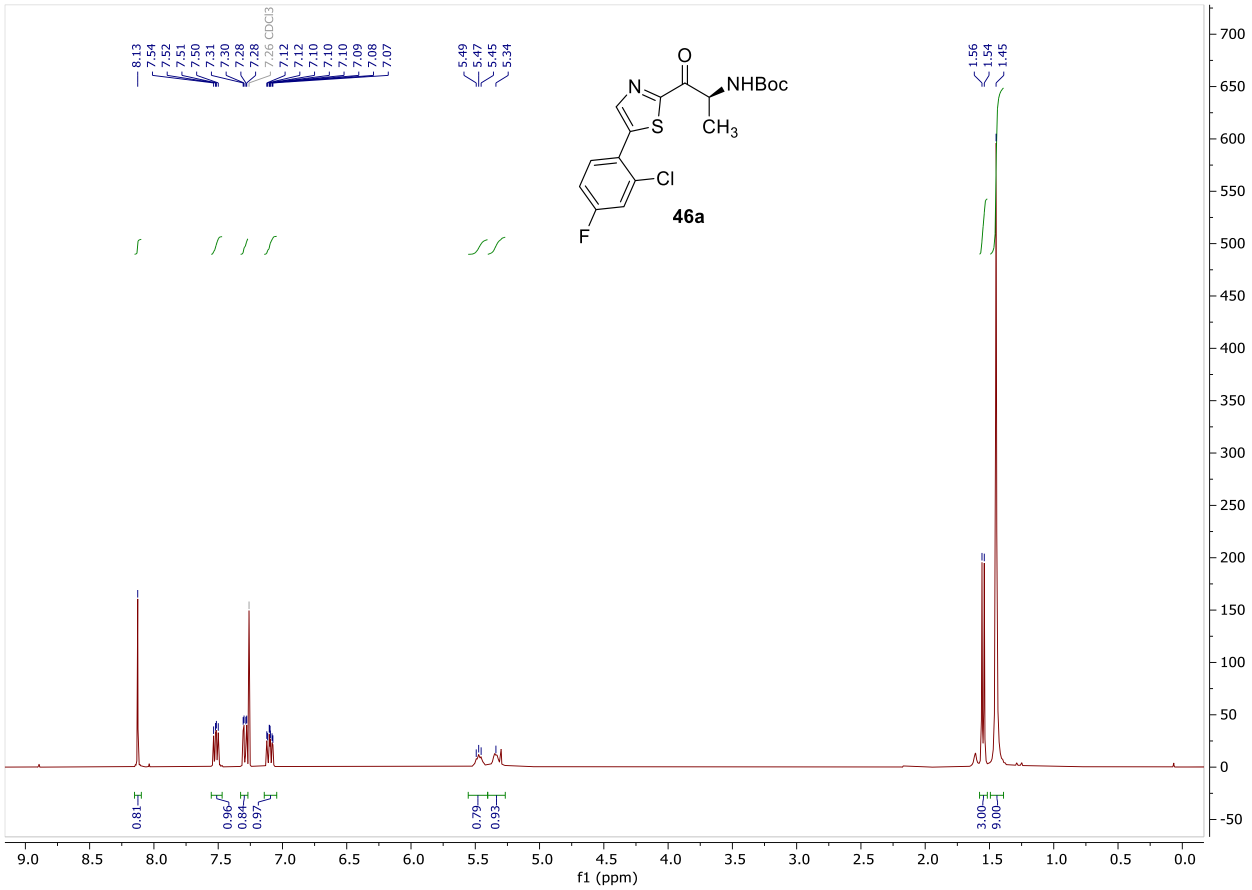

Compound **46a** (^{13}C NMR, 101 MHz):

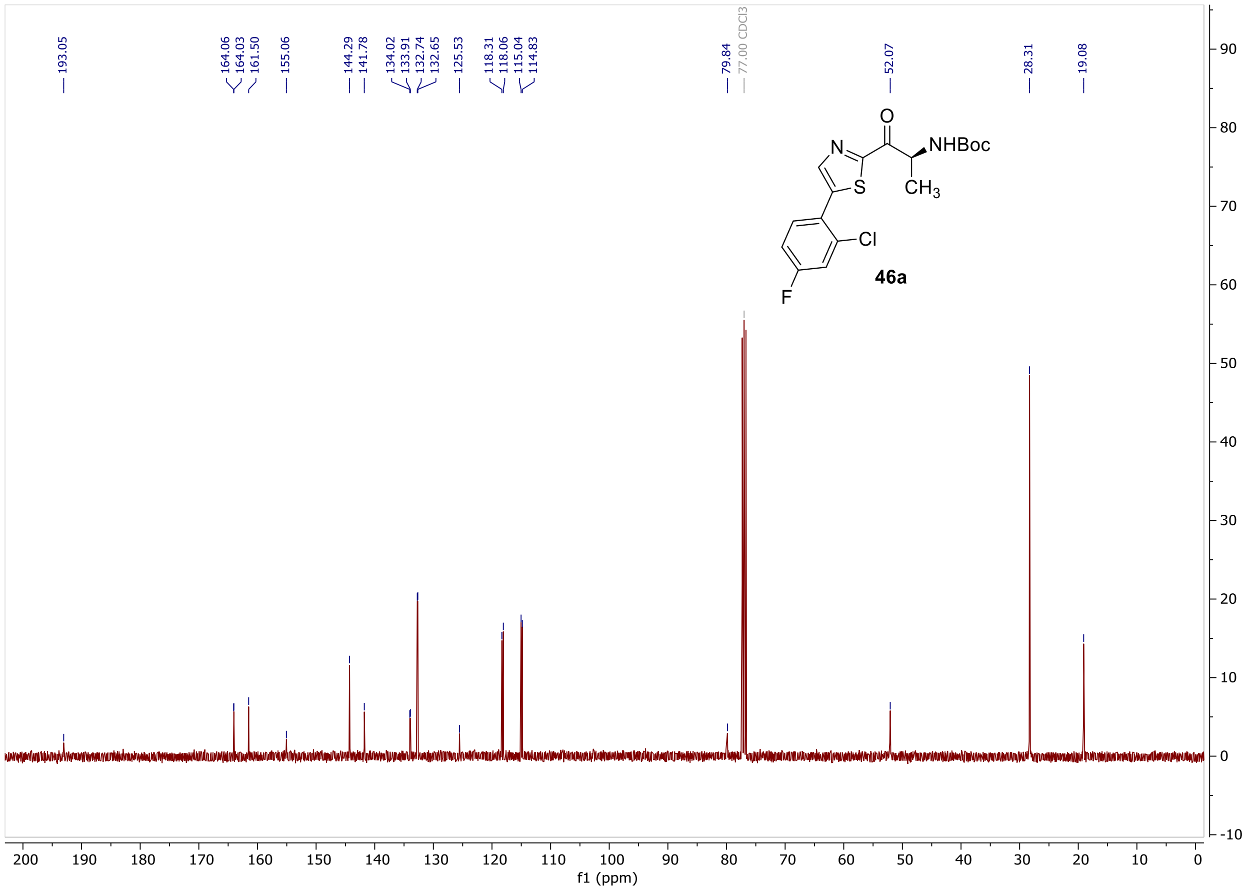

Compound **46a** (^{19}F NMR, 282 MHz):

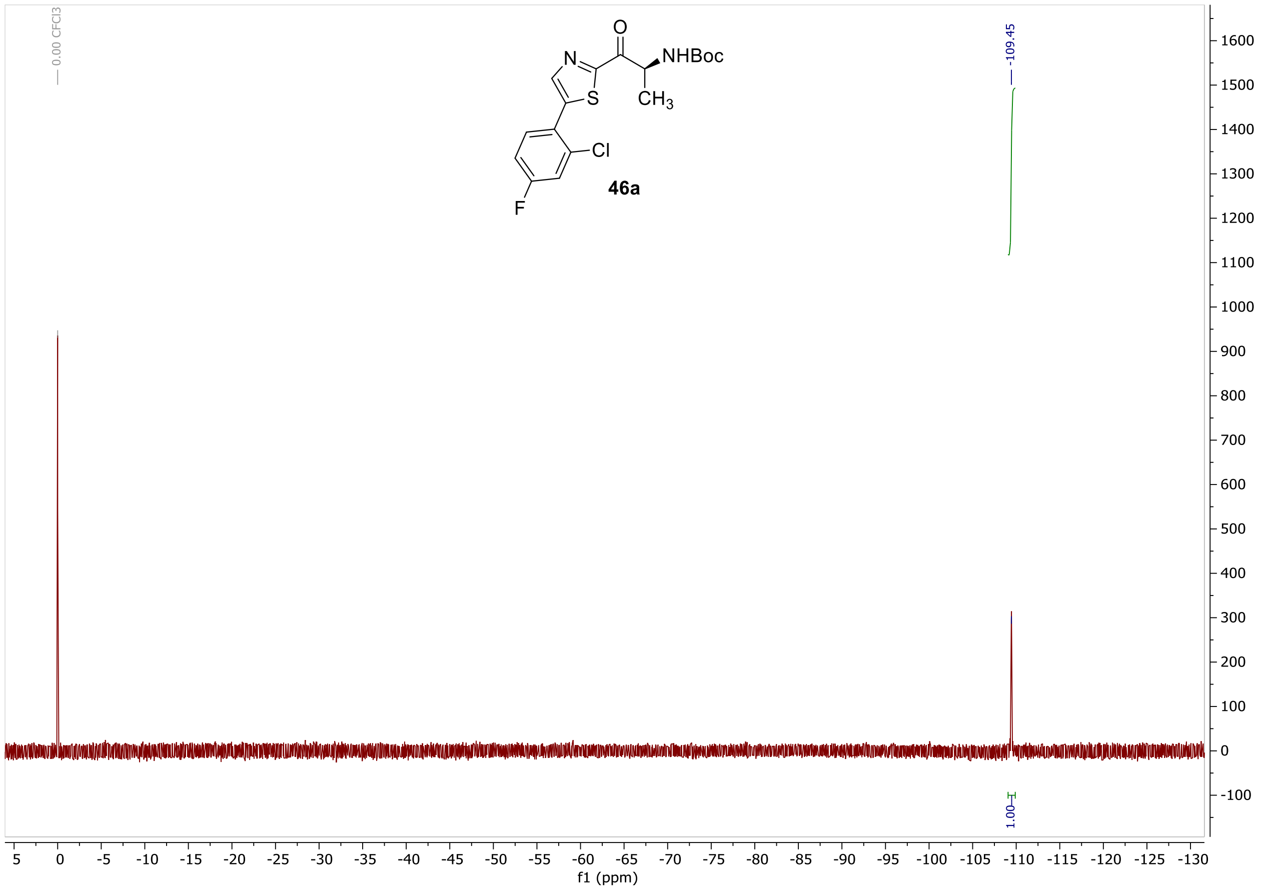

Compound **46b** (^{1}H NMR, 400 MHz):

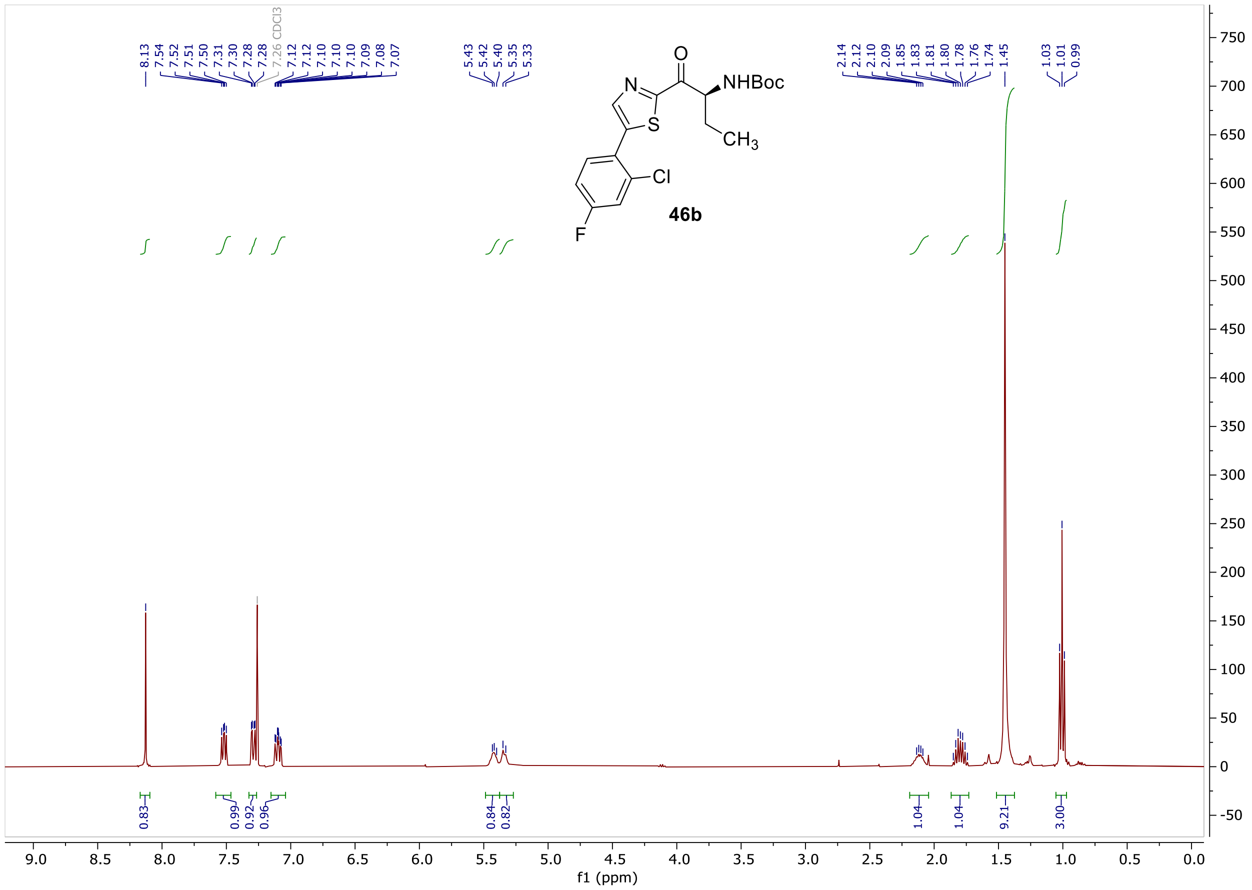

Compound **46b** (^{13}C NMR, 101 MHz):

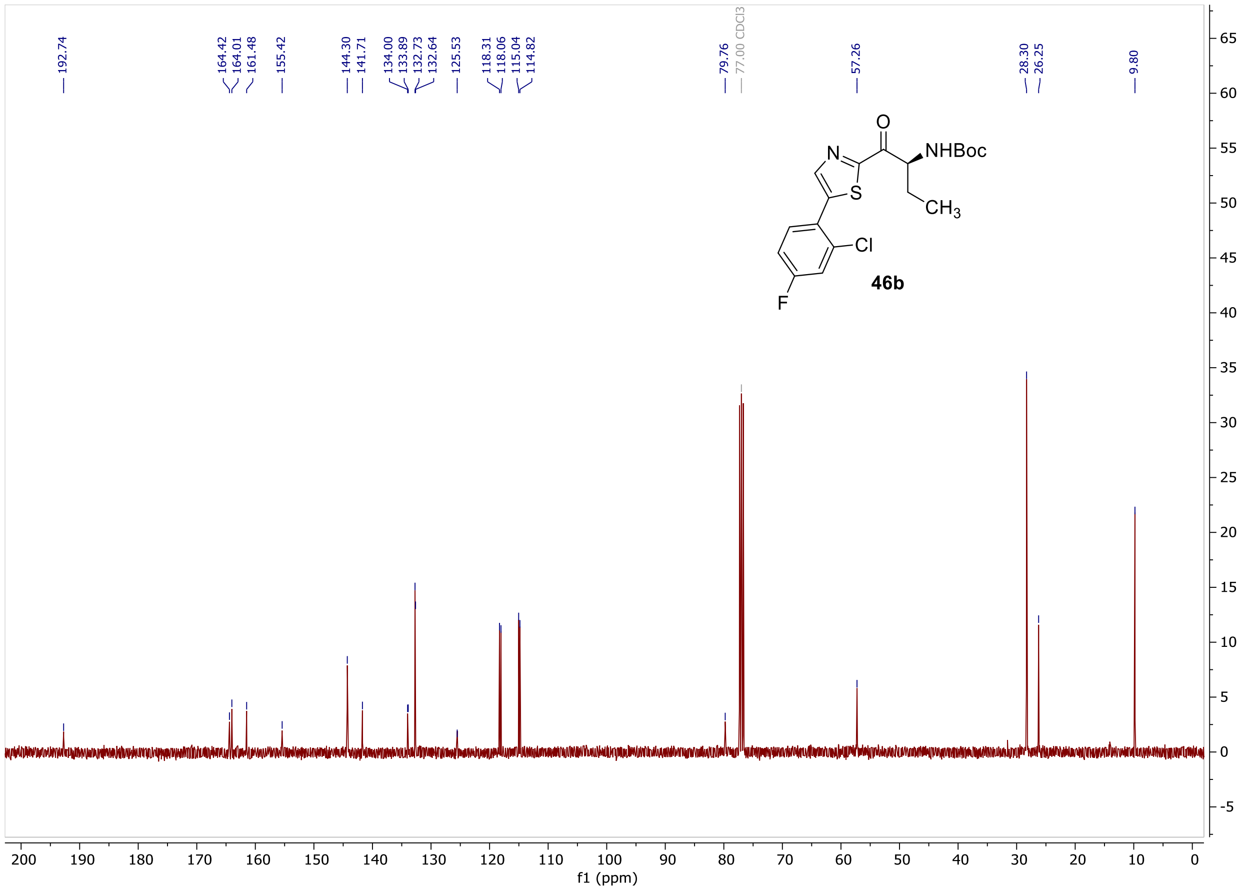

Compound **46b** (^{19}F NMR, 282 MHz):

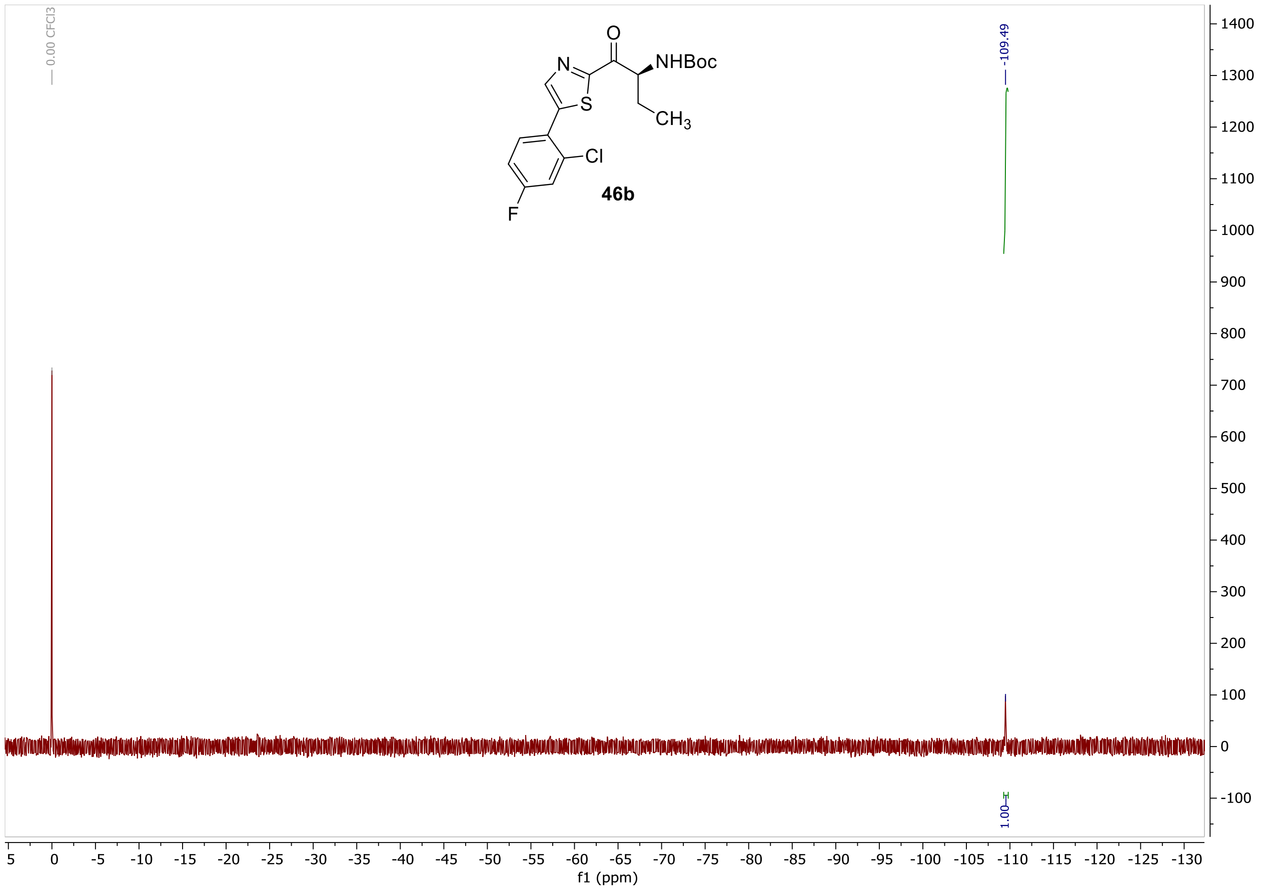

Compound **47** (^{1}H NMR, 400 MHz):

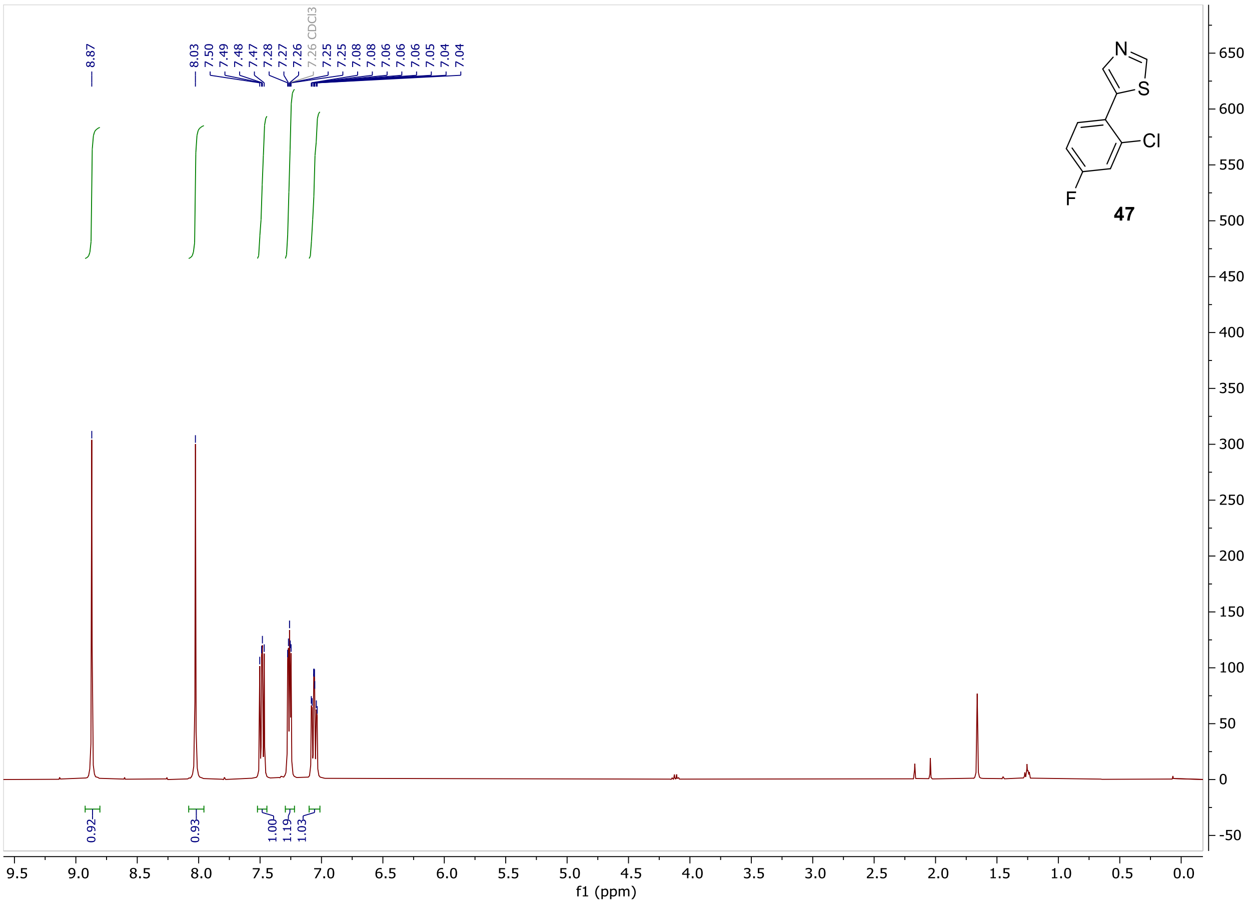

Compound **47** (^{13}C NMR, 101 MHz):

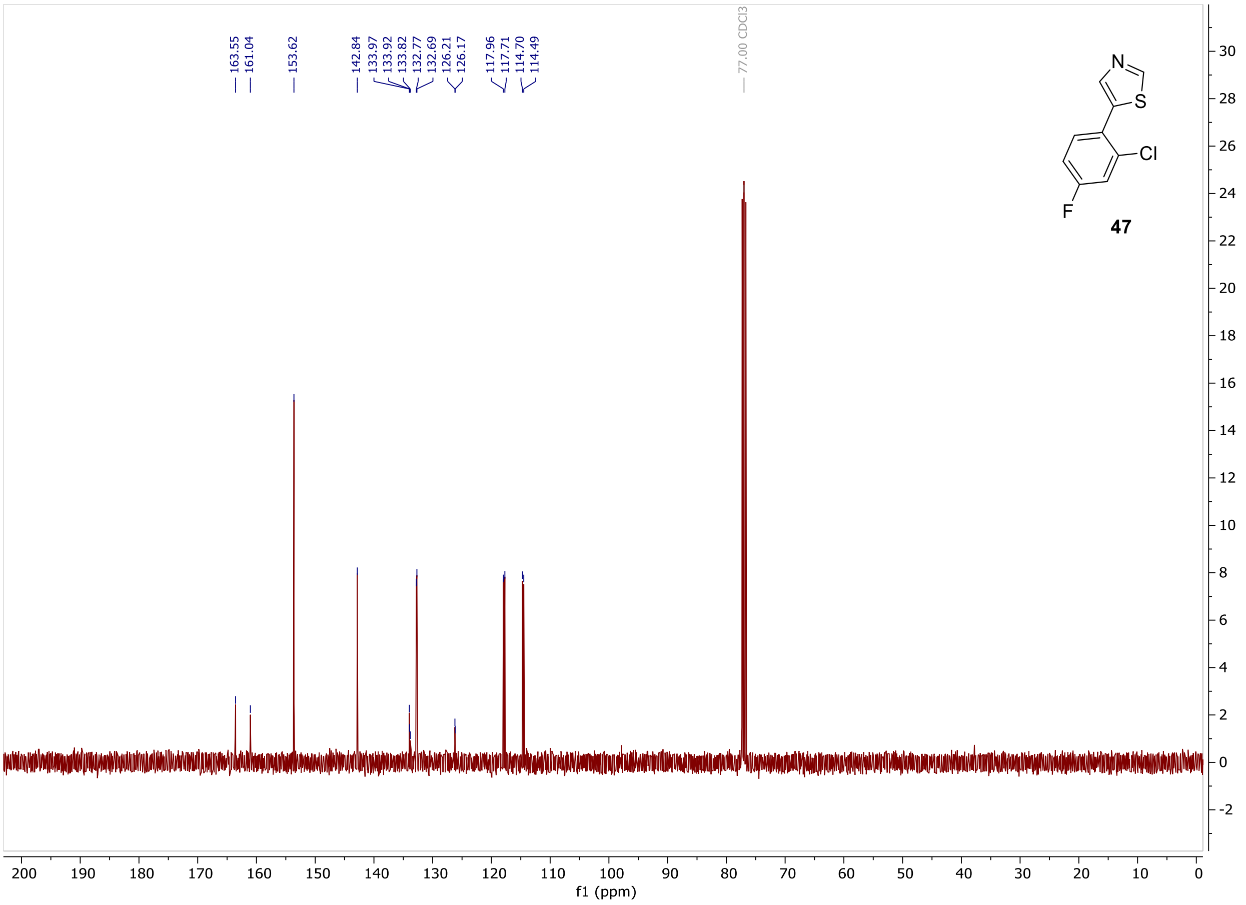

Compound **47** (^{19}F NMR, 282 MHz):

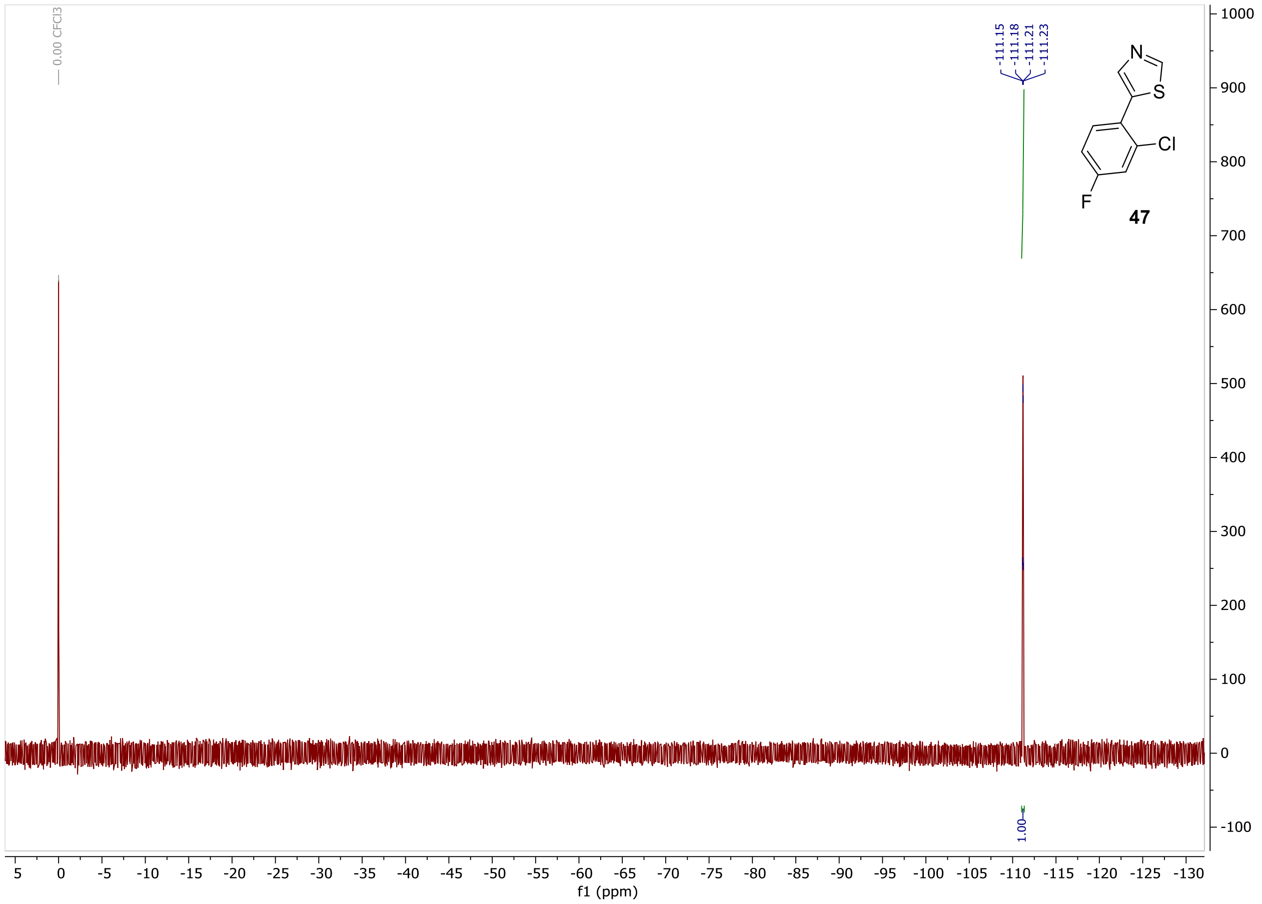

Compound **17** (^{1}H NMR, 400 MHz):

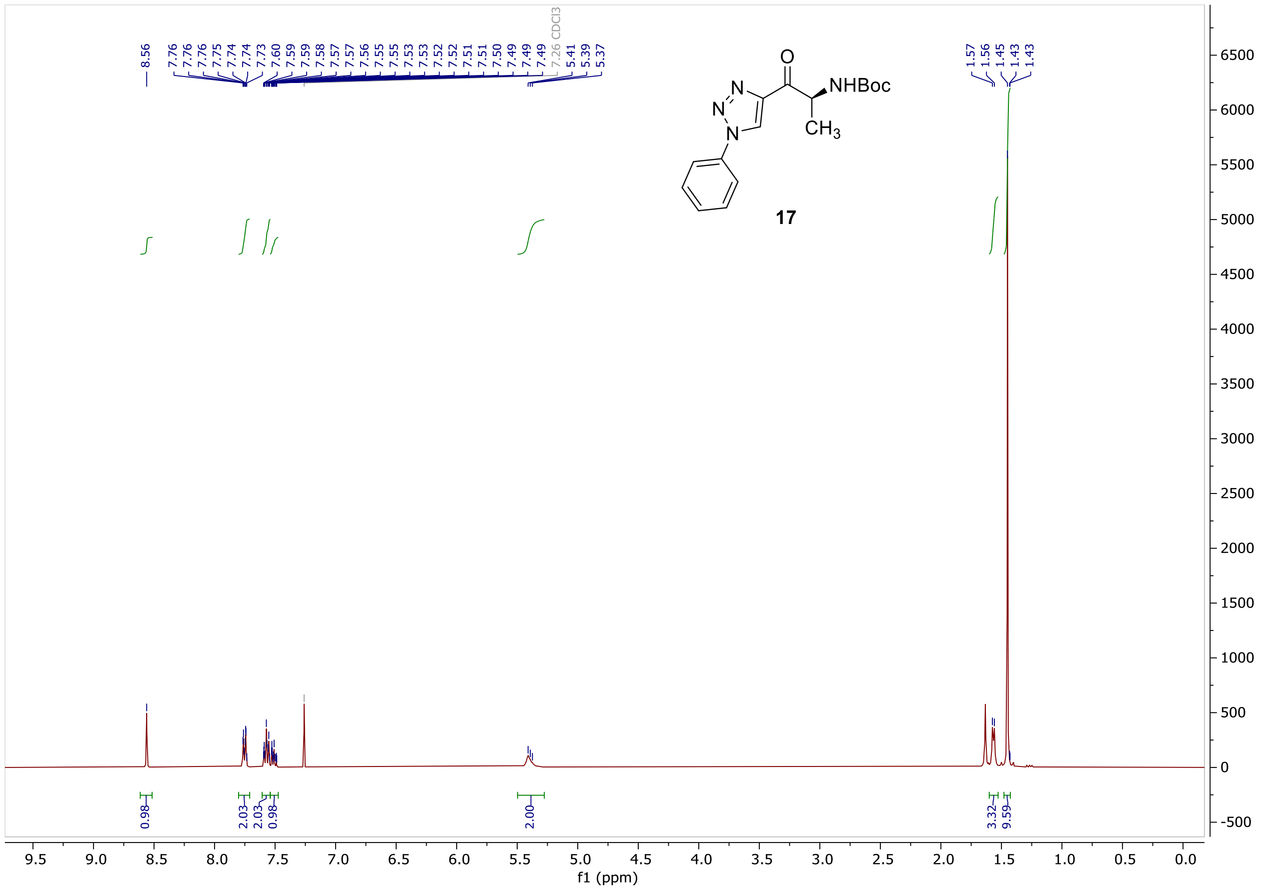

Compound **17** (^{13}C NMR, 101 MHz):

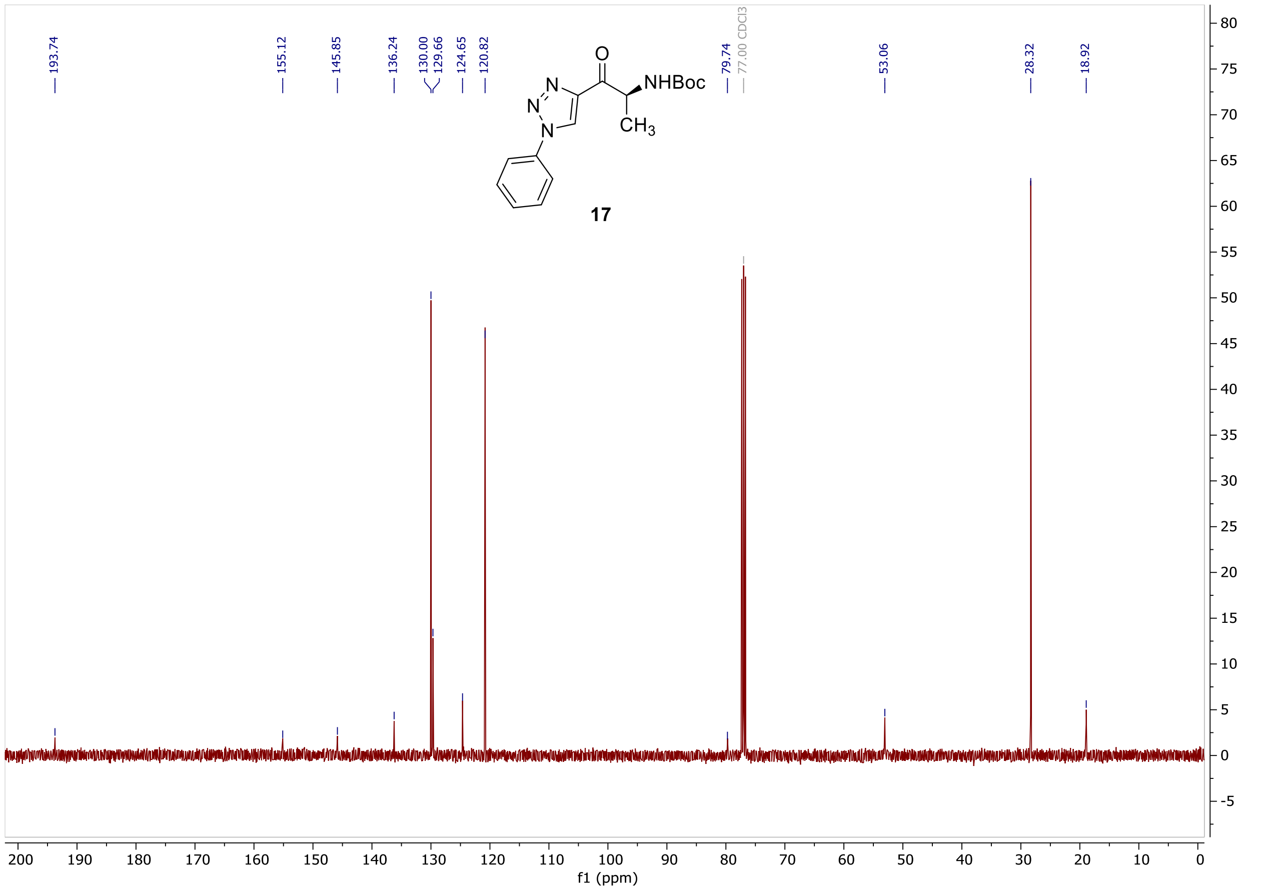

Compound **20a** (^{1}H NMR, 400 MHz):

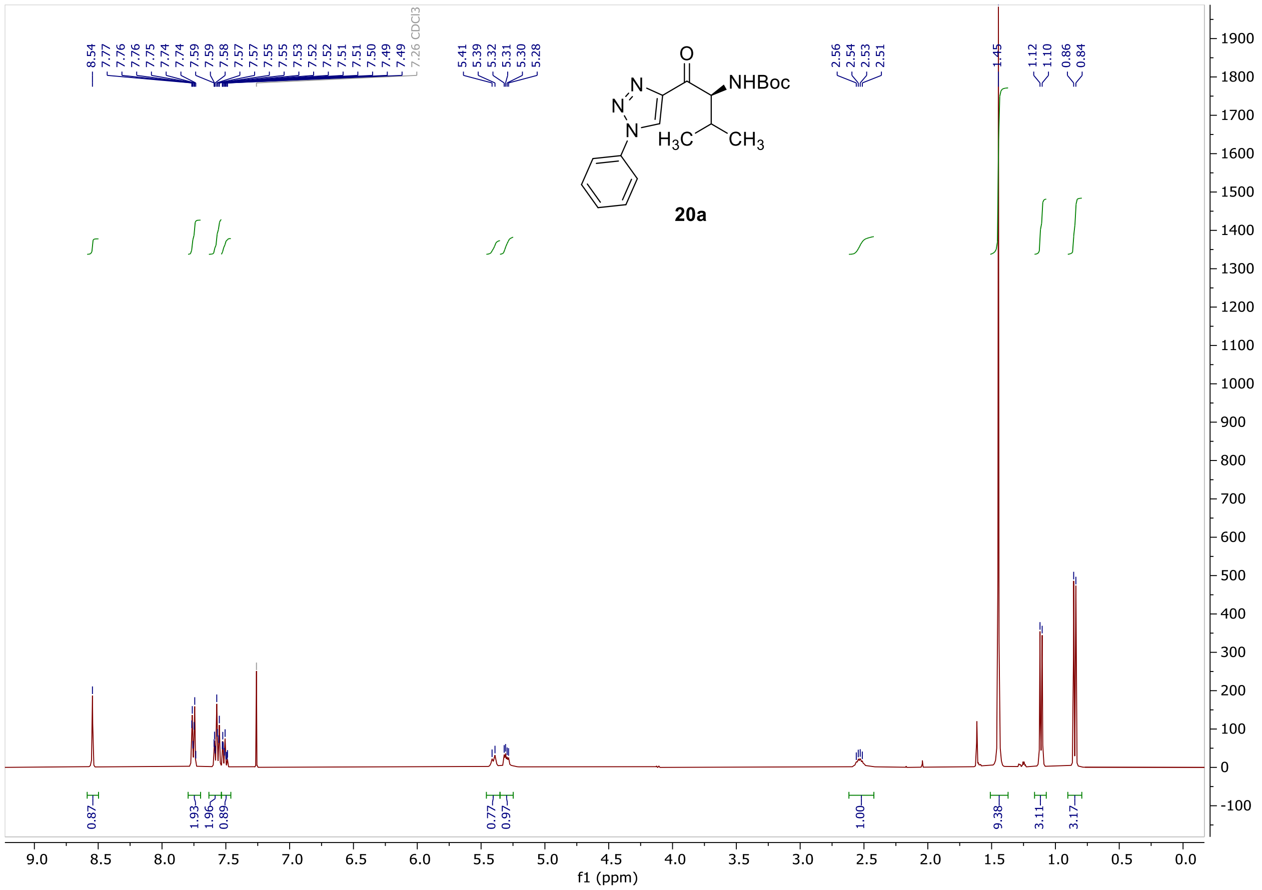

Compound **20a** (^{13}C NMR, 101 MHz):

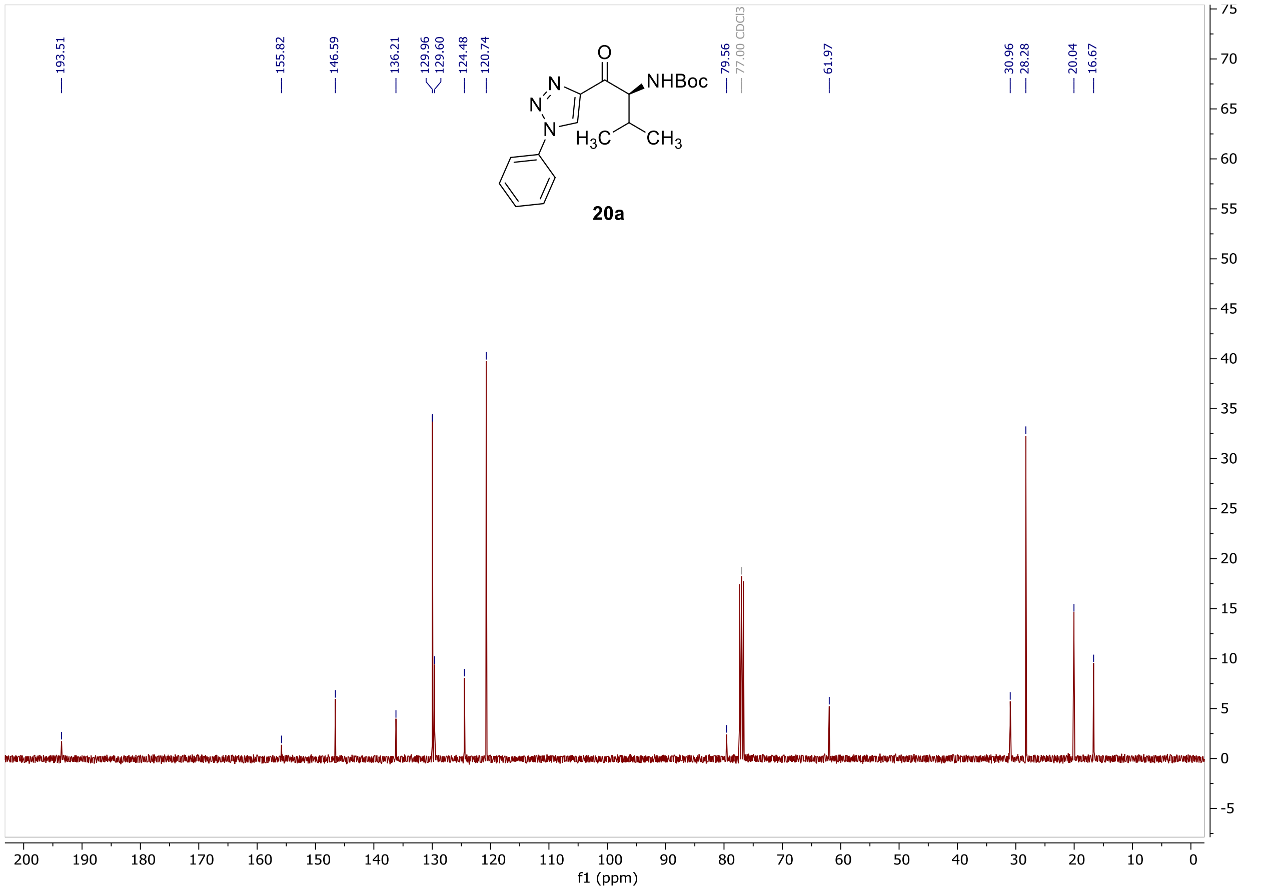

Compound **20b** (^{1}H NMR, 400 MHz):

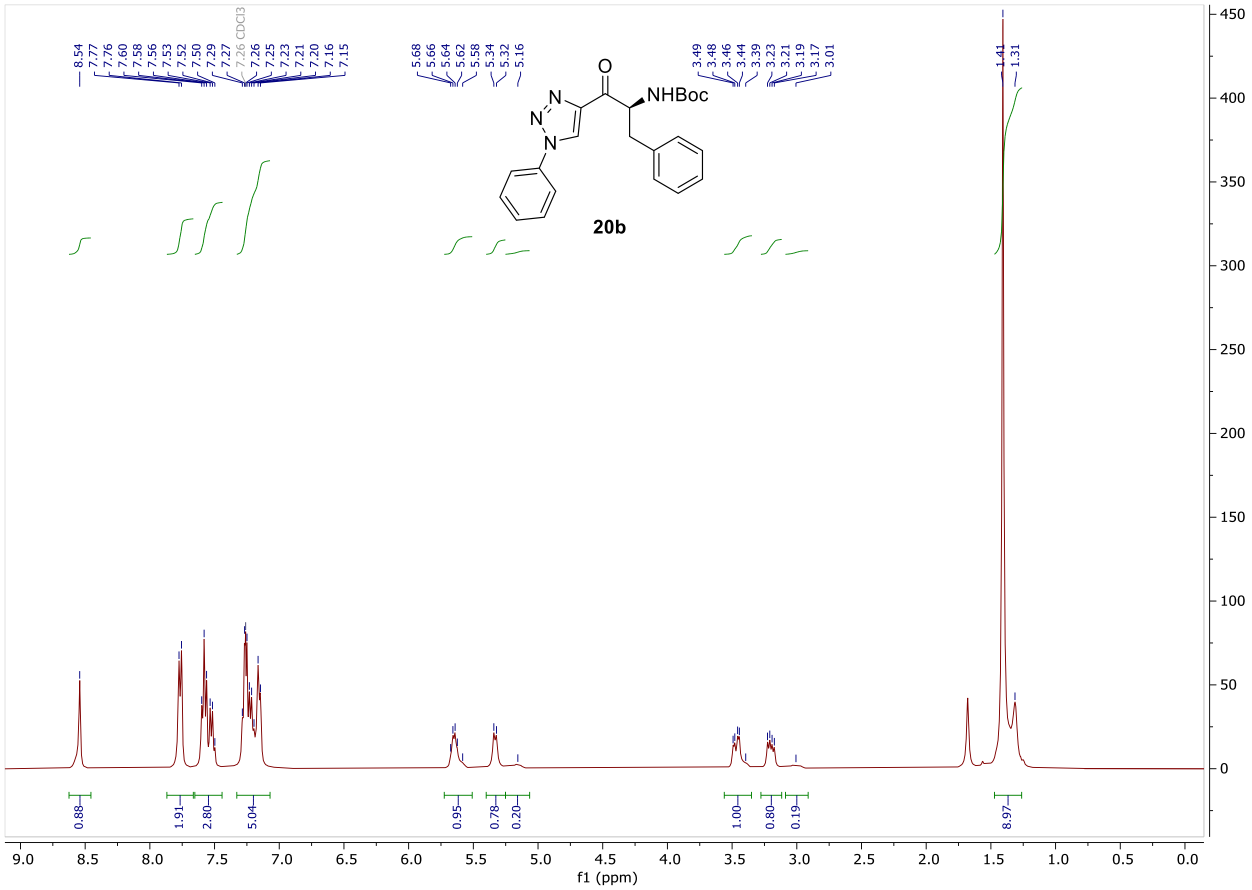

Compound **20b** (^{13}C NMR, 101 MHz):

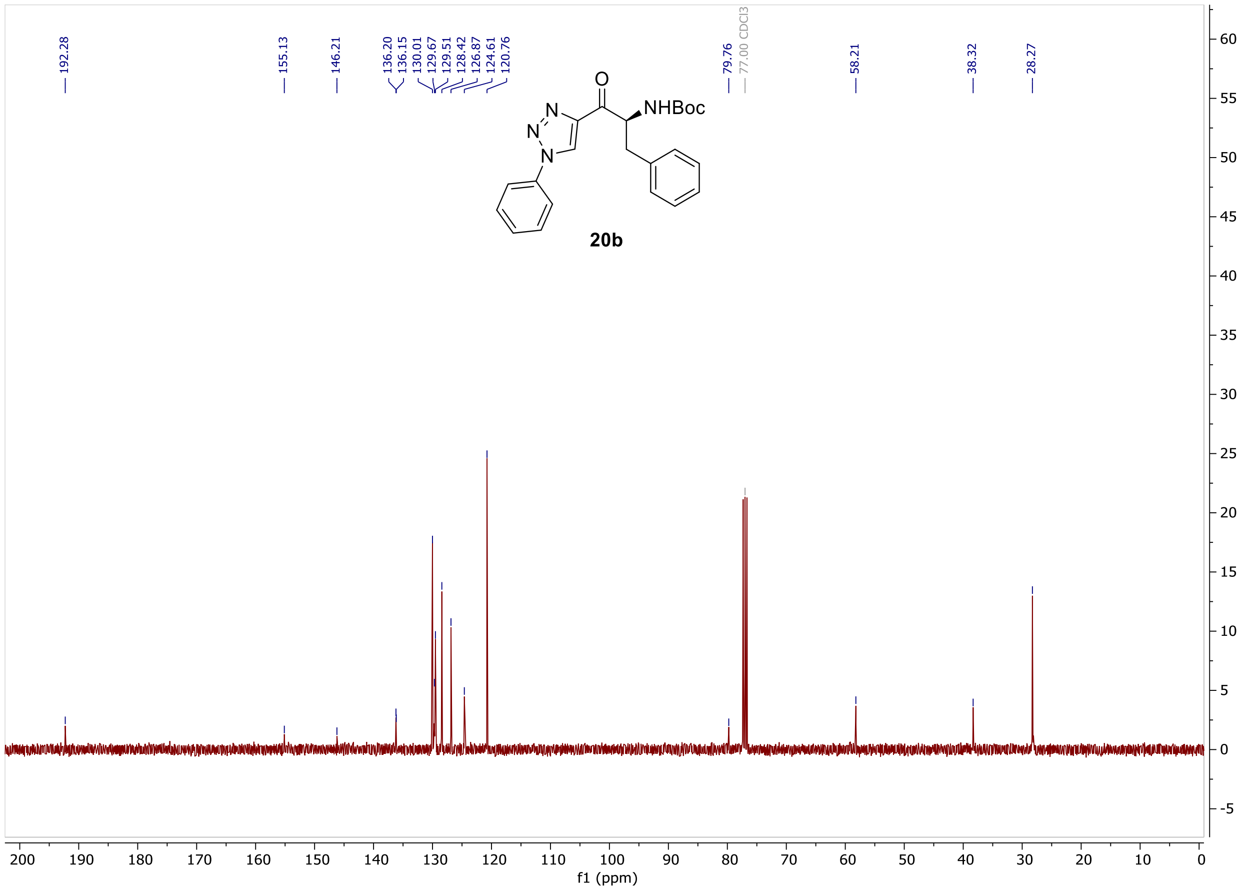

Compound **20c** (^{1}H NMR, 400 MHz):

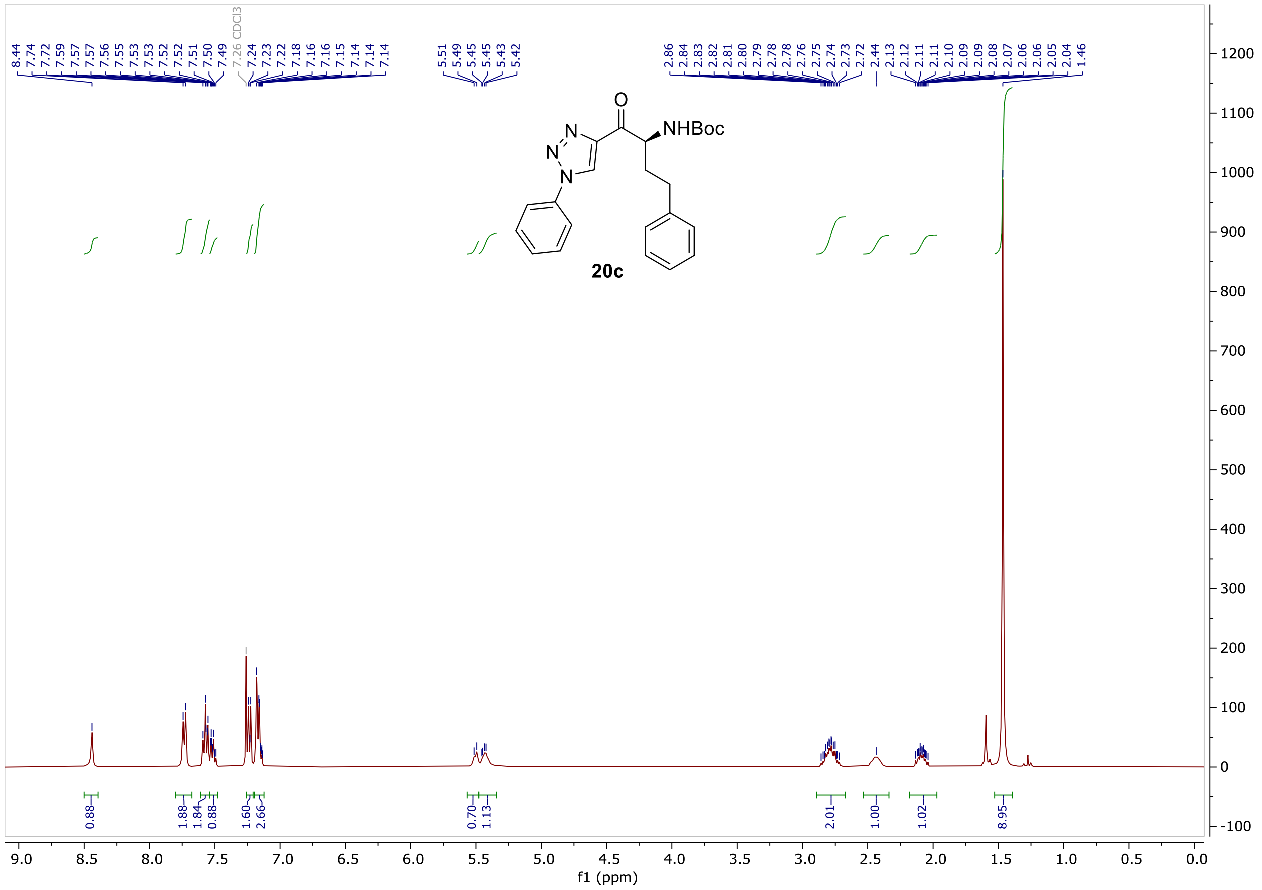

Compound **20c** (^{13}C NMR, 101 MHz):

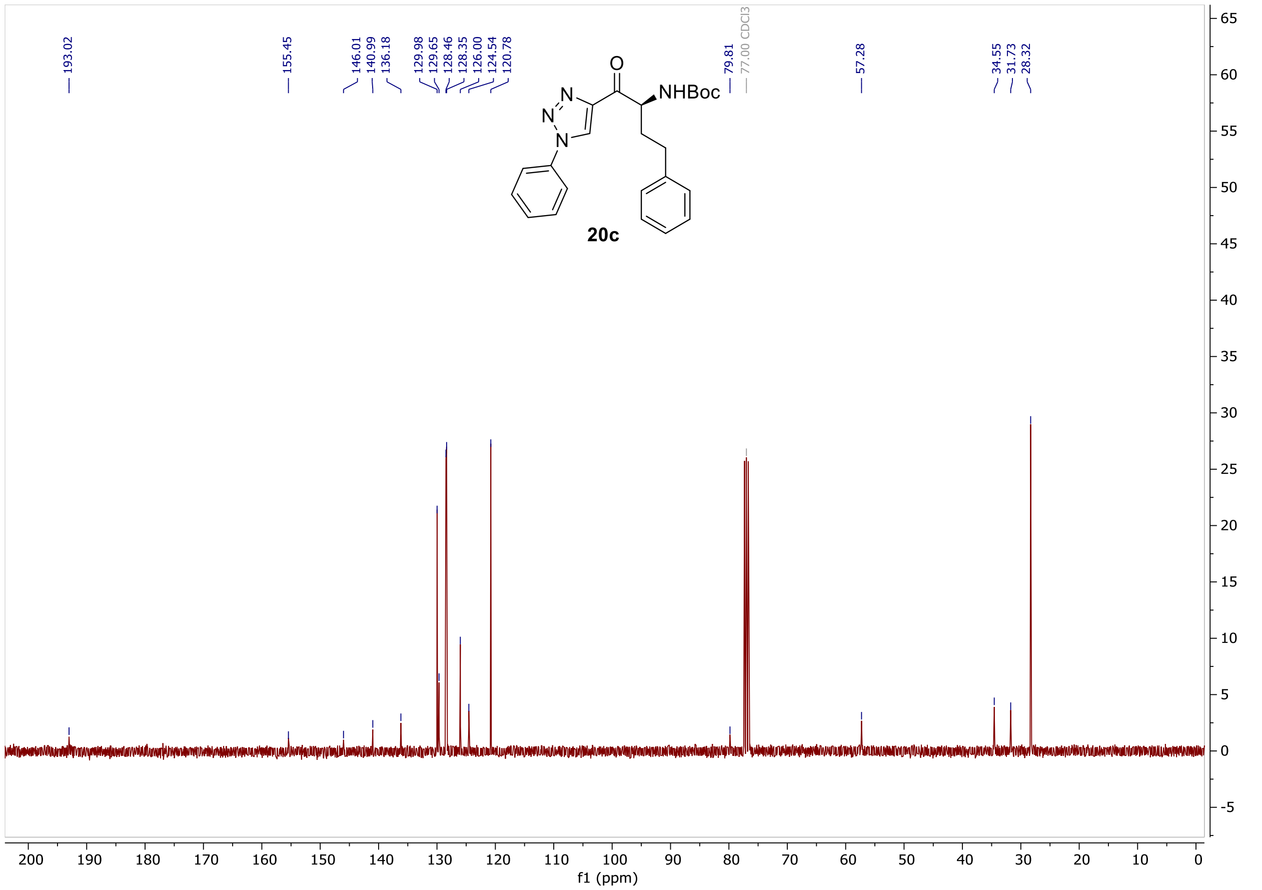

Compound **20d** (^{1}H NMR, 400 MHz):

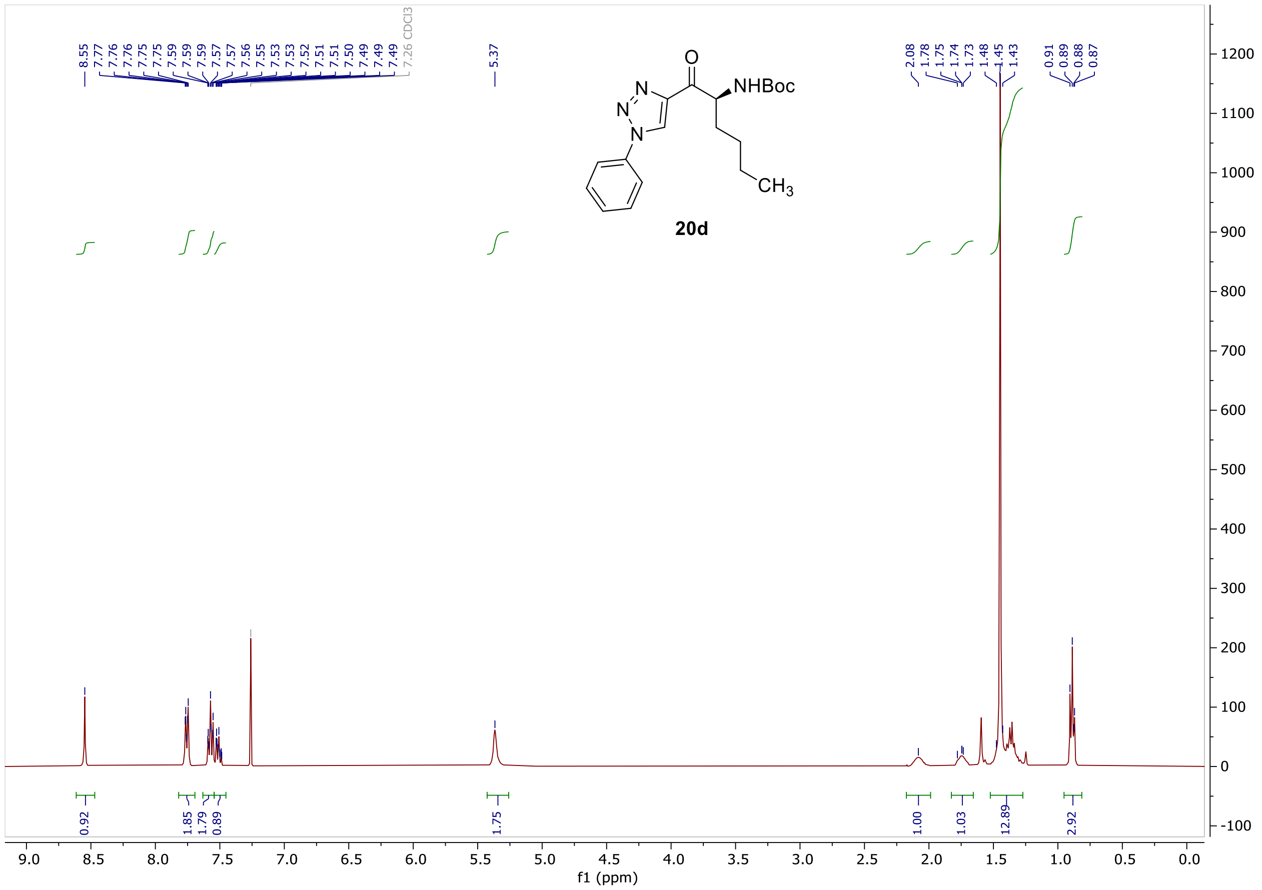

Compound **20d** (^{13}C NMR, 101 MHz):

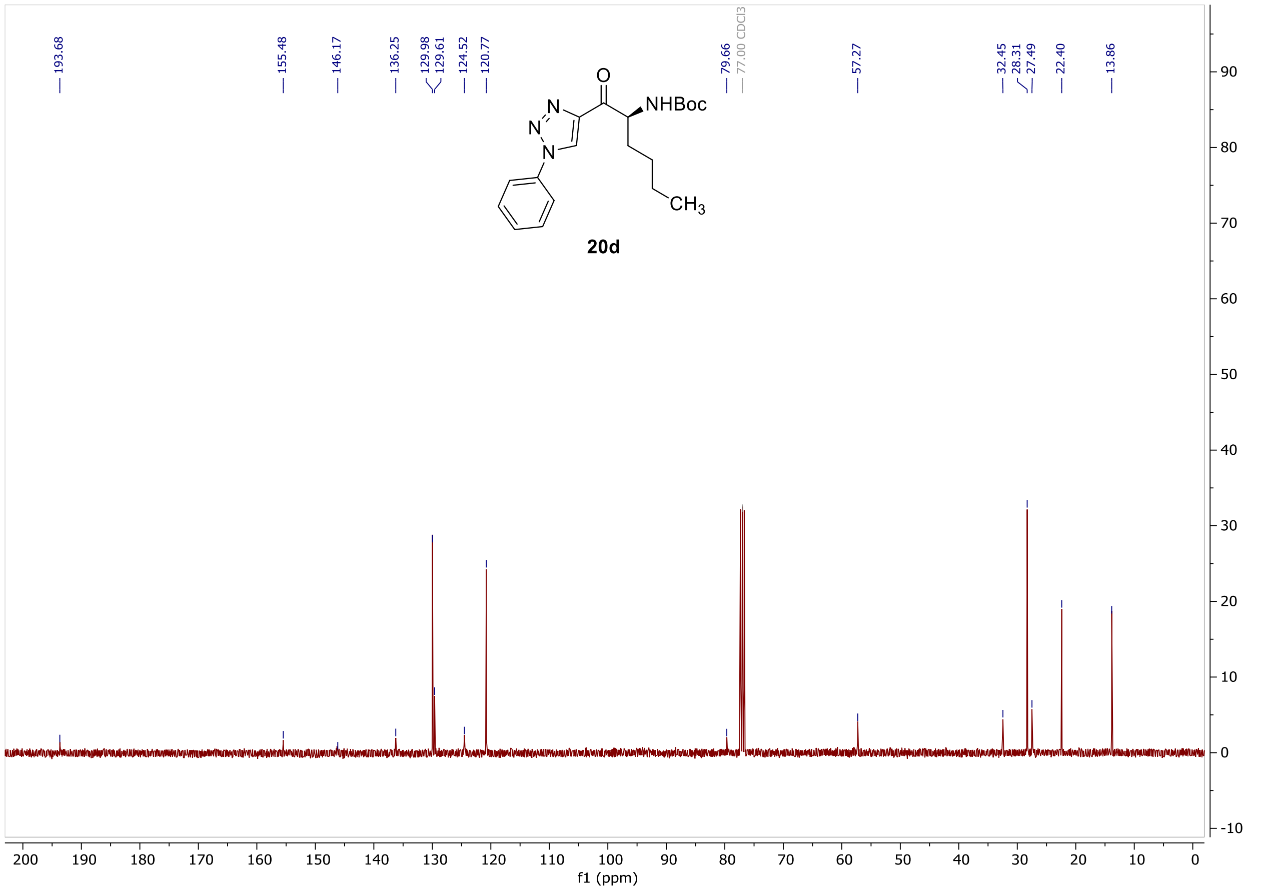

Compound **20e** (^{1}H NMR, 400 MHz):

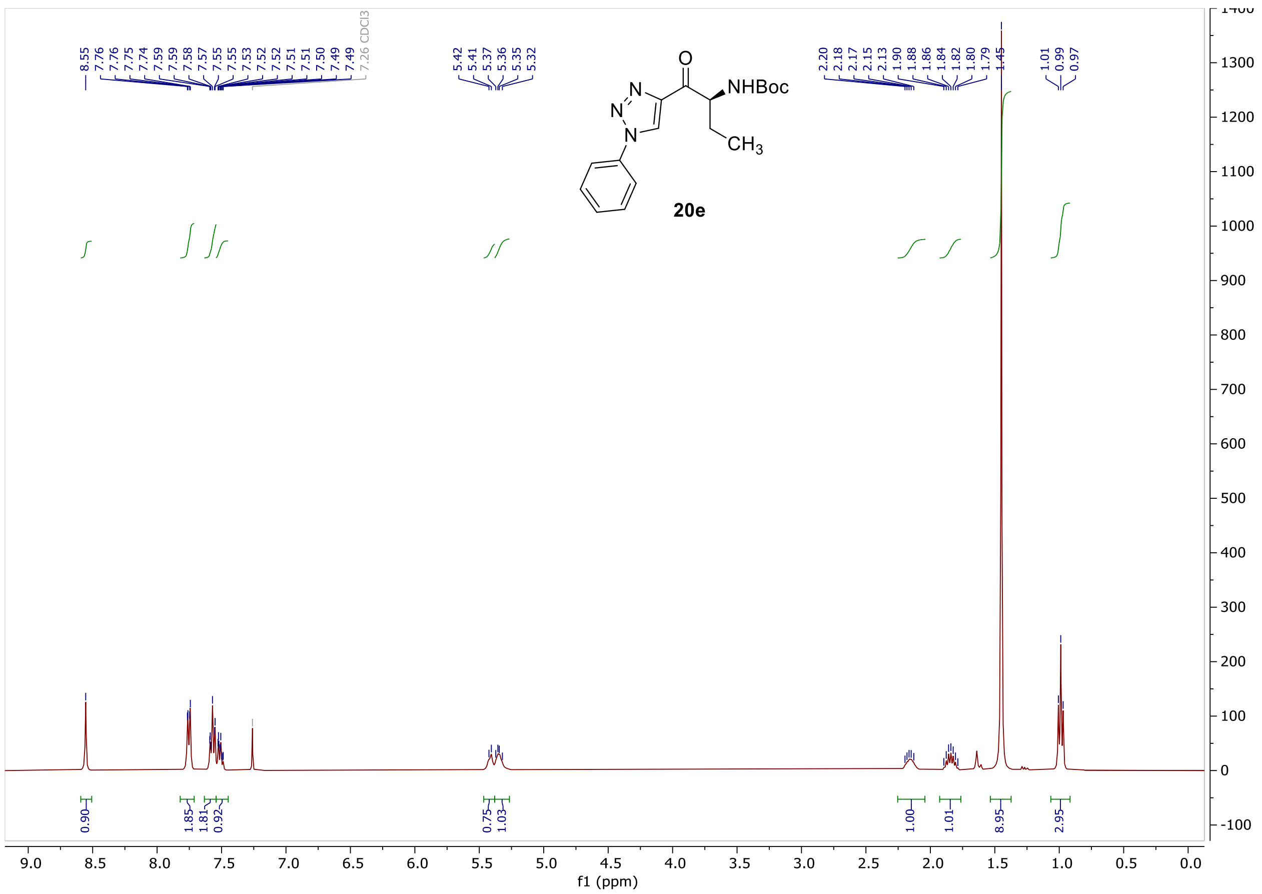

Compound **20e** (^{13}C NMR, 101 MHz):

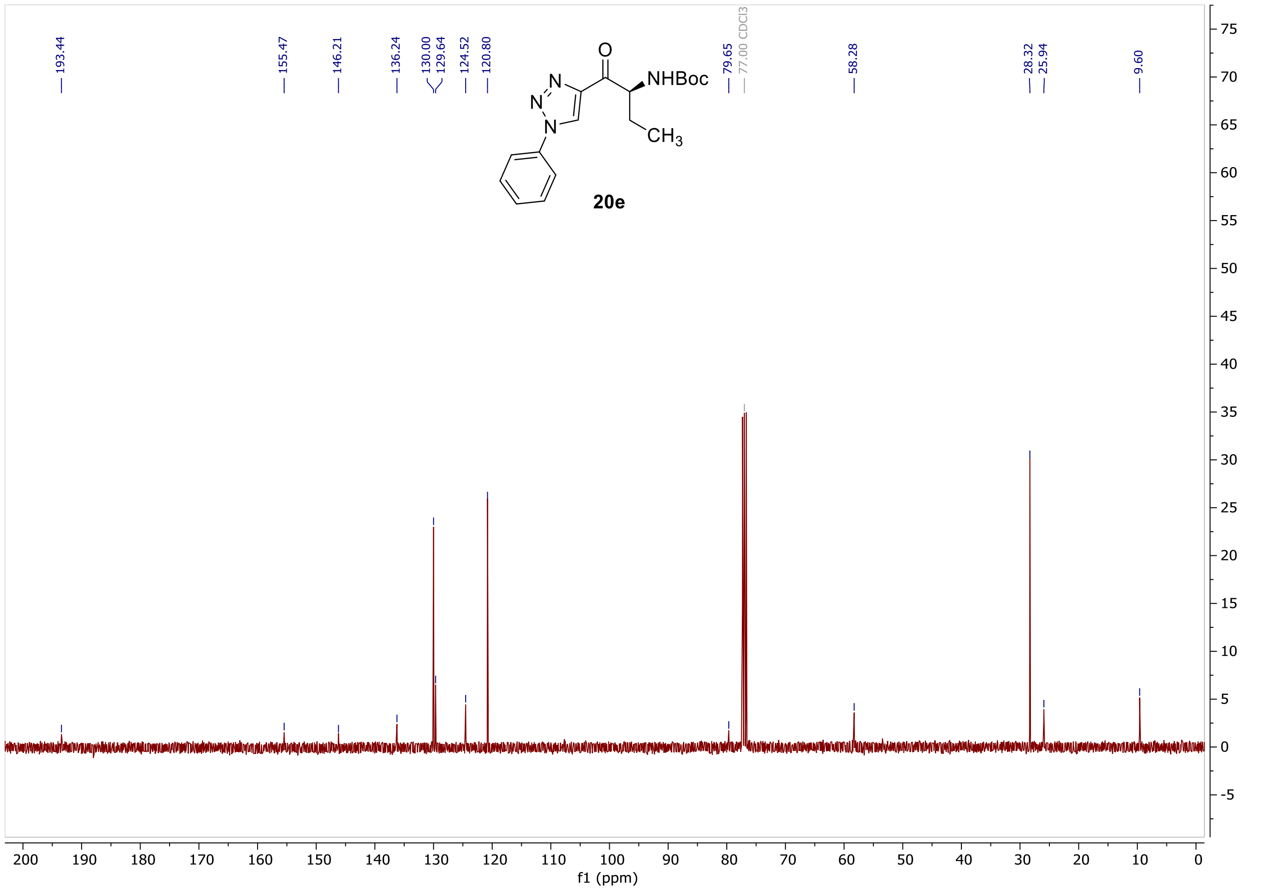

Compound **20f** (^{1}H NMR, 400 MHz):

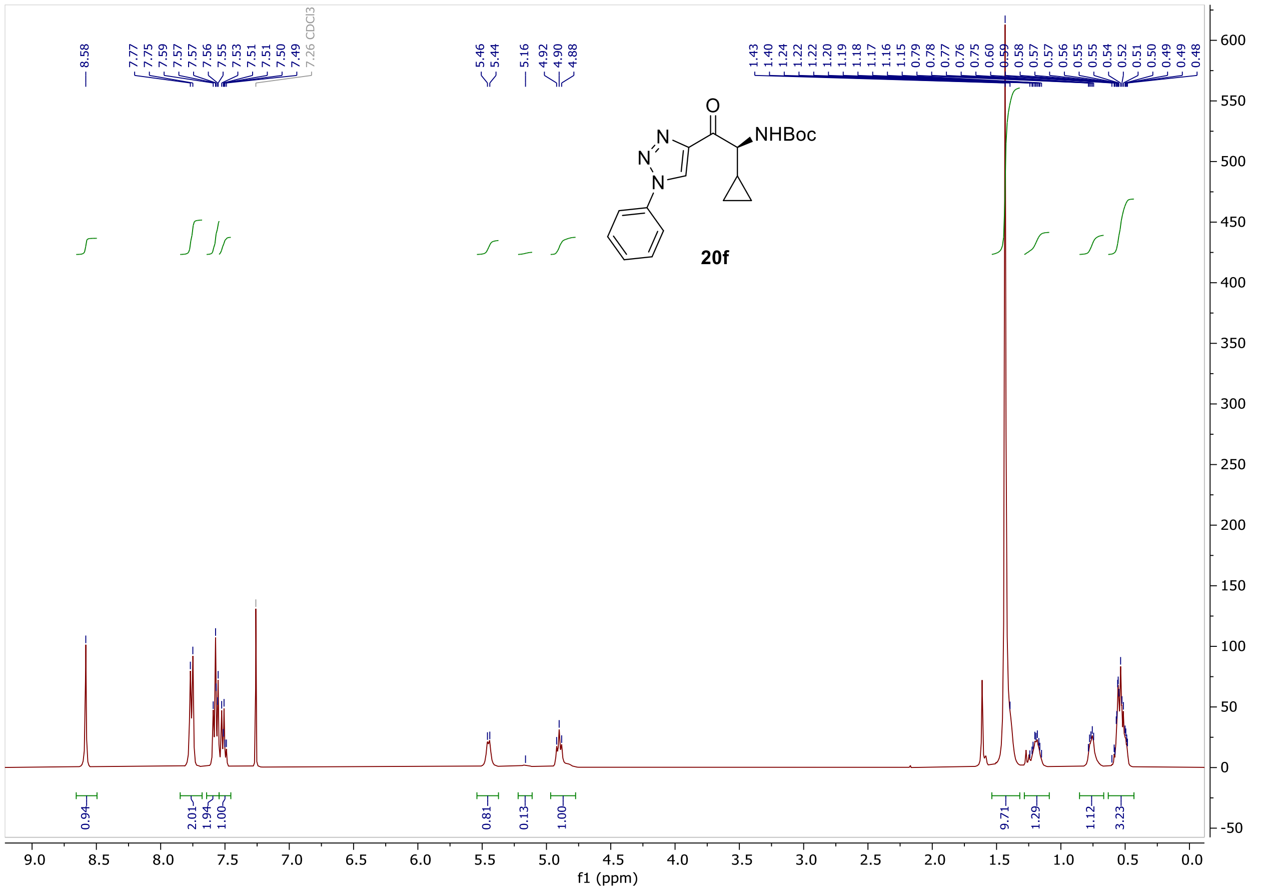

Compound **20f** (^{13}C NMR, 101 MHz):

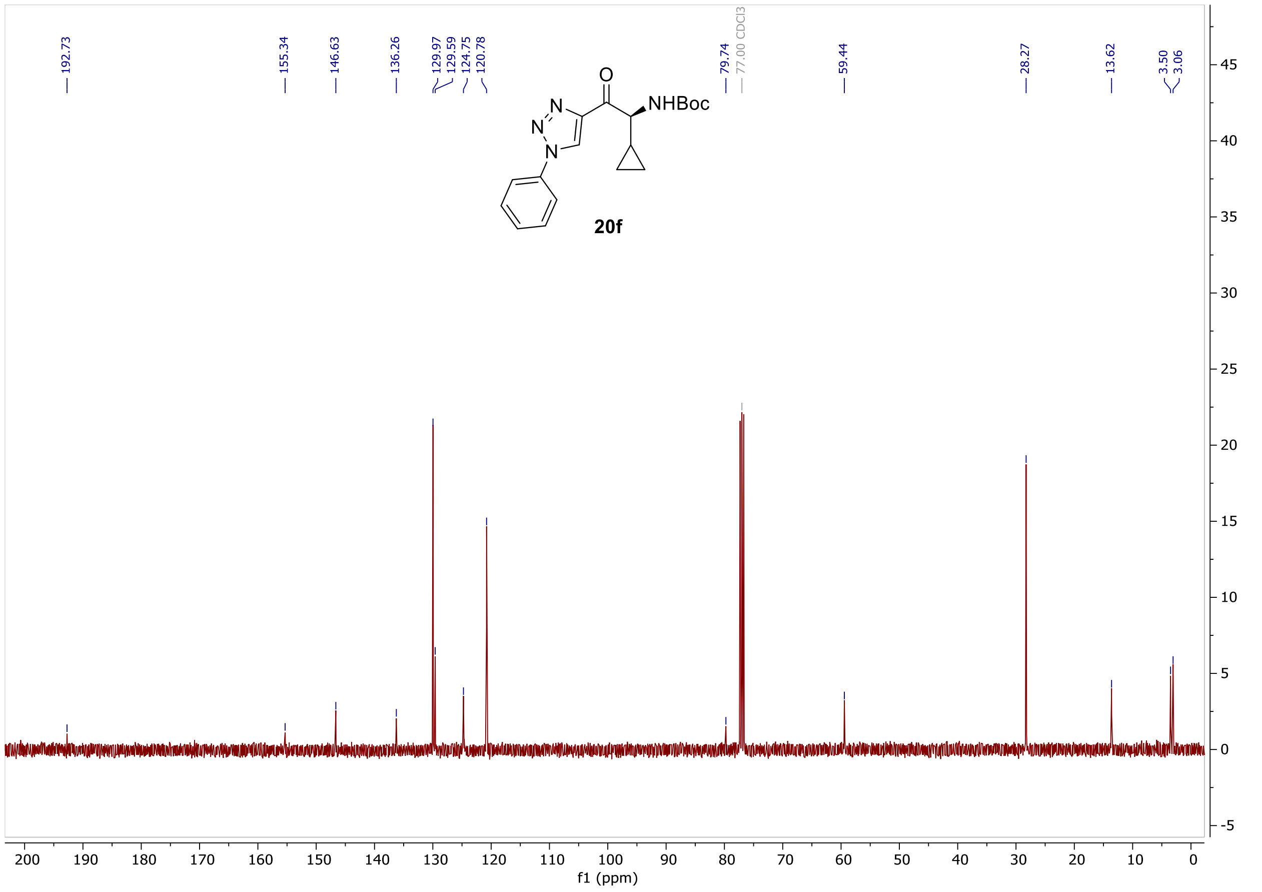

Compound **31a** (^{1}H NMR, 400 MHz):

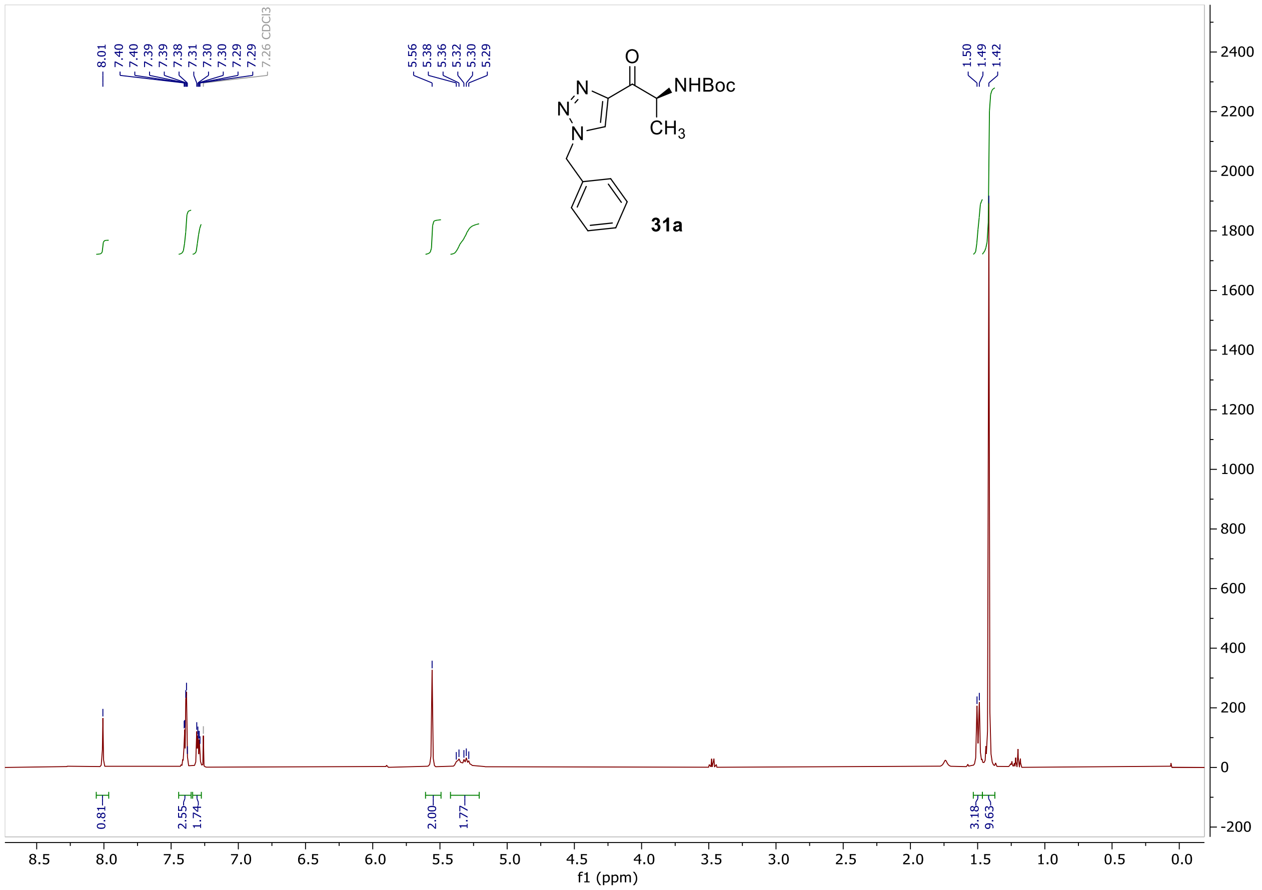

Compound **31a** (^{13}C NMR, 101 MHz):

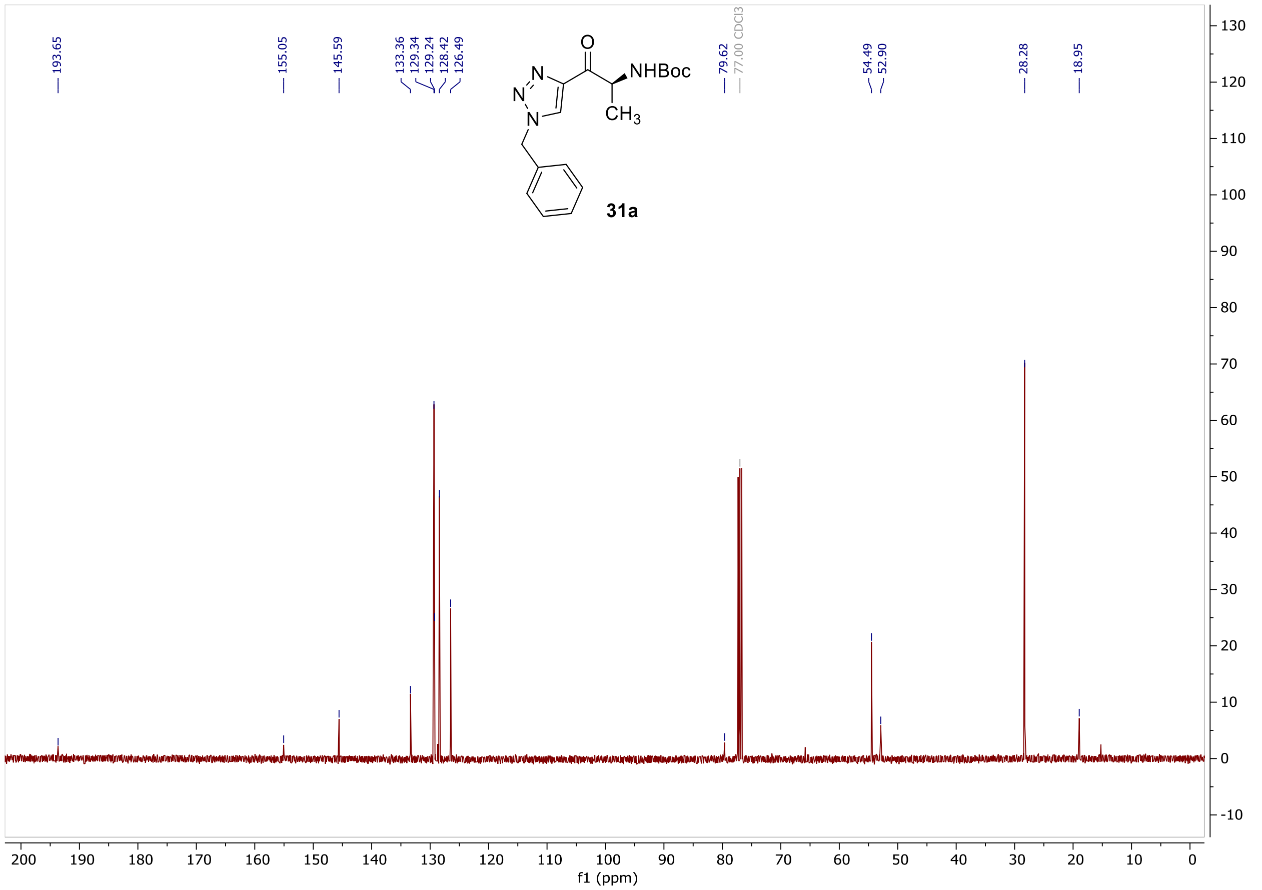

Compound **31b** (^{1}H NMR, 400 MHz):

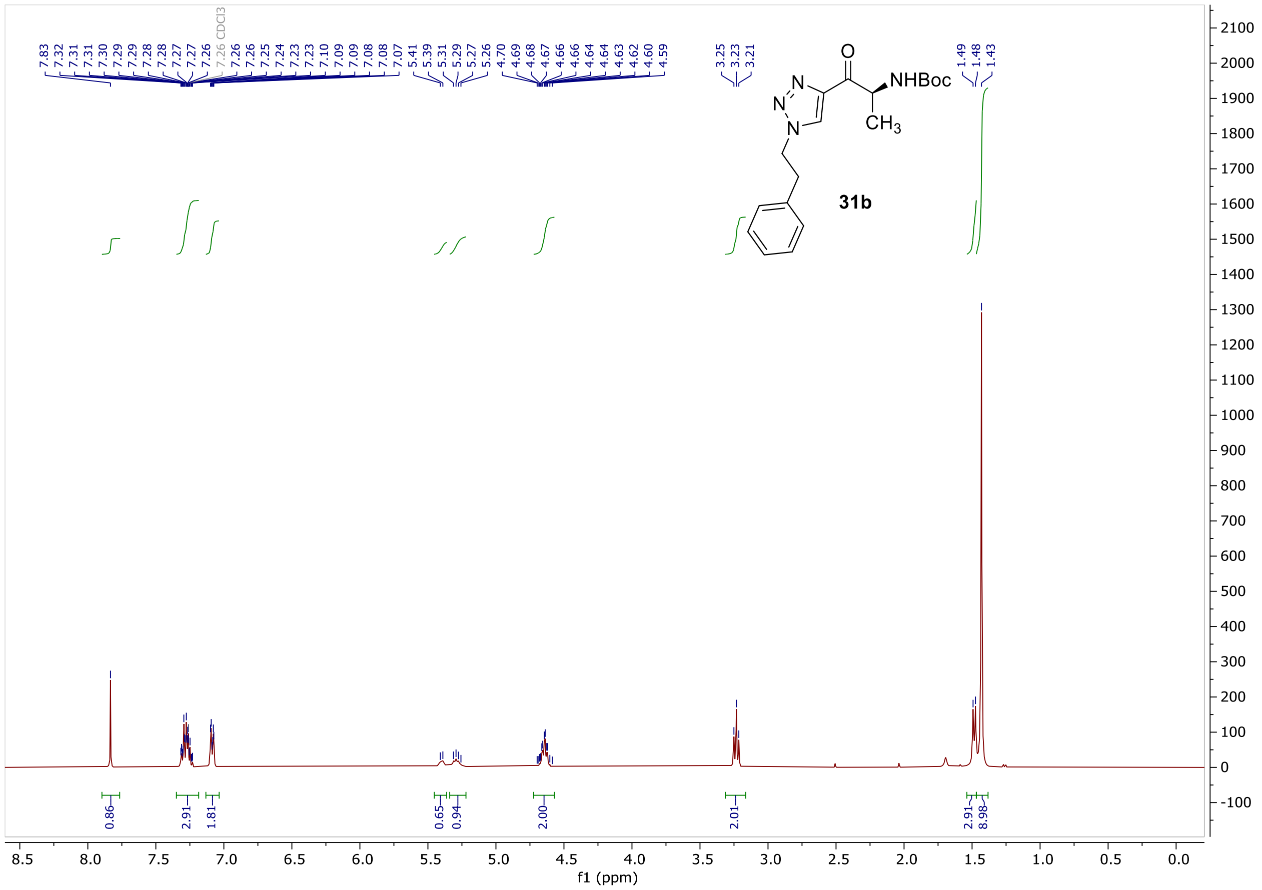

Compound **31b** (^{13}C NMR, 101 MHz):

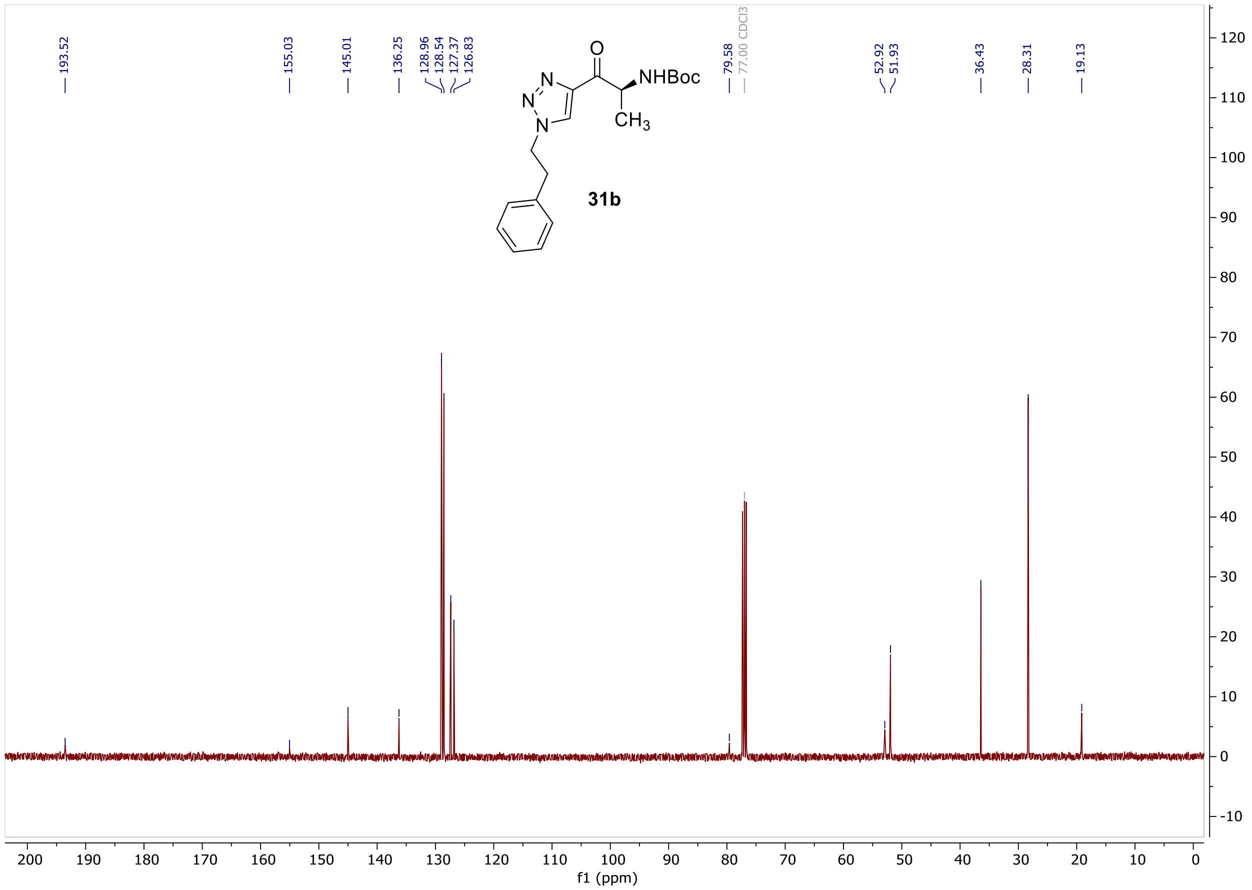

Compound **27a** (^{1}H NMR, 400 MHz):

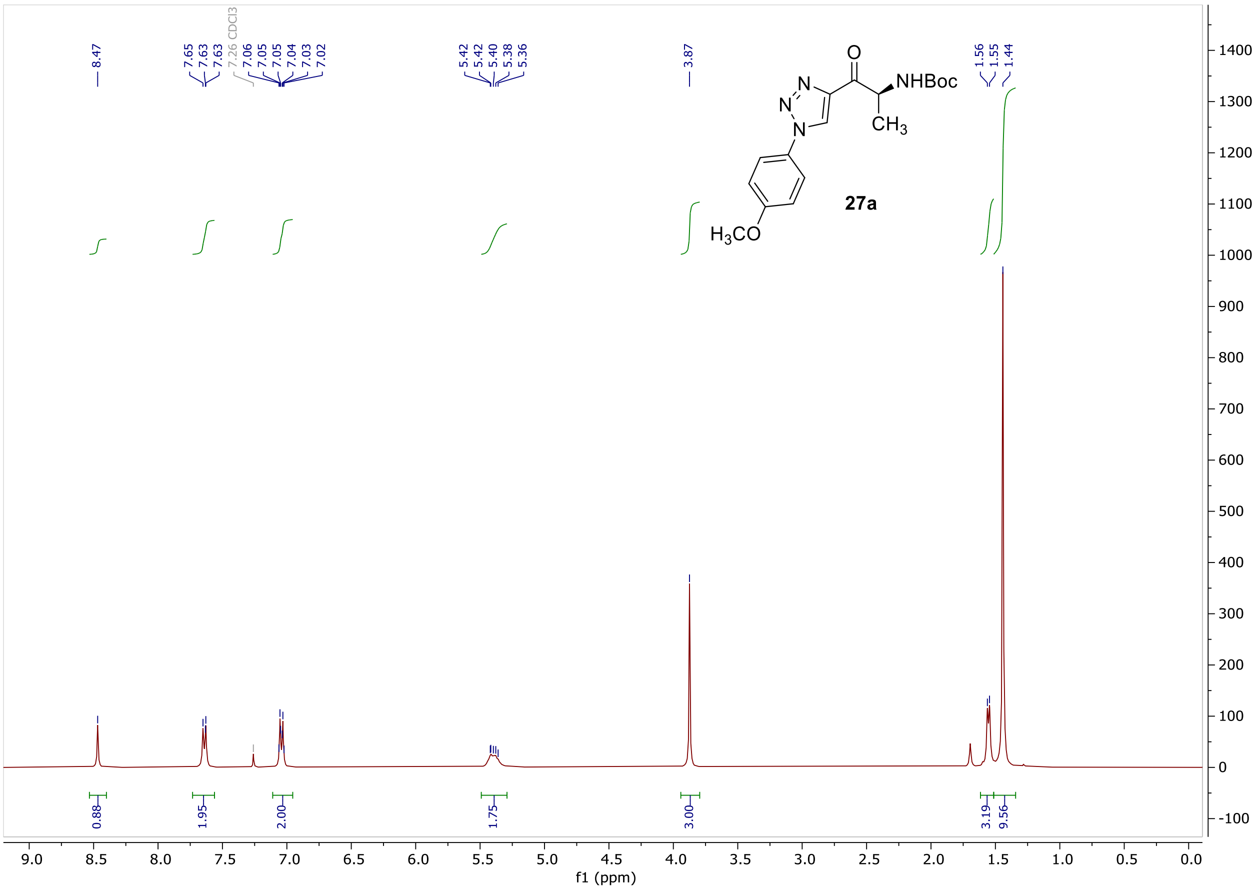

Compound **27a** (^{13}C NMR, 101 MHz):

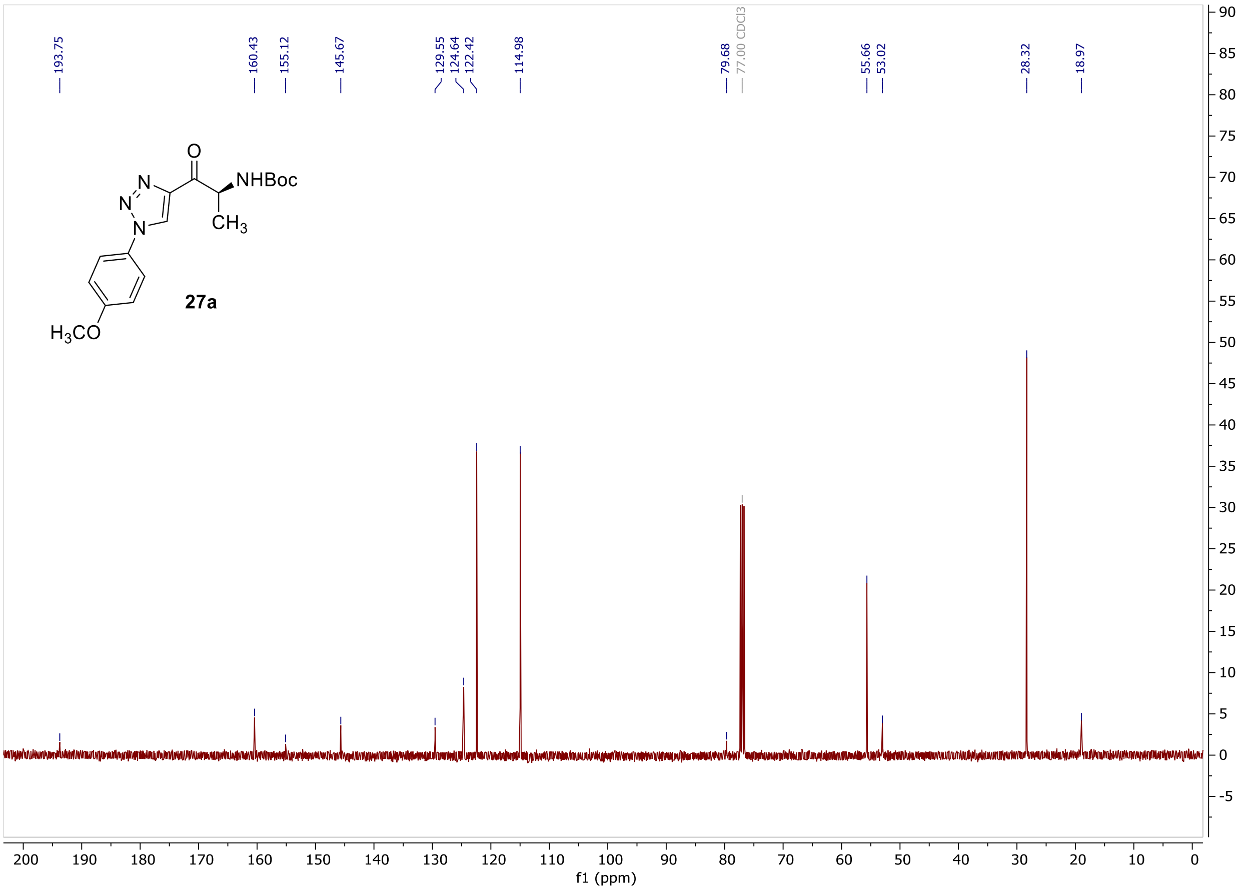

Compound **27b** (^{1}H NMR, 400 MHz):

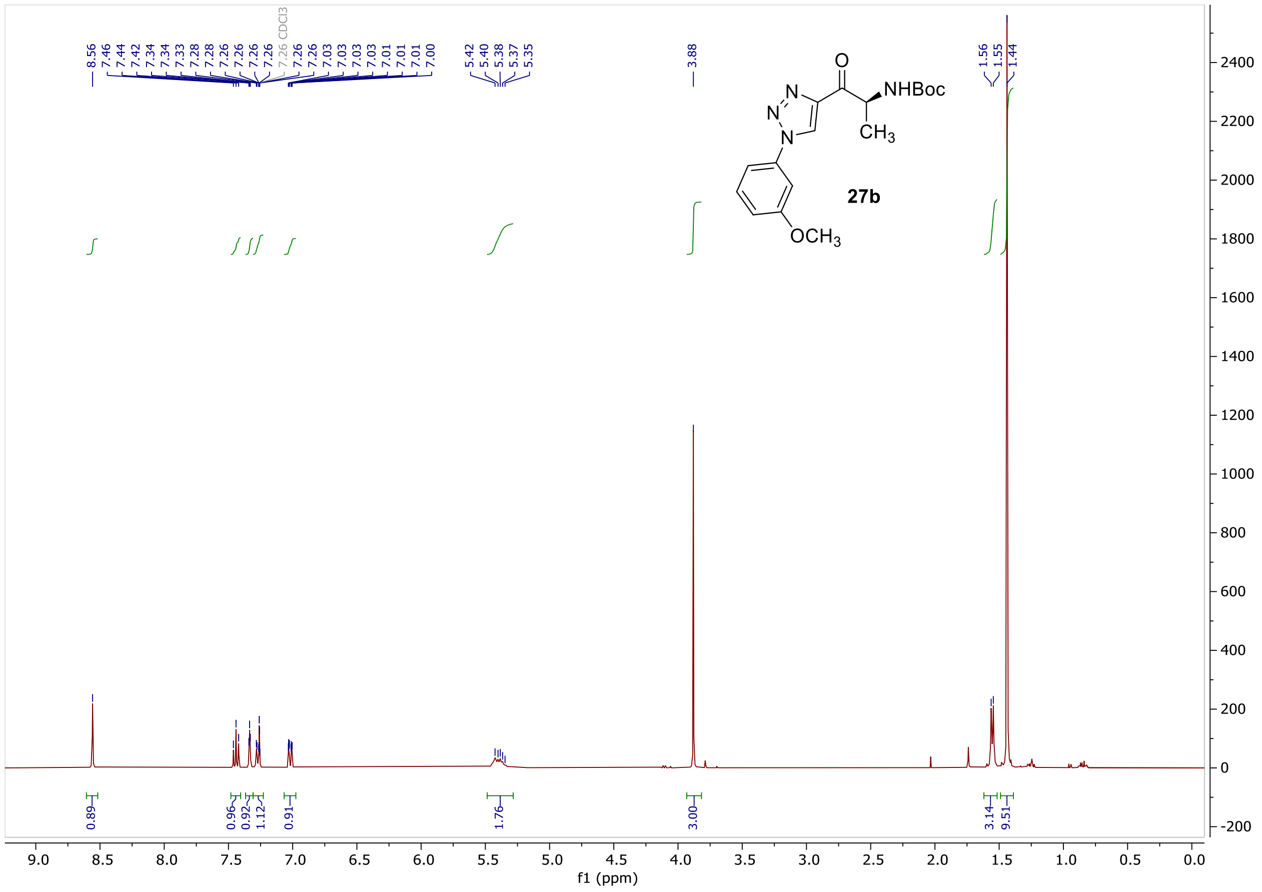

Compound **27b** (^{13}C NMR, 101 MHz):

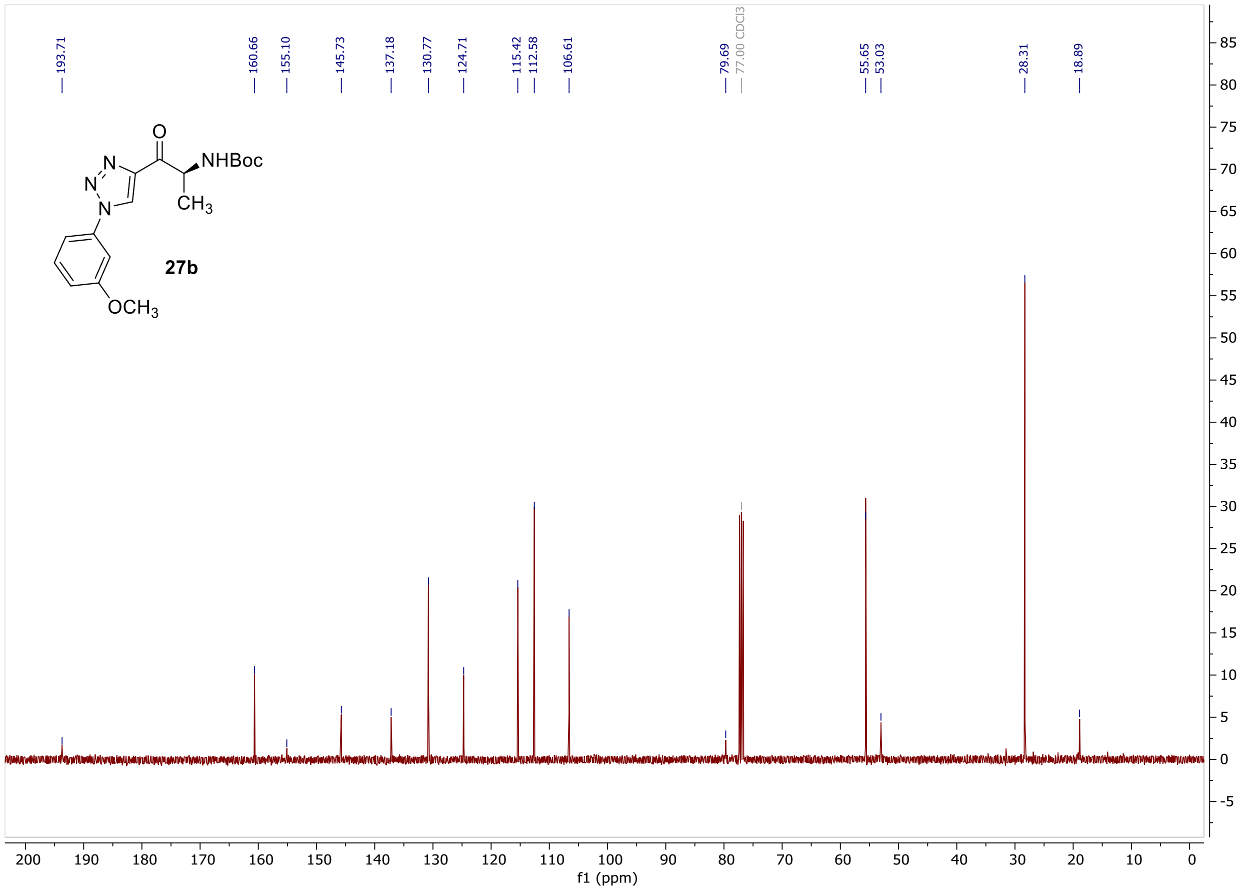

Compound **27c** (^{1}H NMR, 400 MHz):

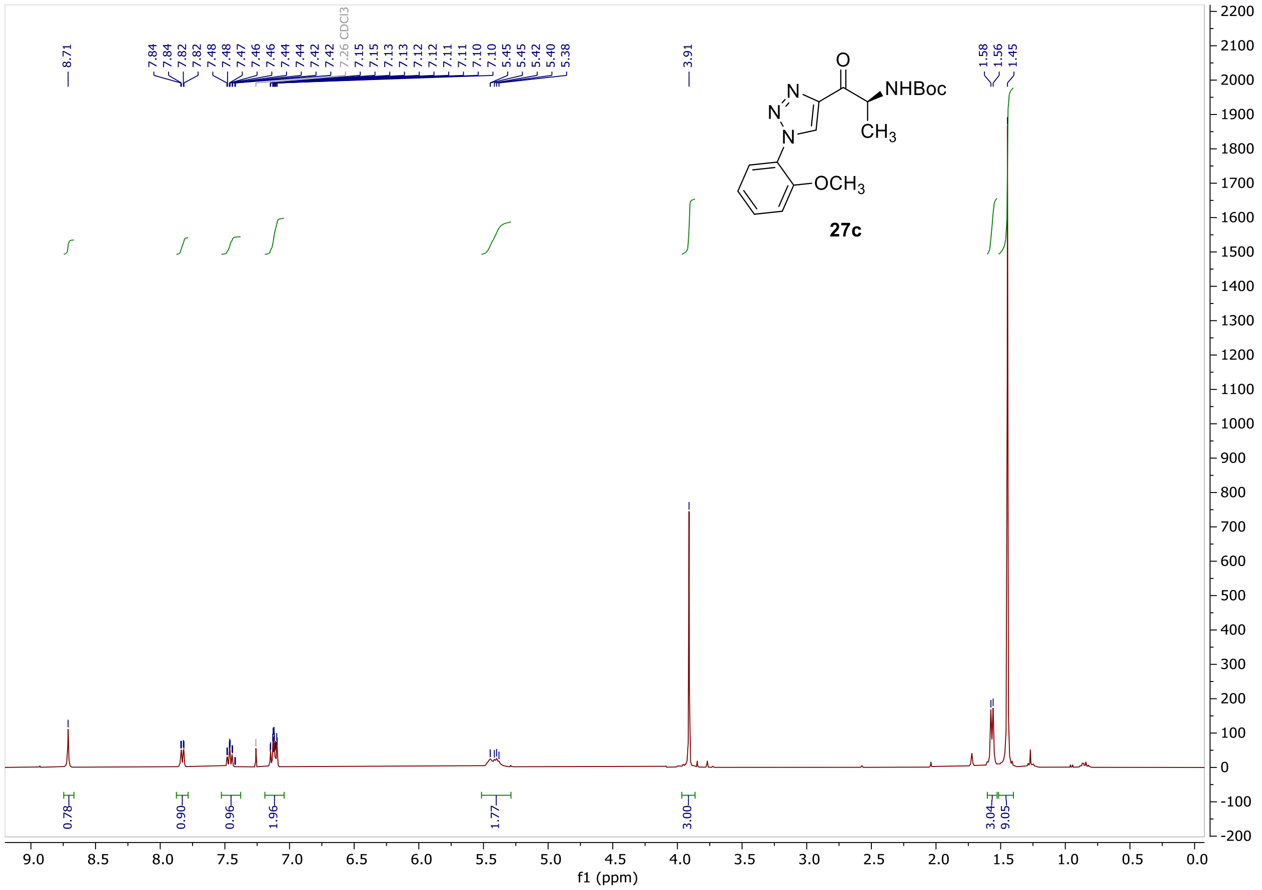

Compound **27c** (^{13}C NMR, 101 MHz):

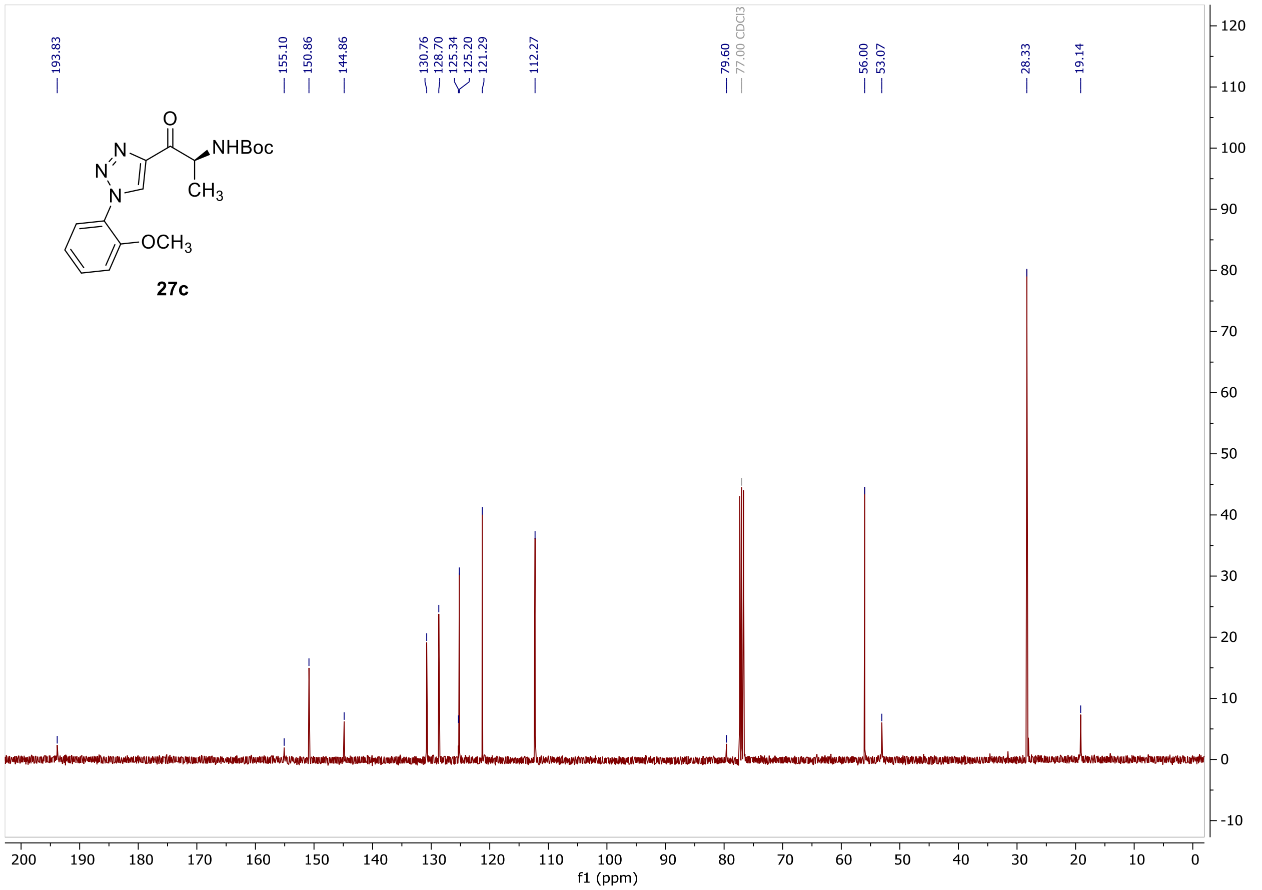

Compound **27d** (^{1}H NMR, 400 MHz):

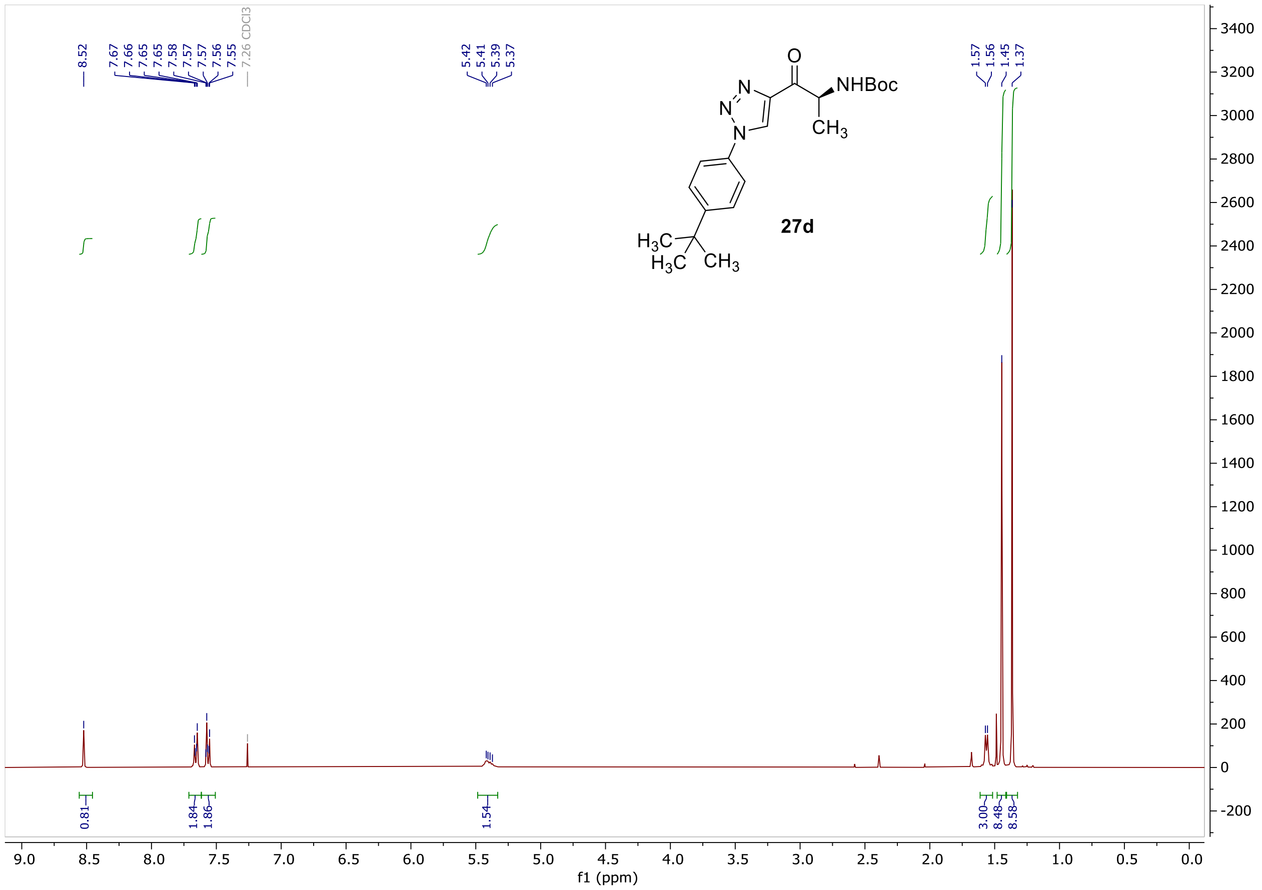

Compound **27d** (^{13}C NMR, 101 MHz):

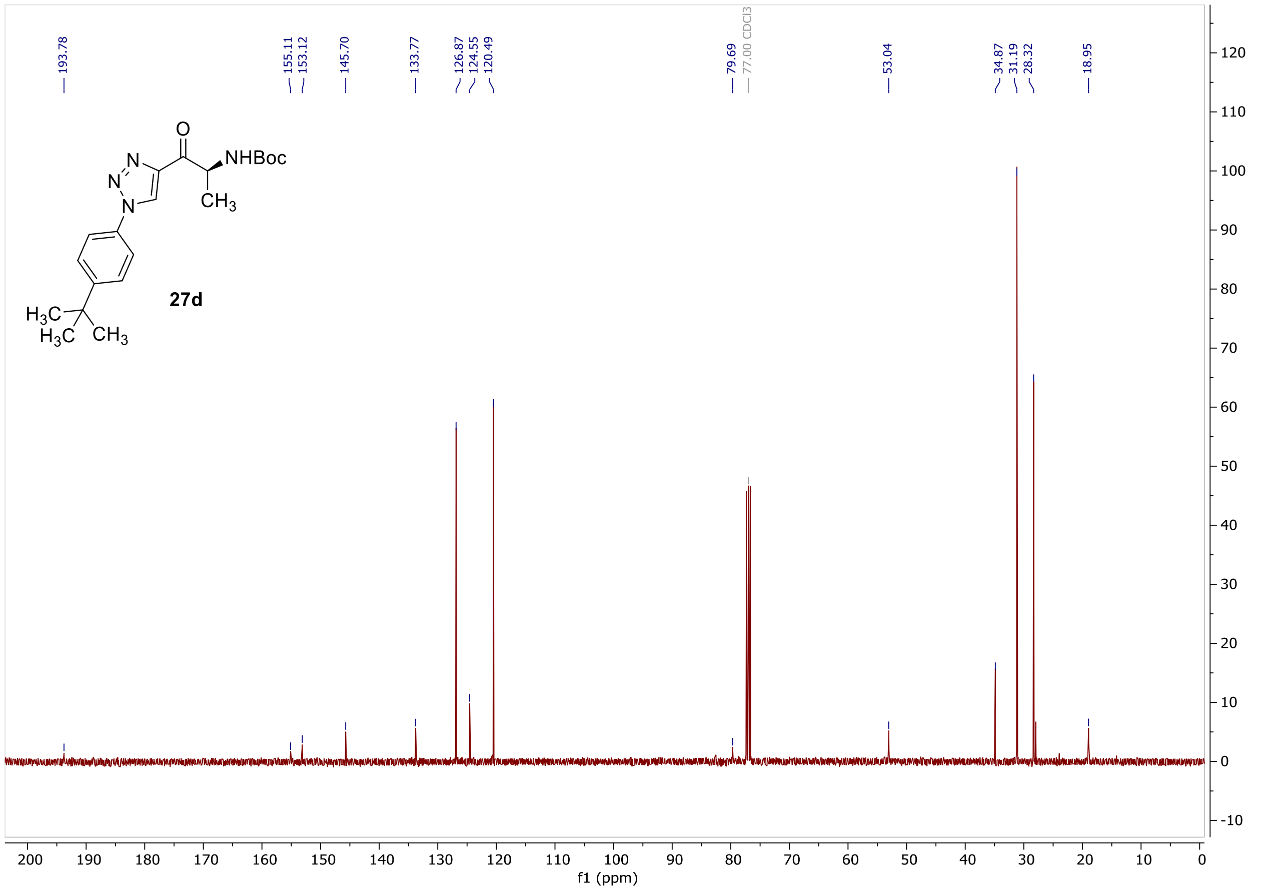

Compound **27e** (^{1}H NMR, 400 MHz):

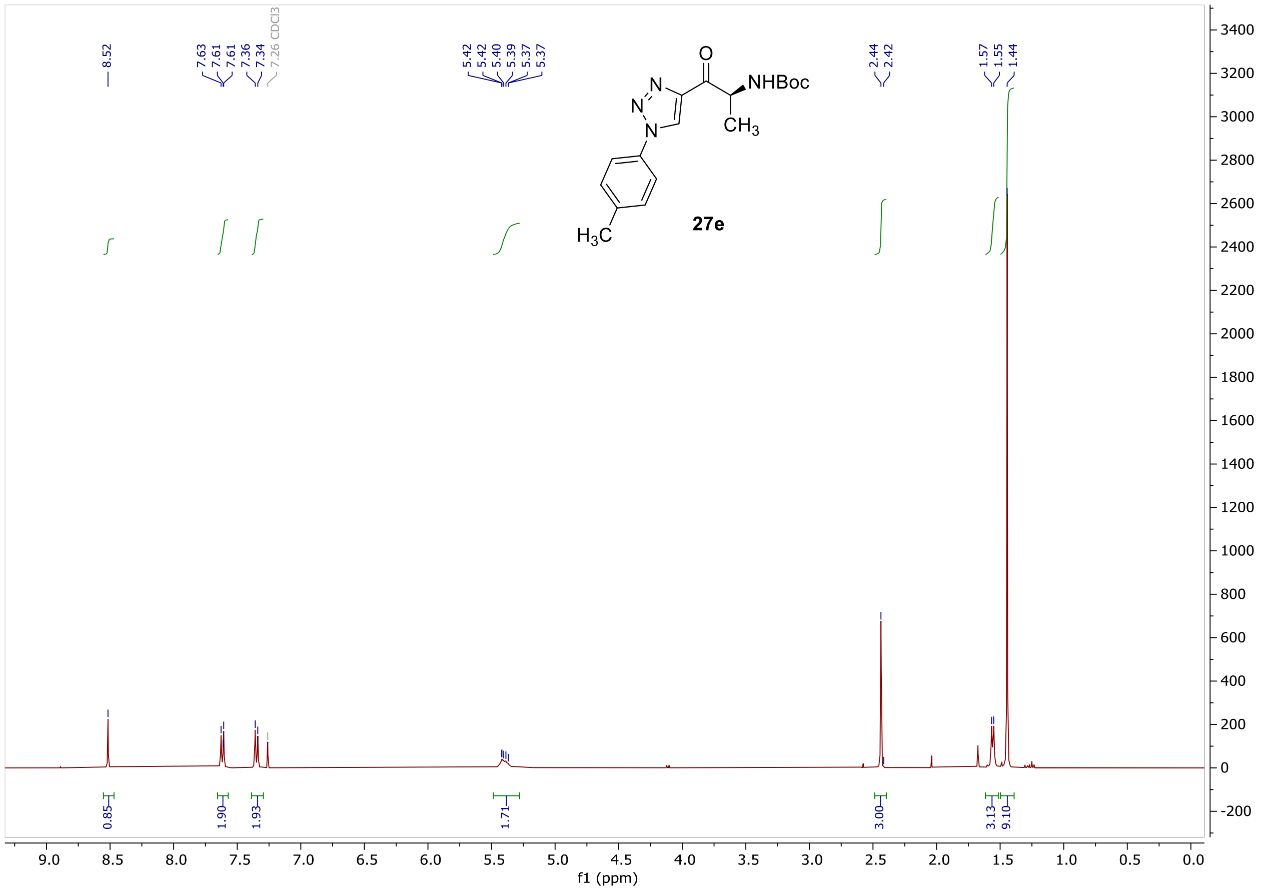

Compound **27e** (^{13}C NMR, 101 MHz):

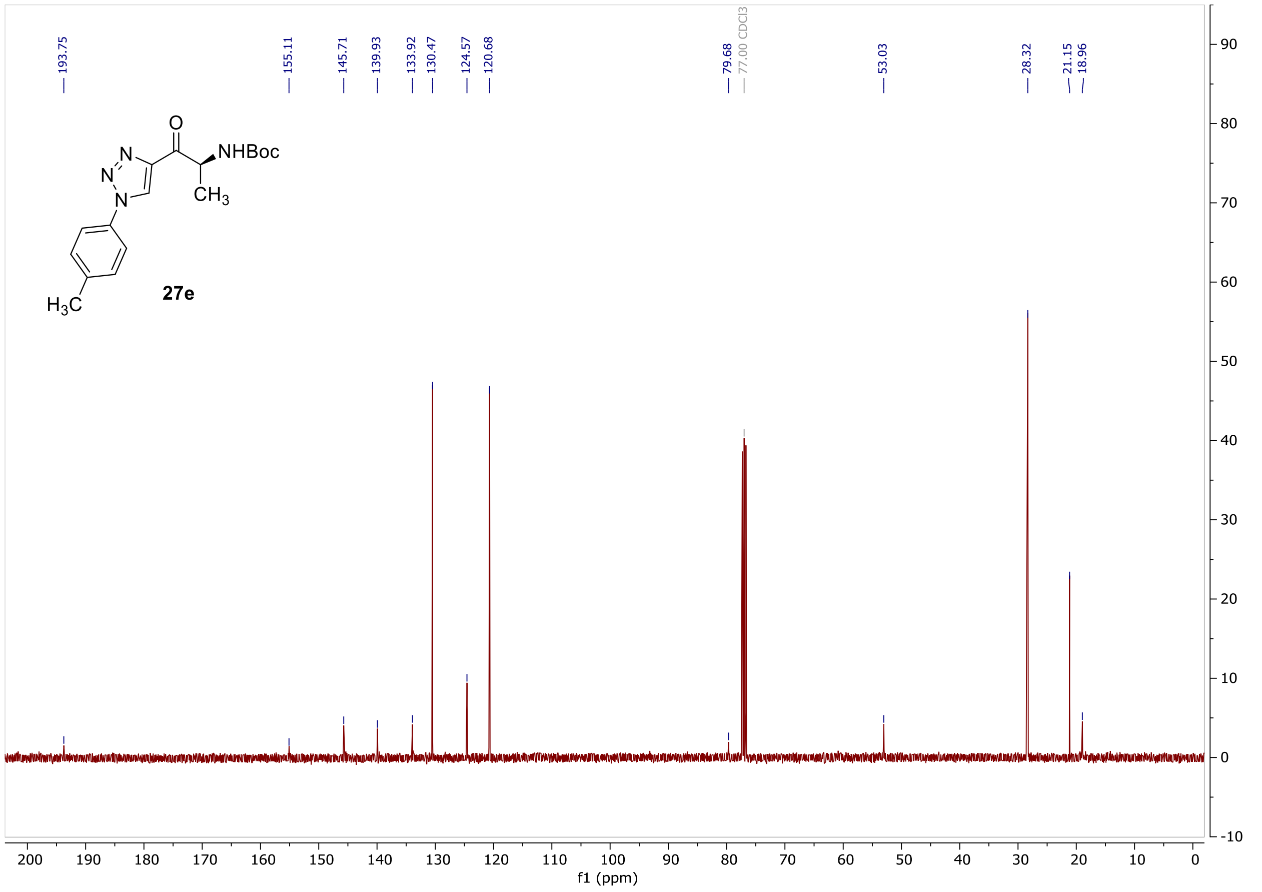

Compound **27f** (^{1}H NMR, 400 MHz):

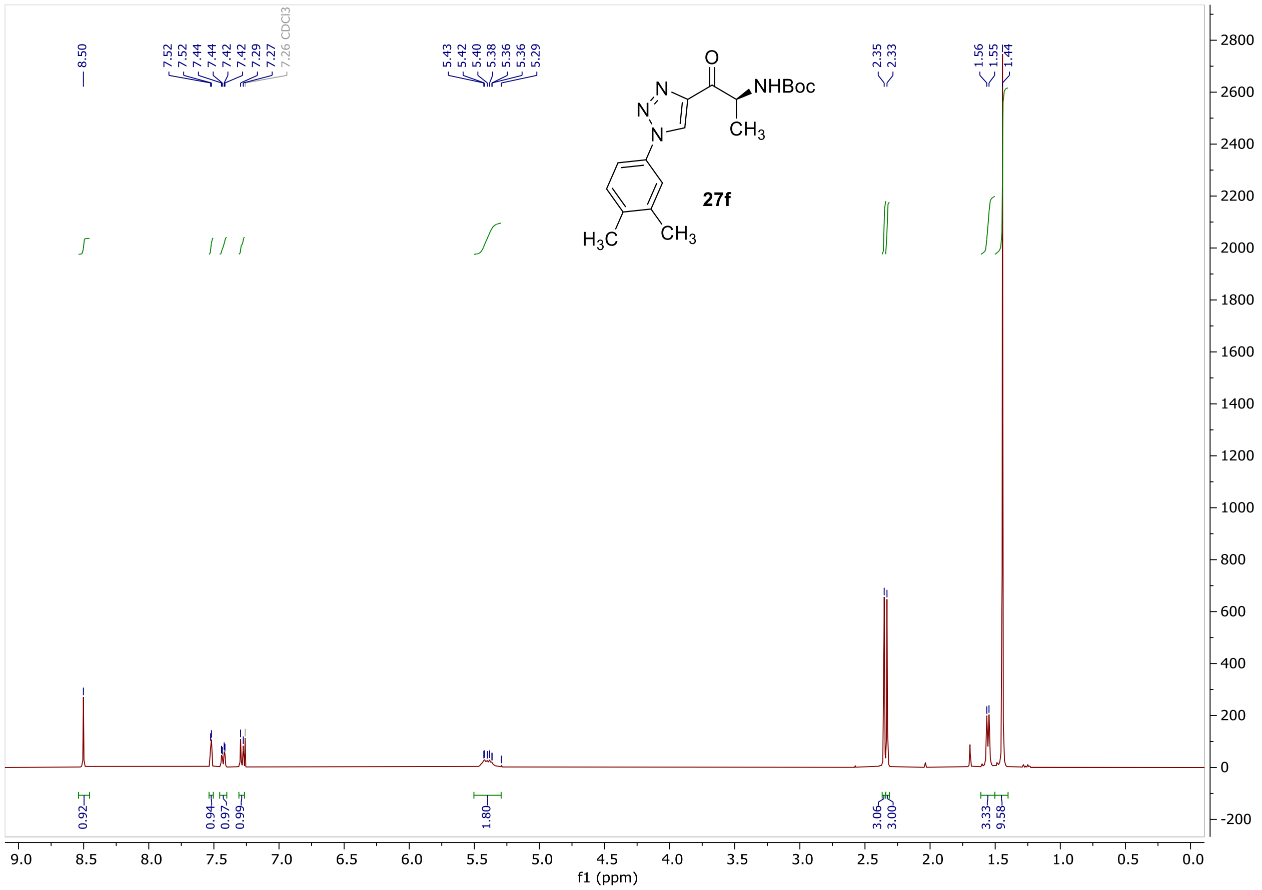

Compound **27f** (^{13}C NMR, 101 MHz):

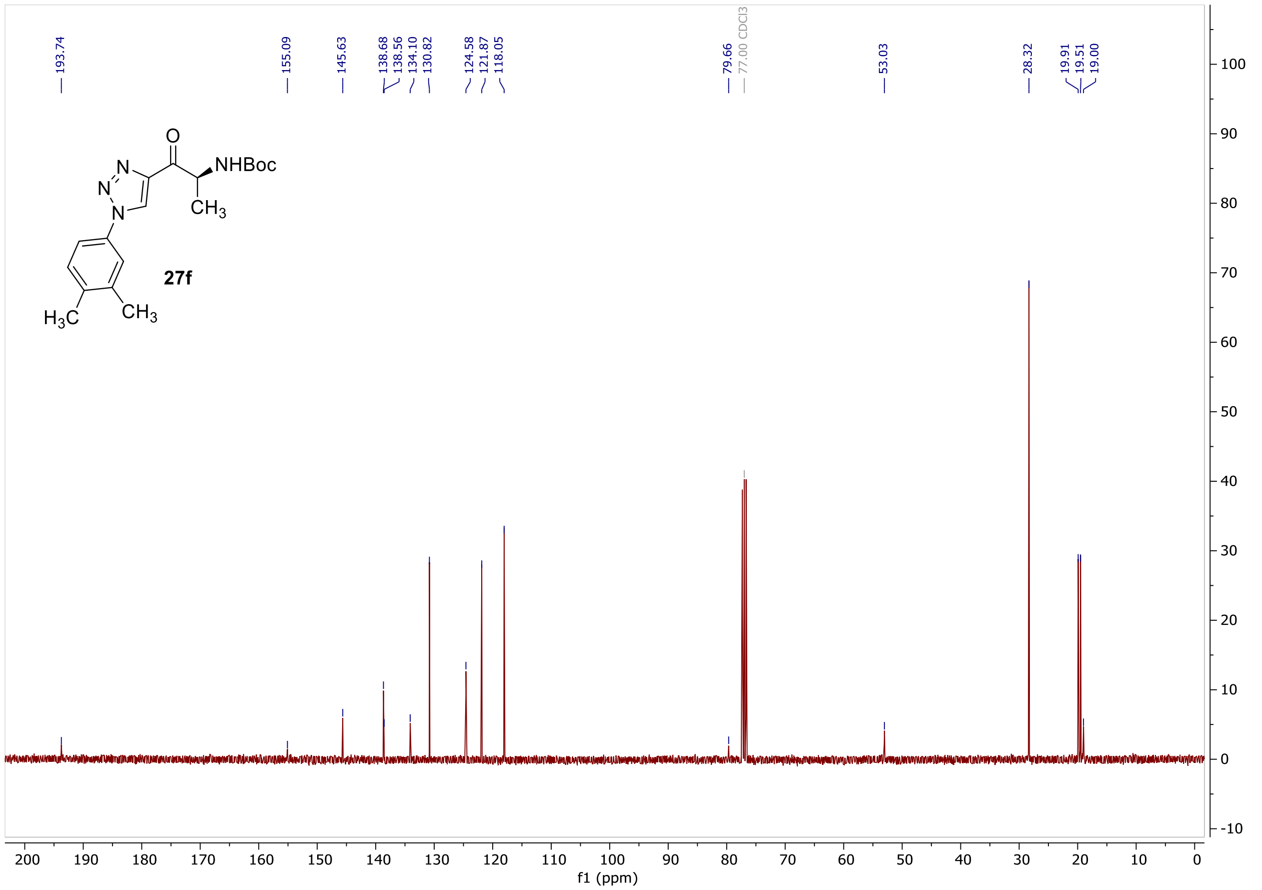

Compound **27g** (^{1}H NMR, 400 MHz):

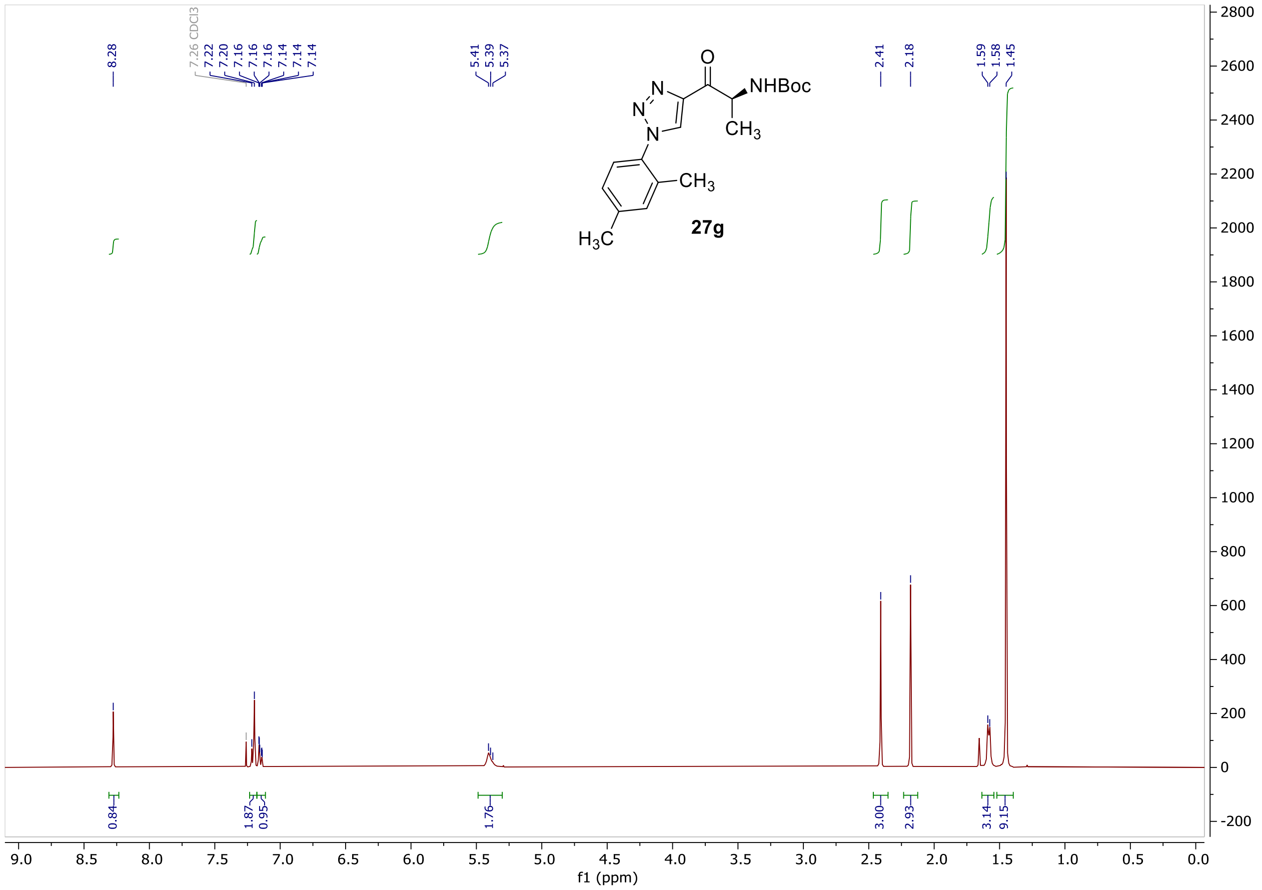

Compound **27g** (^{13}C NMR, 101 MHz):

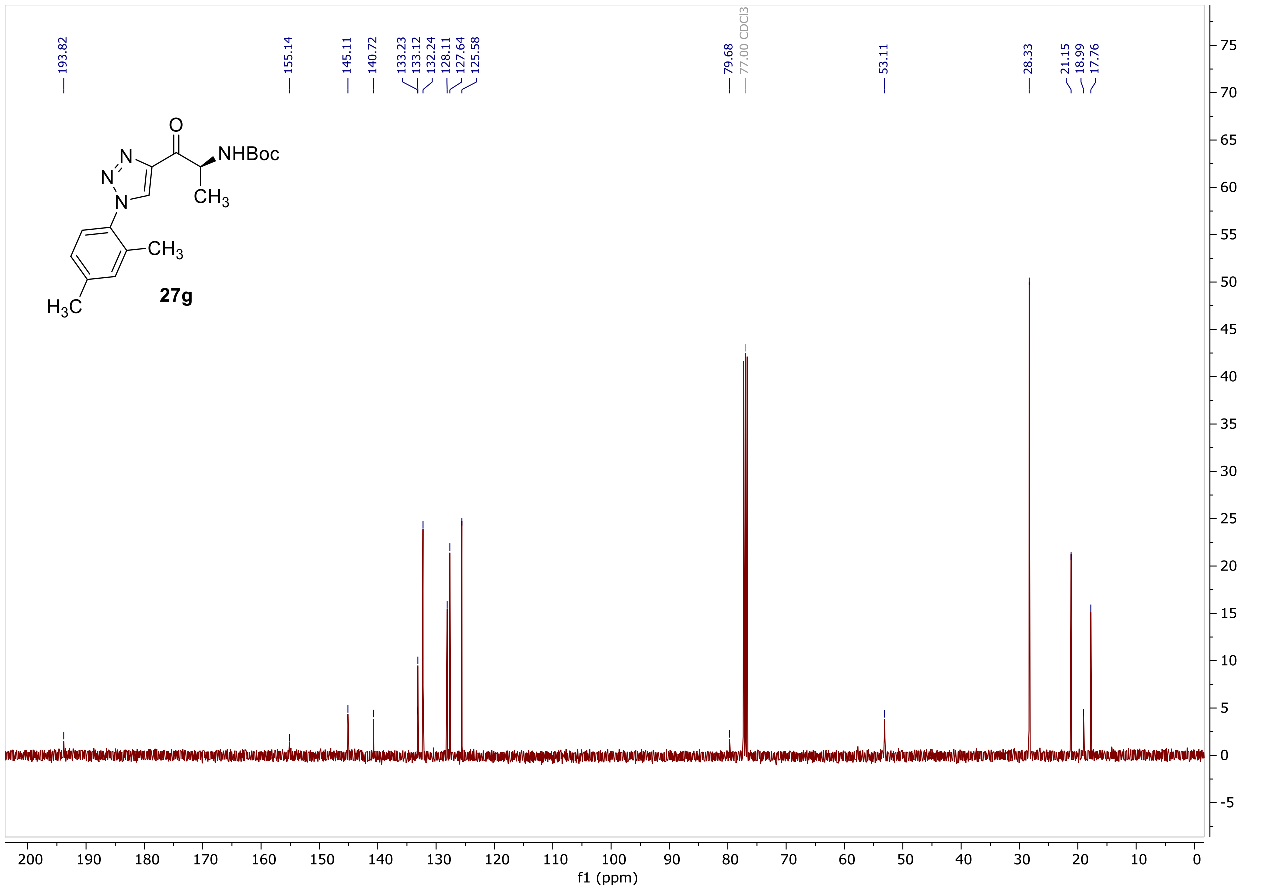

Compound **27h** (^{1}H NMR, 400 MHz):

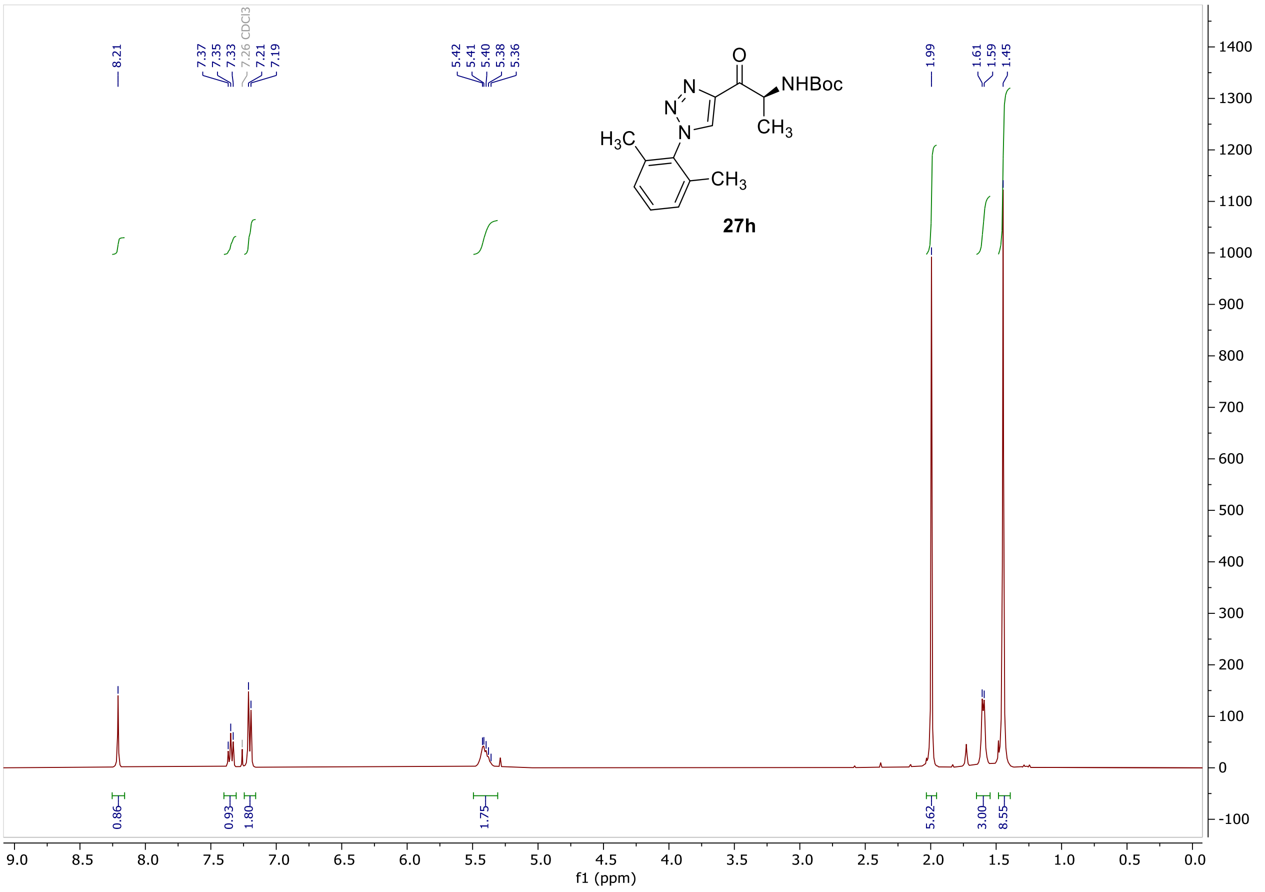

Compound **27h** (^{13}C NMR, 101 MHz):

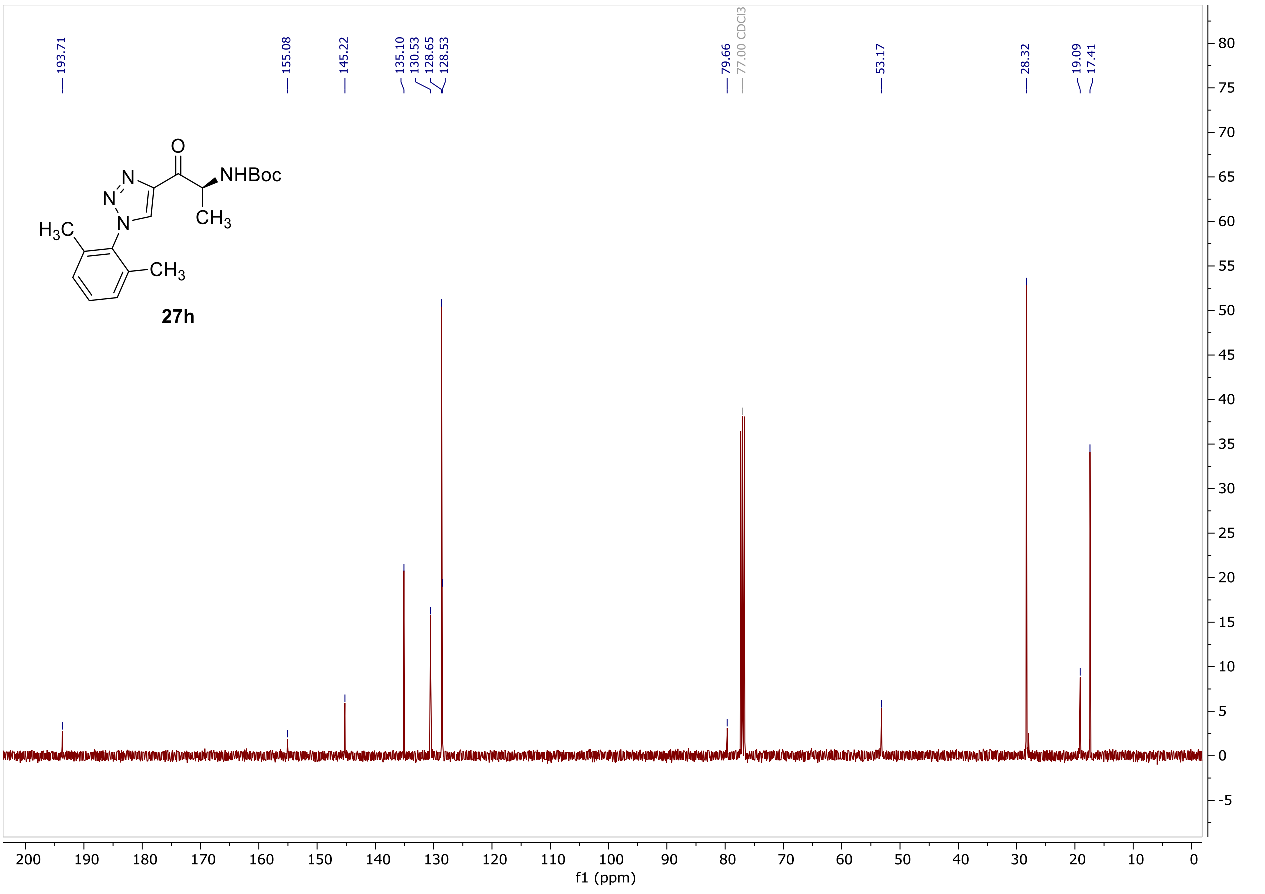

Compound **27i** (^{1}H NMR, 400 MHz):

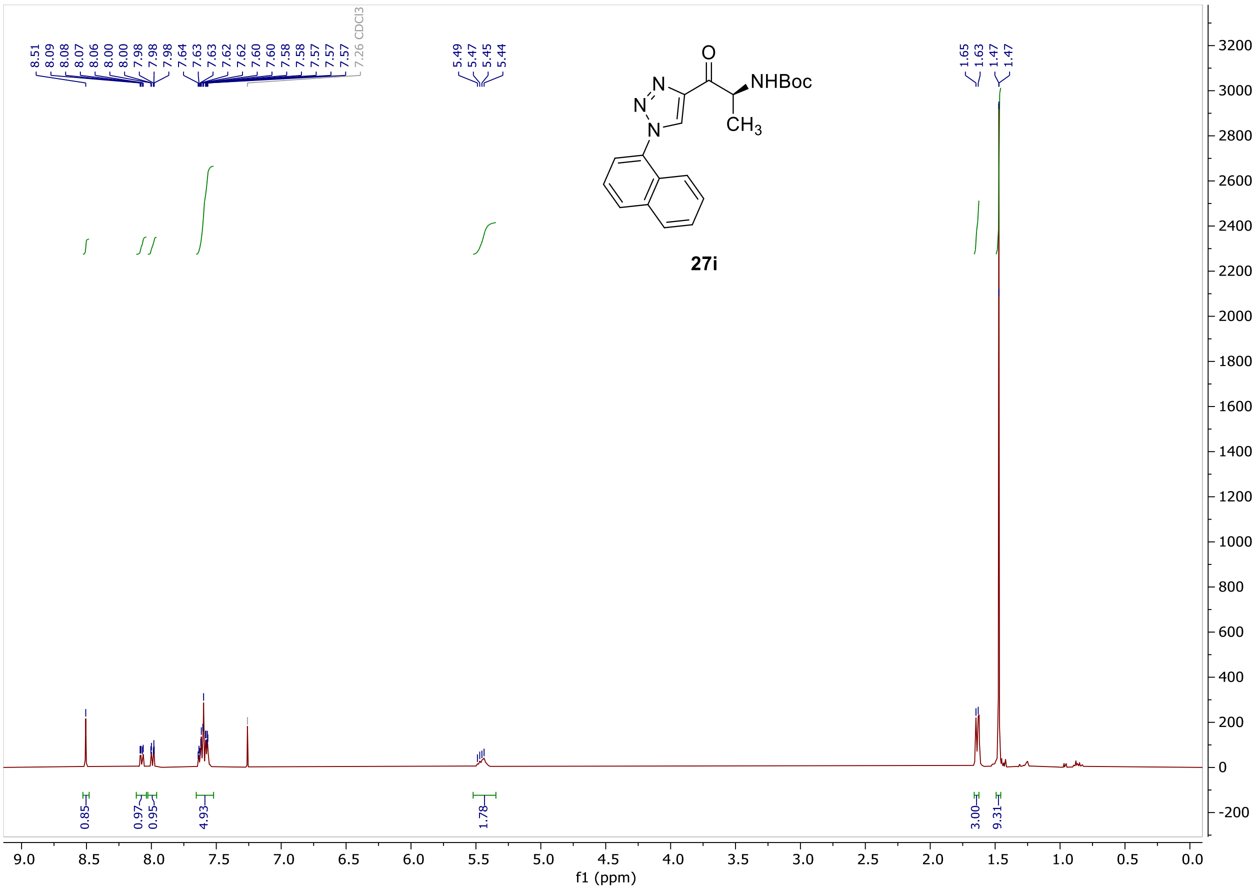

Compound **27i** (^{13}C NMR, 101 MHz):

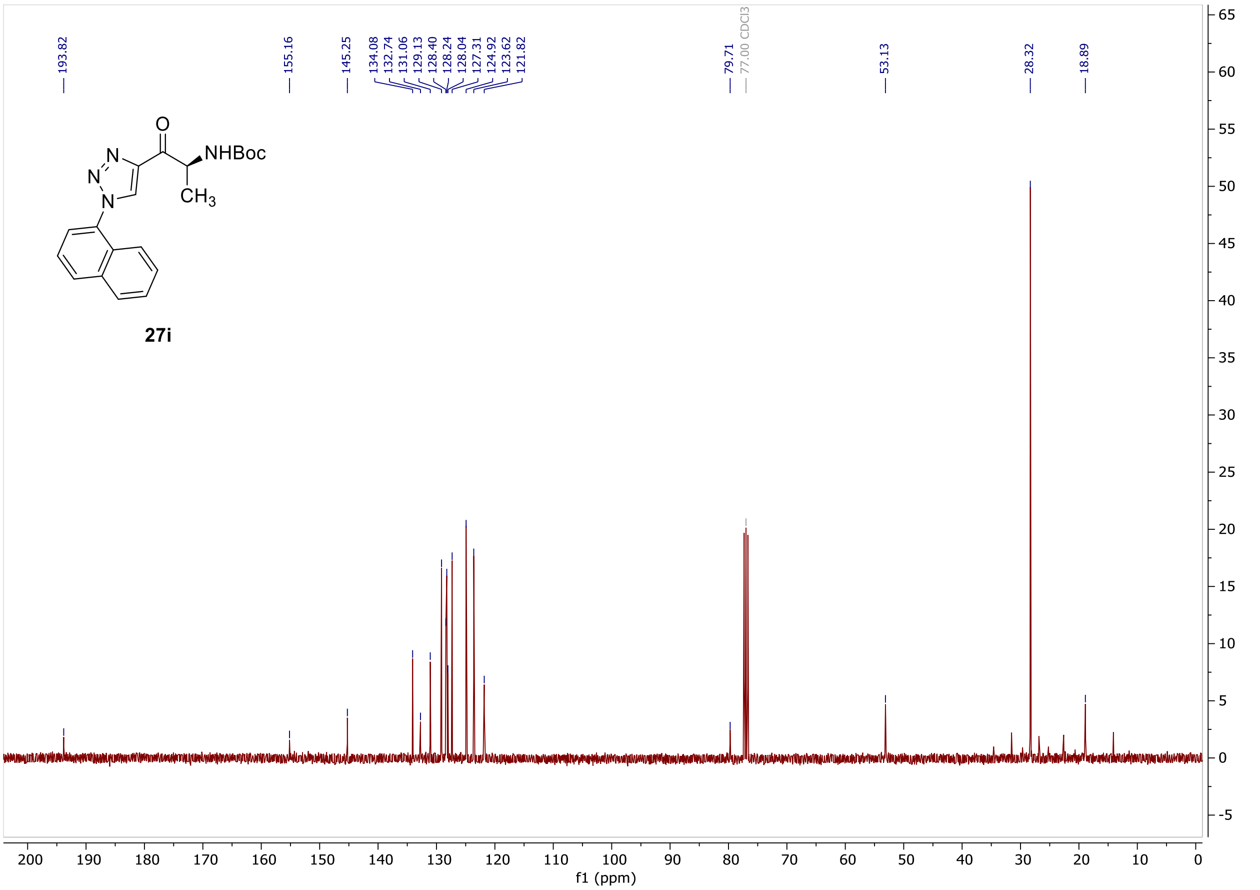

Compound **27j** (^{1}H NMR, 400 MHz):

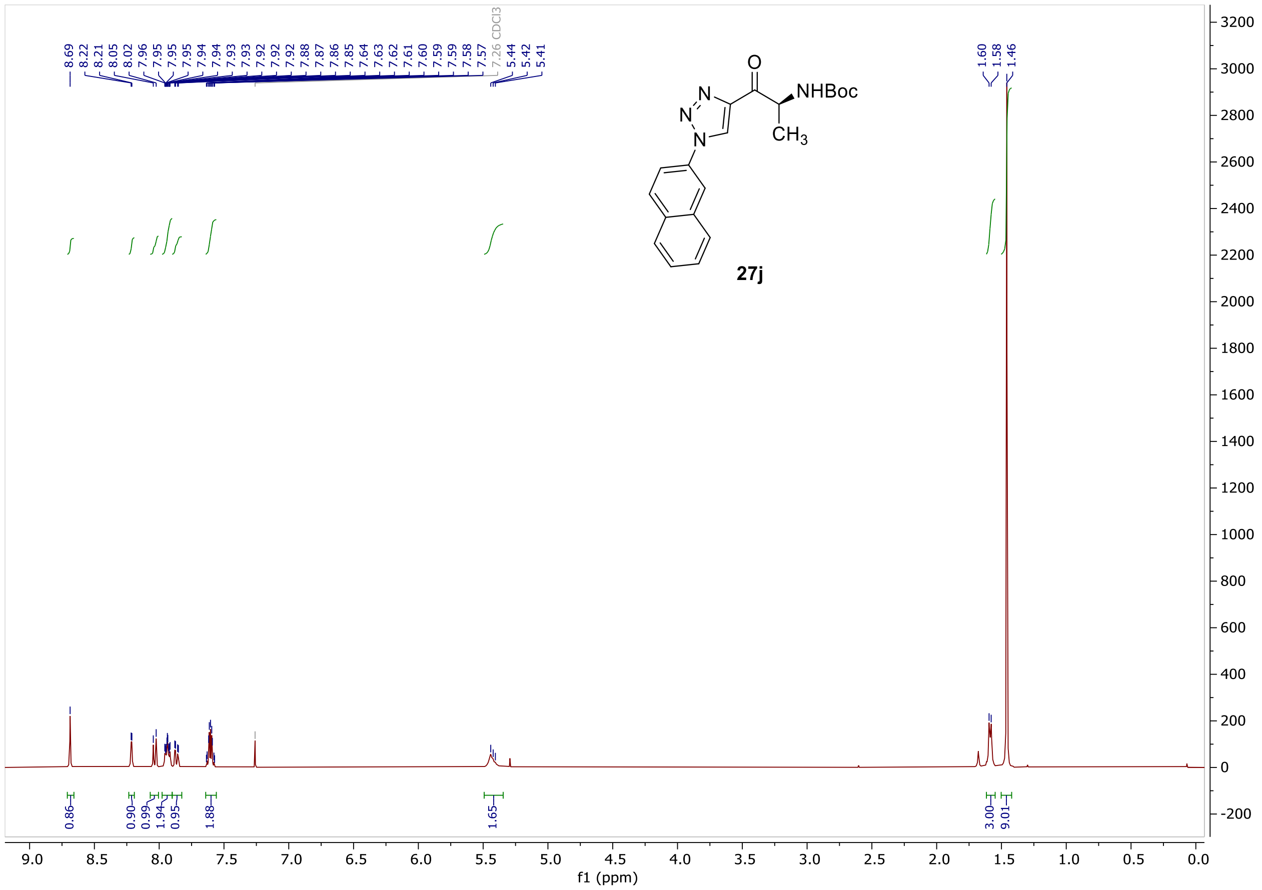

Compound **27j** (^{13}C NMR, 101 MHz):

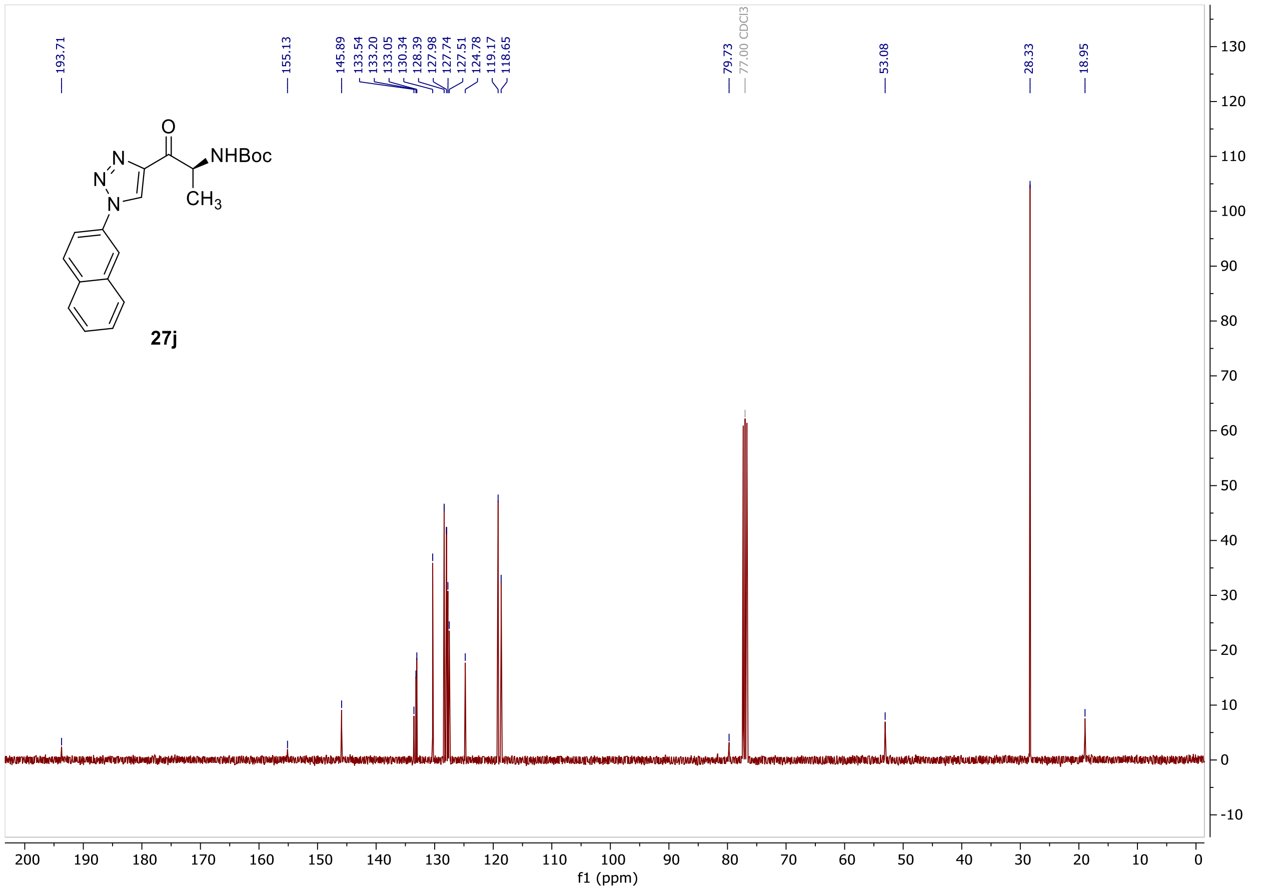

Compound **27k** (^{1}H NMR, 400 MHz):

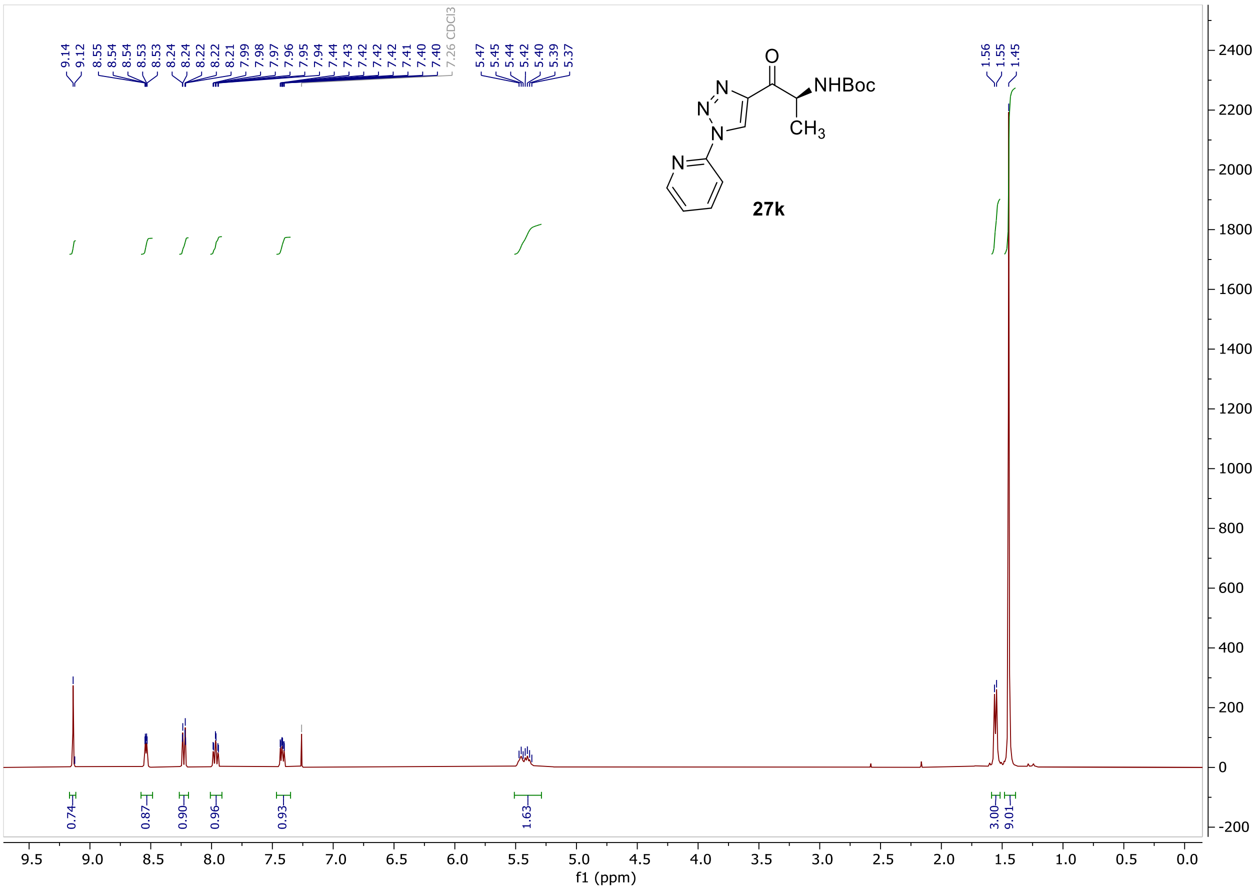

Compound **27k** (^{13}C NMR, 101 MHz):

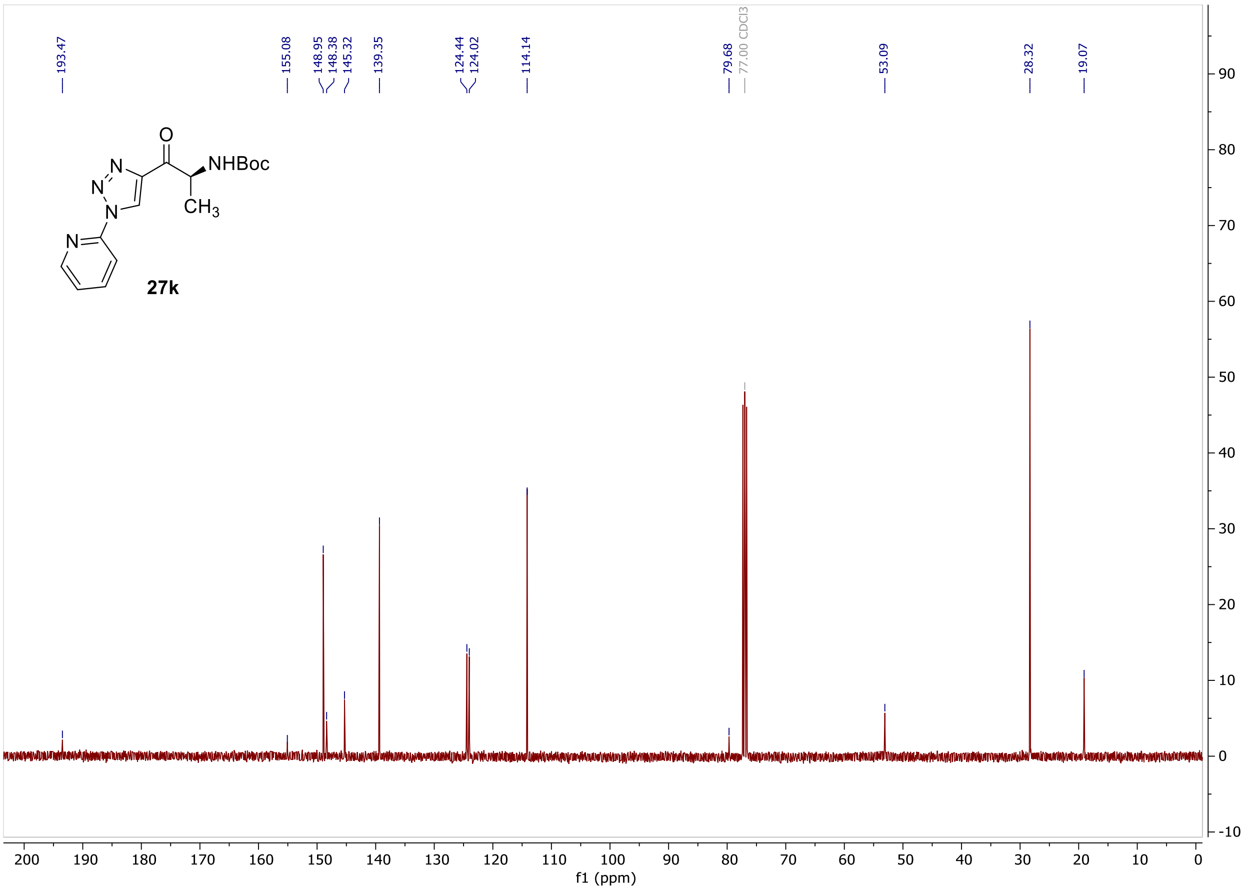

Compound **27l** (^{1}H NMR, 400 MHz):

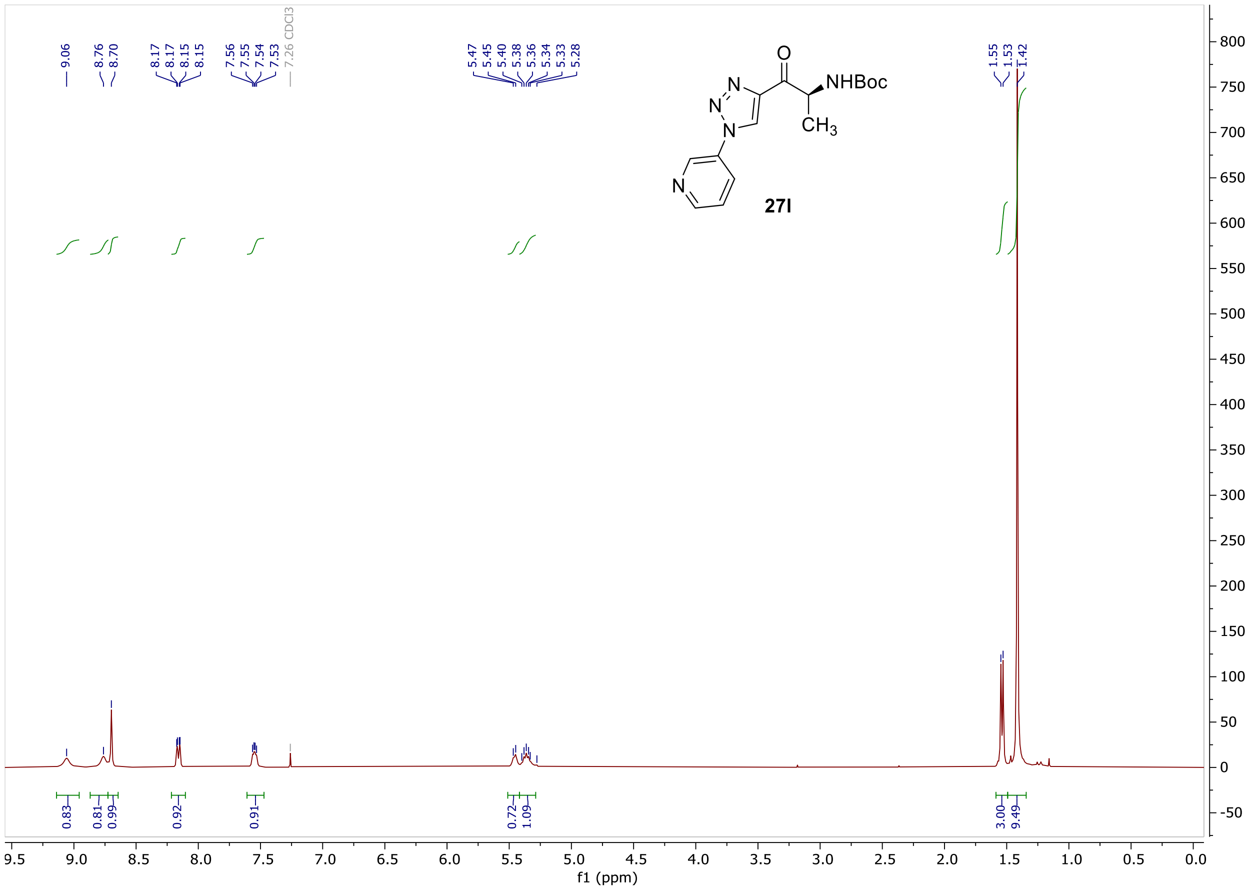

Compound **27l** (^{13}C NMR, 101 MHz):

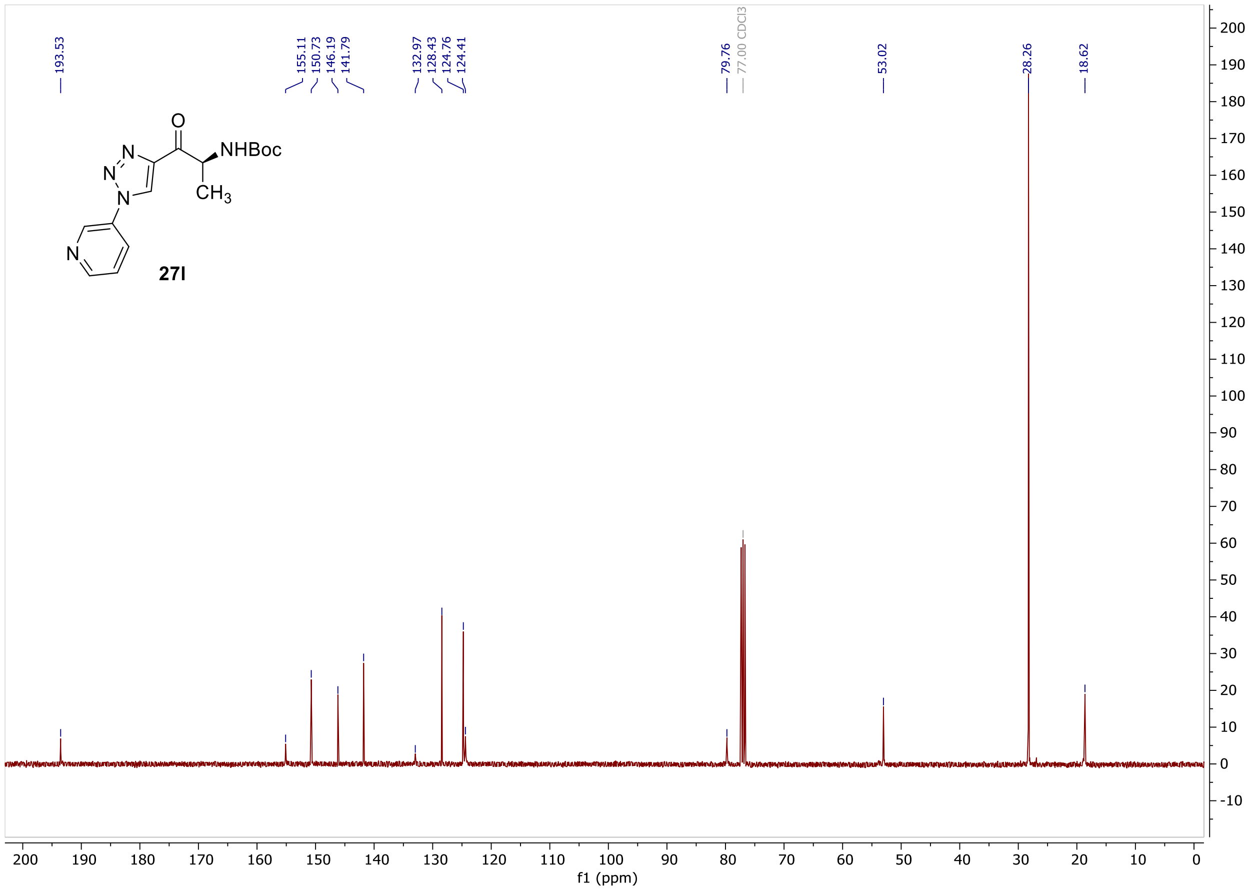

Compound **27m** (^{1}H NMR, 400 MHz):

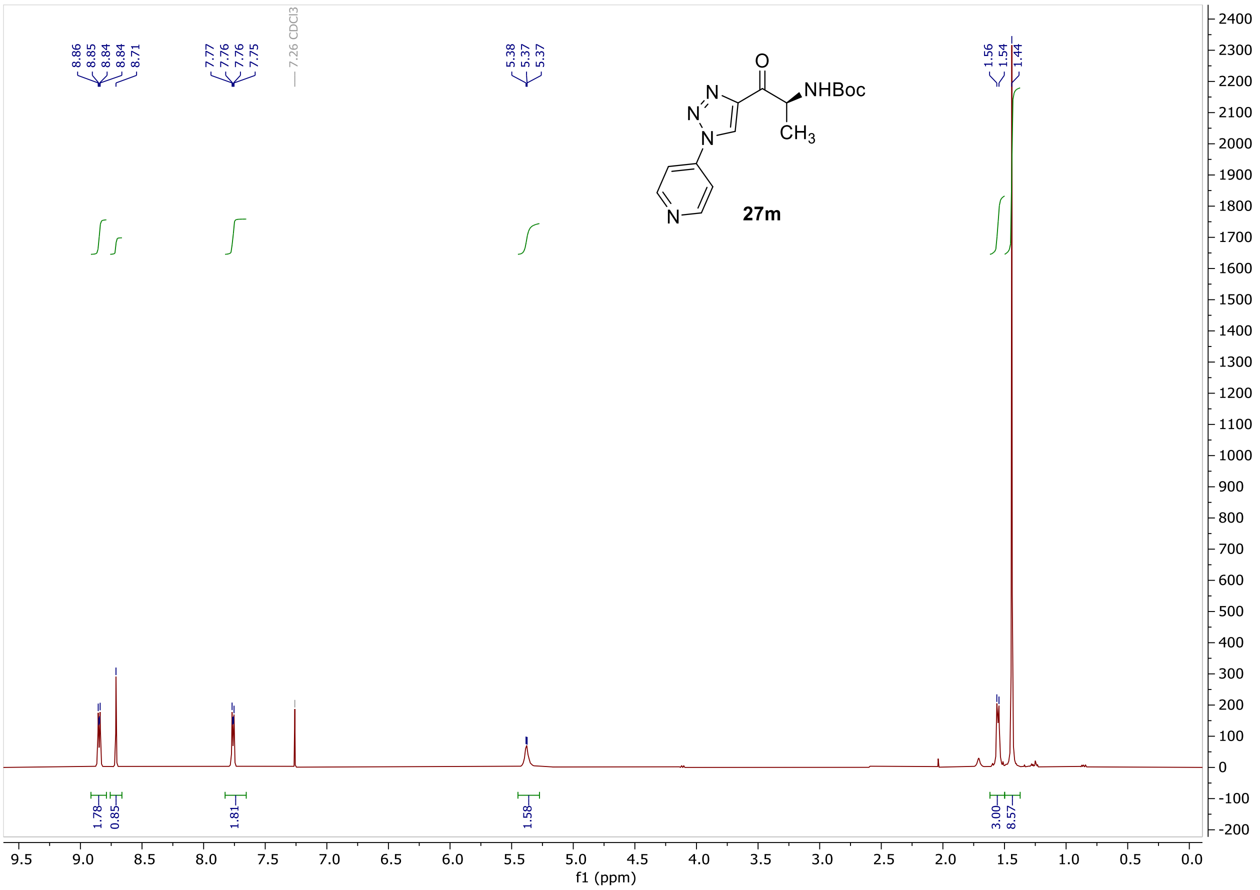

Compound **27m** (^{13}C NMR, 101 MHz):

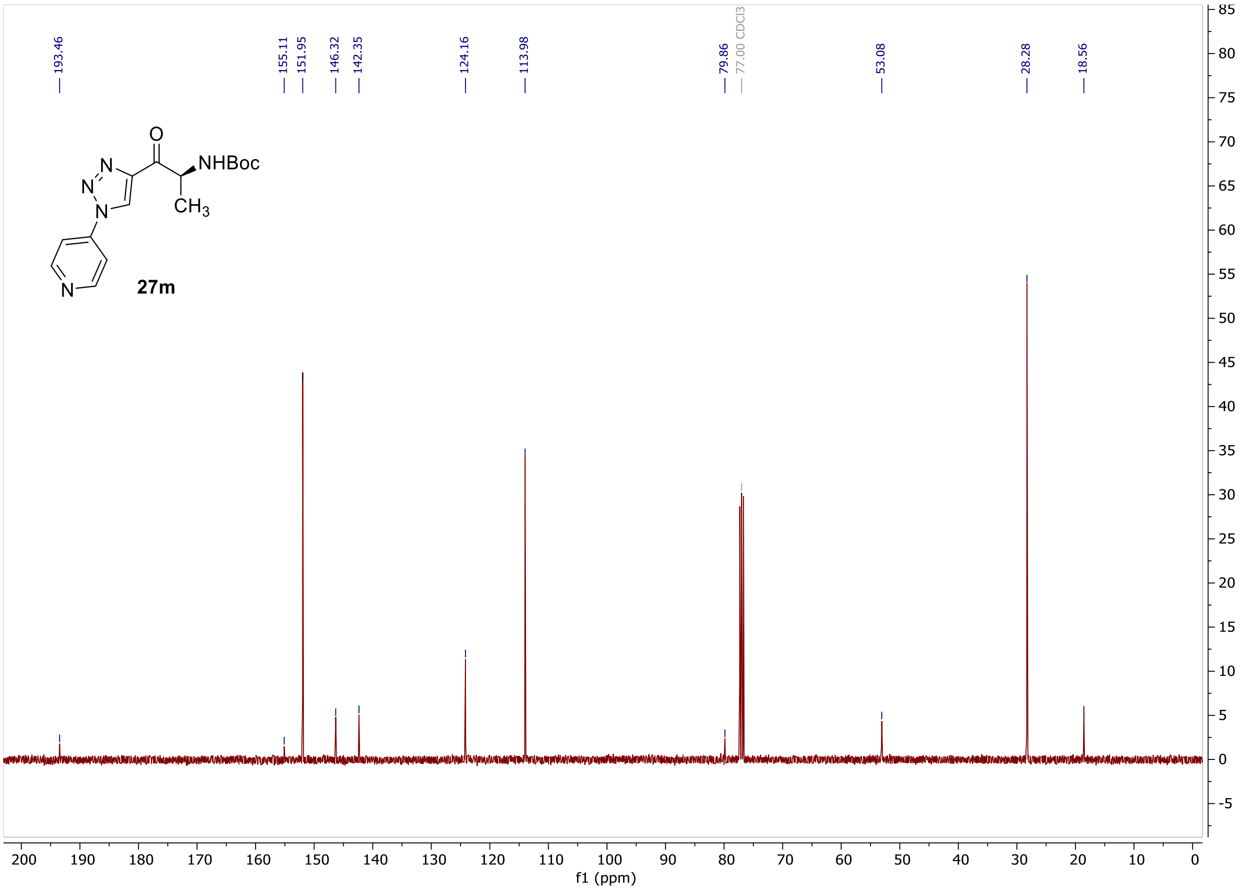

Compound **27n** (^{1}H NMR, 400 MHz):

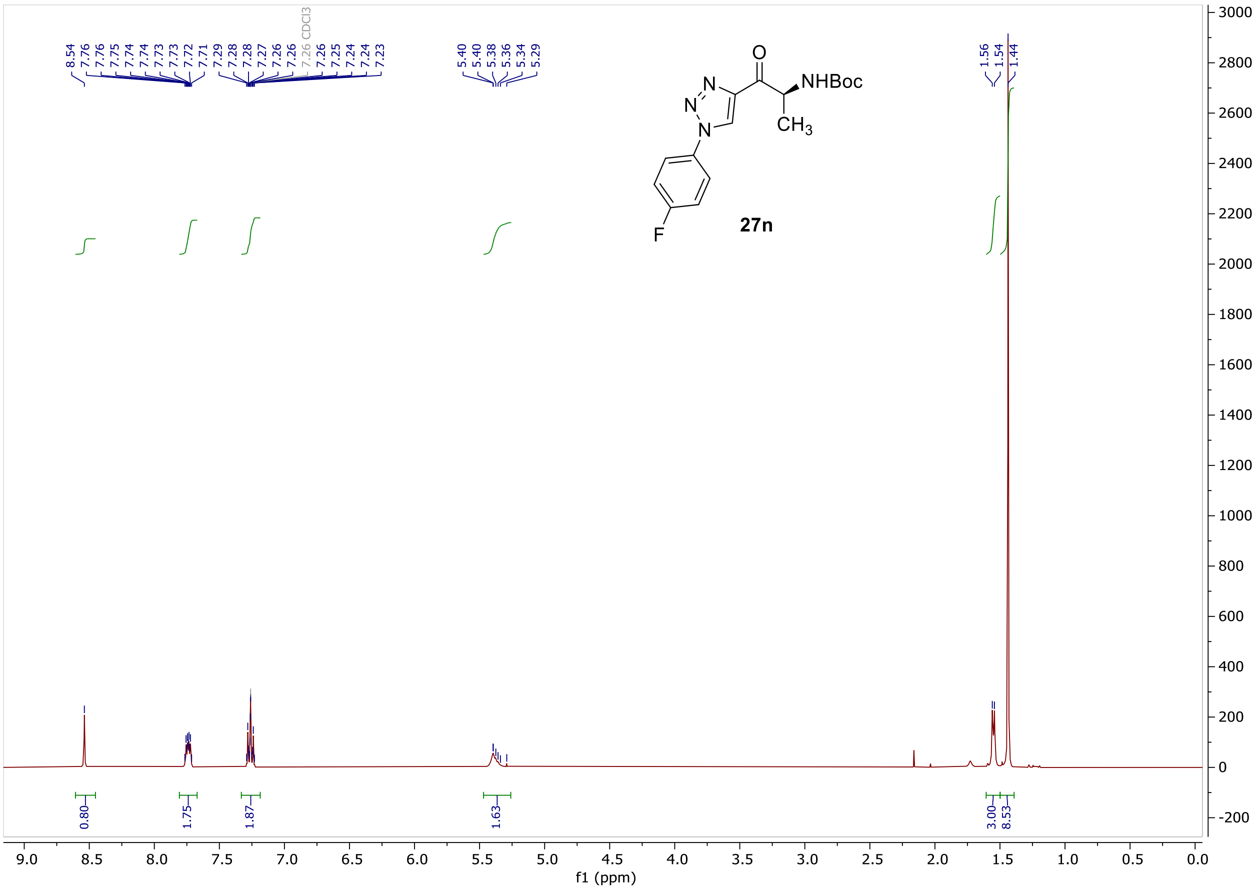

Compound **27n** (^{13}C NMR, 101 MHz):

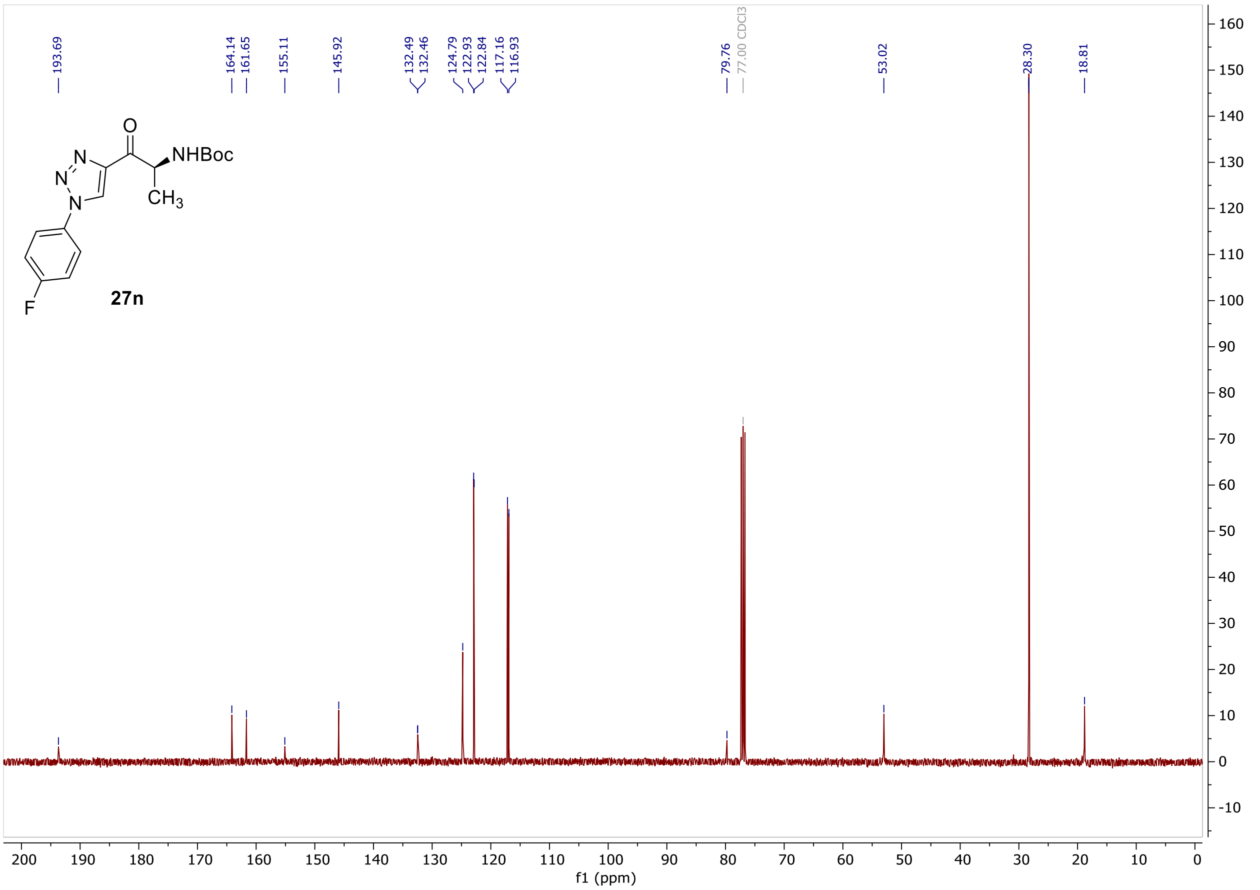

Compound **27n** (^{19}F NMR, 282 MHz):

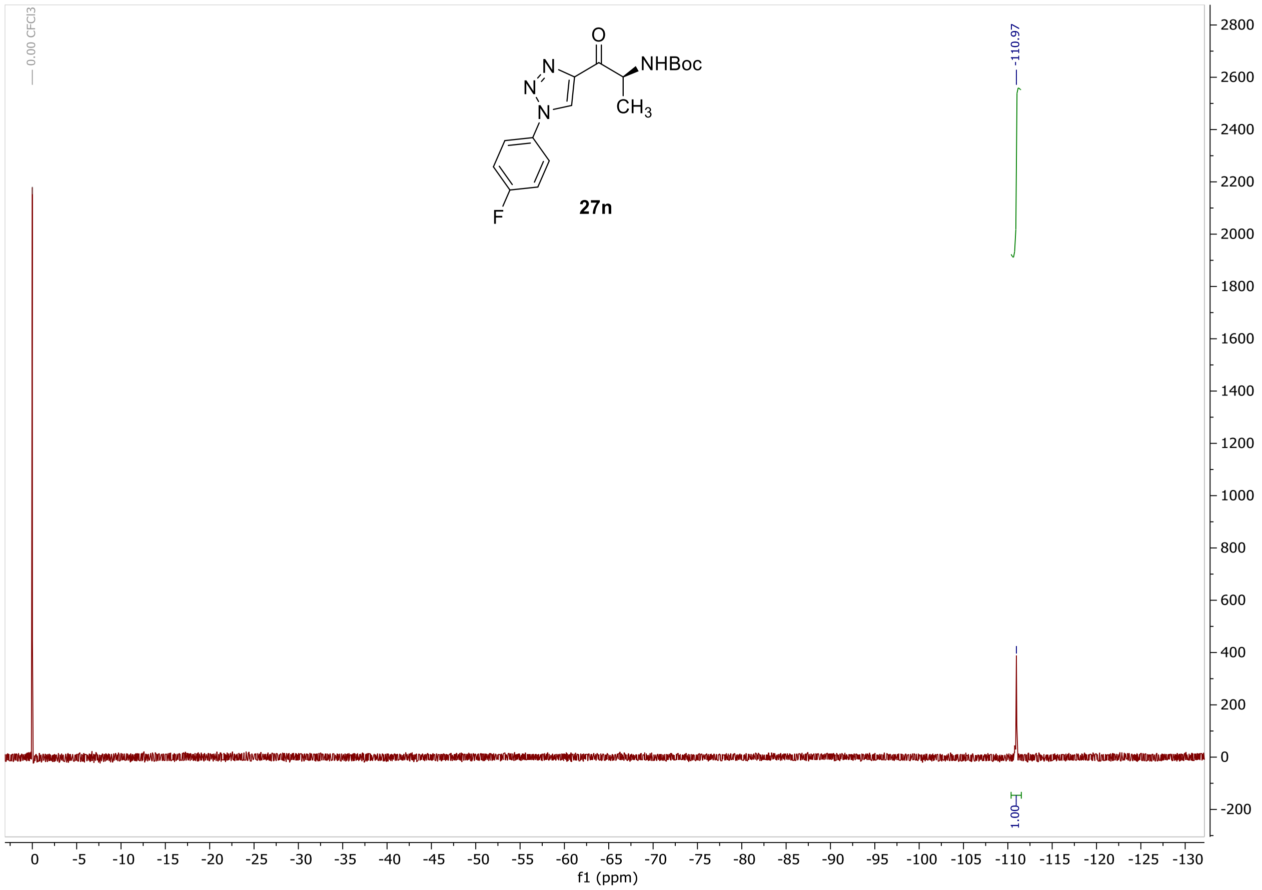

Compound **27o** (^{1}H NMR, 400 MHz):

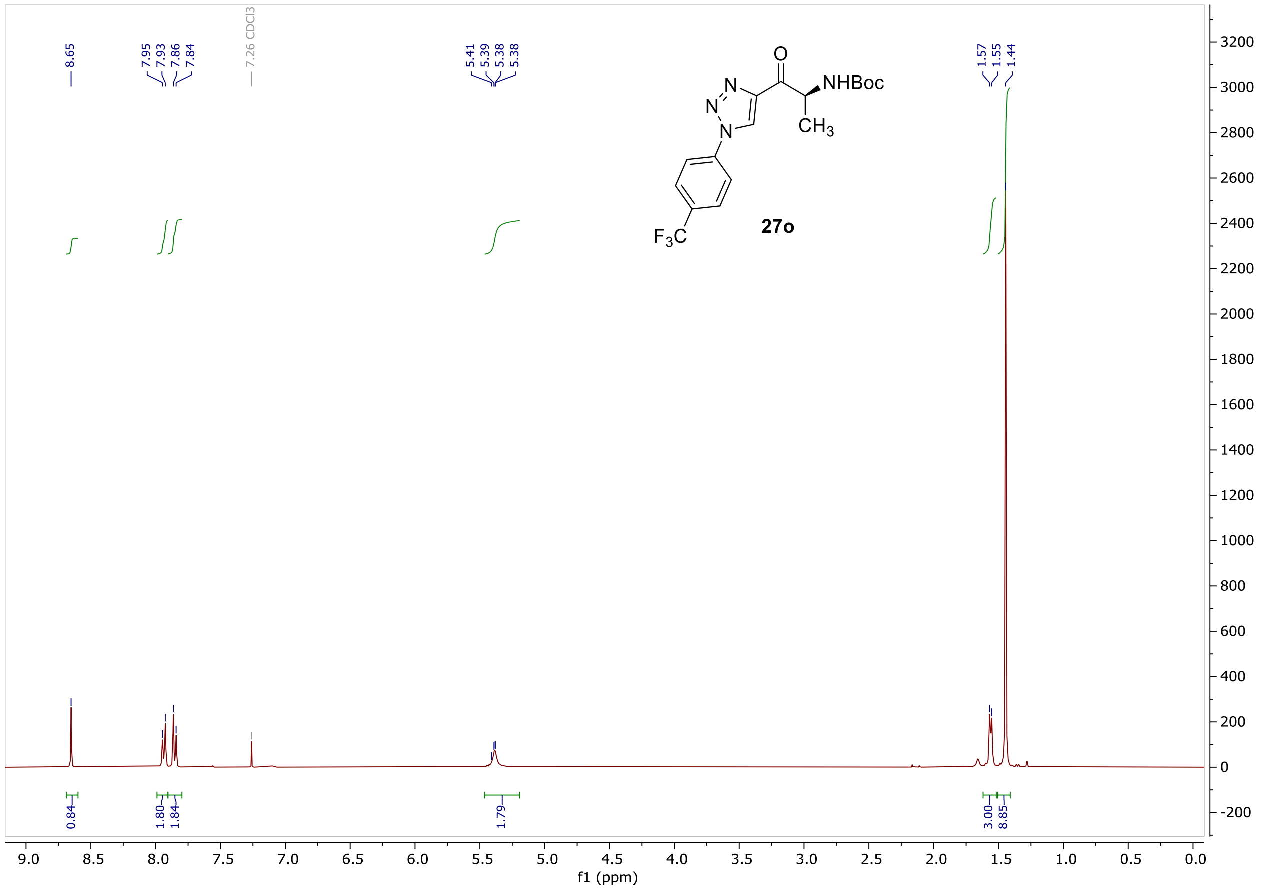

Compound **27o** (^{13}C NMR, 101 MHz):

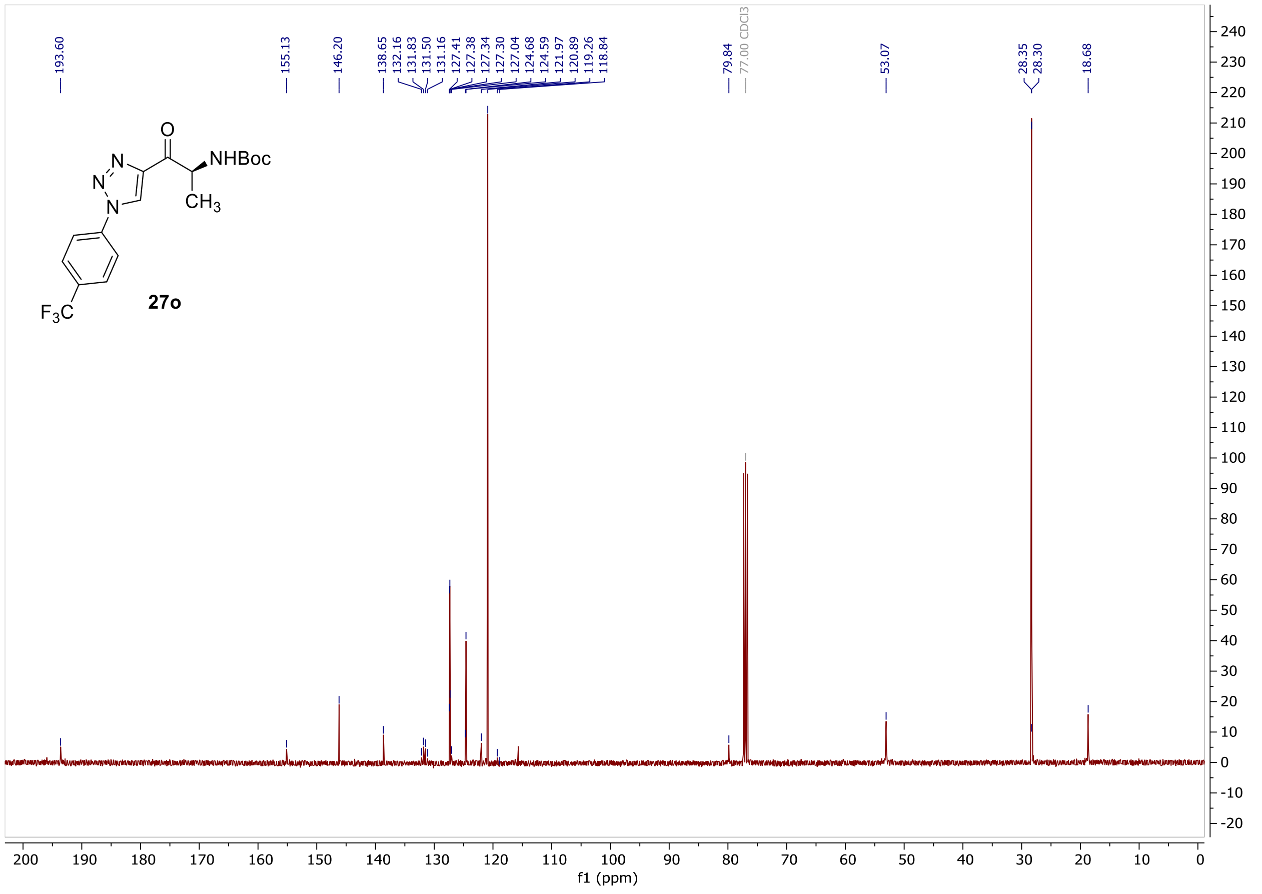

Compound **27o** (^{19}F NMR, 282 MHz):

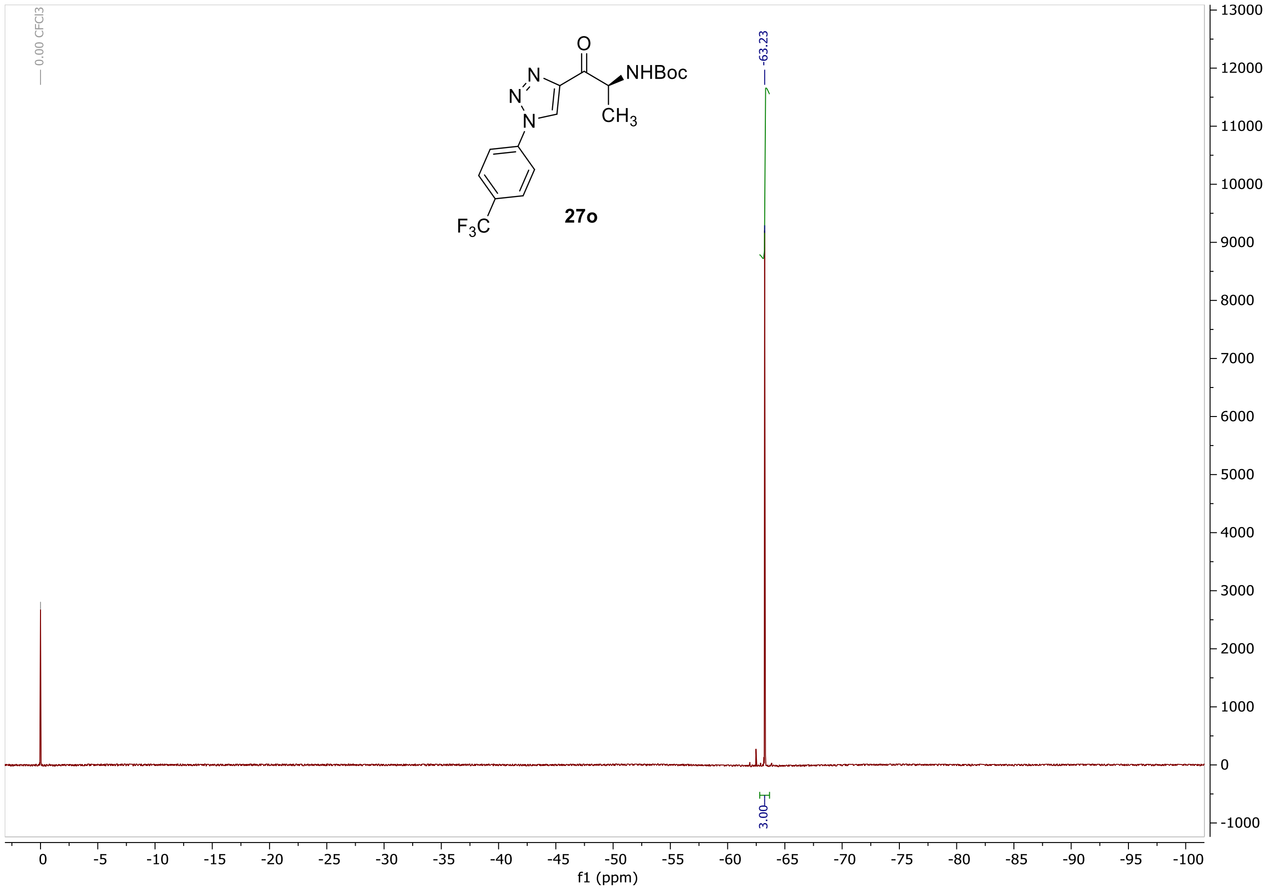

Compound **27p** (^1H NMR, 400 MHz):

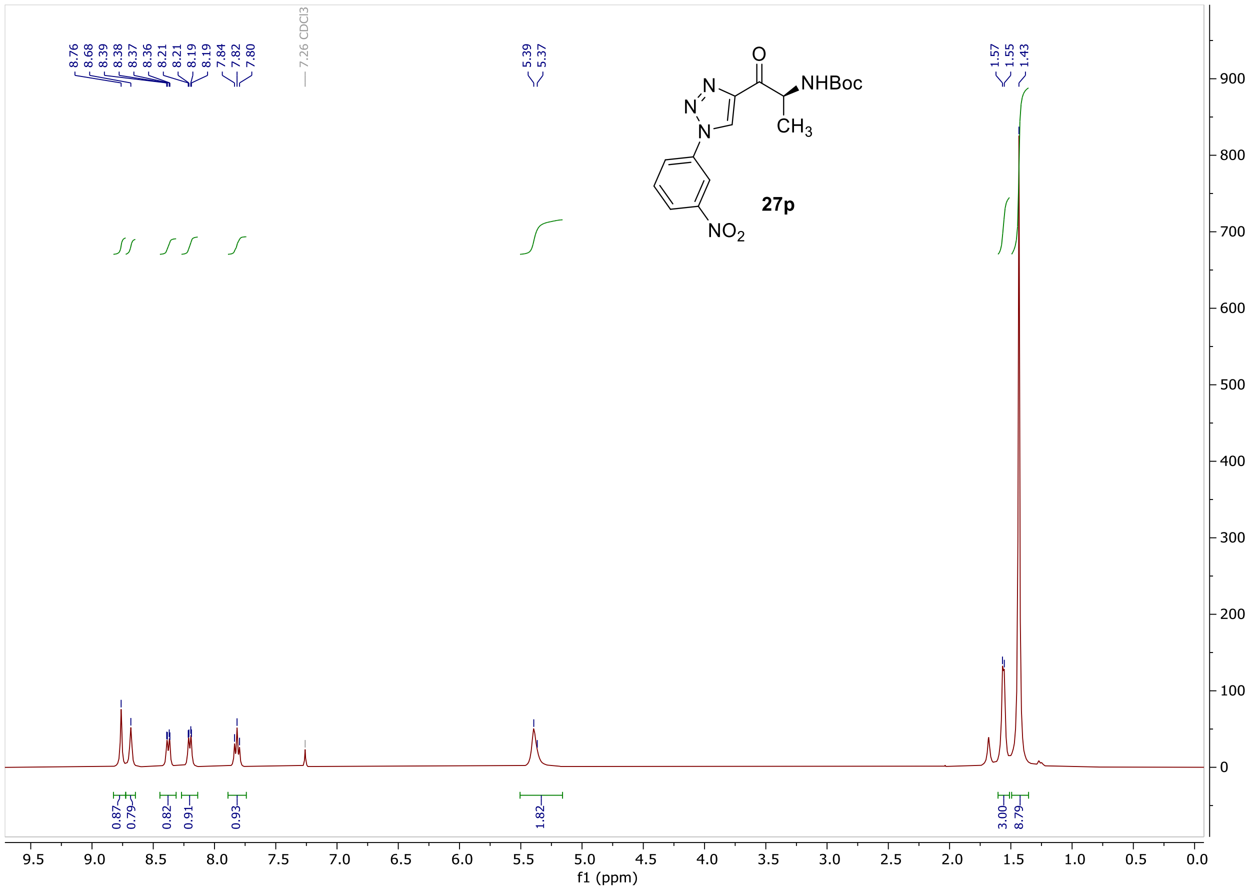

Compound **27p** (^{13}C NMR, 101 MHz):

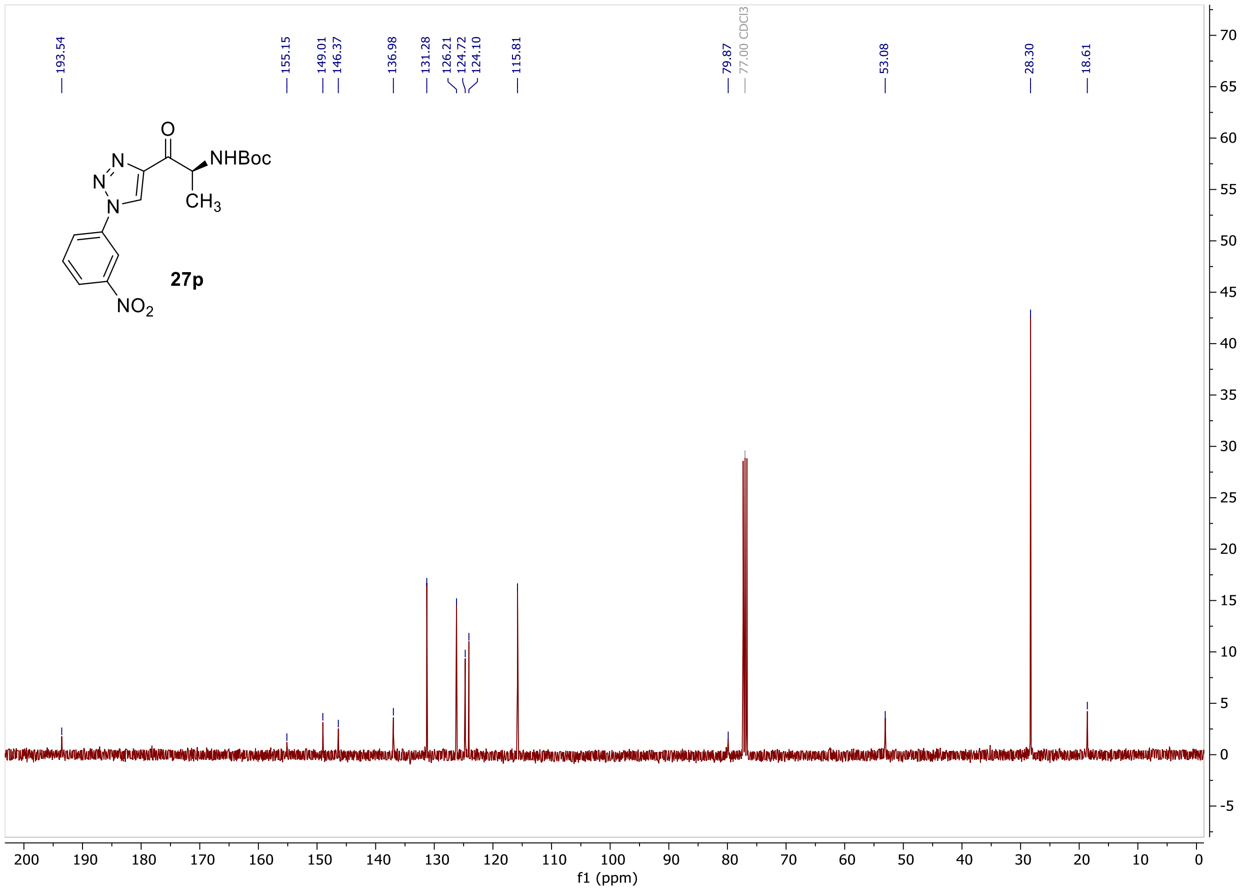

Compound **27q** (^{1}H NMR, 400 MHz):

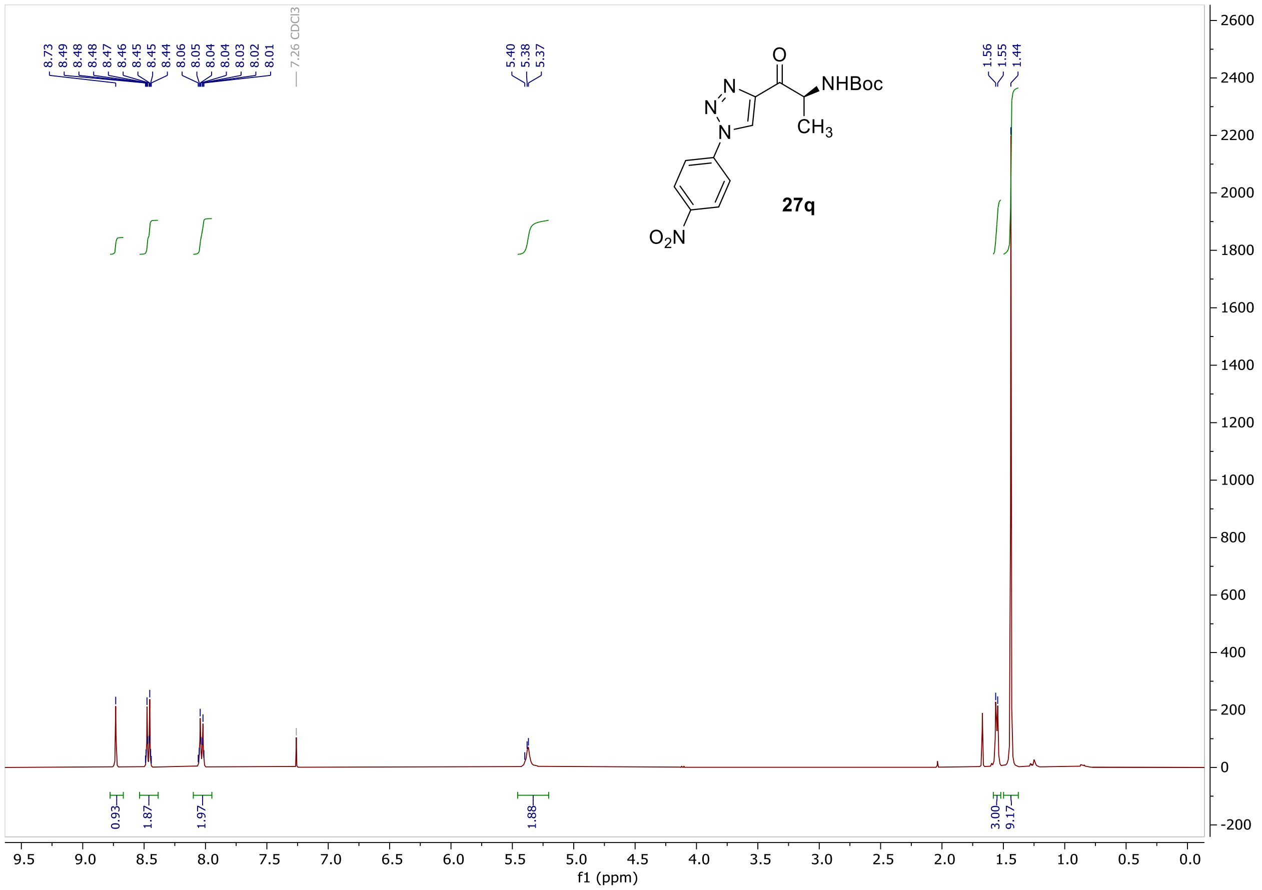

Compound **27q** (^{13}C NMR, 101 MHz):

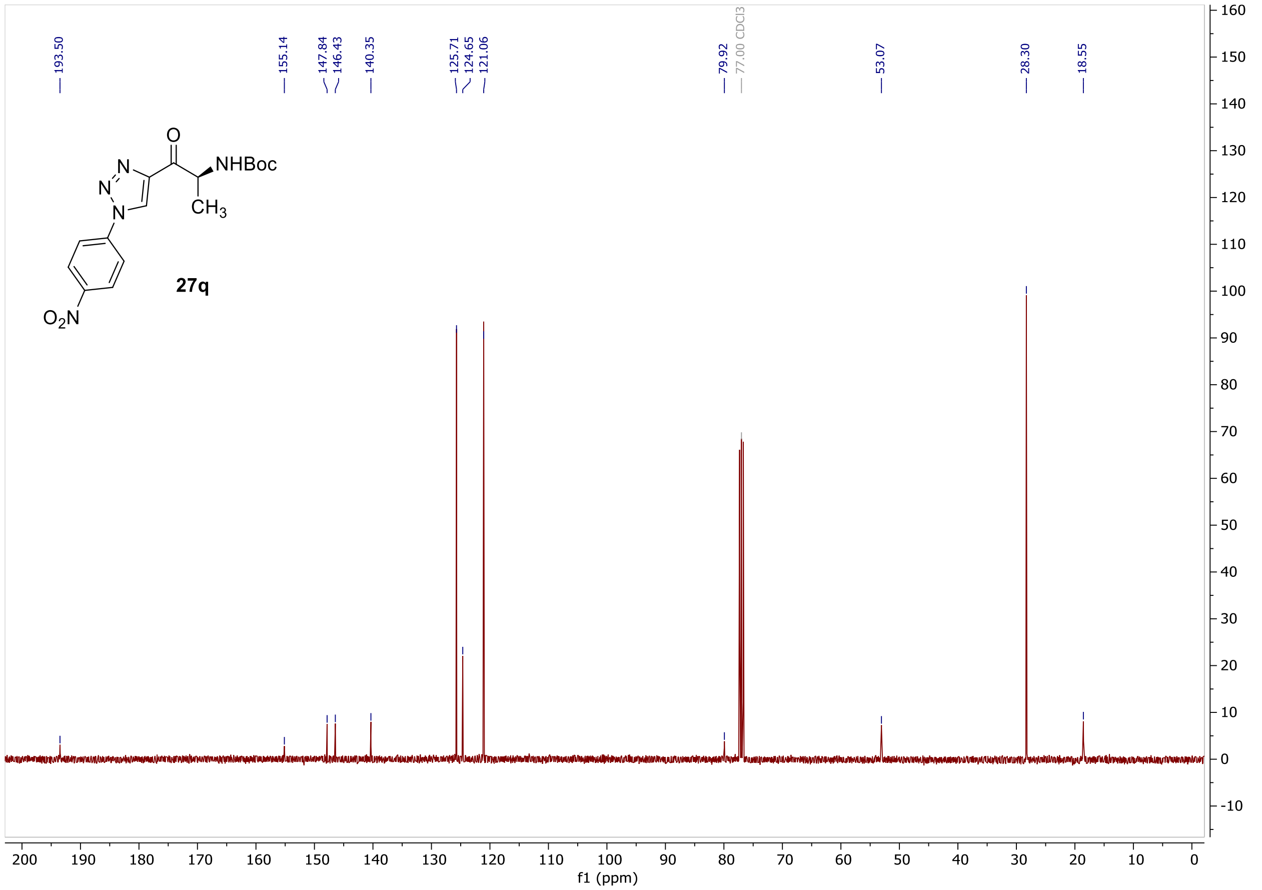

Compound **27r** (^{1}H NMR, 400 MHz):

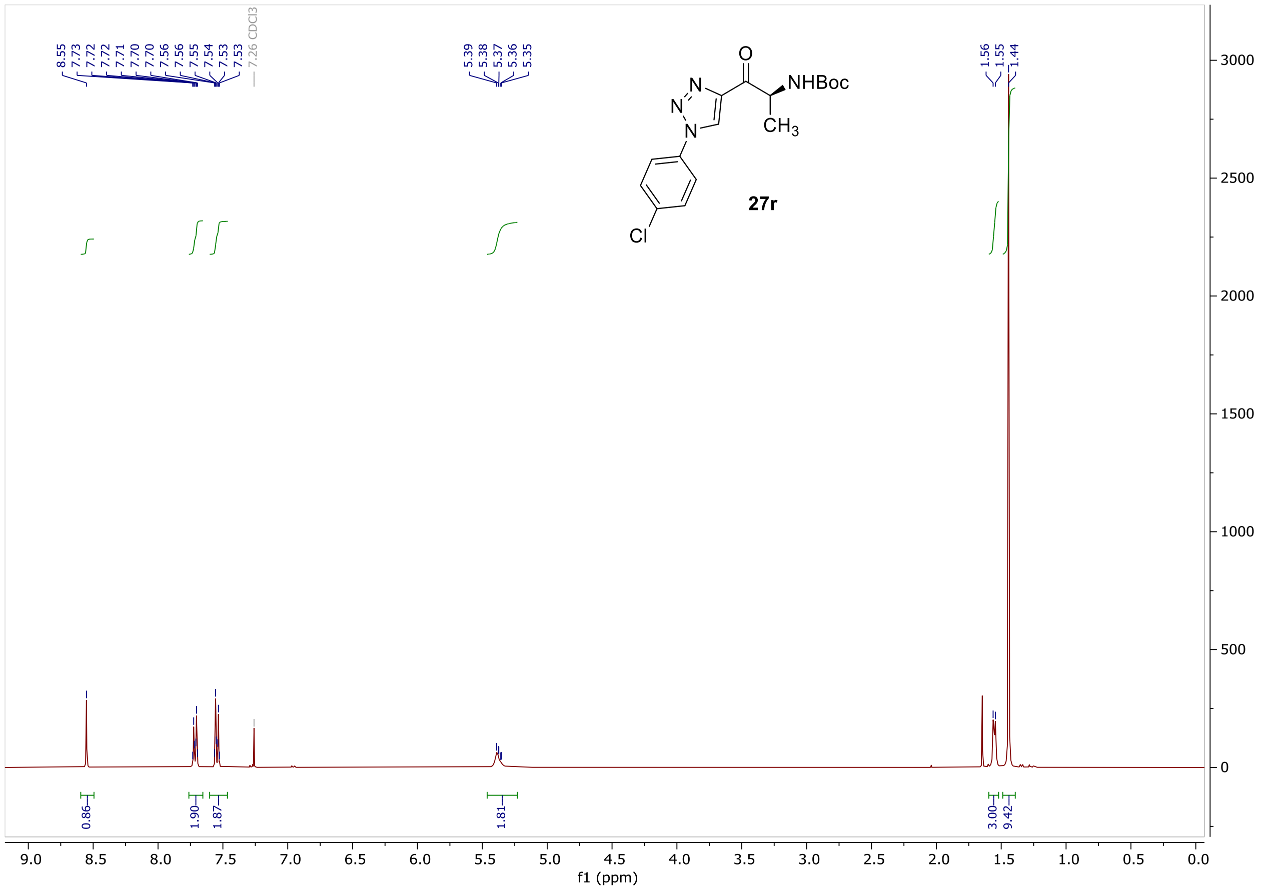

Compound **27r** (^{13}C NMR, 101 MHz):

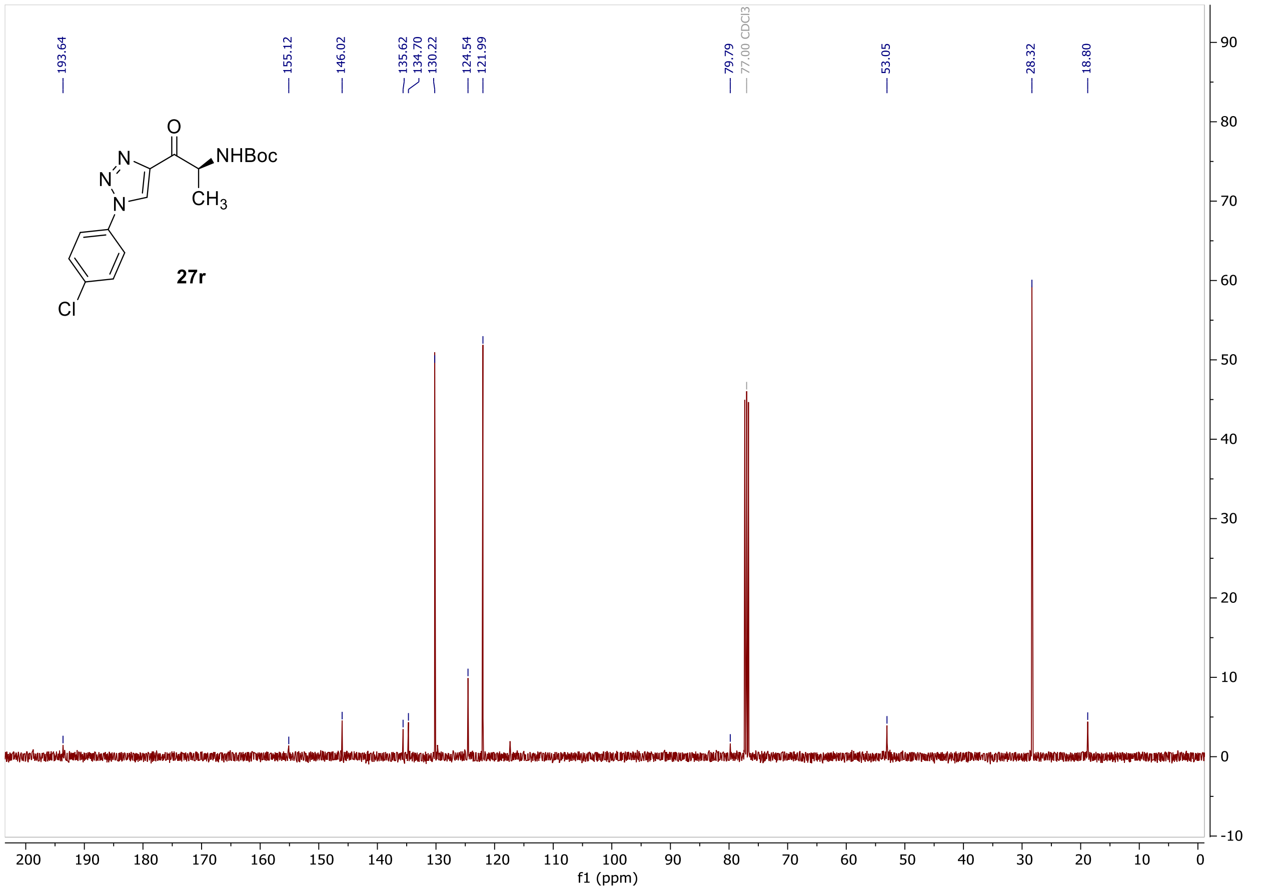

Compound **27s** (^{1}H NMR, 400 MHz):

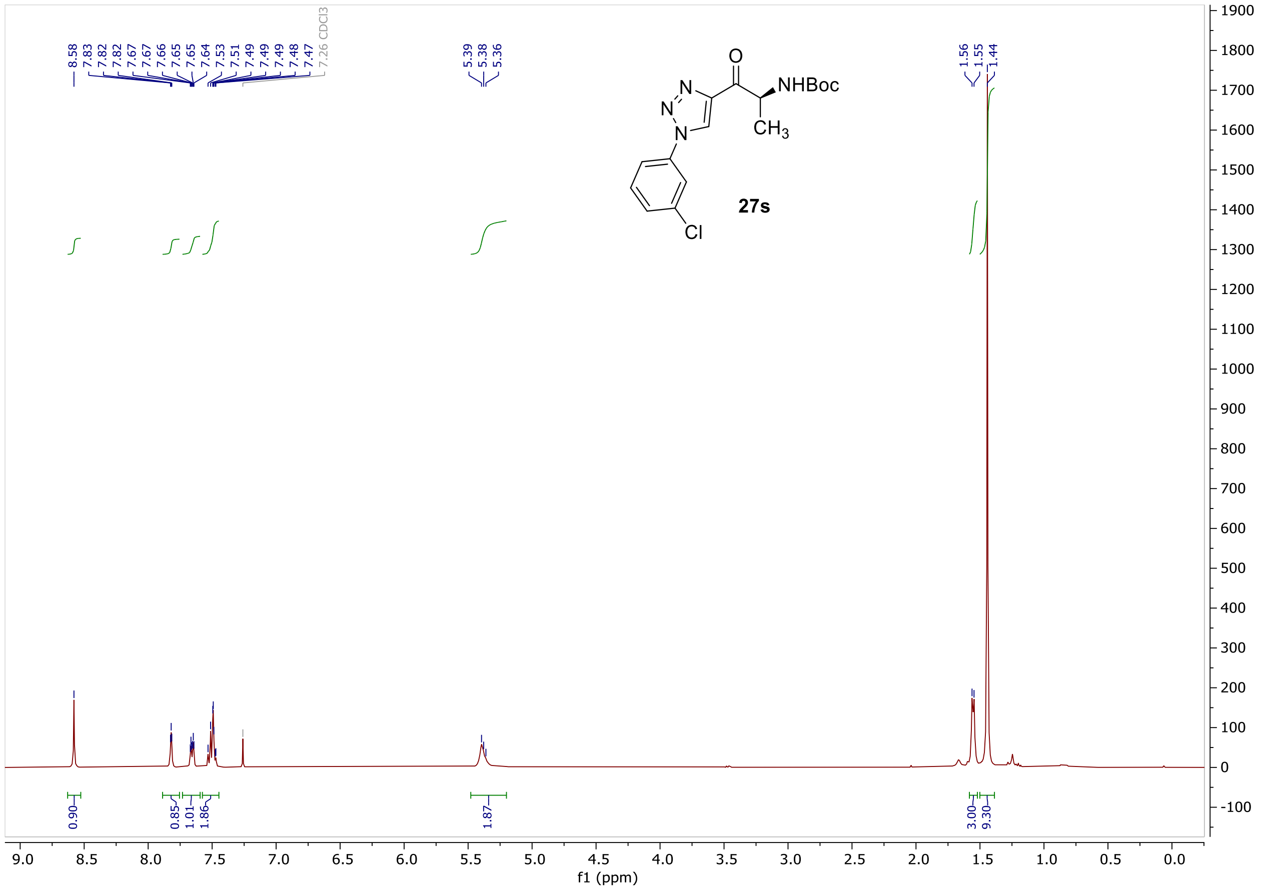

Compound **27s** (^{13}C NMR, 101 MHz):

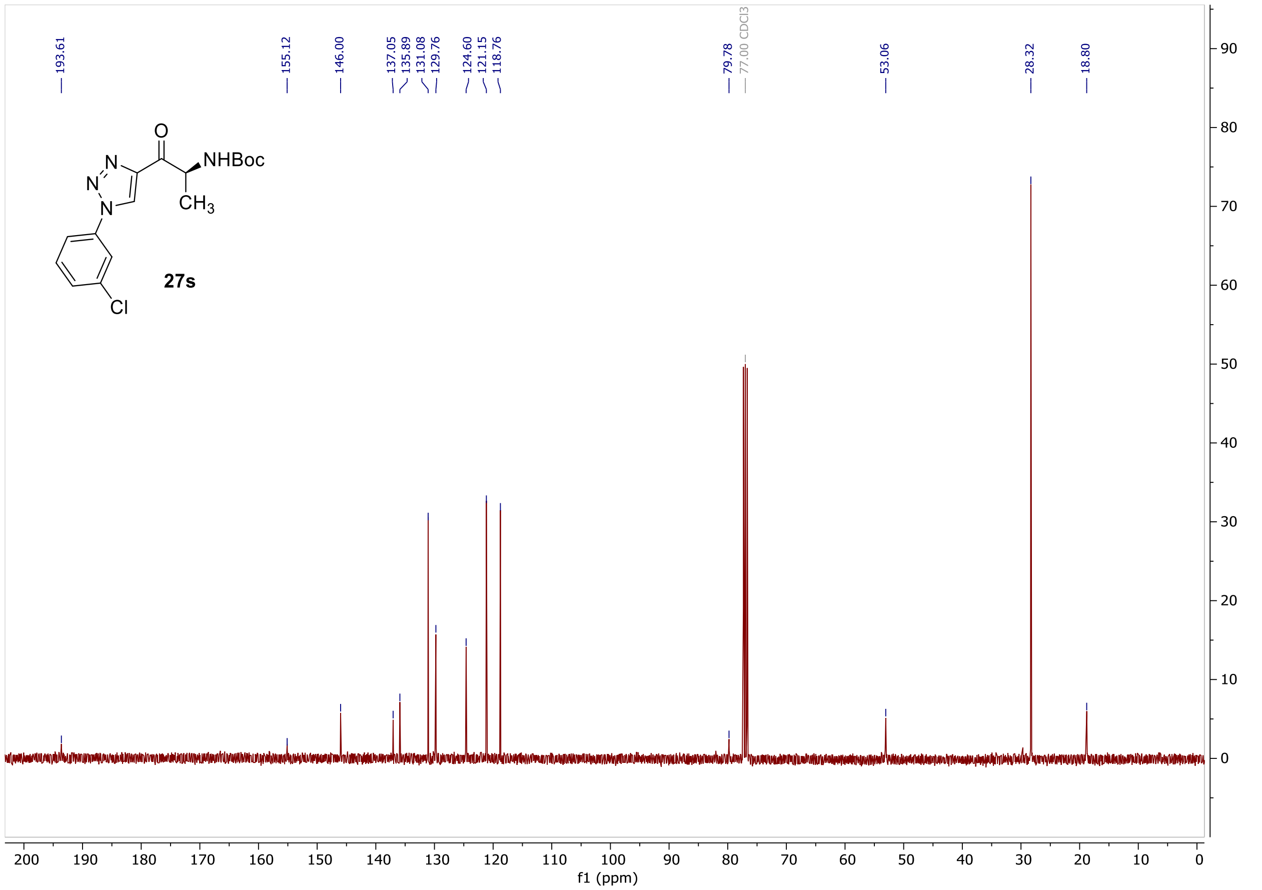

Compound **27t** (^{1}H NMR, 400 MHz):

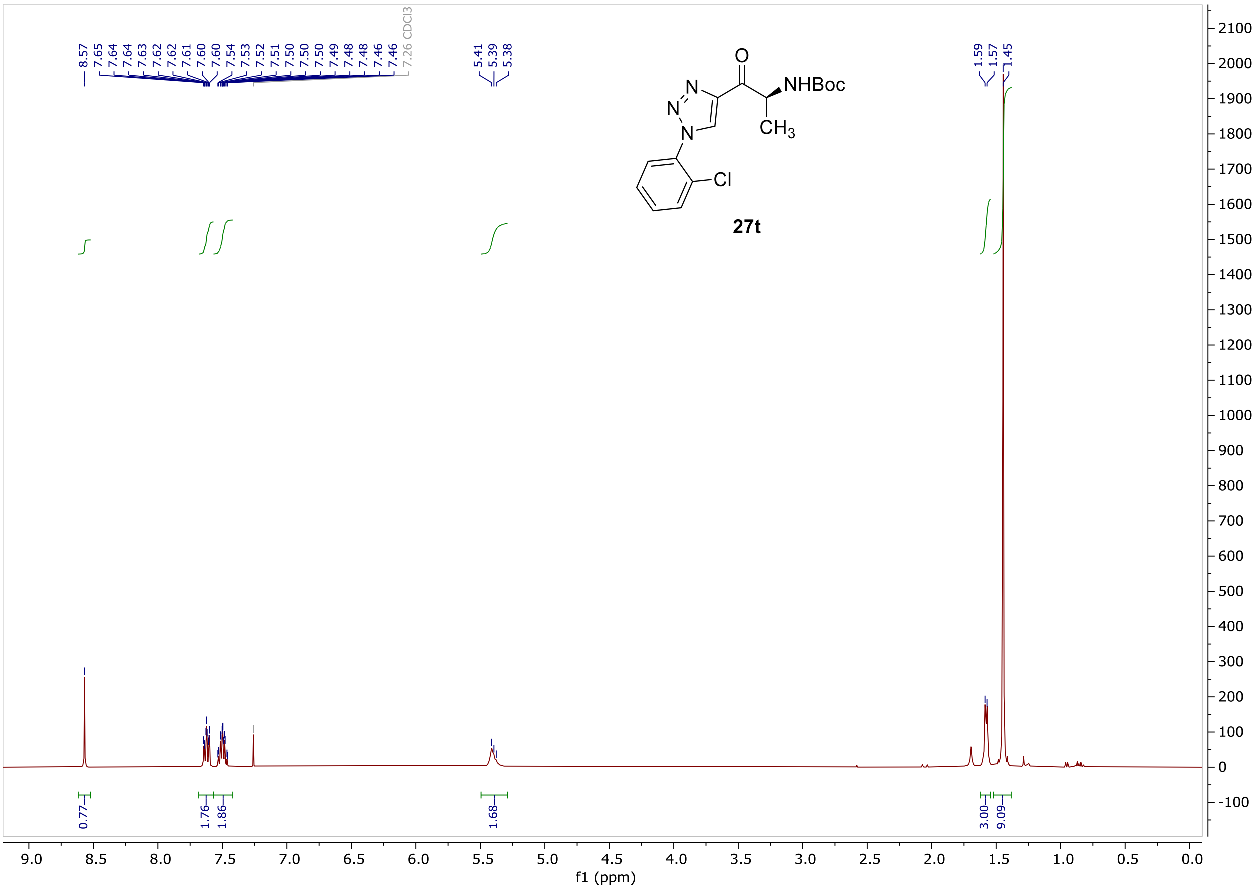

Compound **27t** (^{13}C NMR, 101 MHz):

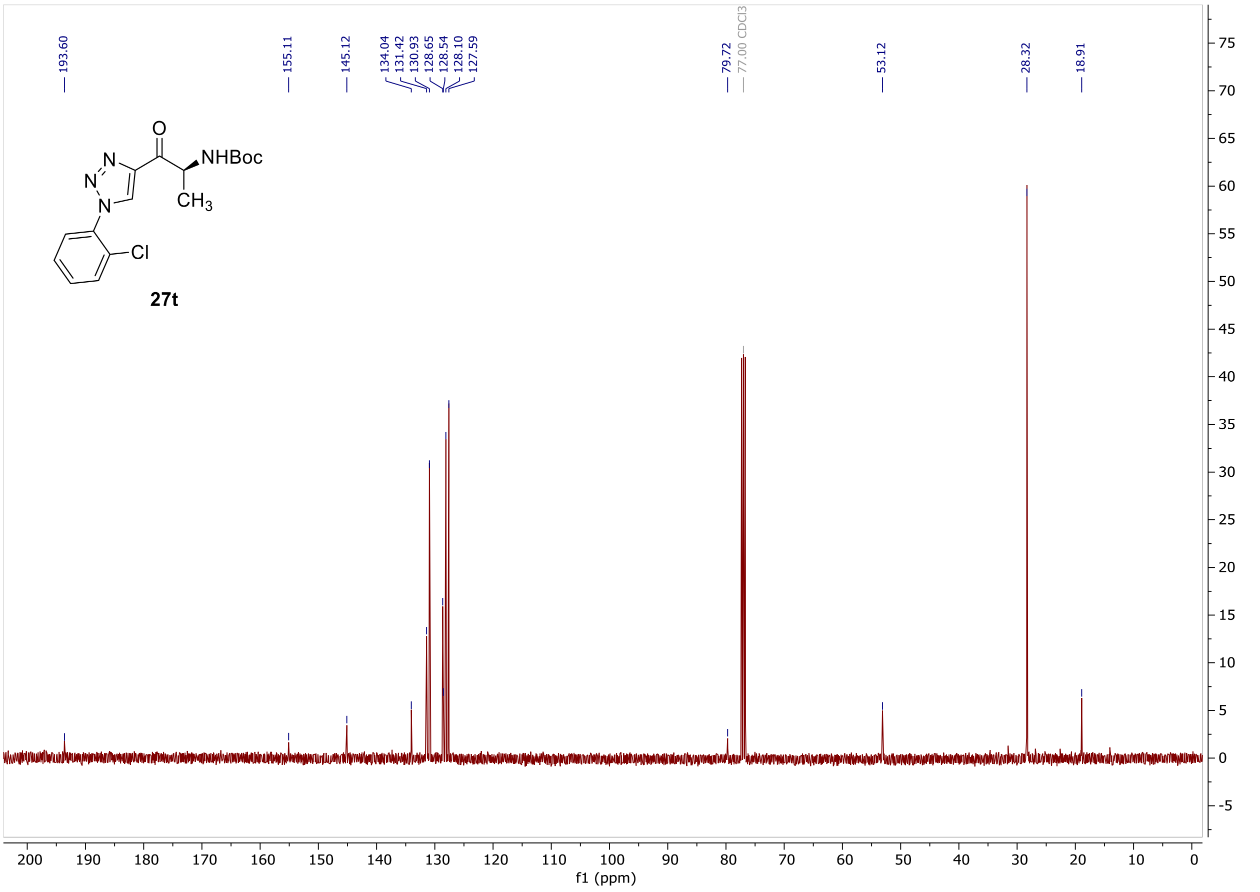

Compound **27u** (^{1}H NMR, 400 MHz):

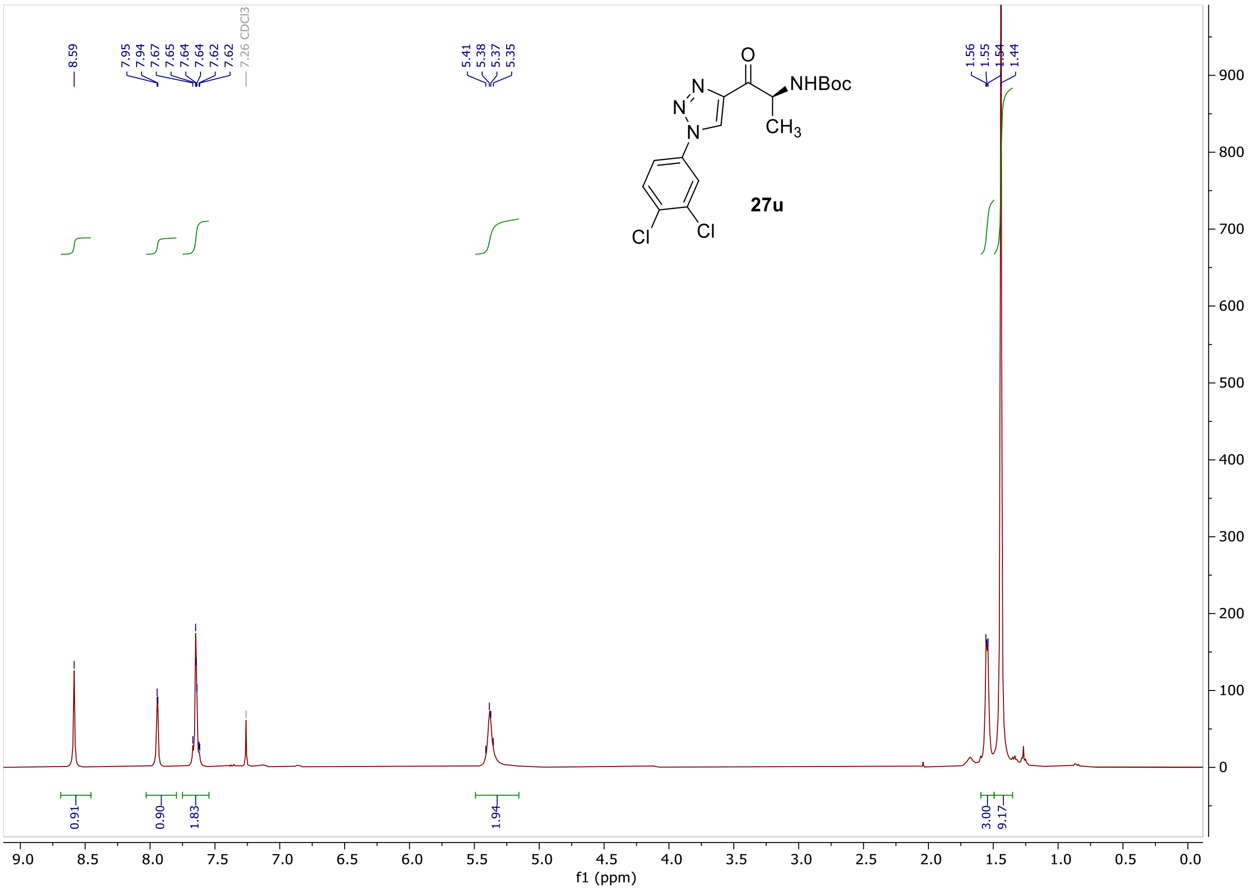

Compound **27u** (^{13}C NMR, 101 MHz):

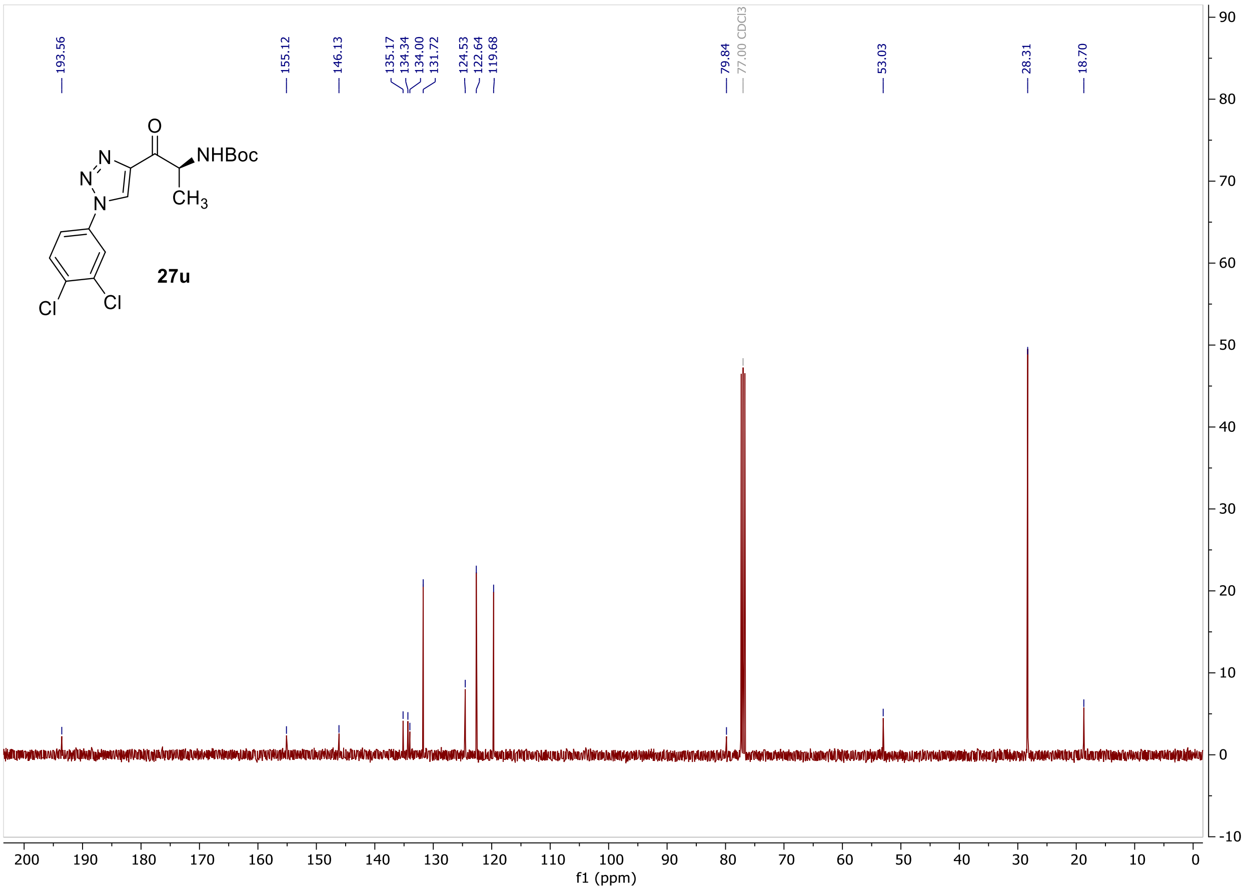

Compound **27v** (^{1}H NMR, 400 MHz):

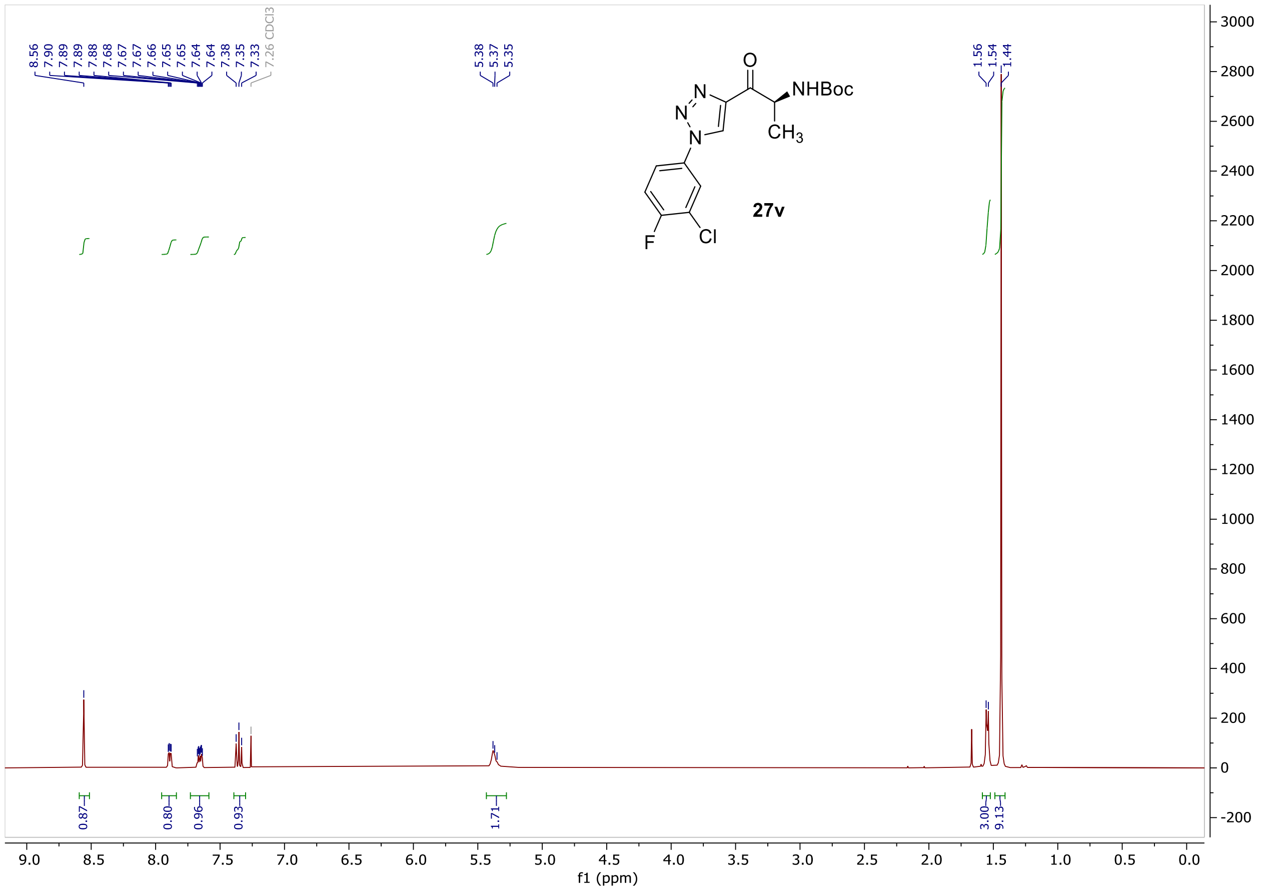

Compound **27v** (^{13}C NMR, 101 MHz):

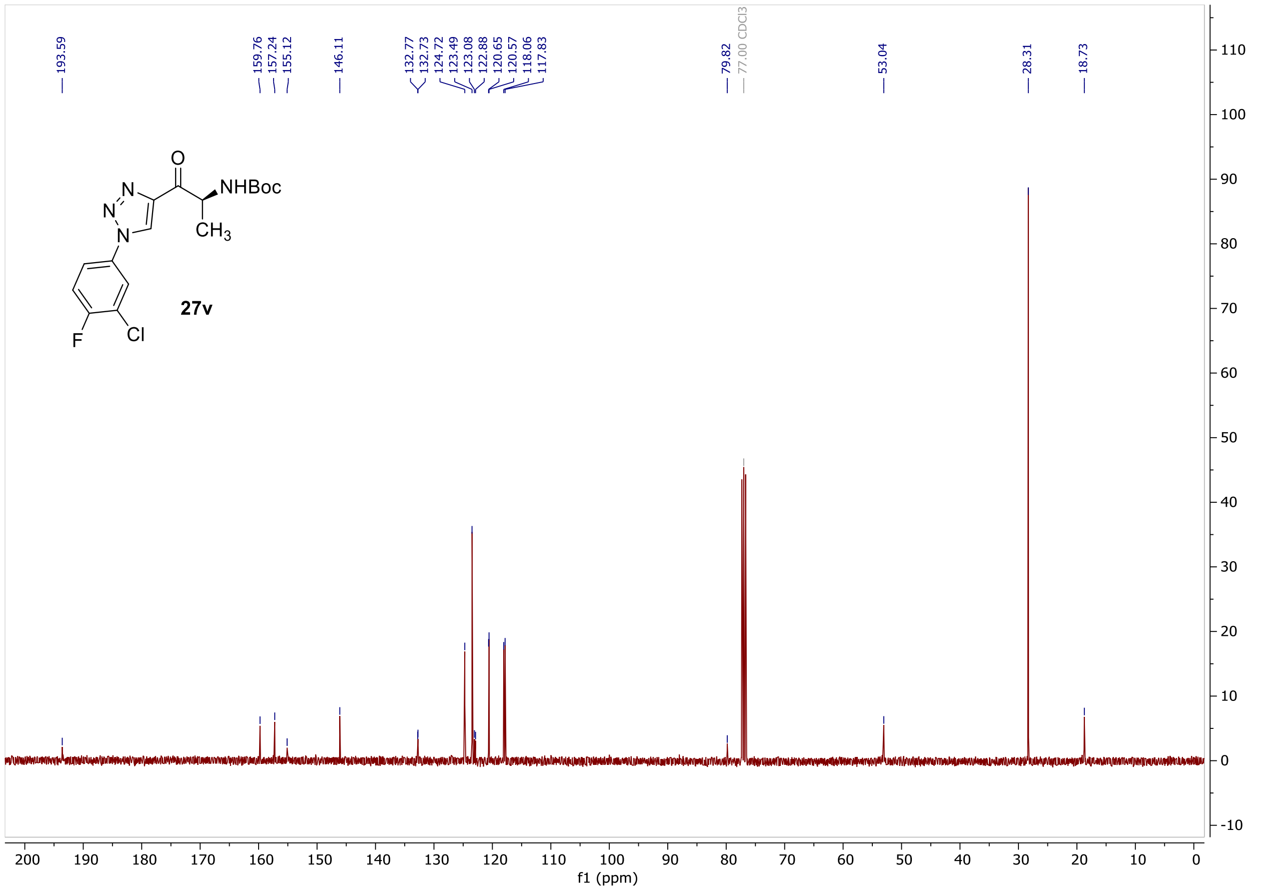

Compound **27v** (^{19}F NMR, 282 MHz):

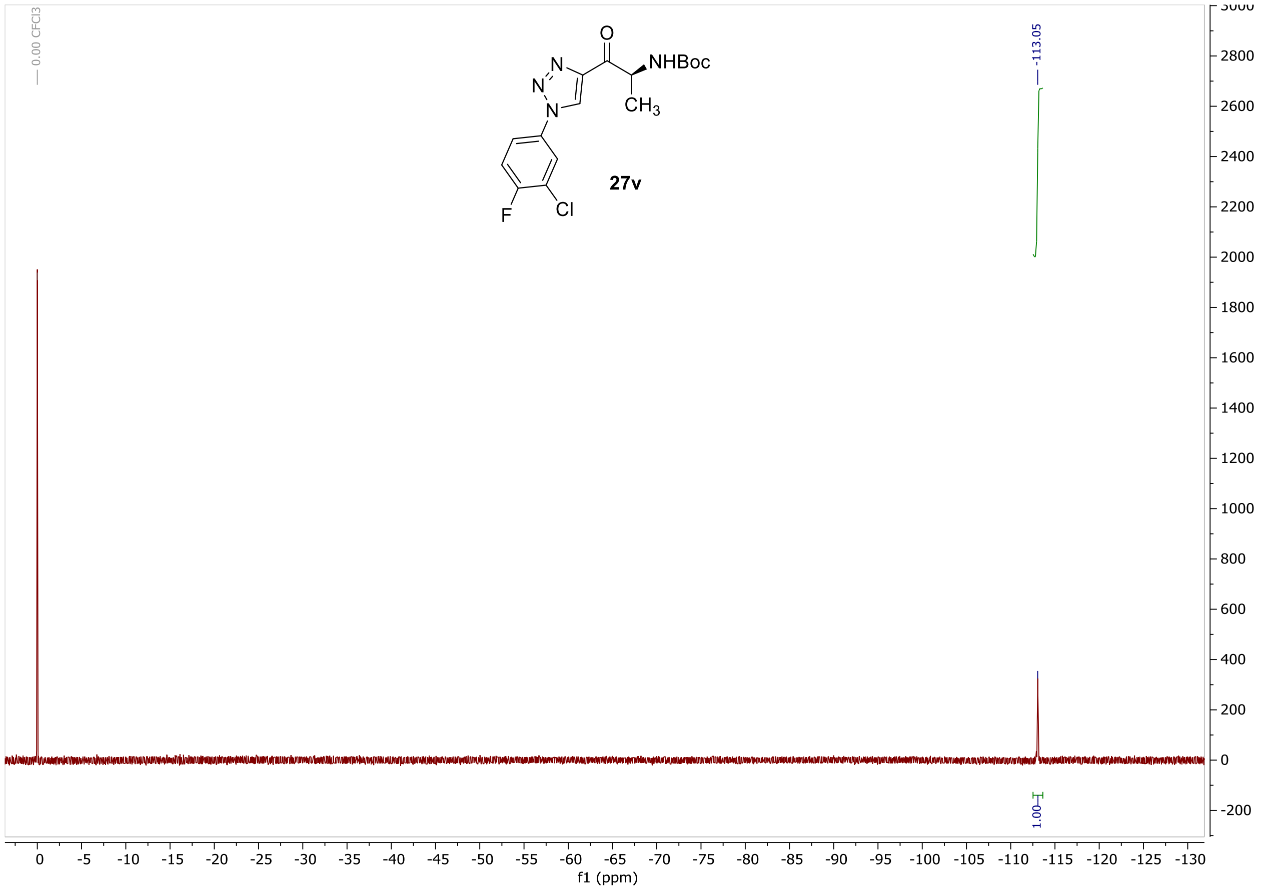

Compound **35a** (^{1}H NMR, 400 MHz):

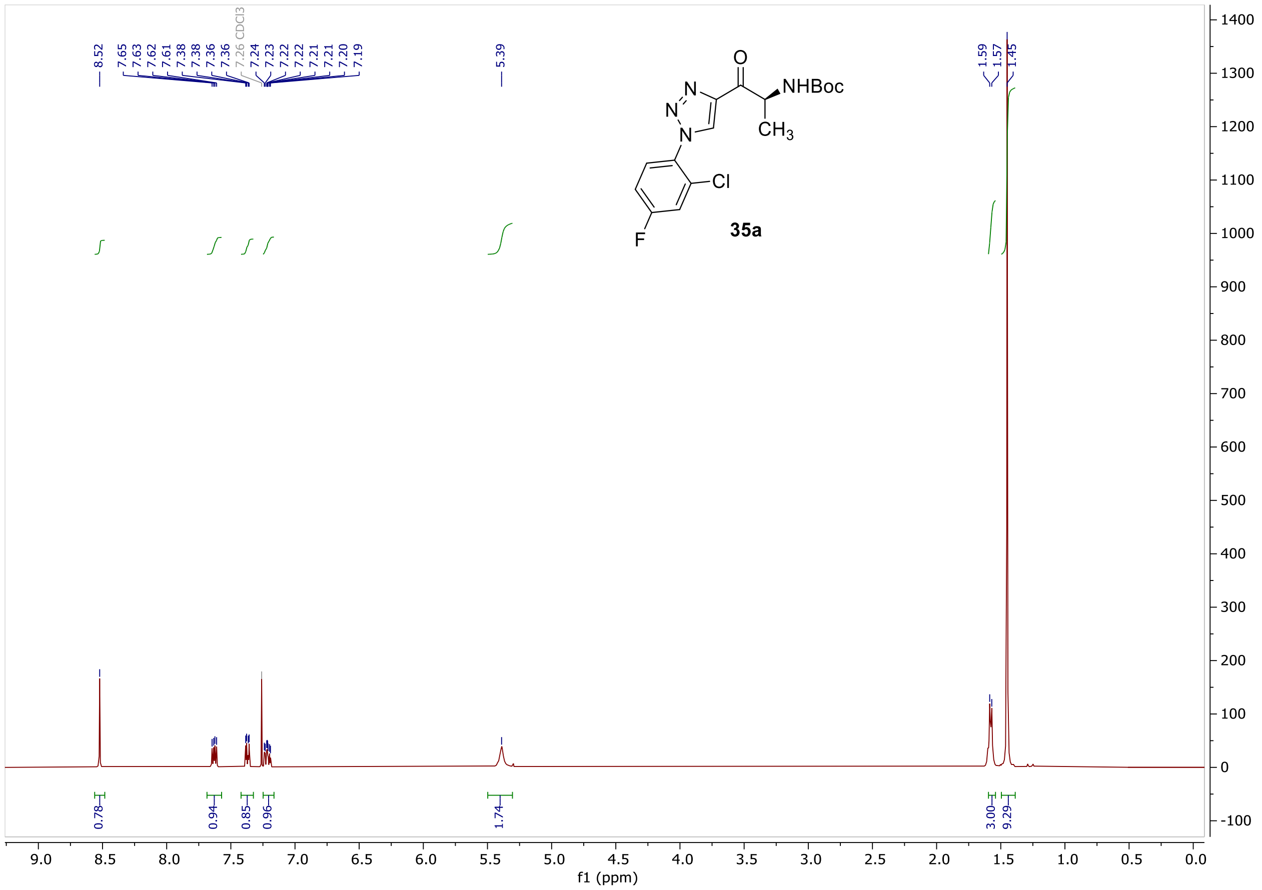

Compound **35a** (^{13}C NMR, 101 MHz):

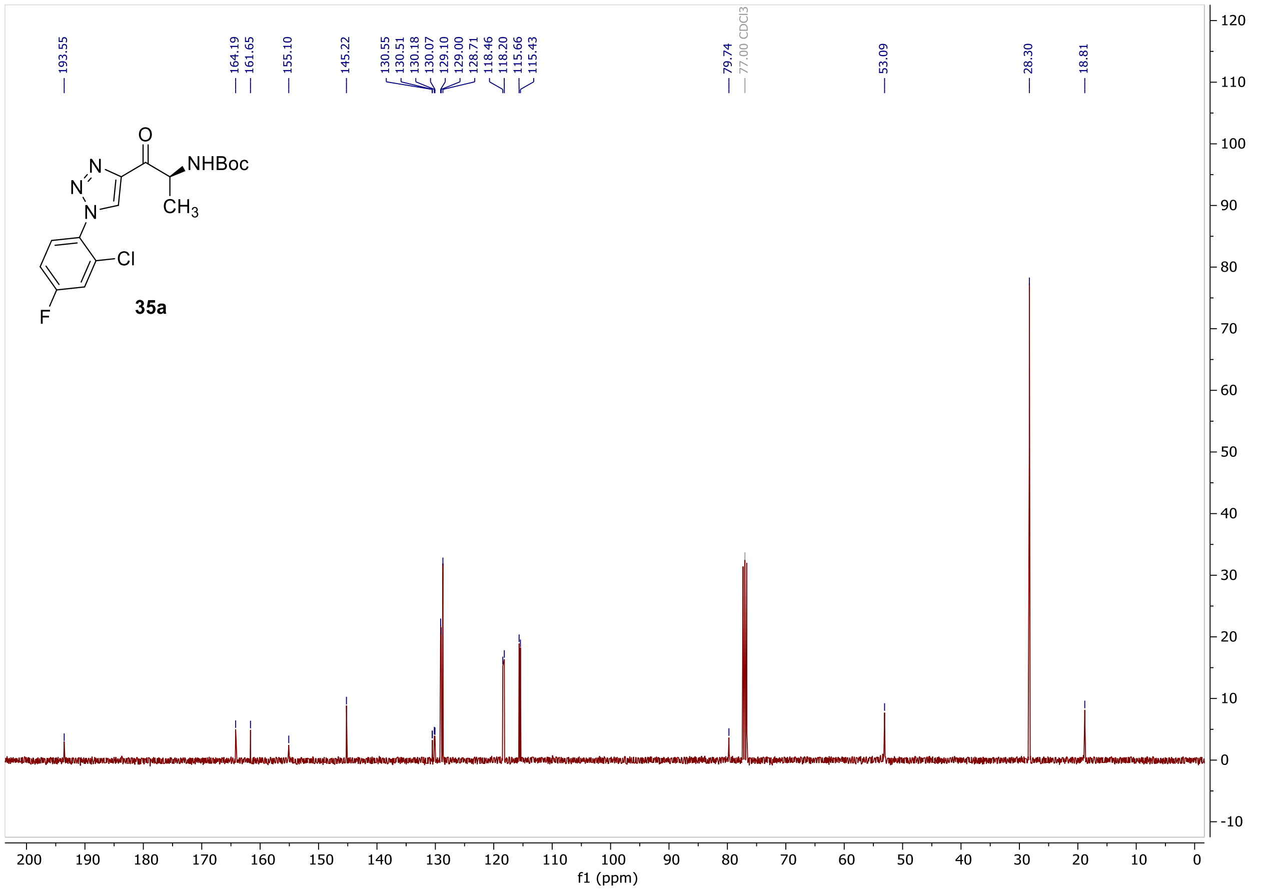

Compound **35a** (^{19}F NMR, 282 MHz):

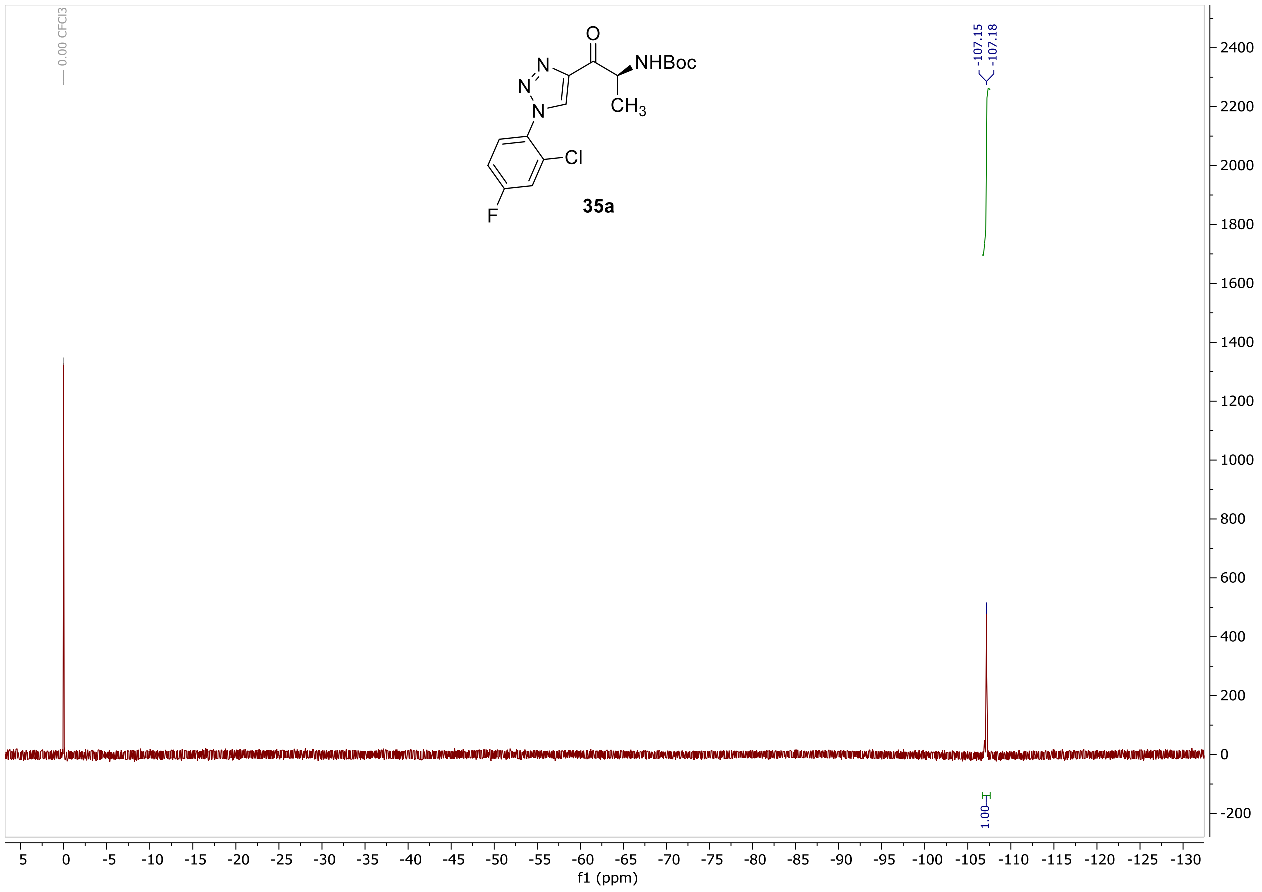

Compound **35b** (^{1}H NMR, 400 MHz):

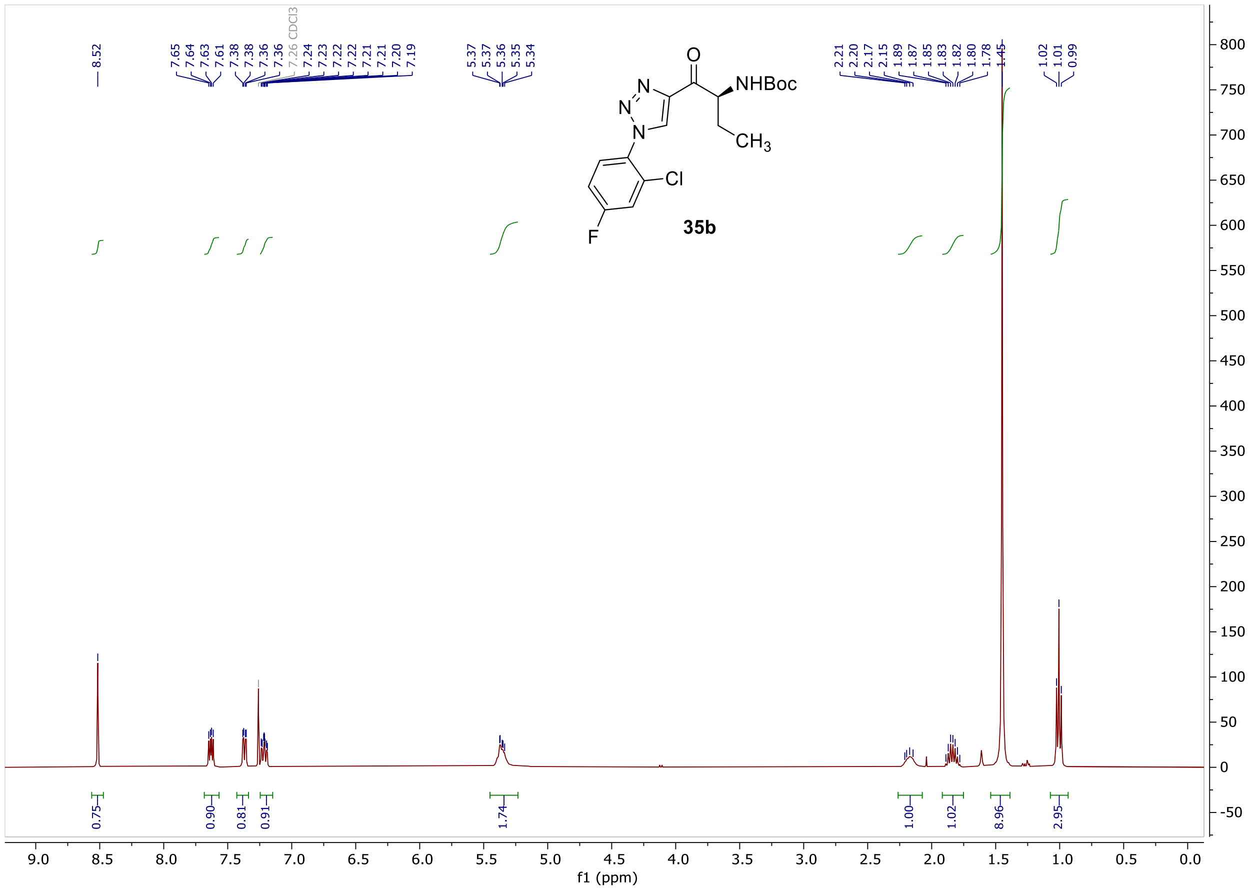

Compound **35b** (^{13}C NMR, 101 MHz):

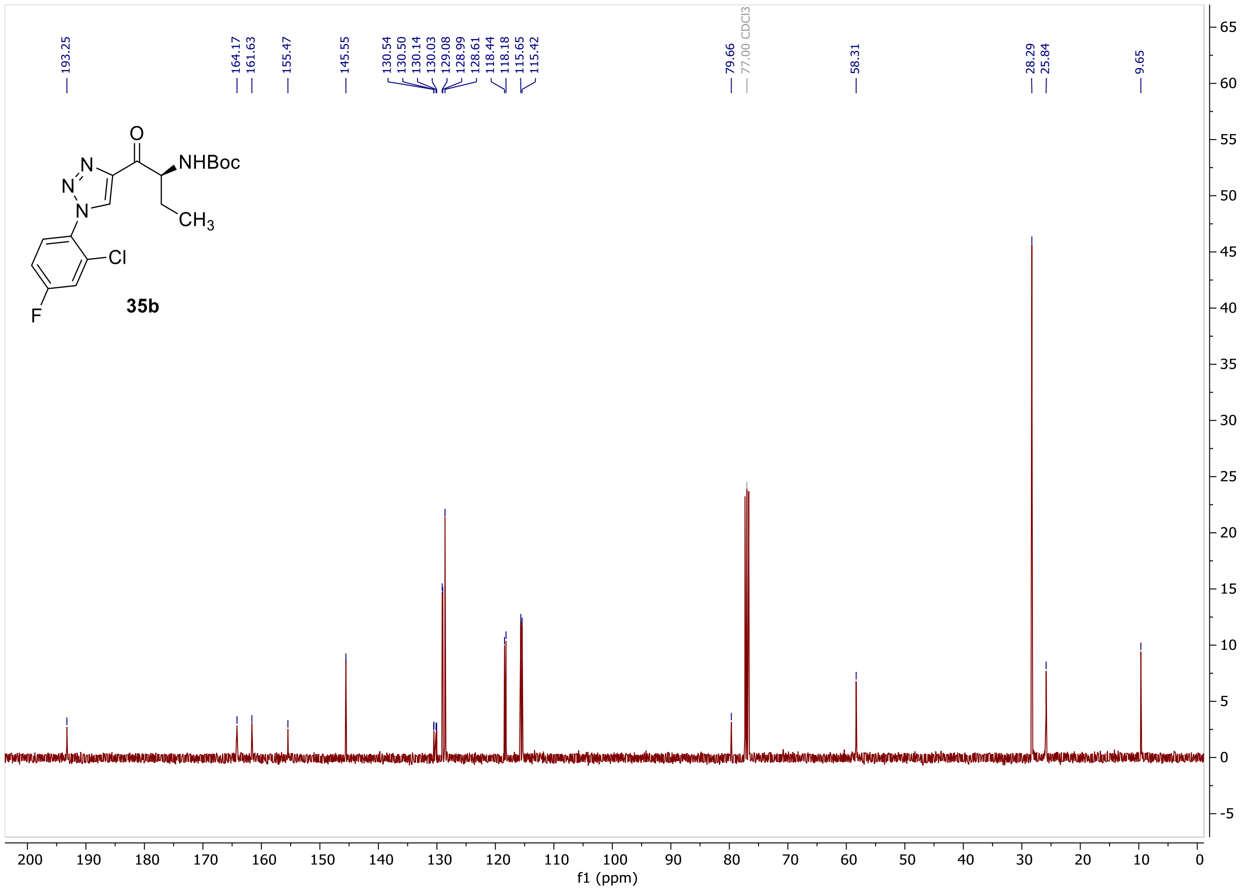

Compound **35b** (^{19}F NMR, 282 MHz):

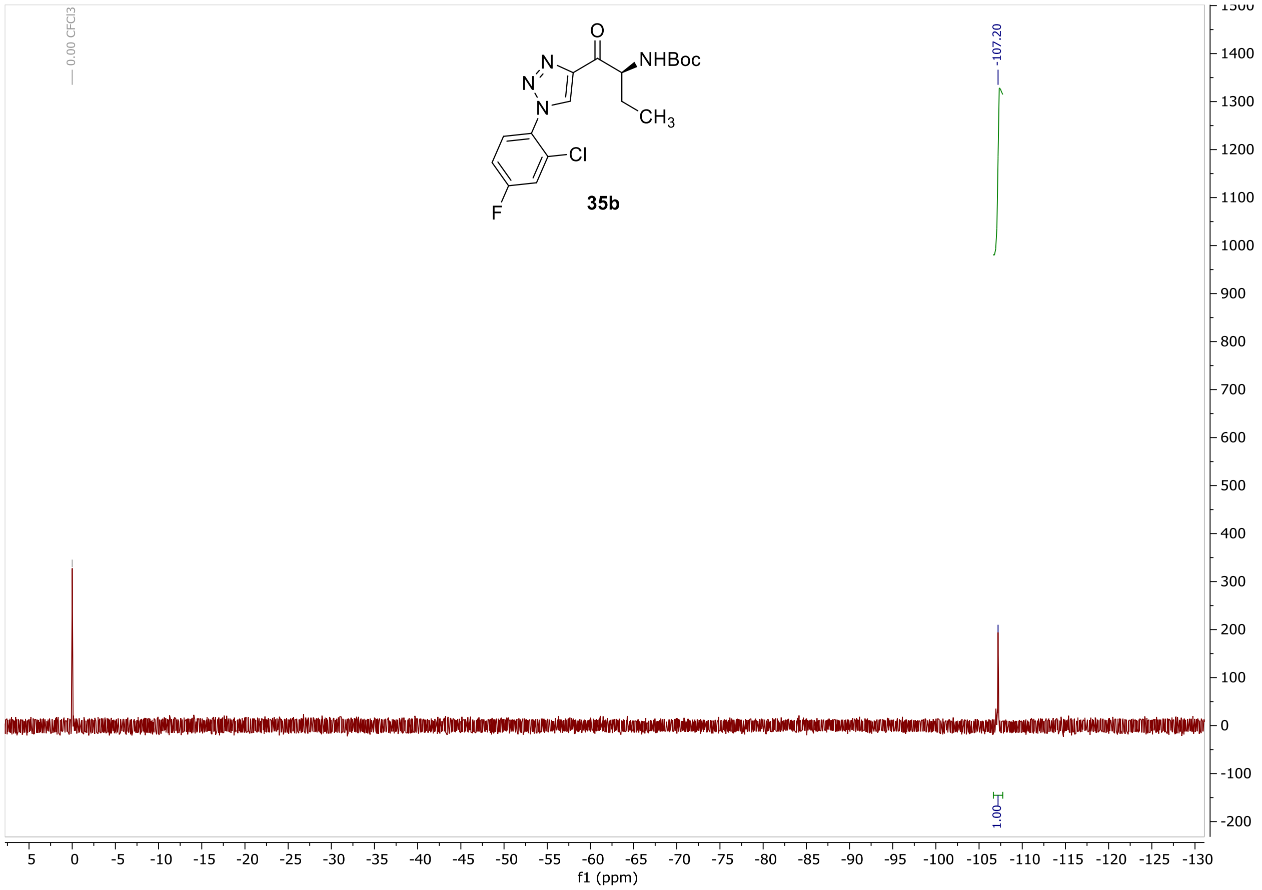

Compound **35c** (^{1}H NMR, 400 MHz):

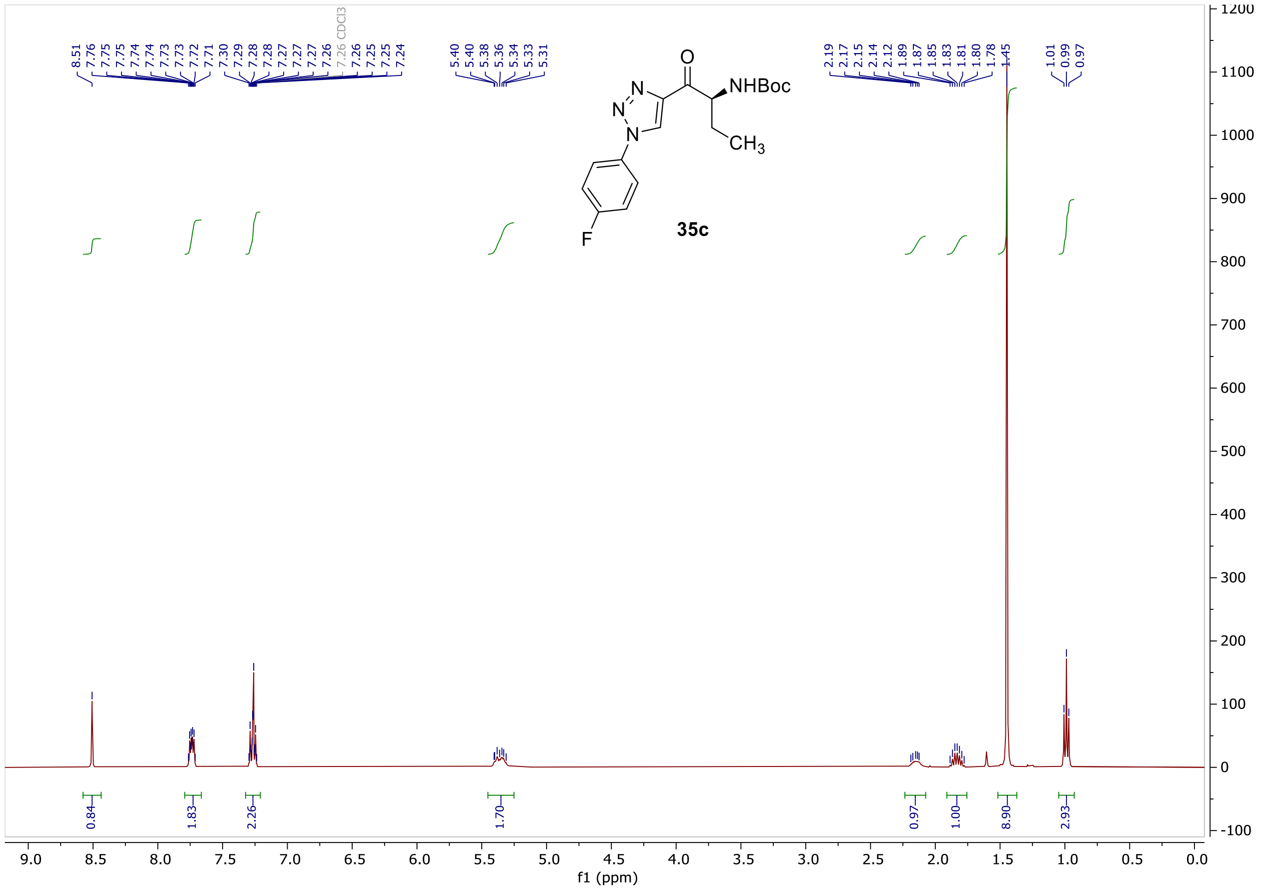

Compound **35c** (^{13}C NMR, 101 MHz):

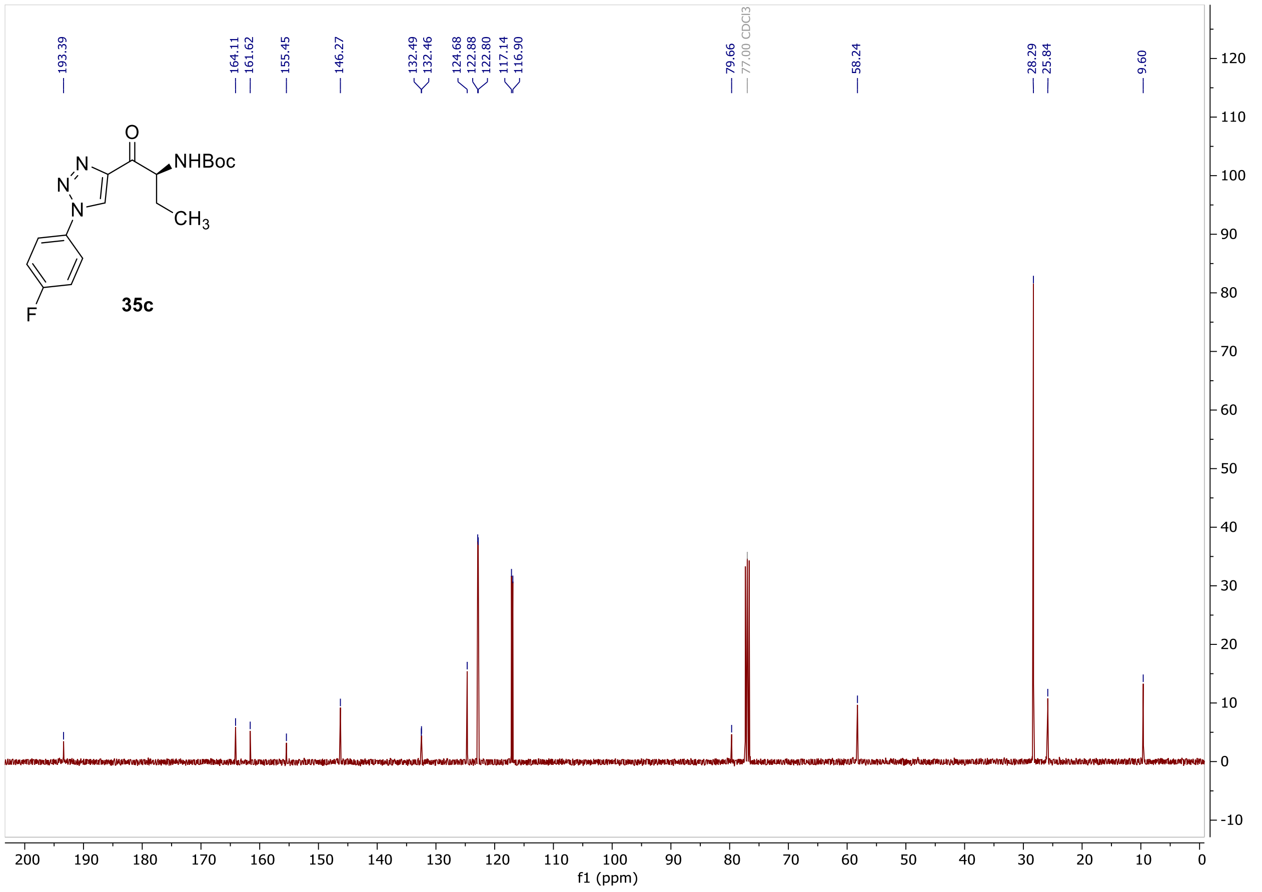

Compound **35c** (^{19}F NMR, 282 MHz):

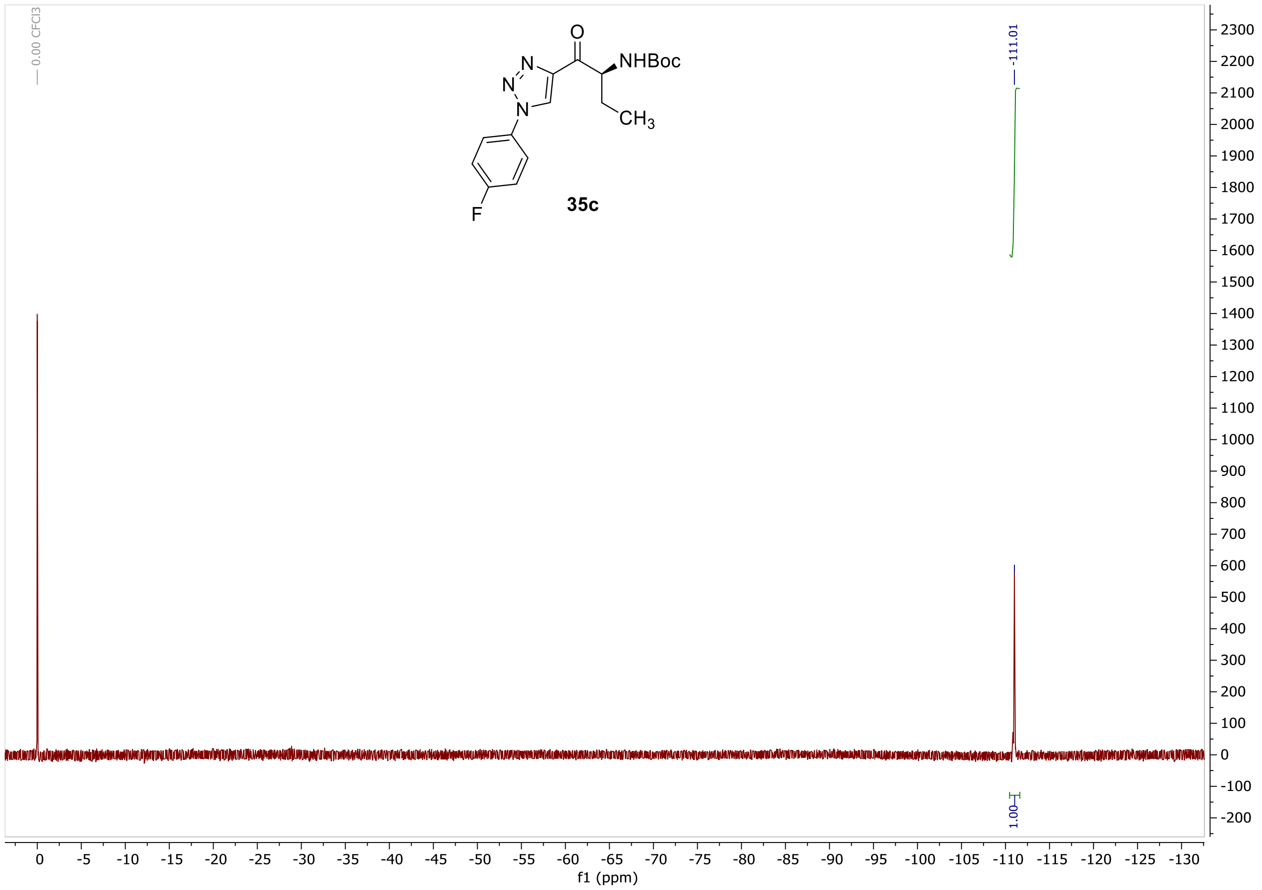

Compound **12** (^{1}H NMR, 400 MHz):

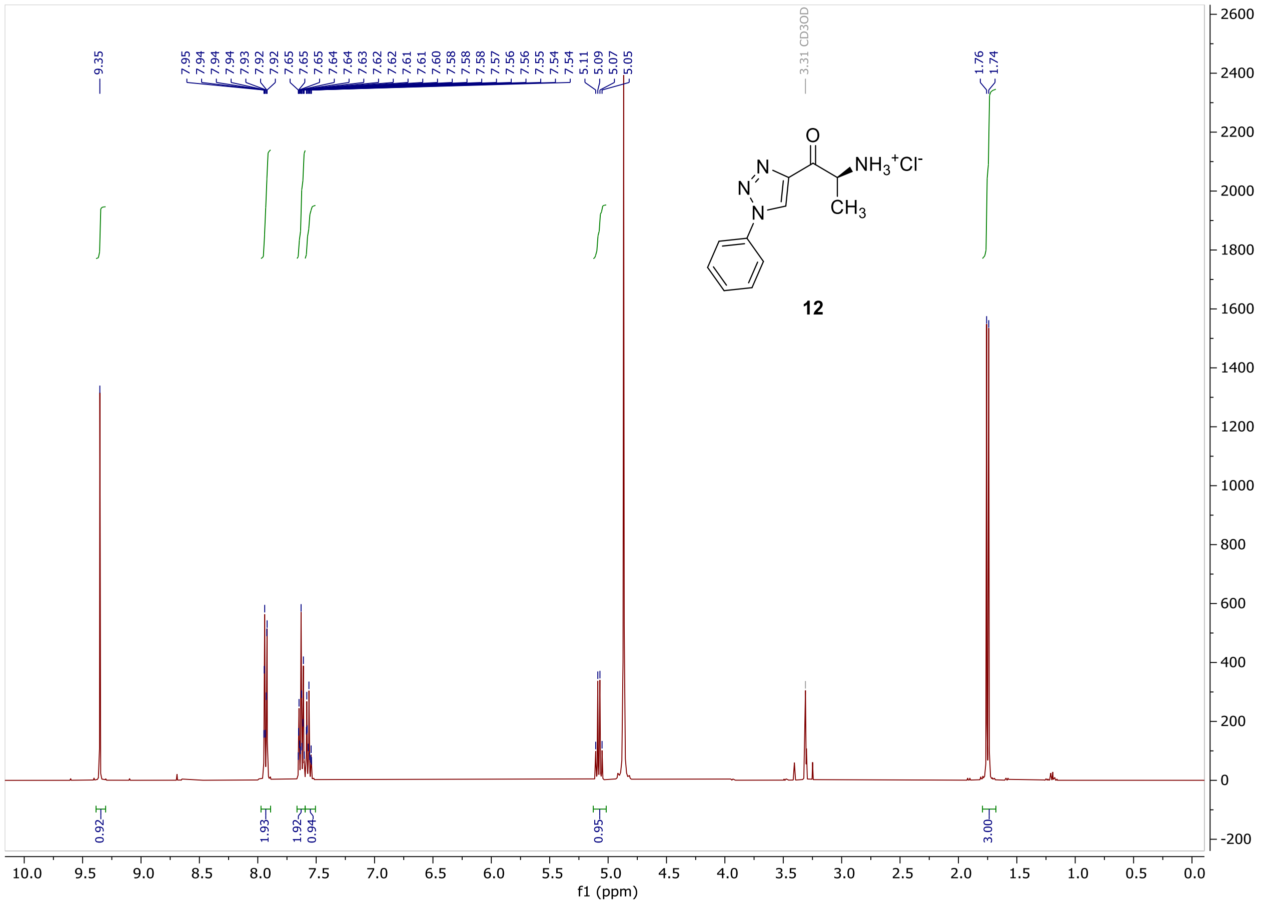

Compound **12** (^{13}C NMR, 101 MHz):

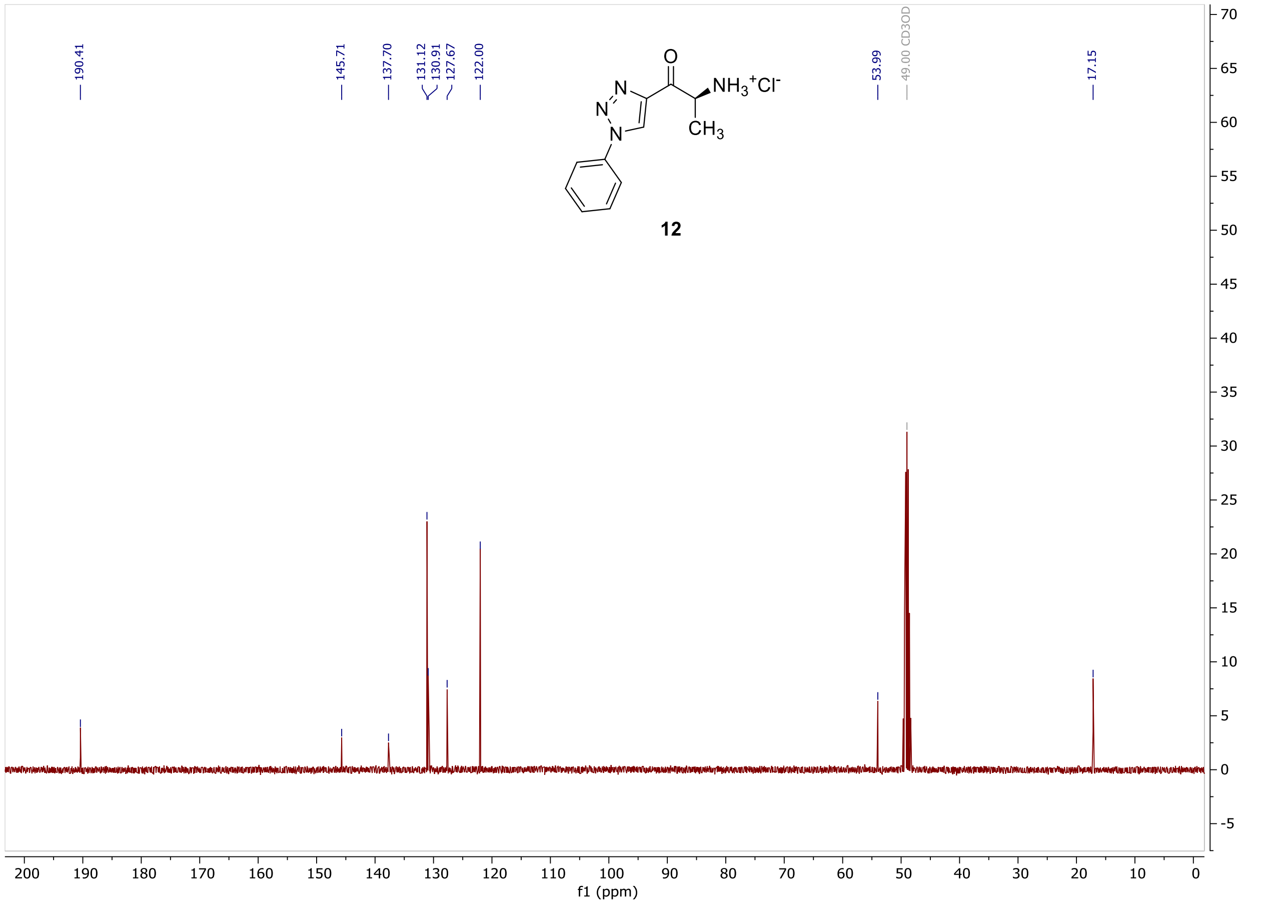

Compound **21a** (^{1}H NMR, 400 MHz):

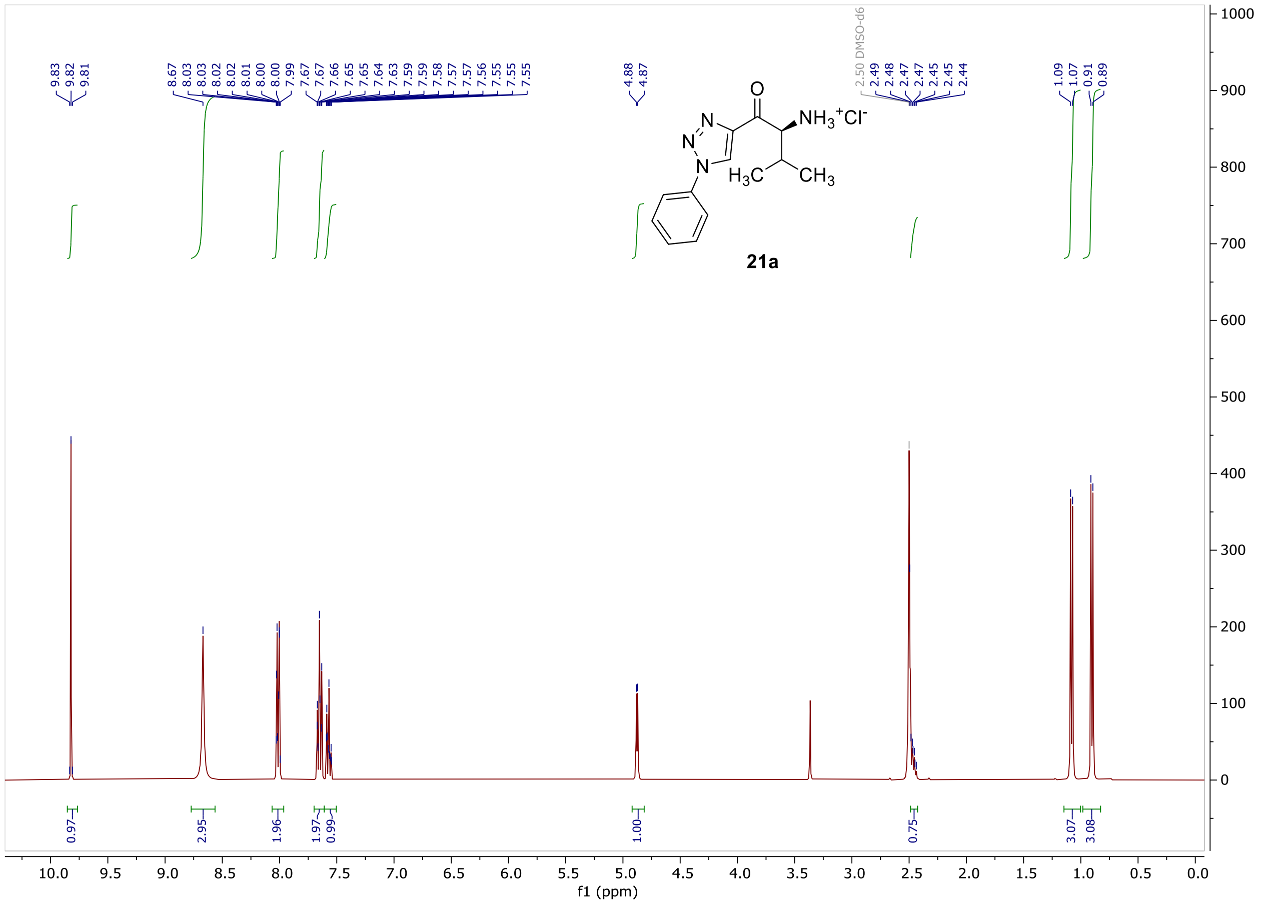

Compound **21a** (^{13}C NMR, 101 MHz):

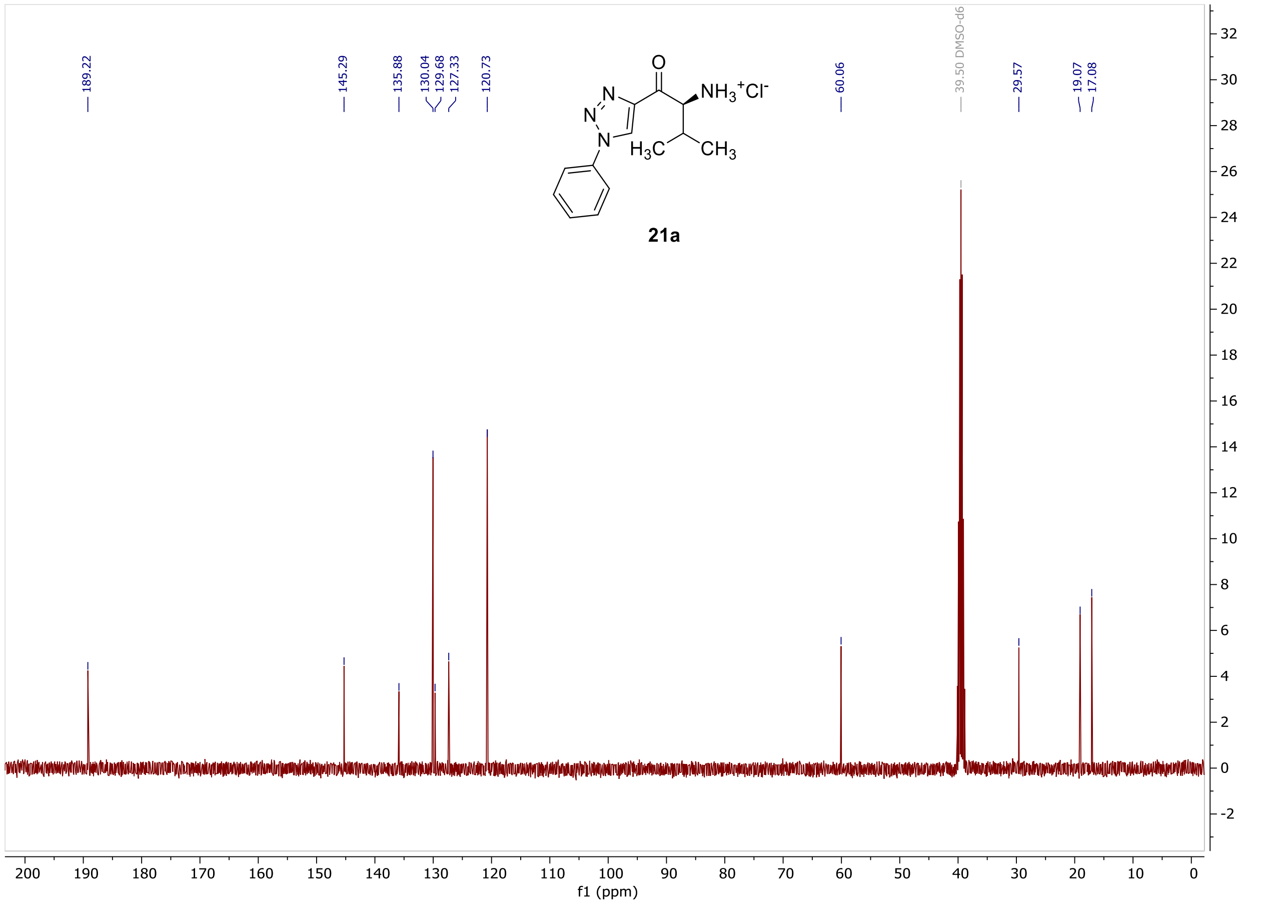

Compound **21b** (^{1}H NMR, 400 MHz):

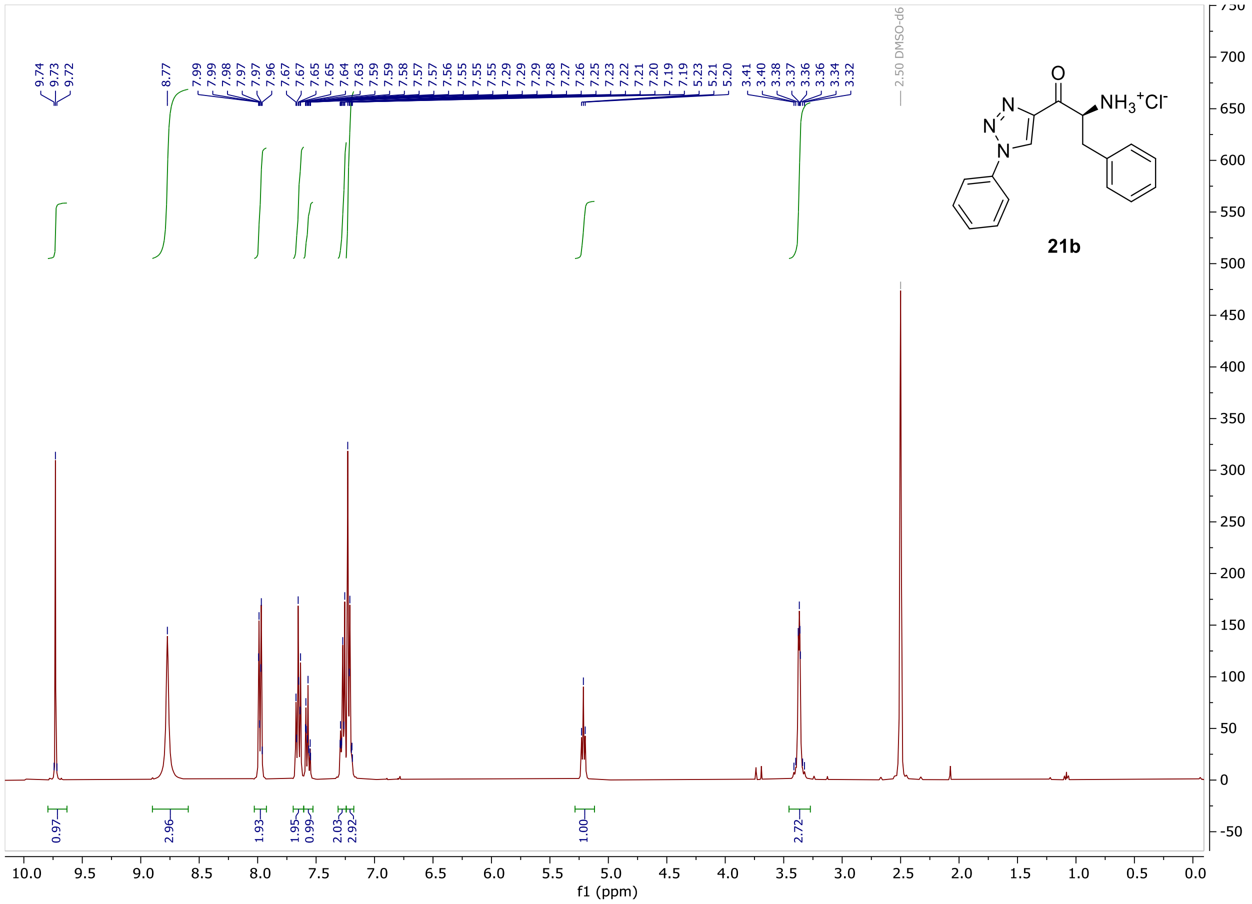

Compound **21b** (^{13}C NMR, 101 MHz):

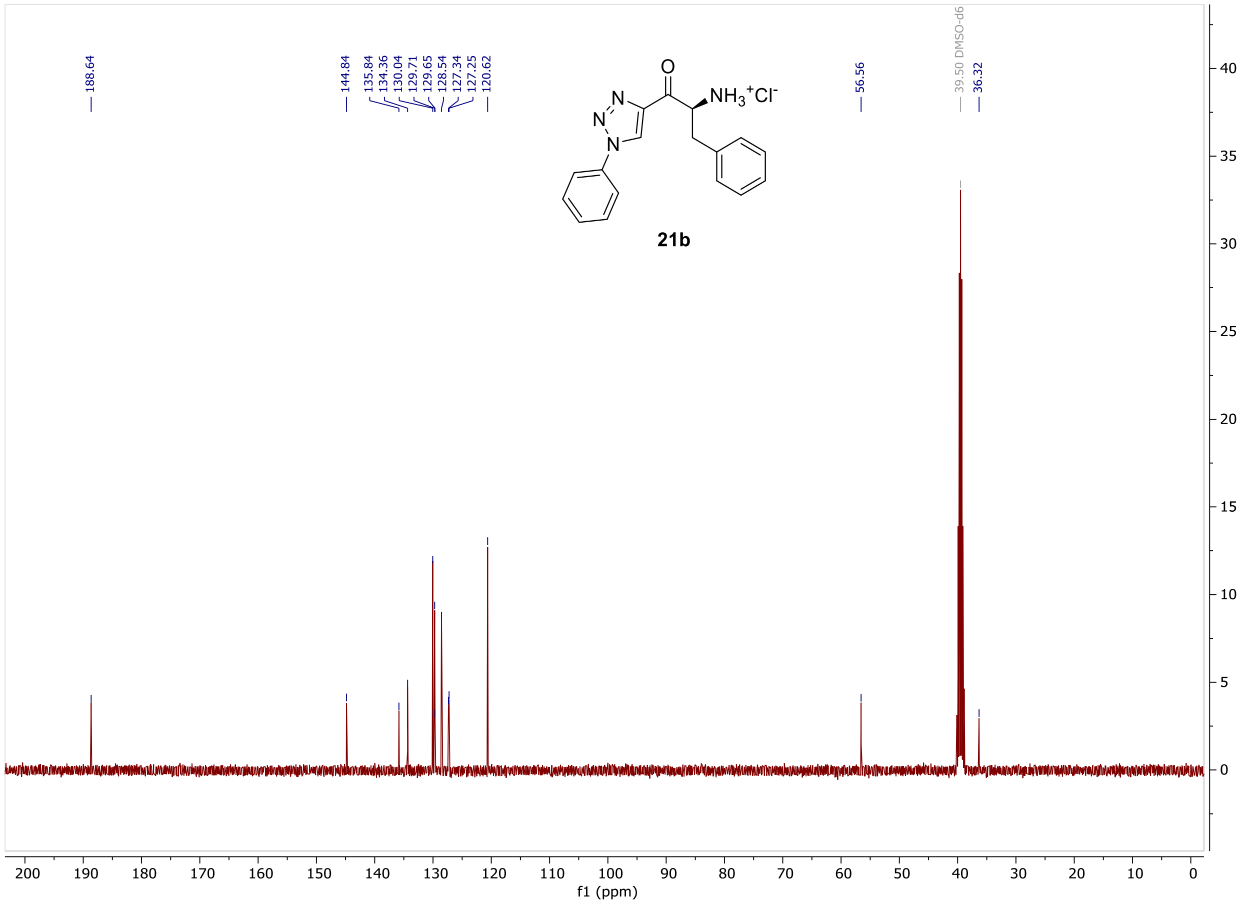

Compound **21c** (^{1}H NMR, 400 MHz):

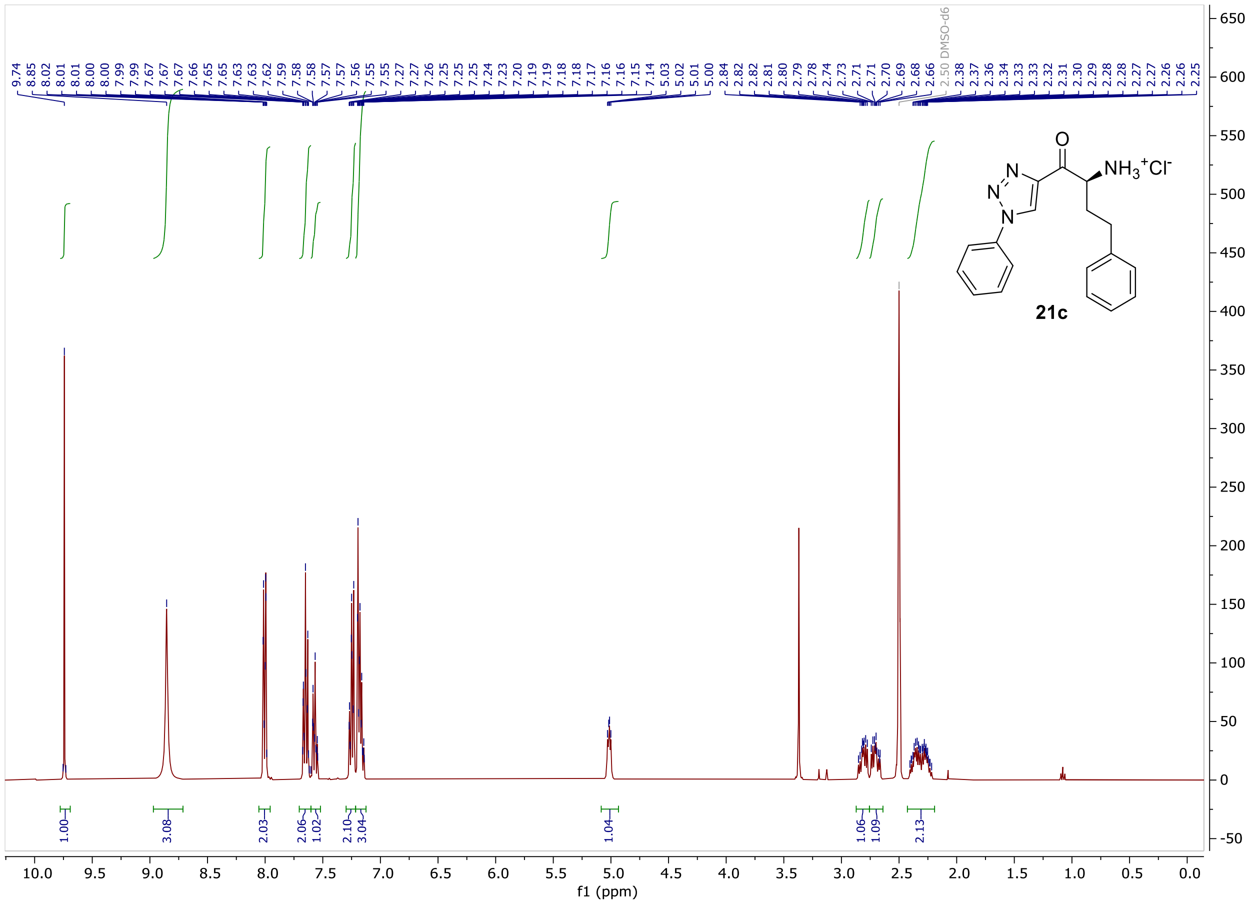

Compound **21c** (^{13}C NMR, 101 MHz):

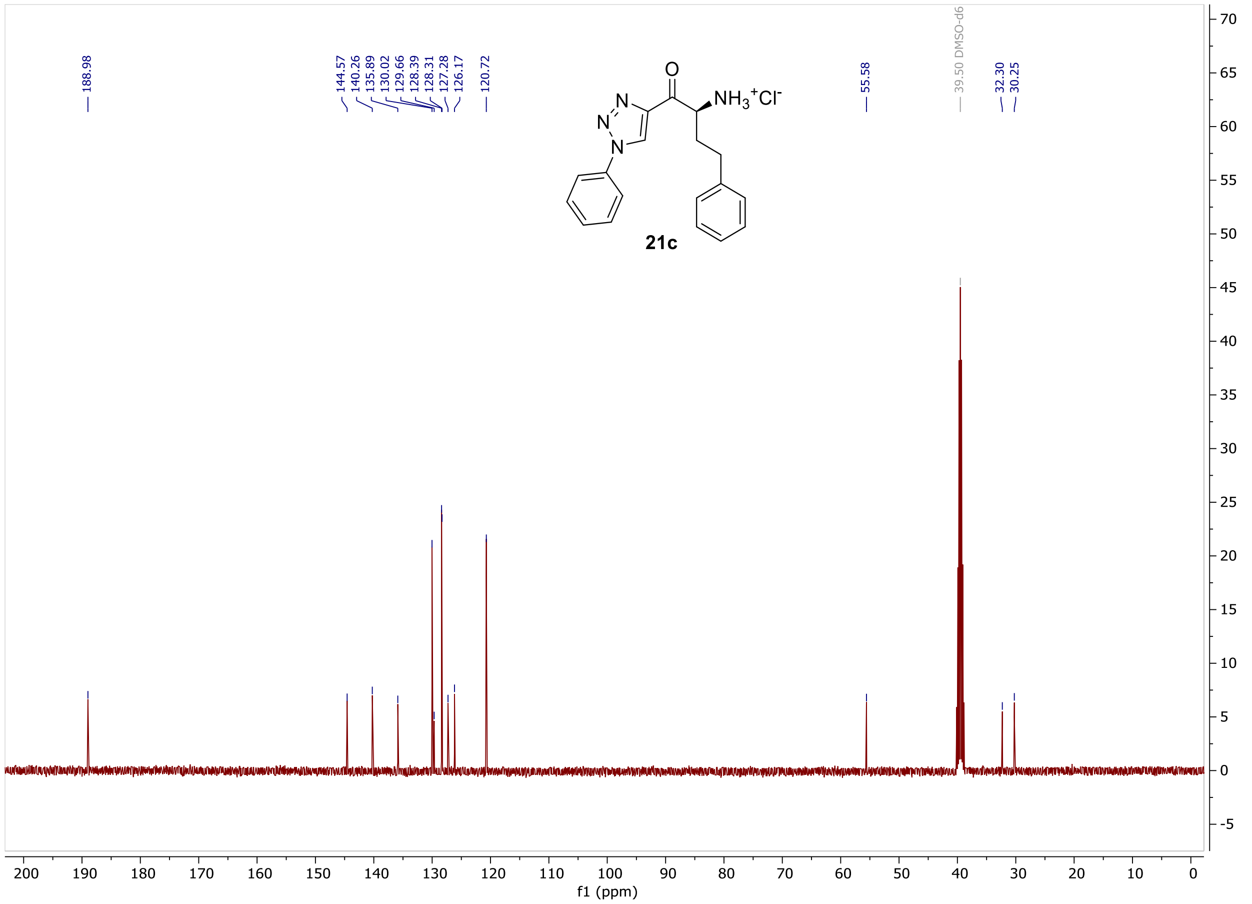

Compound **21d** (^{1}H NMR, 400 MHz):

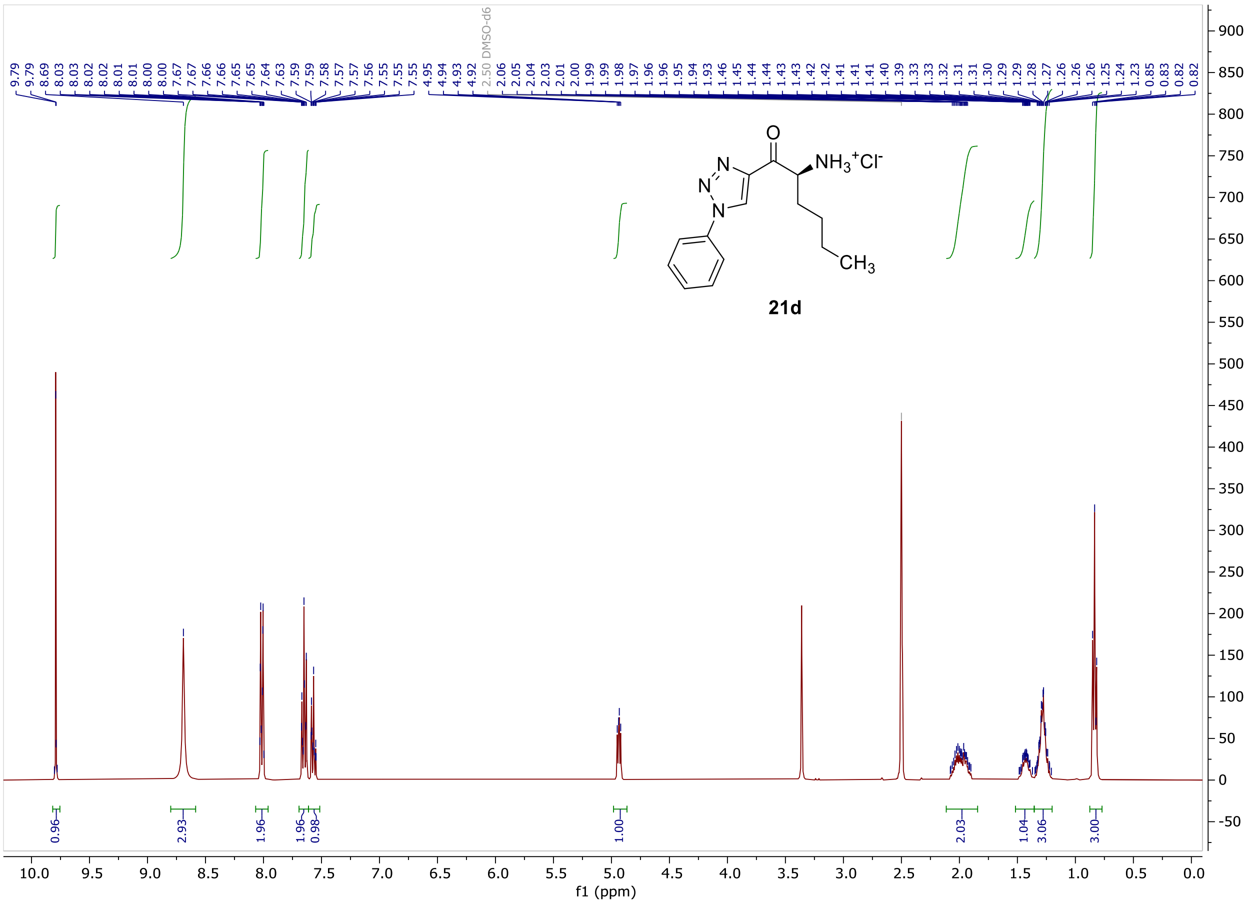

Compound **21d** (^{13}C NMR, 101 MHz):

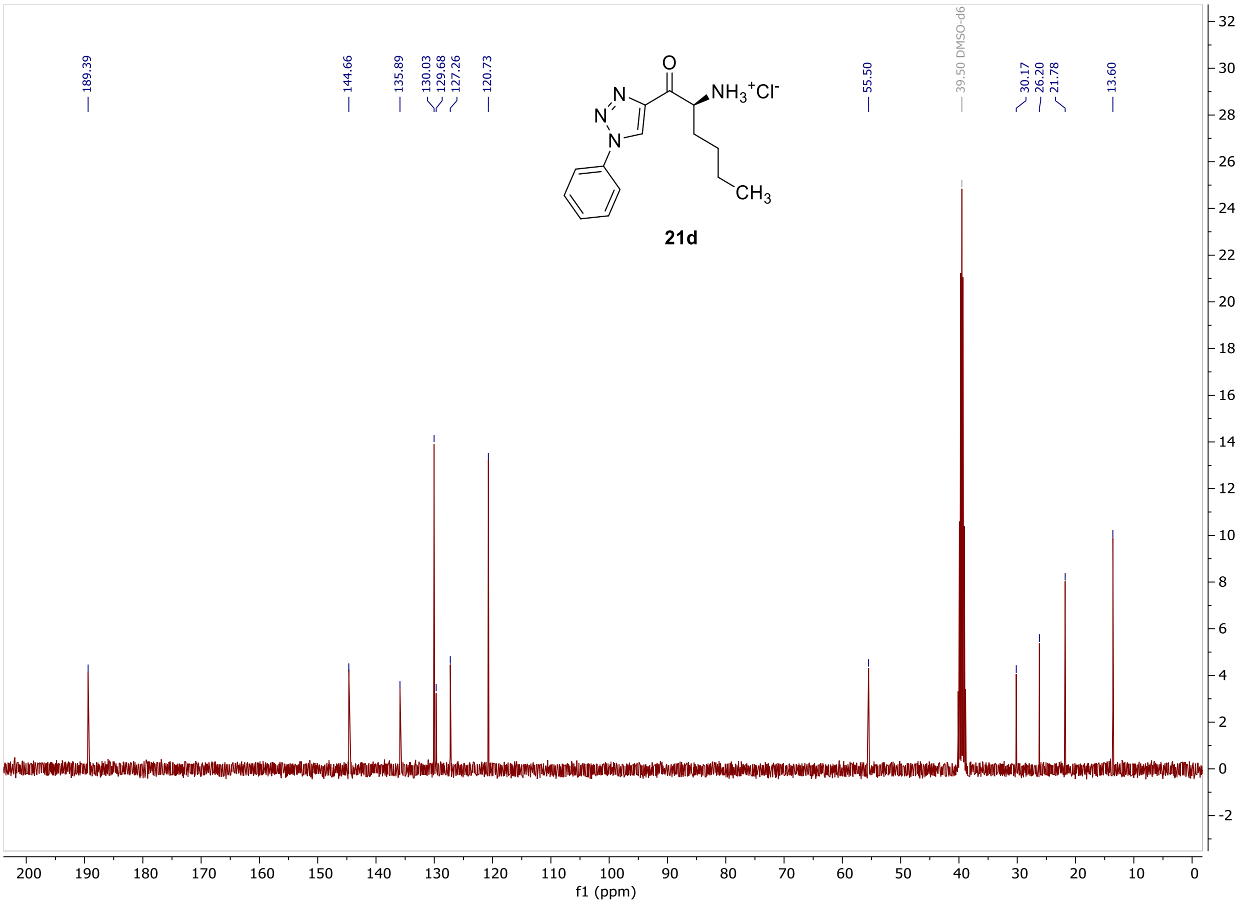

Compound **21e** (^{1}H NMR, 400 MHz):

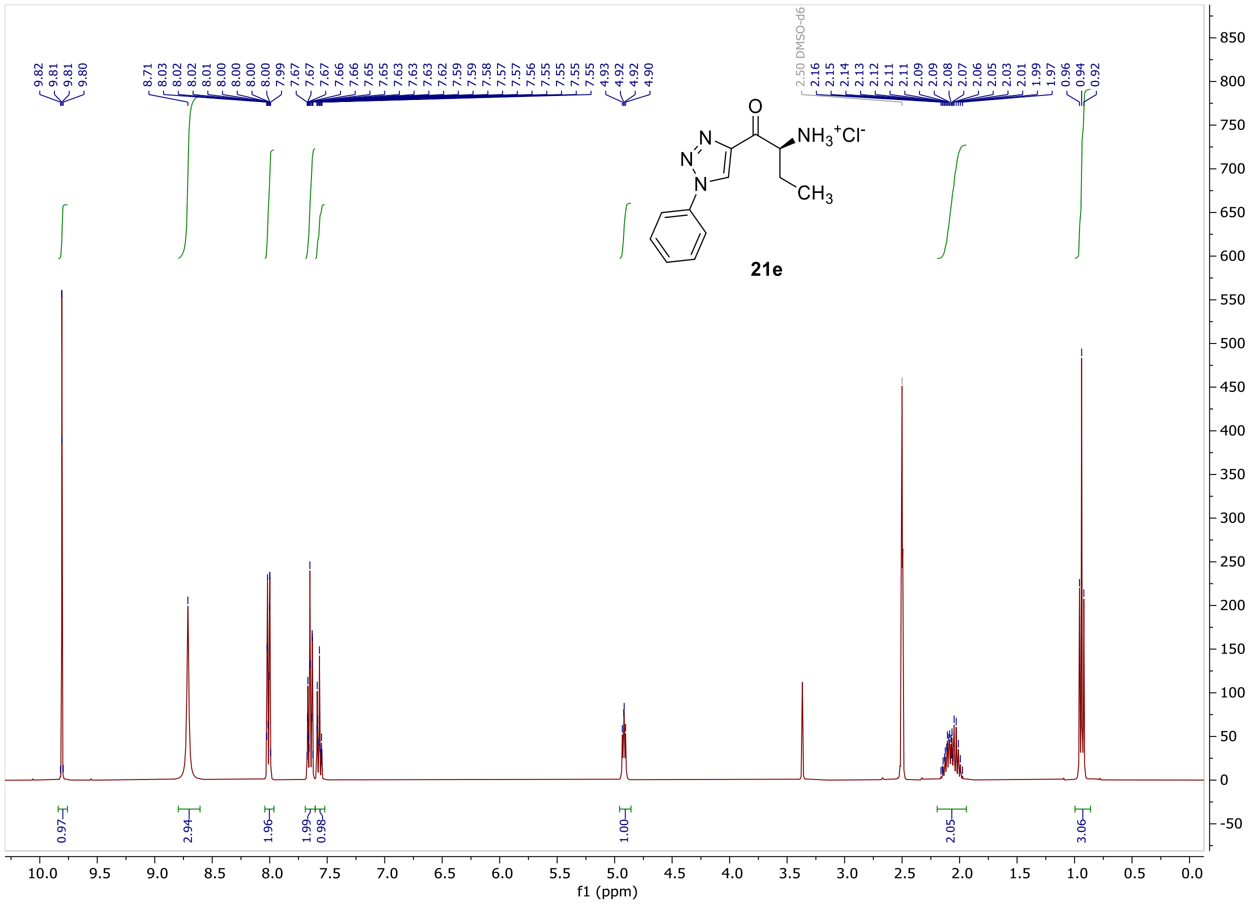

Compound **21e** (^{13}C NMR, 101 MHz):

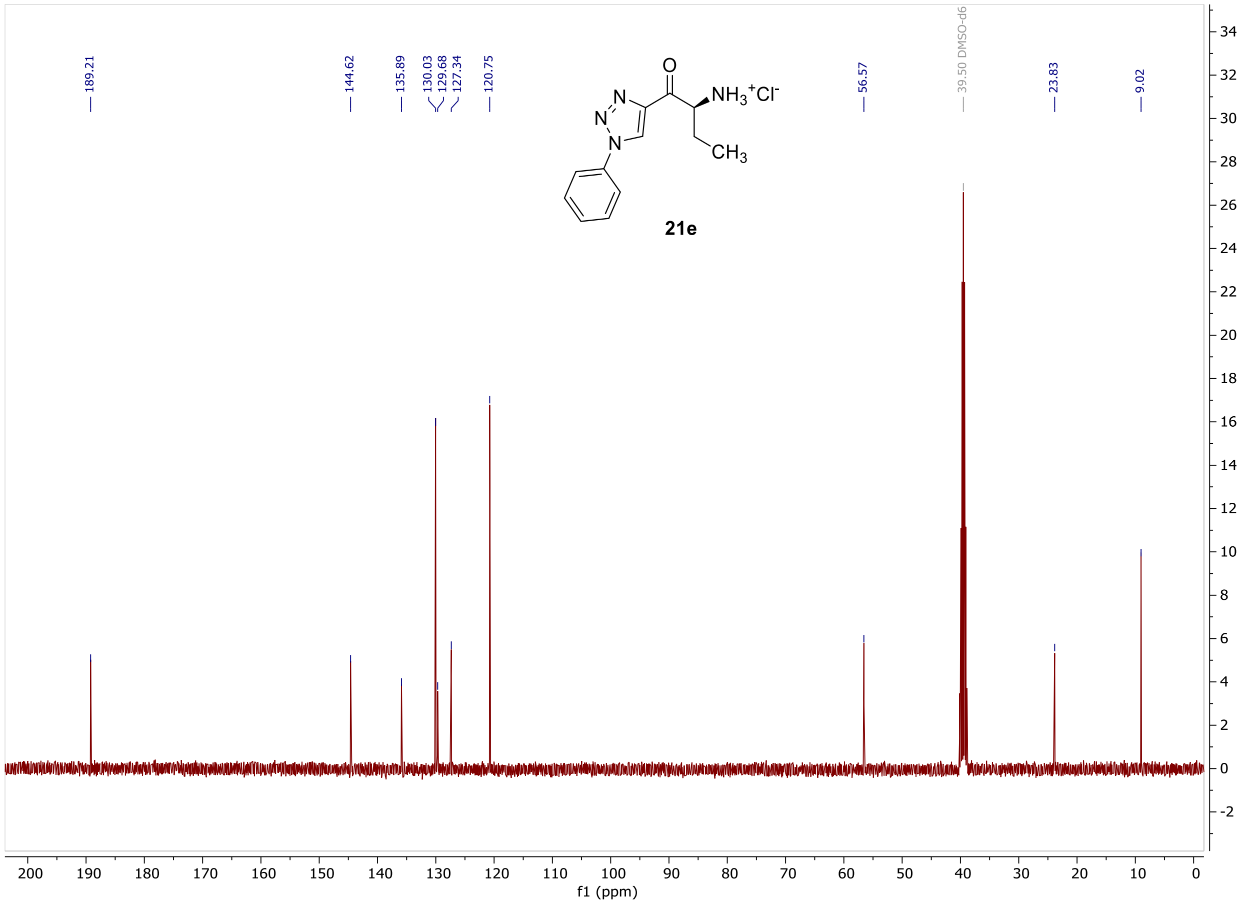

Compound **21f** (^{1}H NMR, 400 MHz):

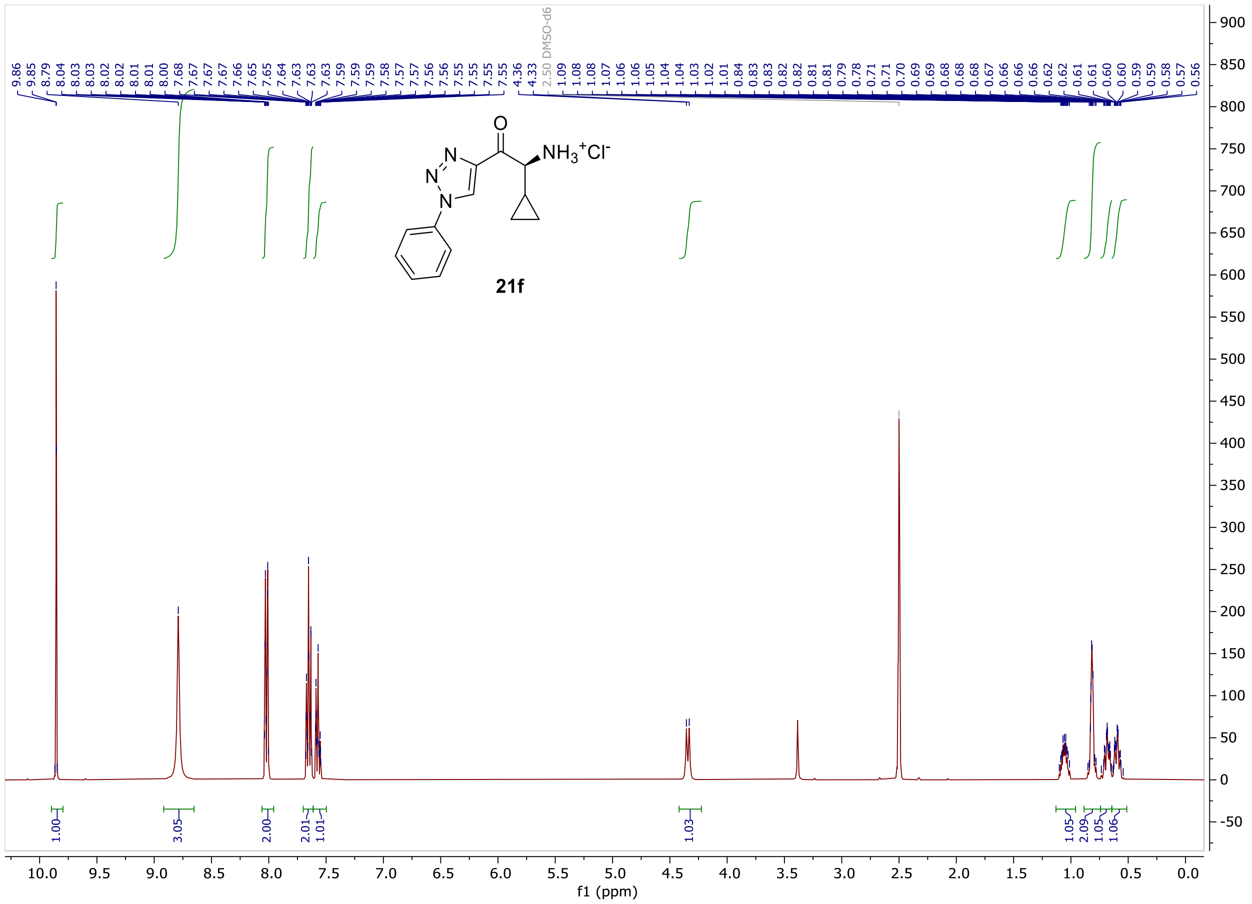

Compound **21f** (^{13}C NMR, 101 MHz):

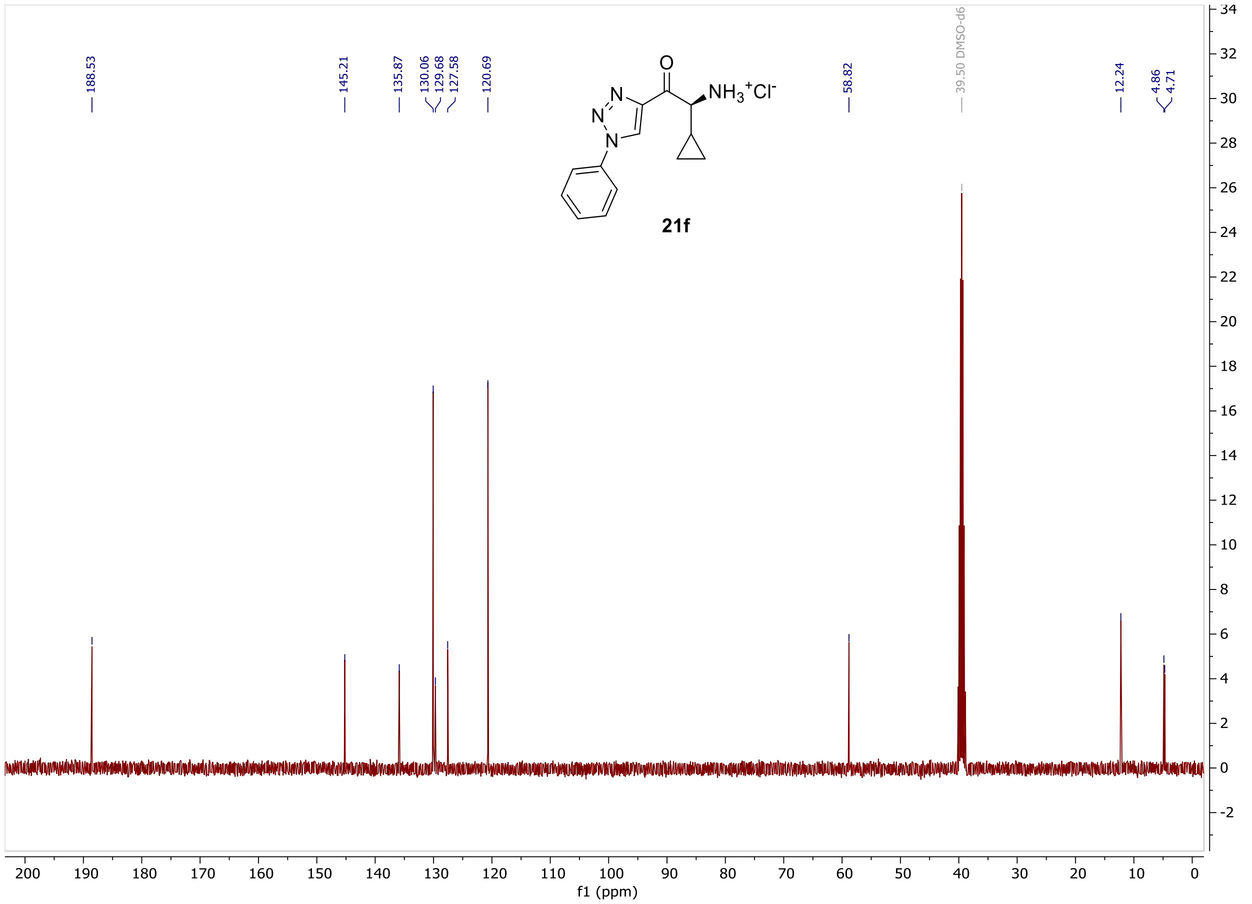

Compound **28a** (^{1}H NMR, 400 MHz):

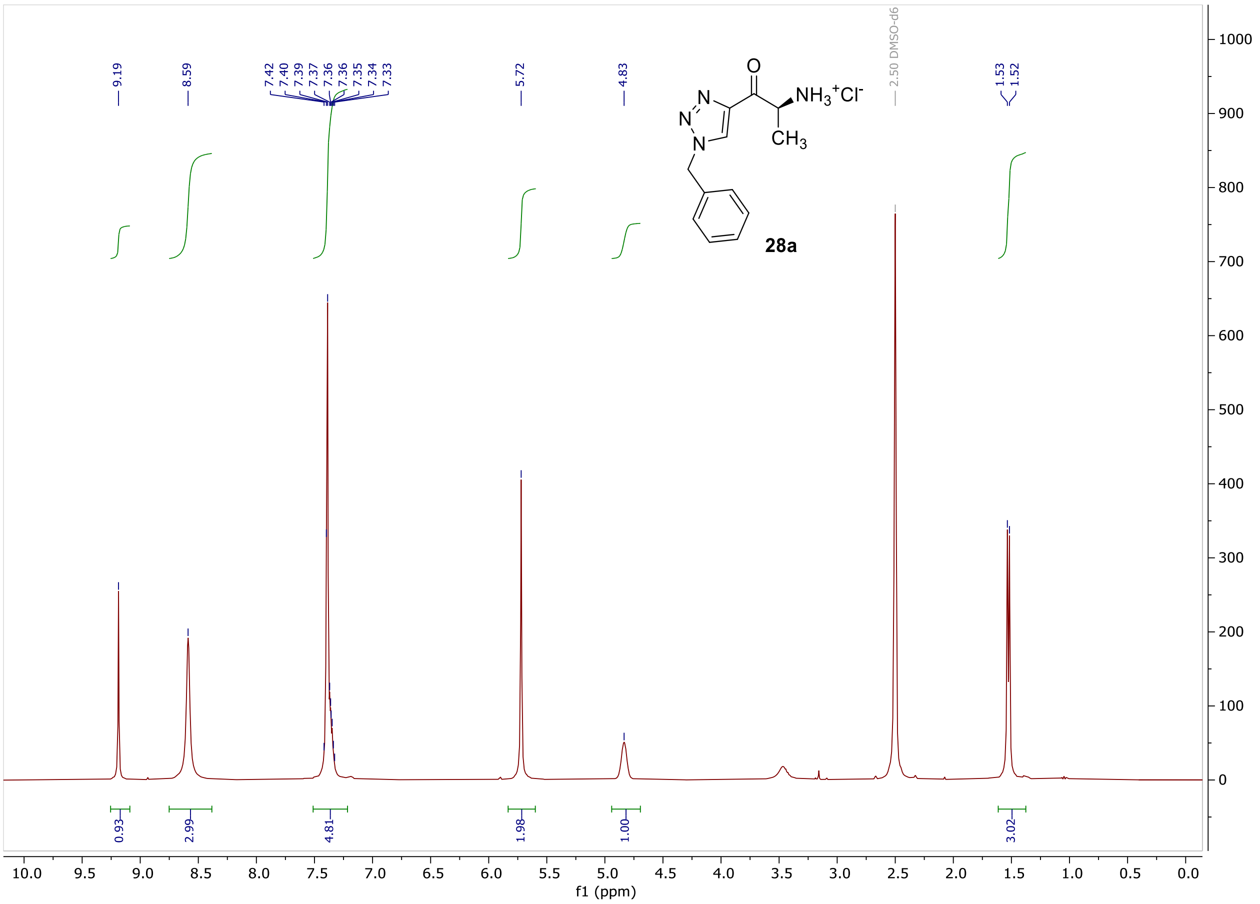

Compound **28a** (^{13}C NMR, 101 MHz):

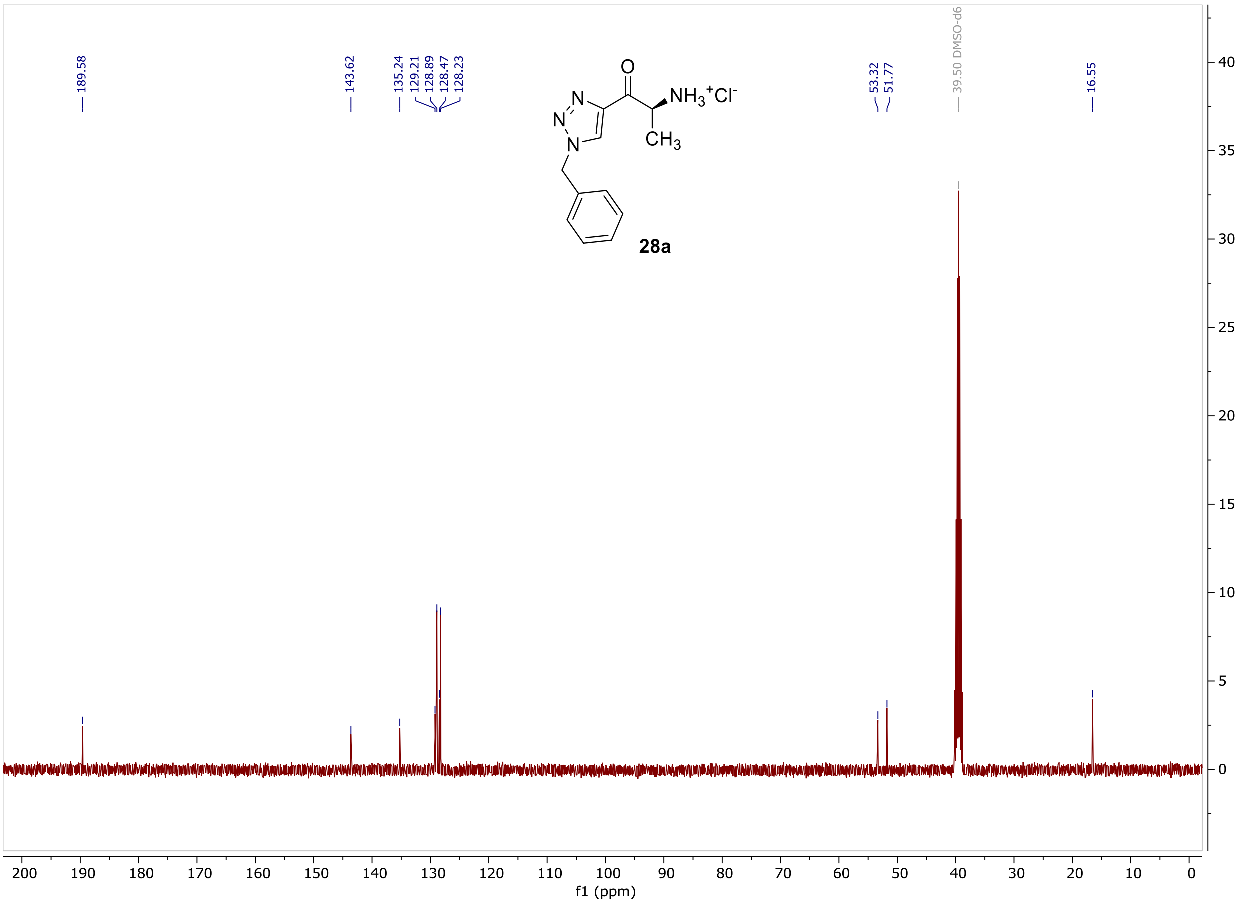

Compound **28b** (^{1}H NMR, 400 MHz):

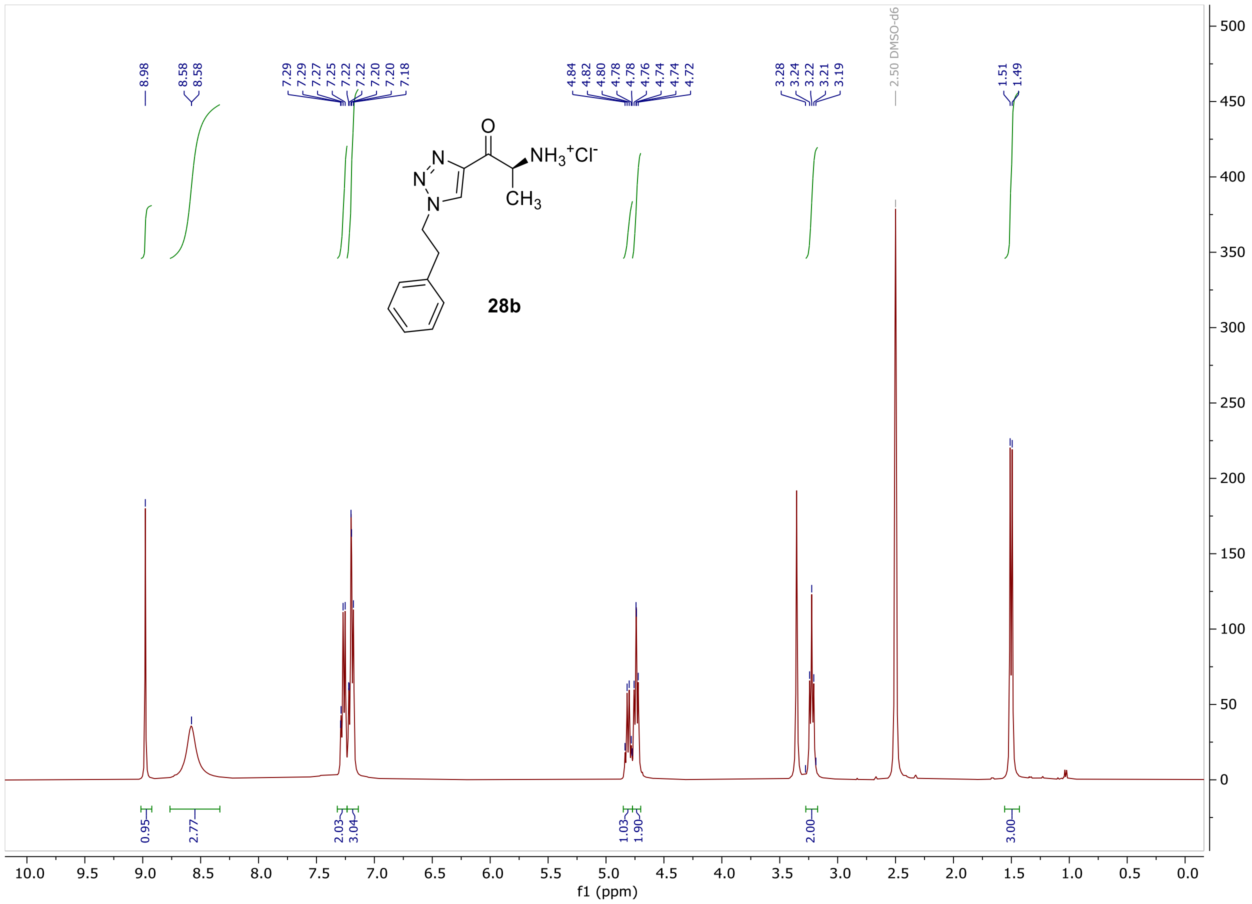

Compound **28b** (^{13}C NMR, 101 MHz):

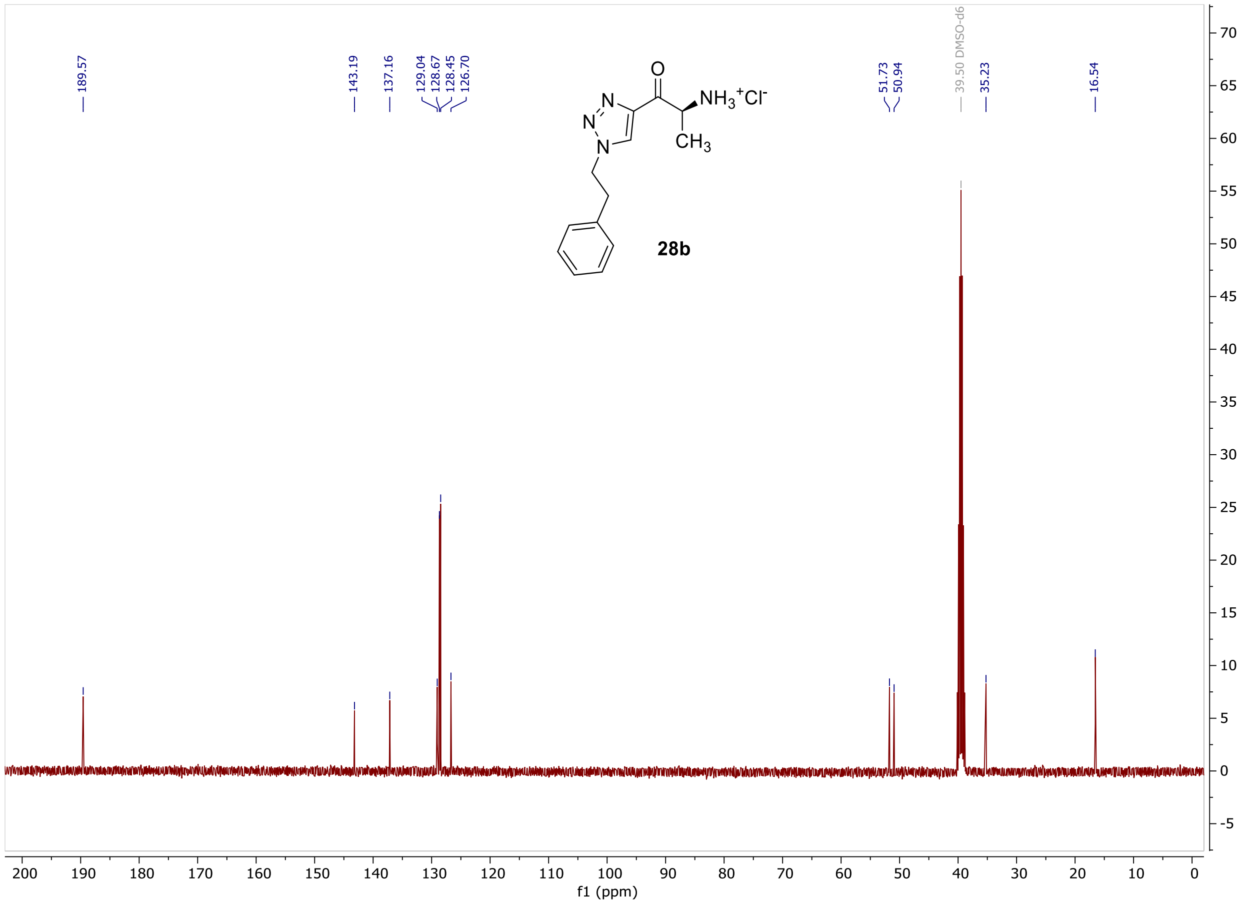

Compound **26a** (^{1}H NMR, 400 MHz):

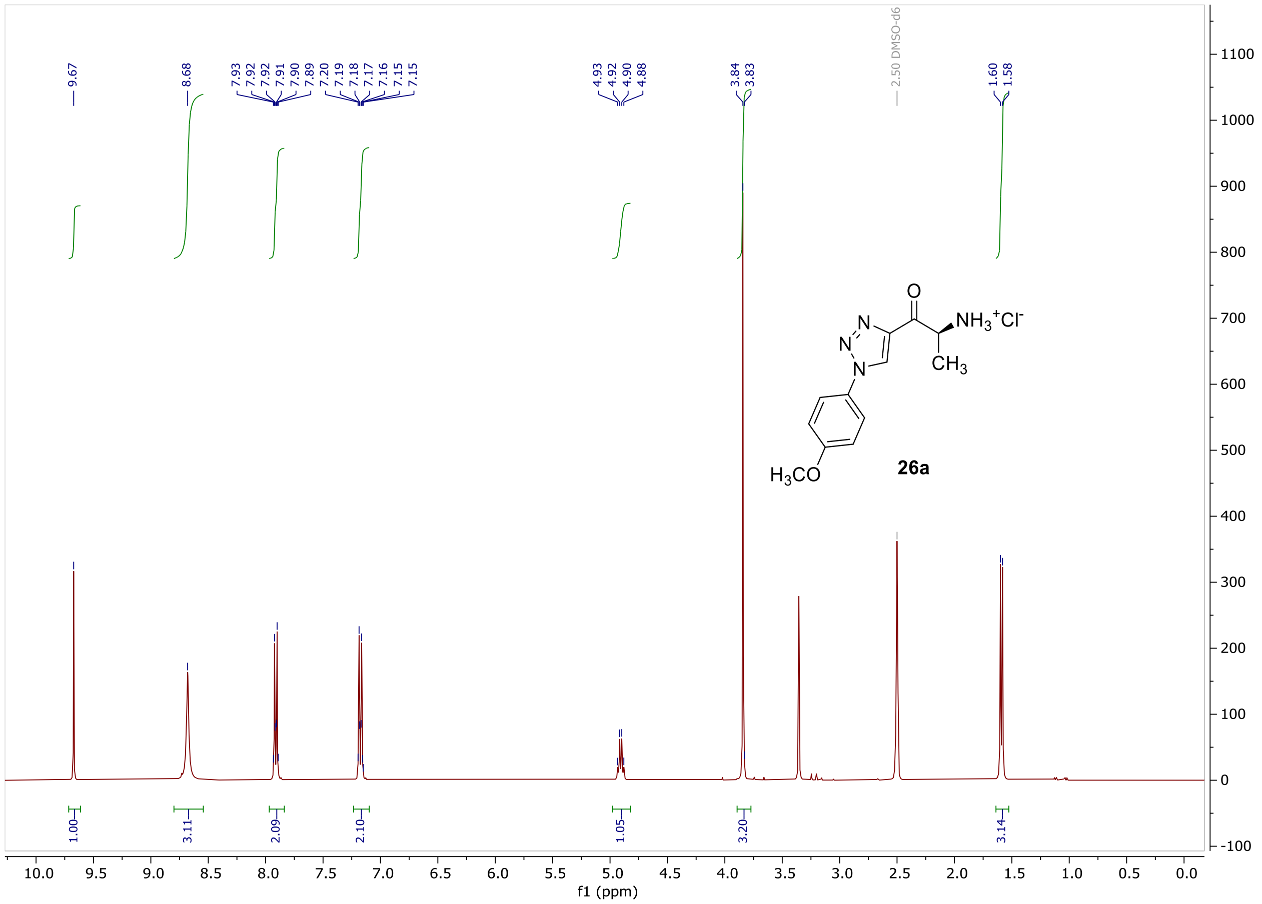

Compound **26a** (^{13}C NMR, 101 MHz):

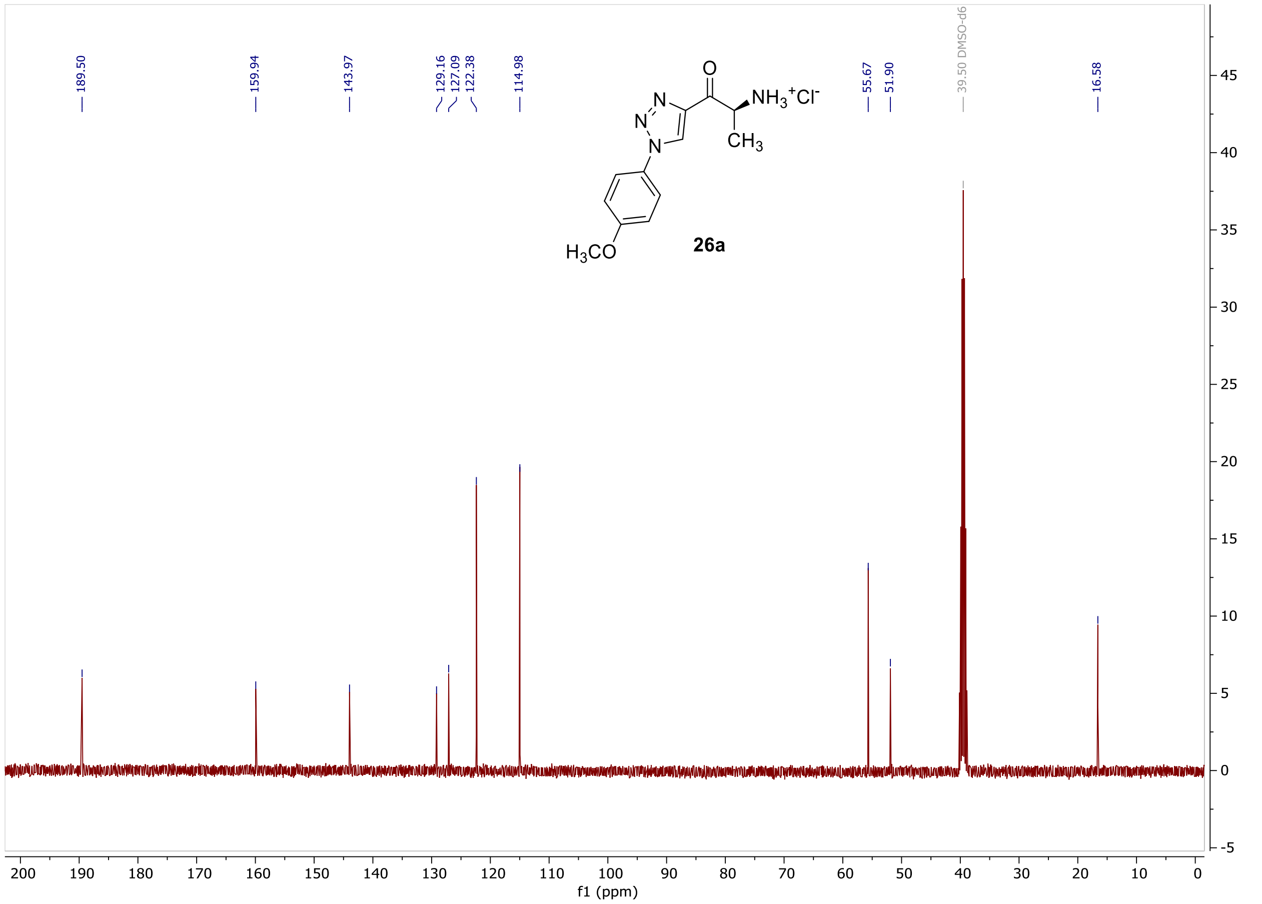

Compound **26b** (^{1}H NMR, 400 MHz):

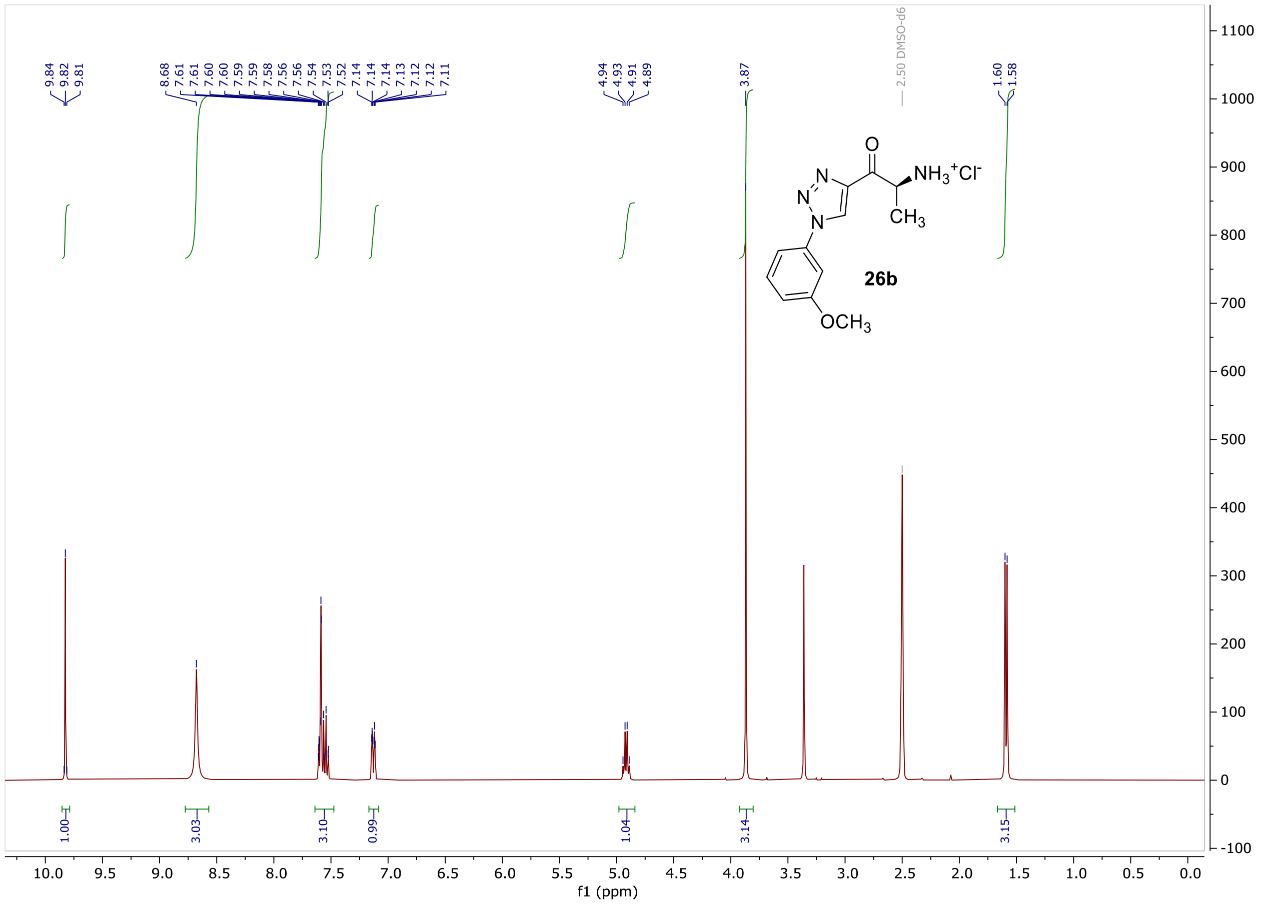

Compound **26b** (^{13}C NMR, 101 MHz):

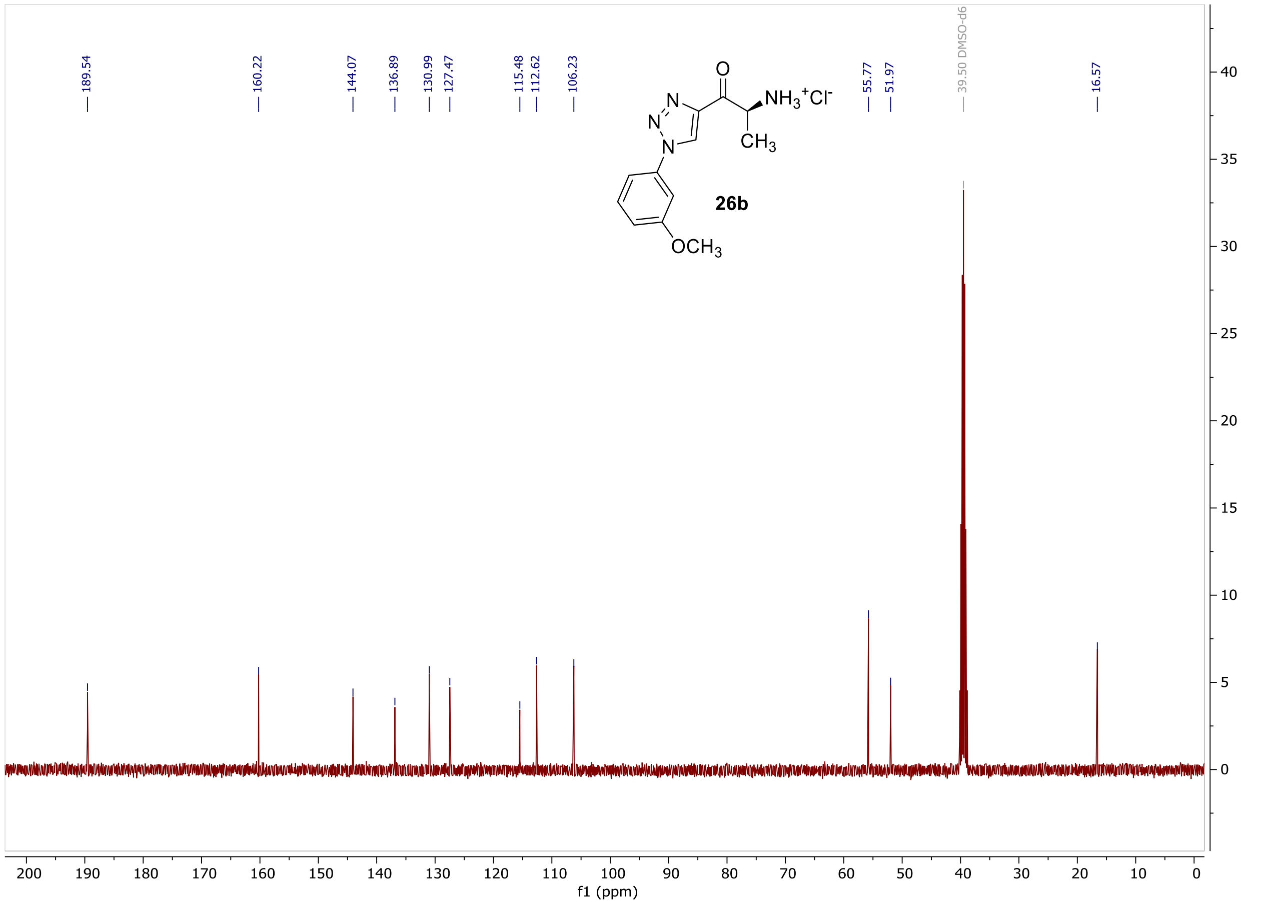

Compound **26c** (^{1}H NMR, 400 MHz):

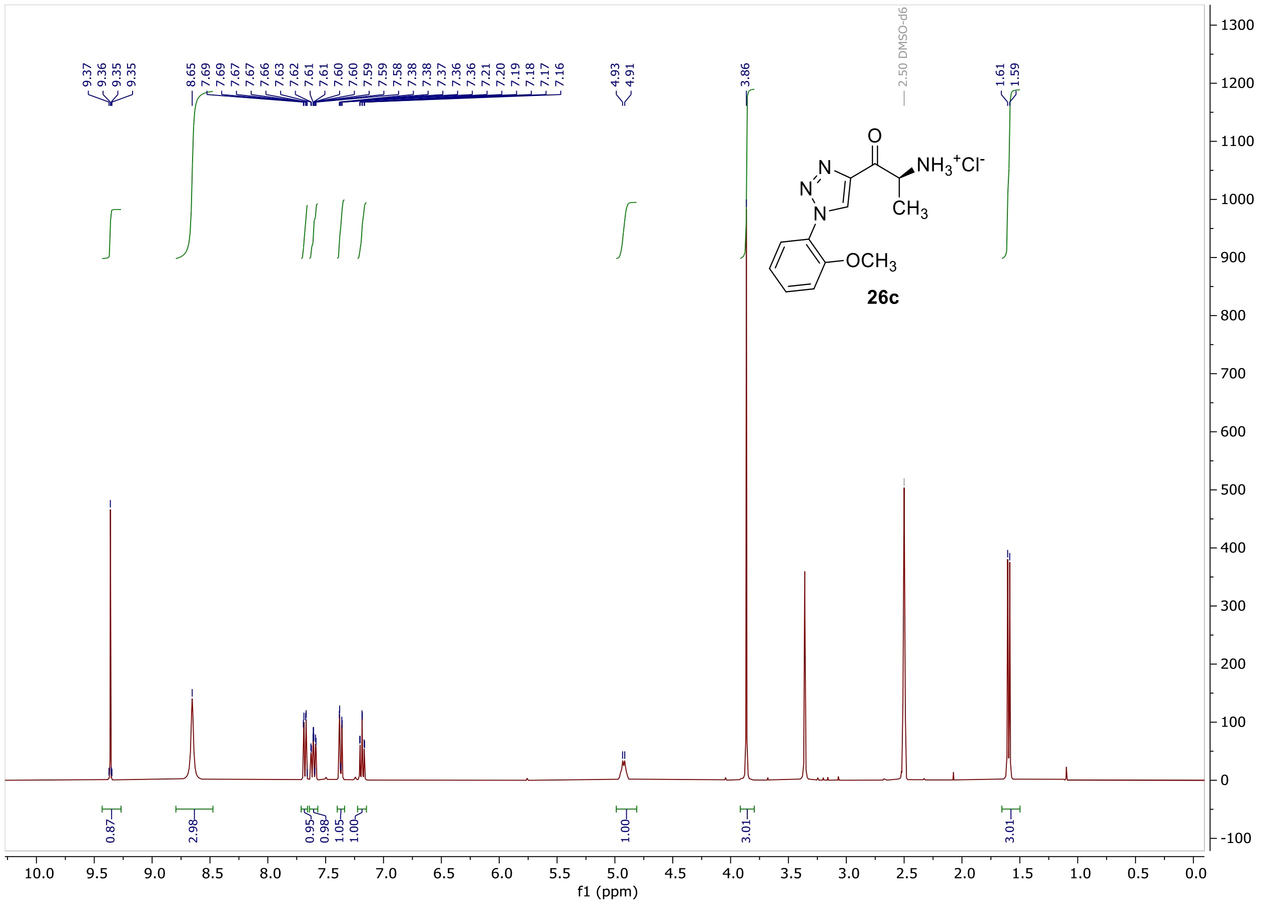

Compound **26c** (^{13}C NMR, 101 MHz):

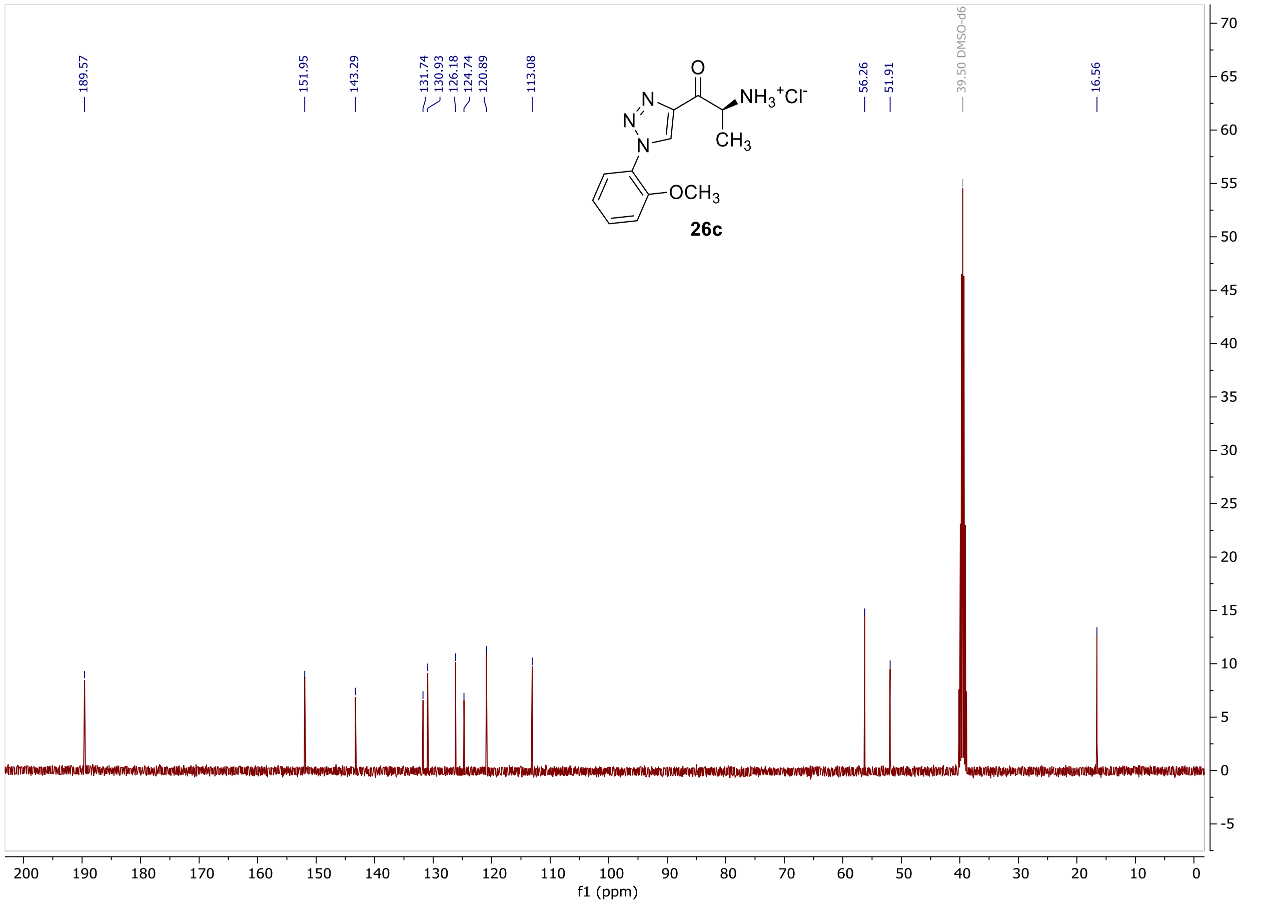

Compound **26d** (^{1}H NMR, 400 MHz):

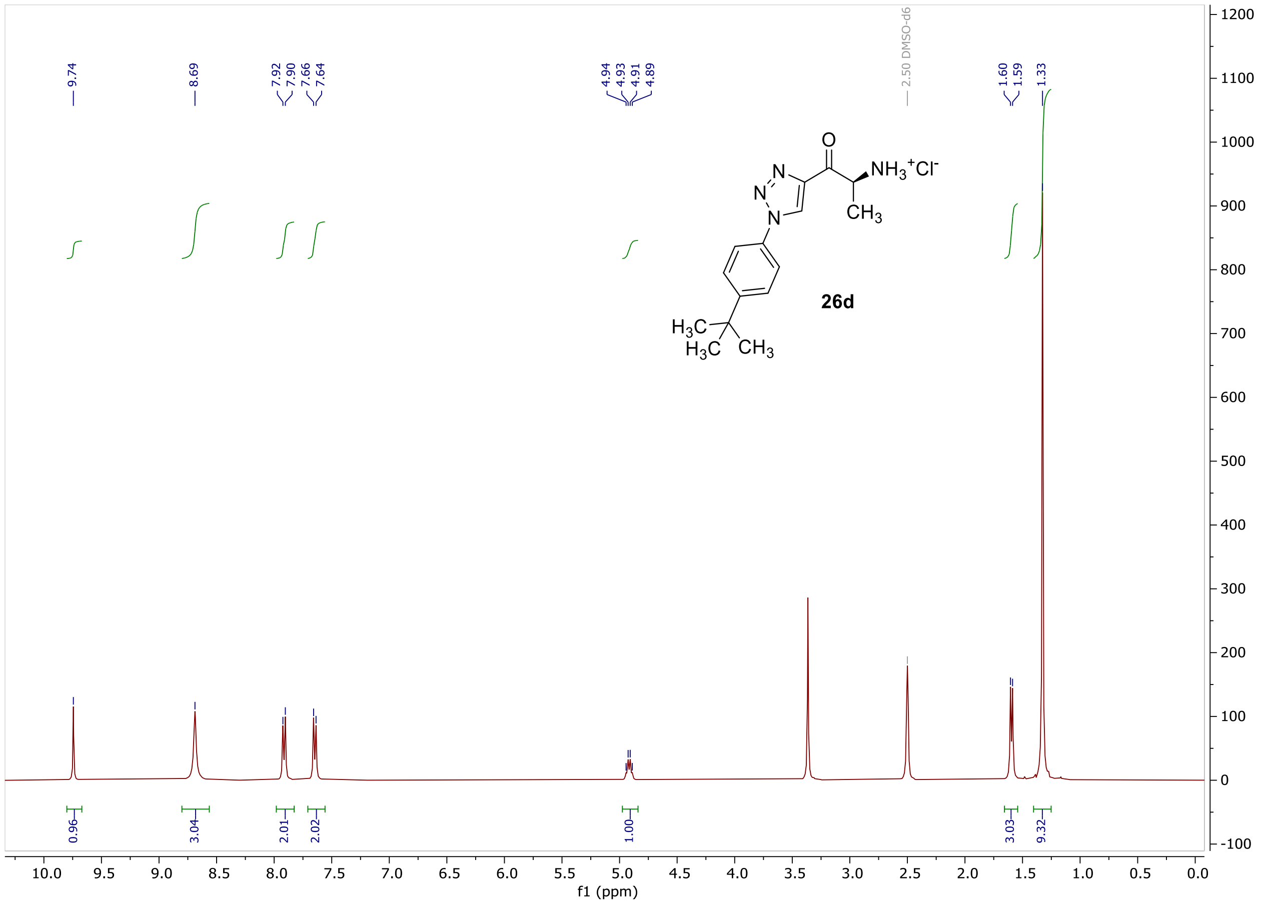

Compound **26d** (^{13}C NMR, 101 MHz):

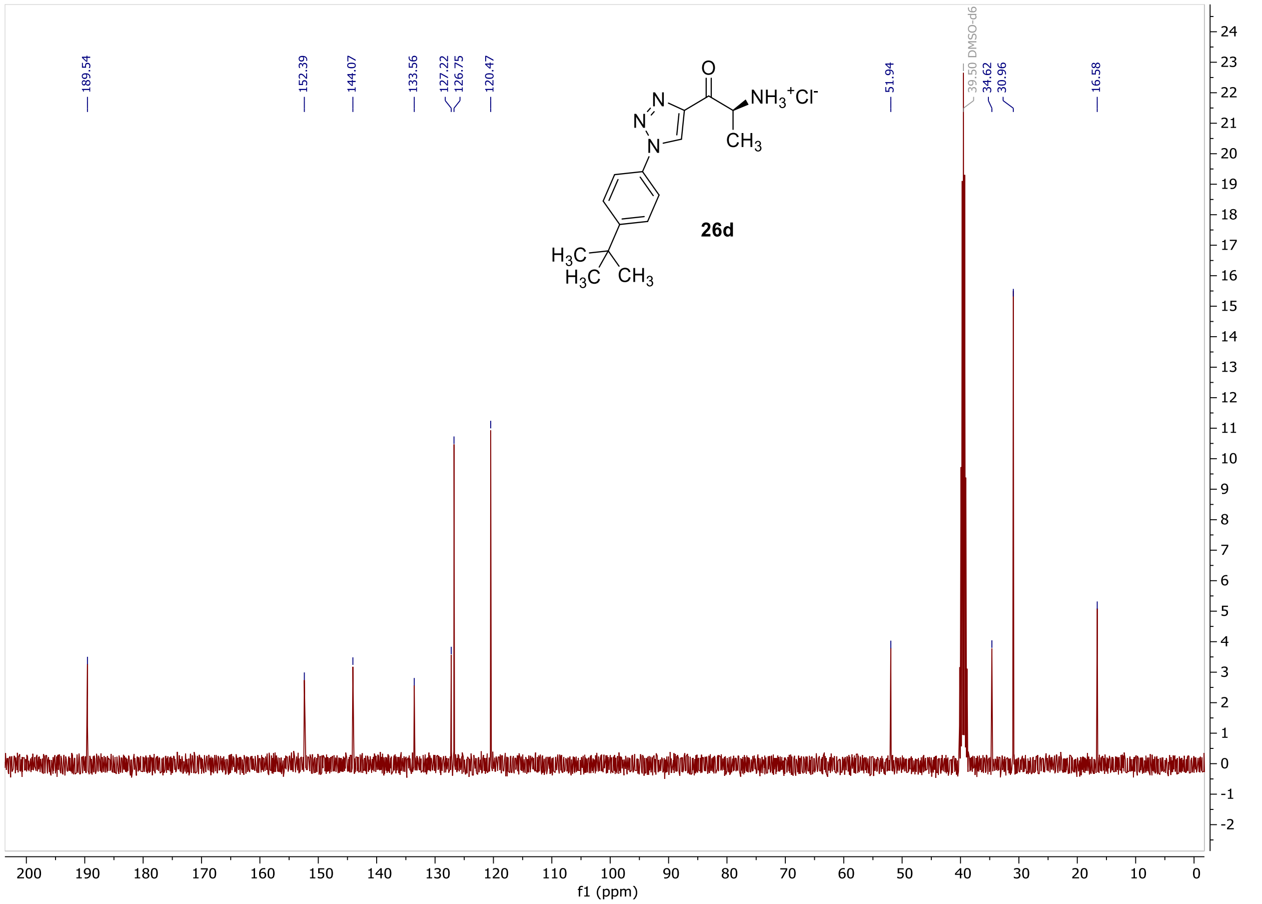

Compound **26e** (^{1}H NMR, 400 MHz):

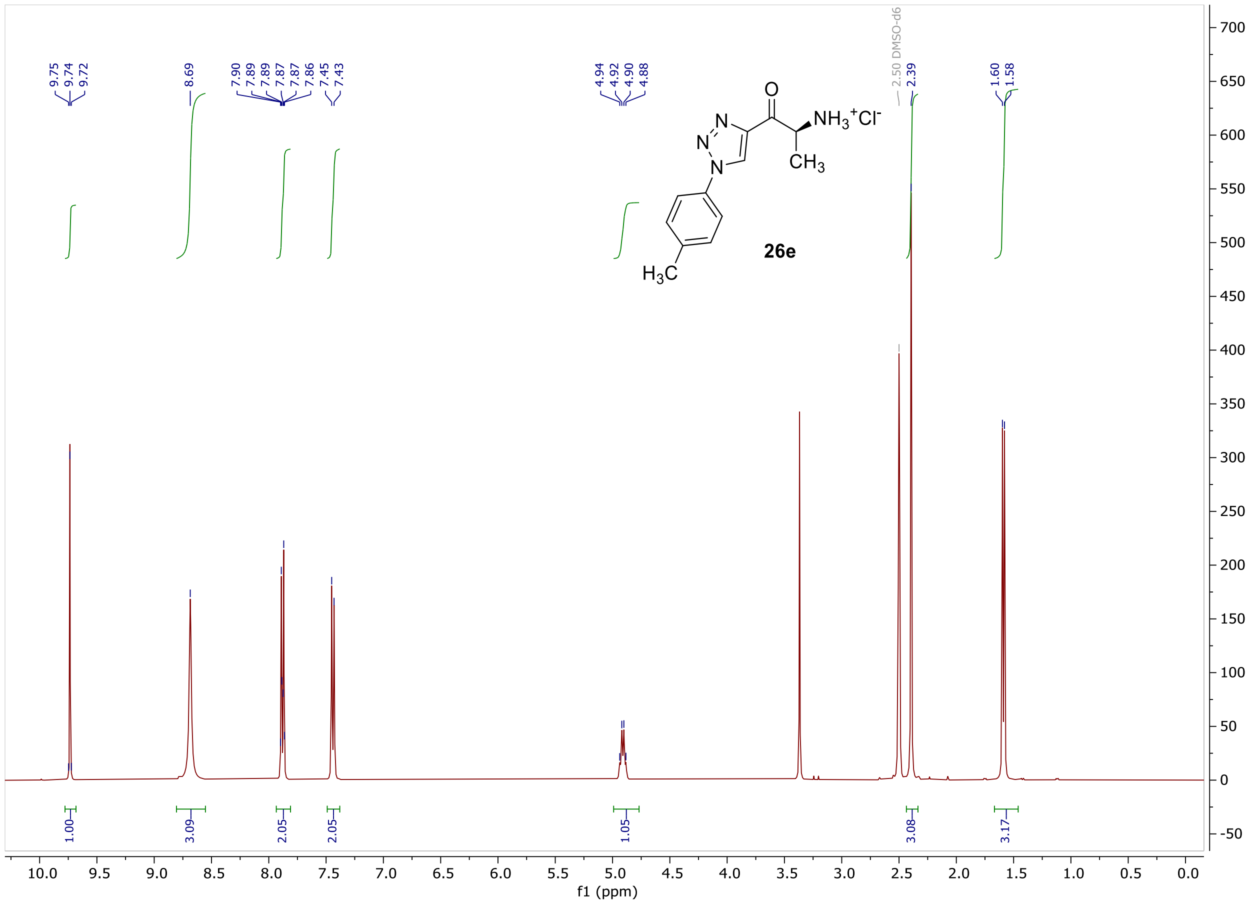

Compound **26e** (^{13}C NMR, 101 MHz):

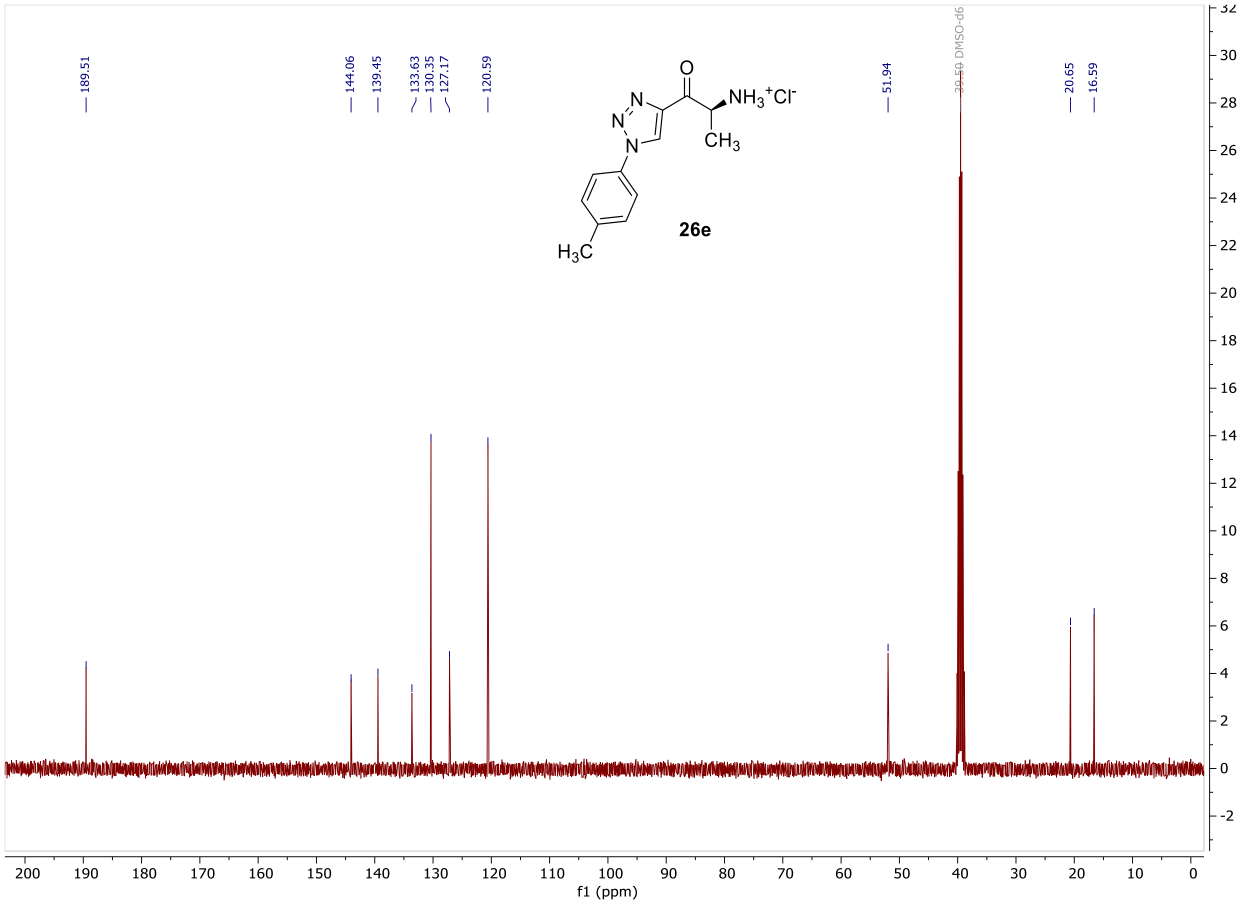

Compound **26f** (^{1}H NMR, 400 MHz):

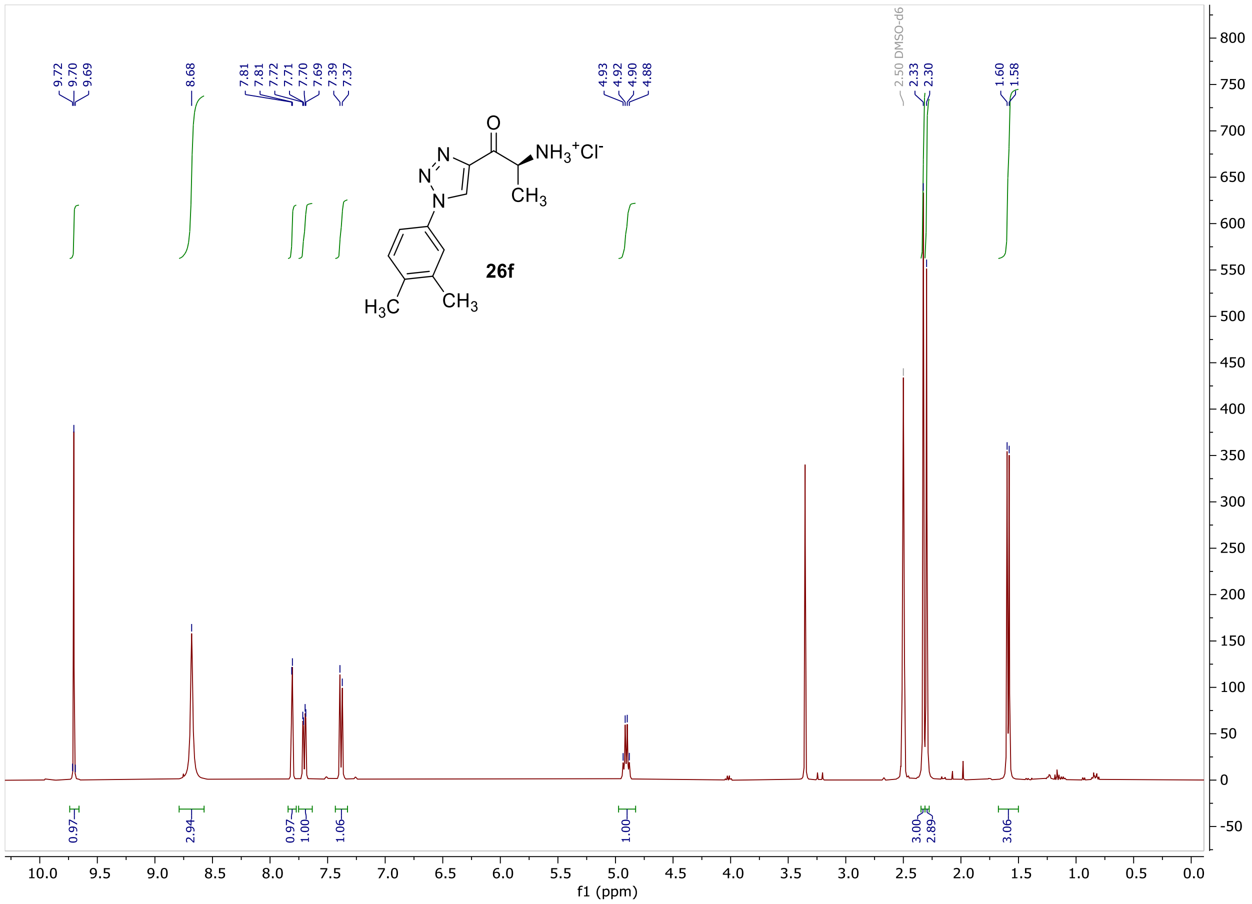

Compound **26f** (^{13}C NMR, 101 MHz):

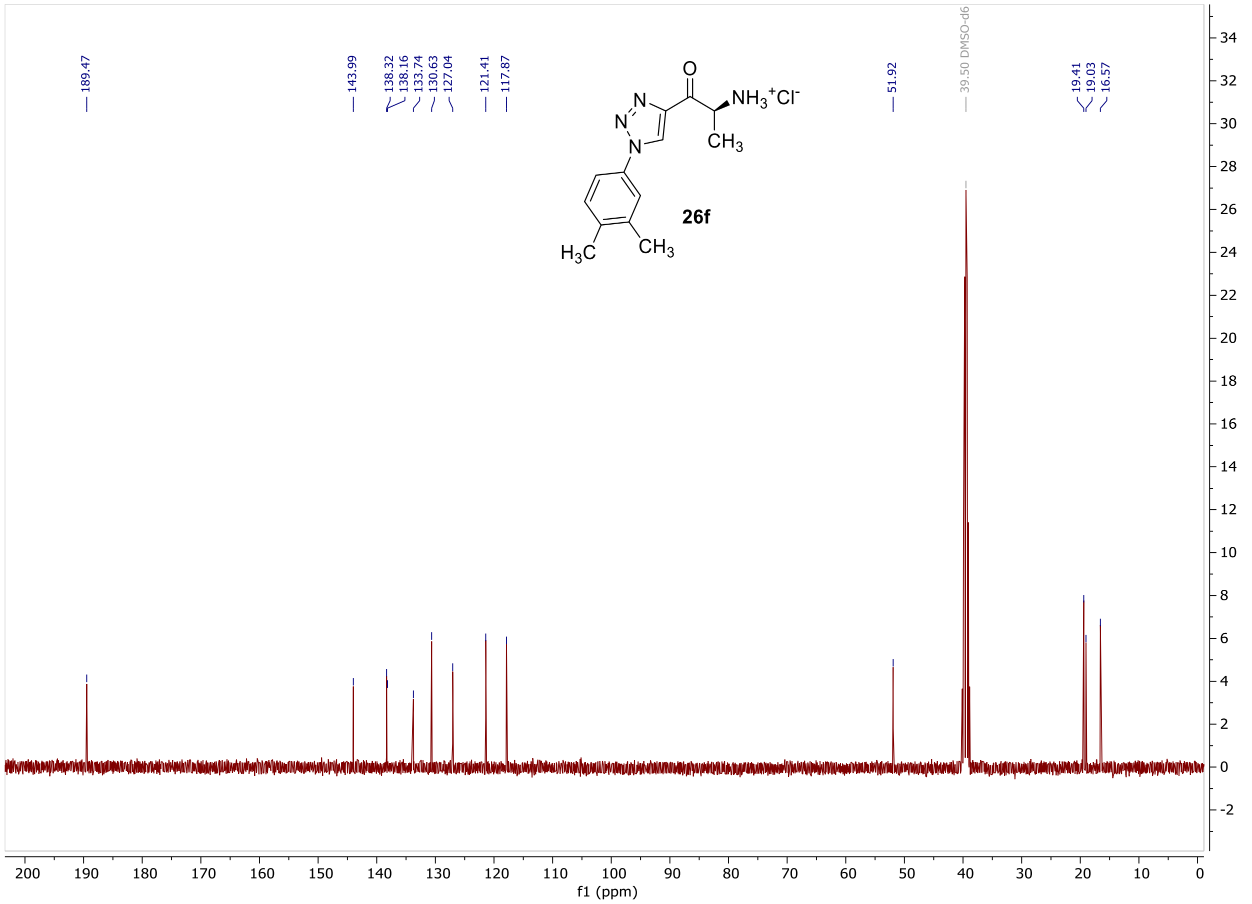

Compound **26g** (^{1}H NMR, 400 MHz):

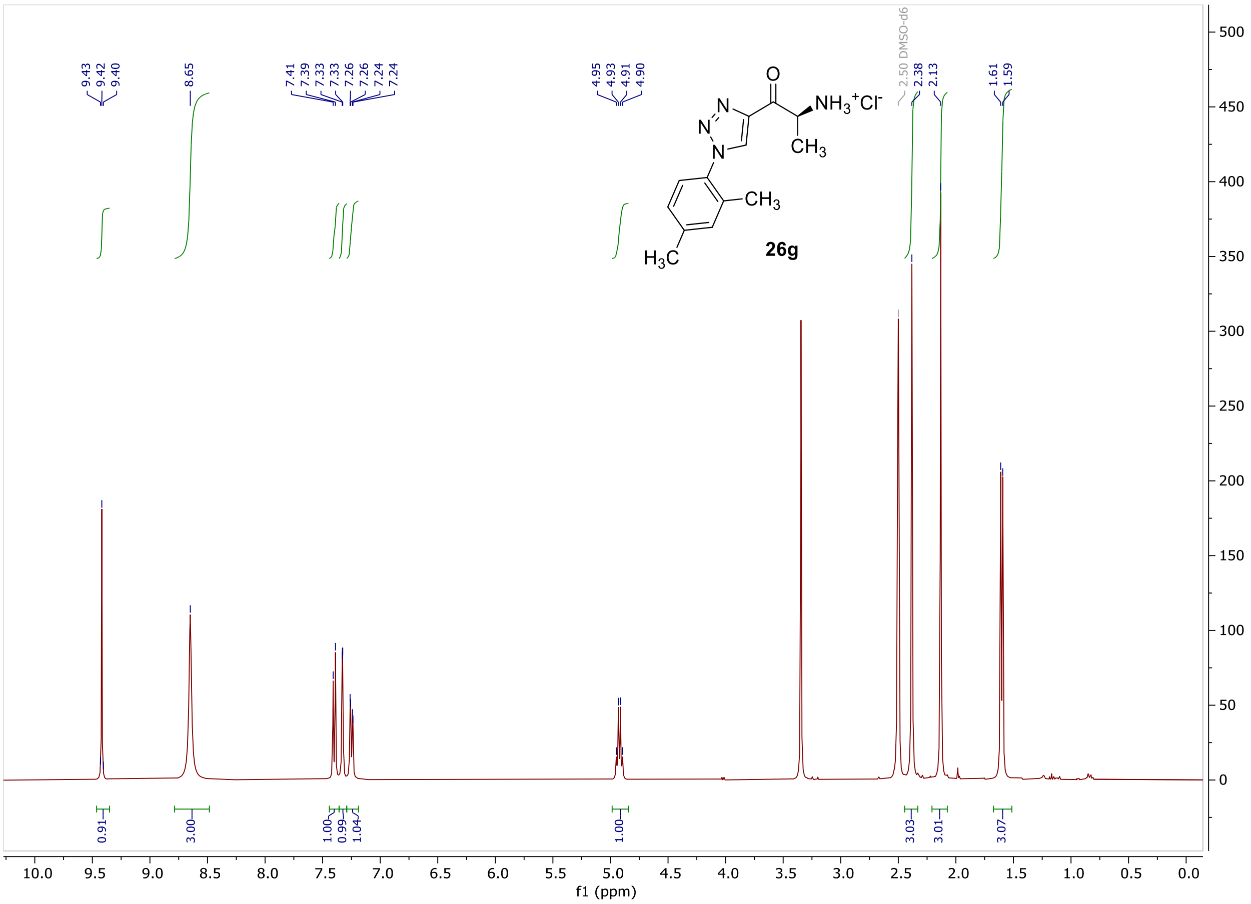

Compound **26g** (^{13}C NMR, 101 MHz):

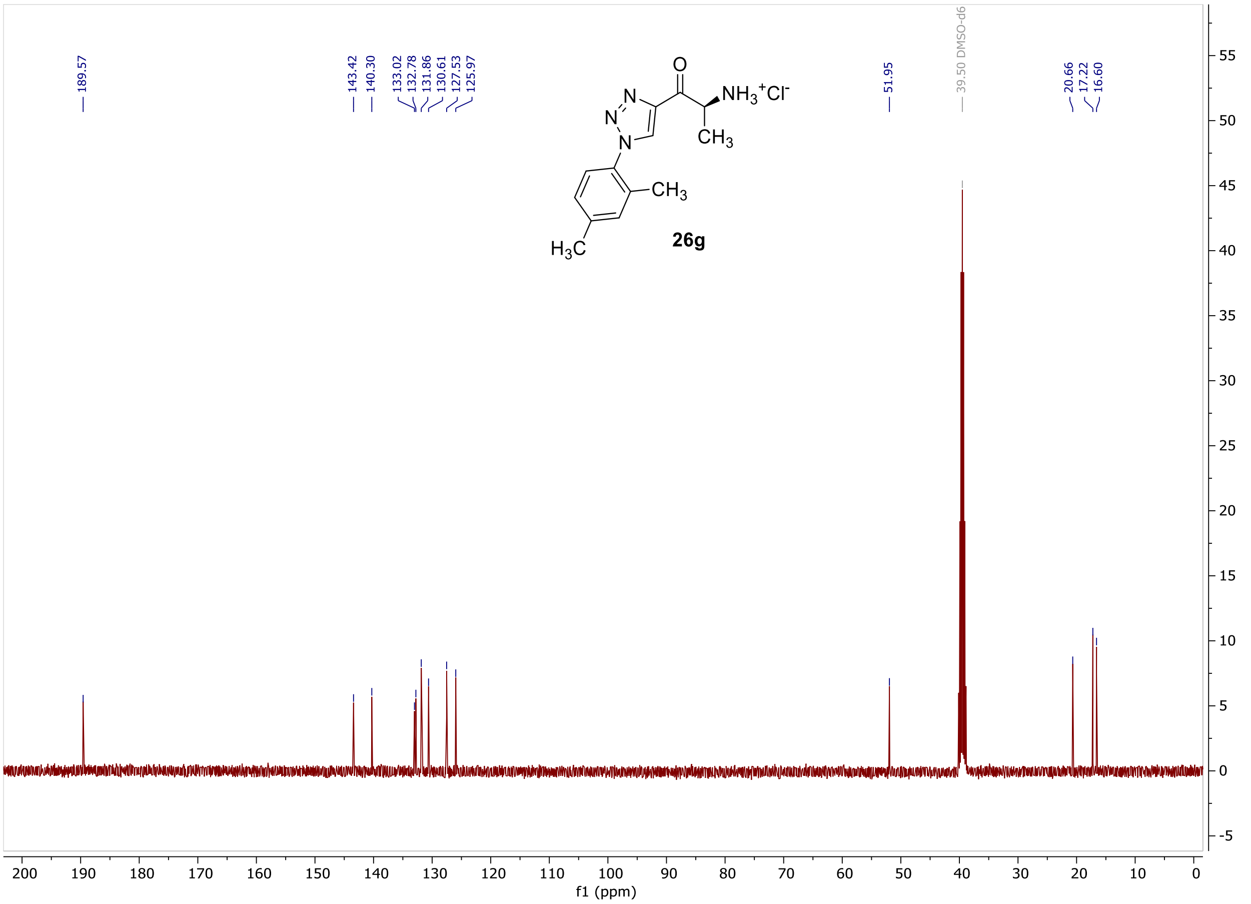

Compound **26h** (^{1}H NMR, 400 MHz):

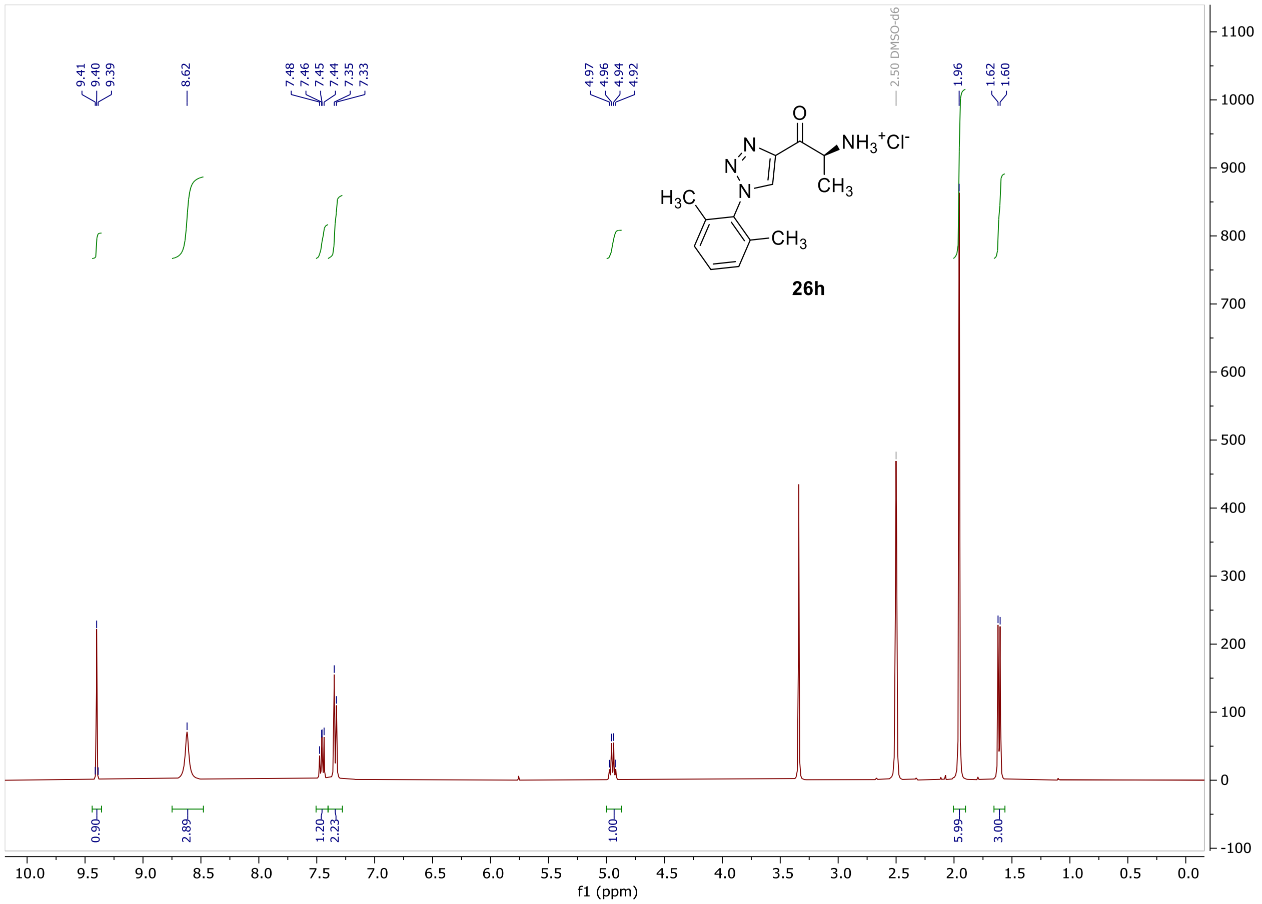

Compound **26h** (^{13}C NMR, 101 MHz):

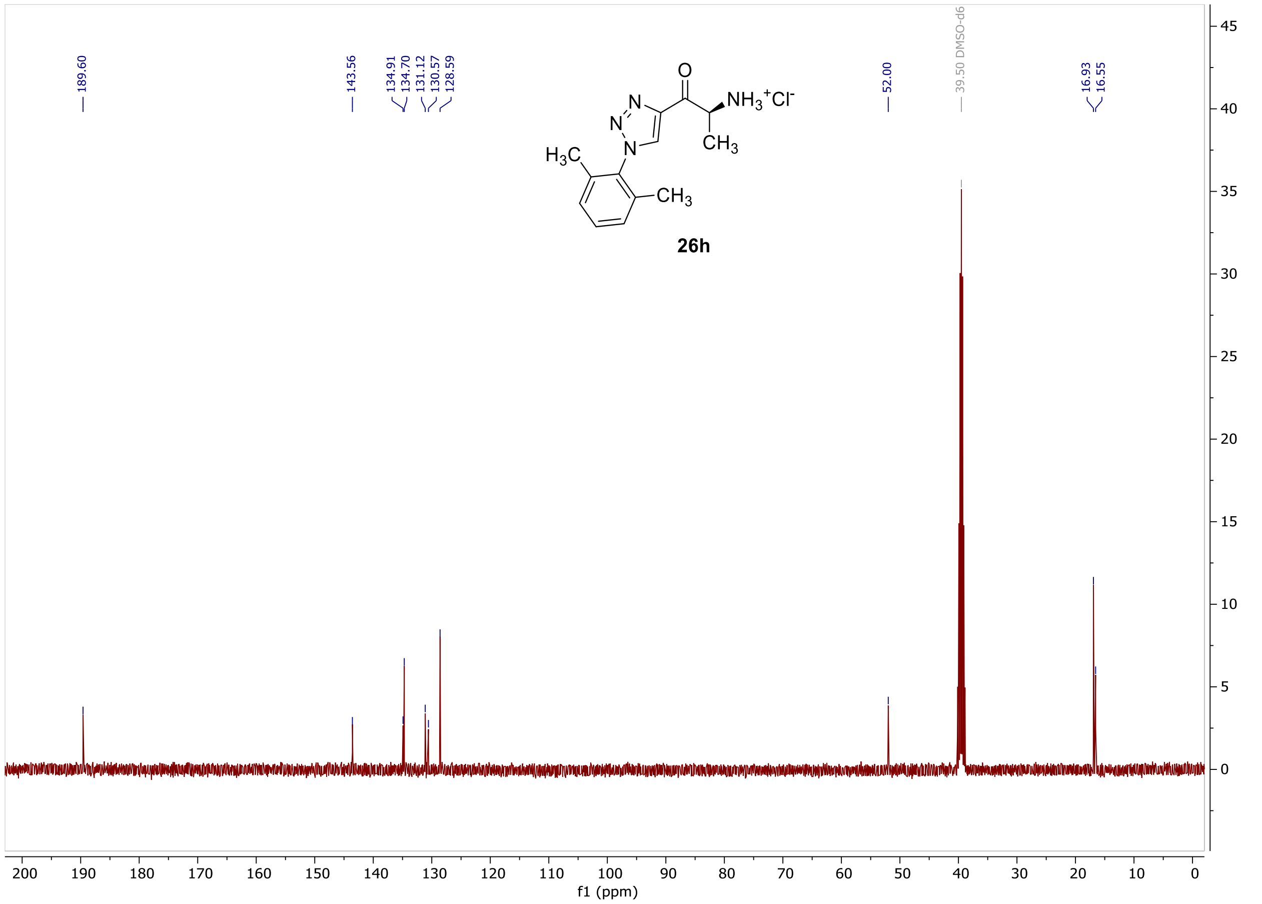

Compound **26i** (^{1}H NMR, 400 MHz):

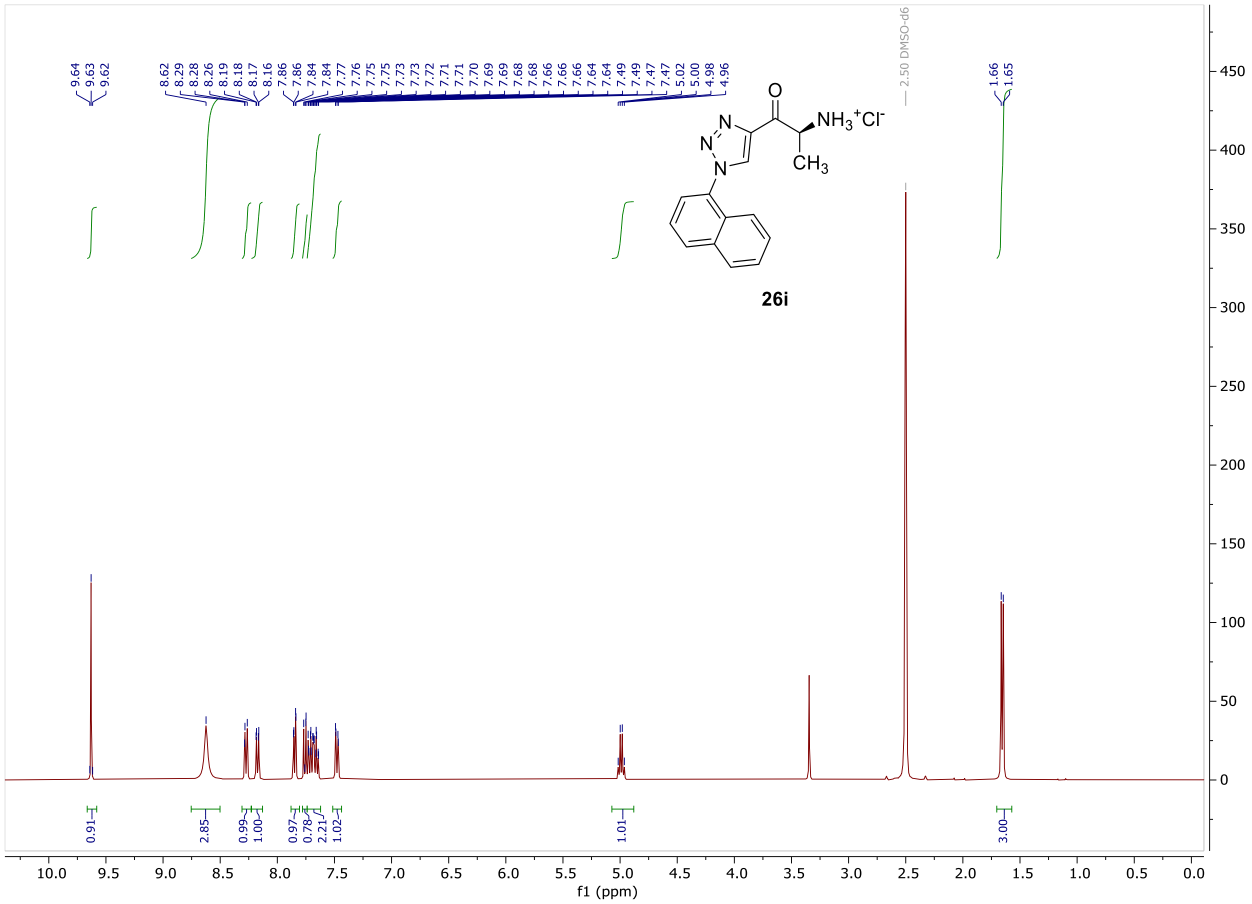

Compound **26i** (^{13}C NMR, 101 MHz):

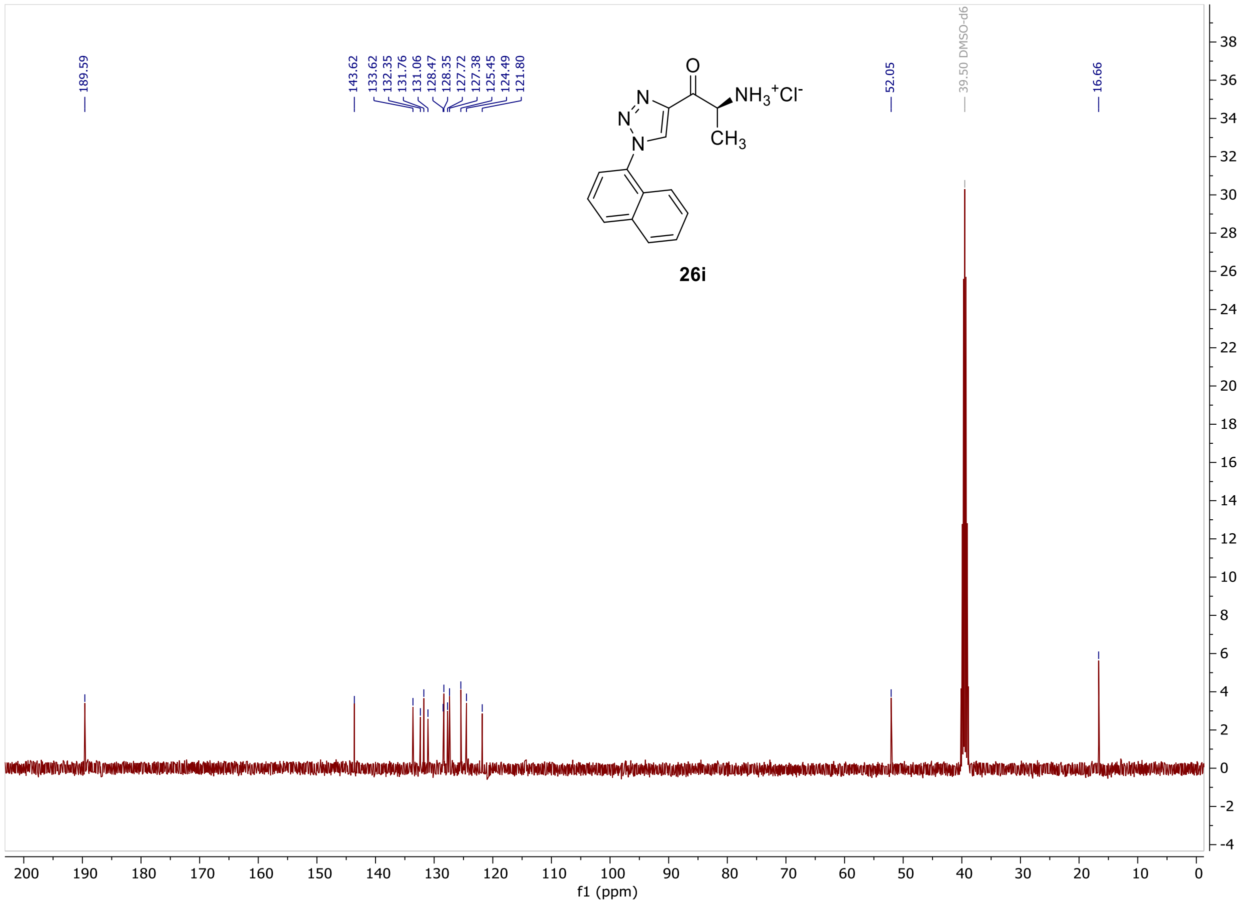

Compound **26j** (^{1}H NMR, 400 MHz):

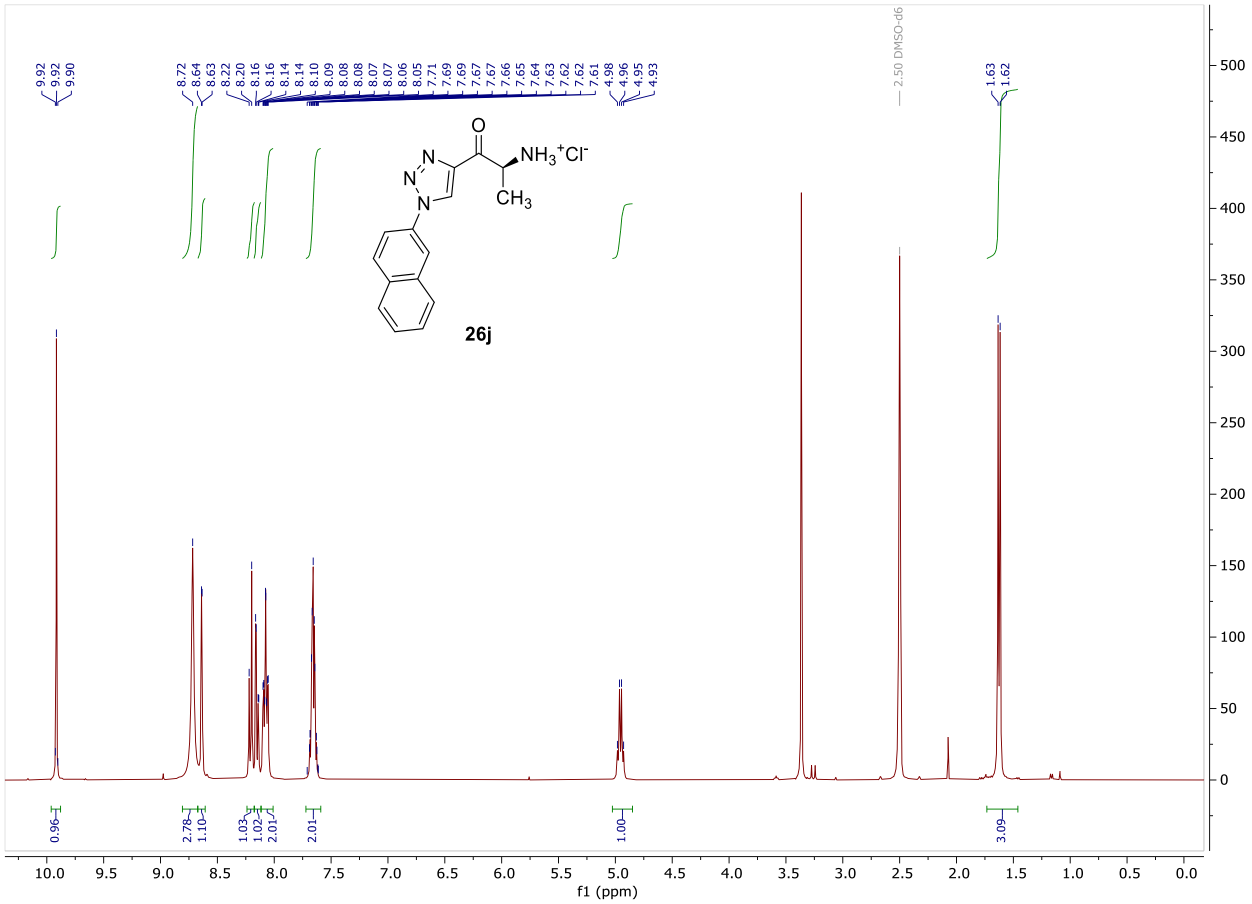

Compound **26j** (^{13}C NMR, 101 MHz):

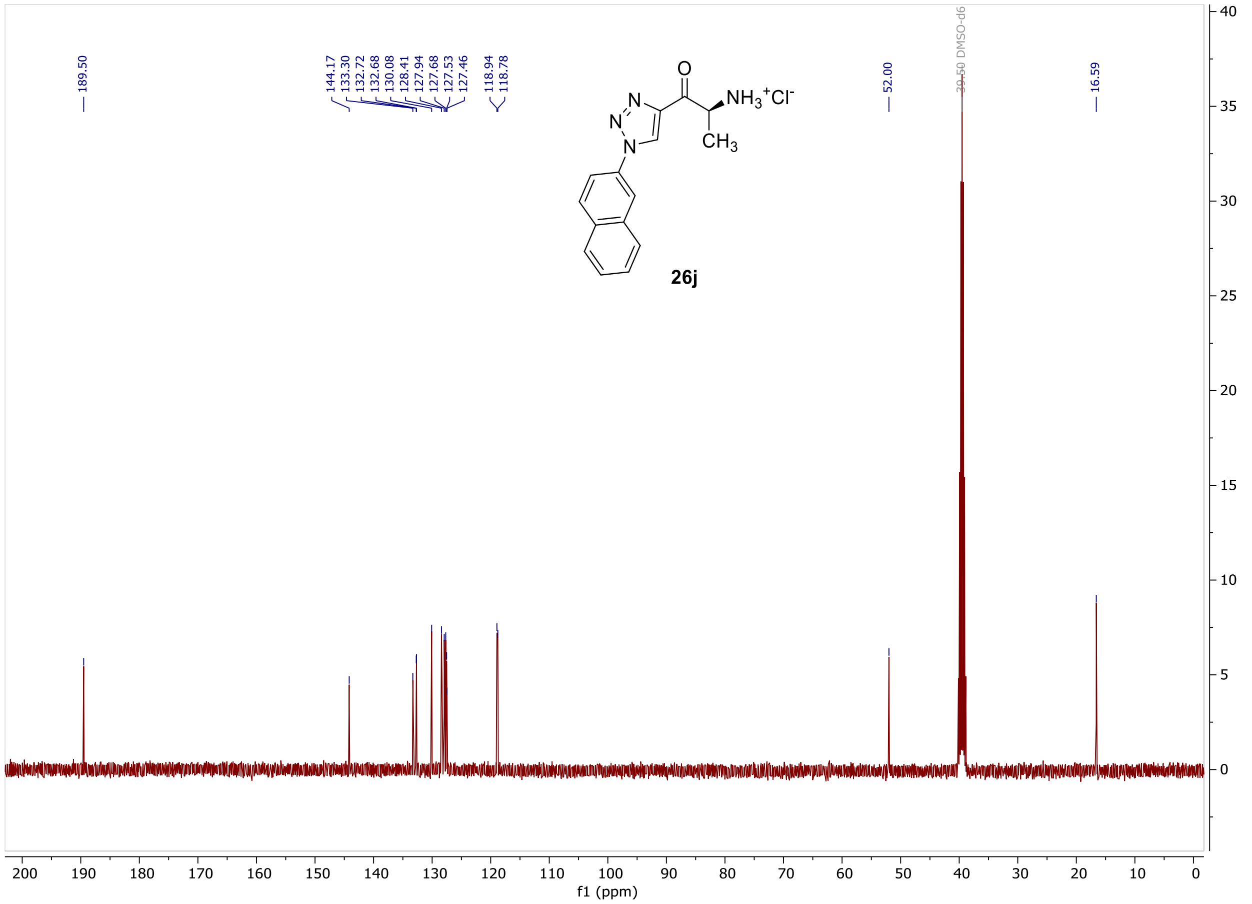

Compound **26k** (^{1}H NMR, 400 MHz):

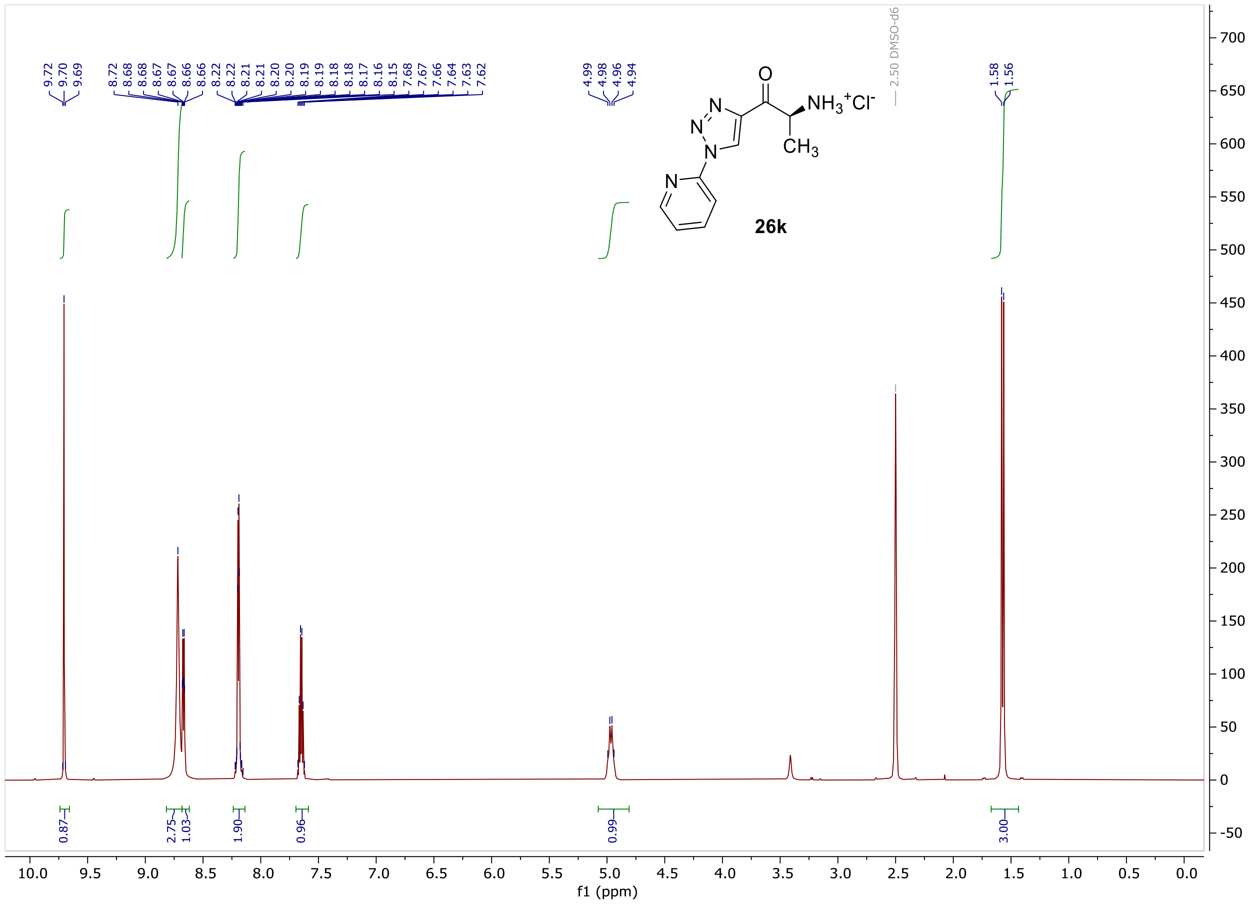

Compound **26k** (^{13}C NMR, 101 MHz):

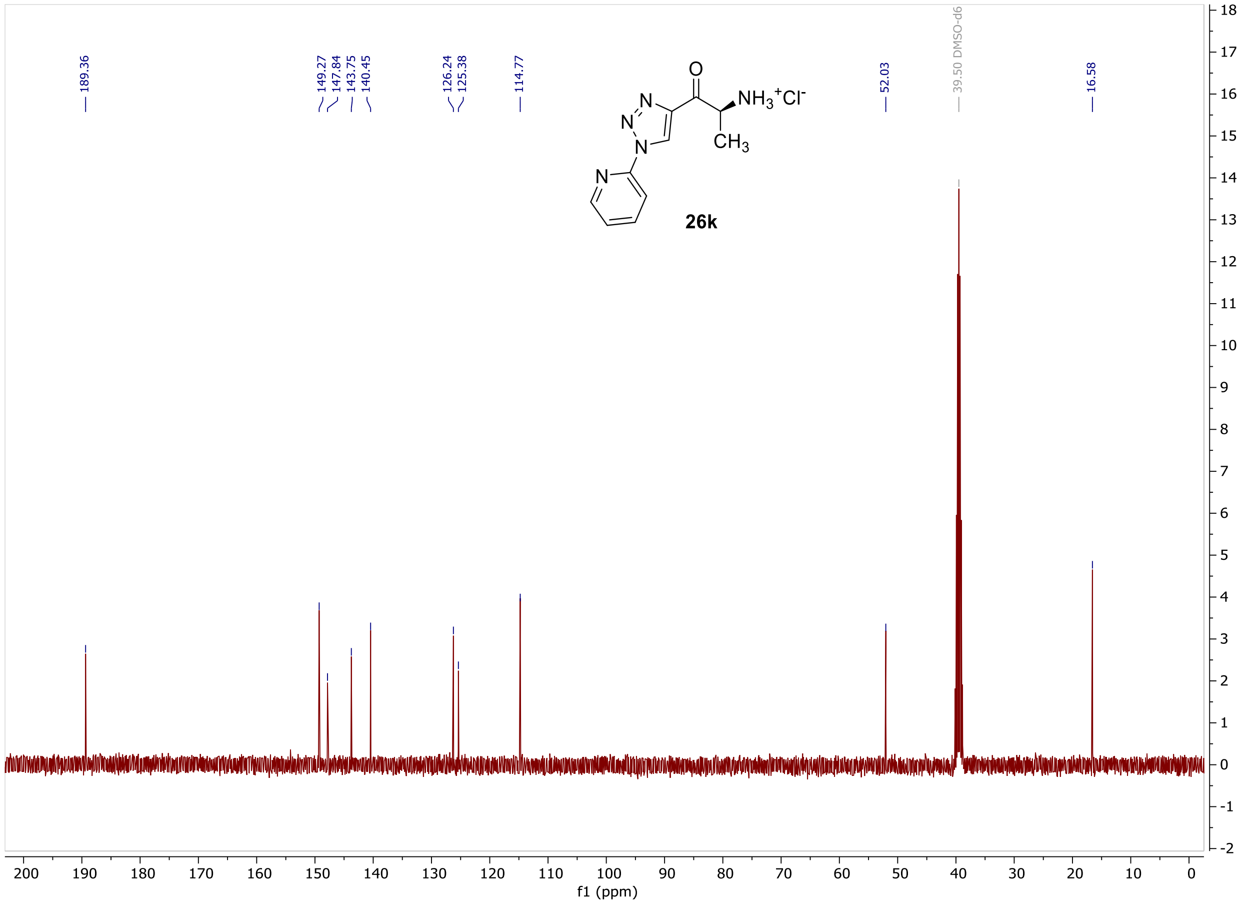

Compound **26l** (^{1}H NMR, 400 MHz):

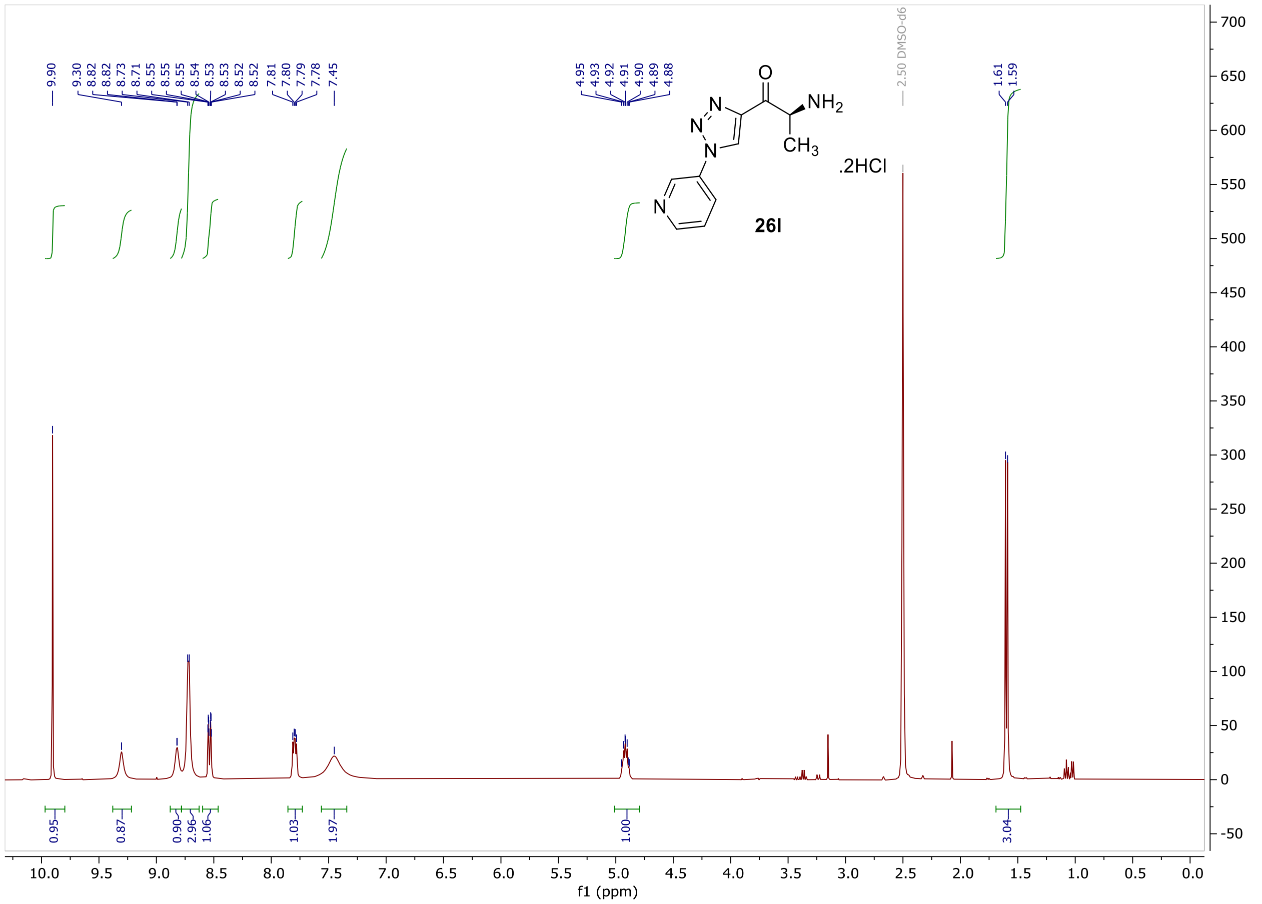

Compound **26l** (^{13}C NMR, 101 MHz):

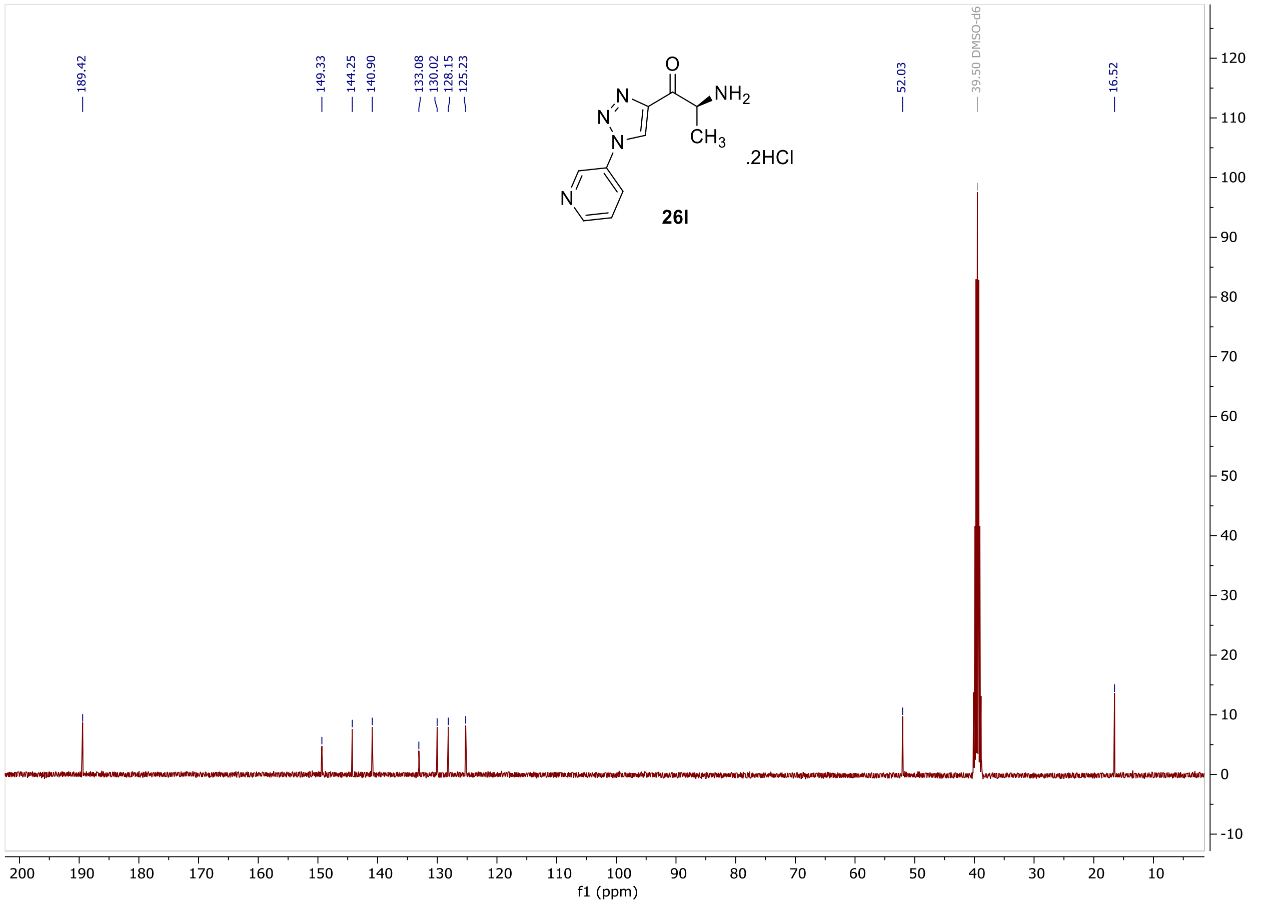

Compound **26m** (^{1}H NMR, 400 MHz):

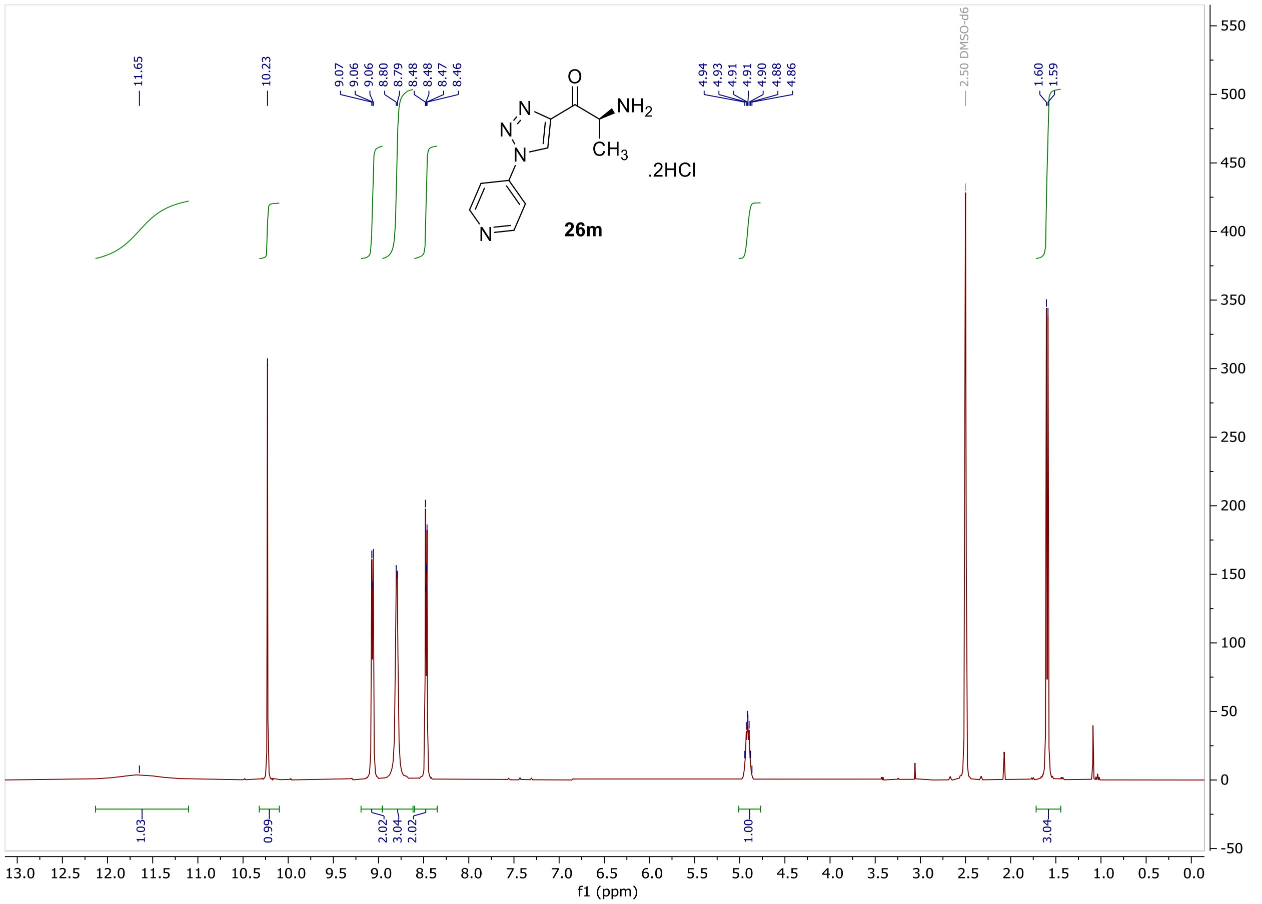

Compound **26m** (^{13}C NMR, 101 MHz):

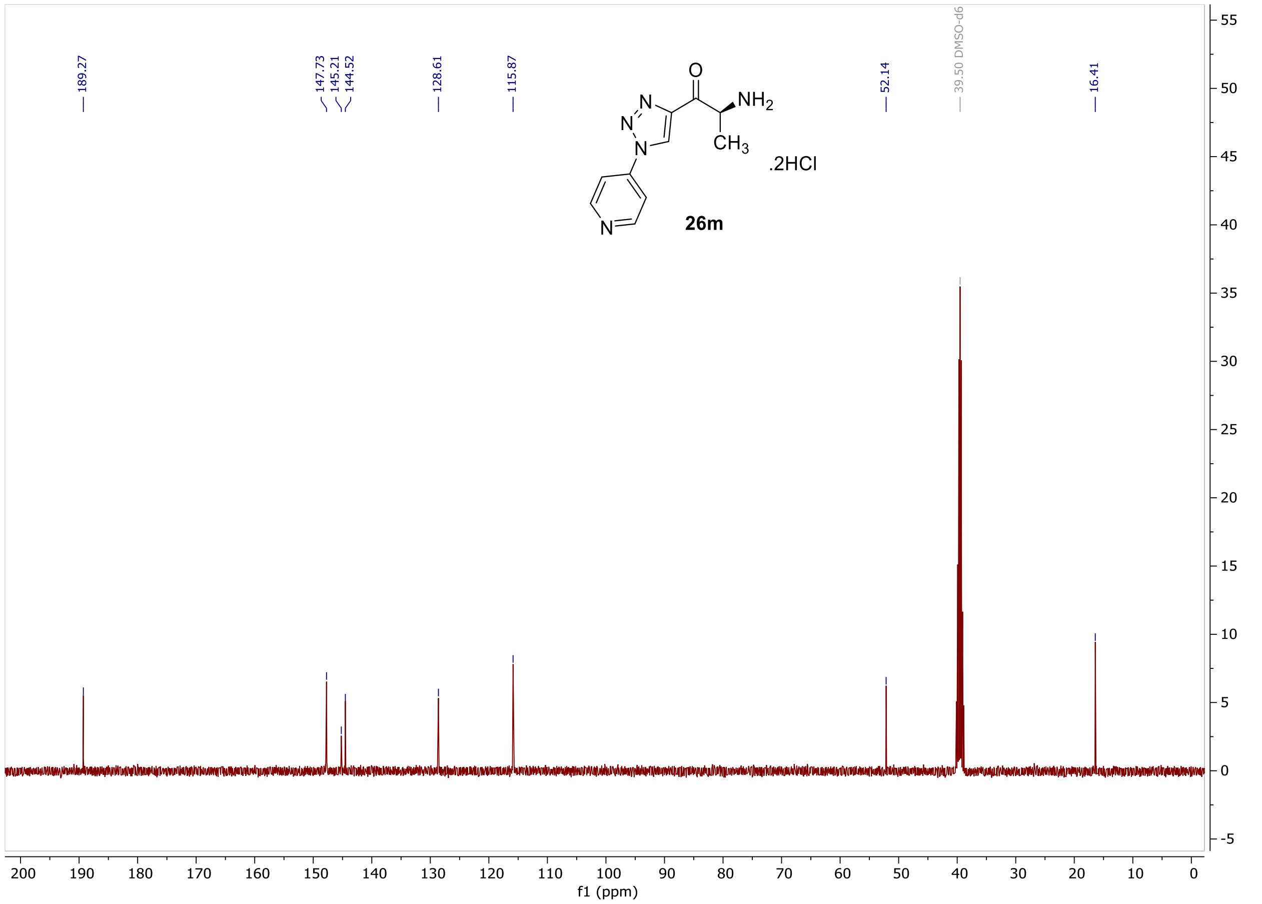

Compound **26n** (^{1}H NMR, 400 MHz):

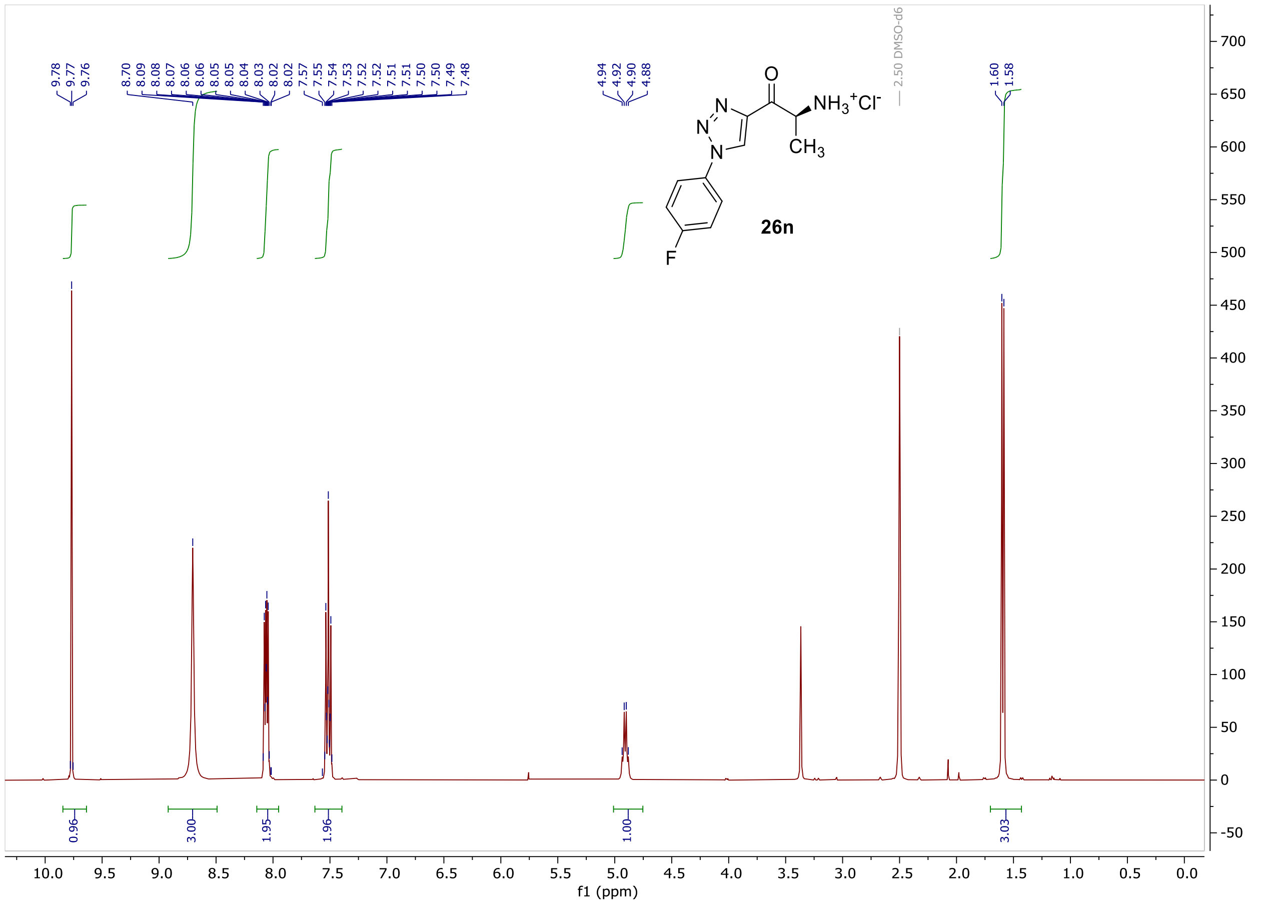

Compound **26n** (^{13}C NMR, 101 MHz):

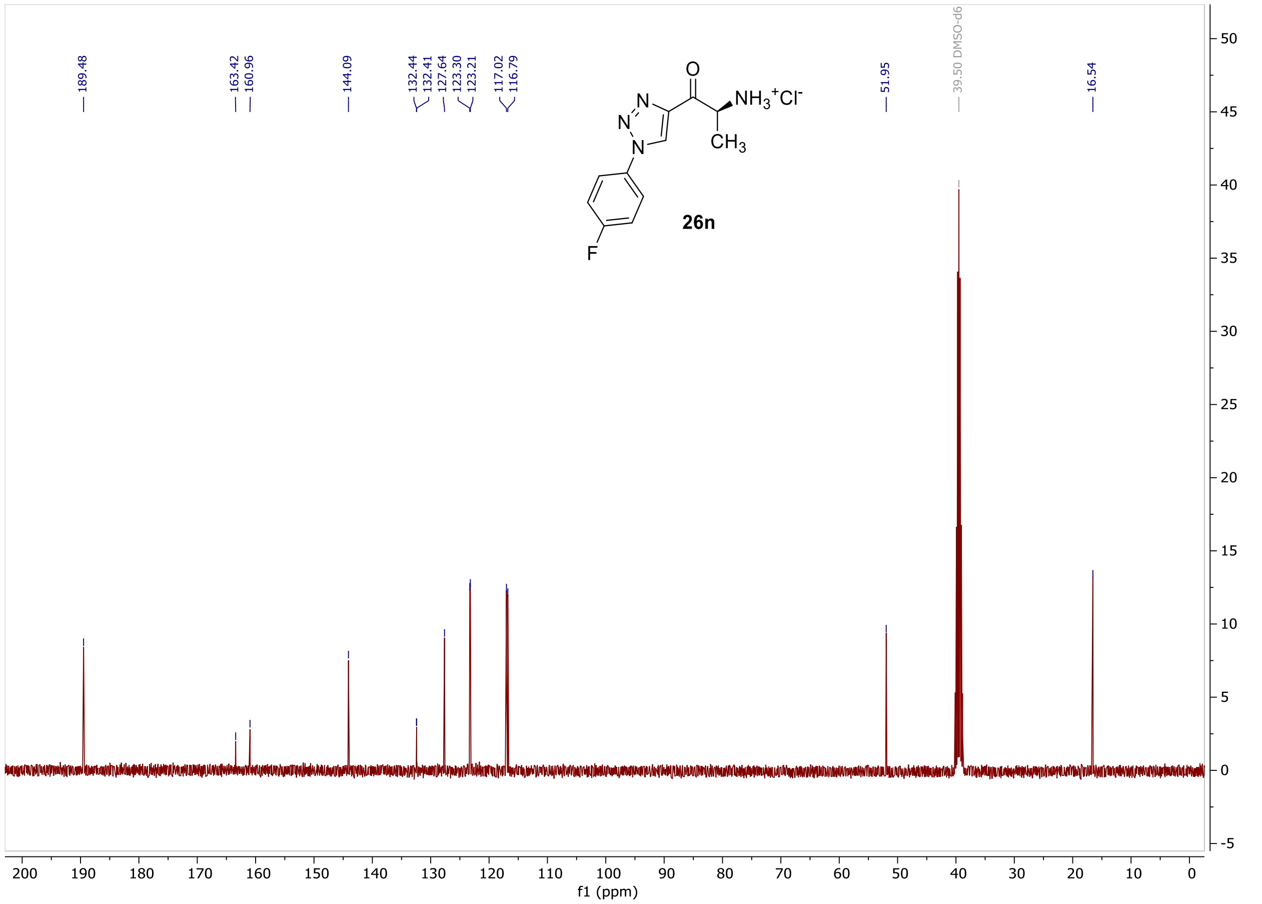

Compound **26n** (^{19}F NMR, 282 MHz):

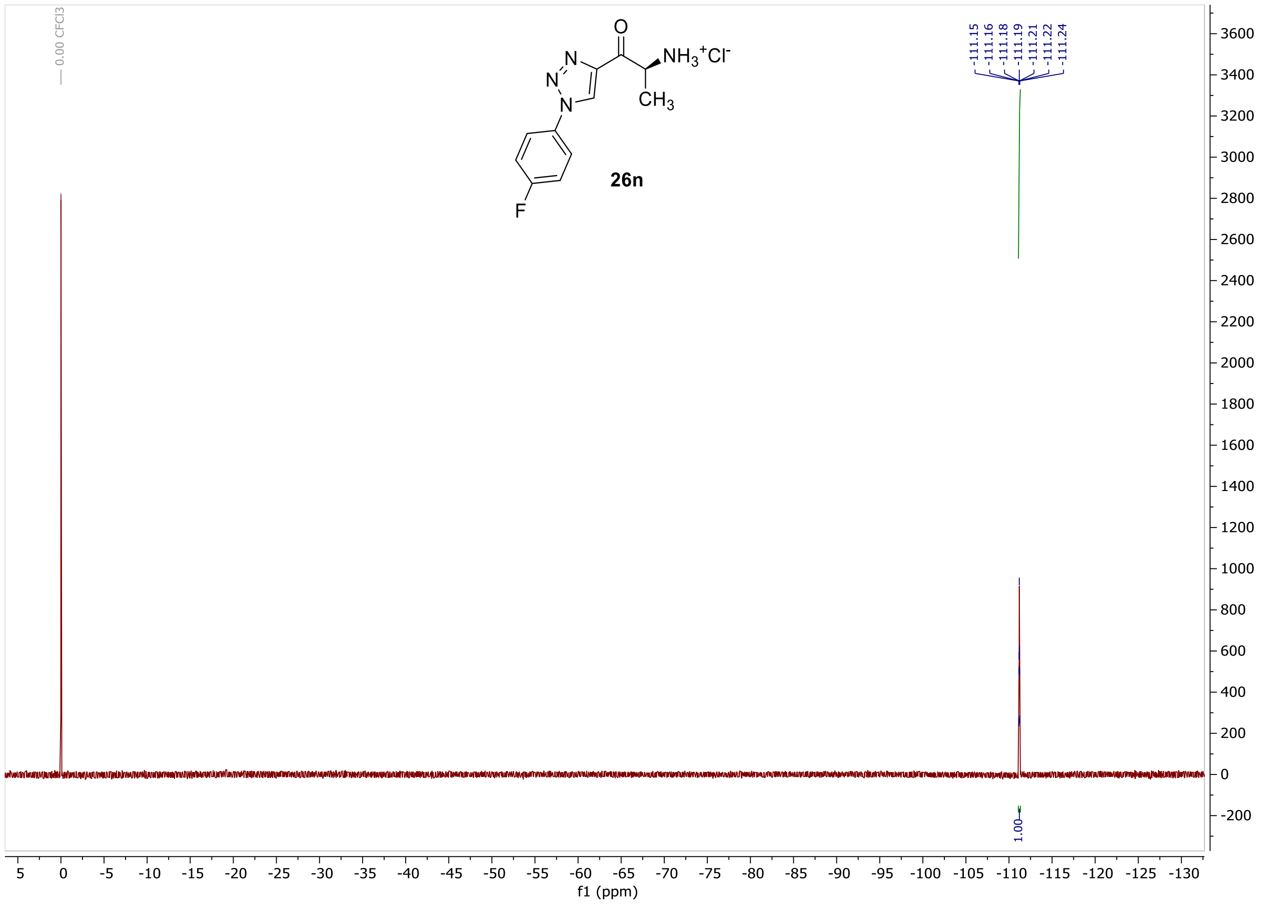

Compound **26o** (^{1}H NMR, 400 MHz):

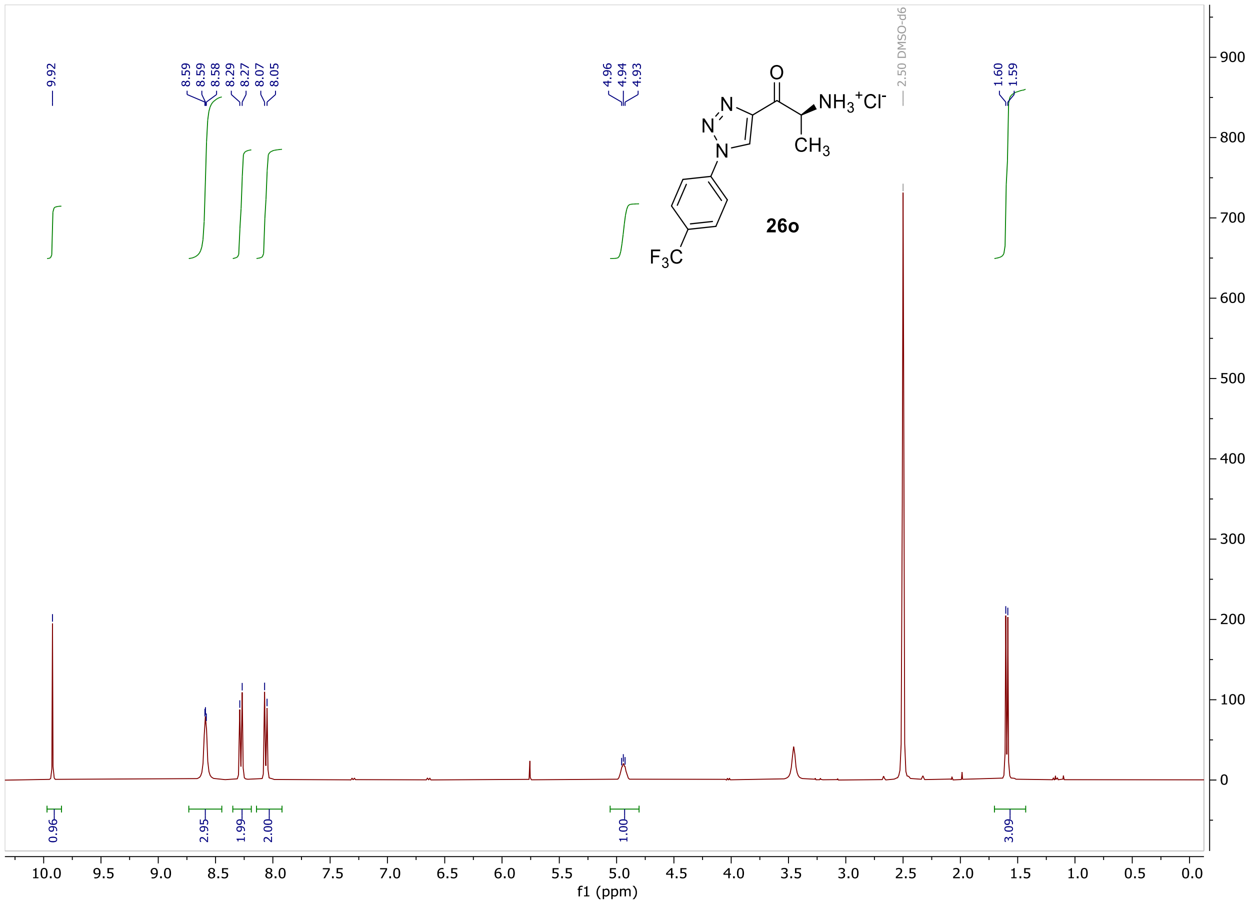

Compound **26o** (^{13}C NMR, 101 MHz):

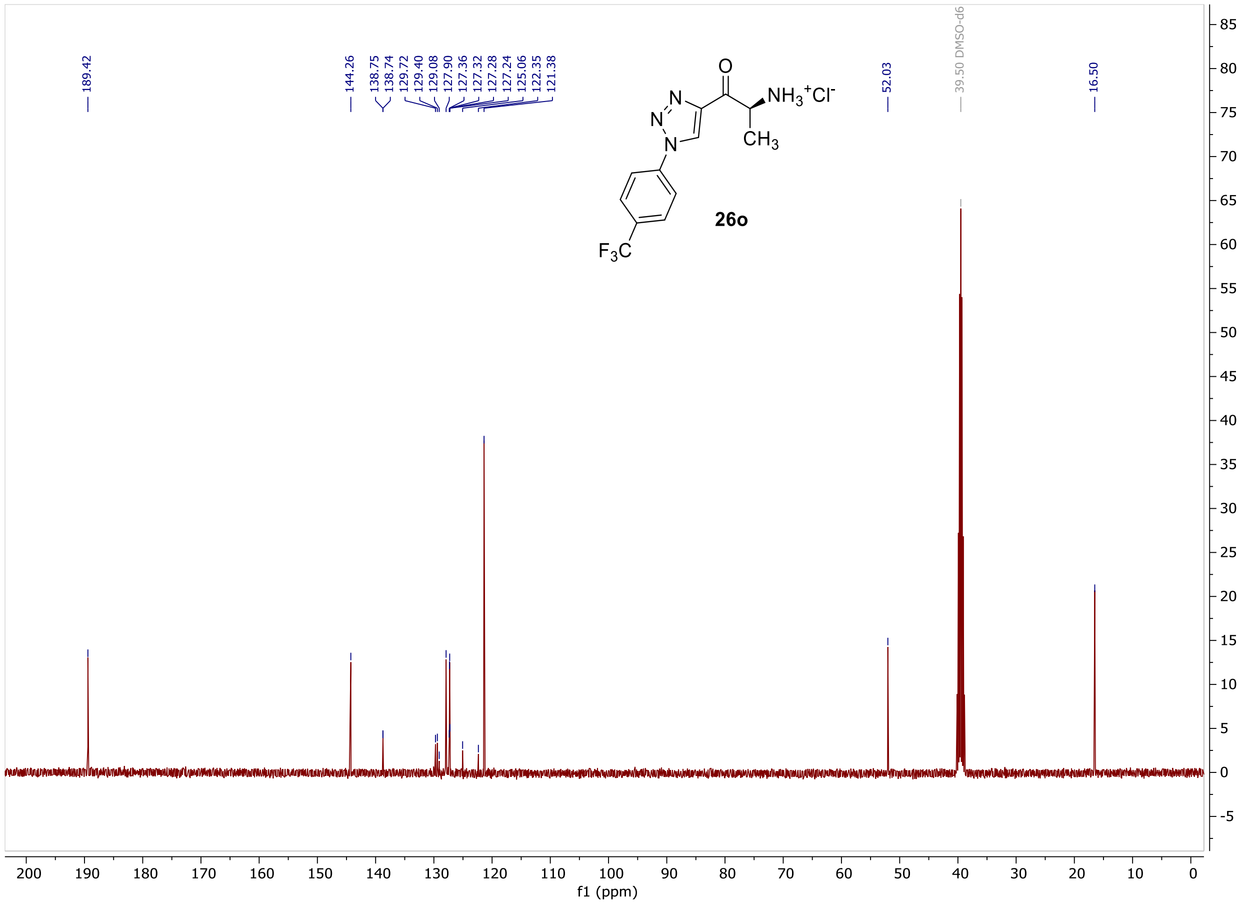

Compound **26o** (^{19}F NMR, 282 MHz):

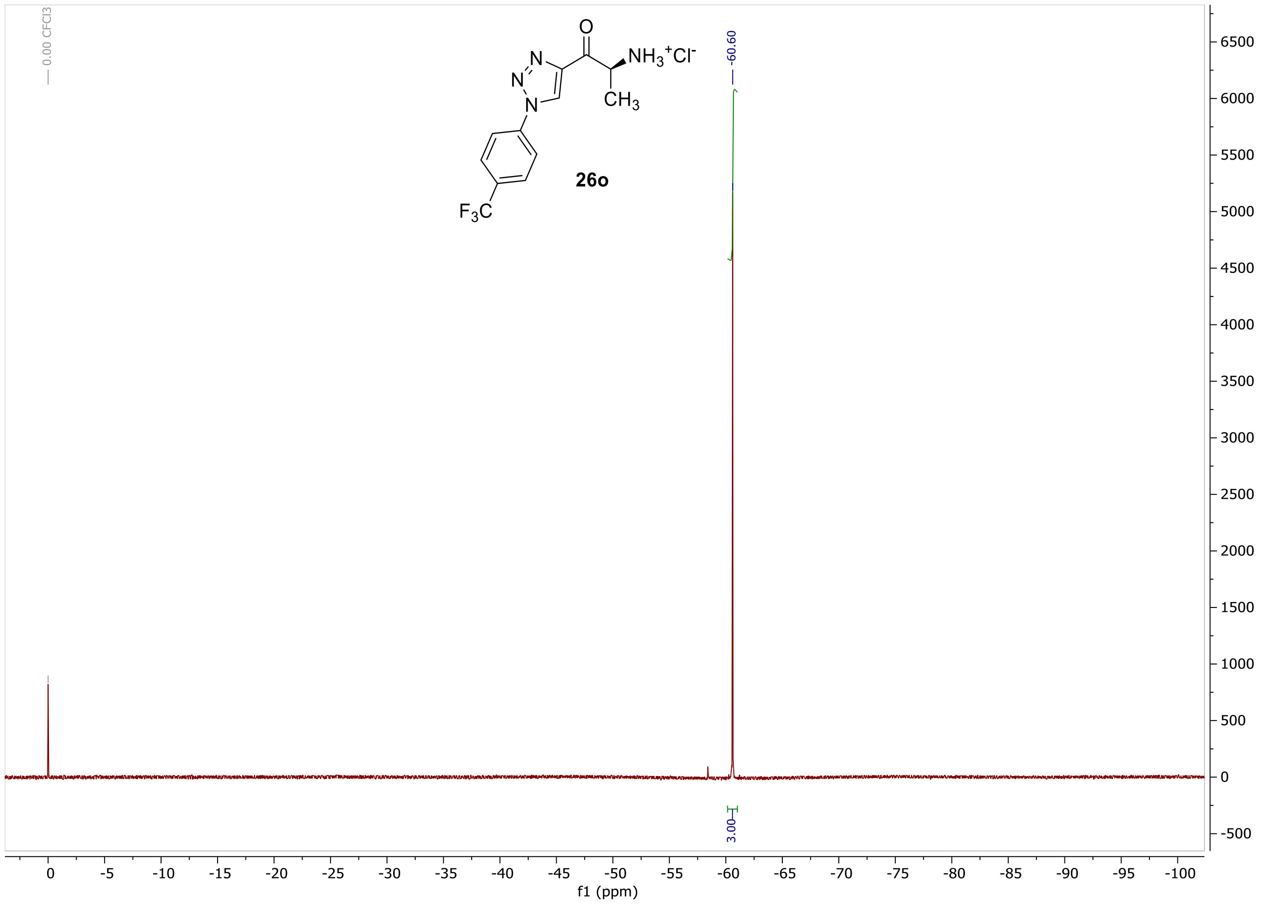

Compound **26p** (^{1}H NMR, 400 MHz):

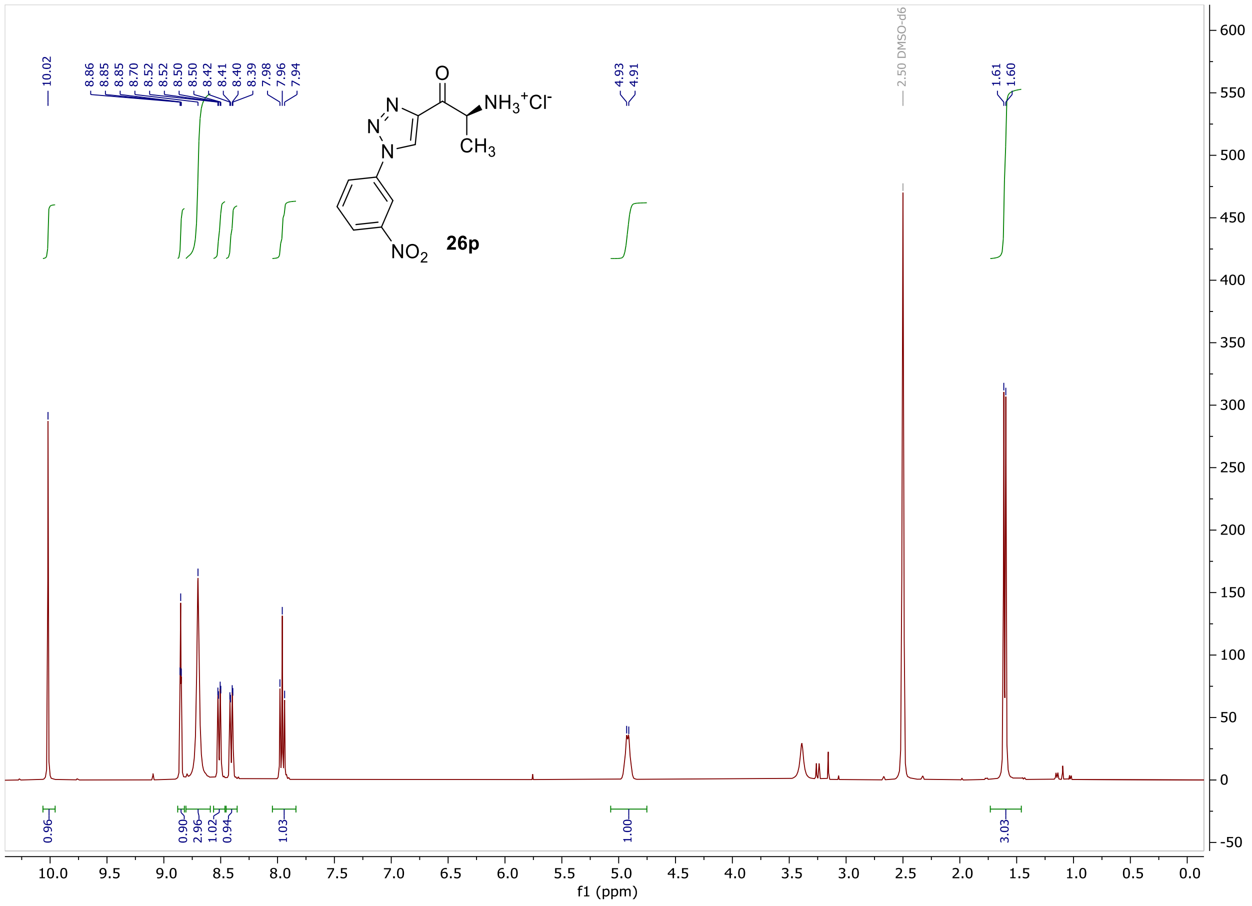

Compound **26p** (^{13}C NMR, 101 MHz):

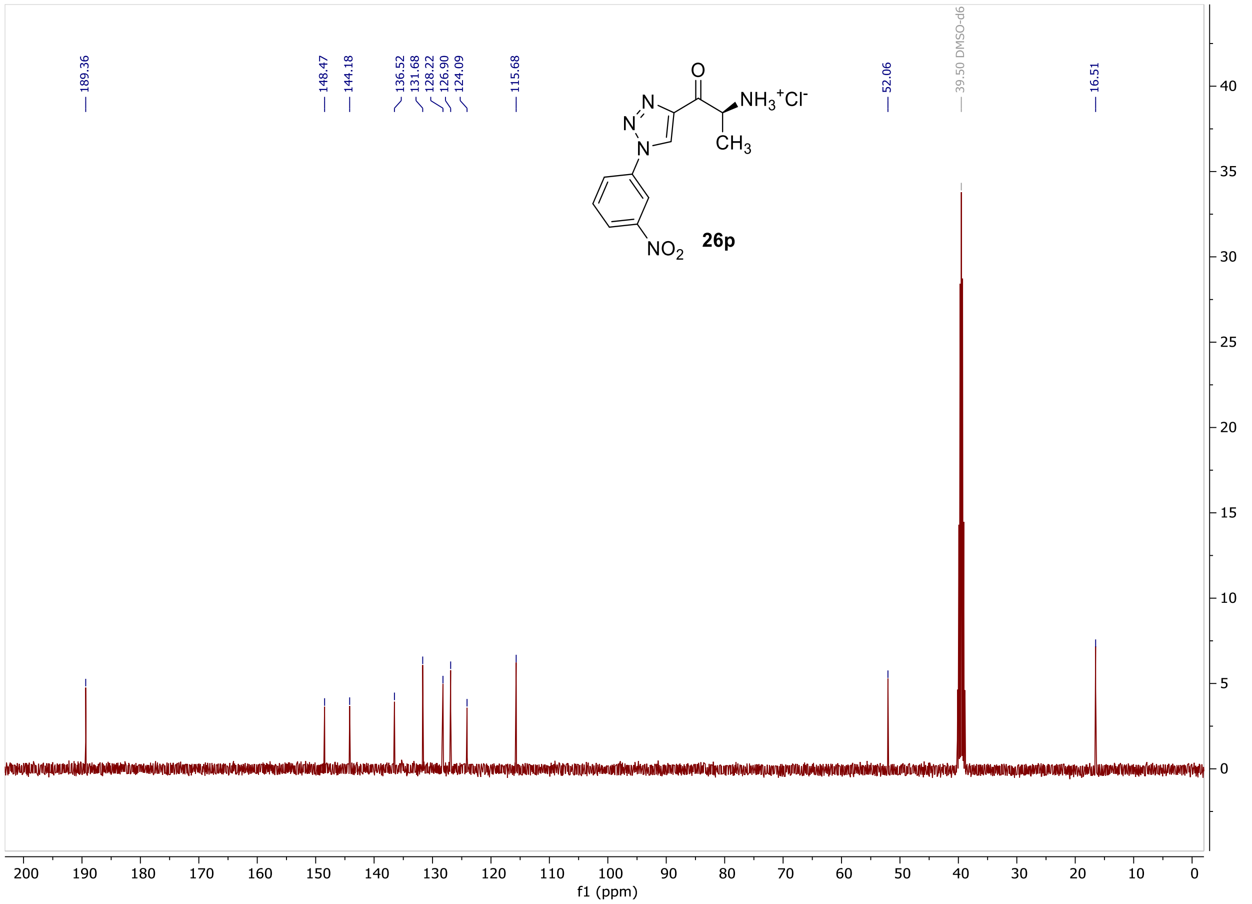

Compound **26q** (^{1}H NMR, 400 MHz):

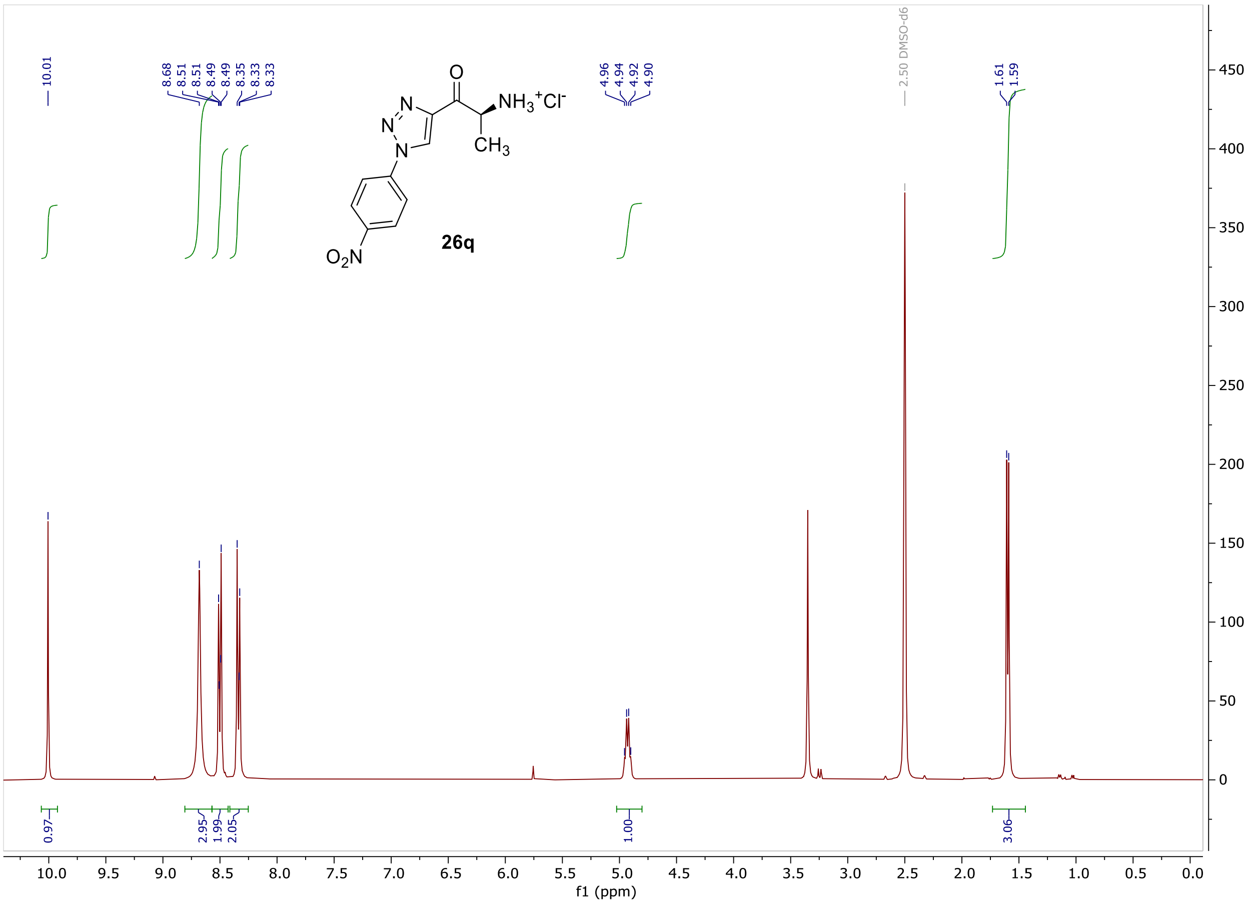

Compound **26q** (^{13}C NMR, 101 MHz):

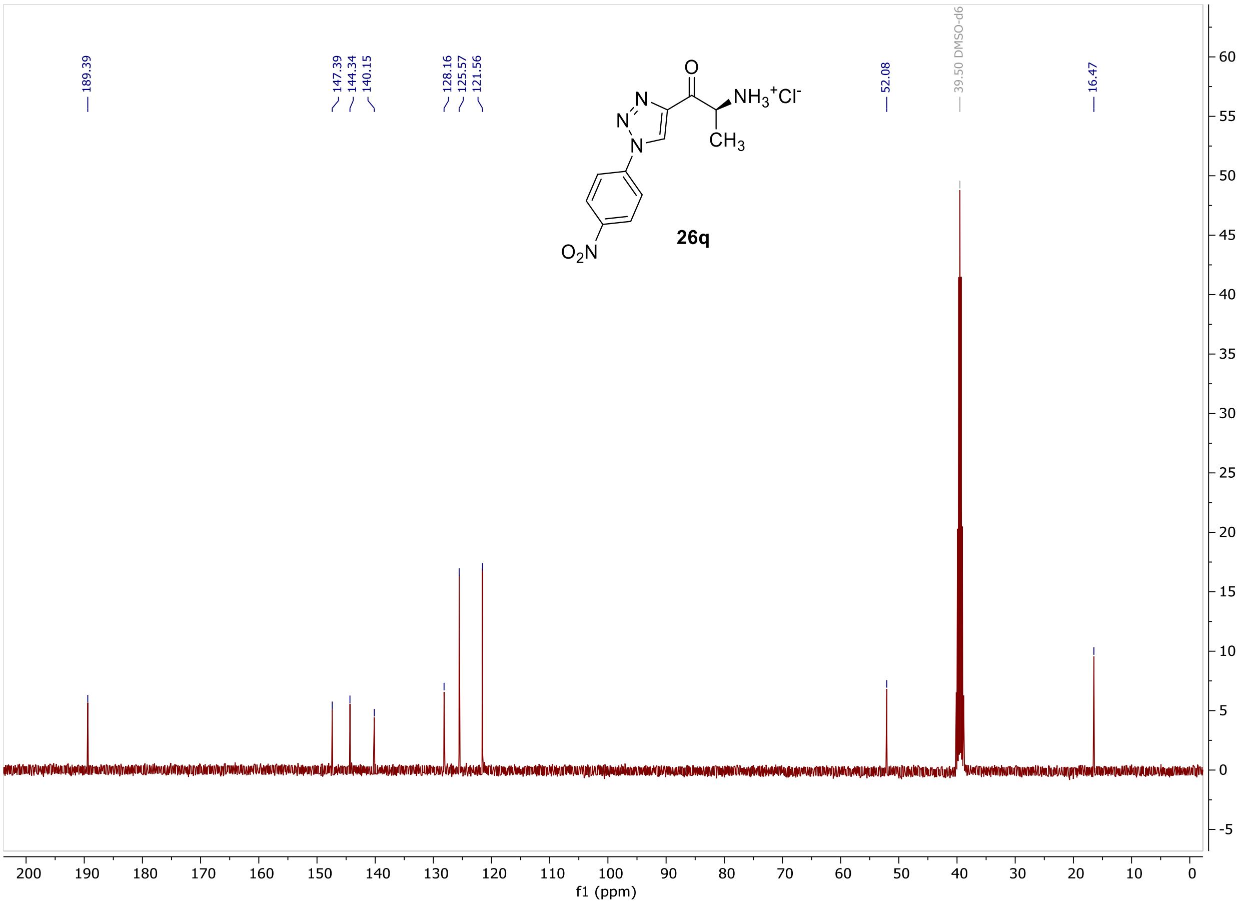

Compound **26r** (^{1}H NMR, 400 MHz):

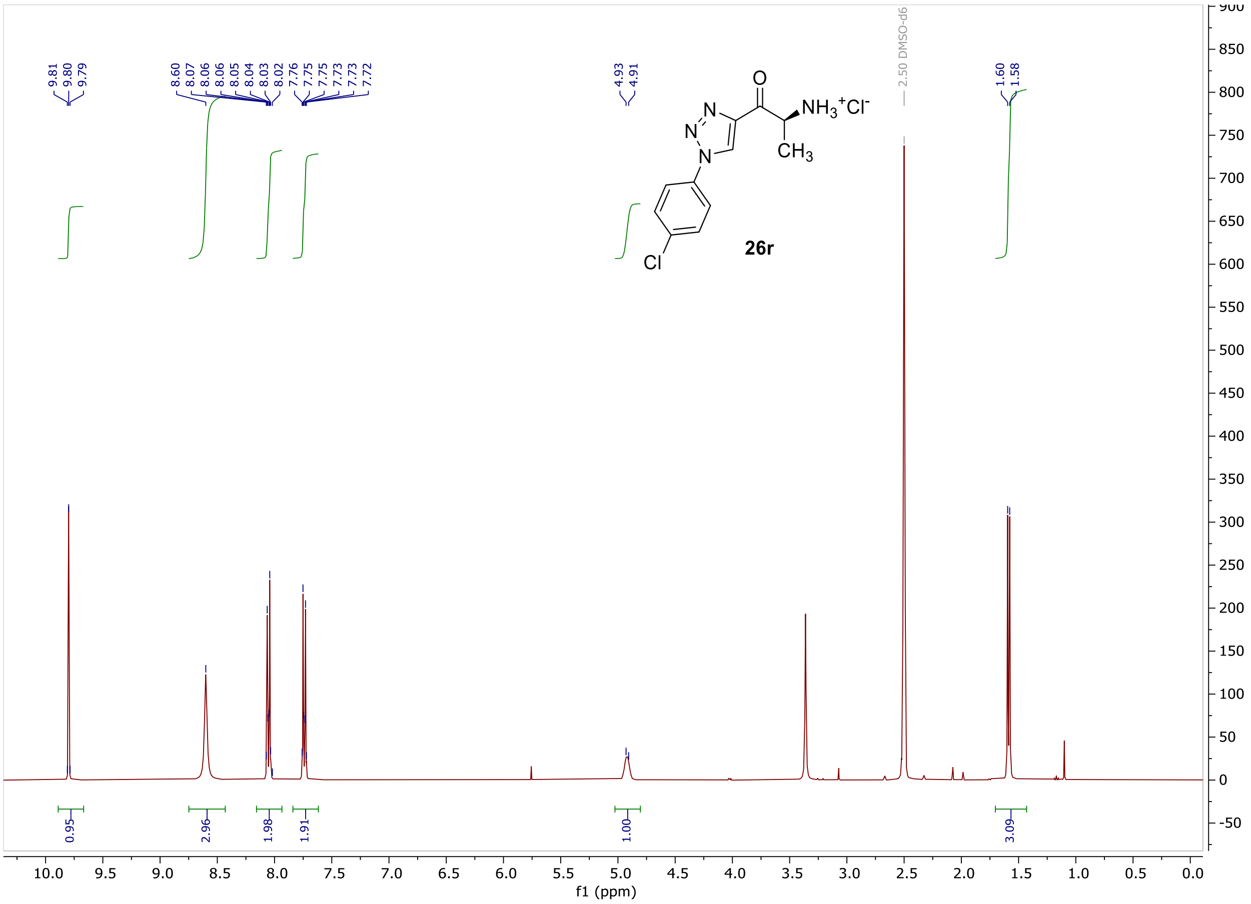

Compound **26r** (^{13}C NMR, 101 MHz):

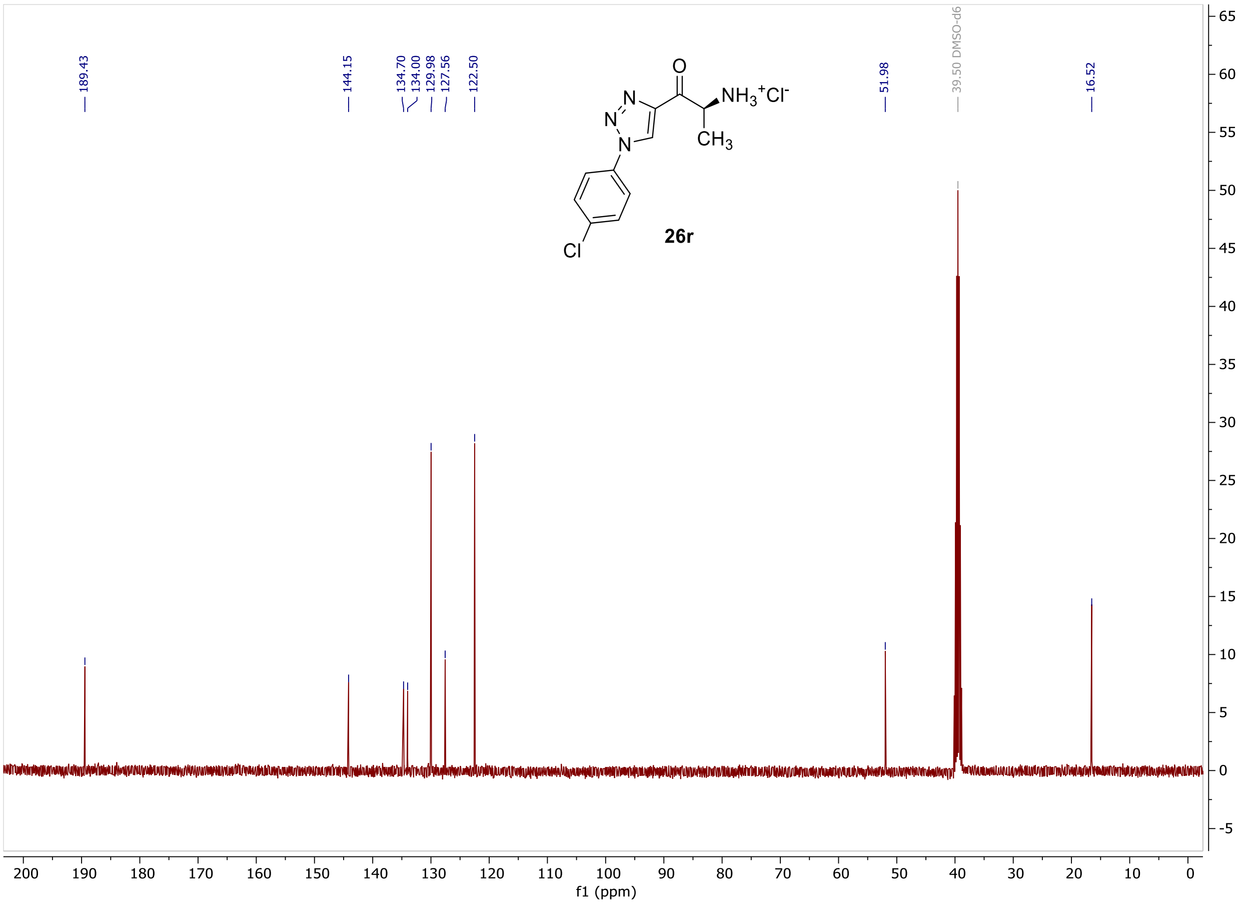

Compound **26s** (^{1}H NMR, 400 MHz):

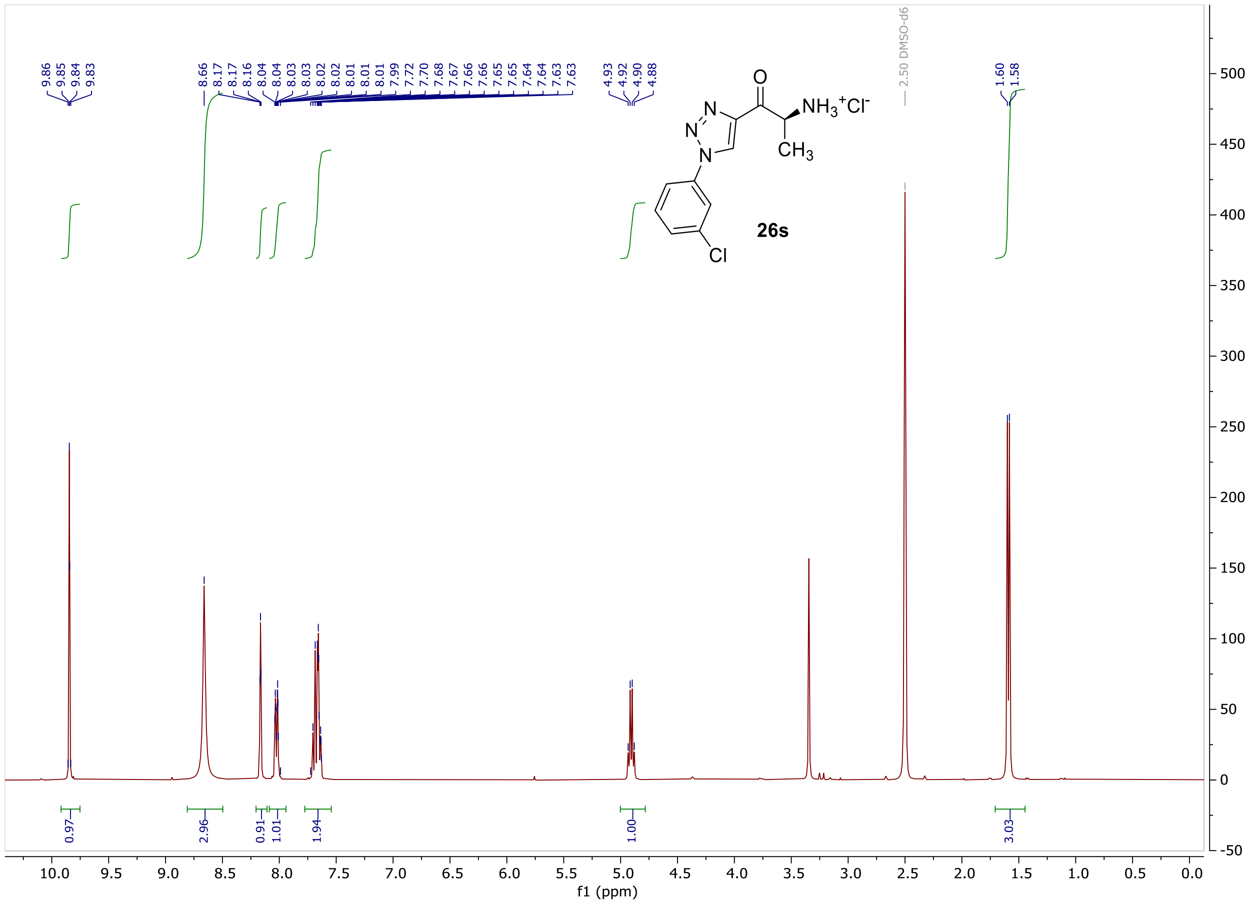

Compound **26s** (^{13}C NMR, 101 MHz):

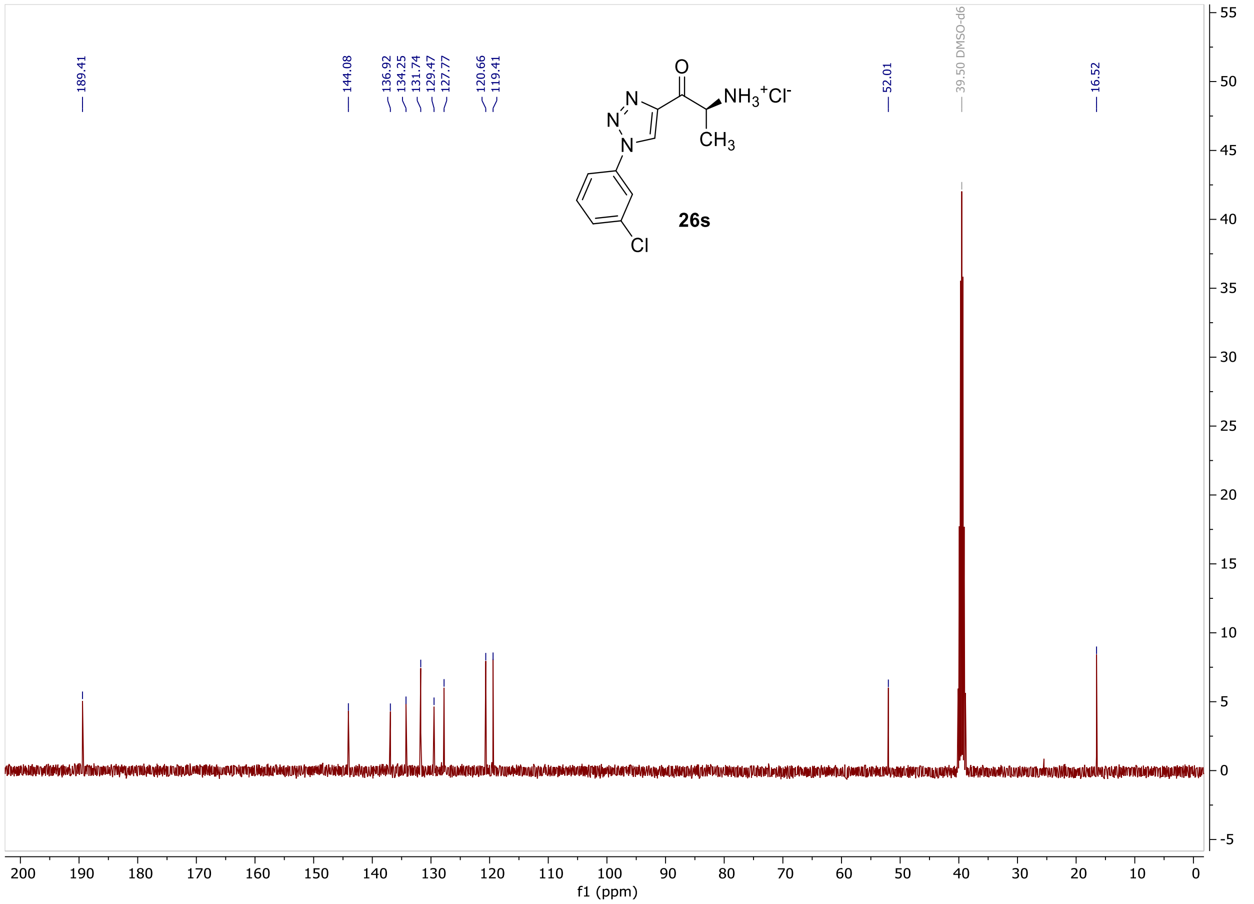

Compound **26t** (^{1}H NMR, 400 MHz):

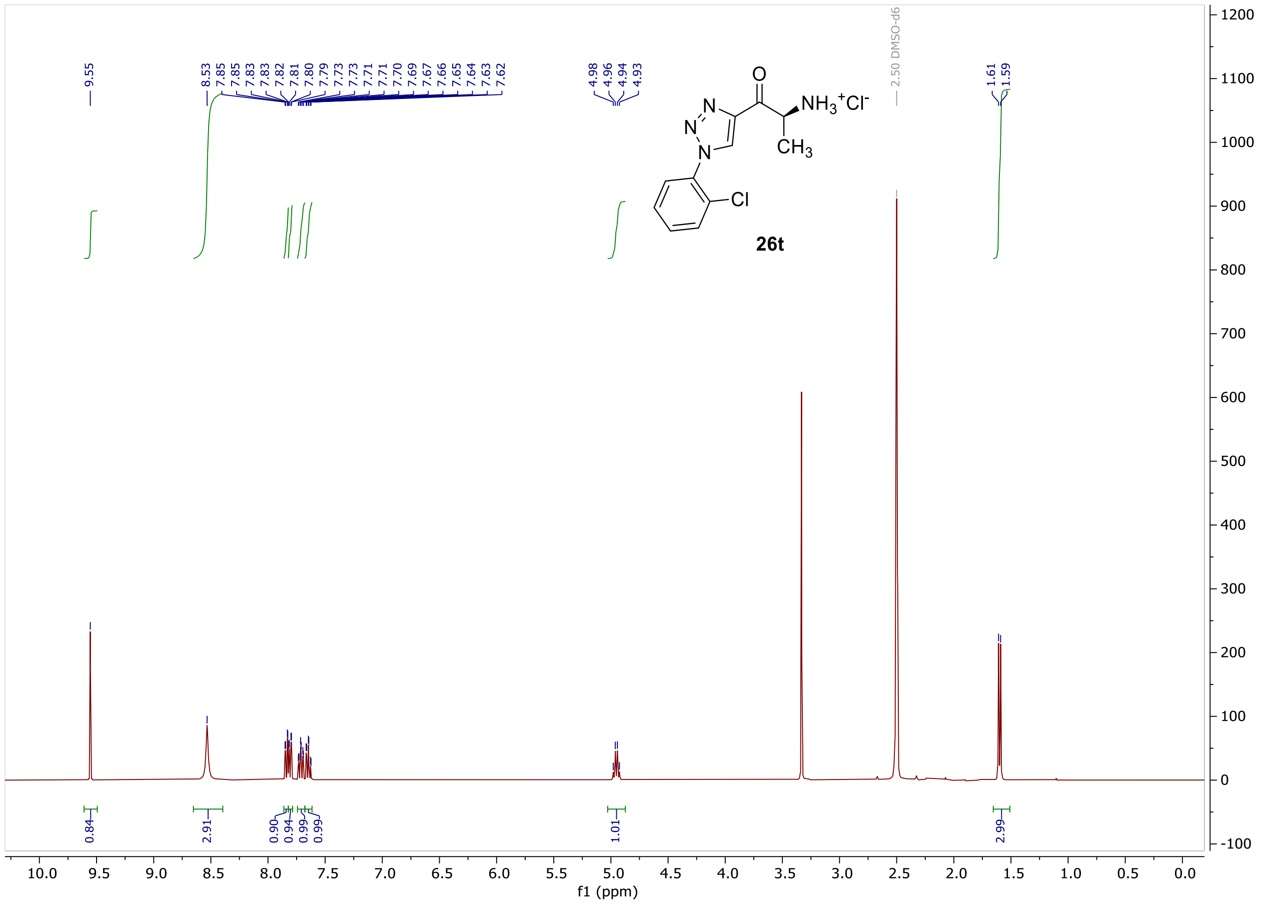

Compound **26t** (^{13}C NMR, 101 MHz):

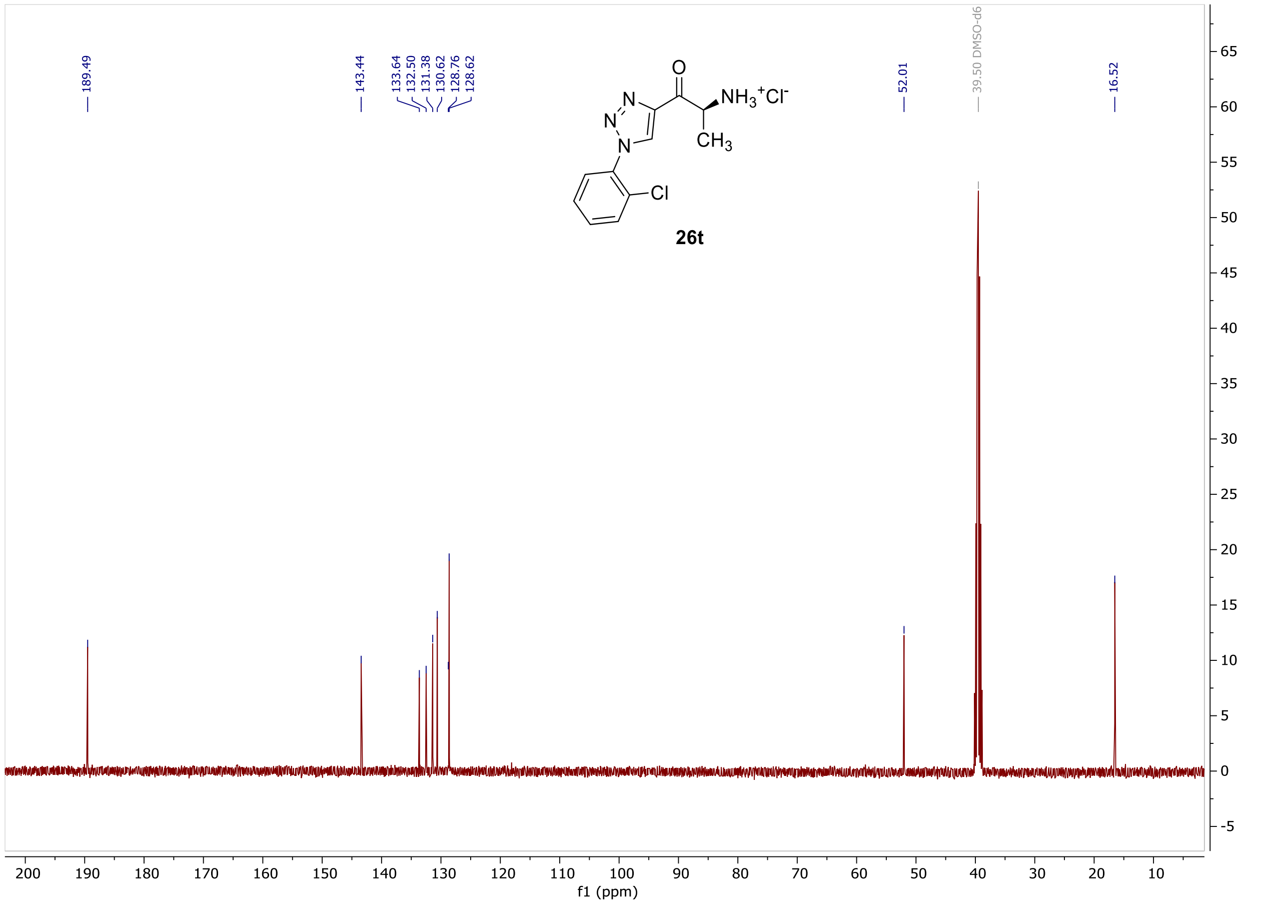

Compound **26u** (^{1}H NMR, 400 MHz):

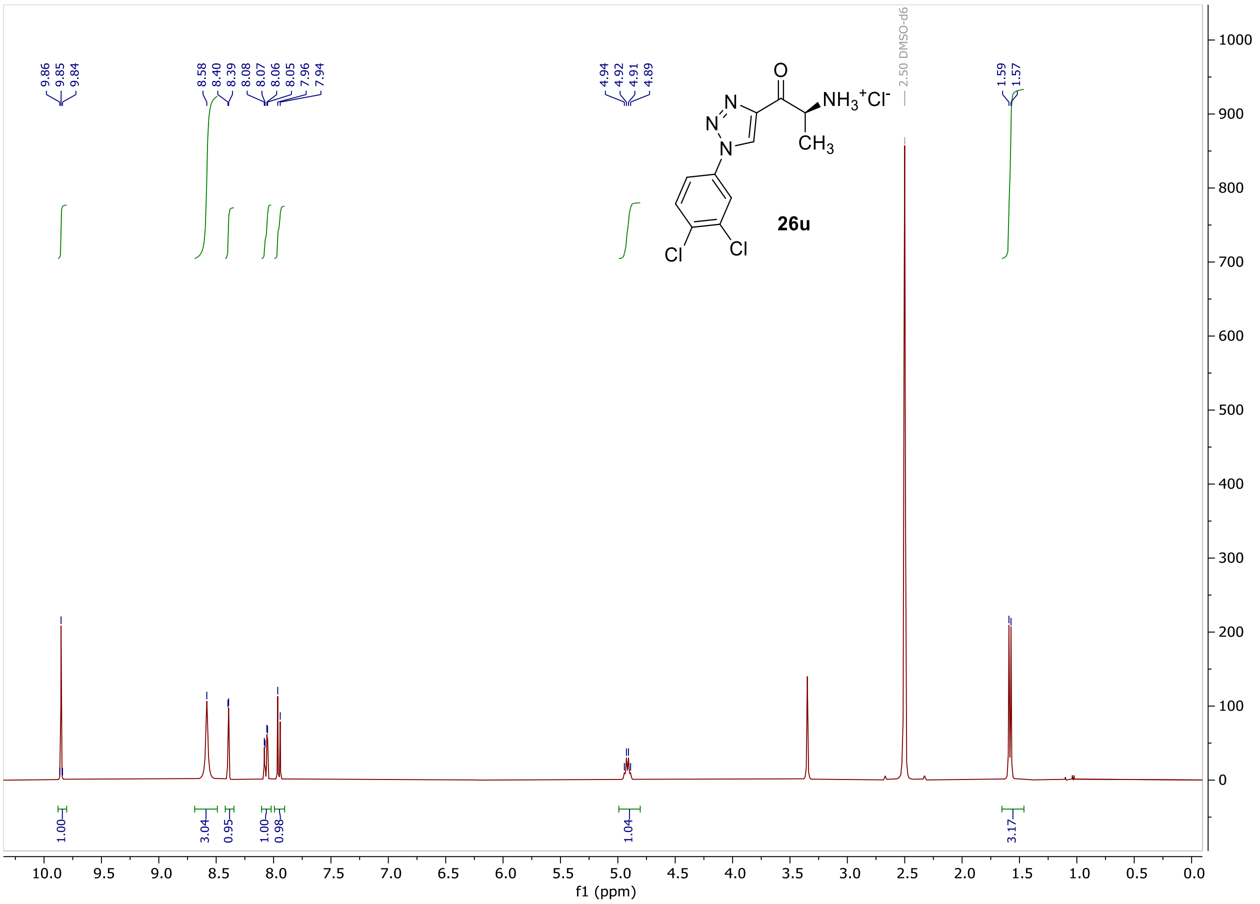

Compound **26u** (^{13}C NMR, 101 MHz):

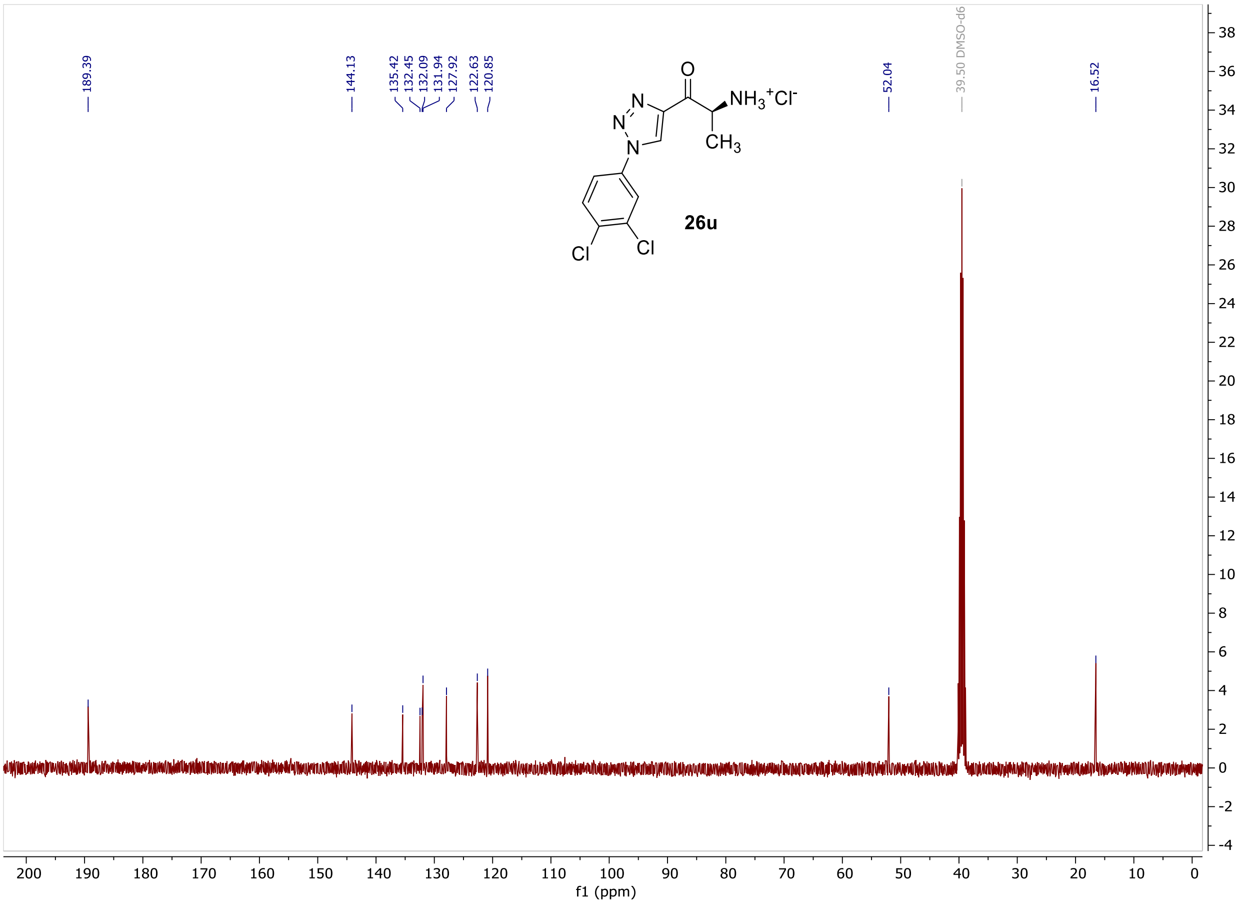

Compound **26v** (^{1}H NMR, 400 MHz):

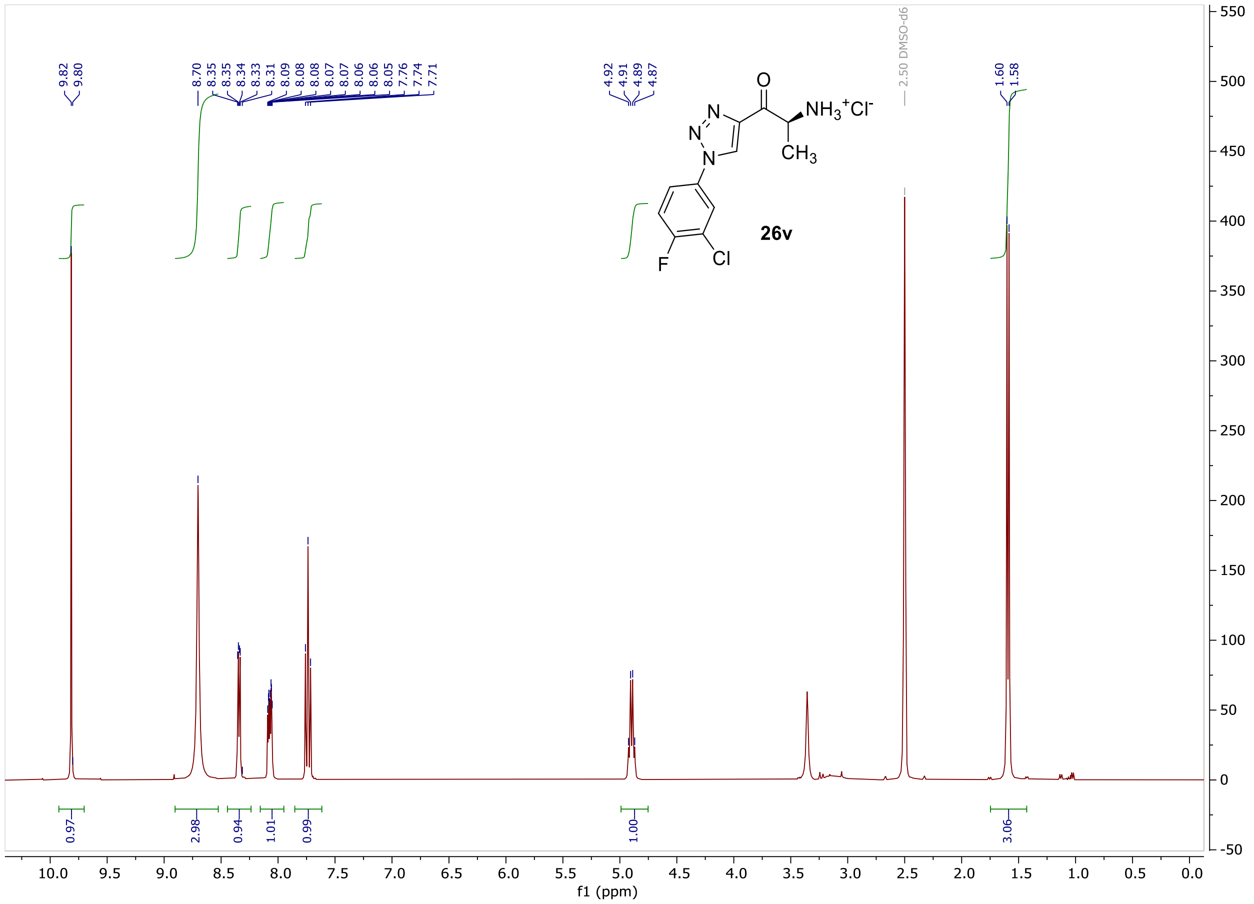

Compound **26v** (^{13}C NMR, 101 MHz):

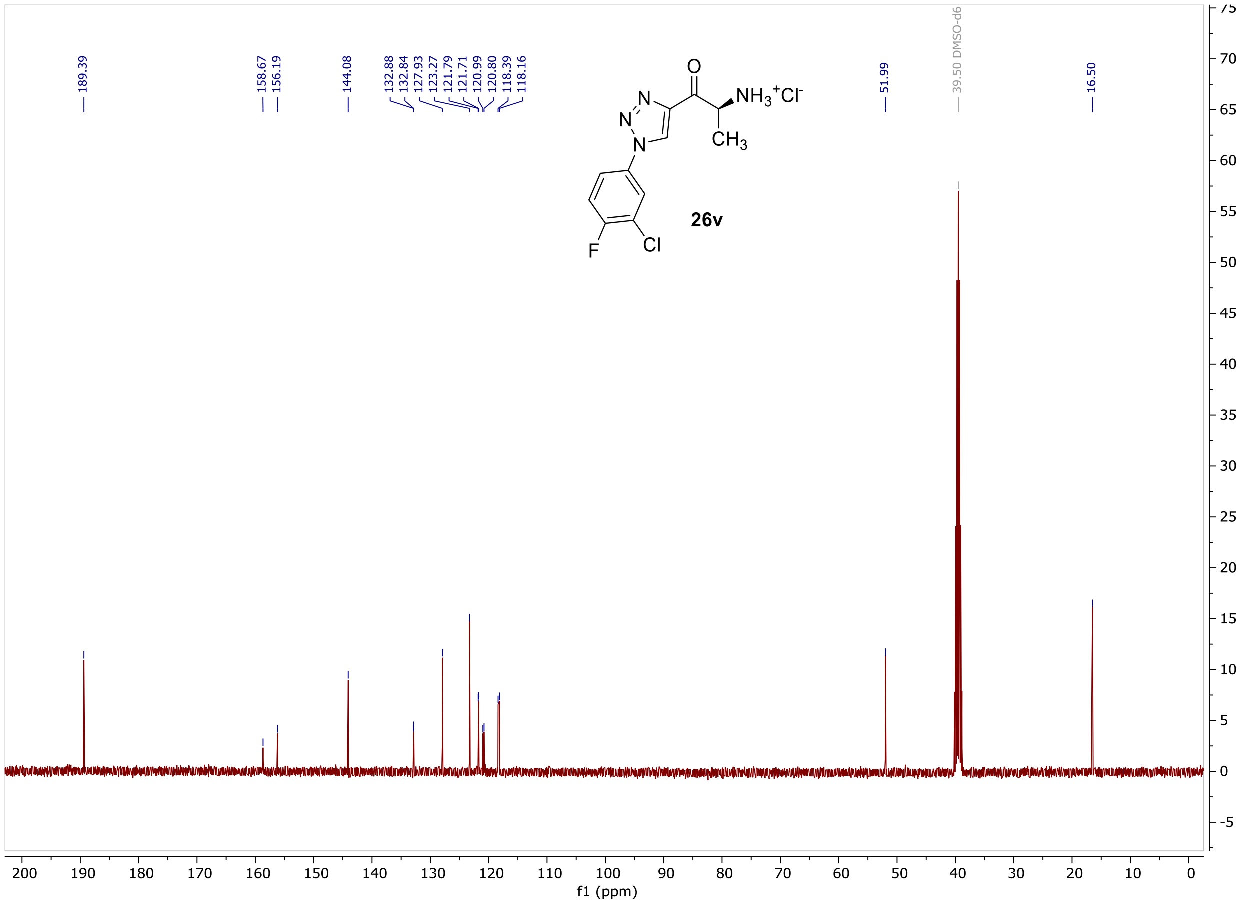

Compound **26v** (^{19}F NMR, 282 MHz):

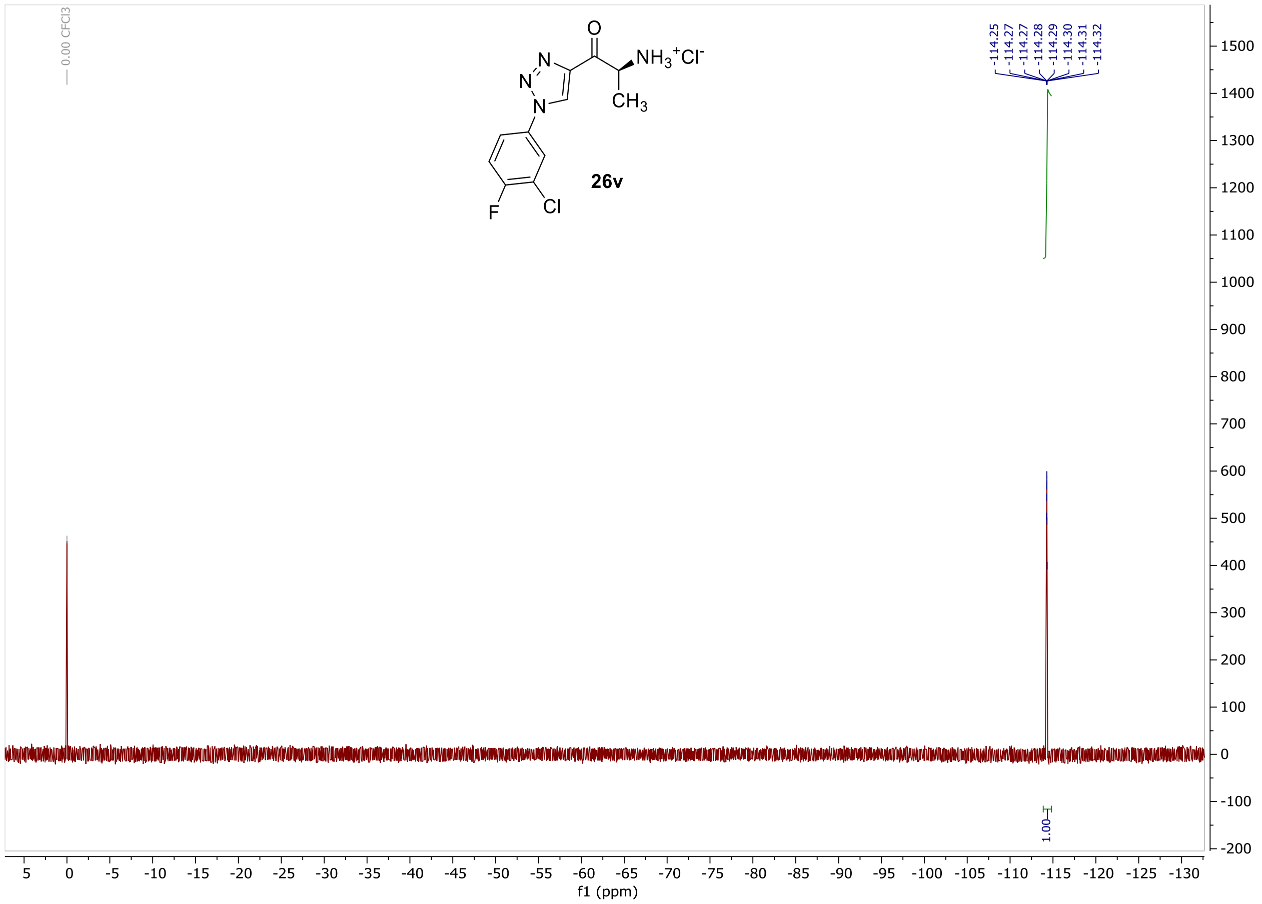

Compound **32a** (^{1}H NMR, 400 MHz):

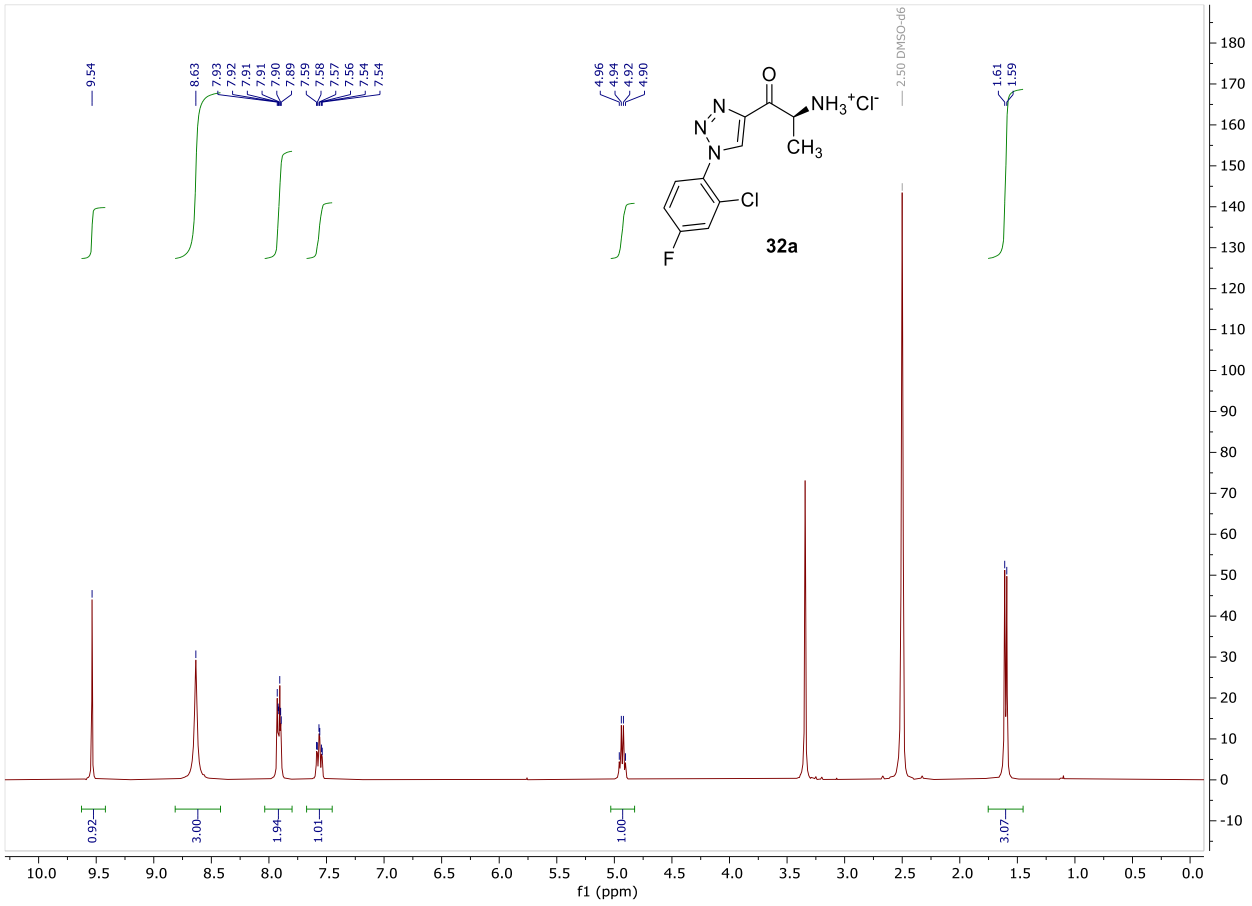

Compound **32a** (^{13}C NMR, 101 MHz):

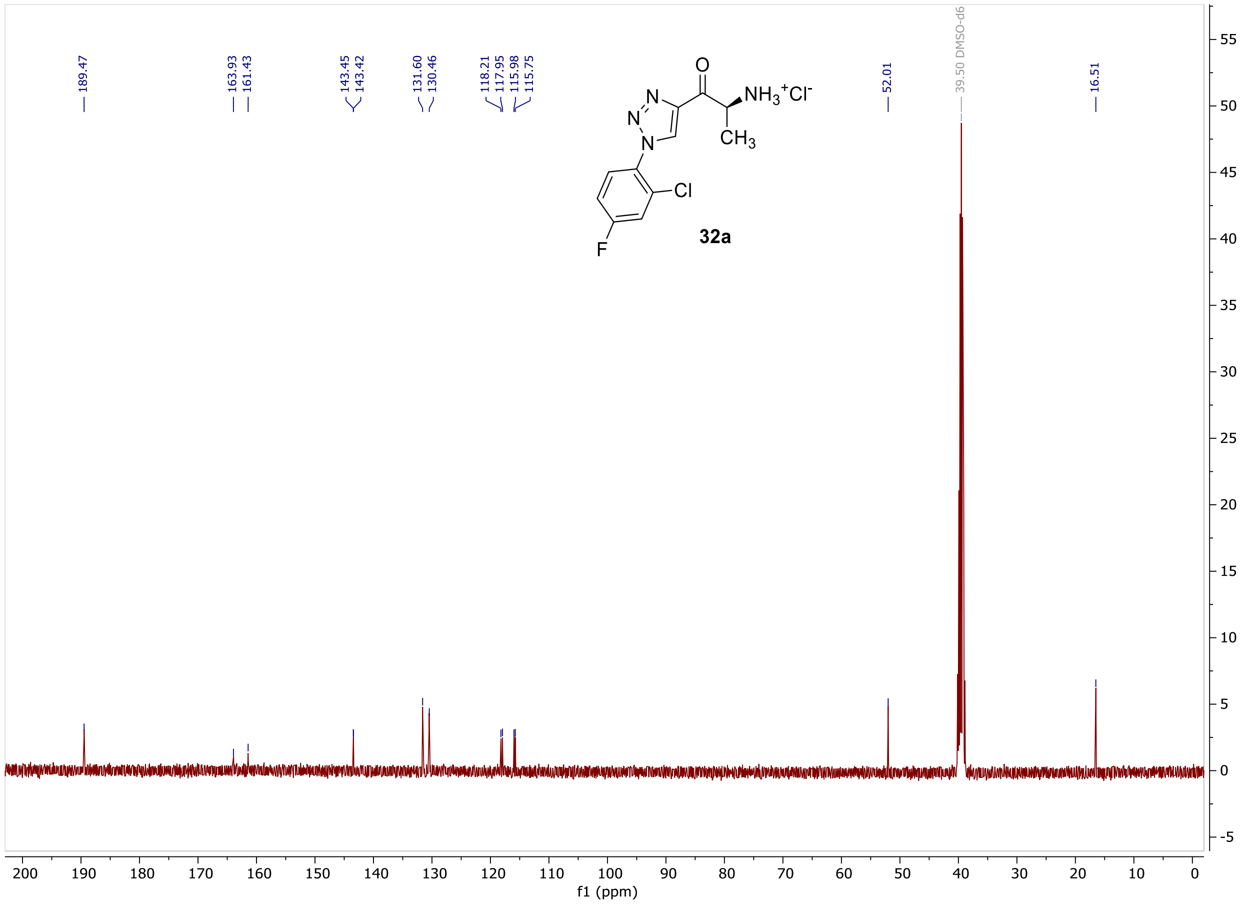

Compound **32a** (^{19}F NMR, 282 MHz):

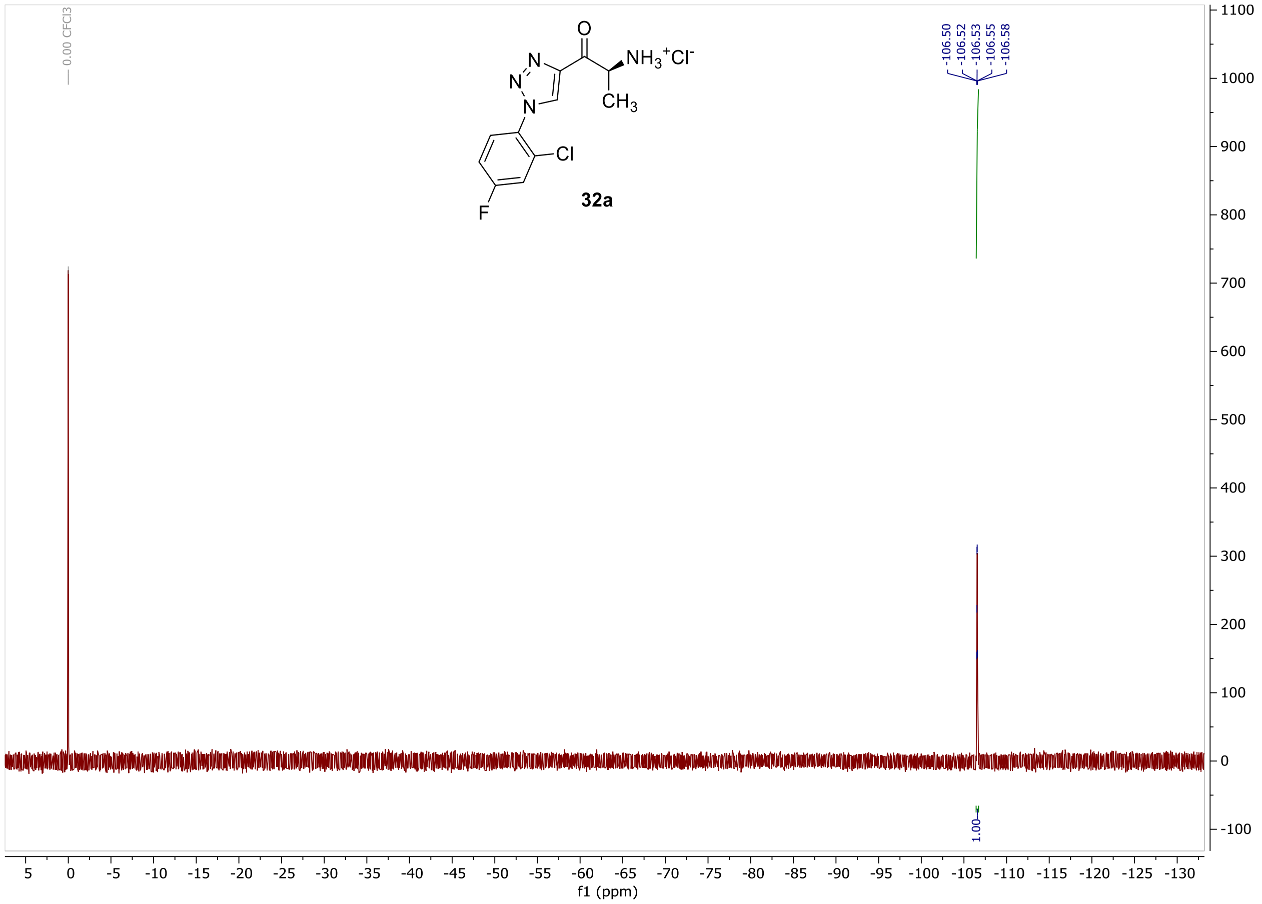

Compound **32b** (^{1}H NMR, 400 MHz):

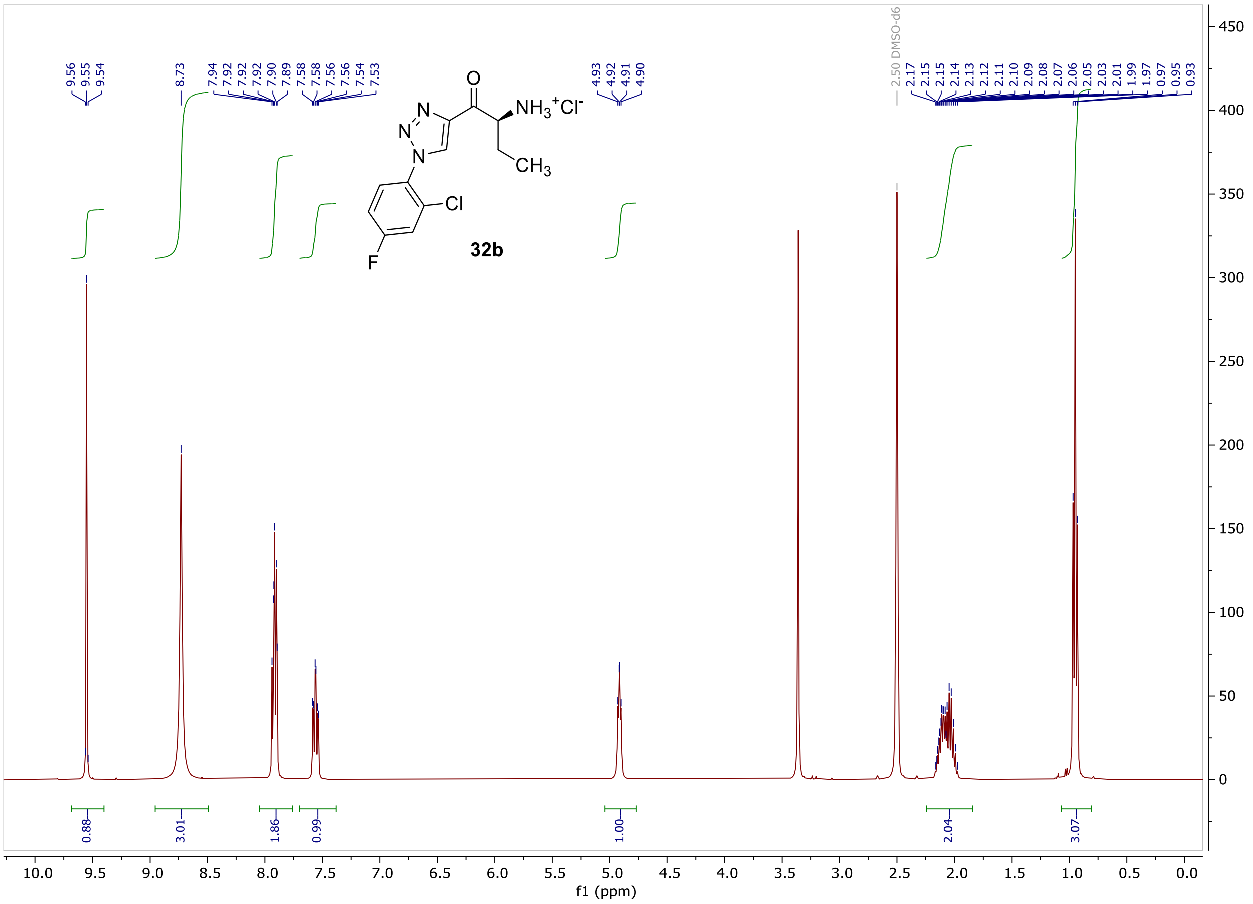

Compound **32b** (^{13}C NMR, 101 MHz):

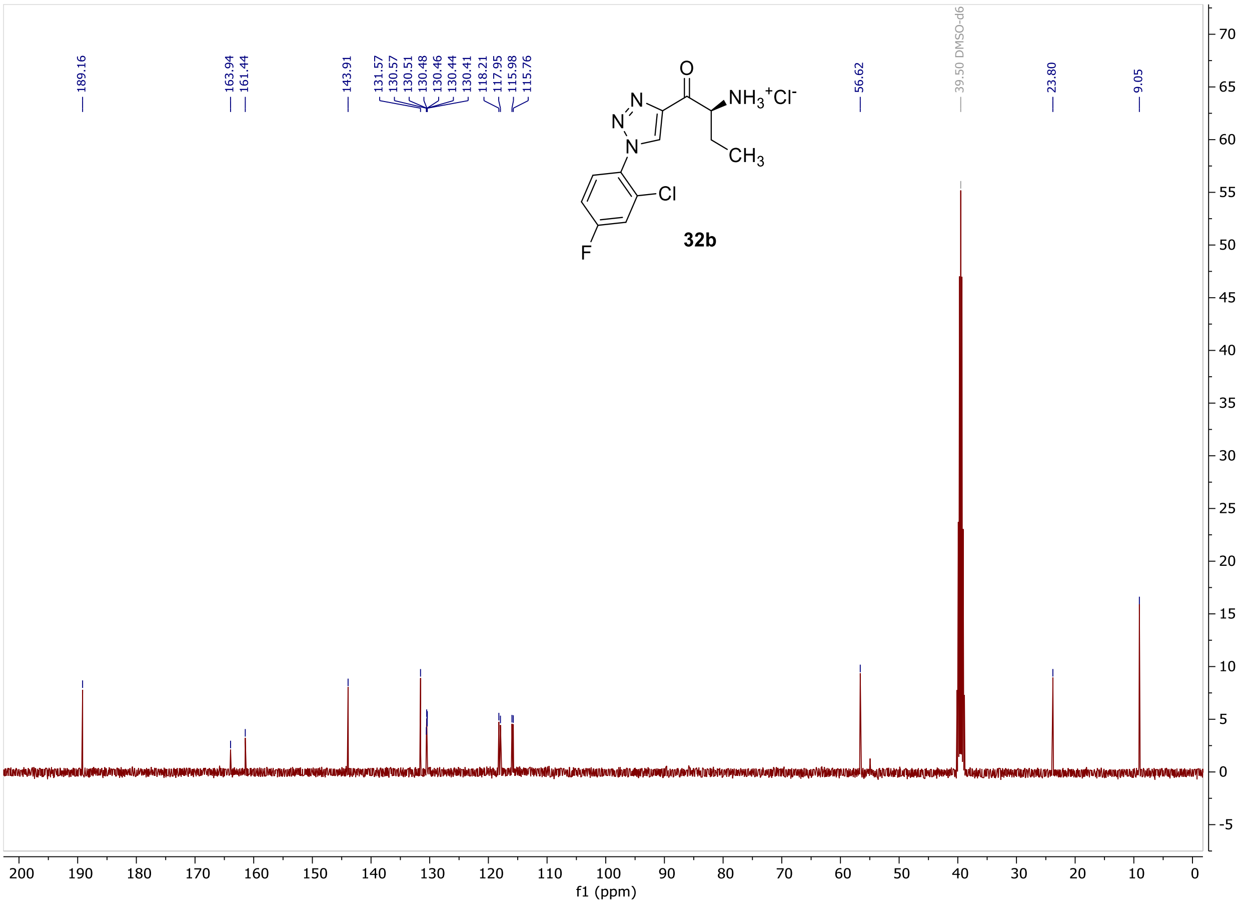

Compound **32b** (^{19}F NMR, 282 MHz):

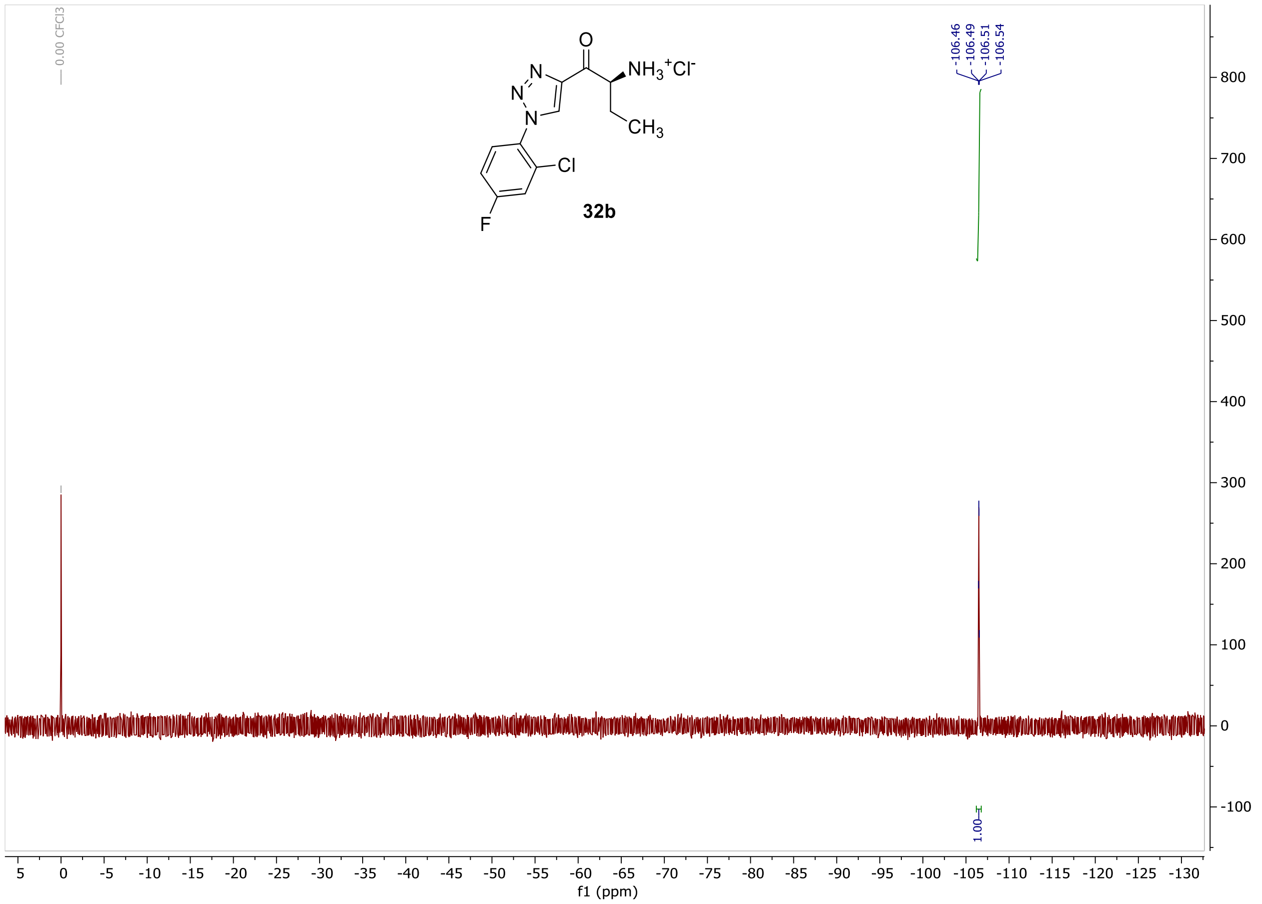

Compound **32c** (^{1}H NMR, 400 MHz):

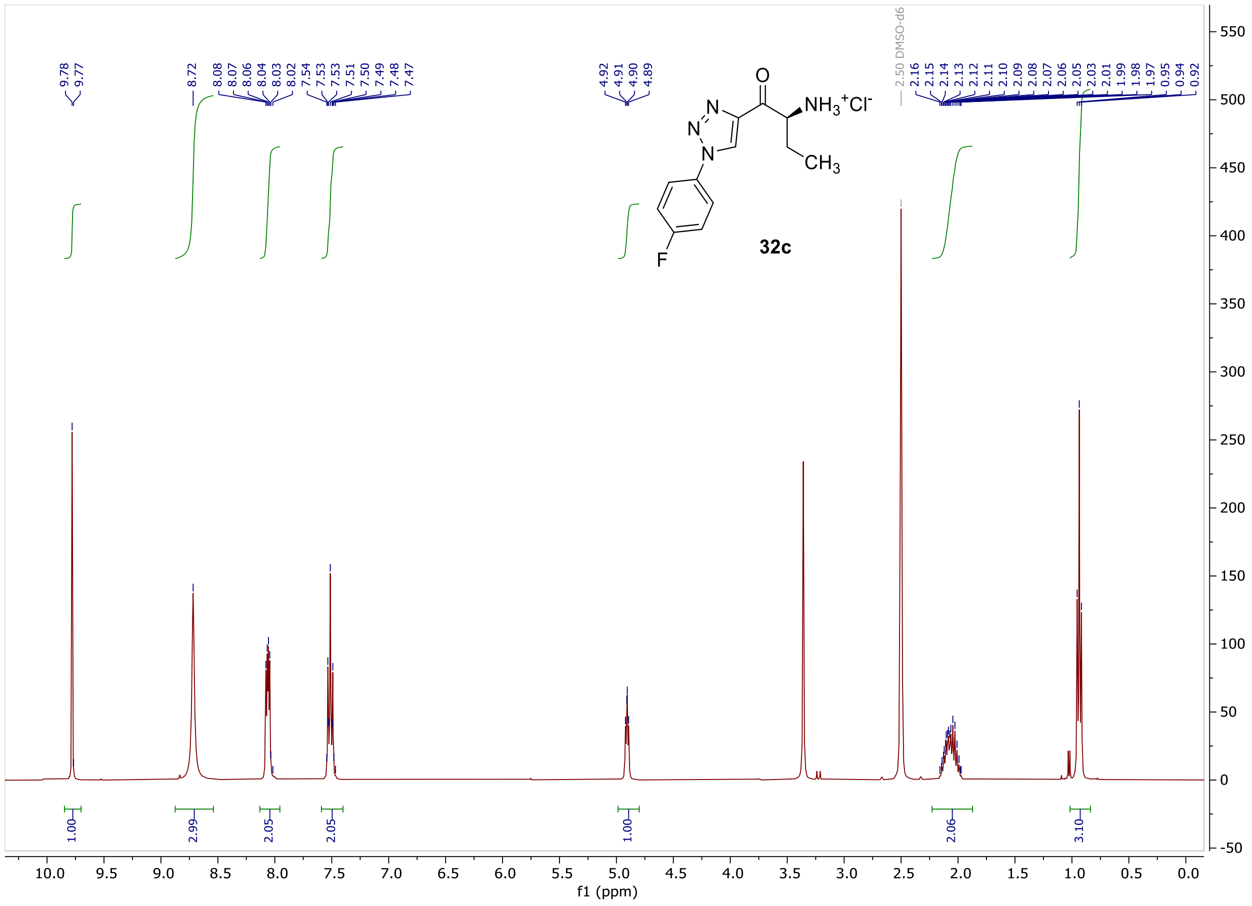

Compound **32c** (^{13}C NMR, 101 MHz):

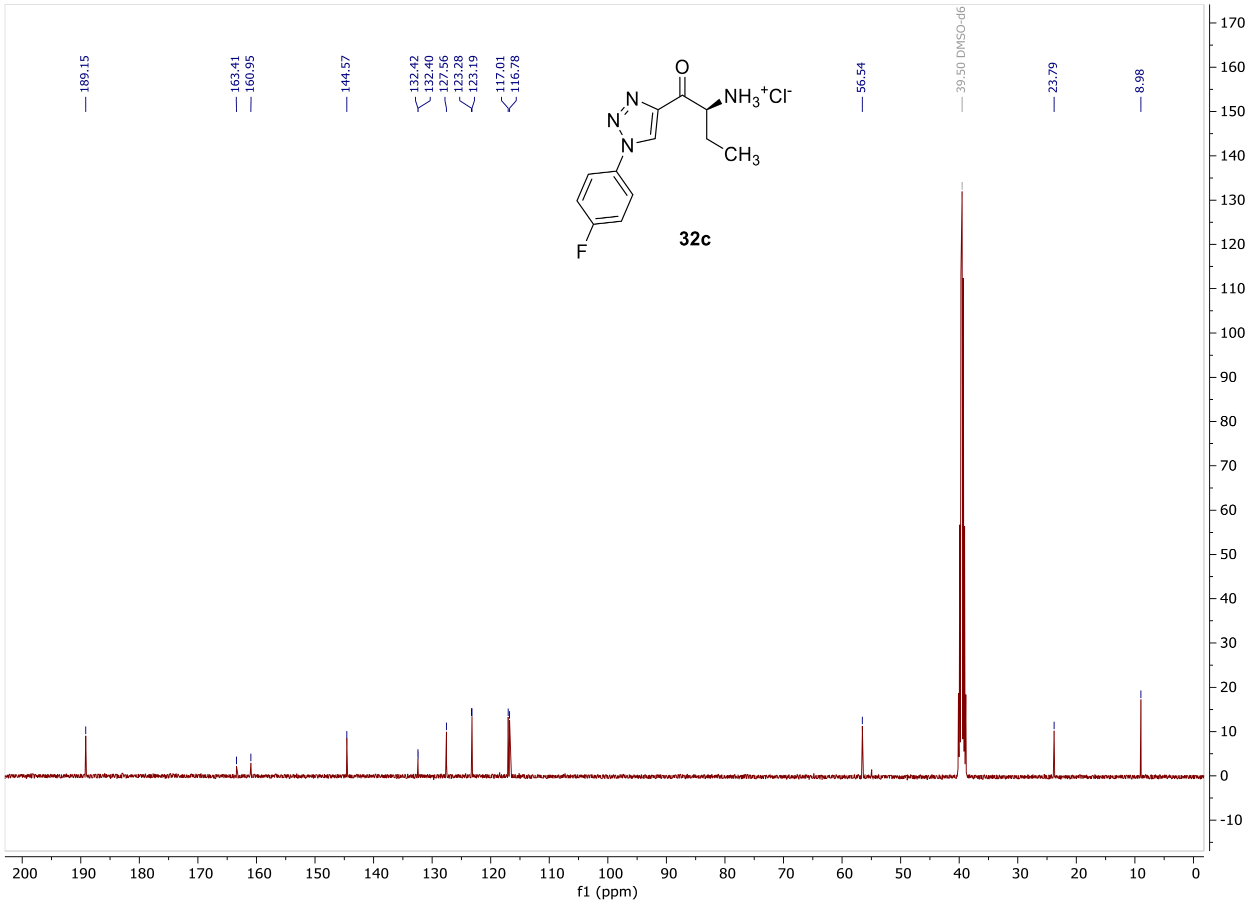

Compound **32c** (^{19}F NMR, 282 MHz):

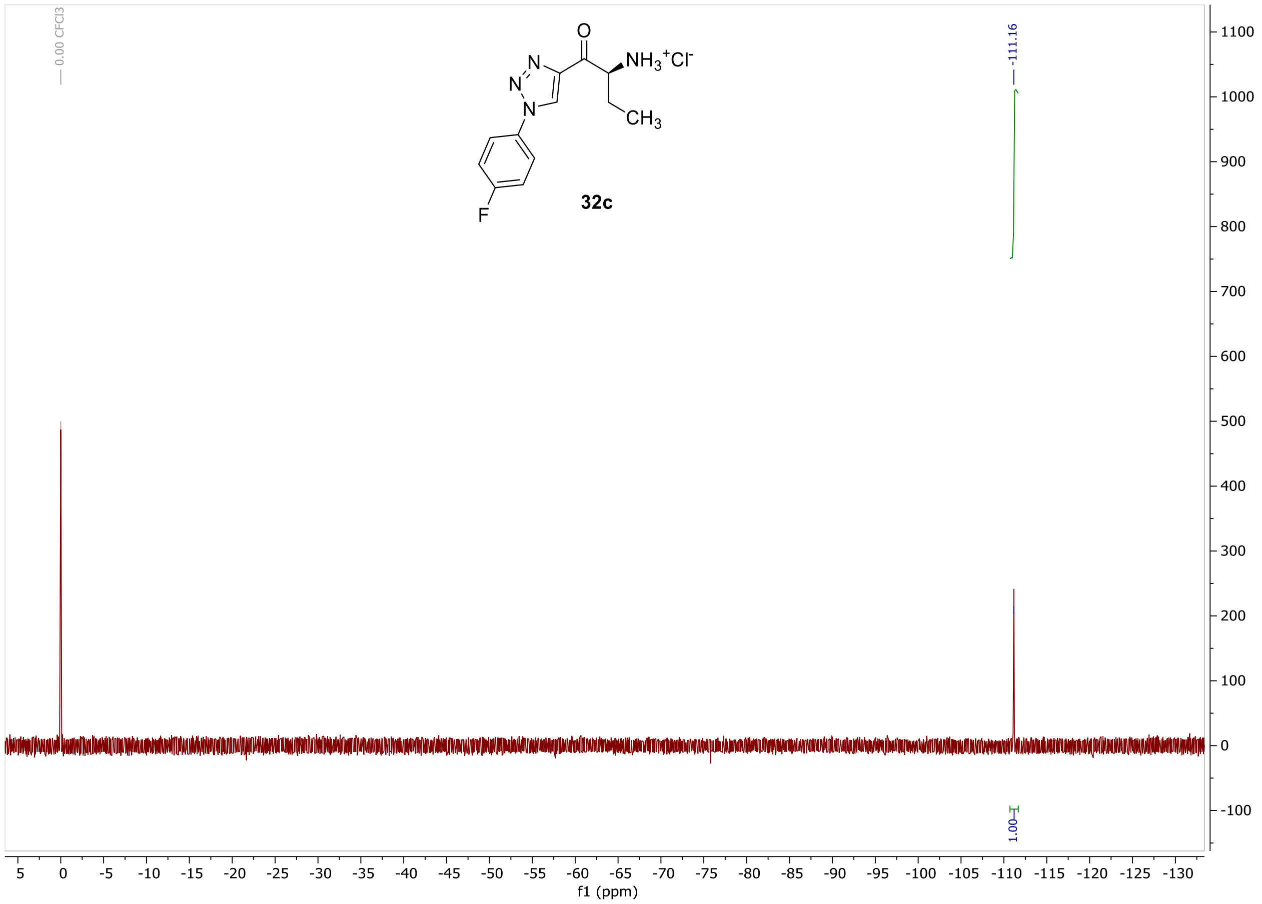

Compound **11** (^{1}H NMR, 400 MHz):

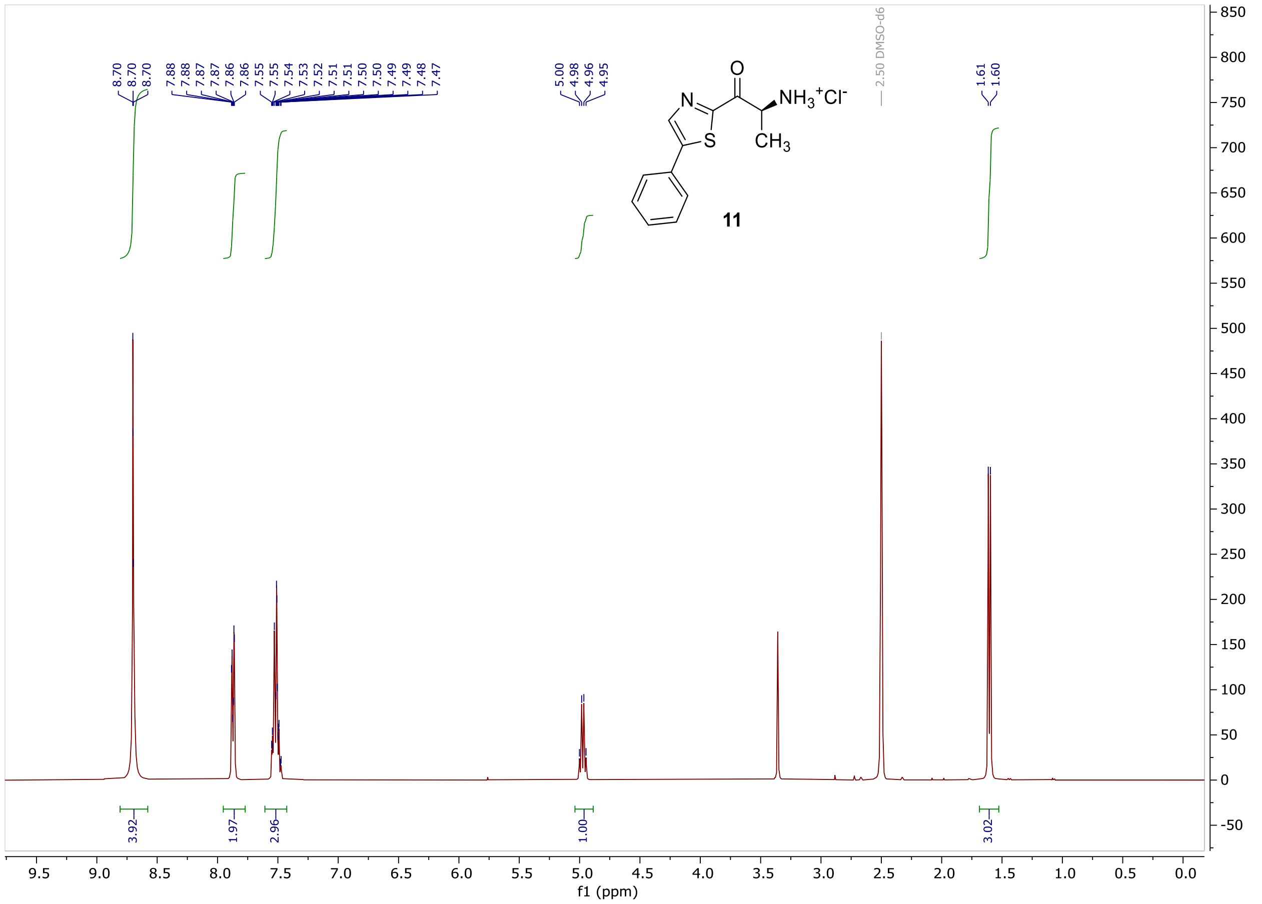

Compound **11** (^{13}C NMR, 101 MHz):

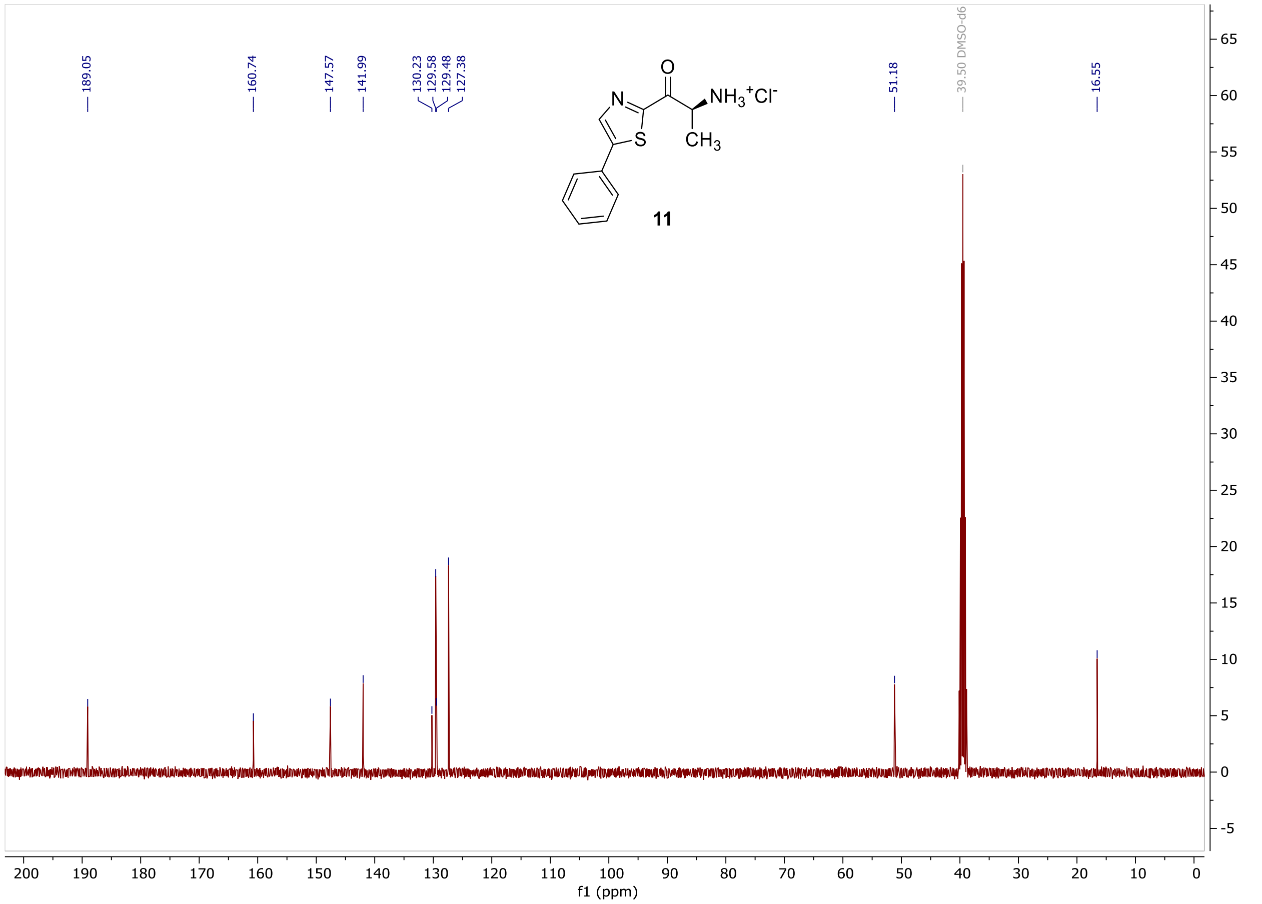

Compound **25a** (^{1}H NMR, 400 MHz):

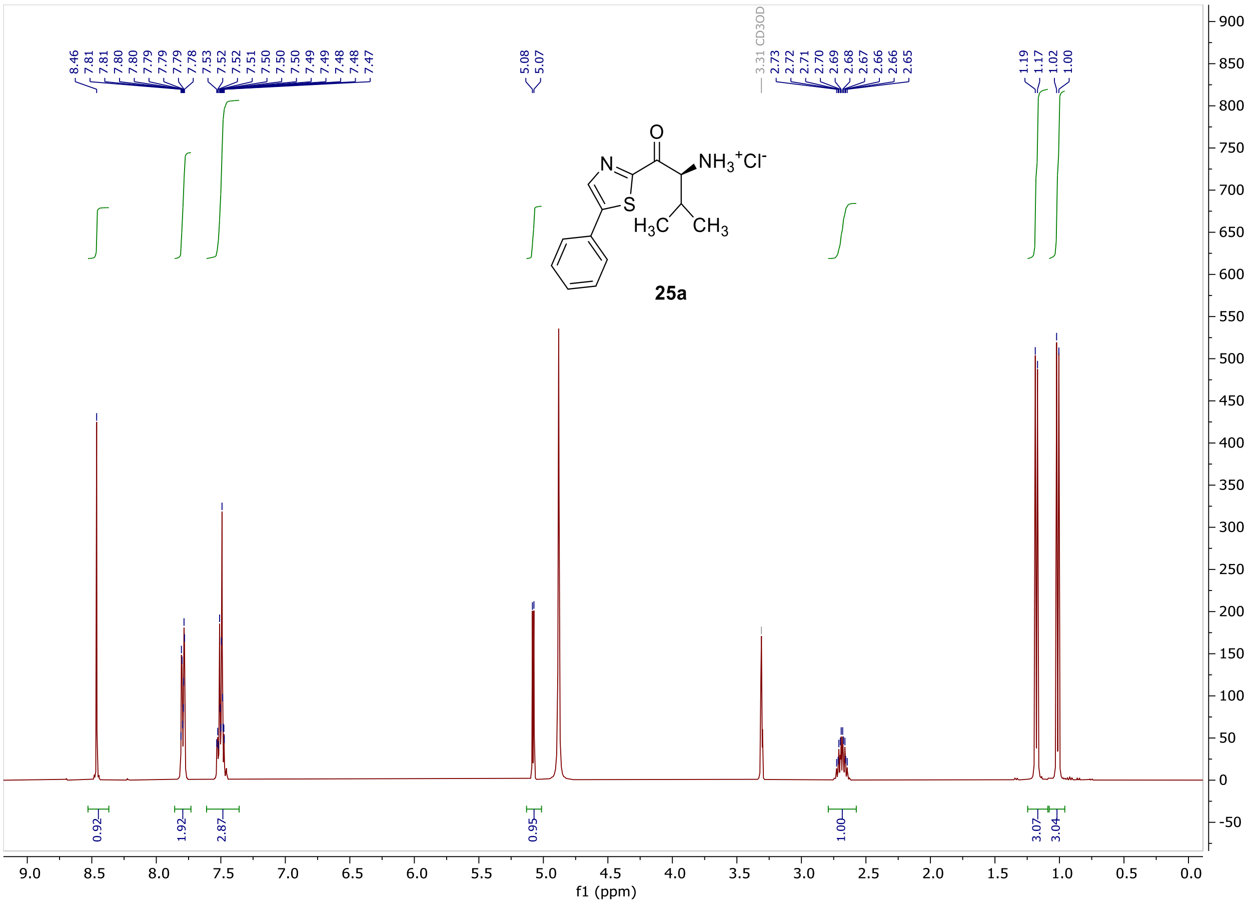

Compound **25a** (^{13}C NMR, 101 MHz):

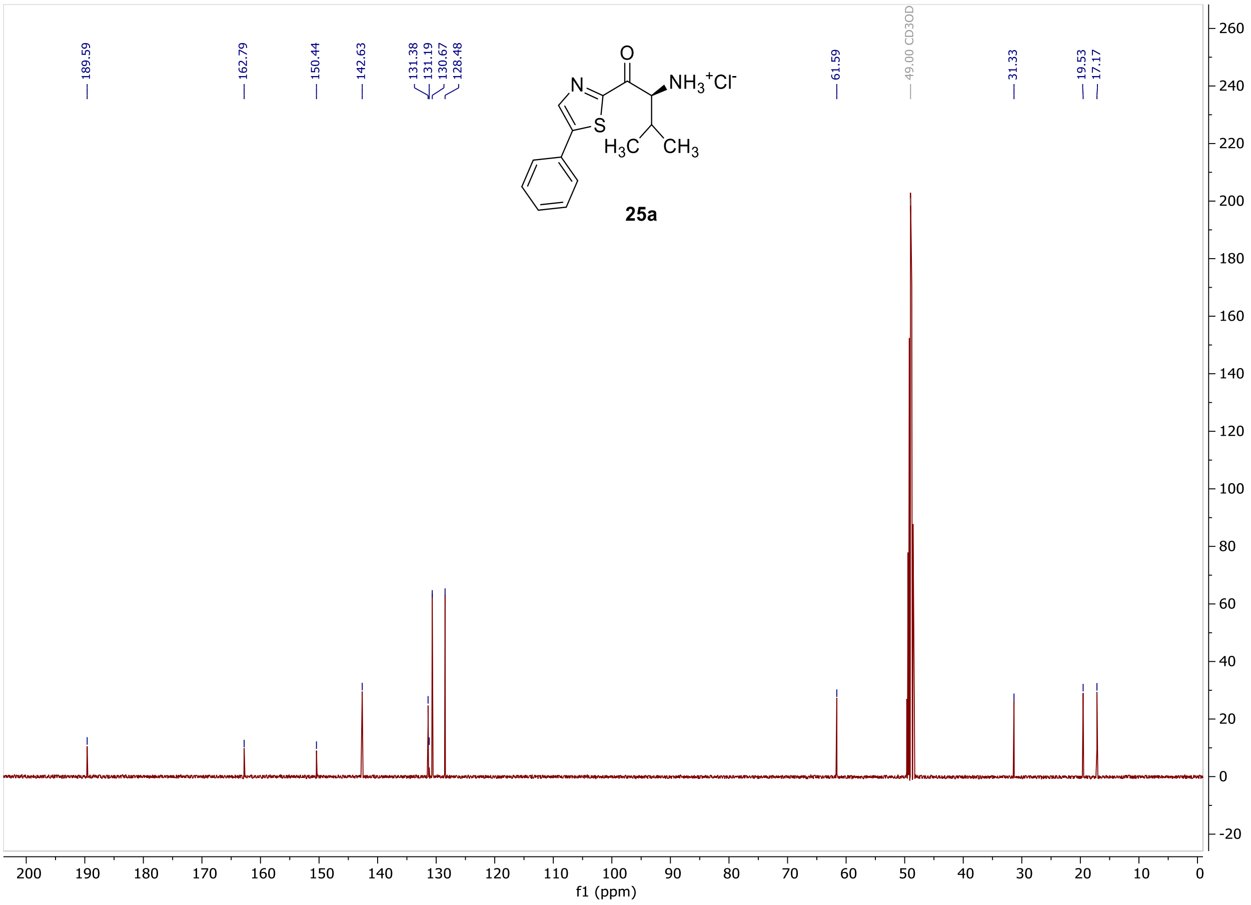

Compound **25b** (^{1}H NMR, 400 MHz):

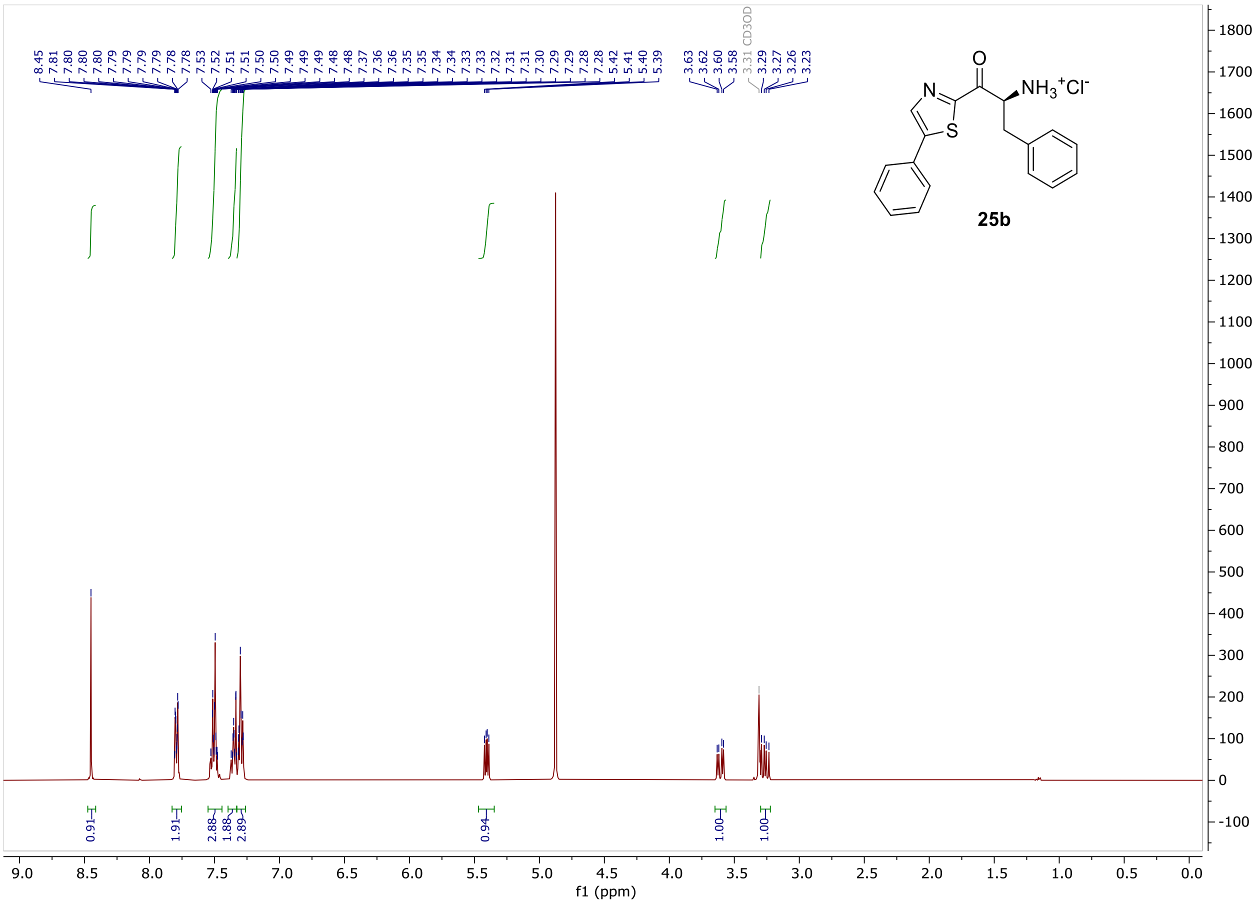

Compound **25b** (^{13}C NMR, 101 MHz):

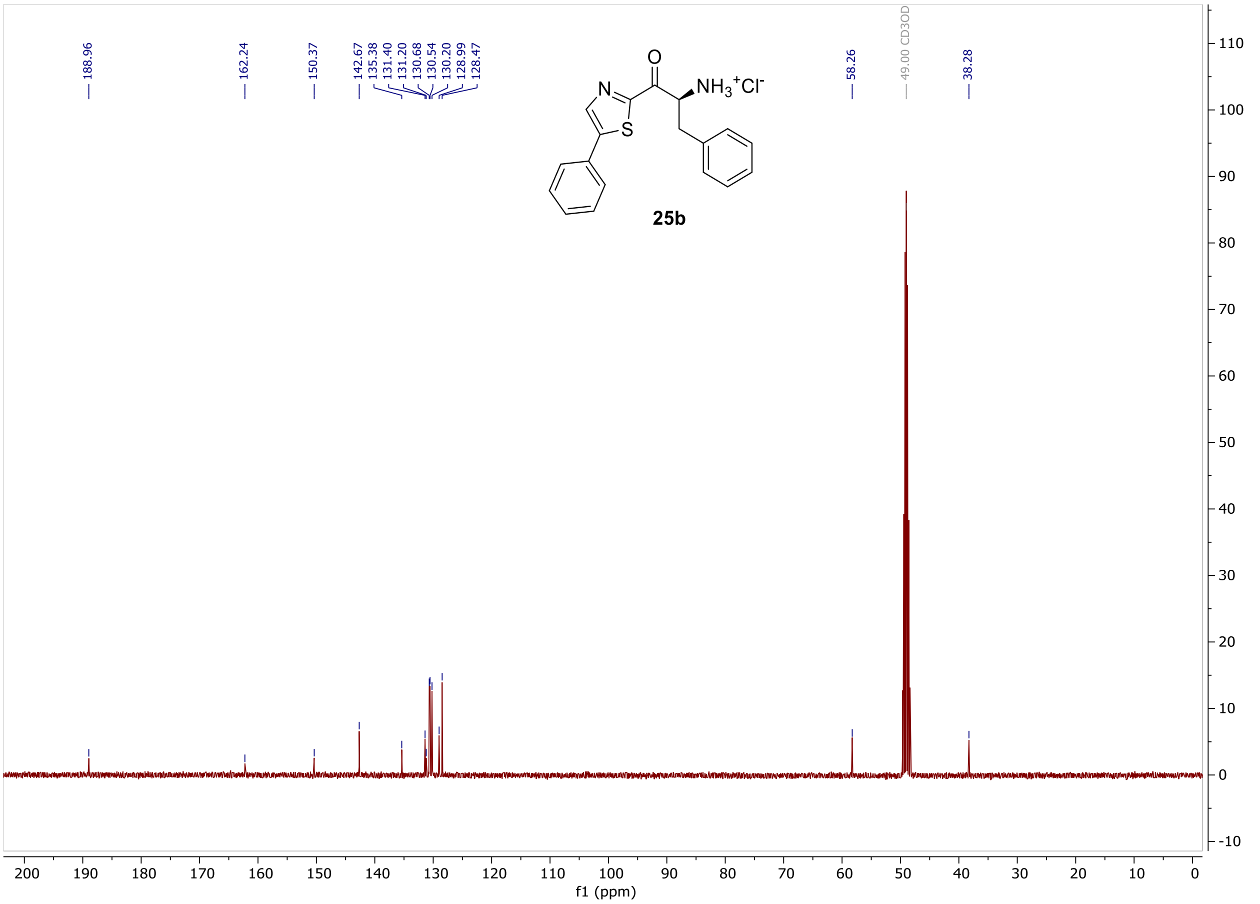

Compound **25c** (^{1}H NMR, 400 MHz):

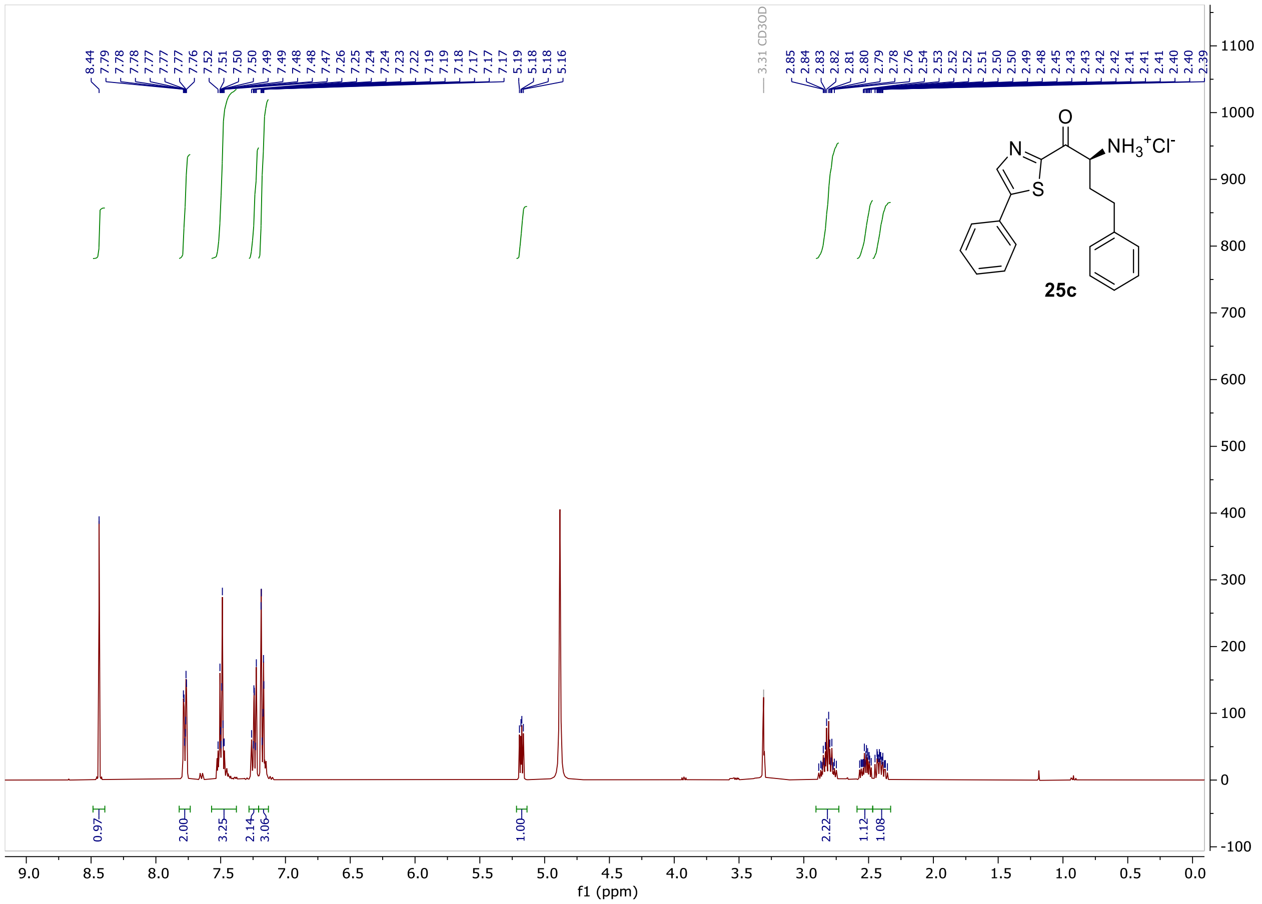

Compound **25c** (^{13}C NMR, 101 MHz):

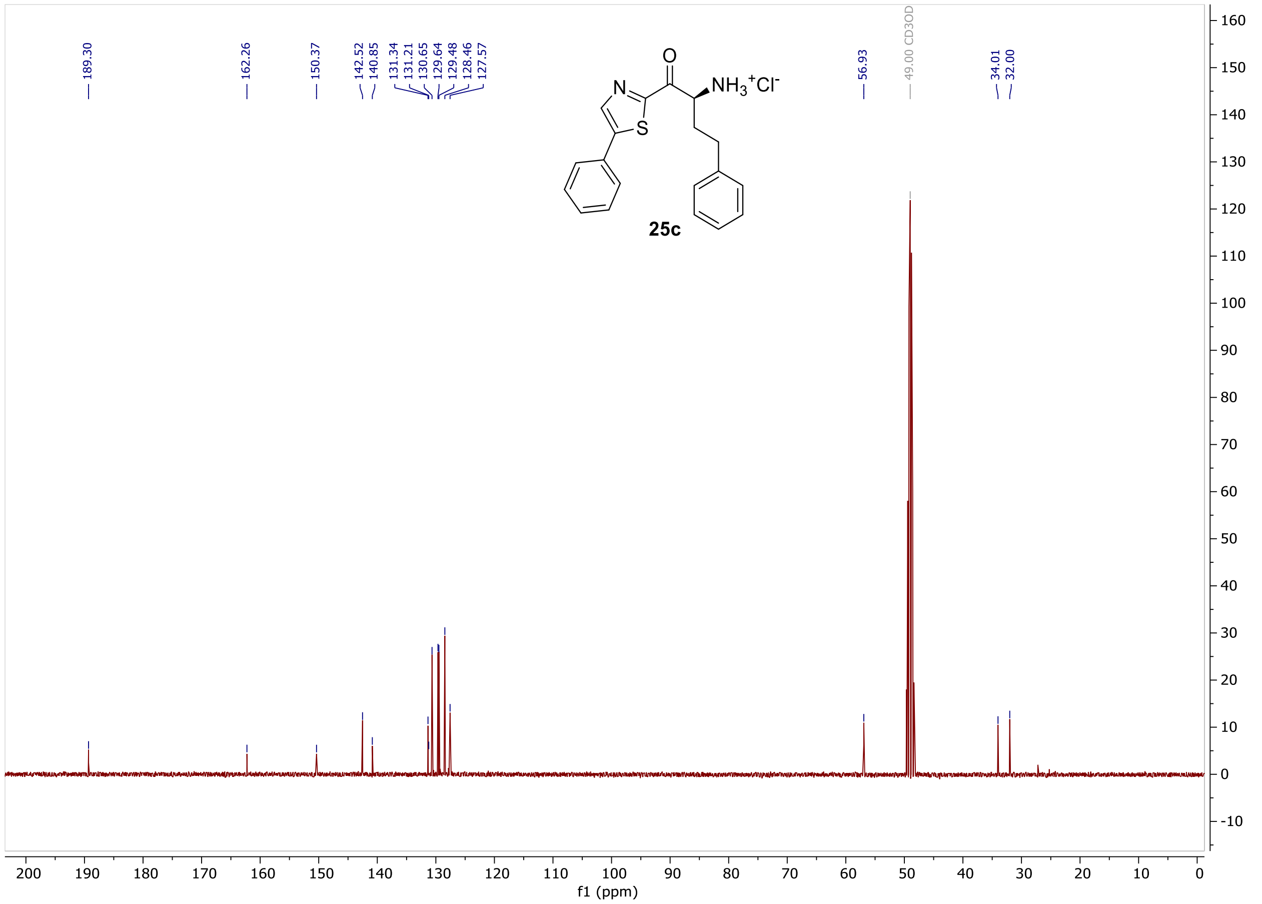

Compound **25d** (^{1}H NMR, 400 MHz):

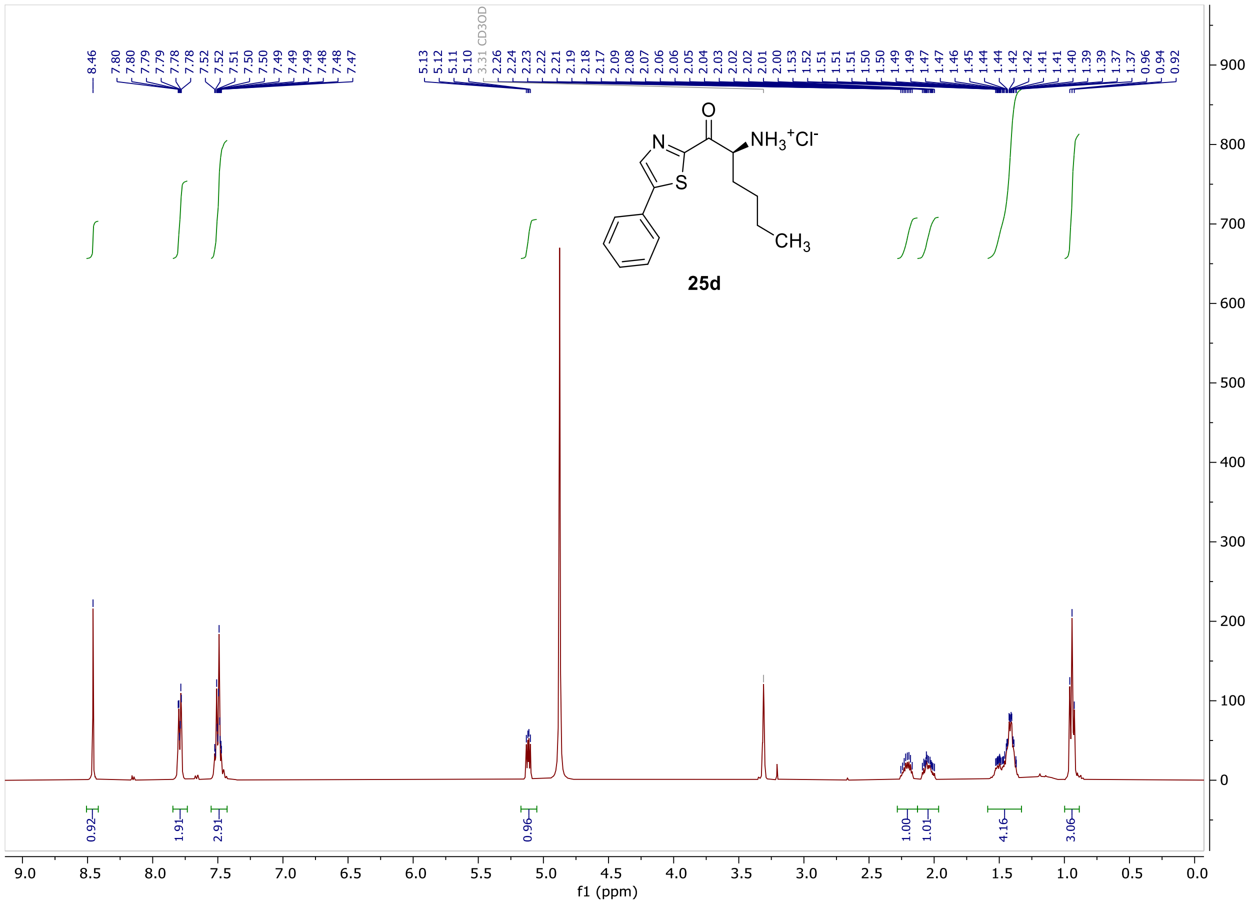

Compound **25d** (^{13}C NMR, 101 MHz):

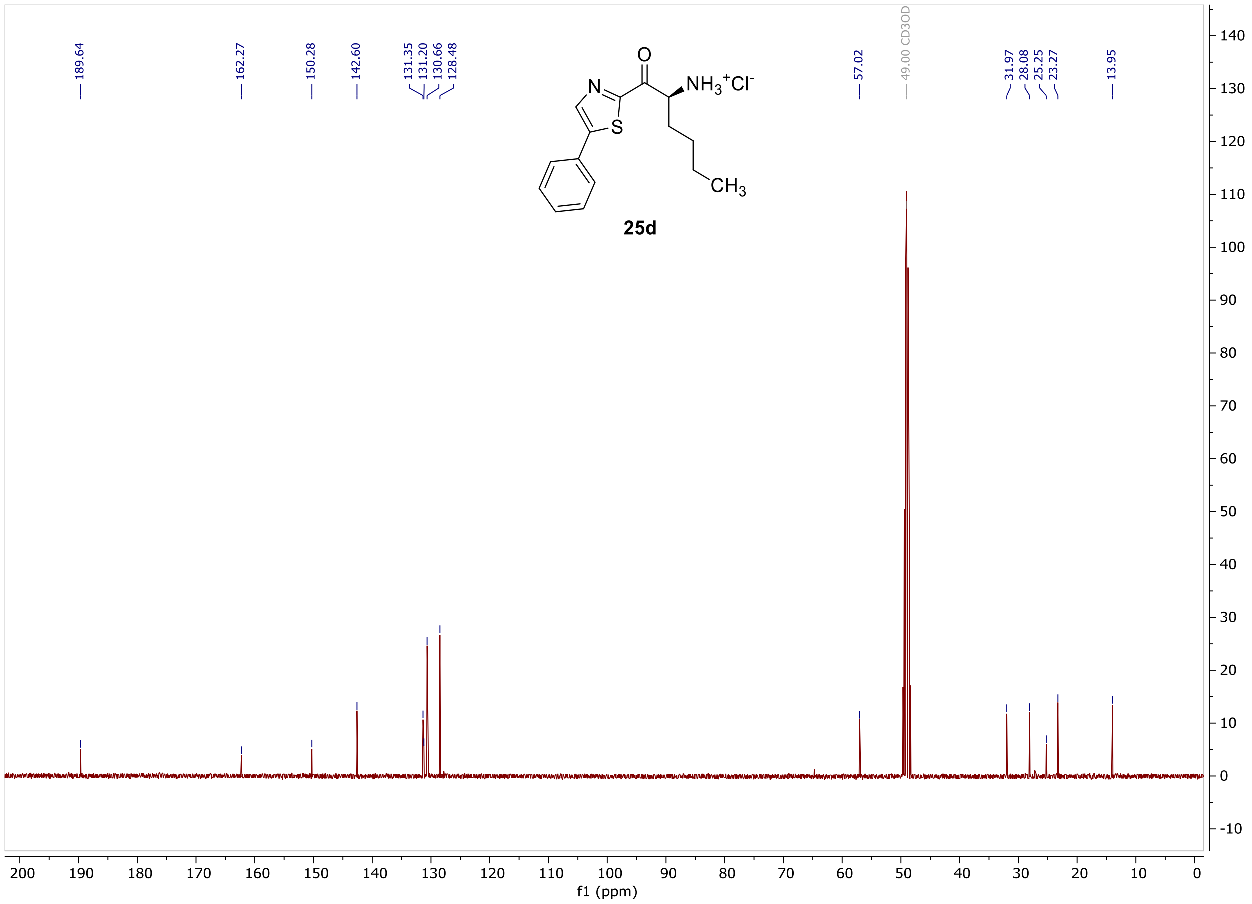

Compound **25e** (^{1}H NMR, 400 MHz):

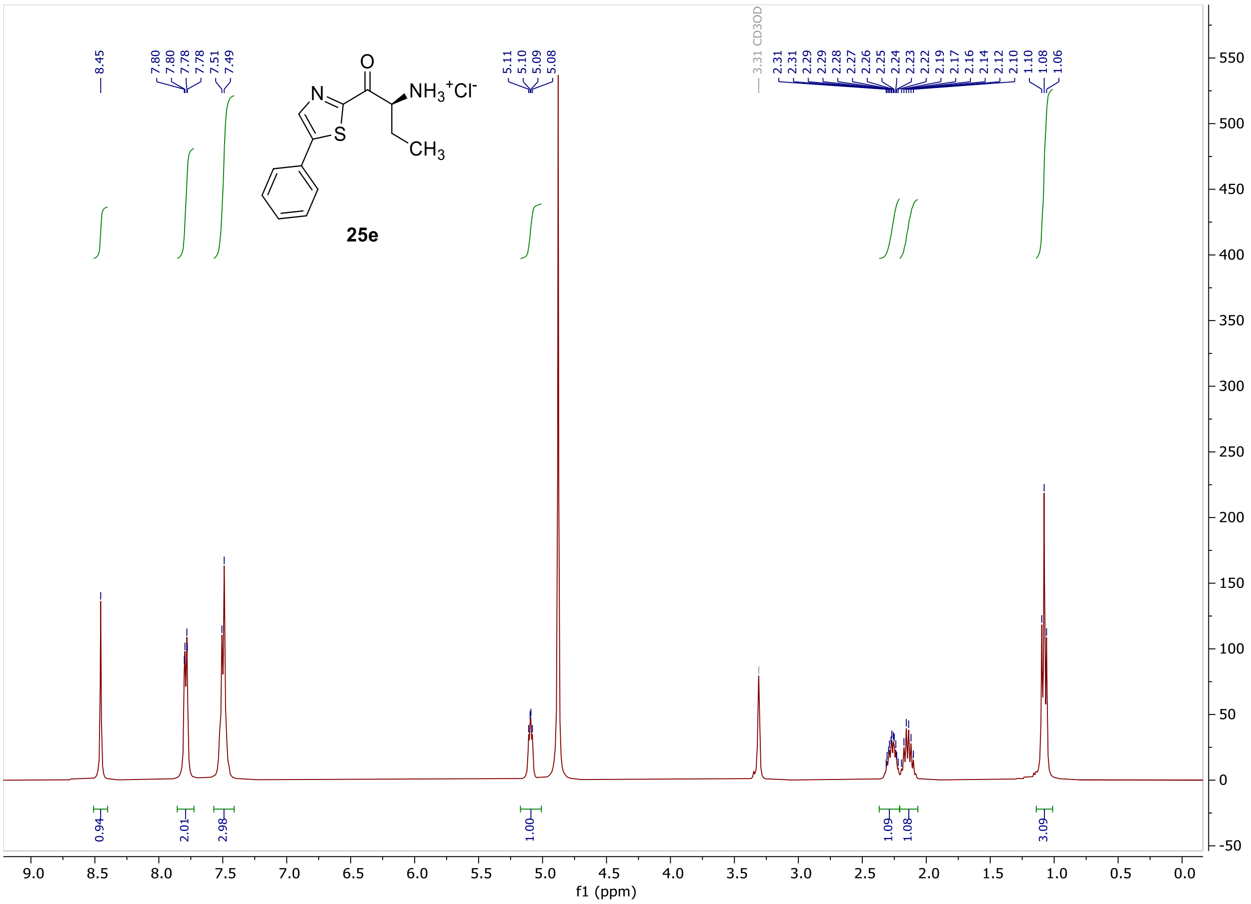

Compound **25e** (^{13}C NMR, 101 MHz):

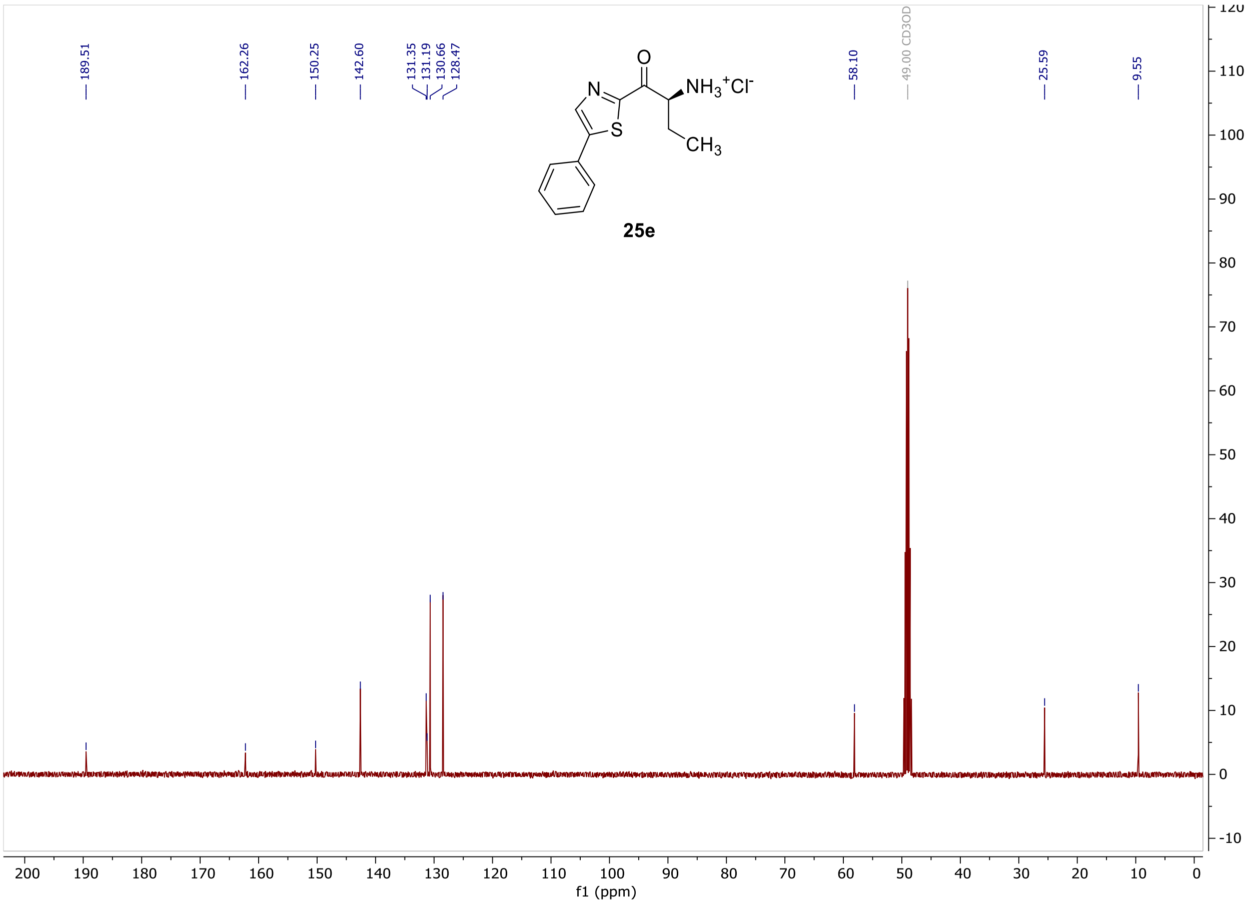

Compound **25f** (^{1}H NMR, 400 MHz):

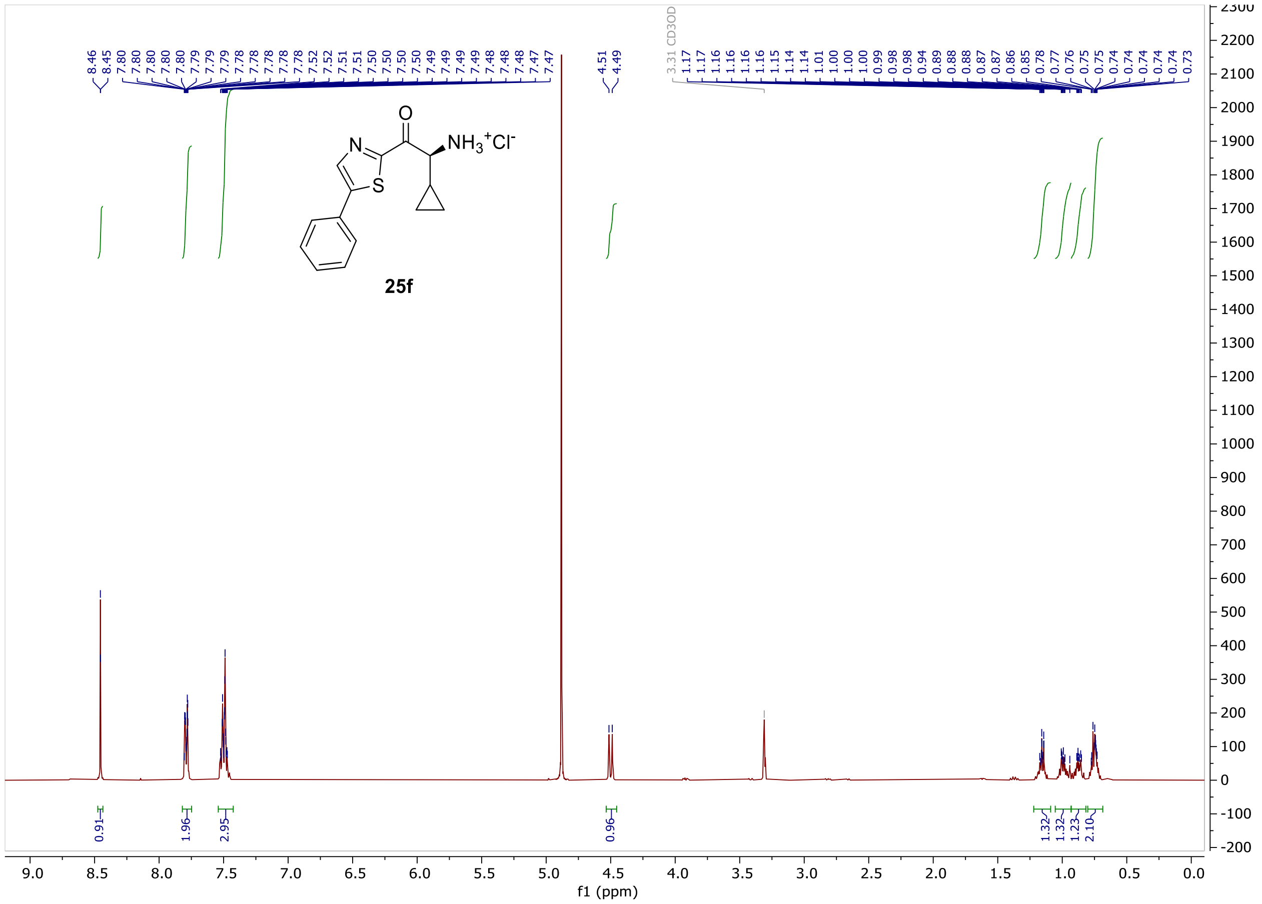

Compound **25f** (^{13}C NMR, 101 MHz):

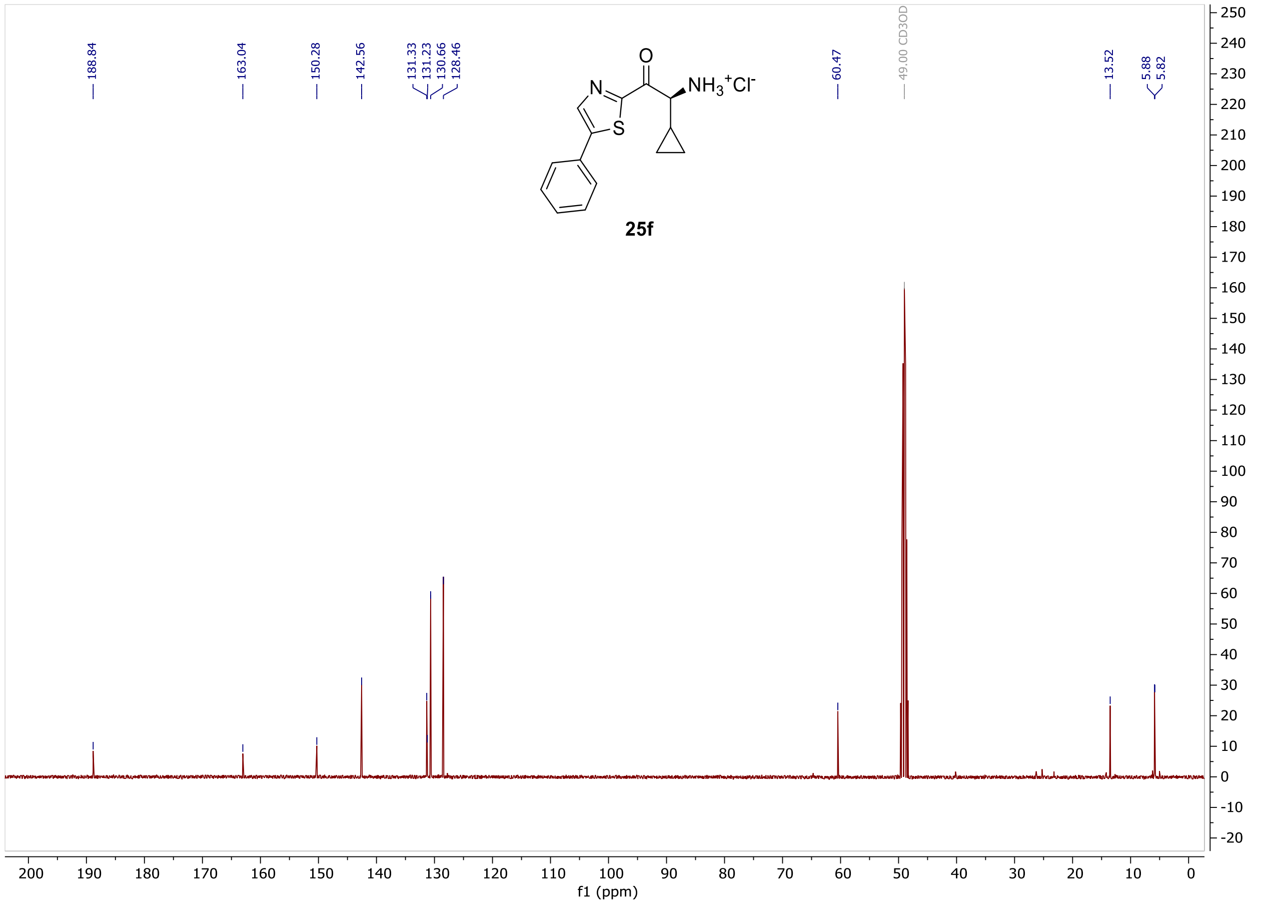

Compound **34a** (^{1}H NMR, 400 MHz):

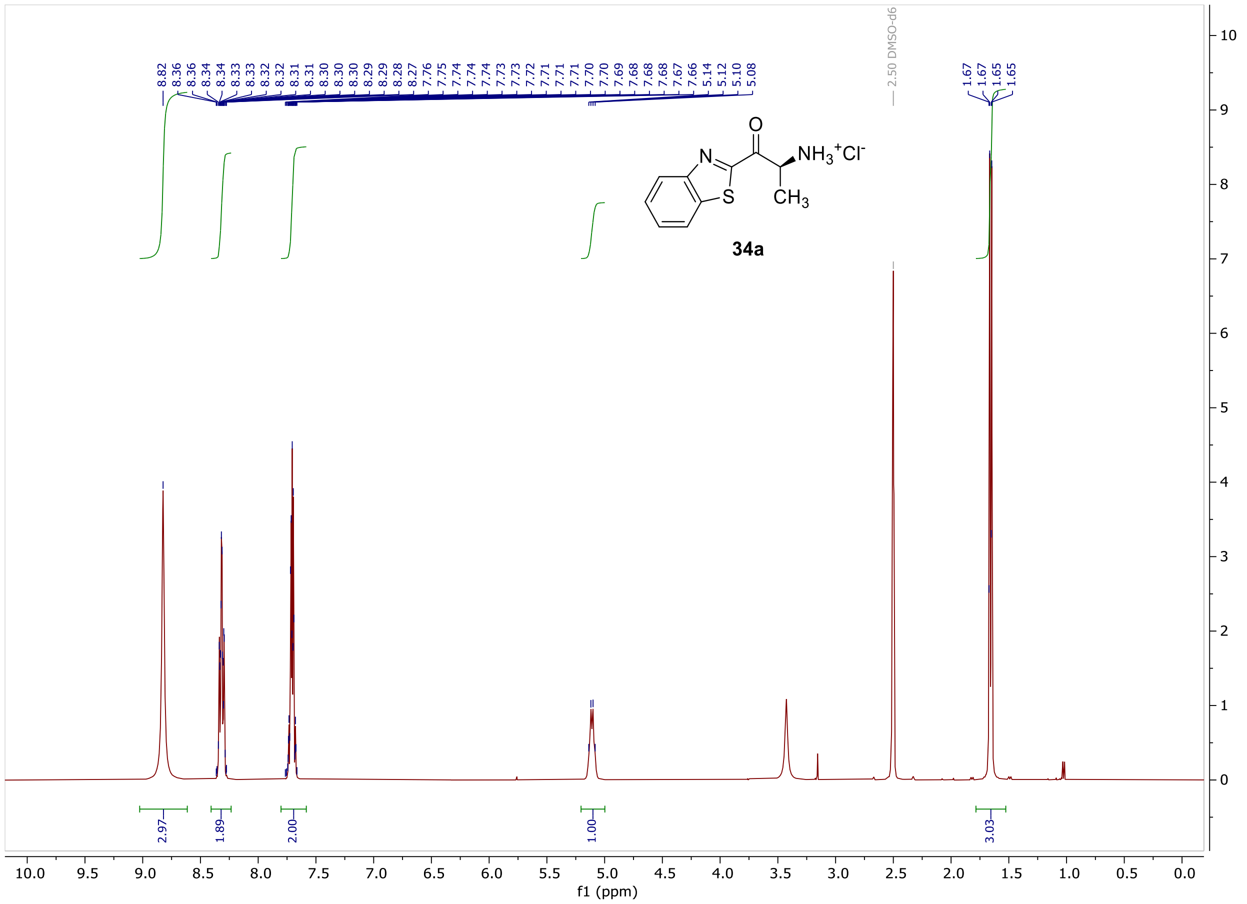

Compound **34a** (^{13}C NMR, 101 MHz):

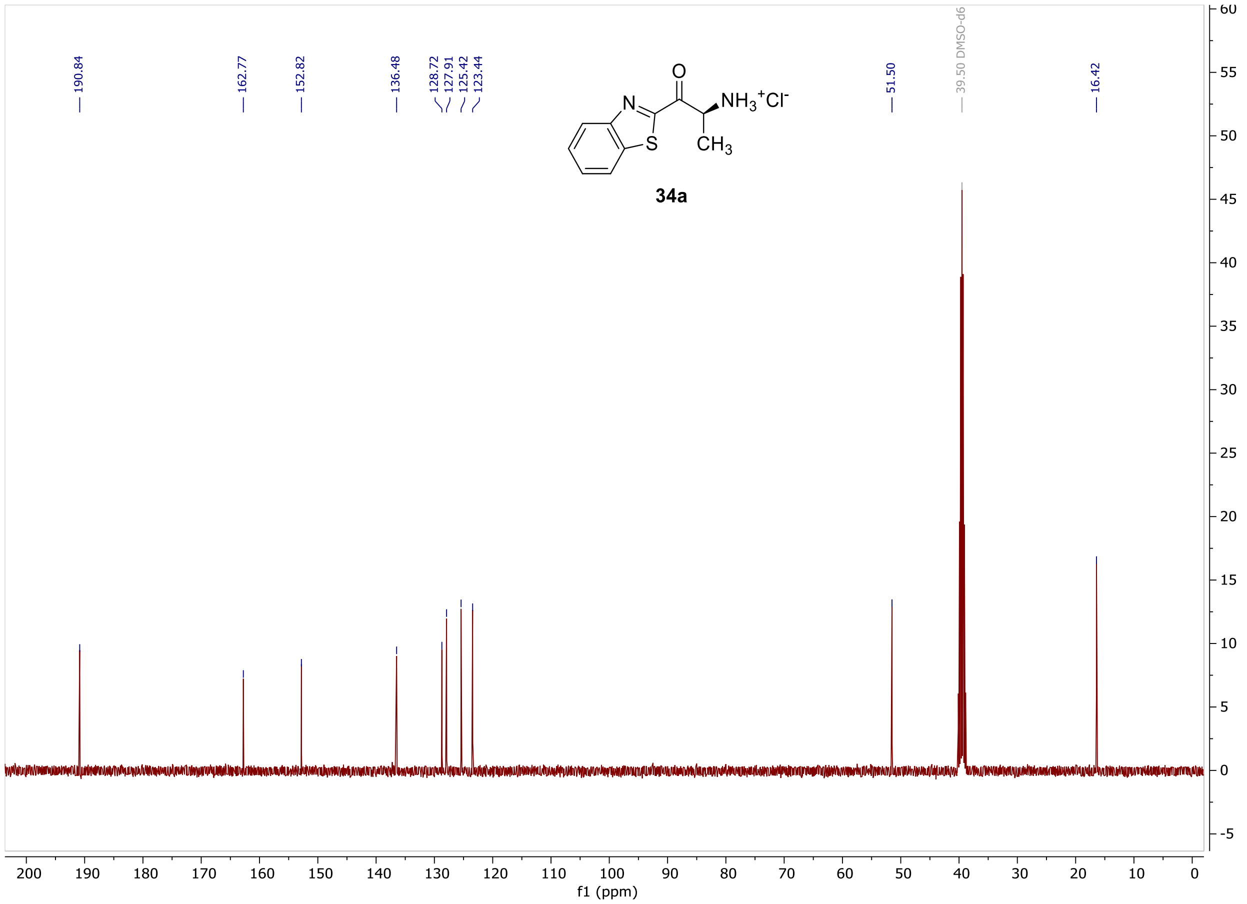

Compound **34b** (^{1}H NMR, 400 MHz):

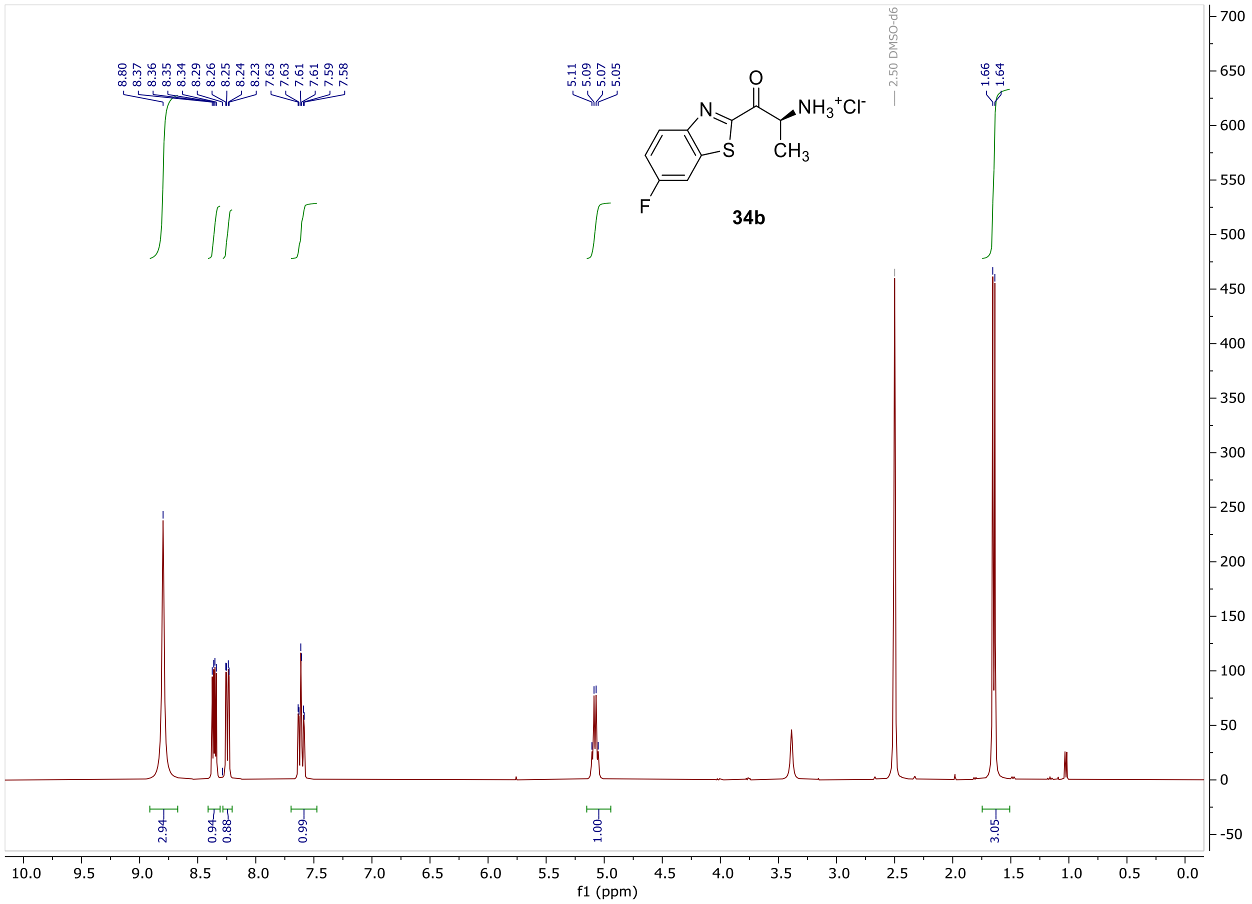

Compound **34b** (^{13}C NMR, 101 MHz):

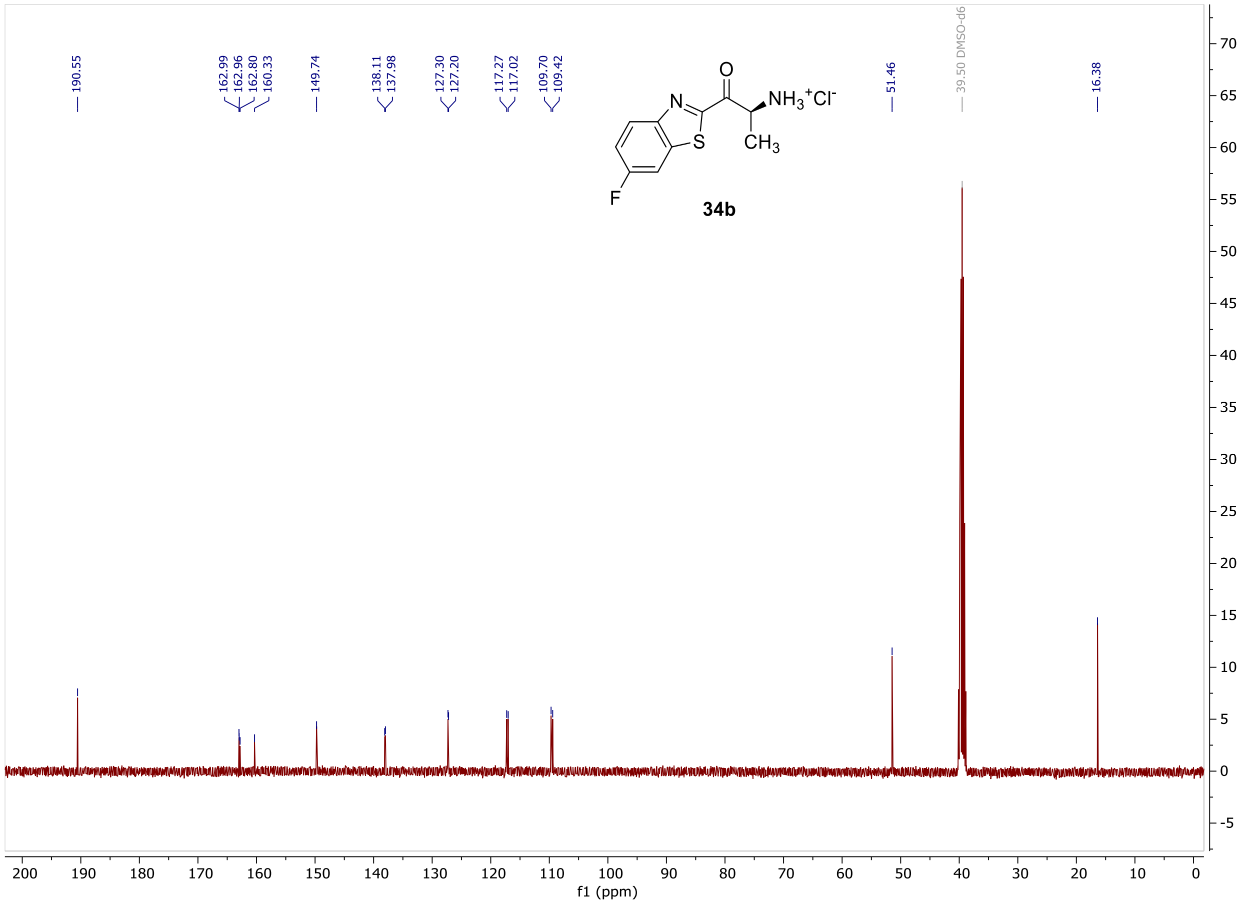

Compound **34b** (^{19}F NMR, 282 MHz):

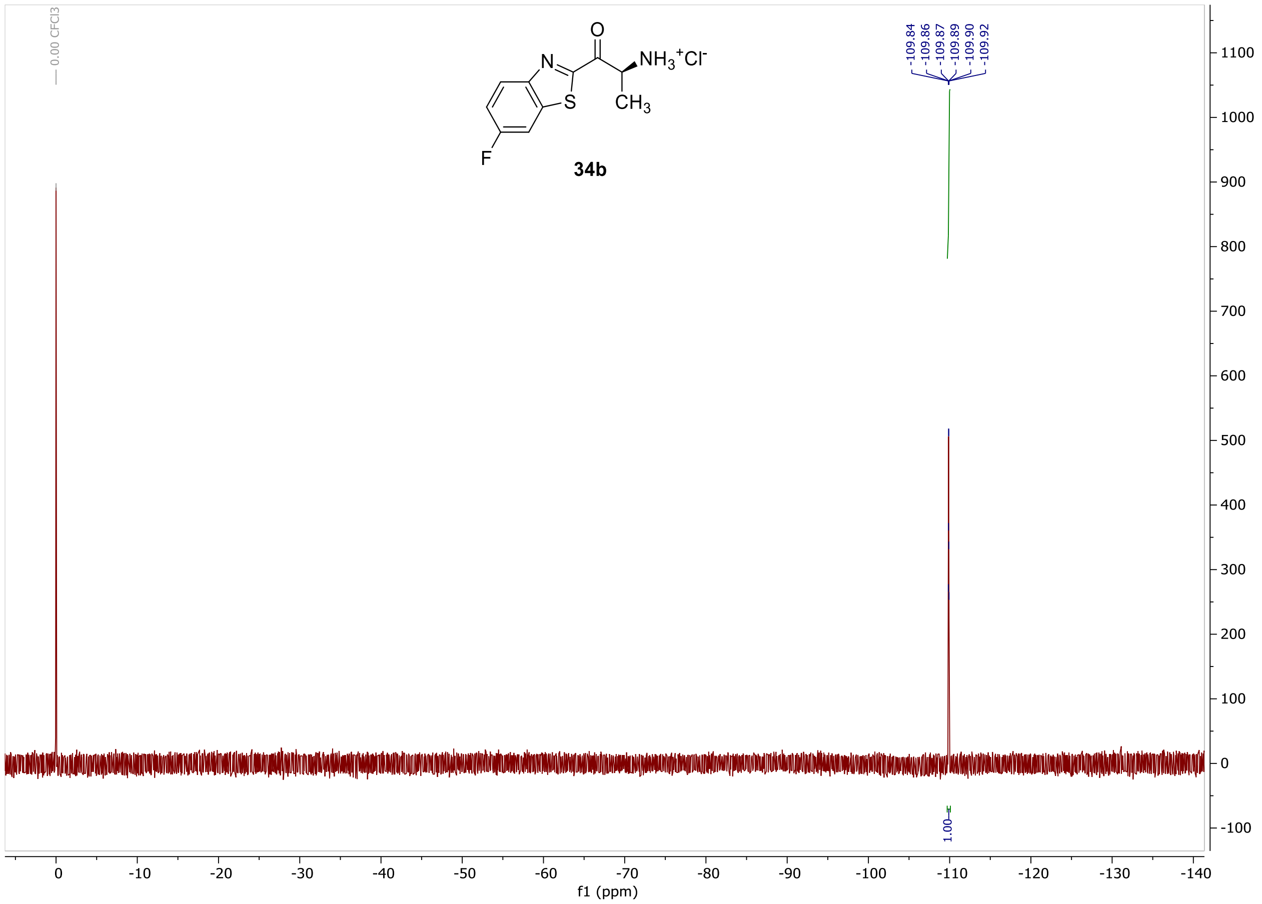

Compound **34c** (^{1}H NMR, 400 MHz):

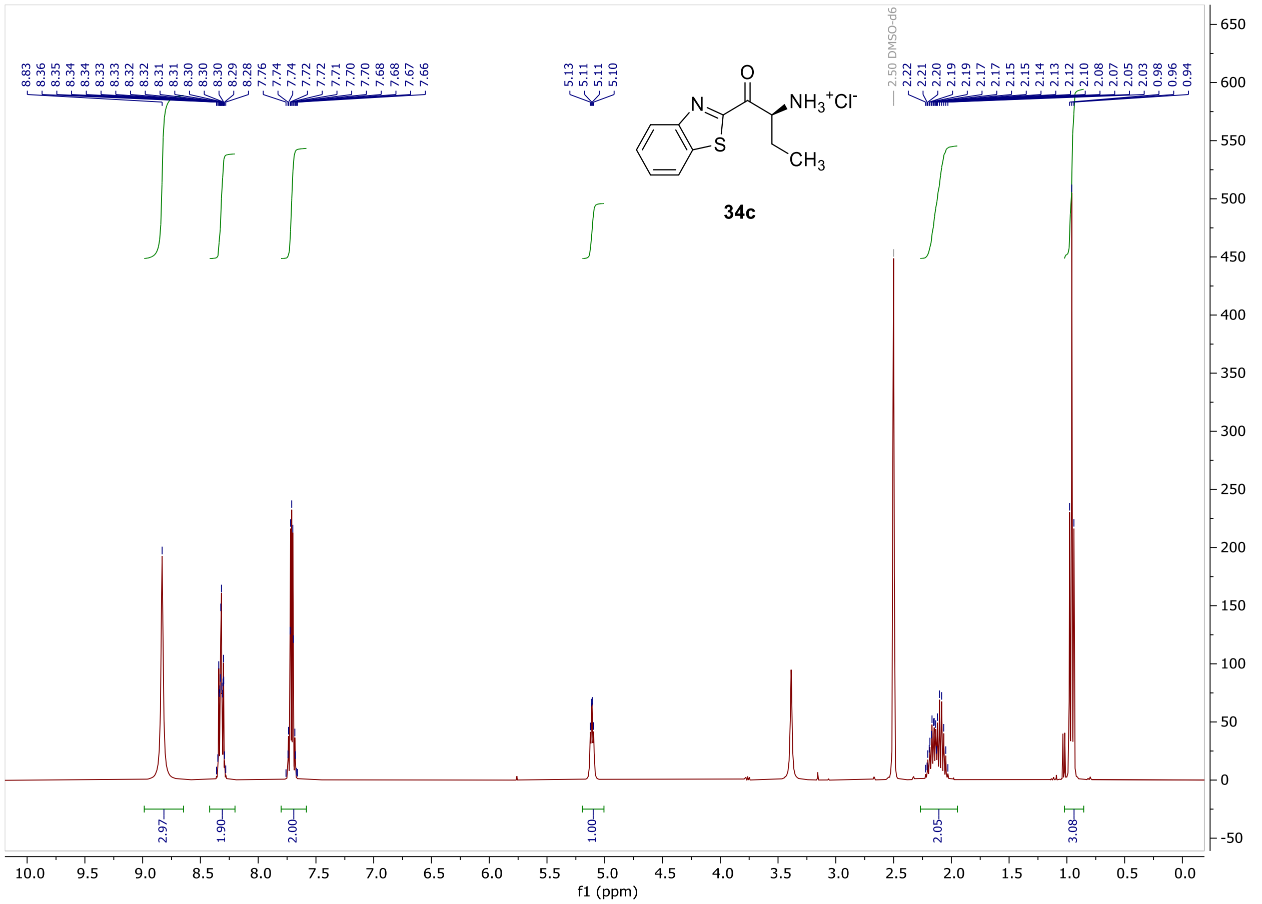

Compound **34c** (^{13}C NMR, 101 MHz):

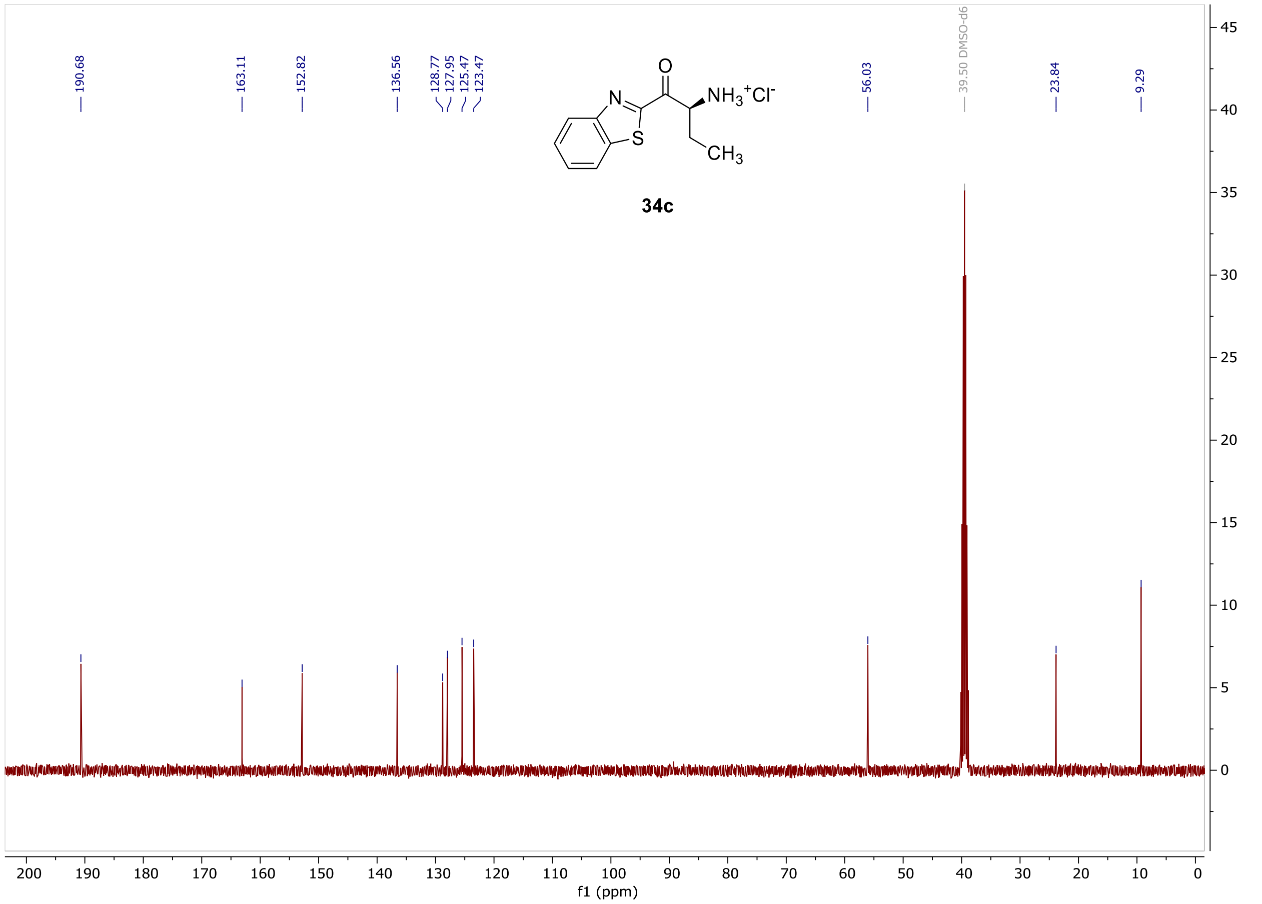

Compound **34d** (^{1}H NMR, 400 MHz):

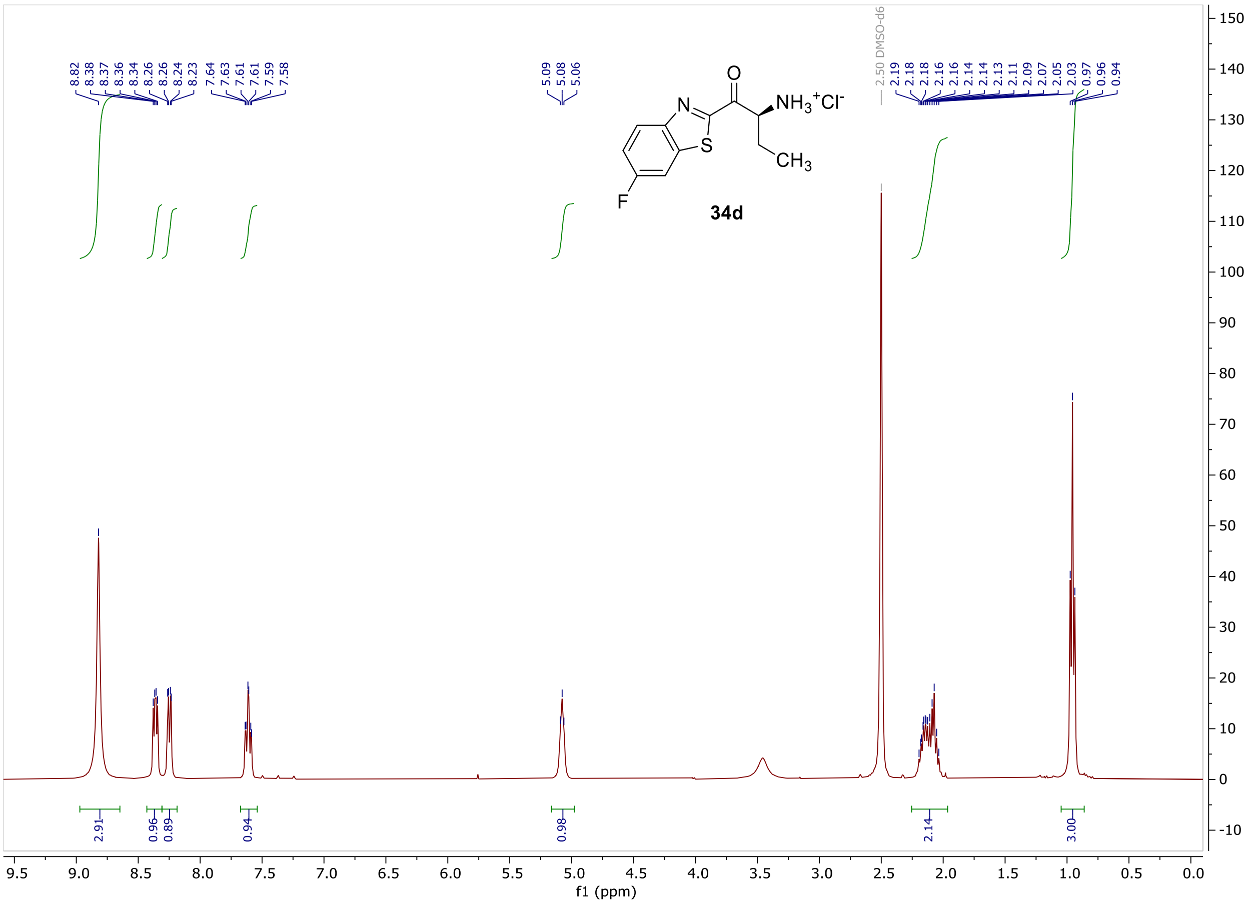

Compound **34d** (^{13}C NMR, 101 MHz):

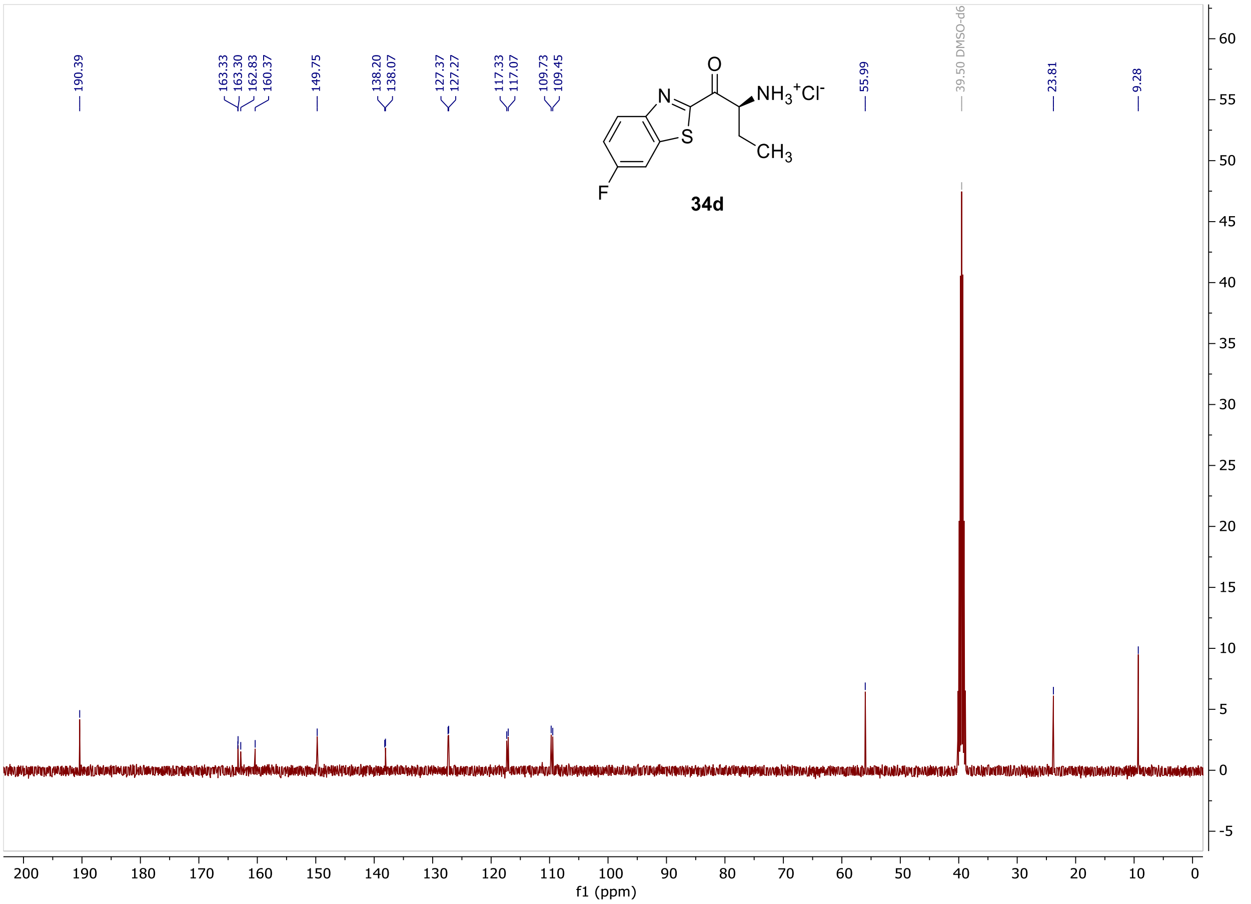

Compound **34d** (^{19}F NMR, 282 MHz):

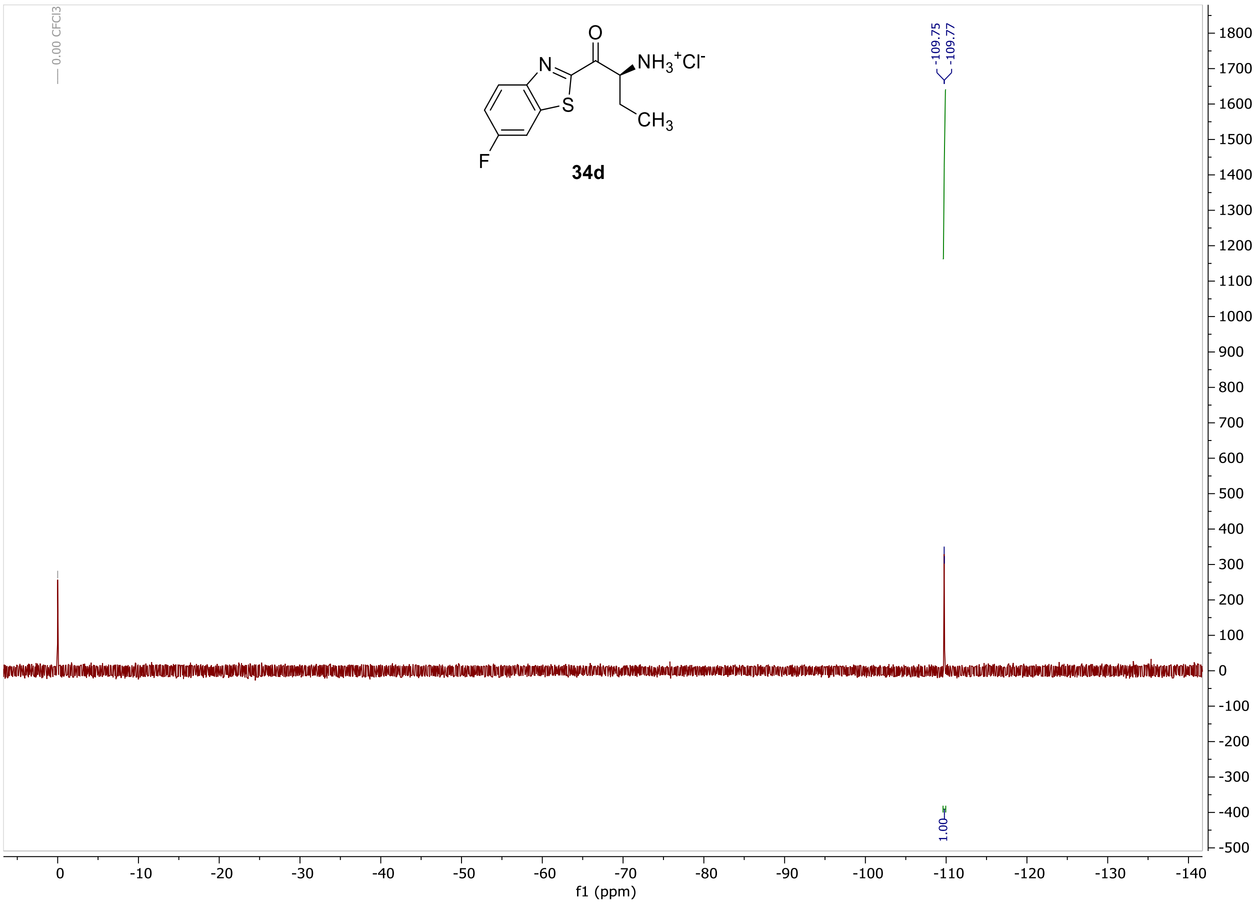

Compound **49a** (^{1}H NMR, 400 MHz):

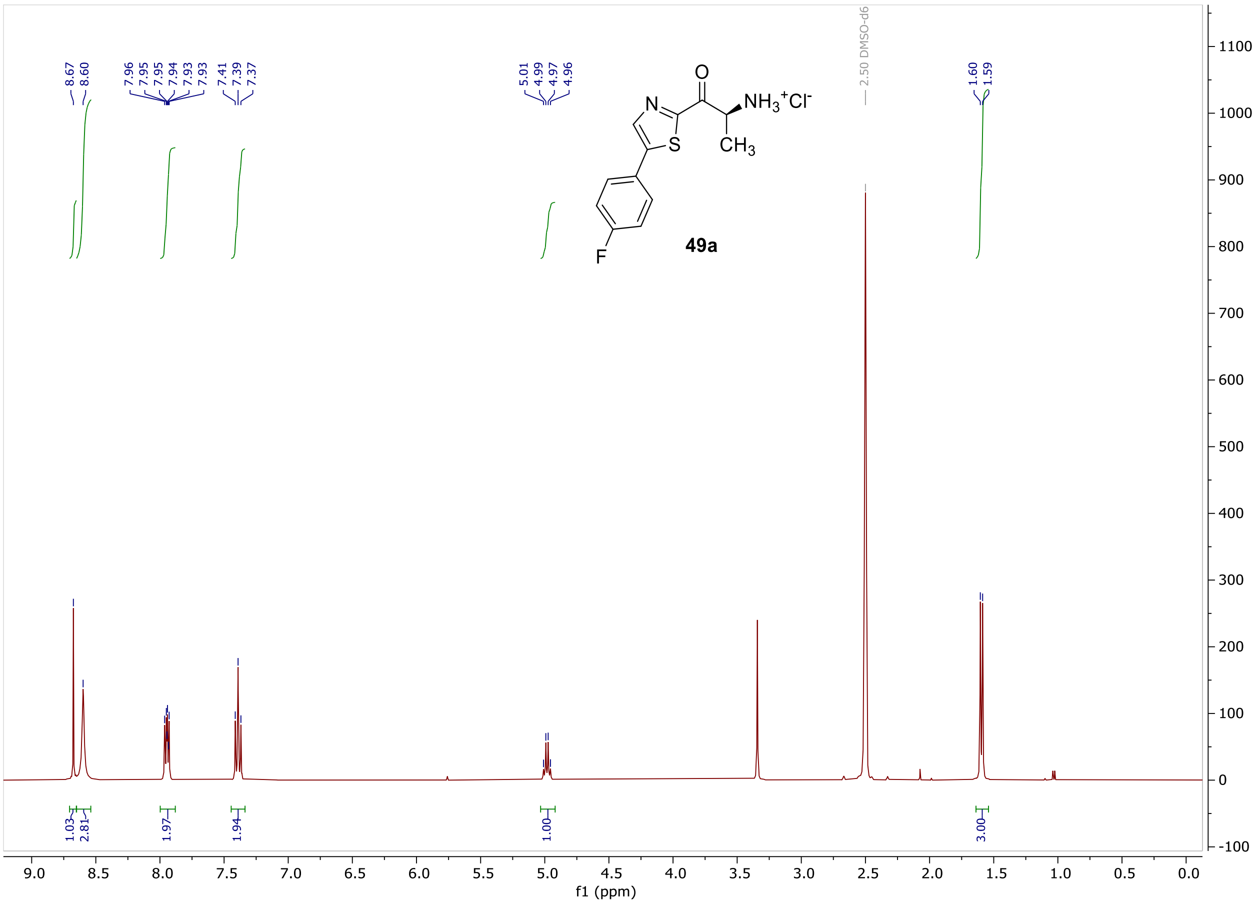

Compound **49a** (^{13}C NMR, 101 MHz):

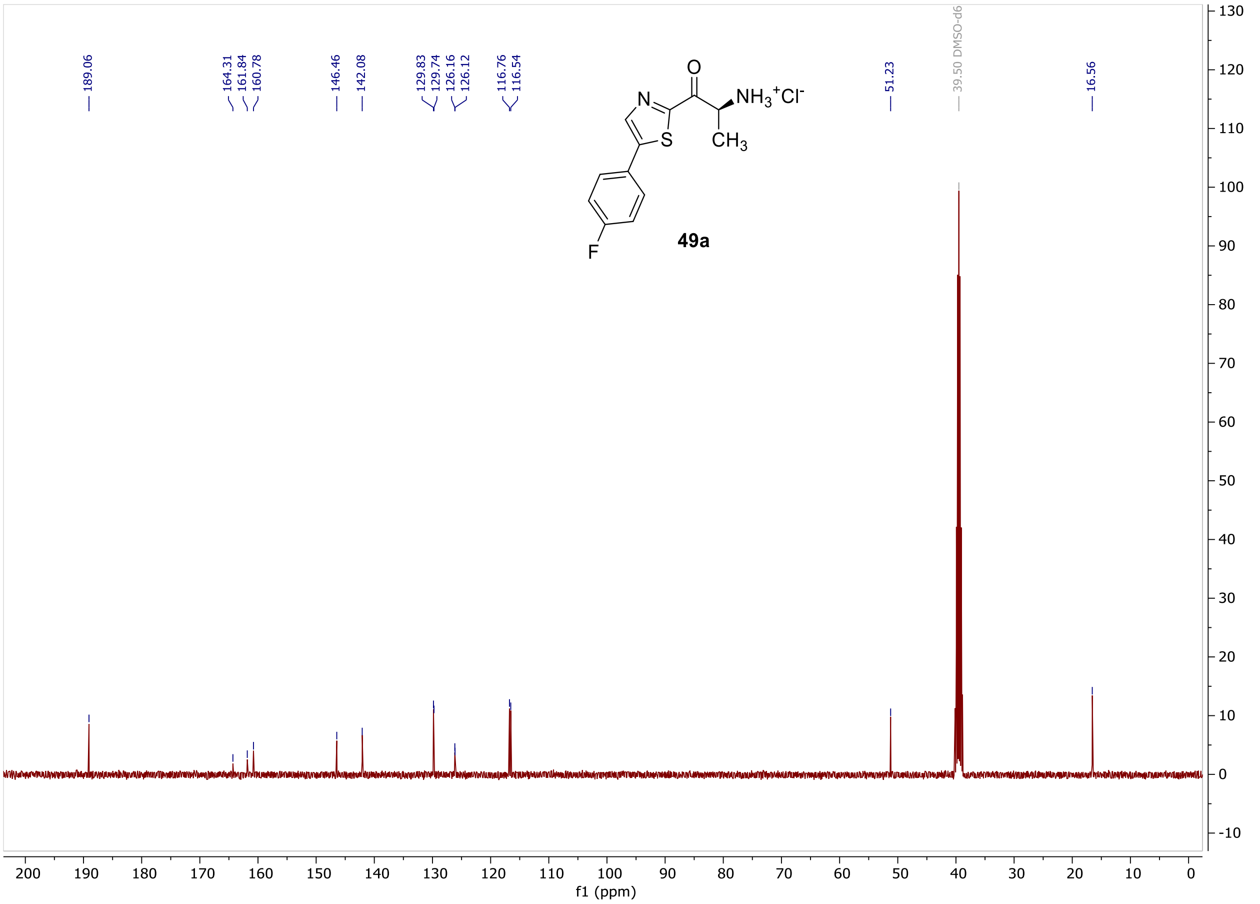

Compound **49a** (^{19}F NMR, 282 MHz):

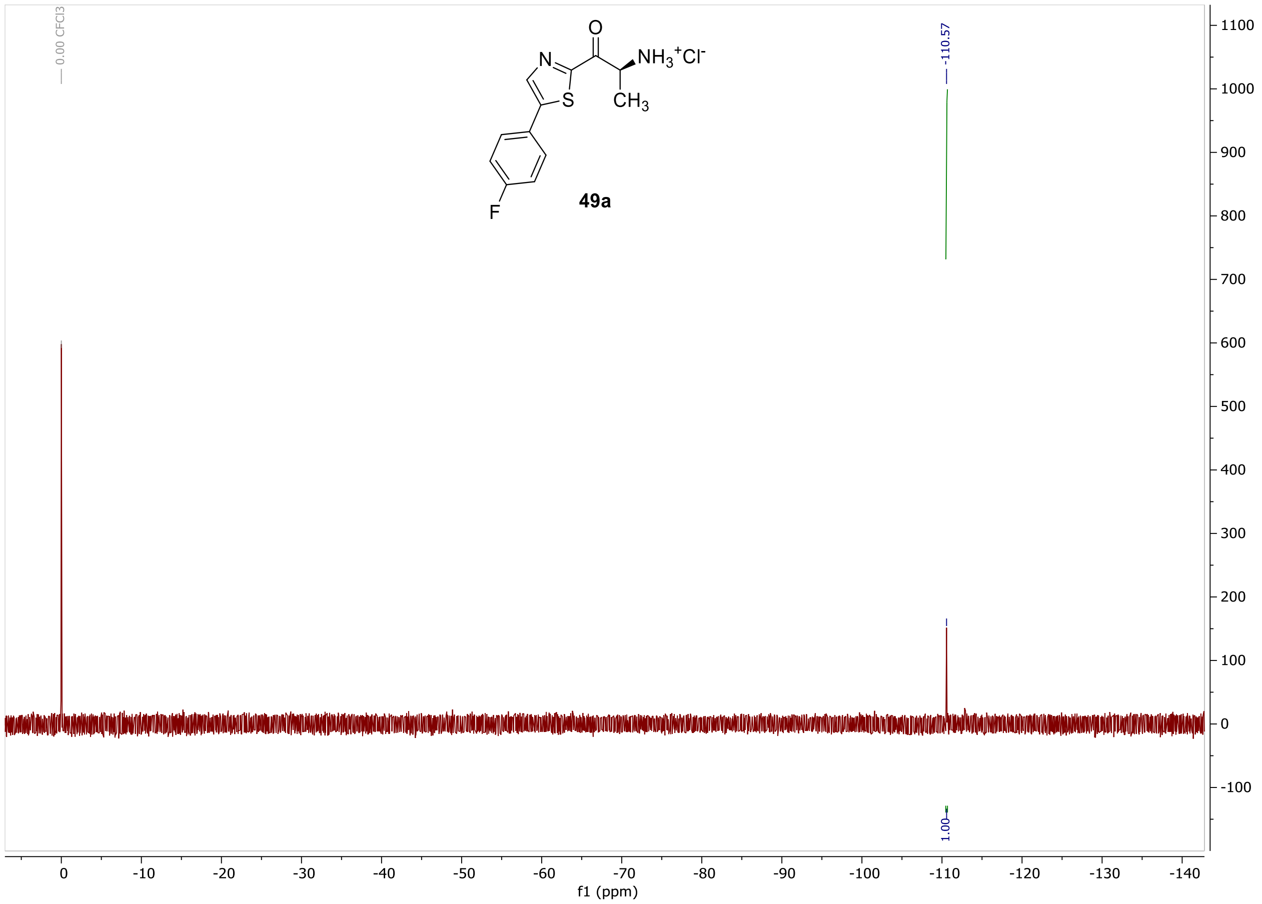

Compound **49b** (^{1}H NMR, 400 MHz):

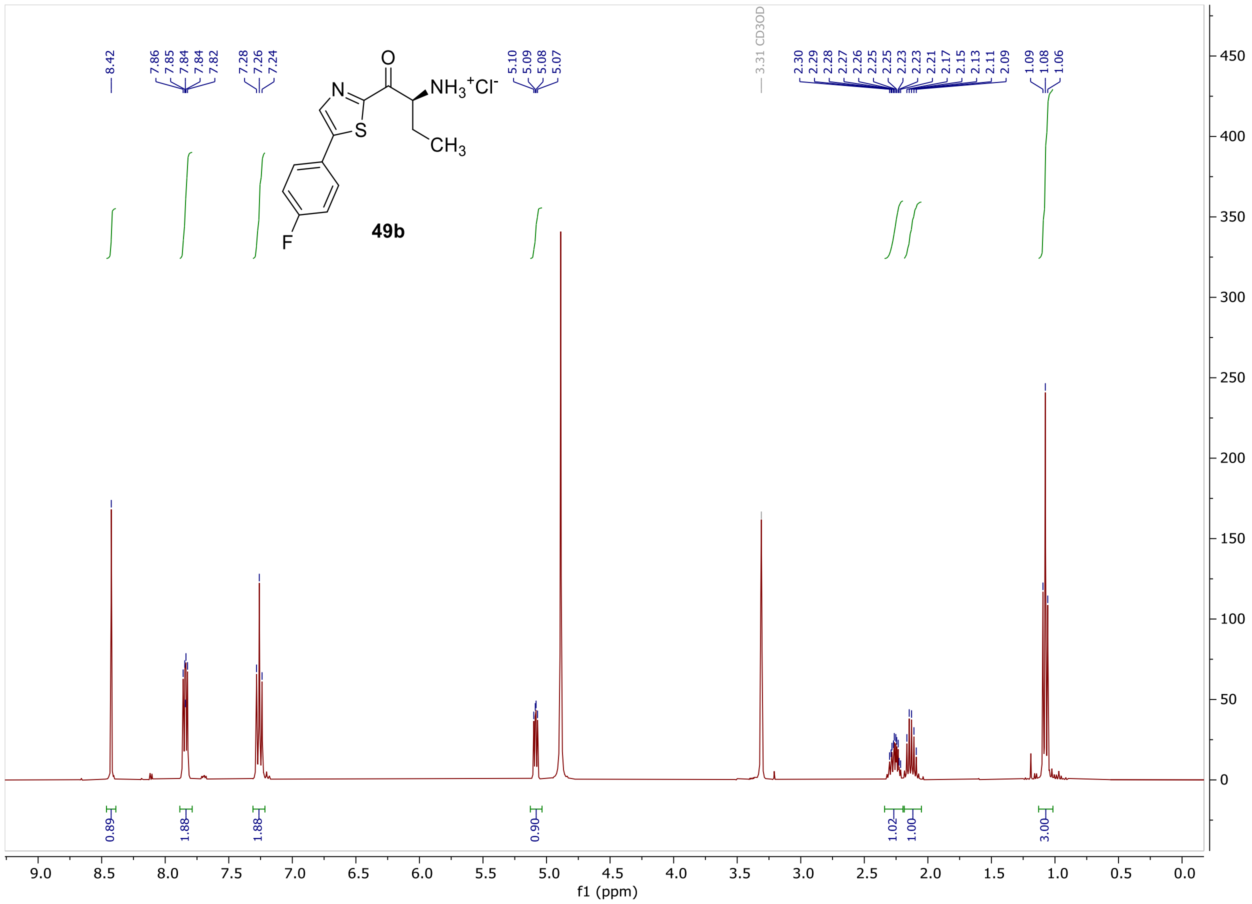

Compound **49b** (^{13}C NMR, 101 MHz):

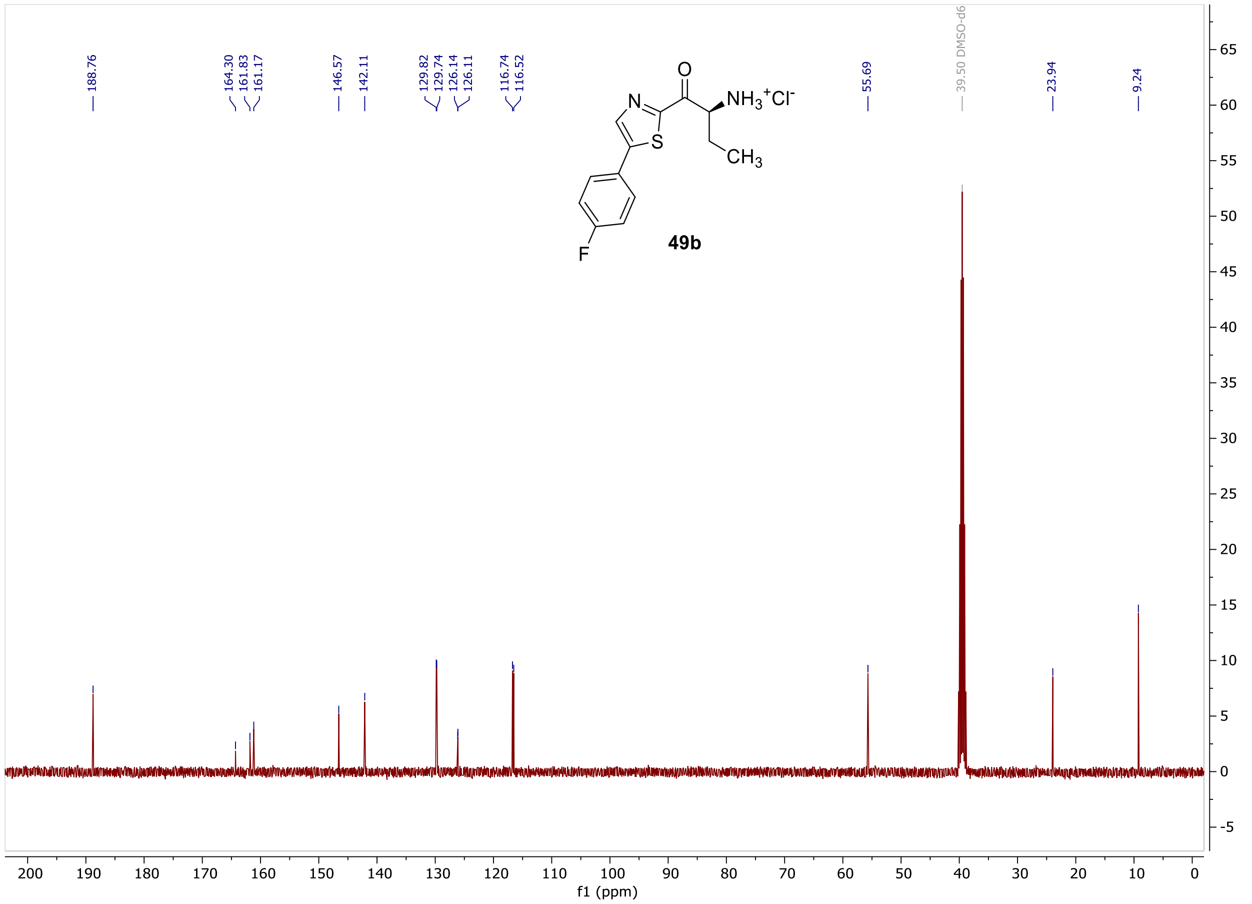

Compound **49b** (^{19}F NMR, 282 MHz):

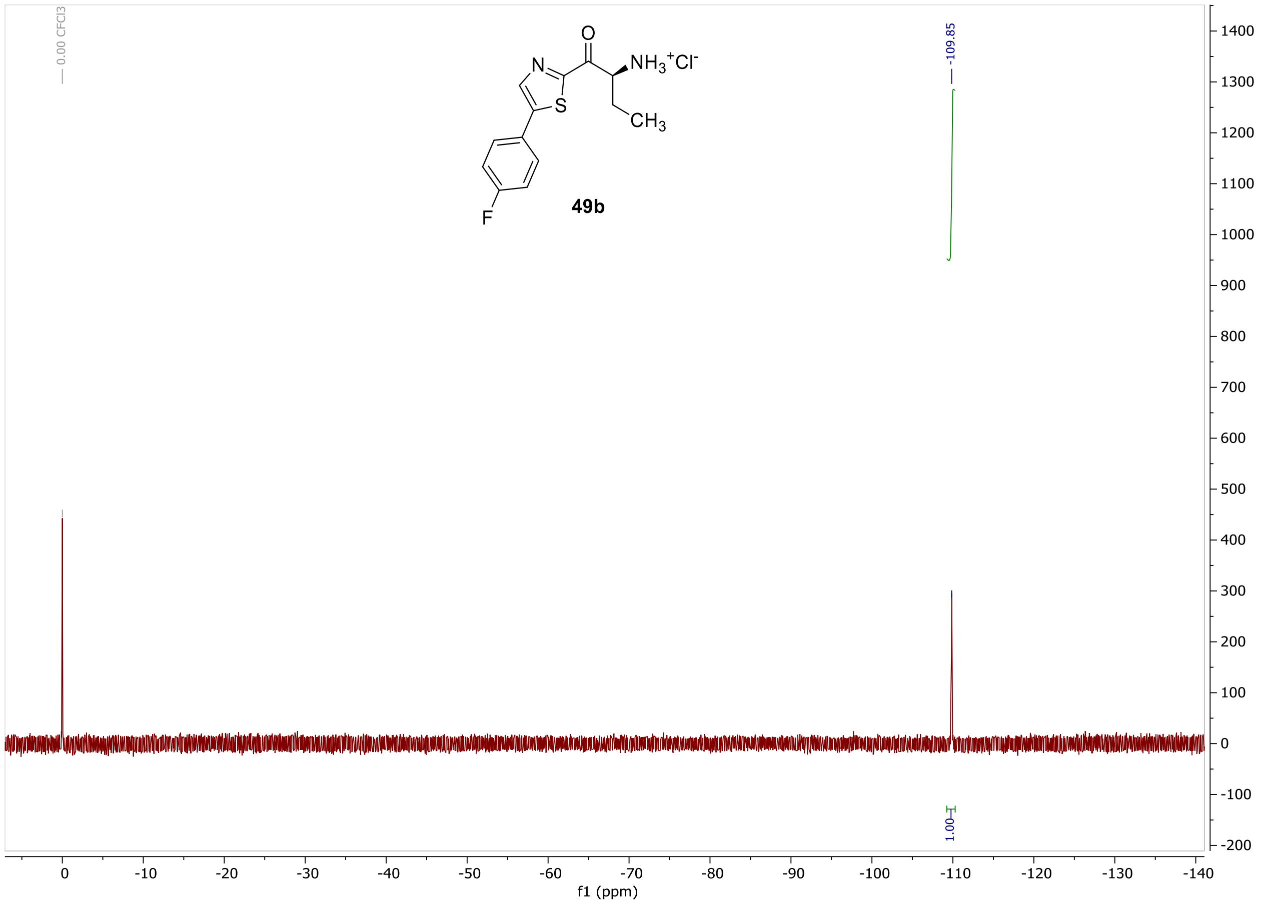

Compound **49c** (^{1}H NMR, 400 MHz):

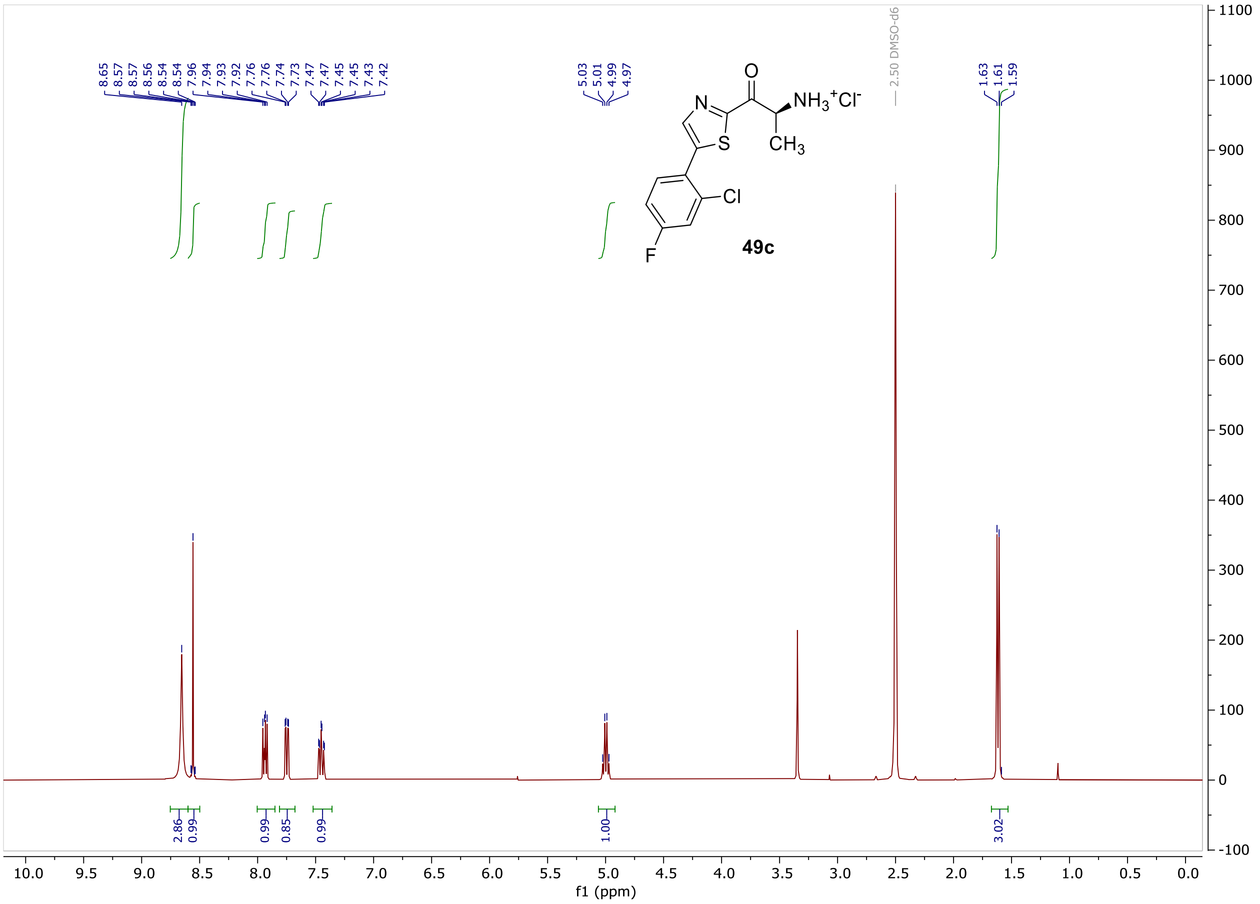

Compound **49c** (^{13}C NMR, 101 MHz):

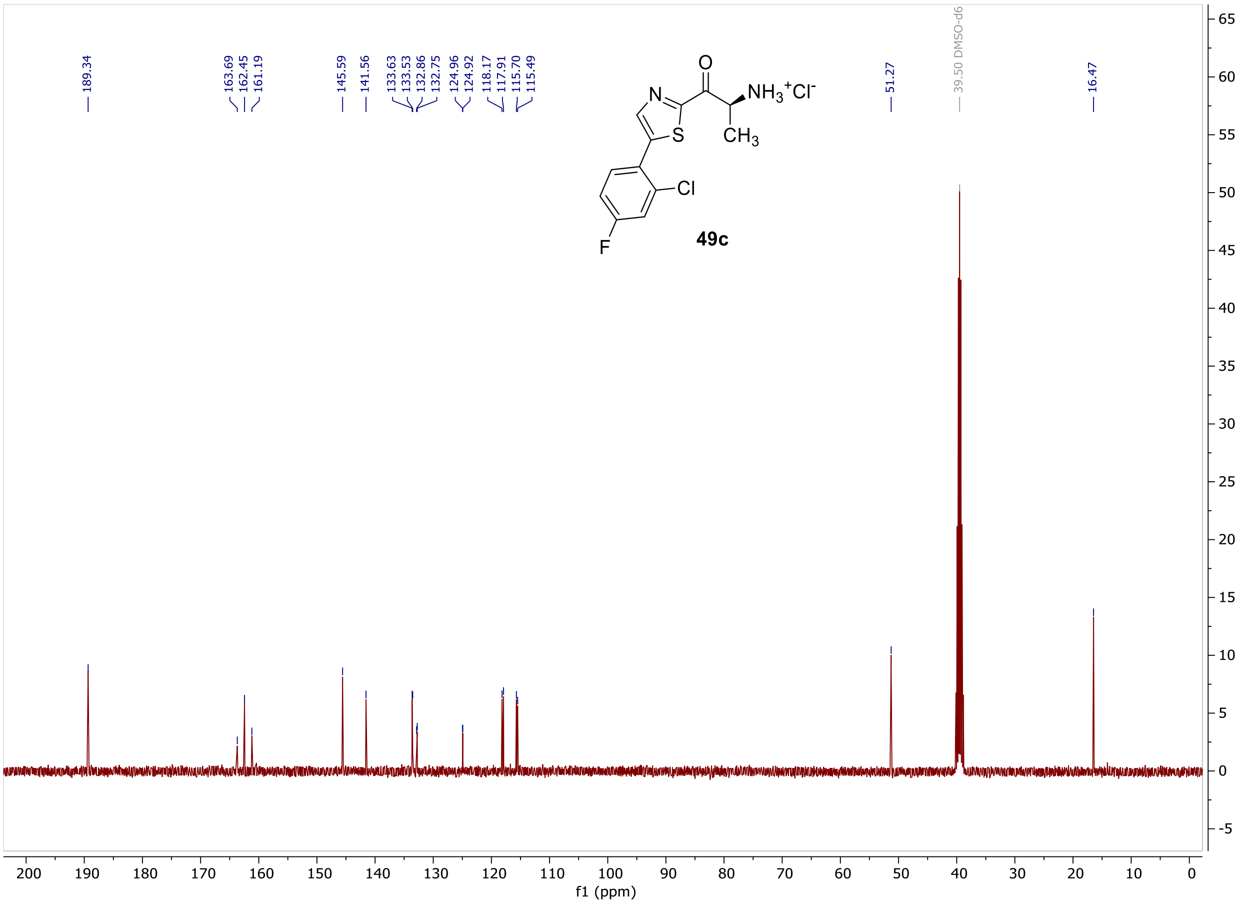

Compound **49c** (^{19}F NMR, 282 MHz):

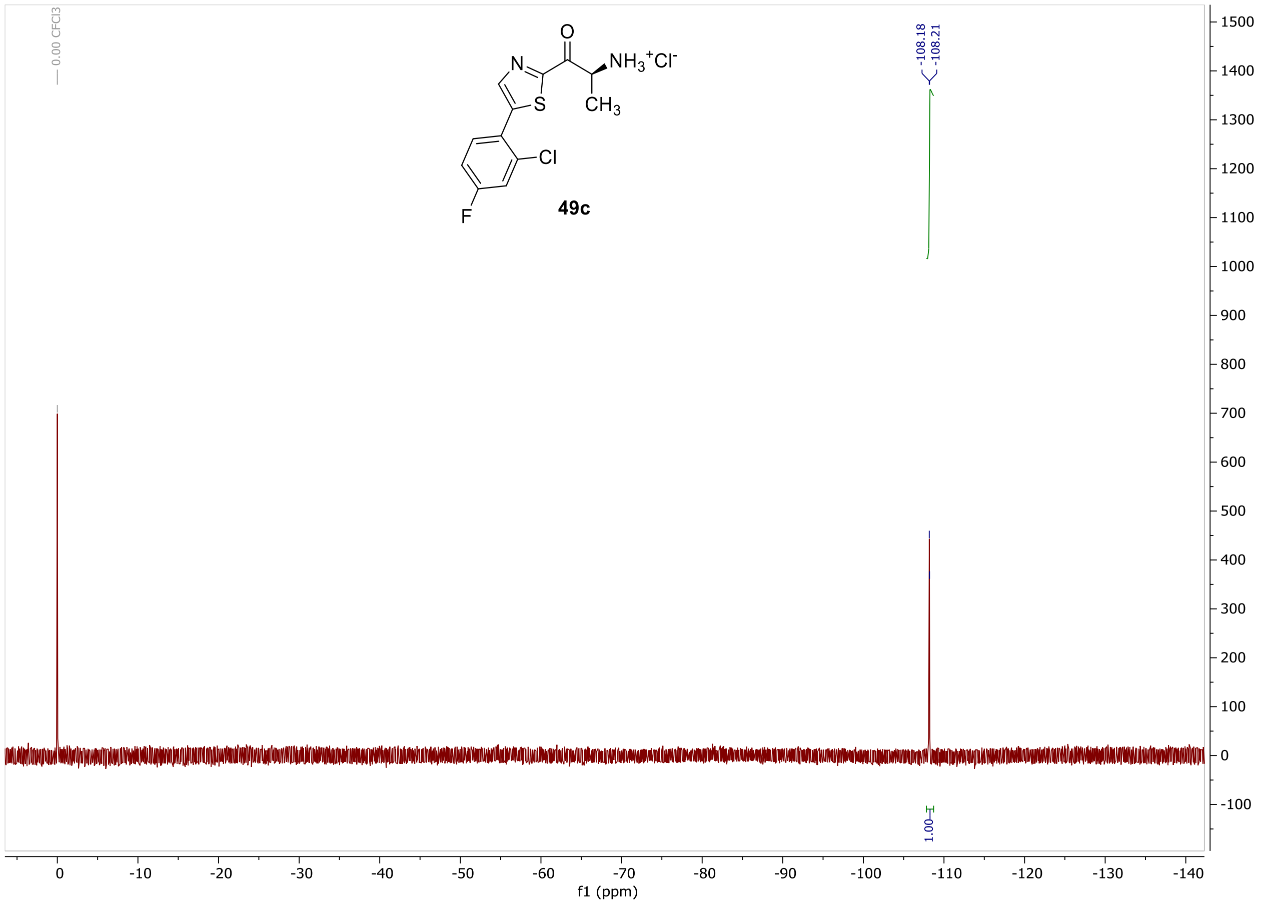

Compound **49d** (^{1}H NMR, 400 MHz):

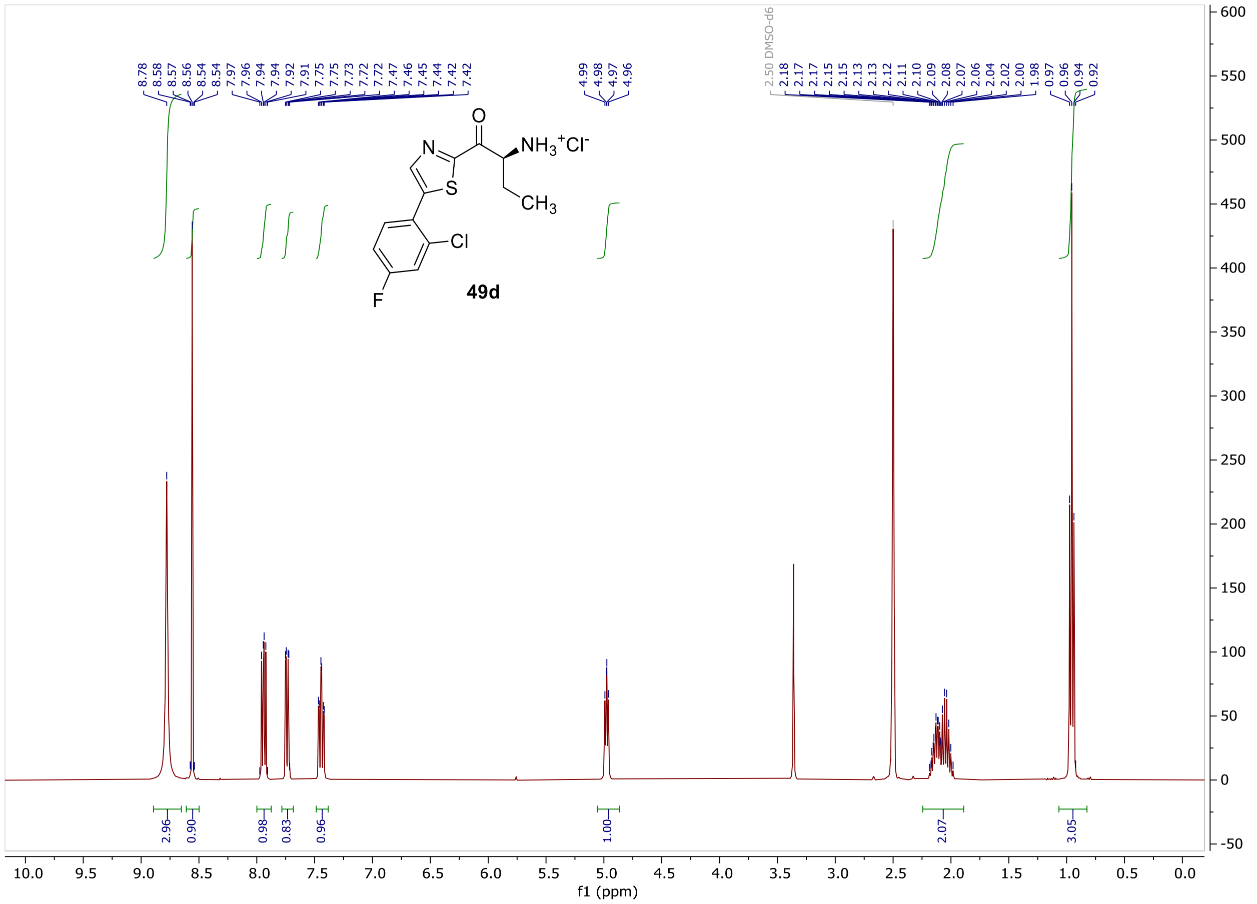

Compound **49d** (^{13}C NMR, 101 MHz):

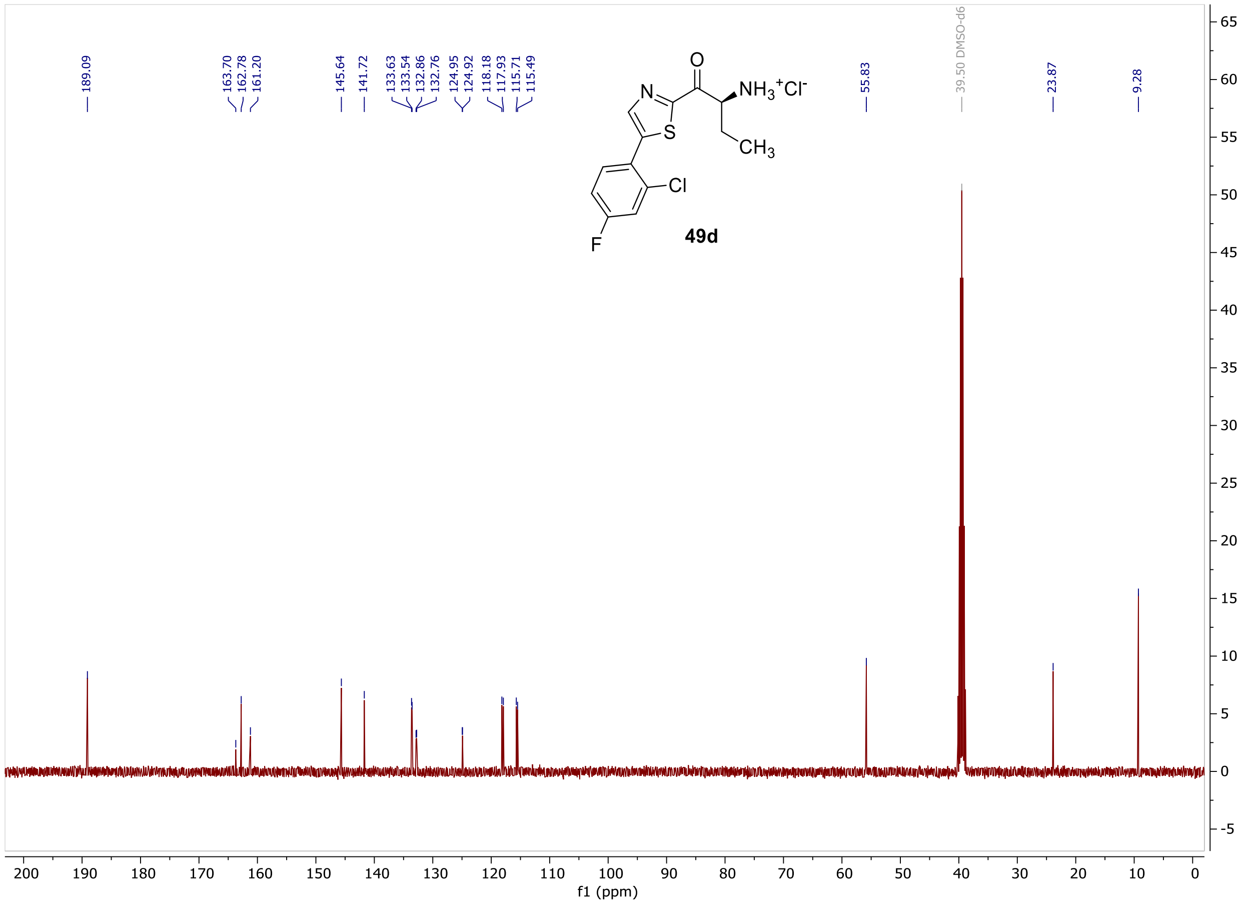

Compound **49d** (^{19}F NMR, 282 MHz):

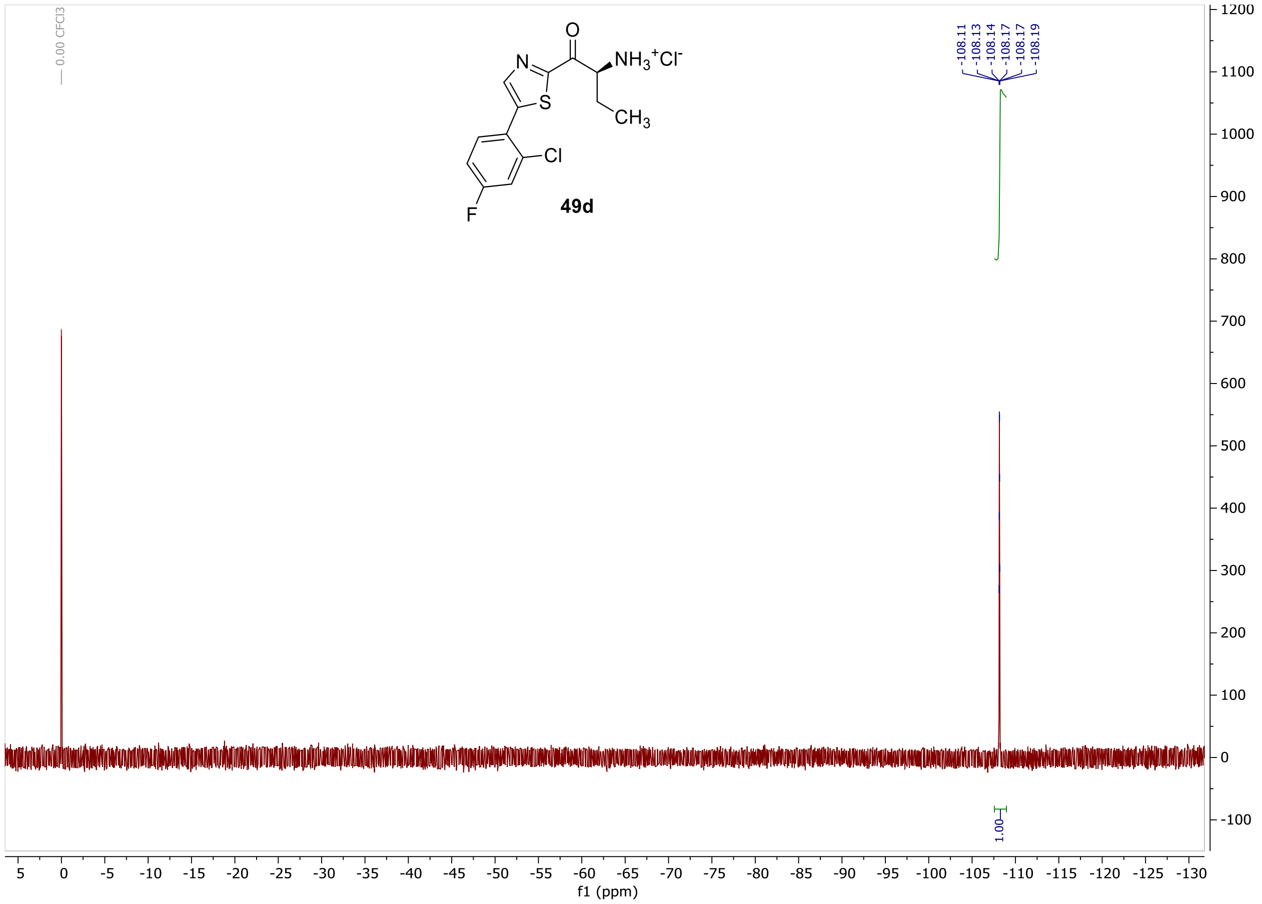

Supplement: Supplementary file 1 [file pharmaceuticals-14-01291-s001.zip › pharmaceuticals-1447267-supplementary/Supplementary_material_2-NMR.pdf]
